# Pre-Flight Calibration of the Mars 2020 Rover Mastcam Zoom (Mastcam-Z) Multispectral, Stereoscopic Imager

**DOI:** 10.1007/s11214-021-00795-x

**Published:** 2021-02-18

**Authors:** Alexander G. Hayes, P. Corlies, C. Tate, M. Barrington, J. F. Bell, J. N. Maki, M. Caplinger, M. Ravine, K. M. Kinch, K. Herkenhoff, B. Horgan, J. Johnson, M. Lemmon, G. Paar, M. S. Rice, E. Jensen, T. M. Kubacki, E. Cloutis, R. Deen, B. L. Ehlmann, E. Lakdawalla, R. Sullivan, A. Winhold, A. Parkinson, Z. Bailey, J. van Beek, P. Caballo-Perucha, E. Cisneros, D. Dixon, C. Donaldson, O. B. Jensen, J. Kuik, K. Lapo, A. Magee, M. Merusi, J. Mollerup, N. Scudder, C. Seeger, E. Stanish, M. Starr, M. Thompson, N. Turenne, K. Winchell

**Affiliations:** 1 Department of Astronomy, Cornell University, Ithaca, NY 14850 USA; 2 Cornell Center for Astrophysics and Planetary Science, Cornell University, Ithaca, NY 14850 USA; 3 Department of Earth, Atmospheric, and Planetary Sciences, Massachusetts Institute of Technology, Cambridge, MA 02139 USA; 4 School of Earth and Space Exploration, Arizona State University, Phoenix, AZ 85287 USA; 5 Jet Propulsion Laboratory, Pasadena, CA 91109 USA; 6 Malin Space Science Systems, San Diego, CA 92121 USA; 7 Niels Bohr Institute, University of Copenhagen, Copenhagen, Denmark; 8 USGS Astrogeology Science Center, 2255 N. Gemini Drive, Flagstaff, AZ 86001 USA; 9 Earth, Atmospheric, and Planetary Sciences Department, Purdue University, West Lafayette, IN 47907 USA; 10 Johns Hopkins Applied Physics Laboratory, Laurel, MD 20723 USA; 11 Space Science Institute, 4765 Walnut St., Suite B, Boulder, CO 80301 USA; 12 Joanneum Research Forschungsgesellschaft mbH, Steyrergasse 17, 8010 Graz, Austria; 13 Geology Department, Western Washington University, Bellingham, WA 98225 USA; 14 Geography Department, University of Winnepeg, 515 Portage Ave, Winnipeg, MB R3B 2E9 Canada; 15 Division of Geological and Planetary Sciences, California Institute of Technology, Pasadena, CA 91101 USA; 16 The Planetary Society, 60 S Los Robles, Pasadena, CA 91101 USA; 17 Centre for Terrestrial and Planetary Exploration, University of Winnipeg, 515 Portage Ave, Winnipeg, MB R3B 2E9 Canada

**Keywords:** Calibration, Camera, Mars

## Abstract

The NASA *Perseverance* rover Mast Camera Zoom (Mastcam-Z) system is a pair of zoomable, focusable, multi-spectral, and color charge-coupled device (CCD) cameras mounted on top of a 1.7 m Remote Sensing Mast, along with associated electronics and two calibration targets. The cameras contain identical optical assemblies that can range in focal length from 26 mm ($25.5^{\circ }\, \times 19.1^{\circ }\ \mathrm{FOV}$) to 110 mm ($6.2^{\circ } \, \times 4.2^{\circ }\ \mathrm{FOV}$) and will acquire data at pixel scales of 148-540 μm at a range of 2 m and 7.4-27 cm at 1 km. The cameras are mounted on the rover’s mast with a stereo baseline of $24.3\pm 0.1$ cm and a toe-in angle of $1.17\pm 0.03^{\circ }$ (per camera). Each camera uses a Kodak KAI-2020 CCD with $1600\times 1200$ active pixels and an 8 position filter wheel that contains an IR-cutoff filter for color imaging through the detectors’ Bayer-pattern filters, a neutral density (ND) solar filter for imaging the sun, and 6 narrow-band geology filters (16 total filters). An associated Digital Electronics Assembly provides command data interfaces to the rover, 11-to-8 bit companding, and JPEG compression capabilities. Herein, we describe pre-flight calibration of the Mastcam-Z instrument and characterize its radiometric and geometric behavior. Between April 26$^{th}$ and May 9$^{th}$, 2019, ∼45,000 images were acquired during stand-alone calibration at Malin Space Science Systems (MSSS) in San Diego, CA. Additional data were acquired during Assembly Test and Launch Operations (ATLO) at the Jet Propulsion Laboratory and Kennedy Space Center. Results of the radiometric calibration validate a 5% absolute radiometric accuracy when using camera state parameters investigated during testing. When observing using camera state parameters not interrogated during calibration (e.g., non-canonical zoom positions), we conservatively estimate the absolute uncertainty to be $<10\%$. Image quality, measured via the amplitude of the Modulation Transfer Function (MTF) at Nyquist sampling (0.35 line pairs per pixel), shows $\mathrm{MTF}_{\mathit{Nyquist}}=0.26-0.50$ across all zoom, focus, and filter positions, exceeding the $>0.2$ design requirement. We discuss lessons learned from calibration and suggest tactical strategies that will optimize the quality of science data acquired during operation at Mars. While most results matched expectations, some surprises were discovered, such as a strong wavelength and temperature dependence on the radiometric coefficients and a scene-dependent dynamic component to the zero-exposure bias frames. Calibration results and derived accuracies were validated using a Geoboard target consisting of well-characterized geologic samples.

**Supplementary Information:**

The online version contains supplementary material available at 10.1007/s11214-021-00795-x.

## Introduction

The Mast Camera Zoom (Mastcam-Z) instrument on the NASA Mars2020 rover *Perseverance* consists of a pair of zoomable and focusable digital CCD cameras (detectors, optics, and filter wheels) that can acquire multi-spectral (400–1100 nm), stereoscopic images of Mars with focal lengths ranging from 26 mm–110 mm. Externally mounted calibration targets enable relative reflectance calibration and two electronics boards in the rover body enable data processing and transmission of images to the rover’s central computer. The cameras are mounted atop a 1.7 m tall mast that enables them to be rotated $360^{\circ }$ in azimuth and $\pm 90^{\circ }$ in elevation.

The primary science objectives of the Mars2020 mission are to assess the present and past habitability of Jezero Crater on Mars, search for materials with high biosignature preservation potential and evidence of past life, obtain samples that are scientifically selected to represent the geologic diversity and potential habitability of the field site, and to contribute to the preparation for human exploration (Farley et al. 2020; Williford et al. 2018). Mastcam-Z is one of seven PI-led investigations on Mars2020 and provides observations essential to the completion of mission objectives in the form of visible color, multispectral, and stereo context images at pixel scales ranging from one hundred microns to tens of meters. The objectives of the Mastcam-Z investigation are to characterize the overall landscape geomorphology, processes, and nature of the geologic record at the Mars2020 rover field site, assess current atmospheric and astronomical conditions, events, and surface-atmosphere interactions, and provide operational support and scientific context for rover navigation, other Mars2020 instrument investigations including contact science, and sample selection, extraction, and caching (Bell et al. 2020). The success of these objectives, as well as the overall Mastcam-Z scientific investigation, requires delivery of well characterized and calibrated cameras to Mars.

Herein we describe a series of pre-flight component-level, stand-alone camera-level, and integrated rover-level tests and calibration activities performed in order to enable raw Mastcam-Z images to be geometrically and radiometrically calibrated following downlink to Earth. Calibration results are discussed in the context of best-practices and suggested operational strategies when fine radiometric accuracy is required. We also briefly describe the test sequences that will be performed with the cameras during cruise and on Mars in order to validate pre-flight calibrations, monitor for potential changes in the calibrations over time, and to enable additional in-situ relative reflectance calibration for more direct comparisons to laboratory reflectance spectra of rocks and minerals. More details about the Mastcam-Z instrument, and its science investigation, can be found in Bell et al. (2020, this journal). More details about the general goals of the Mars2020 mission, and the specific goals of other payload instruments also carried by the rover, can be found in Farley et al. (2020, this journal).

In addition to describing the results of the calibration in the main text of this article, the Calibration Plan and as-run Calibration Procedures and Logs are available in the Supplementary Online Material (SOM) of this manuscript. The SOM also contains the python scripts used to analyze the radiometric data and system spectral response profiles ($r_{\lambda ,k}$, see Sect. 3.5) for each filter in comma-separated values (CSV) text format. Including the scripts used to derive geometric model parameters and image quality via the Modulation Transfer Function (MTF) is, unfortunately, impractical due to the number of specialized software packages and human-in-the-loop steps required to reduce the geometric dataset. Calibration data, including relevant ancillary data (pressure, temperature, source identification, etc.), will be submitted to the Planetary Data System (PDS) within approximately 6 months of landing.

## Brief Instrument Description

### Camera Overview

**Fig. 1:**
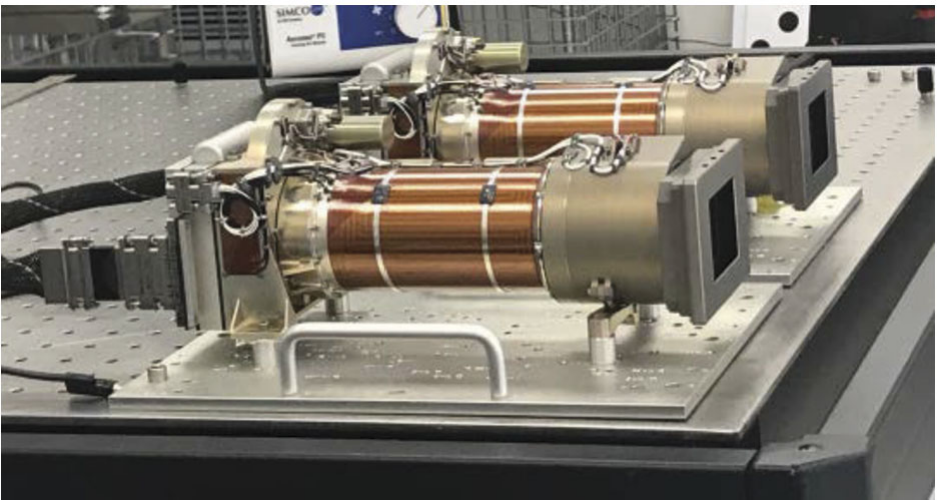
Image of the Mastcam-Z cameraheads on an optical bench in a cleanroom at Malin Space Science Systems (MSSS) acquired during stand-alone calibration. For scale, the screw holes on the optical tables are spaced 1 inch apart. During stand-alone calibration, the Mastcam-Zs were mounted up-side-down, parallel, and 9 inches (23.0 cm) apart

**Fig. 2:**
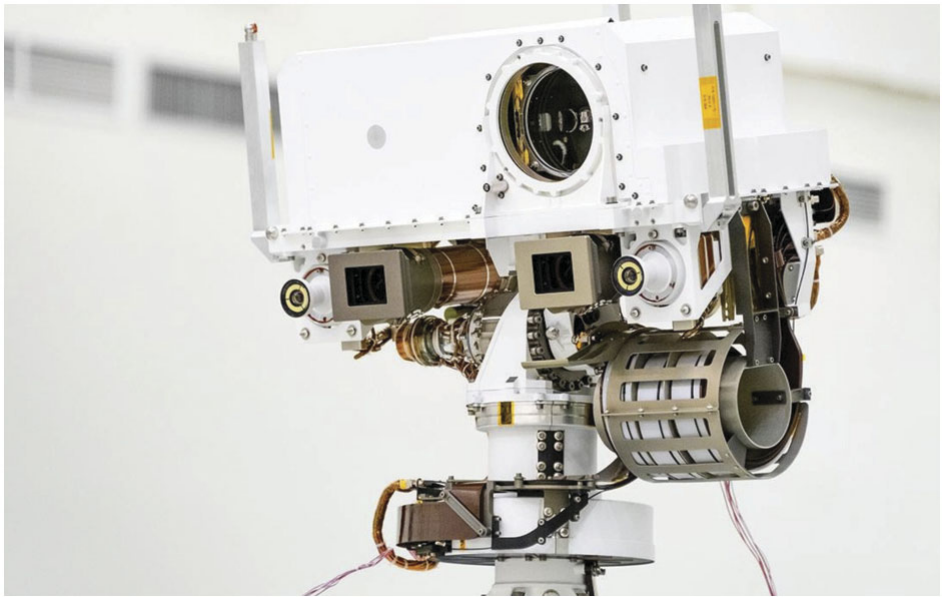
Image of the Mastcam-Z cameraheads integrated on the Mars2020 rover’s Remote Sensing Mast (RSM) acquired during Assembly, Test, and Launch Operations (ATLO) at the Jet Propulsion Laboratory (JPL). For scale, the stereo baseline between the two Mastcam-Z cameraheads is $24.3 \pm 0.1$ cm. Credit: NASA/JPL-Caltech

Mastcam-Z (Bell et al. 2020) consists of two zoomable color cameras mounted on the rover’s Remote Sensing Mast (RSM). Each instrument consists of a Digital Electronics Assembly (DEA) within the rover Warm Electronics Box (WEB) and a camerahead (Figs. 1 and 2) that is independently capable of focus and zoom. The cameraheads are mounted 24.3 cm apart on the RSM and each consist of an optomechanical lens assembly and focus plane array (FPA) with associated electronics. The Mastcam-Z optomechanical lens assembly is a simplified version of the original zoom/focus/filter wheel assembly developed, but later descoped and not flown, for the Mars Science Laboratory (MSL) Mastcam. The FPAs and DEAs are built-to-print copies of the same assemblies on MSL Mastcam. Mastcam-Z will provide panoramic, stereoscopic, color, and multi-spectral (400–1100 nm) images and selected mosaics of the Martian surface.

**Fig. 3:**
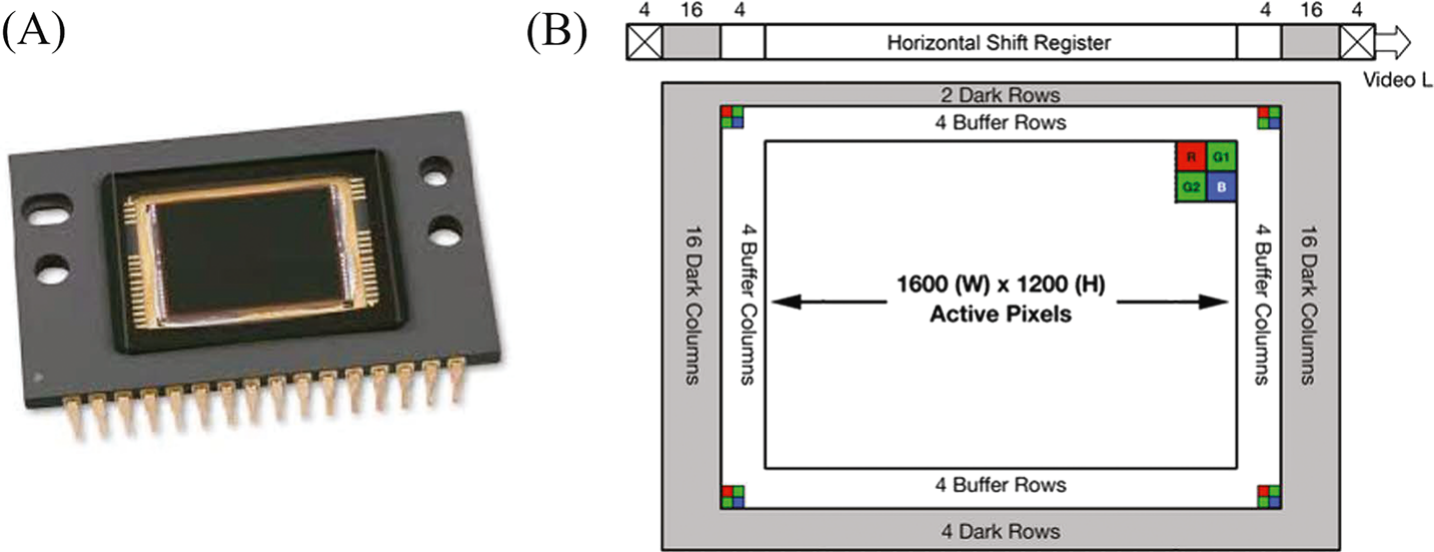
(**A**) Vendor image of the ON Semi KAI-2020 detector; (**B**) Simplified schematic of the Kodak KAI-2020 interline transfer CCD. Each pixel is overlain by a Bayer pattern filter, shown as the $2\times 2$ colored cells, bonded directly to the CCD’s active pixels. In this view, pixel clocking is up and to the right (first pixel read out is G1). The active region consists of $1600\times 1200$ pixels surrounded by buffer pixels, dark shielded pixels, and the horizontal shift register. Note that the Bayer pattern pixels are not shown to scale in the image. Note that the schematic is drawn looking down on the detector. Images are displayed from the perspective of the detector looking out. Therefore, while the readout and pixel $(1,1)$ is in the upper-right of this schematic, they are instead in the upper left of the camera images displayed throughout this manuscript. Images are adapted from the online vendor manual (https://www.onsemi.com/pub/Collateral/KAI-2020-D.eps)

Each Mastcam-Z camera system is optically and electronically identical to and autonomous from each other in terms of mechanical packaging, power, and command and data handling (C&DH). Each camerahead consists of a lens assembly with a 4:1 compound zoom design, focus mechanism, filter wheel mechanism (with three spectral filters replicated and four differing between the filter wheels), Kodak KAI-2020CM interline transfer CCD detector (Fig. 3), and electronics to drive the CCD clocks and digitize the video signal. The CCDs have $1600 \times 1200$ active pixels and are capable of relatively high frame rate acquisitions to produce “video” at ∼4 frames/sec. Video will typically be acquired in HD-format, $1280 \times 720$ pixels. Characteristics of the Mastcam optics and detector that are useful in the calibration, analysis, and interpretation of data products are described in Table 1.

**Table 1:** Mastcam-Z instrument electronic and optical characteristics

Characteristic		
Focal Length	26.16 mm (Zoom 0000 mc) to 109.9 mm (Zoom 9600 mc)	
F-Number	f/7 (26 mm) to f/9 (110 mm)	
Field of View (FOV)	25.6^∘^ × 19.2^∘^ (26 mm) to 6.18^∘^ × 4.63^∘^ (110 mm)	
Instantaneous FOV (IFOV)	283 μrad (26 mm) to 67.4 μrad (110 mm)	
Spatial Scale (m/pixel)	148-540 μm at 2 m, 7.4-27 cm at 1 km range	
Baseline Stereo Seperation	24.3 ± .1 cm	
Focus Range	∼0.5 m–∞ (26 mm) to ∼1.0 m–∞ (110 mm)	
Number of Spectral Filter	Seven Plus the RGB Bayer Pattern (See Table 2)	
CCD Pixel Pitch	7.4 μm × 7.4 μm	
CCD Pixel Format	1640 (H) × 1214 (V), 1600 (H) × 1200 (H) Active	
CCD Detector Gain	15.6 ± 0.20 $\frac{e^{-}}{DN}$ (Left)	15.6 ± 0.40 $\frac{e^{-}}{DN}$ (Right)
CCD Detector Read Noise	22 ± 3 $e^{-}$ (Left)	21 ± 5 $e^{-}$ (Right)
CCD Detector Full Well	21,827 ± 107 $e^{-}$ (Left)	21,791 ± 200 $e^{-}$ (Right)
CCD Linearity	Non-linearity <0.6%, see Sect. 3.3.1	

The Mastcam-Z optical zoom assembly is an all-refractive design consisting of one moving focus lens group, two moving zoom lens groups, three stationary lens groups, and a plano element (spectral filters). Each lens provides fields of view between $6^{\circ }\times 5^{\circ }$ (110 mm f/9.5) and $26^{\circ } \times 19^{\circ }$ (26 mm f/7) and can focus as close as 1 m for focal lengths up to 50 mm and as close as 2 m for focal lengths greater than 50 mm and less than 100 mm. The overall camera system design is required to enable a Modulation Transfer Function (MTF) of greater than 0.2 at the Nyquist frequency of the detector (47 l.p./mm or 0.35 l.p./pixel) over the full range of focal lengths for targets greater than 2 m distance, including the near IR bands. As described in Sect. 3.8, MTF at Nyquist is $> 0.26$ for all filters across all zoom level, and $> 0.4$ for the L0/R0 across all zoom levels (see Table 8). Herein, we adopt the definition of Nyquist for a color Bayer imaging system, as described in Bell et al. (2017), which differs from the standard 0.5 l.p./pixel of a monochrome imaging system.

The CCD provides RGB color by means of a Bayer pattern filter; the weighted spectral response of which is shown in Fig. 20. Each Mastcam-Z camerahead has an IR-cutoff filter (L0/R0) for color imaging, and a set of narrowband filters for multispectral science imaging (see Sect. 3.5). The bandpasses of the narrowband filters are similar to the filters used on the Mars Exploration Rover (MER) Pancam (Bell et al. 2003) and MSL Mastcam (Malin et al. 2017) instruments (Table 2, Fig. 20), although the location of the filters in the filter wheels has been updated between Mastcam and Mastcam-Z to keep the shorter wavelength filters in the left Mastcam-Z and longer wavelengths filters in the right Mastcam-Z for operational simplicity and increased fidelity of calibrated reflectance spectra (see Bell et al. 2020; Kinch et al. 2020, for additional detail). This arrangement is similar to that implemented for MER Pancam. The 805 nm filters (R1/L1) are included in both Mastcam-Zs to facilitate stereo imaging. Narrowband multispectral imaging with Mastcam-Z is accomplished through the superposition of the narrowband filters on the red, green, and blue microfilters of the Bayer pattern CCD (see Sect. 3.5). The spectral bandwidths described in Table 2 are based on the system throughput testing described in Sect. 3.5.

**Table 2:** Mastcam-Z Effective Wavelengths^a^, Band Passes^b^, Spectral Leakage^c^, and Reference DN Level^d^ ($F_{\mathit{ref}}$)

Left Mastcam-Z				Right Mastcam-Z			
Filter	$\lambda _{\mathit{eff}} \pm \mathit{HWHM}$ (nm)	Leakage	$F_{\mathit{ref}}(DN)$	Filter	$\lambda _{\mathit{eff}} \pm \mathit{HMWM}$ (nm)	Leakage	$F_{\mathit{ref}}(DN)$
L0R	630±43	0.43%	6185	R0R	631±43	0.4%	6151
L0G^e^	544±41	0.26%	7212	R0G^e^	544±42	0.36%	6984
L0B	480±46	1.1%	6834	R0B	480±46	1.2%	6821
L1^f^	800±9	0.66%	392	R1^f^	800±9	0.63%	396
L2^f^	754±10	0.6%	596	R2	866±10	0.62%	219
L3^f^	677±11	0.84%	1171	R3	910±12	0.61%	165
L4^f^	605±9	0.67%	1250	R4	939±12	0.64%	114
L5^f^	528±11	0.46%	1645	R5	978±10	0.63%	47
L6^f^	442±12	1.0%	1425	R6	1022±19	0.48%	59
L7	590±88,ND6^g^	–	–	R7	880±10, ND5^g^	–	–

^a^Effective Wavelength ($\lambda _{\mathit{eff}}$) is defined as the weighted average of wavelength with the normalized system spectral response ($\bar{r_{\lambda ,k}}$, see Sect. 3.5) and solar radiance at the top of the atmosphere ($S_{o_{ph}}$) in units of [$\frac{ph}{s\, m^{2}\, sr\, nm}$]: $\lambda _{\mathit{eff}}=\frac{\int \bar{r_{\lambda ,k}} S_{o_{ph}} \lambda d\lambda}{\int \bar{r_{\lambda ,k}} S_{o_{ph}}d\lambda}$. The system spectral response includes the effects of the optics, filters, and detector quantum efficiency (see Sect. 3.5)

^b^Bandpasses are provided as the Half-Width-Half-Maximum (HWHM), which is defined as one-half the difference between the two wavelengths at which the response is half of the peak in-band response

^c^Spectral Leakage is defined as the percentage ratio of the integrated out-of-band response to the integrated in-band-response of $\bar{r_{\lambda ,k}}$. For purposes herein, the in-band to out-of-band cutoff is defined as the closest wavelengths on either side of $\lambda _{\mathit{eff}}$ that correspond to 1% of the peak in-band response

^d^The reference DN level ($F_{ref,k}$) is defined as the above-bias signal of a 10 ms exposure observing, through filter *k*, a perfectly diffuse and white sunlit surface on Mars at zero-incidence when Mars is at perihelion (1.38 AU) and ignoring atmospheric attenuation (see Sect. 4.1.6)

^e^There are two identical green filters per 2 × 2 Bayer unit cell (see Fig. 3)

^f^Filters where the narrowband response partially or completely blocks one or more of the Bayer RGB filter responses

^g^NDX refers to a $10^{-X}$ neutral density coating for solar imaging

The $24.3\pm .1$ cm boresight separation between the cameraheads, when mounted on the RSM, is a compromise between the need to minimize the instrument stereo baseline to provide human-eye-fusible stereo and the need to maximize the baseline to provide better stereo resolution at distance. Similar to the MSL Mastcam and MER Pancam instruments, the Mastcam-Z cameraheads are mounted on the RSM with their optic axes tilted toward each other by a “toe-in” angle of $1.17^{\circ }\pm .03^{\circ }$ per camera. Within this document we refer to camera S/N ID 1 as the “left camera” and camera S/N ID 2 as the “right camera”. Note that, during stand-alone calibration, the cameras were mounted $180^{\circ }$ relative to their orientation on the rover, so the left camera was actually on the right side of the optics bench when looking down the camera boresight (on the RSM, the cameras are mounted in a hanging configuration). Additional details regarding the Mastcam-Z instrument and science investigation can be found in Bell et al. (2020, this journal).

### Mastcam-Z Calibration Targets

Mastcam-Z relies on a set of radiometric calibration targets to verify and monitor instrument calibration and to provide an instantaneous estimate of local illumination conditions in order to allow conversion of images from units of radiance (the instrument observable) to units of reflectance (the material property). There are both *Primary* and *Secondary* calibraiton targets. Both are mounted on the rover deck and visible to the Mastcam-Z. The targets are described in detail in Kinch et al. (2020, this journal).

**Fig. 4:**
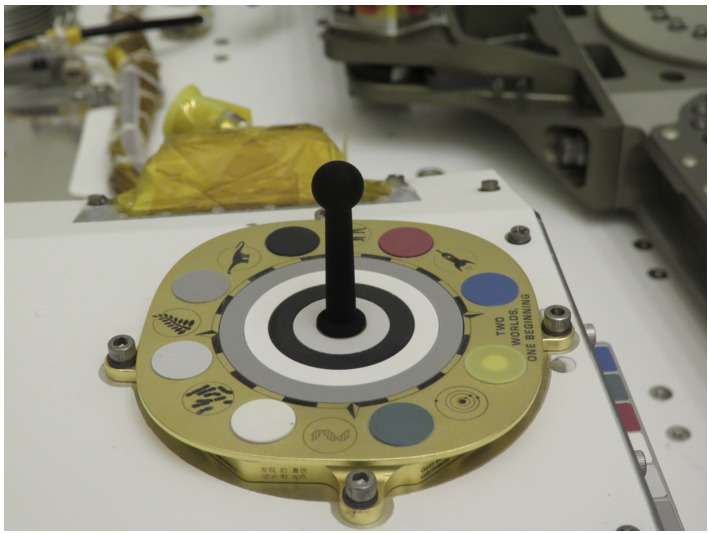
Mastcam-Z primary calibration target on the top of the RPFA box imaged during ATLO inspections at Kennedy Space Center in March 2020. The base of the target fits inside an $80\times 80$ cm envelope (see Kinch et al. 2020, this journal)

The primary calibration target (Fig. 4) combines elements of the designs of camera calibration targets from the Mars Exploration Rovers (Bell et al. 2003), Phoenix (Leer et al. 2008), and Mars Science Laboratory (Malin et al. 2017) missions. The body of the target is constructed of gold-anodized aluminum with a central shadow post painted with an IR-black coating. Mounted in the aluminum frame are four central grayscale rings and eight outer circular color and grayscale patches made from ceramic materials with well-characterized reflectance properties. Underneath the 8 color and grayscale patches around the periphery are strong permanent magnets designed to attract even weakly magnetic martian dust particles, thereby keeping the center of each patch relatively free of dust (Kinch et al. 2020). Constraining the amount of dust that falls onto each patch requires careful generation of a post-landing baseline as well as implementation of a long time-series of self-consistent observations. A quantitative answer to the amount of dust deposited on Martian calibration targets has been surprisingly hard to derive from previous missions (Madsen et al. 2009) and is one of the goals of the Mastcam-Z investigation. The top surface and sides of the primary calibration target carries an engraved motto, graphics, and an inspirational message for public outreach. The total mass of the primary target is 103 g and it is mounted on top of the Rover Pyro Firing Assembly (RPFA) on the rear starboard side of the vehicle.

**Fig. 5:**
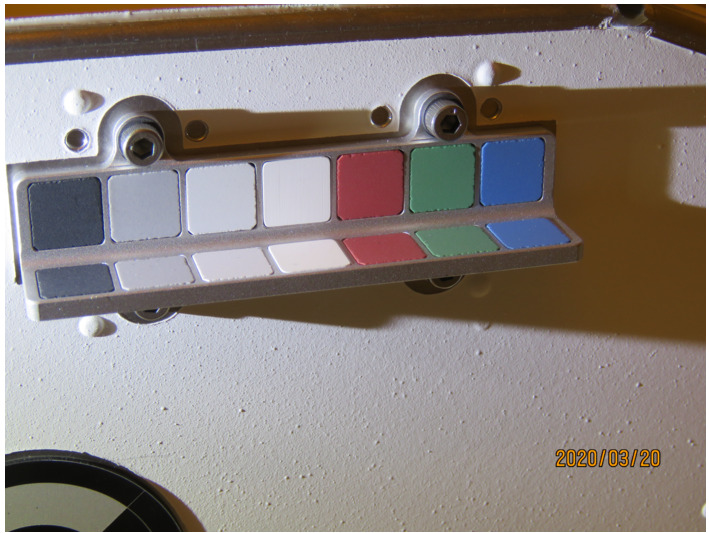
Mastcam-Z secondary calibration target on the front face of the RPFA box imaged during ATLO inspections at Kennedy Space Center in March 2020. For scale, the distance between the mounting bolts above the target is 44 mm (see Kinch et al. 2020, this journal)

The secondary calibration target (Fig. 5) is a simple angled “shelf” frame of bead-blasted aluminum holding a total of 14 optical patches. Four distinct grayscales and three distinct colors are each included twice, once on a vertical surface and once on a horizontal surface. The colors and grayscales are identical to the colors and grayscales on the primary target and made from the same materials, but the overall design is significantly simpler with no embedded magnets or gnomon. The total mass of the secondary target is 15 g and it is located directly below the primary target on the vertical front face of the RPFA box. This vertical mounting positions the secondary target in a different dust deposition environment from the primary target both during landing and during surface operations, while still allowing observations of the secondary target to be in the same image frame as the primary target.

**Fig. 6:**
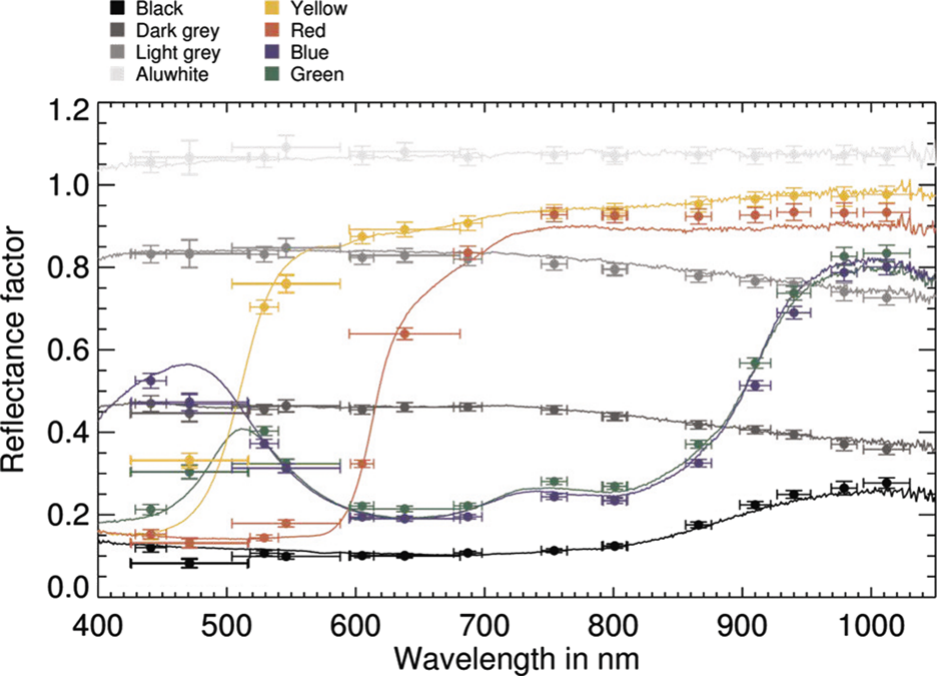
Laboratory reflectance (lines, $R^{*}_{lab}$) at incidence $=0^{\circ }$, emission $= 0^{\circ }$, and calibrated Mastcam-Z $R^{*}$ values (dots with error bars) of calibration target witness samples derived from 34 mm observations of the Geoboard made during stand-alone testing (see Sect. 3.12). For details on laboratory characterization of calibration target color and grayscale materials see Kinch et al. (2020, this journal). Mastcam-Z $R^{*}$ values were derived using the techniques described in Sect. 4.1.7

The spectral reflectance of the color and grayscale materials in the two targets can be seen in Fig. 6, which also overplots calibrated Mastcam-Z relative reflectance ratios ($R^{*}$) of witness samples of these materials contained in Geoboard images acquired during stand-alone calibration (see Sect. 3.12). The calibrated reflectance ratios were derived using the techniques outlined in Sect. 4.1 and show root-mean-square (RMS) differences of 6.4% and 3.6% relative to the laboratory spectra for the broadband and narrowband filters, respectively. The larger variance for the broadband filters is the result of not accounting for the non-solar spectral shape and complex illumination conditions of the multiple light sources used during testing (see Sect. 3.12). The bidirectional reflectance functions of all calibration target materials were carefully characterized during pre-flight testing. These characterizations, together with details of the design, manufacture and testing of the calibration targets, can be found in Kinch et al. (2020, this journal).

### Integration with Rovers

**Fig. 7:**
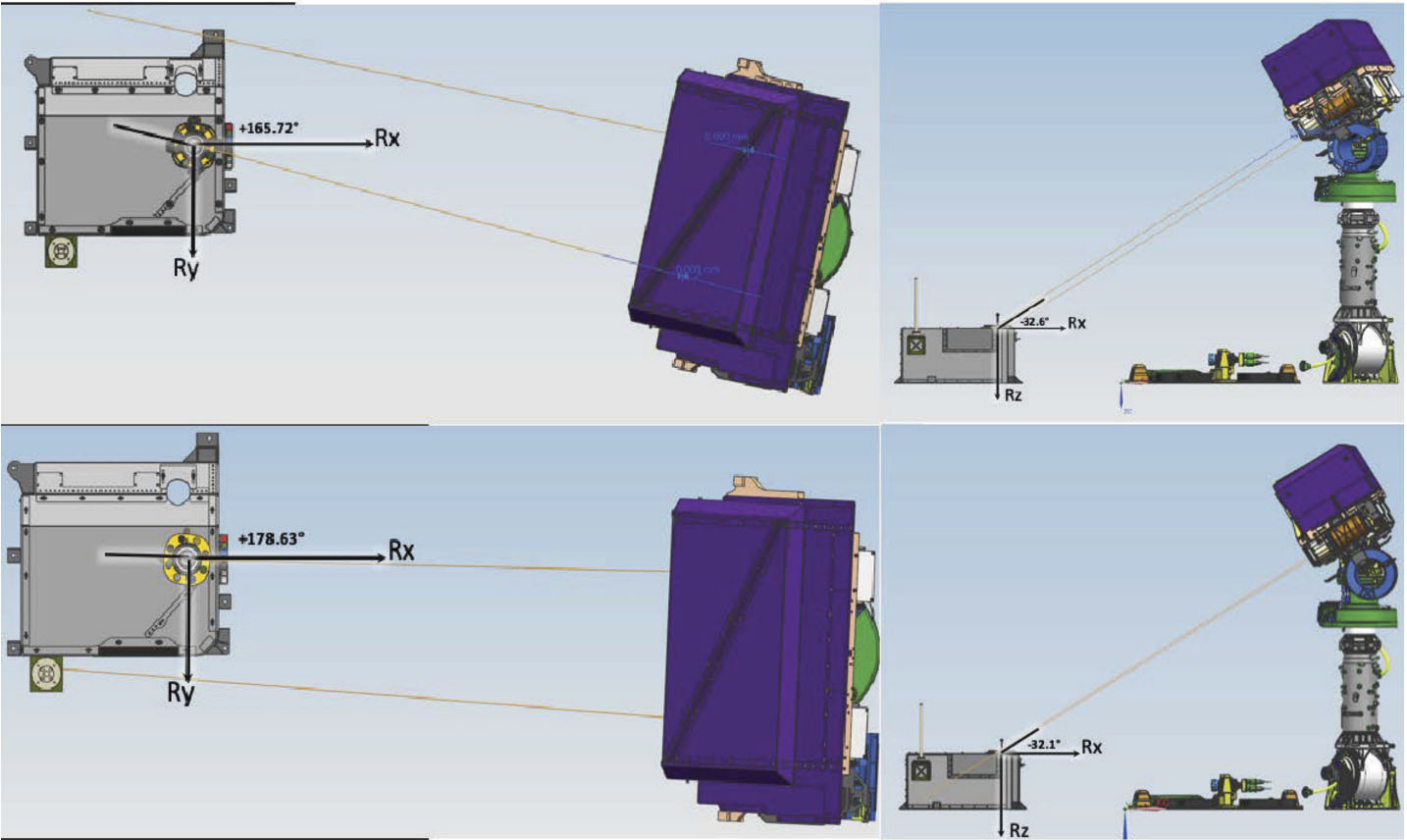
Schematic showing position of the Mastcam-Z left (top) and right (bottom) cameraheads when pointing at the Primary Flight Calibration Target. Azimuth and elevation pointing values are depicted in Rover Frame.

The Mastcam-Z cameras are mounted on the RSM at a height of 211.6 cm above the Martian surface (see Fig. 7). The left and right camera boresights are separated by $24.3\pm .1$ cm with a $1.17^{\circ } \pm .03^{\circ }$ toe-in per camera, and are positioned 12.7 cm to the left and 12.2 cm to the right, respectively, of an azimuth actuation axis that is located on the front right corner of the rover, 55.9 cm starboard of the vehicle’s centerline. Elevation actuation of both cameras occurs along an axis that is located 8.0 cm below the camera boresights or 191.9 cm above the surface. The nominal location of the left Mastcam-Z in the rover navigation frame is XYZ = (+107.57, +43.17, −211.64) centimeters.

## Pre-Flight Testing and Calibration

### Introduction and Philosophy

The primary goals of Mastcam-Z pre-flight testing and calibration were to develop a detailed understanding of instrument performance under a range of environmental conditions relevant to Mars, validate pre-assembly predictions of instrument performance so that models could be constructed to interpolate or extrapolate expected performance to conditions where pre-flight testing was not possible, and to acquire sufficient data to enable the conversion of measured Data Number (DN) to an accurate estimate of the incident physical radiance ($\frac{W}{m^{2} sr}$) integrated across each filter’s bandpass.

At the stand-alone instrument level, a series of tests were conducted between April 26$^{th}$ and May 9$^{th}$, 2019, at Malin Space Science Systems (MSSS) in San Diego, CA. The flight camera systems (fully assembled) were driven by Ground Support Equipment (GSE) designed to simulate their respective DEAs (see Sect. 3.2.3). Some geometric tests were also conducted during ATLO at the Jet Propulsion Laboratory from July 22$^{nd}$–23$^{rd}$ and October 19$^{th}$–30$^{th}$, 2019, and at the Kennedy Space Center on March 19$^{th}$, 2020. Collectively, these tests were designed to characterize Mastcam-Z’s radiometric and geometric properties. For a detailed description of test parameters and conditions, see the Mastcam-Z Calibration Plan (JPL Document D-101345) provided in the SOM of this manuscript. By the completion of calibration activities, $\sim 45,000$ images had been acquired (see Table 3).

**Table 3:** Number of images acquired by test for stand-alone calibration at MSSS and ATLO calibration at JPL and KSC

Test description	Script IDs^a^	Date(s)	Left camera	Right camera
Photon Transfer (Ambient)	412TAMB	May 01,02	905	900
Photon Transfer (TVAC)	413TN10	April 27	180	180
Radiometric Testing (Ambient)	423TAMB	May 06	2267	2193
Radiometric Testing (TVAC)	425TN10	April 27	786	815
Solar Radiometric Testing (TVAC)	426TN10	April 27	52	45
Spectral Throughput (Ambient)	433TAMB	May 05,06	8908	14432
Dark Current (Ambient and TVAC)	441TEMP	April 26	179	245
JPL-Method Geometric Cal. (Ambient)	462TAMB	May 02	382	383
Photogrammetric Geo. Cal. (Ambient)	468TEMP	May 04,07	328	329
Photogrammetric Geo. Cal. (TVAC)	465TEMP	April 28	148	150
Affine Transform Cal. (Ambient)	466TAMB,467TAMB	May 02,03	1940	1912
Affine Transform Cal. (TVAC)	466TEMP,467TEMP,468TEMP	April 28,29	560	537
Macbeth Color Target (Ambient)^b^	466TAMB,466TEMP	May 01	238	238
MTF Calibration (Ambient)	471TAMB	May 03-07	920	1022
MTF Calibration (TVAC)	473TEMP,491TN10,492TN10	April 27	203	383
Stray Light (Ambient)	481TAMB	May 07	29	34
Geoboard, Caltarget (Ambient)	491TAMB,471TAMB	May 08	383	647
Team Portrait (Ambient)	GROUP	May 08	7	11
Misc. Temperature Ramps (TVAC)^c^	zf1_*, zf2_*	April 26,27	354	355
Misc. Auto-focus text files^d^	af_*	April 26 -May 08	336	333
JPL ATLO Geo. Cal. (Ambient)^e^	ZL_*, ZR_*	July 22,23	251	250
JPL ATLO Geo. Cal. (TVAC)^f^	ZL_*, ZR_*	Oct. 19-30	52	52
KSC ATLO Geo. Cal. (Ambient)^g^	ZL_*, ZR_*	March 19, 2020	20	22
Total Image Count	**44896**			

^a^Script ID’s refer to Calibration Plan’s section numbers where the test is described. The “TAMB” refers to ambient (room) temperature, “TN10” means −10^∘^C temperature, and “TEMP” stands for various temperatures

^b^The 116-patch Macbeth target was imaged in a thermal/vacuum (TVAC) chamber at ambient conditions with and without the TVAC chamber’s window (under identical illumination). These data offer constraints on the window’s spectral transitivity. The standard 24-patch Macbeth was imaged in the Geoboard tests (491TAMB)

^c^The temperature ramps are the verification and validation tests where the Mastcam-Zs stared at a dot/MTF hybrid target while the TVAC changed temperatures. This data set includes stare tests with and without autofocus

^d^The autofocus files give the focus motor-count and JPEG compression size of each trial image taken in the autofocus routine. Apart from the “af_” prefix, the filename is the same as the image captured after the autofocus routine finds best-focus

^e^After installation on the Mars2020 rover, the Mastcam-Zs were geometrically calibrated at ambient with the NavCams, HazCams, and the SuperCam RMI. These tests are colloquially called the “Calipalooza”

^f^The thermal tests comprise stare tests during several temperature ramps

^g^The “closeout” images were taken at the Kennedy Space Center (KSC) to validate the camera-models produced from the JPL ATLO tests^e^

#### Radiometric Testing

Each of the radiometric tests performed during stand-alone calibration were targeted at understanding a parameter in the camera equation that converts the Digital Number ($DN$) reported by the imaging system into physical units of incident radiance. The camera response ($DN$) is proportional to the incoming radiance at the front aperture ($L_{\lambda }$), weighted by the spectral response ($r(\lambda )$) of the instrument. We have adopted a form of the camera equation that relates the measured digital-number (DN) to Mastcam-Z’s optical parameters and detector properties:

1 $$ DN_{ijkl} = \frac{ A_{o}\varOmega _{l}\,(t+t_{sm,ij})}{ g \, F_{ijkl} } \, r_{o,\mathit{ijk}} \int _{\lambda _{1}}^{\lambda _{2}} \bar{r}_{\lambda ,k} L_{ \lambda } \frac{\lambda }{hc} d\lambda + B_{ij} + (t+t_{sm,ij}) \frac{D_{ij}}{g} + N_{ij}, $$

where $DN_{\mathit{ijkl}}$ is the 11-bit digital-number of pixel $(i,j)$ using filter $k$ and zoom position $l$. The etendue, or optical throughput, ($A_{o} \varOmega _{l}$ $[m^{2}sr]$) is defined as the product of the collecting area ($A_{o}$) and square of the instantaneous field of view ($\varOmega =\mathit{IFOV}^{2}$) at zoom setting $l$. Detector-specific properties include the gain ($g$ $[ \frac{e^{-}}{DN}]$), static bias ($B_{ij}$ $[DN]$), and dark current ($D_{ij}$ $[ \frac{e^{-}}{s}]$). In addition to the exposure time ($t$ $[\mathrm{s}]$), $t_{sm,\mathit{ijk}}$ $[\mathrm{s}]$ is a filter-dependent correction to the commanded exposure that accounts for a dynamic component of the detector’s bias response (i.e., zero-second exposure, see Sect. 3.3.3). Flat field coefficients ($F_{\mathit{ijkl}}$) provide the scaling factor necessary to make pixel (i,j) behave like the average-pixel for zoom position $l$ and filter $k$ (see Sect. 3.6). The conversion between incident photon flux and the electron generation rate in the detector is given by the system’s spectral response ($r_{ \lambda ,k}$ $[\frac{e^{-}}{ph}]$) which, for each filter $k$, we break into a wavelength-independent radiometric coefficient ($r_{o,k}$ $[ \frac{e^{-}}{ph}]$) and a normalized spectral profile that describes the system’s weighted spectral throughput ($\bar{r}_{\lambda ,k}$ [unitless]). Note that both $r_{o,\mathit{ijk}}$ $[\frac{e^{-}}{ph}]$ and $\bar{r}_{\lambda ,k}$ are dependent on which RGB Bayer pattern filter sits above pixel (i,j). The radiance incident upon the front aperture is given by $L_{\lambda }$ $[\frac{\mathrm{W}}{\mathrm{m}^{2} \, \mathrm{sr} \, \mathrm{nm}}]$, and $\frac{\lambda }{hc}$ $[\frac{ph}{J}]$ is a conversion factor between energy and photon flux. For completeness, we include $N_{ij}$ as the zero-mean Poisson noise of the incidence photon flux in $[DN]$. Wavelength dependent parameters are integrated over the spectral range of the detector, generally $300 - 1100~\mbox{nm}$ ($[\lambda _{1},\lambda _{2}]$). Note that the values of $r_{o,\mathit{ijk}}$, $D_{ij}$, and $B_{ij}$ have significant, but well characterized, temperature dependencies (see Sects. 3.7, 3.3.2, and 3.3.3, respectively). Equation (1) can be inverted to derive the mean, bandpass-integrated, spectral radiance incident upon the camera’s front aperture:

2 $$ \begin{aligned} \langle L_{\lambda } \rangle _{\mathit{ijkl}} & \equiv \frac{ \int \bar{r}_{k} L_{\lambda } \frac{\lambda }{hc} d\lambda }{ \int \bar{r}_{k} d\lambda } \\ & = \frac{ g \, F_{\mathit{ijkl}} }{ A\varOmega _{l}\,(t+t_{sm,ij})}\, \frac{(DN_{\mathit{ijkl}} - B_{ij} - \frac{D_{ij}}{g} (t+t_{sm,ij}) )}{ r_{o,\mathit{ijk}} \int _{\lambda _{1}}^{\lambda _{2}} \bar{r}_{k} d\lambda }, \\ \langle \lambda \rangle _{\mathit{ijkl}} & \equiv \frac{ \int \bar{r}_{k} \lambda d\lambda }{ \int \bar{r}_{k} d\lambda }. \end{aligned} $$

Equation (2) forms the basis of the radiometric calibration pipeline discussed in Sect. 4.1. After describing the test facilities and calibration targets $\&$ sources in Sect. 3.2, Sects. 3.3-3.7 will discuss the radiometric tests conducted to derive the parameters outlined in Equation (1). Section 3.3 will discuss CCD characterization, deriving detector gain ($g$), read noise ($RN$), full well, linearity, dark current ($D_{ij}$), bias response ($t_{sm,ij}$ and $B_{ij}$), and present the bad pixel map. Section 3.4 will derive the etendue ($A_{o}\varOmega _{l}$) and f-number ($f_{\#}$) as a function of zoom. Section 3.5 will present the system spectral throughput ($r_{\lambda ,k}=r_{o,k} \: \bar{r}_{\lambda ,k}$). Section 3.6 will discuss the flat field correction maps ($F_{\mathit{ijkl}}$) and Sect. 3.7 will derive the radiometric coefficients ($r_{o,\mathit{ijk}}$), including a description of their observed temperature dependence.

#### Geometric Testing

Complementing the radiometric calibration, which measures the electronic properties of the system, the geometric calibrations measures the spatial and mechanical properties of the Mastcam-Z cameraheads. The Modulation Transfer Function (MTF) is used to characterize the spatial sensitivity and optical quality of the system, while stray light testing verified the rejection of light outside the field of view. The geometric camera model describes the change in camera properties, such as focal length and image center, with motions of the zoom and focus mechanisms. Geometric calibration also measures the orientation of the cameras with respect to each other, such as the toe-in angle and center-to-center stereo baseline. Sections 3.8-3.11 describe the geometric calibrations, including image quality via MTF measurements (Sect. 3.8), stray light testing (Sect. 3.10) and derivation of the geometric camera model parameters (Sect. 3.11).

In designing the geometric calibration tests we followed best-practices and lessons-learned from previous calibration efforts (e.g., Bell et al. 2004, 2017; Caplinger 2013), while also adding to the canonical test programs. In deriving geometric camera parameters, for example, we obtained datasets appropriate for two different and complementary analysis techniques. One is a legacy method developed by JPL that is based on the precise a-priori knowledge of target location via metrology surveys and solves for coefficients of the CAHVOR camera model (Di and Li 2004; Gennery 2001, 2006; Bell et al. 2017; Maki et al. 2012). This analysis technique has been used on all NASA rover-based cameras flown to-date. The second analysis technique uses an industry-standard photogrammetric approach to solve for target location and camera parameters together in a single bundle adjustment (i.e., no metrology required) and derive coefficients for the OpenCV camera model (Zhang 2000; Bradski 2000; Klopschitz et al. 2017). Section 3.11.1 presents preliminary results from both the metrology-dependent (JPL) and the pure-photogrammetric techniques, comparing initial results between the two methods and models. Follow-on papers will be published with detailed results of the geometric calibration for each technique (J.N. Maki, personal communication, May 2$^{nd}$, 2020; C. Tate, personal communication, May 2$^{nd}$, 2020). For MTF testing, we used an open-source industry standard software package: MTF Mapper (https://sourceforge.net/projects/mtfmapper/). This software was used to both generate target patterns for use in testing (see Sect. 3.2.2) as well as analyze observations of the targets to derive system MTF (Sect. 3.8).

Near the end of stand-alone calibration at MSSS, a geologic test target was observed. Section 3.12 describes observations of this geoboard, whose primary purpose is to verify calibration accuracy. Past experience, including peer review of the MER Pancam Calibration Plan and the results from that effort (Bell et al. 2004, 2006), as well as from calibration of the MSL Mastcam (Bell et al. 2017), demonstrate that it is important to validate instrument performance and the pre-flight calibration pipeline by obtaining independent observations of reflectance standards and well-characterized geologic samples. Observations of these materials can be compared to laboratory measurements to assess the true level of expected uncertainties (see Sect. 3.12).

### Sources and Facilities

#### Test Facilities

Stand-alone calibration was primarily performed at ambient temperature and pressure in a class 10,000 clean room at MSSS in San Diego, CA, between April 26$^{th}$ and May 9$^{th}$, 2019. Limited data were also acquired at temperatures ranging from $-50^{\circ }$ C to $+50^{\circ }$ C inside a thermal-vacuum chamber at MSSS during Verification and Validation (V&V) testing. While more extensive vacuum chamber testing was planned to be conducted at Arizona State University, schedule pressure and various hardware development delays necessitated reducing the scope of the planned calibration to exclusively use the MSSS facilities. Regardless, an extensive dataset was collected (see Table 3). Temperature-dependent effects were characterized from a combination of V&V data and analog testing using a Commercial-Off-The-Shelve Mastcam-Z simulator known as the Mastcam-Z Analog Spectral Imager (MASI, see Sect. 3.2.2).

#### Calibration Source and Targets

##### Integrating Sphere

**Fig. 8:**
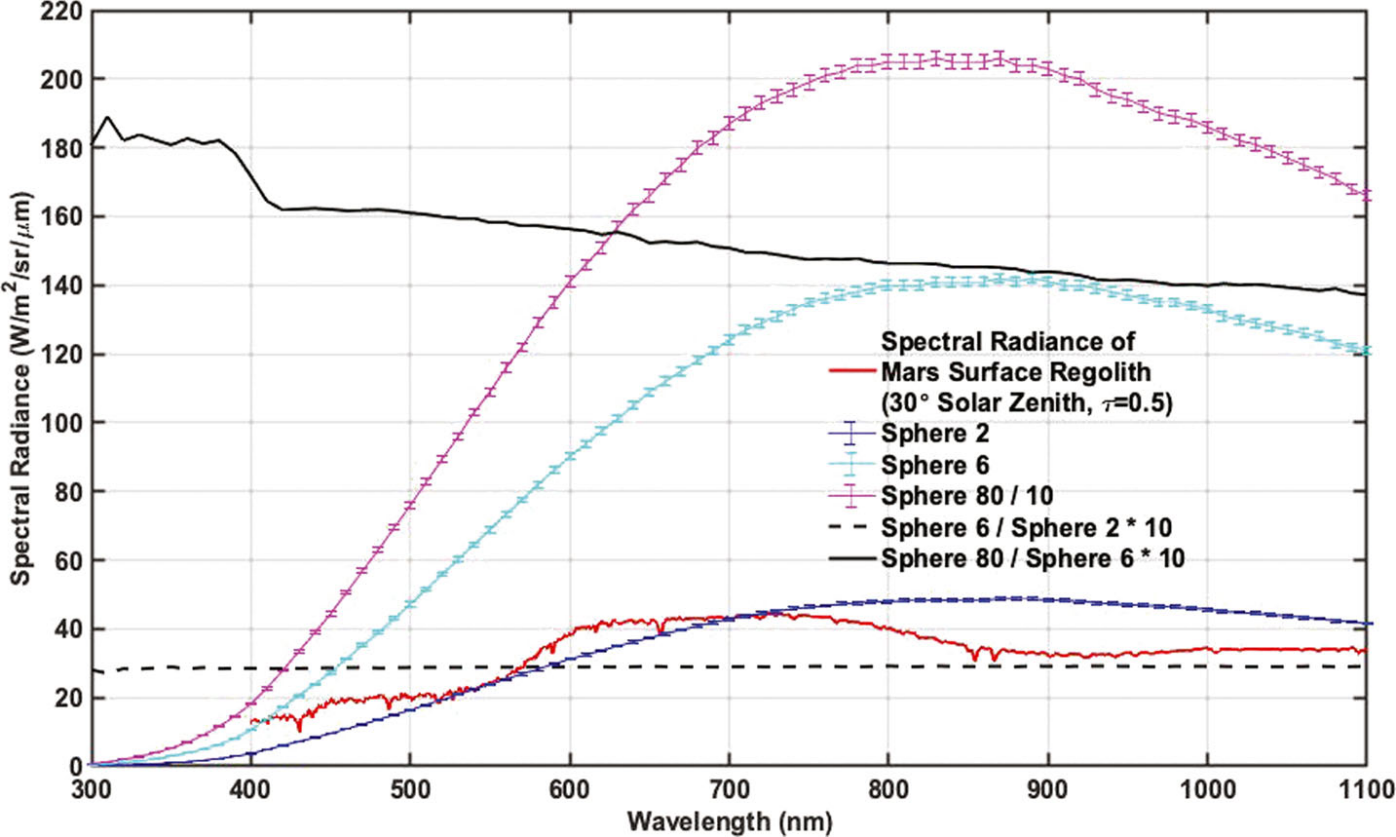
Comparison of integrating sphere spectral radiance at three ISOP levels: full flux (all three lamps at maximum power, ISOP = 80 $\frac{\mathrm{mW}}{\mathrm{cm}^{2}\mathrm{sr}}$ [1–2.5 μm], divided by 10 for plotting purposes) and for two values using the single tunable lamp at $\mathit{ISOP}=6$ $\frac{\mathrm{mW}}{\mathrm{cm}^{2}\mathrm{sr}}$ and $\mathit{ISOP}=2$ $\frac{\mathrm{mW}}{\mathrm{cm}^{2}\mathrm{sr}}$, respectively. Error bars represent the $95\%$ confidence interval, which is typically $\sim 1\%$ of the sphere output at a given wavelength (see sphere calibration reports in the SOM). For comparison, the typical spectral radiance of the Martian regolith is also plotted, assuming an atmospheric $\tau $ of 0.5 and solar zenith angle of $30^{\circ }$. Regolith reflectance is approximated by Mars Global Simulant 1 (Cannon et al. 2019). The dashed black lines shows the ratio between the two single lamp spectra (multiplied by 10 for plotting purposes) and is found to be spectrally flat. The solid black line shows the ratio between full flux and 6 $\frac{\mathrm{mW}}{\mathrm{cm}^{2}\,\mathrm{sr}}$ single lamp output (also multiplied by 10). At full flux, the introduction of two additional lamps slightly alters the spectral shape of the integrating sphere output. Care was taken to only use a single lamp through all radiometric testing (except for calibrating the solar filters) as to not alter the spectral shape of the incident flux during radiometric testing

All radiometric tests outside of the spectral throughput scans made use of a Spectralon-coated broadband integrating sphere designed by Labsphere. This source uses NIST-traceable halogen lamps to provide spatially-uniform broadband flux over a $4''$ aperture with $<1\%$ spatial variation across the field. For most testing, a single, tunable lamp was used allowing for fluxes ranging from ∼0.1–10 $\frac{\mathrm{mW}}{\mathrm{cm}^{2}\mathrm{sr}}$ integrated from 1–2.5 μm. In the case of solar filter testing, all three lamps were used to provide a maximum flux of 80 $\frac{\mathrm{mW}}{\mathrm{cm}^{2}\mathrm{sr}}$ (also integrated from 1–2.5 μm). The integrated radiometer used to measure integrating sphere output (ISOP) is calibrated for and sensitive to the wavelength range 1–2.5 μm, so ISOP levels are set and reported as the integrated flux over this range. In order to determine the spectral radiance of the sphere in $\frac{\mathrm{mW}}{\mathrm{cm}^{2}\mathrm{sr}\upmu \text{m}}$, the ratio between the displayed and calibration curve ISOP is used to scale the calibration spectra from Labsphere. As shown in Fig. 8, the spectral shape of sphere output is constant over the flux range of the tunable lamp. When multiple lamps are used, however, the spectral shape can change. For this reason, radiometric calibrations were performed using only the tunable lamp. The one exception is solar filter (R7/L7) flat field generation (see Sect. 3.7), where all three lamps were turned to full power. Calibration curves were generated for multiple ISOP values, including full power (ISOP = 80 $\frac{\mathrm{mW}}{\mathrm{cm}^{2}\mathrm{sr}}$). For reference, an ISOP of $\sim 2$ $\frac{\mathrm{mW}}{\mathrm{cm}^{2}\mathrm{sr}}$ is similar to the expected radiance from the Martian regolith, assuming an atmospheric opacity $\tau _{\mathrm{atm}}=0.5$ and $30^{\circ }$ solar zenith angle (see Fig. 8). The integrating sphere was radiometrically calibrated by Labsphere both before and after stand-alone testing. The associated calibration reports are provided in the SOM. To within error, the spectral shape and radiance of the lamps were found to be constant between the two calibrations. When using the single tunable lamp, the output remains spectrally uniform for varying flux levels (Fig. 8).

##### Monochromator

The monochromator setup used for Mastcam-Z stand-alone calibration included an Oriel CS260 F/3.9 monochromator, 250W QTH light source, and a Newport 818-UV/DB radiometer capable of collecting optical power across the 200 nm–1100 nm band. The monochromator went through wavelength accuracy calibration in-house at ASU both before and after stand-alone calibration. This calibration collected a radiance spectrum of a HgAr rare gas lamp and compared the scan’s peak spectral lines with those available from NIST databases to confirm wavelength uncertainty to be $\le 1$ nm. During this calibration, the monochromator was commanded to scan at a 1 nm step-size and its input and exit slits were set to produce a resolution of 1 nm. The radiometer was sent to the manufacturer (Newport) for calibration both before and after Flight Model stand-alone calibration. Results showed radiometer sensitivity to be consistent between the two calibrations. During stand-alone testing the monochromator performed calibration scans over a wavelength range of 300 nm–1100 nm at a step-size of 2 nm to characterize and correct for any wavelength dependence in monochromator output (see Sect. 3.5).

##### Geometric Targets

**Fig. 9:**
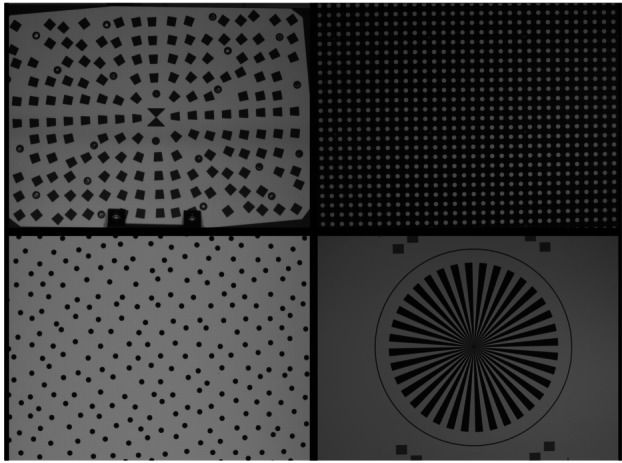
Geometric targets used during stand-alone calibration taken with the right Mastcam-Z. Top Left: MTF Mapper “lensgrid” target used to calculate the MTF at a distance of 1.7 m. Top Right: JPL uniform grid dot target at a distance of 3.2 m. Bottom Left: JR random dot target at a distance of 1.5 m. Both dot targets were used for determining instrinsic camera parameters. Bottom Right: Siemens star target used as an additional target for calculating the MTF at a distance of 2.5 m

Five different geometric targets were used during testing. The first consists of a $40\times 40$ dot grid, where each dot has its own identifying number, that was used for geometric calibration (see Sect. 3.11). This target uses the known distance between and regular pattern of the dots to characterize the geometric distortion of the Mastcam-Z optics. The second target was a proprietary random dot target that is used for the geometric calibration using a pure-photogrammetric technique (see Sect. 3.11). This target uses the known distribution pattern of the dots in combination with proprietary calibration software provided by Joanneum Research (JR; see Sect. 3.11). The third target was a publicly available MTF Mapper “lensgrid” target for use with the MTF Mapper software (https://sourceforge.net/projects/mtfmapper/). This target was printed in two sizes – one small for large focal lengths, and one large for short focal lengths. During testing, however, it was found that the small target was “soft”, or blurry, and therefore limited the maximum MTF that could be measured. As a result, only the large target was used for testing. Fourth, a Siemens star target was also printed as an additional target to use for MTF measurements. While images of this target were acquired, they are not used in the analysis for this manuscript. Finally, an Imatest SVG, consisting of a grid of slant edges similar to the “lensgrid” target was used during V&V testing. All targets used during stand-alone testing were printed on gator board by the Jet Propulsion Laboratory metrology group. Prior to delivery to the test sites (MSSS for stand-alone testing and JPL / Kennedy for ATLO), each target was ID-ed and measured via laser metrology. For the dot targets, dot positions were re-checked and verified during pre-test metrology by the survey team. Under perfect conditions, positional accuracy of the dot patterns and slant edge quality is on the order of 25 microns, but this varies depending on the lab environment (wind currents from the air conditioning, flexing of the targets in the frames, etc.). As a result, targets were certified to sub-mm positional accuracy. Fig. 9 shows a collection of images of the targets acquired by the flight instrument during stand-alone calibration.

##### Light Sources

For non-radiometric tests (e.g. geometric tests, geoboard imaging), additional, uncalibrated light sources were used to improve signal, especially in the near-IR where the fluorescent ceiling lights produced little flux. These sources include halogen shop lamps and commercially available solar-simulator bulbs (i.e. bulbs with a similar color temperature to ambient sunlight). When possible, these lamps were placed symmetrically about the target to allow for as uniform illumination as possible. Little effort was made, however, to measure the absolute position of the lamps with respect to the target(s).

##### Stray-Light Apparatus

**Fig. 10:**
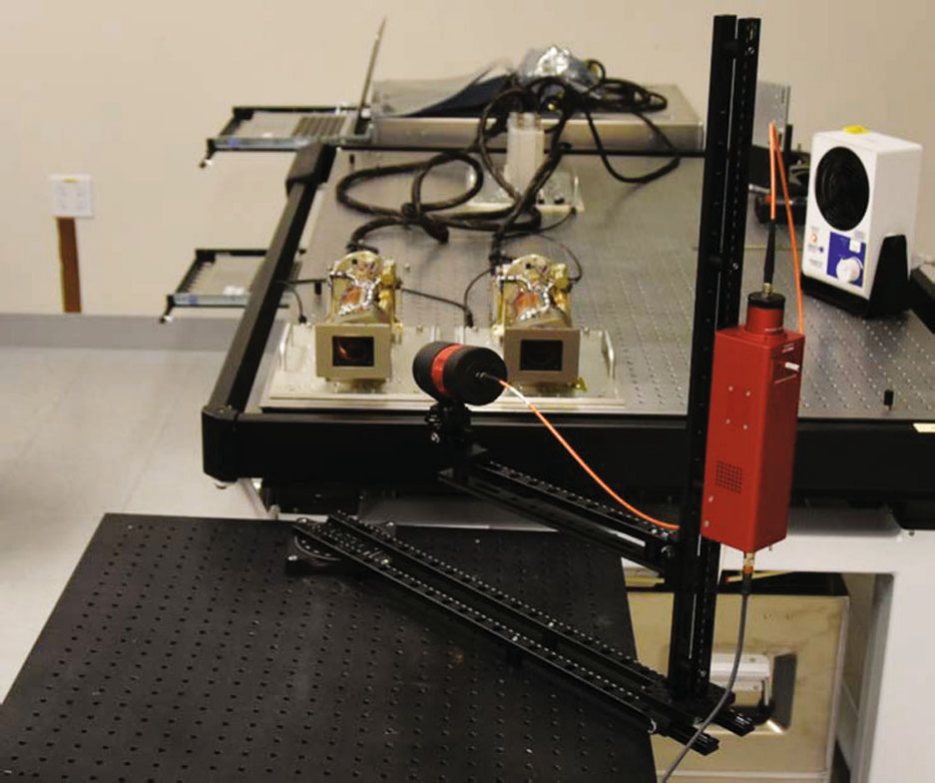
Image of the stray light assembly used during stand-alone calibration. A broadband source is coupled to a $2''$ diameter collimator by an optical fiber. Dovetail rails are mounted on rotation stages that allow for 3-dimensional positioning of the collimator with respect to the camera boresight. Shown here is the collimator at a position of $-20^{\circ }$ in azimuth and $-0^{ \circ }$ elevation

For stray light testing, a collimated source was needed to mimic the far-field imaging of solar rays to understand the out-of-field rejection quality of the Mastcam-Z baffles and optical housing. For this, commercial off-the-shelf (COTS) components, including a broadband light source and collimating lenses, were used to create a $2''$-diameter collimated beam to fill the $1''$ first aperture of the Mastcam-Z cameras (Fig. 10). The source was chosen to maximize collimated flux onto the detector, which was ∼300x dimmer than the flux of the Martian mid-day Sun ($\sim 60$ $\frac{\mathrm{mW}}{\mathrm{cm}^{2}}$ after convolution with the Mastcam-Z bandpass). These components were then combined with rotation and tip-tilt stages in order to position the collimated beam at known azimuth and elevation angles with respect to the cameraheads. From measurements with the MASI camera (see below), we find repeatability of the assembly placement to be ∼10 pixels in azimuth for a fixed elevation.

##### Mastcam-Z Analog Spectral Imager

**Fig. 11:**
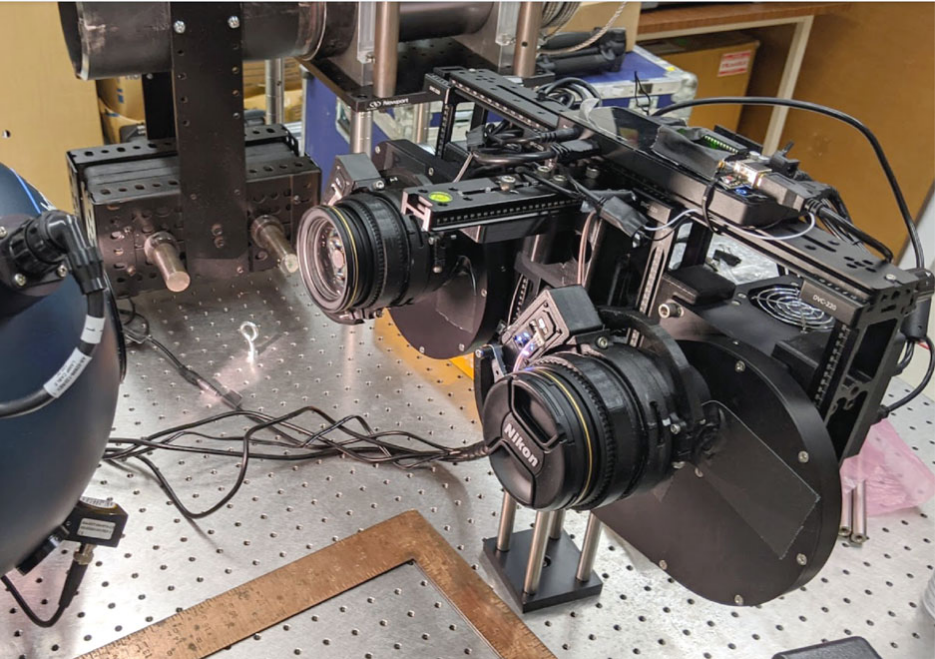
Mastcam-Z Analog Spectral Imager (MASI), as seen in the lab, preparing to collect flat fields. Primary components such as the left and right filter wheels and zoom lenses are visible. For scale, the cameras are mounted with a stereo baseline of 23 cm and the holes on the optical bench are spaced $1''$ apart

**Fig. 12:**
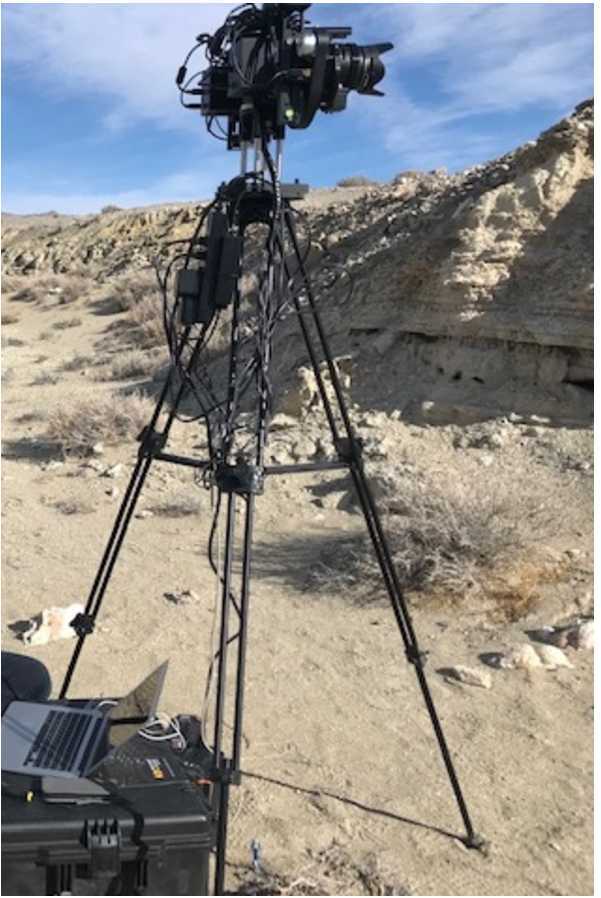
MASI collecting a set of multispectral images in the field at a rover operations training site in Hawthorne, NV, February 2020. For scale, the cameras boresights are mounted 1.7 m from the ground, similar to the flight system

The Mastcam-Z Analog Spectral Imager (MASI) is a Mastcam-Z emulator built primarily using COTS components (Figs. 11 and 12). The system uses the same KAI-2020 CCD detector as Mastcam-Z, and consists of two cameras with the same toe-in angle and stereo separation as the flights system. The cameraheads are supplied by Finger Lakes Instrumentation (FLI), and include eight-position filter wheels. The filters wheels are populated with a complete set of Mastcam-Z flight spare filters, including 2 IR-cutoff filters, 12 narrow-band geology filters, and 2 neutral density filters for solar imaging. The system uses two Nikon zoom lenses with effective focal lengths ranging from 30–110 mm. The cameras are mounted to a base fitted with azimuth and elevation actuators, and the entire system sits on a tripod. Mechanical motions (azimuth, elevation, focus, zoom, and filter position) are controlled using an Arduino (Badamasi 2014) through a USB interface. The detector is packaged within a Joule-Thomson cooler that permits temperature control between $-50^{\circ }$ C and $50^{\circ }$ C, allowing the assessment of temperature-dependent detector properties such as spectral quantum efficiency (see Sect. 3.7.1). MASI was constructed for three main purposes: 1) to perform preparatory testing for the formation of calibration procedures, 2) to characterize any anomalies which arose during the calibration of the Mastcam-Z flight units, and 3) to be utilized in the field as an instrument analog, with the goal of discerning Mastcam-Z’s capabilities and assessing the utility of multi-spectral sequences designed to identify specific minerals or alteration signals. Calibration protocols for geometric and radiometric calibration, dark current, and spectral throughput measurements were performed in advance of the calibration of Mastcam-Z in order to calibrate MASI and determine appropriate procedures for flight unit calibration.. Following support of calibration activities, MASI has been utilized as a Mastcam-Z instrument emulator at Mars analog sites, including participation in the February 2020 Rover Operations Activities for Science Team Training (ROASTT) field exercise. Multispectral datasets have and will continue to be collected and cross-correlated with data from other field and lab instruments to determine Mastcam-Z’s mineralogical identification capabilities, and to ascertain the best filter ratios and spectral parameters for tactical use by the Mastcam-Z team.

#### Ground Support Equipment and Software

Ground Support Equipment (GSE) is used to support instrument checkout, calibration, and testing through the time of integration with the rover. GSE allows flight-like operation of the instrument in both ambient and environmental test conditions. The GSE uses COTS hardware to save development effort, but it is designed to ensure the safety of the flight hardware. The GSE software used for Mastcam-Z’s calibration and testing is based on heritage software used for the MSL Mastcam, MARDI, and MAHLI calibration, although significant upgrades were implemented for Mastcam-Z.

Mastcam-Z calibration involved almost 600 scripts containing over 90,000 individual GSE commands. Prior instrument calibration efforts (e.g., MSL) had been conducted by a small team of engineers entering GSE commands manually, or in the form of handwritten scripts through a primitive GUI. The volume and complexity of the Mastcam-Z science calibration made manual commanding infeasible. To support Mastcam-Z calibration, the MSSS Operations Team devised a software solution to grant non-engineers from university collaborators the power to specify and update test parameters in a shared spreadsheet, and then “expand” that spreadsheet into the ∼600 necessary scripts in near real-time. This efficient process was critical to success, as it allowed real-time changes to calibration procedures based on unforeseen scheduling updates related to personnel, equipment maintenance, anomalous imaging results, or other issues. It also ensured that MSSS would maintain complete control over all commands issued to sensitive flight hardware, thus protecting instrument safety by not allowing direct external authorship of scripts.

Once generated, the GSE command scripts were executed by means of a custom GUI built and maintained by the Operations Team. This was based on an existing MSSS tool for GSE control, but with several key improvements:

- Ability to visually monitor the progress of GSE script execution
- Ability to abort GSE scripts during execution without risk to hardware
- Ability to write out log files documenting every GSE command sent to the cameras
- Ability to constantly monitor the state of the cameras and associated mechanisms
- Ability to intuitively send imaging commands without heavy reliance on documentation
- Ability to conduct detailed image quality analysis in real-time, including histogram plotting, subframe viewing, focus motor-count to distance calculations, debayering, color-stretching, and parsing of metadata
- Built-in error warnings to prevent common commanding mistakes that could result in mechanism faults

Most notably, the ability to abort GSE scripts mid-execution allowed our commanding sequences to become longer and more sophisticated without worrying about lost operating time or unnecessary mechanism usage. For instance, if a calibration target were accidentally bumped in the middle of a test, that test could easily be aborted and then resumed where it left off once the target was readjusted. Furthermore, long and intricate coordination with calibration instruments such a scanning monochromator could be orchestrated without significant risk.

The custom software tools described above were developed by the MSSS Operations Team over several months. This was an iterative process, involving many rounds of revisions and incorporating feedback and suggestions from both science and engineering teams. Because the MSSS Operations Team was both the developer and end-user of this software, troubleshooting problems was easy, and did not cause extreme delays. These capabilities will be available for future instrument calibration efforts.

### CCD Characterization

#### Photon Transfer

The Mastcam-Z CCD’s gain $(g~[\frac{e^{-}}{DN}])$, read noise $(RN~[e^{-}])$, and full well ($FW$ $[e^{-}]$) were derived using the photon transfer technique (Janesick et al. 1987). Images were collected on May 2$^{nd}$, 2019, in an ambient temperature cleanroom for both the left and right cameras using the IR-cutoff filters (L0/R0) at a focal length of 110 mm (zoom motor count $\mathit{zoom}_{mc}=9600$) and ISOP settings of 1, 2, 3, 4, 5, 6, 8, and 10 $\frac{\mathrm{mW}}{\mathrm{cm}^{2}\,\mathrm{sr}}$ (integrated flux from 1-2.5 μm), with eight variable exposure times at each flux level ranging from 0.5 ms–80 ms. Integration times were chosen to sample the full range of well depths for each of the RGB Bayer pattern filters. For each flux level, bias frames were acquired before and after the exposure time ramps. All exposure times and bias observations were collected in sets of 10 frames. For this and other radiometric tests (unless otherwise noted), all frames were acquired at the uncompressed 11-bit output of the cameras (see Bell et al. 2017, for details on image acquisition modes). For additional details on the data acquisition and procedures for the photon transfer analysis, as well as for other tests, please see the Calibration Plan and Procedures provided in the SOM.

**Fig. 13:**
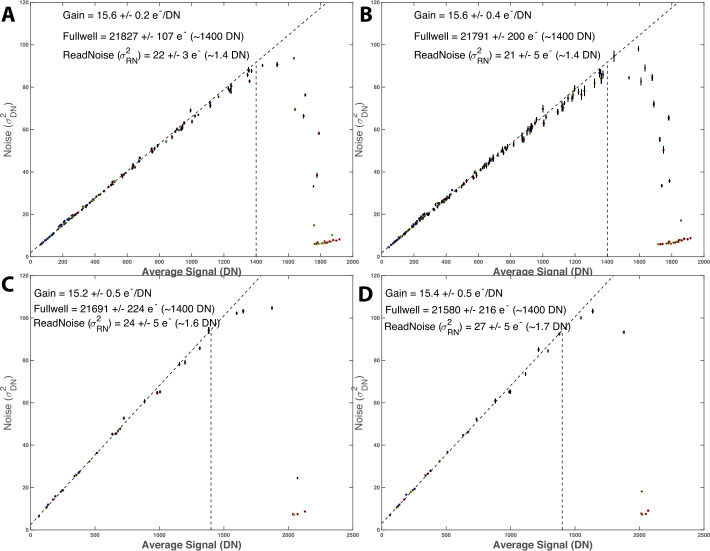
Photon transfer curves for: (**A**) left Mastcam-Z at ambient, (**B**) right Mastcam-Z at ambient, (**C**) left Mastcam-Z at $-5^{\circ }$ C, (**D**) right Mastcam-Z at $-5^{\circ }~\mathrm{C}$. The slope of a linear fit to variance ($\sigma ^{2}$) vs. average DN is 1/gain while the read noise is the offset. Full-well occurs at the signal level where variance deviates from a linear relationship with average DN (Janesick et al. 1987). All photon transfer curves were measured at 110 mm ($\mathit{zoom}_{mc}=9600$). Gain and full well are found to remain constant with temperature, to within error. Read noise is consistent as well, but may increase slightly at colder temperatures

A subset of photon transfer observations were also acquired at a detector temperature of $-5^{\circ }$ C on April 27$^{th}$, 2019, while the cameras were in the TVAC chamber during V&V testing. For this dataset, only two integrating sphere flux levels at 5 and 10 $[\frac{\mathrm{mW}}{\mathrm{cm}^{2}\,\mathrm{sr}}]$ were observed. The analysis scripts used to produce the photon transfer curves (Fig. 13) and derive detector characteristics are provided in the SOM.

**Fig. 14:**
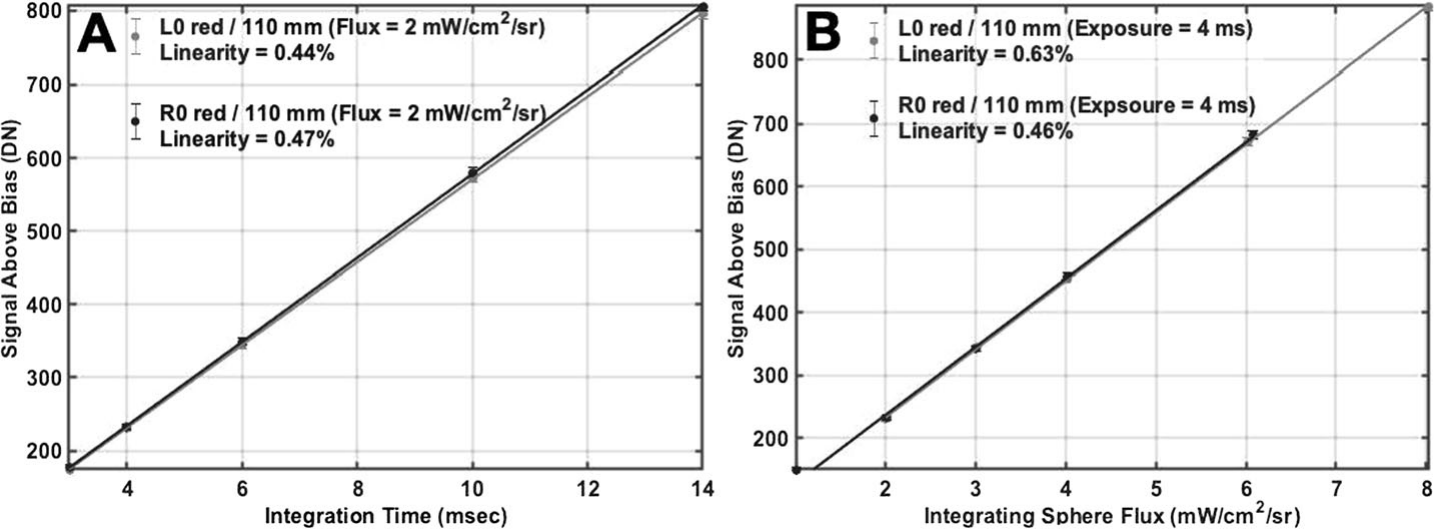
Example of Mastcam-Z linearity in response to changing integration time (**A**) and flux (**B**). In both cases, the cameras nonlinearity is $<0.6\%$. Nonlinearity from increasing flux (B) appears slightly larger than nonlinearity from increasing exposure time (A). Reported DN represent the average signal above bias in a $100\times 100$ pixel box at the center of the CCD from the photon transfer dataset using the L0/R0 filters and the 110 mm zoom setting ($\mathit{zoom}_{mc}=9600$)

The photon transfer curves for the left and right cameras at ambient and $-5^{\circ }$ C are shown in Fig. 13. In order to reduce dependence on fixed-pattern-noise, the read noise and gain where calculated using the spatial variance of the difference between two frames acquired of the same source (as opposed to using the spatial variance of single images). The gain ($g$) of the left and right cameras agreed to within error, with values of $15.6 \pm 0.2$ $[\frac{e^{-}}{DN}]$ and $15.6 \pm 0.4$ $[\frac{e^{-}}{DN}]$, respectively. To within error, the gain was not found to be temperature dependent (see Fig. 13). Read noise may increase slightly at colder temperatures, but was also found to be consistent between ambient and $- 5^{\circ }$ C (within 1-sigma), with a mean value of $22 \pm 3~e^{-}$ (left) and $21 \pm 5~e^{-}$ (right). Detector full well ($FW$) occurred at $21{,}827\pm 107$ $e^{-}$ (left) and $21{,}791\pm 200$ $e^{-}$ (right), consistent with ∼1400 DN above the bias offset. Full well was measured as the point where the variance ($\sigma ^{2}$) deviated from a line with the average signal ($DN$) by more than $5\%$. No color dependence was found for any of the values derived from the photon transfer analysis. These results are consistent with the CCD properties for MSL Mastcam, which uses the same KAI-2020 detector, as reported by Bell et al. (2017). In fact, both CCDs were procured as part of the same lot (M. Caplinger, personal communication, May 7$^{th}$, 2020). Between $10\%$ and $90\%$ full well, both cameras have less than $0.6\%$ deviation from linearity, regardless of whether the signal was derived from increasing exposure time or increasing scene flux (Fig. 14). In this case, deviation from linearity is defined as the sum of the maximum deviations above and below the best-fit line, measured relative to the maximum signal level (https://www.photometrics.com/learn/imaging-topics/linearity).

#### Dark Current

**Fig. 15:**
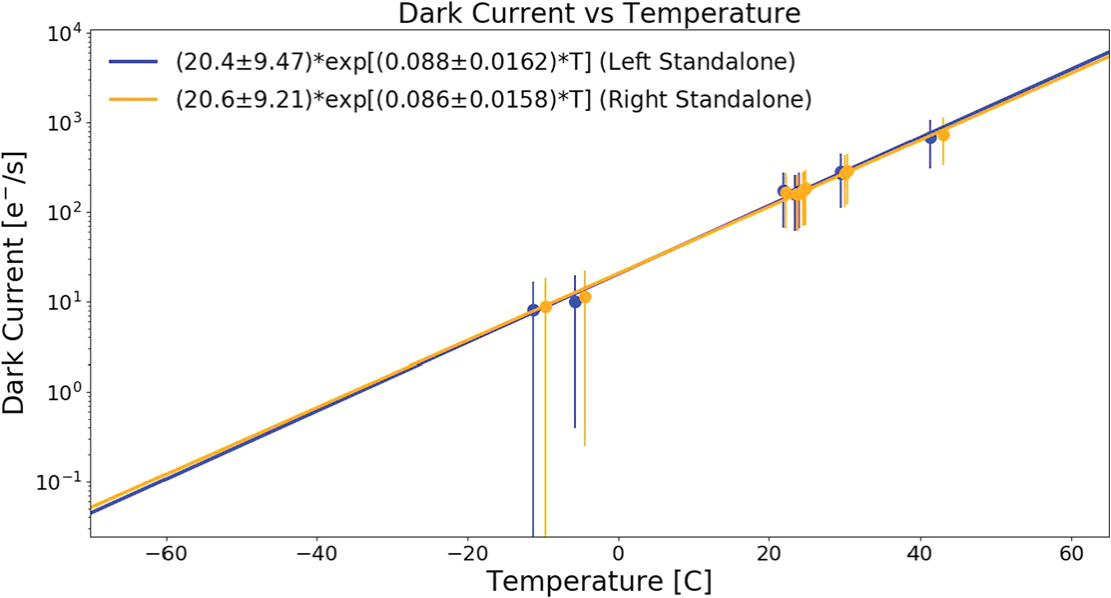
Plot of the measured dark current for the left (blue) and right (orange) cameras. Measured data are plotted as points, while the best fit exponential is plotted as a solid line. The measured dark current is ${<} 1\, DN/s$ for temperature below $-15^{\circ }$ C and $-1^{\circ }$ C, respectively, for the left and right Mastcam-Zs

Dark current is the result of thermally generated electron-hole pairs that are indistinguishable from photo-generated electrons in the detector. Dark current is known to have an exponential temperature dependence and is generated irrespective of scene illumination. For Mastcam-Z, dark current was measured through a series of dedicated observations acquired at several temperatures during V&V temperature ramps at MSSS. Interrogated temperatures included $-10^{\circ }~\mathrm{C}$, $-5^{\circ }~\mathrm{C}$, $20^{\circ }~\mathrm{C}$, $25^{\circ }~\mathrm{C}$, $30^{ \circ }~\mathrm{C}$ and $40^{\circ }~\mathrm{C}$. In total, 8 dark current datasets were acquired on the left Mastcam-Z, while 11 were acquired on the right, including 3 additional measurements at ambient temperatures. For each dataset, lights were turned off in the cleanroom or TVAC chamber area, the solar filter (L7/R7) was rotated into place, and a series of observations at 0, 10, 1000, 10 000, 20 000, and 100 000 ms were acquired. After subtracting zero-second exposures to remove bias (see Sect. 3.3.3), lines were fit to the average response of the central $100\times 100$ pixels in each dataset in order to determine the dark current rate $[\frac{e^{-}}{s}]$ from the slope of the best fit line at each temperature. An exponential form was then fit to these dark current measurements in order to determine the temperature dependence. Fig. 15 plots the measured dark current as a function of temperature for both cameras along with the best fit exponential model. At room temperature ($20^{\circ }$ C), dark current rates were 172.1 $e^{-}/s$ ± 105.2 $e^{-}/s$ (10.9 DN/s ± 6.7 DN/s) and 167.8 $e^{-}/s$ ± 102.5 $e^{-}/s$ (10.6 DN/s ± 6.5 DN/s) for the left and right cameras, respectively. The models predict dark current rates to drop to $<1~\mbox{DN}/\mbox{s}$ ($<16\, e^{-}/s$) for temperature below $-15^{\circ }$ C. As detector operating temperatures are typically $-10^{\circ }~\mbox{C}$ or below (Bell et al. 2017) and integration times are measured in milliseconds, these results suggest that the dark current under Martian conditions will be negligible in the majority of Mastcam-Z observations.

#### Bias and Smear

**Fig. 16:**
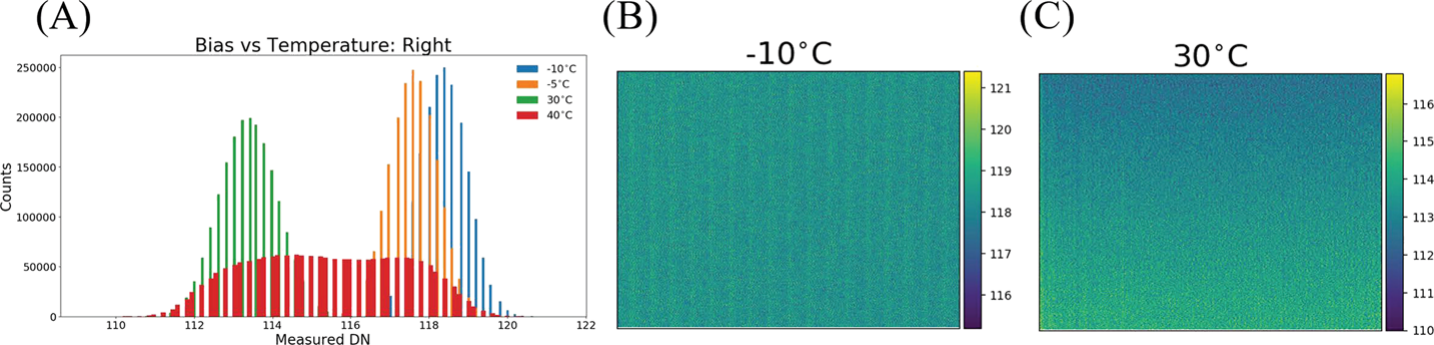
Static bias as a function of temperature for the right Mastcam-Z. Bias data were extracted from the zero second exposure frames of the dark current data sets (L7/R7 filters in place). **A**) Histogram of the distribution of bias values as a function of temperature. Colder temperatures have a narrow, but slightly higher average bias. Warmer temperatures, have a broader, slightly lower distribution. **B**) Spatial dependence of the static bias at a typical Mars temperature of $-10^{\circ }$ C. The bias is observed to be uniform across the detector. **C**) Same as **B**) but at $30^{\circ }$ C. Here we observe a small gradient in the bias frame. Values for all panels are similar for the left Mastcam-Z

In addition to photon-generated scene flux and thermally-generated dark current, each Mastcam-Z image also contains a bias (offset) that is independent of the commanded exposure time. This bias can be isolated by commanding a zero-second exposure and, during calibration, bias frames were routinely acquired and subtracted from images of the integrating sphere, monochromator, or geometric calibration targets prior to processing. On Mars, bias frames will not always be acquired, nor were they universally acquired during calibration. Therefore it becomes necessary to model the bias response in order to remove it within the radiometric calibration pipeline. The majority of the bias signal is a static DC offset that slowly varies with temperature. At ambient, the static bias varies along column groups from 114-117 DN in both cameraheads (see Fig. 16). Using the dark current dataset (see Sect. 3.3.2), which includes a series of zero-second exposures acquired at each temperature point, the static bias is shown to vary by $\sim 5$ DN across the expected Mastcam-Z operating temperatures (see Fig. 16). The spatial pattern of the static bias was also observed to change with temperature, becoming more uniform at colder temperatures (see Fig. 16). Similar to MSL Mastcam, the majority of the static bias for Mastcam-Z is removed in the camera electronics prior to 11-to-8-bit decompanding or image compression (Bell et al. 2017). The removed value is stored in the DARK $\_$ LEVEL $\_$ CORRECTION processing parameter keyword in the PDS image label. Note that zero-second exposures in the presence of illumination are sometimes referred to as shutter frames, while bias frames can be used to describe zero-second exposures without light on the camera. Herein, we use bias frames to refer to either illuminated or dark zero-second exposures.

**Fig. 17:**
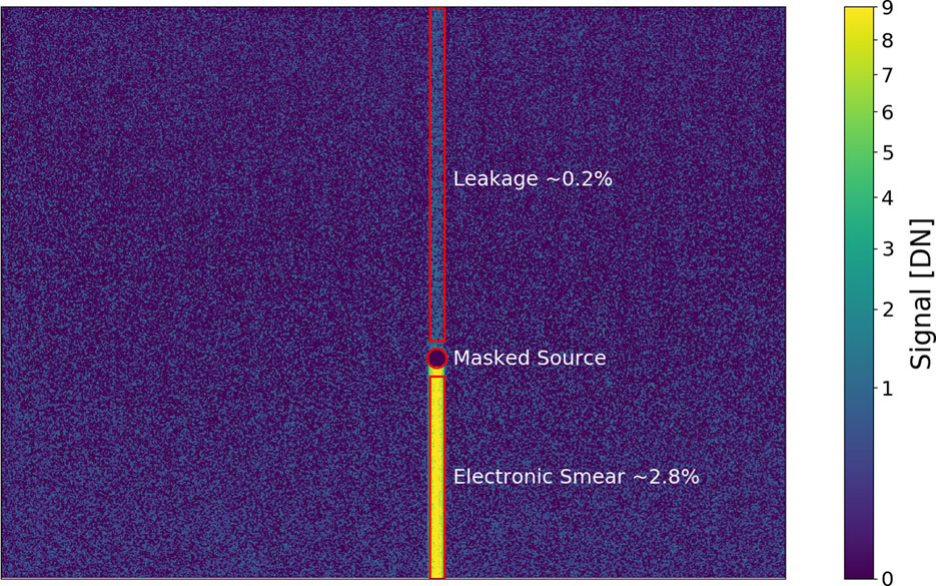
Measurement of the observed electronic smear for a bias frame taken during stray light testing. The bright source signal is masked for clarity. Two forms of signal are observed. First is a weak ∼0.2% signal from long-wavelength photon leakage through the vertical transfer lines. The second, which generates the masked bright source signal, results from the non-zero readout time of the array (see text for details)

In addition to a static (i.e., scene independent) DC offset, there are two additional components in Mastcam-Z shutter frames (illuminated bias frames) that depend on the observed scene; smear and a ghost image. Smear, which is the smaller of the two effects, results from the fact that the KAI-2020 CCD is a progressive scan, interline transfer device (Truesense Imaging 2004). In the CCD, charge is transferred from each photoactive pixel to an adjacent, vertically aligned, light-shielded shift register. Once inside the light-shielded shift register, whose light shielding makes them $\sim 75$ dB less sensitive than the active pixels, the charges are clocked up the CCD one line at a time toward a horizontal shift register at the edge of the detector (see Fig. 3). Once in the horizontal shift register, the charge is clocked out of the device horizontally for digitization. While the shift of charge from the photosensitive pixels into the light-shielded shift registers is fast ($\sim 100$ μs) and simultaneous for all pixels, clocking the collected charge through the vertical and horizontal shift registers takes time. The clock rate for the Mastcam-Z CCDs, like MSL Mastcam, is 20 MHz (Bell et al. 2017). At this rate, the readout time for a full $1640\times1214$ pixel image is 420 ms. Over this time period, some photons can penetrate the light shielded shift register as Mastcam-Z does not have a mechanical shutter. The resulting smear is known as electronic shutter smear. Assuming the light-shielded shift registers are $\sim 75$ dB less sensitive, the 420 ms readout time is equivalent to 0.07 ms integration for the pixels furthest from the readout. For a typical 6 ms integration on Mars, this represents an added flux of only $\sim 1\%$ for the last pixel to be read out (with less of an effect for pixels read out earlier). The excess charge accumulated by this process is “smeared” down the array as it is being clocked out. Smear can also result when longer-wavelength photons, which penetrate deeper into the silicon substrate, generate charge that leaks out into the shift register and is added to the pixel charges during transfer up the channel. This effect generates a vertical streak that extends both above and below the bright source and is similar to blooming, although it can occur in unsaturated sources due to the increased penetration depth of the longer-wavelength photons. Blooming occurs when charge from a saturated pixels leaks out to its neighbor. The linear ramp observed in Fig. 17 shows an example of both electronic shutter smear and vertical leakage (likely from long-wavelength photons) in one of the bias frames acquired during stray light testing (see Sect. 3.10). For this scene, the maximum brightness of the smeared signal was $\sim 2.8\%$ of the unsaturated signal, suggesting an effective sensitivity reduction of ∼64 dB as compared to the active photosites. We note, however, that even though the stray light collimator was not saturated in the bias frame, it is a bright source with a color temperature that is biased toward the near-infrared (the source peaks at ∼1 μm). As a result, the smear in Fig. 17 likely consists of both the long wavelength leakage and traditional electronic shutter smear effects discussed above. In a static scene, the standard electronic shutter smear can be corrected by computing a running sum of the signal levels across each column, subtracting the appropriate fraction from each pixel as you progress (Bell et al. 2017). Blooming and leakage from longer wavelength sources, however, only occur for brighter sources (saturated in the case of blooming) and cannot be readily modeled or removed. The magnitude of the vertical leakage, likely from long-wavelength photon penetration from the collimated source, in Fig. 17 has a magnitude of ∼0.2% of the unsaturated signal.

**Fig. 18:**
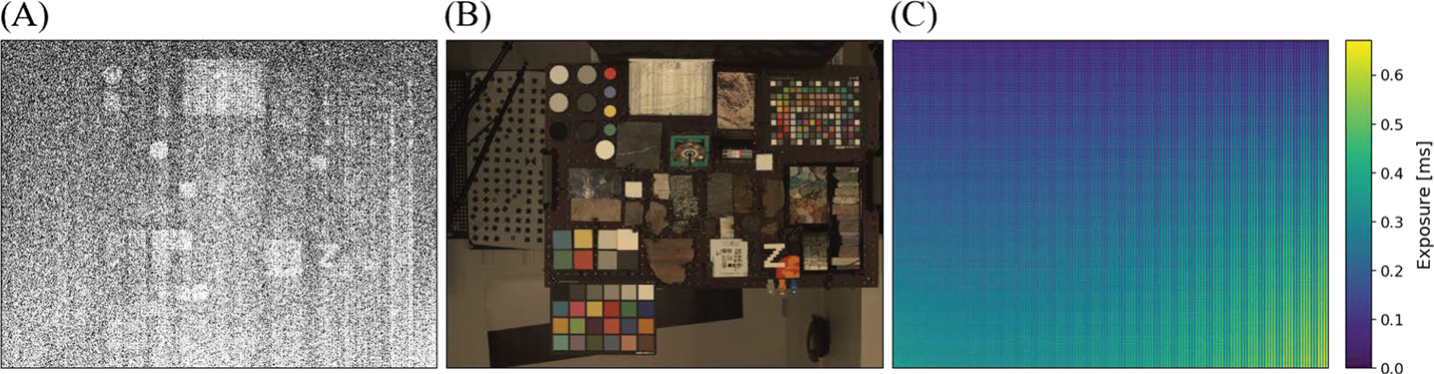
Example of the dynamic bias measured in the R0/L0 filters. (**A**) Example bias frame of the geoboard demonstrating structure in the frame corresponding to brighter targets in the image. Because of the non-zero dynamic bias, a “ghost” image is recorded in addition to the commanded exposure. (**B**) RGB image (L0 filter) of the scene for which the bias frame was acquired. Exposure time was 54 ms. (**C**) Spatial dependence of the dynamic bias for the L0 filter. Maximum additional integration times of ∼0.7 ms are measured, which can correspond to as much as 10% relative signal to a typical L0 exposure on Mars (Table 4). Comparable times are found for the right Mastcam-Z

The larger of the two dynamic bias components is a ghost image that retains the structure of the scene without substantial smearing. A similar effect was seen in Mastcam and was attributed to scene exposure during the $\sim 100$ μs wide transfer pulse created by the DEA to initiate charge transfer from the active pixels to light-shielded vertical shift registers (see Bell et al. 2017, Fig. 16). On Mastcam-Z, a substantially larger dataset was acquired that permitted a more complete analysis of this phenomenon. Mastcam-Z bias frames include ghost images that have equivalent integration times ($t_{sm,\mathit{ijk}}$, see Equation (1)) of up to 0.6 ms for the L0/R0 filters (see Fig. 18). Furthermore, the dynamic bias was observed to be both spatially varying (it worsens farther from the readout location) and wavelength dependent (see Table 4). For L0/R0, the static and dynamic bias were determined by fitting lines to the average response from the ten zero-second exposure images acquired at each sphere ISOP during photon transfer testing (see Sect. 3.3.1). The magnitude of the dynamic bias was observed to be linear with incidence flux on the detectors. For each pixel, the offset of the linear fit against incident photon flux is the static bias level while the slope is proportional to the dynamic bias, and can be converted into an effective integration time by solving for $t_{sm,\mathit{ijk}}$ using Equation (1). For all other filters, the radiometric dataset (see Sect. 3.6) was used to estimate $t_{sm,\mathit{ijk}}$ from the ratio of frame-averaged integrating sphere images at two common flux levels ($\mathit{ISOP}_{1},\mathit{ISOP}_{2}$) and integration times ($t_{1},t_{2}$):

3 $$ \begin{aligned} t_{sm,\mathit{ijk}} &= \frac{\varDelta t_{2} - t_{1}}{1-\varDelta } \\ \varDelta &= \frac{DN_{\mathit{ijkl}}(t_{1},\mathit{ISOP}_{1})-DN_{\mathit{ijkl}}(t_{1},\mathit{ISOP}_{2})}{DN_{\mathit{ijkl}}(t_{2},\mathit{ISOP}_{1})-DN_{\mathit{ijkl}}(t_{2},\mathit{ISOP}_{2})} \end{aligned} $$

where $DN_{\mathit{ijkl}}(t,\mathit{ISOP})$ refers to an average frame commanded at integration time $t$ and observing the integrating sphere at flux $\mathit{ISOP}$. Equation (3) was derived by solving Equation (1) for $t_{sm,\mathit{ijk}}$. For R0/L0, the results of using Equation (3) are consistent with the more detailed (and accurate) approach of fitting the photon transfer dataset described above.

**Table 4:** Table of maximum $t_{sm,\mathit{ijk}}$ values for each filter, represented by the average of $200\times200$ pixels in the lower right hand corner of the array, furthest from the horizontal shift register. The relative magnitude column reports $t_{sm,\mathit{ijk}}$ as a percentage of 6 ms exposure acquired in L0/R0 and scaled to an equivalent in-band radiance for other filters

Left Mastcam-Z				Right Mastcam-Z			
Filter	$\lambda _{\mathit{eff}}\ (\mathrm{nm})$	$< t_{sm,\mathit{ijk}}>$	Rel. Mag.	Filter	$\lambda _{\mathit{eff}}\ (\mathrm{nm})$	$< t_{sm,\mathit{ijk}}>$	Rel. Mag.
L0	530	0.6 ms	10%	R0	530	$ 0.6~\mbox{ms} $	10%
L1	800	1.7 ms	4.1%	R1	800	$ 1.7~\mbox{ms} $	4.0%
L2	754	0.8 ms	2.6%	R2	866	$ 3.0~\mbox{ms} $	4.7%
L3	677	0.6 ms	2.6%	R3	910	$ 4.1~\mbox{ms} $	5.2%
L4	605	0.3 ms	0.9%	R4	939	$ 4.7~\mbox{ms} $	4.2%
L5	528	0.2 ms	0.5%	R5	978	$ 5.7~\mbox{ms} $	2.3%
L6	442	0.4 ms	0.02%	R6	1022	$ 12.4~\mbox{ms} $	6.7%

Different filters have different magnitudes for $t_{sm,\mathit{ijk}}$, with a general trend toward larger values for longer wavelength filters. The R6 infrared filter (995–1033 μm) shows the largest magnitude, with $t_{sm,\mathit{ijk}}$ reaching 12.2 ms in the lower right corner of the array (furthest from the horizontal shift register). While the static bias is temperature dependent, maps of $t_{sm,\mathit{ijk}}$ generated from integrating sphere data acquired at $-5^{\circ }$ detector temperatures were equivalent to those generated at room temperature, suggesting that the dynamic bias is not temperature dependent. Table 4 below shows the maximum magnitude of $t_{sm,\mathit{ijk}}$ for each filter. Full maps of $t_{sm,\mathit{ijk}}$ for each filter are provided in Fig. 47 in Appendix A. The correlation between $t_{sm,\mathit{ijk}}$ and wavelength may be related to the increased penetration depth of longer-wavelength photons into the detector substrate. The maximal effect (lower right of Fig. 18C) occurs furthest from the readout location (upper left of Fig. 18C). While we do not have a physical explanation for this phenomenon at present, we are discussing it with the detector manufacturer and plan to include an updated explanation in a followup publication describing in-flight calibration activities. For a 6 ms exposure using L0/R0, the ghost image would represent up to $10\%$ of the scene flux in the lower right hand corner of the array (Fig. 18). While the magnitude of $t_{sm,\mathit{ijk}}$ increases for some filters, it increases less than exposure time required to obtain the same signal level as a 6 ms integration with L0/R0. As a result, the relative magnitude of the ghost image reduces to a maximum of 0.02–$6.7\%$ of a typical exposure on Mars, with a clear positive wavelength correlation (see Table 4). Regardless, if radiometric precision is required for an observation, we suggest acquiring a zero-second exposure that can be used to remove all three bias effects. Otherwise, an inability to accurately model the dynamic bias may introduce additive errors into the radiometric calibration pipeline and result in a minor increase to radiometric uncertainty, especially in regions distal from the horizontal shift register (see Fig. 3).

#### Bad Pixel Map

Of the 1600 × 1200 active pixels on each Mastcam-Z detector, only 14 in the left Mastcam-Z and 17 in the right Mastcam-Z have been identified as “bad” (Table 5). Bad pixels are identified as having a relative response that is $>2X$ or $<0.5X$ the average response of the central $100\times100$ pixels (see Sect. 3.7), or as having an intermittent temporal response. Typical bad pixels definitions include “hot” (saturated), “gray” (responsive, but at a higher or lower value than the average detector element), and “dead” (non-responsive). At the time of stand-alone calibration, the left Mastcam-Z had 1 intermittent “hot” pixel, 5 “gray” pixels, and 8 “dead” pixels. The right Mastcam-Z had 4 “gray” pixels and 13 “dead pixels,” all of which appear to be blocked by dust particles whose locations may or may not change by the time the rover gets to Jezero Crater. The left Mastcam-Z pixel (938,197) was observed to be “hot”, or saturated, in some frames while acting normally in others. This intermittent behavior had a long time constant, such that periods of either “hot” or normal activity tended to last for hours or days, as opposed to varying frame-by-frame. As such, there may be a way to recover this pixel when it is acting “normally” during flight if appropriate checks are incorporated into the radiometric calibration pipeline (Sect. 4.1). Other than the intermittent activity of pixel (938,197), no additional “hot” pixels were observed in either Mastcam-Z. In most cases, low response appeared to be the result of dust or other particulates on the CCD. These pixels will be re-evaluated after landing to see if pre-launch delivery, ATLO testing, launch and/or landing activities moved or even removed these particulates from the detector. Other bad pixels arose either from a low or marginal responsivity, as compared to the average pixel, or were completely non-responsive or blocked. While the marginal and low pixels may be recovered via appropriate flat field characterization, a response that is $>2$ times lower than the average pixel will necessarily have significantly lower signal, and thus much greater noise contributions than the nominal detector elements. In the calibration pipeline (Sect. 4.1), flags are placed to either pass images through with no bad pixel correction, remove pixels with out replacement, or remove bad pixels and replace them with an interpolation derived from the weighted average of their nearest-neighbors of the same Bayer filter-type (RGB). There was no filter dependence observed during bad pixel detection.

**Table 5:** Table of identified bad pixels. Row and column positions are zero-indexed from the upper left corner of the array’s active area, nearest the readout of the horizontal shift register (upper-left hand corner of Mastcam-Z images, as presented herein, and upper right-hand corner of Fig. 3, which is viewed from the above the detector)

Left Mastcam-Z			Right Mastcam-Z		
Row	Column	Note	Row	Column	Note
625	53	Dust	1178	352	Marginal (2X)
938	197	Intermittent	1178	353	Low (3X)
295	225	Dust	467	503	Dust
151	305	Dust	468	503	Dust
1058	634	Dust	465	504	Dust
227	795	Low (3X)	466	504	Dust
229	795	Marginal (2X)	467	504	Dust
232	796	Blocked	468	504	Dust
791	899	Blocked	465	505	Dust
1025	1200	Blocked	466	505	Dust
1024	1201	Marginal (2.4X)	467	505	Dust
1025	1201	Low (3X)	462	506	Dust
1025	1202	Opaque	463	506	Dust
263	1452	Marginal (2X)	464	506	Dust
			463	507	Dust
			277	895	Low (2.5X)
			1189	1141	Marginal (2X)

### Optical Throughput / Zoom

**Fig. 19:**
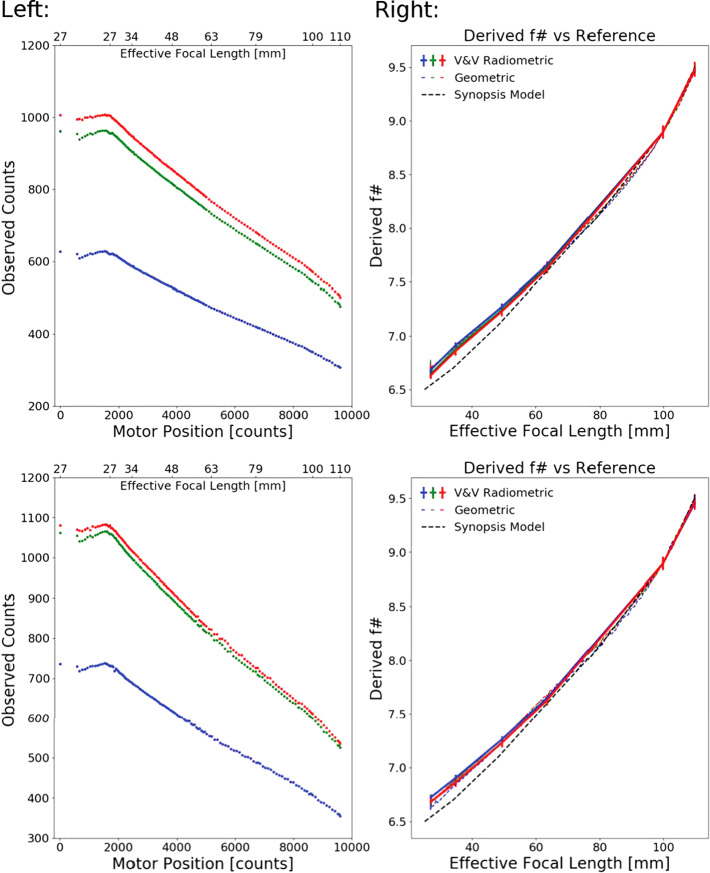
Observed DN as a function of focal length and the derived corresponding change in $f_{\#}$, with R, G, and B pixel values plotted in red, green and blue respectively. (**A**) Observed counts for a fixed scene in the geometric data. A sub-image constant to all zoom levels was used for the analysis to preserve illumination conditions. The lamps and target reflectance are such that the green and red pixels measured a comparable signal. (**B**) The derived $f_{\#}$ as a function of focal length, assuming a quadratic dependence on the change in $f_{\#}$ with effective focal length. Also plotted are the derived values of the $f_{\#}$ from data acquired during V&V testing with an integrating sphere at constant flux of 3.8 $\frac{\mathrm{mW}}{\mathrm{cm}^{2}\,\mathrm{sr}}$ and integration time of 20 ms. The conversion from motor position to focal length is taken from data acquired during V&V testing. See text for more details. (**C**) and (**D**) correspond to the same measurements as (**A**) and (**B**) but for the right Mastcam-Z

While most parameters of the camera equation (see Equation (1)), such as gain, spectral throughput and quantum efficiency, are independent of zoom position, the etendue or optical efficiency ($A_{o}\varOmega $) is not. For a circular aperture, such as Mastcam-Z, $A_{o}\varOmega $ can be expressed in terms of the effective $f_{\#}$ $(A_{o}\varOmega = \frac{\pi A_{d}}{4 f_{\#}^{2}})$, which varies with zoom position (i.e., focal length). As part of the geometric calibration on April 28$^{th}$ and 29$^{th}$, 2019 (see Sect. 3.11), images at 130 unique zoom positions and 116 unique focus positions were acquired of a stationary random dot target (see Sect. 3.2.2) under constant illumination. By maintaining a fixed illumination and holding all camera state parameters constant, other than zoom and focus, any measured change in incident flux is attributable to the zoom-dependent optical throughput ($A_{o}\varOmega $), expressed in terms of an effective optical $f_{\#}$. Because no test was conducted that can measure the absolute $f_{\#}$ of the cameras at a given zoom position, the $f_{\#}$ was anchored at 100 mm assuming $f_{\#_{100\mathrm{mm}}} = 8.9$, as specified by the optical performance model created by Synopsis (https://www.synopsys.com/optical-solutions.html) and presented at the Mastcam-Z Critical Design Review on November 14–15, 2016. Changes in $f_{\#}$ were then modeled (after bias subtraction) using the dependence:

4 $$ \begin{aligned} & A\varOmega _{l_{\mathit{ref}}} = \frac{\pi A_{d}}{4f_{\#l_{\mathit{ref}}}^{2}} \\ & A\varOmega _{l} = A\varOmega _{l_{\mathit{ref}}}\Big(\frac{f_{\#l_{\mathit{ref}}}}{f_{\#l}} \Big)^{2} \end{aligned} $$

where $A_{d}$ is the area of a single detector pixel ($A_{d}= 7.4$ μm^2^). As the term $A_{o}\varOmega $ is directly proportional to the DN measured by the camera (see Equation (1)), the change in DN as a function of zoom can therefore be used to measure the change in $f_{\#}$ as a function of zoom motor position. Fig. 19 plots the observed dependence of $f_{\#}$ with zoom. Images of the dot target were reduced to average DN values by finding the area observed at 110 mm subframe in all shorter focal lengths and averaging over the target in that sub-region. At 26 mm, this corresponded to $\sim 6\%$ of the frame. Recovered $f_{\#}$’s were spot-checked using images of the integrating sphere acquired at a variety of focal lengths (with fixed focus motor position) during V&V testing. Derived $f_{\#}$’s from the integrating sphere datasets matched those derived from the geometric calibration dataset (see Fig. 19).

### Spectral Throughput

Each Mastcam-Z camerahead is equipped with an eight-position filter wheel that is positioned close to the FPA (see Table 2). On each camera, Filter 0 (L0/R0) is an IR-cutoff filter that is used for direct Bayer RGB imaging, and Filter 7 (L7/R7) is a neutral density filter used for solar imaging at 590 ± 88 nm (ND6) and 880 ± 10 nm (ND5) on the left and right cameras, respectively. On May 5$^{th}$ and 6$^{th}$, 2019, the system-level throughput of each filter (CCD quantum efficiency + Bayer transmission + filter transmission + optical transmission) was characterized using an Oriel CS260 F/3.9 monochromator and 250W QTH light source (see Sect. 3.2.2). For each filter in each camera, with the exception of the solar filters, in-band response was determined from monochromator wavelengths sweeps conducted in 2 nm increments from $\sim 30$ nm before to $\sim 30$ nm after the expected cutoff wavelengths determined using component-level measurements. At each wavelength, 10 sub-frame images were acquired at 100 mm zoom around the monochromator slit with bias (zero-exposure) and dark (slit covered) frames collected at the beginning and end of each set. Prior to stepping through the wavelength sweeps for each filter, autofocus and autoexposure algorithms were commanded with the monochromator set to the filter’s central wavelength to determine an integration time (autoexposure target set to 60% full well) and best-focus position (targeted on the slit) for the run. Data validators were on shift to monitor data collection in real-time and report if any saturated frames (or other issues) were observed. In the event that an anomaly (e.g., saturated frame) was observed, the data collection was restarted with a new exposure time and/or focus position.

Following the in-band scan, an out-of-band scan was conducted using an exposure time $\sim 50X$ larger than the in-band scan to detect out-of-band leakage. The out-of-band scans were obtained from 300–1100 nm in 4 nm step sizes for L0/R0 and 6 nm step sizes for the narrowband filters. When a filter’s in-band response fell within a grating transition of the monochromator, a second scan was conducted with a different grating configuration to verify that grating transitions did not modify the observed response. Monochromator scans were not conducted for Filters L7 and R7 as the slit brightness was insufficient to transmit through the neutral density filters. Instead, estimates for the system-level passbands of the solar filters were derived from component-level measurements and vendor data. Note, however, that flat field and radiometric coefficients were derived at the system-level for the solar filters (see Sects. 3.6 and 3.7). Before and after data collection for each filter, calibration scans were conducted using a Newport 818-UV/DB radiometer (see Sect. 3.2.2) and used to spectrally flatten the monochromator output flux in postprocessing.

Postprocessing of the spectral throughput dataset was performed to characterize each filter’s in-band and out-of-band response and estimate the effective wavelength and bandwidth of each filter. The scripts used for postprocessing of the spectral throughput dataset are included in the SOM of this manuscript. Effective center wavelength ($\lambda _{\mathit{eff}}$) is defined as the weighted average of wavelength with the product of the normalized observed spectral response ($\bar{r_{\lambda ,k}}$) and solar radiance ($S_{o_{ph}}$) at the top of the Martian atmosphere in units of [$\frac{ph}{s\, m^{2}\, sr\, nm}$]:

5 $$ \lambda _{\mathit{eff}} = \frac{\int \bar{r_{\lambda ,k}} S_{o_{ph}} \lambda \,d\lambda }{\int \bar{r_{\lambda ,k}} S_{o_{ph}} \,d\lambda } $$

Filter width is defined as half of the width of the band-pass curve at half of the response maximum (half width at half maximum, or HWHM). Leakage is defined as the percent ratio between the integrated out-of-band response to the integrated in-band response. For our analysis $\lambda _{\mathit{on}}$ and $\lambda _{\mathit{off}}$, which define the transition between in-band and out-of-band response, are defined as the nearest wavelengths on either side of $\lambda _{\mathit{eff}}$ that display $1\%$ of the peak response. At each wavelength, the average R, G, and B pixel values were obtained from an average of the 10 frames collected at each wavelength. Prior to pixel extraction, bias frames were subtracted from the average frame, flat field corrections were applied, and a pixel mask was used to ensure that only valid pixels from within the slit were included. For the integration times used, dark current was negligible and the dark current frames were statistically indistinguishable from the bias frames. The average response for each RGB Bayer filter was then divided by the commanded integration time to determine relative spectral response. If a grating transition occurred within the in-band response, then the response from the second monochromator sweep run for that filter using a different grating scheme was used to verify consistency and remove any grating transition effects from the data. The resulting spectral response curves were then normalized. The normalized spectral response ($\bar{r}_{\lambda ,k}$) was then multiplied by the appropriate radiometric coefficient ($r_{o,k}$) to generate the convolved spectral response ($r_{ \lambda ,k}$) in units of $[\frac{e^{-}}{ph}]$ (see Sect. 3.7). Note that, while the spectral scans were derived from data acquired using 100 mm zoom, $r_{\lambda ,k}$ is independent of zoom. The radiometric effects of changing zoom are accommodated by optical throughput ($A_{o} \varOmega _{l}$) variations (see Sect. 3.4). Comma-Separated Value files containing the convolved spectral response ($r_{\lambda ,k}$) for each filter are available in the SOM of this manuscript.

**Fig. 20:**
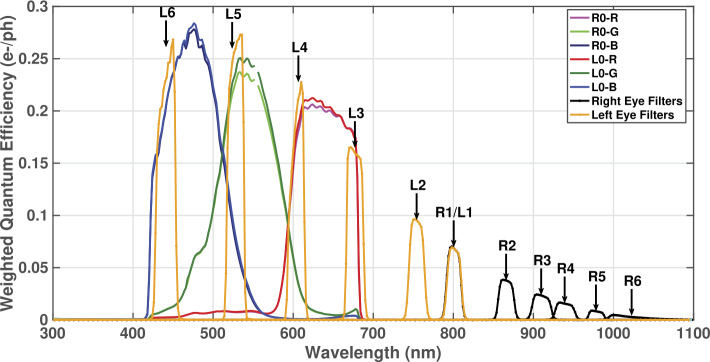
Mastcam-Z Spectral Response ($r_{\lambda ,k}$) for filters L0-L6 and R0-R6 in linear space at a detector temperature of $-5^{\circ }~\mathrm{C}$. For the purposes of this plot, only the dominant RGB Bayer response is shown any given filter, except L0/R0. Data files containing the filters profiles are included in the SOM of the manuscript

Fig. 20 shows the spectral response curve ($r_{ \lambda ,k}$) for each filter. The same data is depicted in logarithmic scaling in Appendix Fig. 44. Table 2 lists the effective center wavelength, HWHM, and leakage for each filter. Leakage magnitudes are $<1\%$ for all filters. When multiple scans of a filter were acquired the resulting profiles, before scaling by $r_{o,k}$, generally agreed to better than $1\%$. The only area that this was not true for is around the monochromator’s 652 nm grating transition, where some scans showed saw-toothed dips in the response for L0/R0. Utilizing a grating scheme that did not have a grating transition in this area subsequently removed this effect. We note that a similar saw tooth pattern is observed at ∼652 nm in the MSL Mastcam spectral profiles (see Fig. 3 in Bell et al. 2017), suggesting that the feature may be related to a grating transition and not Mastcam’s actual spectral response. Relative to the MSL Mastcam spectrum published in Bell et al. (2017), Mastcam-Z appears to have a shorter wavelength cutoff: $\lambda _{\mathit{eff}}$ of 493.5 nm vs. 480 nm, respectively. As Mastcam and Mastcam-Z use the same KAI-2020 CCD and IR-cutoff filter (L0/R0), the blue wavelength cutoff was expected to be the same. The Mastcam-Z response is more in line with vendor-provided quantum efficiencies and filter transmission curves (see Fig. 46). Looking at the filter ratio of a dust storm deep orange sky to a blue Sun on a clear day on Mars, comparison between MSL narrowband and broadband filters suggests an effective wavelength of $\sim 482$ for the blue Bayer channel, consistent with the measured spectral response of Mastcam-Z. A final observation in the Mastcam-Z spectral response is that L4, L5, and L6 appears to have a slightly higher response than the L0 Bayer pixels. To verify this observation, multiple scans at L4, L5, and L6 and L0 were conducted, all of which showed the same result. This result was slightly surprising, and suggests that the IR cut-off filter has a lower peak transmission as compared to the visible-wavelength narrowband filters. Note that, while a temperature dependence was observed in the derived radiometric coefficients ($r_{o,k}$), testing with the MASI system verified that is was primarily a scaling factor and that the observed filter profiles were not appreciably affected (see Sect. 3.7.1). Effective wavelengths and HWHM calculations did not change with temperature at a level to which we could determine them (∼1 nm).

### Flatfield Characterization

Flatfield correction is a standard technique for removing pixel-to-pixel sensitivity differences that result from detector responsivity variations and optical features such as vignetting, filter imperfections, and dust. Flat field images are derived from uniformly illuminated (i.e., “flat”) targets that highlight sensitivity variations within the field of view. Bias and dark current corrected images are multiplied by normalized flatfield maps in an attempt to cancel the effect of these variations and make every pixel respond like a fictitious average pixel. The average pixel is typically defined as the mean response of a central $200\times200$ pixel area of the detector (excluding bad pixels). On May 4$^{th}$–5$^{th}$, 2019, both Mastcam-Z cameras viewed the uniform output of the integrating sphere (see Sect. 3.2.2) under ambient conditions. In order to eliminate the need for multiple data collections, the sphere was placed close enough to the front aperture of each camera to ensure that the full field of view (FOV) was illuminated at 34 mm focal length ($\mathit{zoom}_{mc}$ of 2448). For each filter, 10 identical 11-bit frames were acquired at three integration times and two flux levels (ISOP = 5 $\frac{\mathrm{mW}}{\mathrm{cm}^{2}\,\mathrm{sr}}$ and ISOP = 10 $\frac{\mathrm{mW}}{\mathrm{cm}^{2}\,\mathrm{sr}}$). At the start of each 10-frame sequence, an autoexposure was acquired to the find integration times equivalent to 30% and 60% full well, respectively. The third integration time was a set of zero-second bias frames. This dataset was repeated at 34 mm ($\mathit{zoom}_{mc}=2448$), 63 mm ($\mathit{zoom}_{mc}=5196$), and 100 mm ($\mathit{zoom}_{mc}=8652$). On April 27$^{th}$, 2019, an identical dataset was acquired in the MSSS TVAC chamber at both ambient and −10^∘^ C (FPA at $-5^{\circ }$). While the chamber was at $-10^{\circ }$ C, images of the solar filters (L7/R7) were acquired with the integrating sphere at full power ($\mathrm{ISOP} = 80~\frac{\mathrm{mW}}{\mathrm{cm}^{2}\,\mathrm{sr}}$) to obtain sufficient signal for postprocessing. In the calibration plan and image logs, these series of observations are labeled as radiometric testing (see Table 3). Note that the impediment of the chamber window necessitated an increased distance between the cameras and integrating sphere during thermal vacuum testing, so the full field of view was not illuminated in those datasets at 100 mm.

**Fig. 21:**
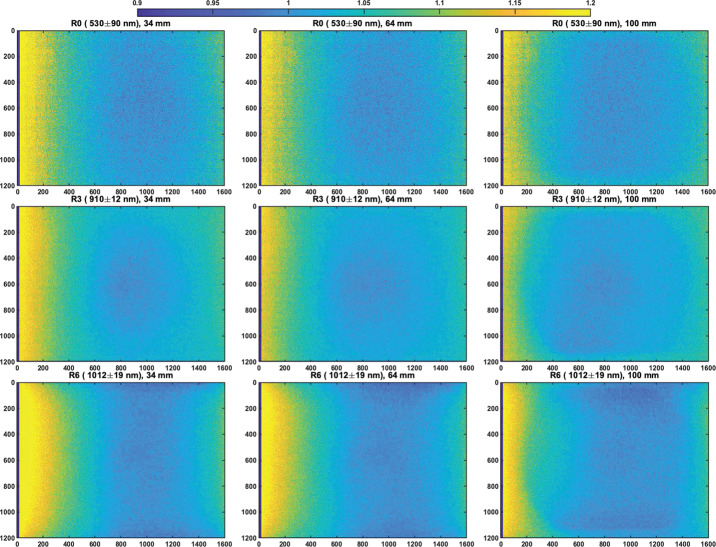
Flatfield correction maps for filters R0, R3, and R6 at zoom levels of 34 mm (left, $\mathit{zoom}_{mc}=2448$), 63 mm (middle, $\mathit{zoom}_{mc}=5196$), and 100 mm (bottom, $\mathit{zoom}_{mc}=8652$). Note that the vignetting on the left-hand-side of the array, likely caused by the edge of the filter encroaching into the field of view, decreases for longer focal lengths

Postprocessing of the radiometric test dataset included averaging the 10-frame image stacks for each filter, integration time, and flux level. The average frames were then bias-subtracted, dark-corrected, and response ($\mbox{DN}/\mbox{ms}$) arrays were created by dividing the difference between the two average frames for each flux level by the difference in commanded integration times. The difference between the two response ($\mbox{DN}/\mbox{ms}$) arrays was then divided by the difference between the average response ($\mbox{DN}/\mbox{ms}$) of a $200\times 200$ pixel box centered on the array that was devoid of bad pixels. The average response in the central box was determined for the red, green, and blue Bayer pixels independently. The scripts used to generate the flatfield correction maps are provided in the SOM. In the resulting flatfield correction maps (see Fig. 21), both small-scale pixel-to-pixel responsivity variations and larger scale optical effects can be seen. The 4-pixel repetitive Bayer-pattern (see Fig. 3), however, is not visible in most filters confirming that dividing by the average response of each Bayer RGB channel group separately has effectively removed the effects of the Bayer pattern filter from the normalized response. For shorter wavelengths where particular Bayer pattern filters are spectrally blocked by the narrowband filter (e.g., L6), the Bayer pattern shows up in the flatfield due to a lack of signal. Dividing the appropriate flatfield correction map ($F_{\mathit{ijkl}}$) by a bias subtracted and dark correct image frame of a uniform source will nominally produce a uniform array for each Bayer channel with a value equivalent to the average of a central $200\times200$ pixel box in the uncorrected image. For filters with in-band wavelengths $>\sim 800$ nm, the Bayer filters are transparent and the radiation incident on the detector is uniform.

**Fig. 22:**
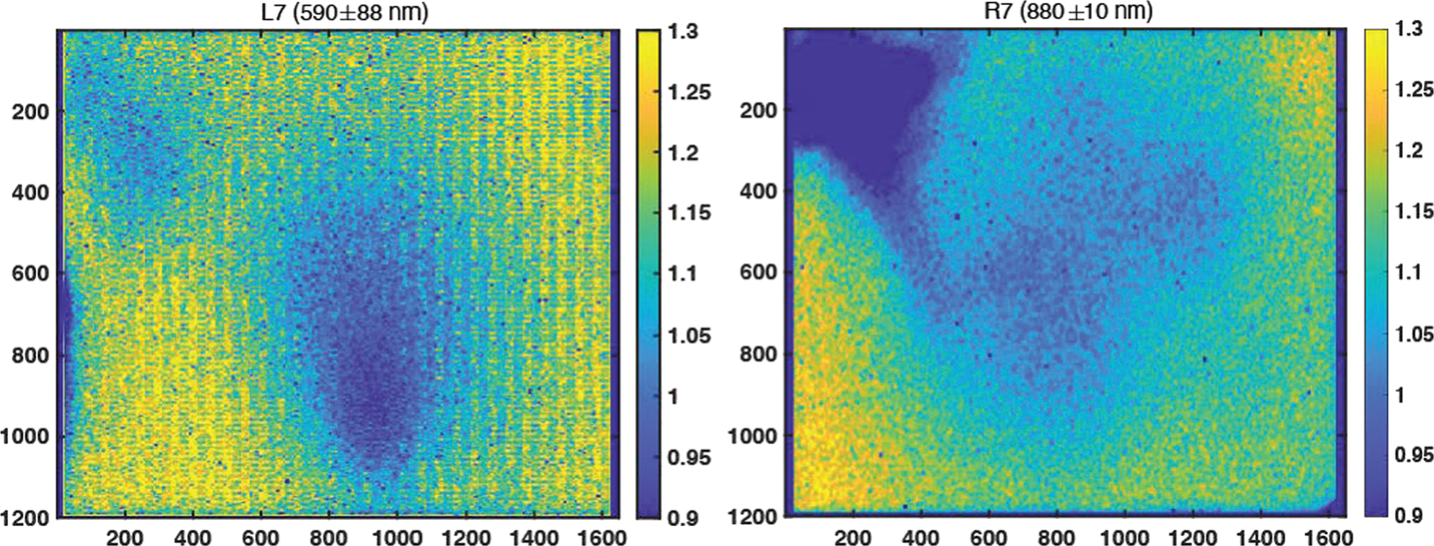
Flatfield correction maps for the solar filters L7 and R7 at 100 mm ($\mathit{zoom}_{\mathit{cnt}}=8652$) focal length. Note the pinhole defect in the upper-left-hand corner of the R7 flatfield

Nonuniformity is statistically defined as the ratio of the standard deviation to the mean. When the entire array is considered, the Mastcam-Z flatfields have nonuniformity values ranging from 3.4%–6.3%. Statistically, there is little variation in the reported nonuniformity with filter or zoom, suggesting that pixel-to-pixel responsivity variations are dominating the standard deviation. When only the central $200\times200$ pixel box is considered, the nonuniformity falls within the range 1.7%–2.4% across all filters and tested zoom levels. Maximum flatfield variations, on the other hand, are up to $25\%$ near the edge of the field where the filter begins to vignette the CCD’s rectangular FOV (see Fig. 21). As expected, the boundary of this effect moves outward for longer focal lengths as the fields of view contracts. Fig. 21 shows flatfield maps for 34 mm, 63 mm, and 100 mm and filters R0, R3, and R6. Fig. 22 shows the resulting flatfields for the L7 and R7 solar filters. Note that the R7 solar filter appears to have a pinhole defect near the upper left hand corner of the array. This area of the array should be avoided for solar observations that require radiometric accuracy. For the portion of the FOV that was illuminated at each zoom position, flatfields generated from observations of the integrating sphere viewed through the TVAC chamber window were identical to those collected at ambient (to within error), suggesting that the relative flatfield coefficients are temperature invariant. Flatfield correction maps for the remaining filters and zoom levels are provided in Sect. A.5 (Figs. 48–54).

Due to time constraints the radiometric dataset was limited to three zoom positions. A second dataset, described in Sect. 3.4, was acquired at 67 zoom positions over the full range of 26 mm ($\mathit{zoom}_{mc}=0000$) to 110 mm ($\mathit{zoom}_{mc}=9600$) with the integrating sphere as close to the front aperture as safely possible (fully filling the 26 mm FOV). This dataset, known as the continuous zoom test (see Table 3), acquired 8-bit frames at ISOP = 5 $\frac{\mathrm{mW}}{\mathrm{cm}^{2}\,\mathrm{sr}}$ and ISOP = 10 $\frac{\mathrm{mW}}{\mathrm{cm}^{2}\,\mathrm{sr}}$ for filters L0/R0. Similar to the radiometric dataset, three integration times were acquired at each flux level. Using the method described above flatfield correction maps were generated for L0/R0 at all 67 zoom positions. Composite flatfields can then be approximated for narrowband filters at arbitrary zoom positions by:

6 $$ F_{\mathit{ijkl}} = F_{\mathit{ijkl}_{\mathit{ref}}} \frac{f_{ij0l}}{f_{ij0l_{\mathit{ref}}}}, $$

where $F_{\mathit{ijkl},\mathit{ref}}$ is the filter dependent flat-field at one of the three reference zoom positions available in the radiometric dataset, and $f_{ij0l}$ is the L0/R0 flat-field from the “continuous zoom” data-set, which has been median filtered to reduce pixel-to-pixel noise resulting from 11-to-8-bit companding and the smaller number of frames (1 vs. 10) obtained at each flux level and integration time. Please see Sect. 4.1.5 for a discussion of this process. Equation (6) is an approximation that applies the smoothly varying zoom-dependence contained in $f_{ij0l}$ while retaining the high frequency pixel-to-pixel variability captured by $F_{\mathit{ijkl}_{\mathit{ref}}}$. It should be noted, however, that the continuous zoom dataset was only collected for the R0/L0 filters. Therefore, Equation (6) necessarily assumes no filter dependence on the low-frequency component of the flatfield. At 100 mm, approximate flatfields generated for all non-L0/R0 filters using Equation (6) showed average differences of $<1\%$ and standard deviations of $3\%$–$4\%$ as compared to the actual 100 mm flatfields calculated from the radiometric dataset. For these tests, the approximate flatfields ($F_{\mathit{ijkl}}$) were generated using measured flatfields at 63 mm zoom from the radiometric datasets ($F_{\mathit{ijkl},\mathit{ref}}$) and L0/R0 100 mm and 63 mm flatfields from the continuous zoom dataset ($f_{ij0l}$). While, based on this test, flatfields derived using Equation (6) appear to compare favorably to those calculated directly from the radiometric dataset, we caution the use of non-reference zoom positions for datasets where radiometric accuracy is critical.

### Radiometric Coefficients

The radiometric dataset used to generate flatfield coefficients (Sect. 3.6) was also used to estimate the radiometric coefficient $r_{o,k}$ $[ \frac{e^{-}}{ph}]$ required to convert observed DN values into observed physical radiance $\langle L_{\lambda } \rangle _{\mathit{ijkl}}$ [$\frac{\mathrm{W}}{\mathrm{cm}^{2}\, \mathrm{sr}}$] using Equation (2). As defined in Equation (1), $r_{o,k}$ is independent of zoom position and accounts for the combined effects of transmission through the optical path (including the lenses, filter, and Bayer pattern) and detector quantum efficiency. The optical throughput ($A_{o}\varOmega _{l}$, see Sect. 3.4) accounts for changes in zoom position through a changing $f_{\#,k}$. In order to determine $r_{o,k}$, the $\mbox{DN}/\mbox{ms}$ response describe in Sect. 3.6 was multiplied by the detector gain ($g$, see Sect. 3.3.1), divided by the optical throughput ($A_{o}\varOmega _{l}$, see Sect. 3.4), and divided by the integrated integrating sphere flux (see Sect. 3.2.2) weighted by the normalized spectral response ($\bar{r_{\lambda ,k}}$, see Sect. 3.5). For datasets acquired in the thermal vacuum chamber, a correction was applied for the window transmission (see Table 14 in Appendix A). This correction, which varies between $0.898\pm 0.005$ for R6 and $0.95\pm 0.006$ for L3, was determined from constant illumination observations of a 116-patch Macbeth color target imaged on May 1$^{st}$ with and without the chamber window in-place (see Table 14). Correction values are consistent with expectations for bare (i.e., no anti-relection coating) borosilicate glass.

Radiometric coefficients were independently derived for all three focal lengths acquired during each radiometric dataset in both the cleanroom and TVAC chamber (see Sect. 3.6) and, with one exception, results from different zoom positions and flux values were consistent to within error (see Fig. 43 in Appendix A). The 100 mm dataset acquired in the cleanroom with the sphere directly up against the camera (9 cm distance), however, resulted in derived radiometric coefficients that were uniformly $10\%$ higher than those derived from other tests in the thermal vacuum chamber at ambient or $-10^{\circ }\ \mathrm{C}$. This increased sensitivity was wavelength independent, suggesting either that the integrating sphere was generating more flux than expected or the proximity of the sphere to the camera was introducing wavelength-independent stray light at this particular zoom position. Review of the calibration logs revealed that the integrating sphere’s radiometer readout was slowly increasing during the script execution, likely resulting from light reflected off the camera’s front aperture and back into the sphere due to their relative proximity (M. Barr and L. Dobrowski; Labsphere, personal communication, February 18$^{th}$, 2020). While the sphere was still emitting a spatially uniform flux, it is unclear whether the recorded flux readings were accurate. As such, we do not use this dataset in derivation of the radiometric coefficients. Fortunately, the 100 mm dataset in question is redundant with the 100 mm datasets acquired in the chamber at ambient and $-10^{\circ }~\mbox{C}$, as well as a subset of the photon transfer tests in both the cleanroom and chamber that were acquired at 100 mm for L0/R0. All of these other datasets produce radiometric coefficients that, for the same temperature, are consistent to within error (see Fig. 43). In Table 6, we report the radiometric coefficients derived from thermal vacuum testing at a $-10^{\circ }~\mathrm{C}$ chamber temperature ($-5^{\circ }$ C detector temperature), which is representative of the average operational temperature on Mars. The ambient radiometric coefficients, determined from all datasets other than the 100 mm close-proximity dataset discussed above, are provided in Table 13. As added validation, radiometric coefficients for L0/R0 were also derived from the photon transfer dataset (see Sect. 3.3.1) and found to be identical, to within error, to those derived from the radiometric dataset acquired at ambient in the vacuum chamber (see Fig. 43). Similarly, the derived spectral response ($r_{\lambda ,k}=r_{o,k}\, \bar{r}_{\lambda ,k}$) is consistent with predictions made by combining the vendor-provided optical transmission, filter transmission, and detector quantum efficiency (see Figs. 45 and 46).

**Table 6:** Mastcam-Z Radiometric Calibration Coefficients ($r_{o,k}$)^a^ at a detector temperature of $-5^{\circ}$ C

Filter	Effective wavelength	Red		Green		Blue	
	±HWHM (nm)	$r_{o,k}$ $[\frac{e^{-}}{ph}]$	*β* [10^−3^/C]	$r_{o,k}$ $[\frac{e^{-}}{ph}]$	*β* [10^−3^/C]	$r_{o,k}$ $[\frac{e^{-}}{ph}]$	*β* [10^−3^/C]
L0	530±90	0.202±0.0056	1.08±0.05	0.24±0.0058	0.18±0.06	0.273±0.006	0±0.1^b^
L1	800±9	0.0665±0.0019	2.24±0.05	0.0443±0.0013	1.86±0.05	0.0425±0.0013	0±0.06^b^
L2	754±10	0.0925±0.001	1.83±0.05	0.03±0.001	1.68±0.07	0.000719±0.001	0.00±0.05^b^
L3	677±11	0.16±0.0007	1.76±0.05	0.0109±0.00068	0.18±0.05	0.00275±0.00067	0.00±0.05^b^
L4	605±9	0.217±0.00049	0.75±0.05	0.0621±0.0005	2.57±0.05	0.00106±0.0005	0.00±0.05^b^
L5	528±11	0.0111±0.00028	0.54±0.05	0.264±0.00028	0±0.05^b^	0.1±0.00028	1.28±0.05
L6	442±12	0.0047±0.00013	0.00±0.05	0.0175±0.00014	0±0.05^b^	0.256±0.00013	−1±0.5
L7^c^	590±88	1.97e-07±1.52e-08	0.85±0.10	1.44e-07±4.01e-08	0.89±0.50	1.31e-07±2.84e-08	0.85±0.50
R0	530±90	0.196±0.0056	1.08±0.05	0.226±0.0059	0.18±0.06	0.267±0.0059	0.0±0.1^b^
R1	800±9	0.0668±0.0017	2.24±0.05	0.0437±0.0011	1.86±0.05	0.042±0.0011	0.00±0.06
R2	866±10	0.0364±0.0021	3.01±0.05	0.0352±0.00063	2.88±0.05	0.0352±0.00019	2.96±0.05
R3	910±12	0.0231±0.0031	3.56±0.05	0.0229±0.00016	3.51±0.05	0.0227±0.00025	3.53±0.05
R4	939±12	0.0155±0.0057	4.19±0.05	0.0156±0.0012	4.2±0.05	0.0155±0.00057	4.18±0.05
R5	978±10	0.00825±0.00015	5.56±0.05	0.00835±0.0053	5.45±0.05	0.00819±0.002	5.46±0.05
R6	1022±19	0.00429±0.00022	7.43±0.05	0.00439±0.00077	7.47±0.05	0.00432±0.0063	7.45±0.05
R7^c^	880±10	2.57e-07±2.43e-08	3.40±0.50	2.57e-07±4.20e-08	3.20±0.50	2.50e-07±3.10e-08	3.30±0.50

^a^The $r_{o,k}$ reported here are the peak response, which must be multiplied by the peak normalized $\bar{r_{\lambda ,k}}$ to obtain $r_{\lambda ,k}$

^b^These *β* coefficients are set to zero because of large relative uncertainties

^c^To derive solar filter radiometric coefficients, the vendor provided spectral profile were used

At both $-10^{\circ }~\mbox{C}$ and ambient temperatures, $r_{o,k}$ $[ \frac{e^{-}}{ph}]$ was derived to within a $1\sigma $ uncertainty of $\sim 3\mbox{--}5\%$ for L0/R0 and $\sim 1\mbox{--}3\%$ for each narrow-band filter’s dominant Bayer channel (see Table 6). This error propagates uncertainties in $\bar{r}_{\lambda ,k}$ (±∼ 1 nm), integrating sphere radiance (±∼ 1%), derived gain ($g=15.6\pm 0.2$ $\frac{e^{-}}{s}$), chamber window transmission ($\sim 1\%$), error in estimates of $f_{\#, l}$, and the Poisson noise in the observed camera response ($\sigma _{\mathrm{DN}/\mathrm{ms}} \sim \sqrt{\mathrm{DN}/\mathrm{ms}}$). Radiometrically calibrating L0/R0 integrating sphere images from the continuous zoom test (see Sect. 3.4), at zoom positions not acquired in the radiometric dataset, recovered ISOP values to an absolute accuracy of $4-5\%$, consistent with this error budget. When using 34 mm, 63 mm, and 100 mm focal lengths, radiometric coefficients can also be derived in the canonical units of [$\frac{\mathrm{W}\,\mathrm{m}^{-2}\, \mathrm{nm}^{-1}\, \mathrm{sr}^{-1}}{\mathrm{DN}\, \mathrm{s}^{-1}}$] (see Table 7). These values are not zoom-invariant, but do allow comparison to previously reported calibrations for fixed focal length systems (e.g. Bell et al. 2006, 2017). Since the gain and optical throughput are incorporated within the radiometric coefficient when presented in these units, the formal error decreases. As a result, the radiometric accuracy of Mastcam-Z will be higher when observing at 34 mm, 63 mm, or 100 mm.

**Table 7:** Mastcam-Z radiometric calibration coefficients for 34 mm and 100 mm focal lengths in units of (W/m^2^/nm/sr)/(DN/s) at a detector temperature of $-5^{\circ}$. Note that these results can be scaled to other focal lengths by scaling for changes to optical throughput ($A_{o}\varOmega _{l}$, see Sect. 3.4)

Filter number	Effective center wavelength (nm)	Red	Green	Blue
		[(W/m^2^/nm/sr)/(DN/s)] ±1*σ* uncertainty		
*34 mm zoom*^a^				
L0	530	2.99e-07±8.18e-09	2.8e-07±7.71e-09	2.98e-07±8.39e-09
L1	800	3.33e-06±3.74e-08	4.99e-06±5.69e-08	6.34e-06±6.68e-08
L2	754	2.39e-06±2.56e-08	7.74e-06±9.64e-08	0.000325±1.24e-05
L3	677	1.44e-06±1.76e-10	2.81e-05±2.37e-07	8.47e-05±1.43e-06
L4	605	1.5e-06±1.3e-08	6.33e-06±5.8e-08	0.000702±4.03e-05
L5	528	2.62e-05±5.13e-07	1.17e-06±1.92e-08	4.01e-06±6.81e-08
L6	442	6.83e-05±7.43e-07	2.28e-05±2e-07	1.36e-06±5.89e-09
L7^b^	590	-±-	-±-	-±-
R0	530	3.02e-07±1.13e-08	2.89e-07±1.18e-08	3.01e-07±1.39e-08
R1	800	3.27e-06±1.16e-08	4.95e-06±1.81e-08	6.39e-06±5.52e-09
R2	866	5.14e-06±1.61e-08	5.29e-06±1.5e-08	5.27e-06±1.37e-08
R3	910	6.56e-06±3.91e-08	6.62e-06±3.95e-08	6.66e-06±3.92e-08
R4	939	9.27e-06±9.3e-09	9.26e-06±1.15e-08	9.31e-06±1.43e-08
R5	978	2.18e-05±1.73e-07	2.16e-05±1.71e-07	2.15e-05±1.71e-07
R6	1022	1.68e-05±6.87e-08	1.68e-05±6.42e-08	1.68e-05±7.26e-08
R7^b^	880	-±-	-±-	-±-
*100 mm zoom*^a^				
L0	530	5.02e-07±1.65e-08	4.73e-07±1.58e-08	5.04e-07±1.84e-08
L1	800	5.63e-06±7.84e-08	8.44e-06±1.24e-07	1.07e-05±1.47e-07
L2	754	4.05e-06±3.58e-08	1.31e-05±1.25e-07	0.000387±1.22e-05
L3	677	2.46e-06±5.86e-09	4.83e-05±4.46e-07	0.000174±4.07e-06
L4	605	2.52e-06±4.43e-08	1.08e-05±2.13e-07	0.000638±2.01e-05
L5	528	4.49e-05±5.43e-07	2e-06±1.52e-08	6.85e-06±6.38e-08
L6	442	0.000111±1.97e-06	3.71e-05±1.62e-07	2.28e-06±8.91e-09
L7^c^	590	3.13e-01±2.39e-02	4.80e-01±1.33e-02	5.71e-01±1.24e-01
R0	530	5.06e-07±7.55e-09	4.86e-07±5.07e-09	5.09e-07±3.17e-09
R1	800	5.49e-06±1.39e-08	8.29e-06±1.76e-08	1.07e-05±1.24e-08
R2	866	8.61e-06±9.7e-09	8.86e-06±8.04e-09	8.83e-06±6.72e-09
R3	910	1.1e-05±2.38e-08	1.11e-05±2.16e-08	1.12e-05±2.3e-08
R4	939	1.54e-05±7.43e-08	1.54e-05±7.17e-08	1.55e-05±7.56e-08
R5	978	3.62e-05±7.79e-08	3.59e-05±7.22e-08	3.56e-05±6.29e-08
R6	1022	2.79e-05±4.34e-09	2.8e-05±9.34e-09	2.8e-05±8.34e-09
R7^c^	880	9.93e-01±9.30e-02	9.91e-01±1.61e-01	1.02e-00±1.26e-01

^a^The coefficients reported here are subject to the same temperature dependence and *β* parameters as those presented in Table 6

^b^Solar radiometric coefficients were only measured at 100 mm focal length

^c^To derive solar filter radiometric coefficients, the vendor provided spectral profile was used instead of measured $\bar{r_{\lambda ,k}}$

While $r_{o,k}$ values derived from different focal lengths and flux values in the radiometric and continuous zoom datasets were self-consistent, the coefficients derived at $-10^{\circ }\ \mathrm{C}$ ($-5^{\circ }\ \mathrm{C}$ detector temperature) and ambient in the TVAC dataset were not. Specifically, the ratio between $r_{o,k}$ values derived at $-10^{\circ }$ C and ambient resulted in up to a $35\%$ increase in sensitivity at the warmer temperature (see Sect. 3.7.1). This effect was observed to be wavelength dependent, with the near-infrared filters (e.g., R6) showing a substantially larger effect than the shorter wavelength filters (e.g., L6). In order to investigate this effect further, a test campaign was conducted using the MASI Mastcam-Z simulator (see Sect. 3.2.2). These tests showed that the temperature dependence can be described by a linear scaling factor, $\beta $, depicted in Table 6 alongside estimates of $r_{o,k}$ for Mars-like conditions ($-5^{\circ }~\mathrm{C}$ detector temperature).

#### Temperature Dependence

**Fig. 23:**
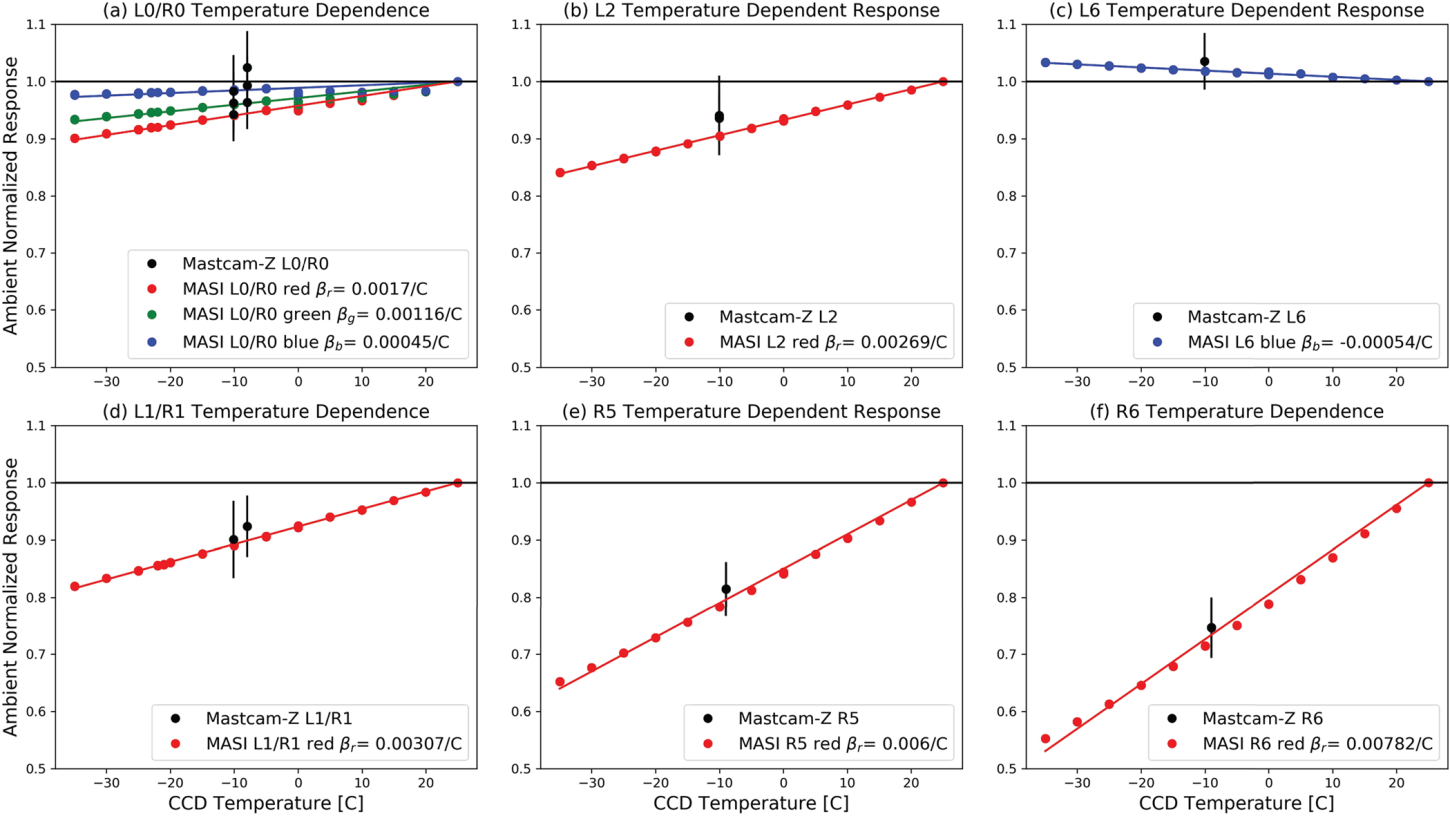
Temperature dependent radiometric responsivity of the MASI Mastcam-Z simulator. Radiometric coefficient variability was determined to be linear with temperature in the $-35^{\circ }$ C to $+25^{\circ }$ C range expected on Mars. Here we compare the single temperature measurements of the Mastcam-Z flight units (black points) to a more complete data set taken with the MASI. Six of the most representative filters are shown. Panel (**a**) shows the red, green, and blue pixel ratios for the broadband filters L0/R0, (**b**) for L2 for which only the red pixels are illuminated, (**c**) for L6 for which only the blue pixels are illuminated, (**d**) for the 800 nm L1/R1, (**e**) for R5, and (**f**) for the R6 broadband infrared filter with the greatest effect

A temperature dependence of the Mastcam-Z system spectral response became apparent during TVAC radiometric tests at non-ambient conditions on April 30$^{th}$, 2019. The derived radiometric coefficients for the near-infrared filters were up to $35\%$ more sensitive for the ambient tests as compared to those at −5^∘^ C detector temperatures (see Fig. 23). The effect was negligible at visible green, grew monotonically in the infrared, and even seemed to reverse for the blue filters. A similar effect was observed in the Mitel CCDs used for the Mars Exploration Rover cameras, which shows increased near-IR sensitivity and decreased blue sensitivity at higher temperatures (Herkenhoff et al. 2004). This effect is likely caused by the increased photon absorption length that results from an increase in the silicon bandgap as detector temperature is reduced (see Janesick 2001, Sect. 3.2.3.3). As observed in Mastcam-Z, this effect is most pronounced for near-infrared photons and known to result in a linear dependence of sensitivity with temperature. Interestingly, this effect has the opposite sign to the temperature dependence observed in most infrared and near-infrared detectors, where colder temperatures lead to a decrease in semiconductor band-gap and corresponding increase in long-wave sensitivity (Razeghi and Henini 2002).

Mastcam data from Mars further support the proposition that there is a temperature-dependence of response inherent to the detector. We investigated Mastcam images of the calibration target (Bell et al. 2017) over sols 1033–1039 (while the rover was stationary) that were taken with the Sun above $75^{\circ }$ elevation and all filters. Over a $2.3^{\circ }$ C temperature range, the 445-nm radiance decreased relative to 867-to-1012-nm radiances by $1.0\mbox{--}1.5\%$ per degree of warming, although other variables such as azimuth to the Sun were also changing. We also investigated Mastcam images of the Sun in 440-and-880-nm filters over sols 33-1400. The blue/IR ratio decreased by ${\sim} 9\pm 3\%$ over 35 degrees of warming, after correction for transmission through the atmosphere of ${\sim} 2\%$ per unit path optical depth (Lemmon et al. 2019).

**Fig. 24:**
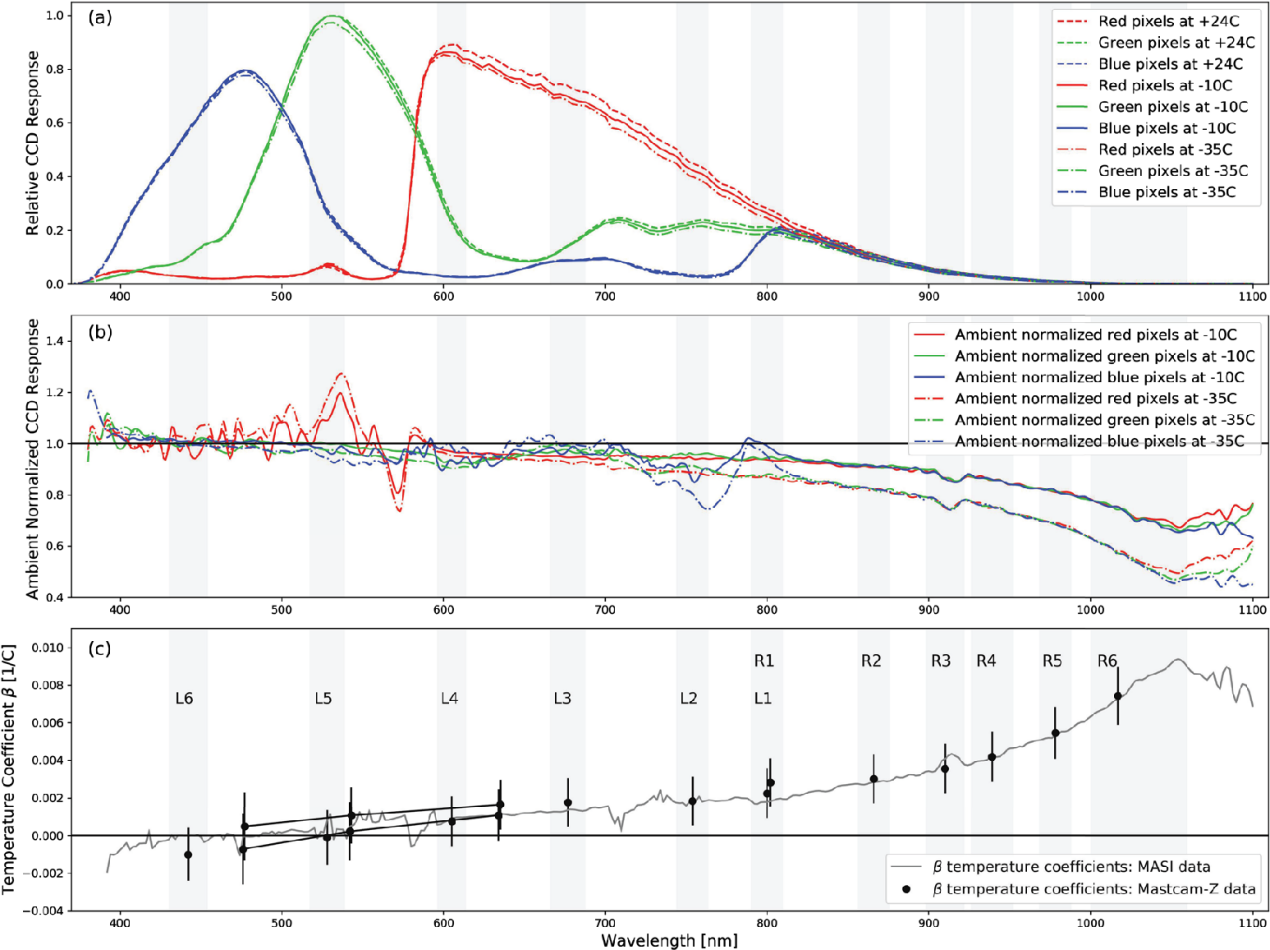
Three monochromatic scans at varying temperature confirmed an approximately linear decrease of quantum efficiency for wavelengths between 400 nm and 1100 nm. Panel (**a**) is the normalized response of MASI at three temperatures when the mono-chronometer’s light was focused on the detector without any filters in the beam. Notice that each of three Bayer-pattern colors behave differently until about 800 nm. Although MASI’s response greatly decreases in the infrared, there is enough signal for a robust measurement up to $\sim 1060$ nm. Panel (**b**) shows the $-10^{\circ }$ C and $-35^{\circ }$ C curves divided by the ambient $+24^{\circ }$ C curves. Panel (**c**) shows MASI’s linear fitting of the temperature coefficient versus wavelength. The black dots are approximate measurements derived from the TVAC tests with Mastcam-Z. The disagreement between filters 0 and 1 is likely the result of slight difference in ISOP values during testing

On August 15$^{th}$, 2019, monochromator scans and integrating sphere observations were acquired using the MASI Mastcam-Z simulator (Sect. 3.2.2) at detector temperature ranging from $-40^{\circ }~\mathrm{C}$ to $+25^{\circ }~\mathrm{C}$ at Arizona State University. MASI is built around the same KAI-2020 detector as Mastcam-Z and utilizes Mastcam-Z flight spare filters, although the electronics are COTS parts that may not act the same as the MSSS DEAs. MASI’s detector is packaged in a temperature-controlled chiller that can be set between $-50^{\circ }$ C and $50^{\circ }$ C. The same monochromator (Sect. 3.2.2) and integrating sphere (Sect. 3.2.2) used for flight unit testing were also used for the MASI experiment. Data collection and post-processing were performed using the methods described in Sects. 3.5 and 3.7, respectively. Fig. 23 shows the relative changes observed in radiometric response for a collection of filters. In each case, the changes in the radiometric coefficient were observed to be linear with temperature. Furthermore, the relative change observed in the flight unit radiometric response matched the relative change observed in MASI at the same temperature (see black points in Figs. 23 and 24). This temperature dependence can be modeled using a single coefficient ($\beta $) for each filter that is equivalent to the slope of relative response change with temperature (Equation (7)). The monochromator scans also show that the change was smooth enough that effective wavelength centers and HWHM values for each filter remained unaffected. Using these $\beta $ coefficients (Table 6), the Mastcam-Z radiometric coefficients ($r_{o,k}$) and spectral response ($r_{\lambda ,k}$) derived at −5^∘^ C and +25^∘^ C detector temperatures can be scaled to the various operating temperatures the instrument will experience while on Mars. After landing, we plan to validate the derived $\beta $ coefficients as part of the Mastcam-Z in-flight calibration activities by observing the relative brightness between different color patches on the flight calibration target (Sect. 2.2) as a function of temperature (i.e., time of day). The ratio of the gray ring brightness to the blue color patch brightness should show a correlation with FPA temperature.

7 $$ \begin{aligned} r_{o,k}(T) & = r_{o,k}(T_{\mathit{ref}}) \Bigg( \frac{r_{o,k}(T)}{r_{o,k}(T_{\mathit{ref}})} \Bigg) \\ & = r_{o,k}(T_{\mathit{ref}}) \big( 1+\beta _{k} (T- T_{\mathit{ref}})\big), \end{aligned} $$

where the reference temperature is nominally $T_{\mathit{ref}}=-5^{\circ }~\mathrm{C}$ (Table 6). Although the spectral slope of the $\beta $ coefficients can technically change the filters’ bandpass shapes, this effect is less than 0.5 nm for all filters. The largest effect is for R6, where the cut-off can shift by several nanometers over a large enough temperature change but effectively leaves the cut-on and peak transmission unchanged.

### MTF Testing and Focus

The Modulation Transfer Function (MTF) is an effective means of specifying the resolution of a camera system and measures the ability of an optical system to transfer various levels of detail (i.e. spatial frequencies) from the object being observed to the recorded image. Mastcam-Z has a requirement of having MTF $>0.2$ at Nyquist sampling (47 l.p./mm or .35 l.p./pixel) across all filters and zoom positions for targets distances >2 m. During V&V testing and stand-alone calibration between April 26$^{th}$ and May 4$^{th}$, 2019, a series of well-characterized bar targets were observed at multiple focus and zoom positions in order to characterize the MTF and depth of field of each Mastcam-Z camerahead (see Table 3). In addition to determining the optical performance of the optomechanical assemblies, the images collected during MTF/PSF calibration were also used to determine the performance and repeatability of the stepper motor counts for the Hall Effect sensors used to measure the position of the focus group and two moving zoom groups in the zoom assemblies. Time constraints limited MTF to three zooms settings based on vendor measurements of the optical assembly MTF prior to installation in the camerahead. The points at 34 mm and 100 mm were chosen to cover the range of the expected observation modes, while 61.8 mm was chosen as an in-between value that best approximates the ability to recover the full MTF vs. zoom variation, based on an optical performance model developed by Synopsis, to an accuracy of 3.4% (1-sigma).

MTF testing was separated into four individual data collections meant to asses autofocus performance, distance dependence, temperature dependence, and wavelength dependence of the Mastcam-Z cameras optical performance, using slant edge targets (e.g. MTF Mapper lensgrid, Imatest SVG) and the MTF Mapper software. The first test took a series of images about best focus for a fixed target to compare the derived best focus position to that calculated through the on-board autofocus algorithm. The second test evaluated the change in MTF as a function of distance from the camera. The third verified the ability to maintain best focus images of consistent quality as a function of temperature. Finally, the fourth test measured the wavelength dependence of the best focus position for a fixed target position. In summary, measured MTF values at Nyquist sampling (47 l.p./mm or 0.35 l.p./pix) were always greater >0.26 for all filters and zoom positions, and >0.4 for all zooms at filters L0/R0, confirming that both Mastcam-Z cameraheads have met their frequency resolution requirements (see Table 8).

**Table 8:** Derived MTF values at Nyquist frequency as a function of filter and zoom at the center and edge of the field of view. Values are derived from autofocused or best-focus images of the MTF “lensgrid” target acquired during geometric calibration. The target was at a distance of 2.55 m, 4.68 m, and 7.68 m for the 34 mm, 63 mm, and 100 mm zoom positions, respectively. See Sect. 3.8 for more details

Filter number	Effective center wavelength (nm)	34 mm center^a^–edge^b^	63 mm center–edge	100 mm center–edge
L0	530	0.45–0.47	0.45–0.42	0.41–0.41^d^
L1	800	0.40–0.41	0.39–0.39	0.38–0.37
L2	754	0.32–0.35	0.43–0.42	0.37–0.39
L3	677	0.45–0.45	0.43–0.41	0.40–0.44
L4	605	0.45–0.50	0.44–0.42	0.41–0.38
L5	528	0.48–0.47	0.46–0.46	0.42–0.40
L6^c^	442	0.43–0.45	–	0.43–0.40
R0	530	0.49–0.50	0.47–0.45	0.42–0.47
R1	800	0.42–0.44	0.39–0.40	0.40–0.35
R2	866	0.38–0.40	0.38–0.38	0.35–0.31
R3	910	0.36–0.37	0.36–0.36	0.36–0.32
R4	939	0.36–0.38	0.35–0.36	0.32–0.30
R5	978	0.33–0.36	0.35–0.34	0.30–0.28
R6	1022	0.33–0.34	0.33–0.32	0.26–0.26

^a^The center value is derived from the MTF edge closest to the pixel (824,600)

^b^The edge value is derived from the MTF edge closest to the pixel (1500,600)

^c^Autofocus failed in the L6 filter for the 63 mm zoom position

^d^Typical errors in derived MTF values are ∼.02-.03 and chart orientation can affect these single derived values of the MTF. We find fairly flat MTF response across the field of view (see Fig. 30) and do not find significantly increased or decreased performance in MTF across any given frame

#### MTF Versus Autofocus

On May 5$^{th}$, 2019, a series of images were taken to test the accuracy of the autofocus algorithm of the Mastcam-Z cameraheads. The autofocus algorithm works by acquiring a series of images with large steps of the focus motor position between them, calculating the “entropy” of the image (i.e. the compressed image file size), fitting a second order polynomial to the entropy-position curve, and returning the motor position of highest entropy, which by the definition of entropy contains the most contrast, and thus is in best focus. In this way, a best motor position can be determined at a resolution higher than the focus spacing of the acquired image stack.

**Fig. 25:**
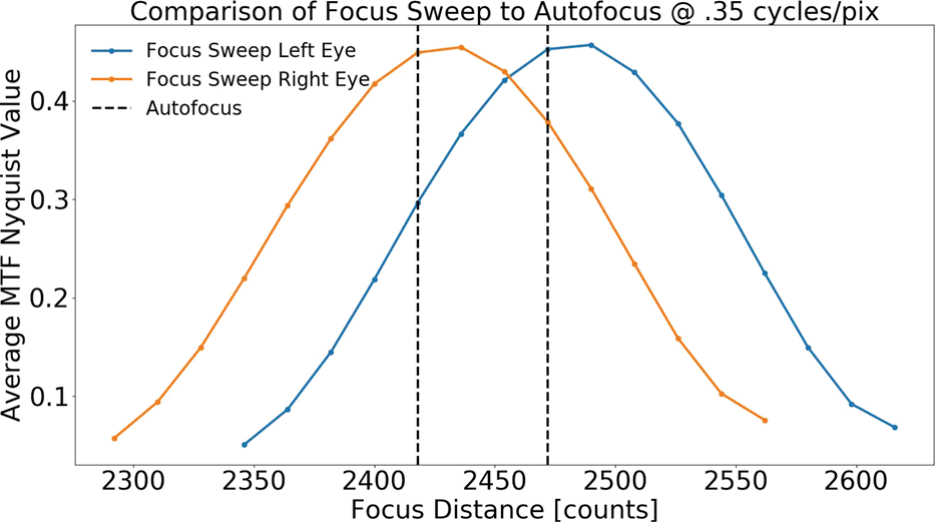
Comparison of the measured best focus position to that determined by the autofocus algorithm. For each camera, a series of images were acquired at varying focus positions, with the average MTF Nyquist value calculated at each position. The highest MTF value corresponds to best focus. The blue and orange lines represent the left and right Mastcam-Zs, respectively. The dotted line plots the best focus position as determined by an autofocus frame on the same target. Good agreement is found between the focus stacks and autofocus algorithm

To test this algorithm, we first commanded an autofocus image of a target at ∼3 m from the camerahead to be acquired in order to determine the best focus position, as determined by the algorithm. Because of the mode in which the camera was operated during calibration, the individual images could not be stored for comparison. However, the positions and derived entropy of the autofocus algorithm were saved. A series of focus images with smaller steps between focus positions were then acquired, generating a focus stack at higher resolution than the autofocus routine. For both the autofocused images and the focus-stack images, the average MTF value at Nyquist was then computed across the FOV and compared. This process was then repeated for each filter. Fig. 25 shows the results of this analysis for the clear L0/R0 filters. In all, we find good agreement between the derived value from the autofocus algorithm and the measured best-focus position from the focus stacks. While there appears to be a preference for slightly shorter focal distances (larger motor count values) than that returned from the autofocus algorithm (Fig. 25), the difference in focus motor step sizes between the focus-stacks and autofocus files makes it inconclusive that this is a general result of the algorithm.

#### MTF Versus Distance

**Fig. 26:**
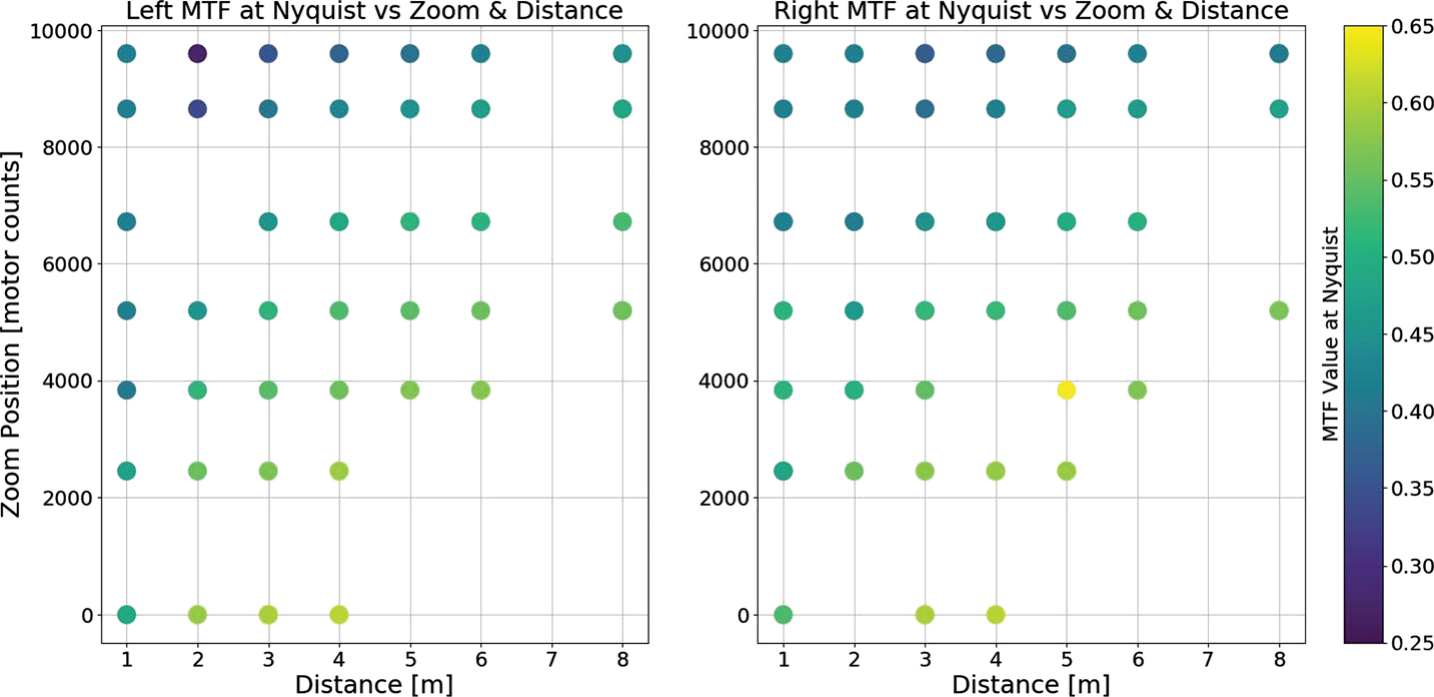
Plot of the derived average MTF values as a function of zoom and distance from data acquired during V&V testing. We find increasing performance in focus with both decreasing focal length (fewer motor counts) and increasing distance

During V&V testing the Imatest SVG target (see Sect. 3.2.2) was imaged at several distances from the camera; with autofocused images acquired at each position. Fig. 26 plots the average derived Nyquist MTF as a function of distance for both the left and right cameras. We find a strong dependence on focus distance vs. zoom position, with shorter distance achievable at wider FOV. Even at the narrowest FOV, however, we find a Nyquist MTF of 0.25 or better for distances as close as 2 m. This improves to an $\mathrm{MTF}_{\mathit{Nyquist}}$ of 0.5 for the widest FOV. From these data, we can fit the expected MTF at Nyquist as a function of focal length and distance from the camera (Fig. 26).

#### MTF Versus Temperature

**Fig. 27:**
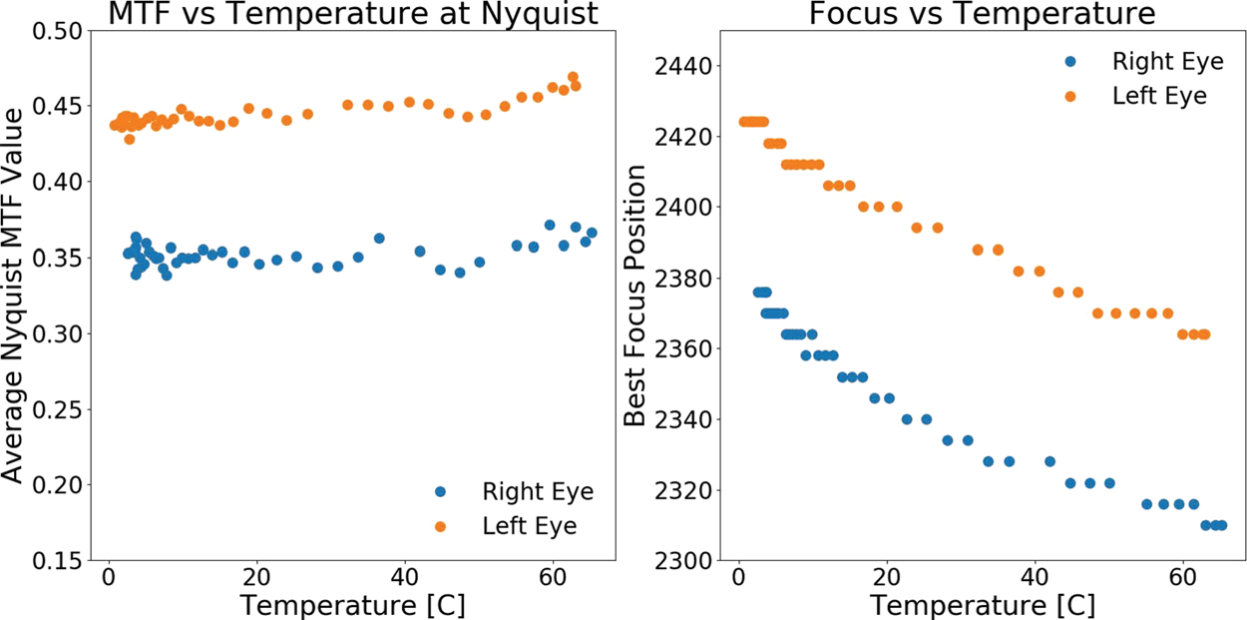
An analysis of measured MTF vs temperature with autofocus. Left: Derived average Nyquist MTF values as a function of temperature from a series of auto-focused frames at 100 mm at a target distance of 3 m. With the appropriate correction in focus motor position, the highest achievable MTF remains constant as a function of temperature. Right: Plot demonstrating the required change in best-focus motor position as a function of temperature

Two sets of temperature dependent MTF data of the SVG target were acquired during V&V testing of the Mastcam-Z cameraheads. The first, taken on a ramp driven from $0^{\circ }$ C to $65^{\circ }$ C, kept a fixed target position and acquired autofocused images throughout the temperature ramp. Fig. 27 plots the derived constrast values at Nyquist (47 l.p./mm or 0.35 l.p./pixel) as a function of temperature as well as the commanded motor focus position for each image. We find an overall constant autofocused MTF Nyquist value across all temperatures, demonstrating that, to within our ability to measure, the best obtainable geometric resolution of the cameras is not a function of temperature. We find a difference in best-focus position of less than 100 motor counts throughout the entire temperature range, further demonstrating the robustness of the geometric performance of the cameras with temperature.

**Fig. 28:**
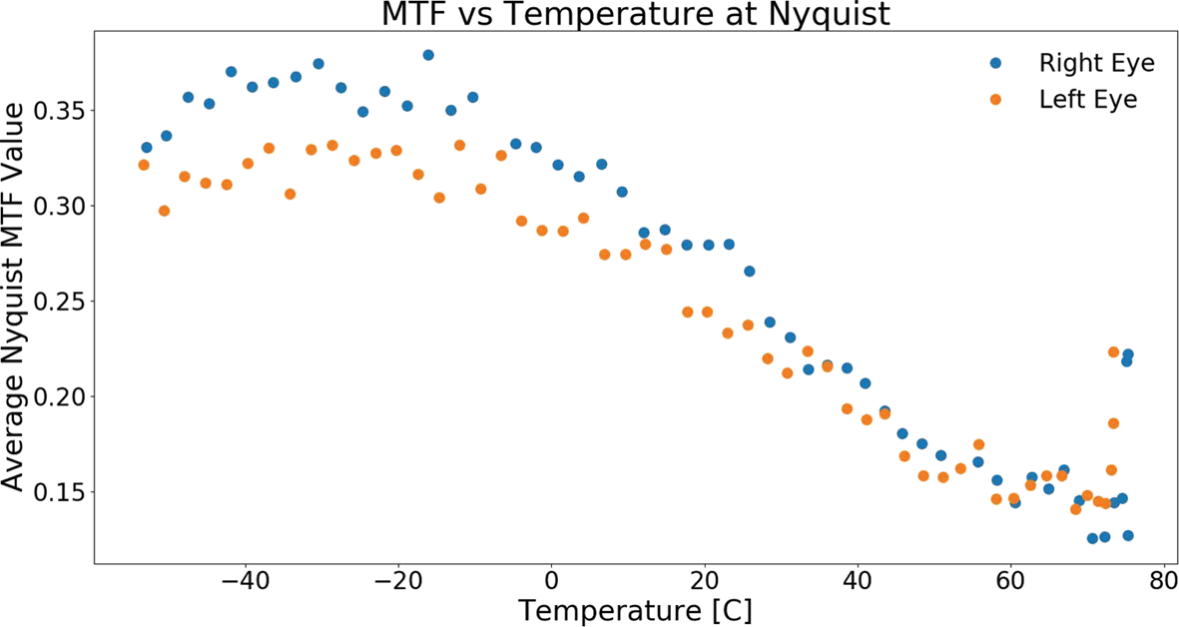
An analysis of measured MTF vs temperature without autofocus. Here, the camera focus position was held fixed as a function of temperature, at 132 and 174 motor count for the left and right eye, respectively, and the degradation in MTF was measured. We find a reduction in the average Nyquist MTF of approximately a factor of two over the temperature range from $-20^{\circ }$ C to $80^{\circ }$ C. At temperatures less than $-20^{ \circ }$ C, we find a roughly constant quality in measured MTF, suggesting smaller changes to the optics at lower temperatures

The second dataset acquired used a fixed focus motor position, through a temperature ramp that ran from $-55^{\circ }$ C to $75^{\circ }$ C, again keeping a fixed target position. The focus motor was locked at the best focus position for the target (determined through autofocus) at $-55^{ \circ }$ C. In this way, we are able to constrain how much the MTF value at a fixed configuration is affected by temperature and the extent to which the focus motor position is correcting this dependence. The derived Nyquist MTF values remain relatively constant for temperature $<-20^{\circ }$ C without any motion of the focus motor (Fig. 28). From $-20^{\circ }$ C to $75^{\circ }$ C we observe a degradation in the derived MTF value from ∼0.3 lp/pix to ∼0.15 lp/pix, that varies roughly linearly with temperature. While it is unfortunate that the more flight-like conditions of low temperature with autofocus were not acquired, the combination of the stable MTF at low temperatures with the highly repeatable autofocus performance at high temperatures suggests that similarly good performance should be expected in flight.

We note that for both these temperature dependent datasets, the comparison between the absolute values of MTF in the left and right Mastcam-Zs are ambiguous owing to the toe-in orientation of the cameras in the thermal vacuum chamber, resulting in each camerahead having a different orientation relative to the chamber window, and the relative position of the target within the FOV of each camera, resulting in MTF values being derived from different sections of the detector. As a result, the data presented in this section represent a minimum MTF that includes effects from the chamber window and are used only to evaluate relative change for each camerahead as a function of temperature.

#### MTF Versus Filter

**Fig. 29:**
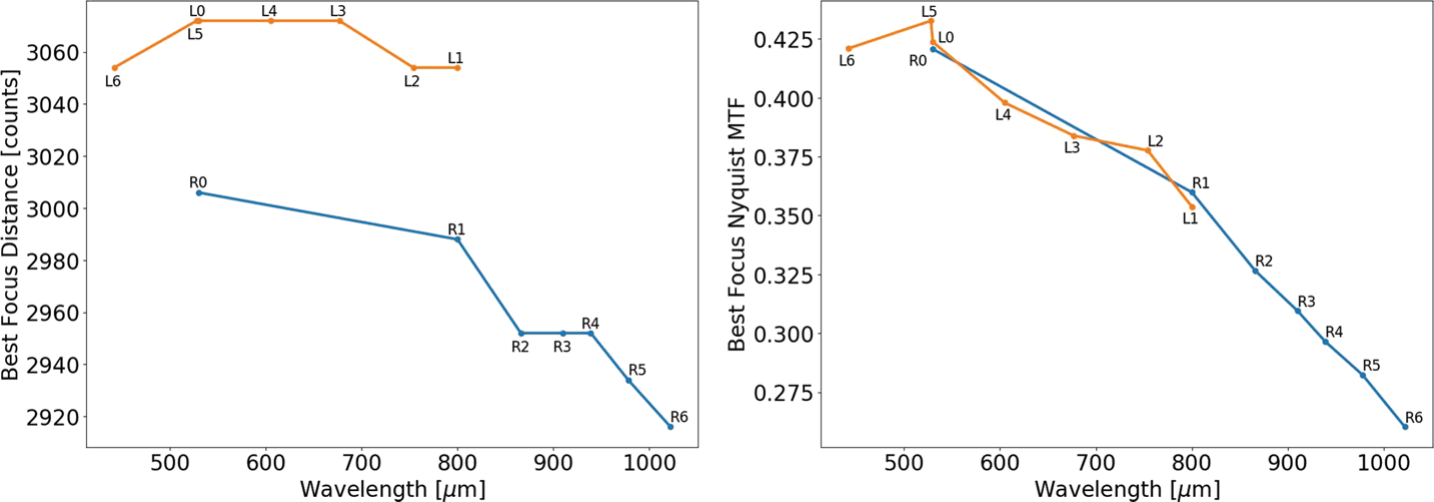
Derived best focus position and MTF at Nyquist as a function of filter for a fixed target position. As in Fig. 25, a focus stack was taken for a fixed target position for each filter and the best-focus image was determined from the maximum measured MTF at Nyquist. Left: Comparison of the derived best focus motor position vs filter. For the visible band filters, we find a roughly constant focus position vs wavelength, resulting from the narrower range of wavelengths, suggesting a single autofocus frame can be acquired for all filters. For the near-IR filters, we find a systematic trend towards fewer motor counts for increasing wavelength. Right: Comparison of the derived best MTF value at Nyquist vs filter. For both cameras, we find a comparable decrease in MTF at Nyquist for increasing wavelength following the expected trend for decreased resolution with increasing wavelength resulting from the fixed effective aperture of the Mastcam-Z cameras

A set of observations were taken to evaluate the color dependence of Mastcam-Z’s MTF performance. For each filter, a focus sweep around the best-focus position (as determined through an autofocus image) were taken using the large “lensgrid” MTF target (SN007) at a distance of 7.68 m from the camera. For each focus sweep, the best focus distance in motor counts and the best MTF at Nyquist were then determined (see Fig. 29). Targets were illuminated with a combination of ambient fluorescent lighting and halogen work lamps.

For the left camera, we find a relatively constant motor position for best focus for each filter, with variations of only ∼ 20 motor counts, or ∼0.5% found between L0 (530 nm) and L1 (800 nm). We observe a general decrease in the best MTF at Nyquist for increasing wavelengths, with a change of ∼15% between the L0 and L1 filters.

For the right camera, as expected, we find a larger trend in the motor position of best focus vs wavelength resulting from the larger wavelength range of the filters in this camerahead. The change in motor position varies by ∼3% from R0 (530 nm) to R6 (1022 nm), comparable to the change necessary for an over 100^∘^ C change in temperature. We measure the same ∼0.5% shift in best focus position between the R0 (530 nm) and R1 (800 nm) filters that was observed with the left camerahead. The average MTF value at Nyquist is also found to drop by ∼40% going from the R0 to R6 filters, and follows the expected trend of decreased resolution with increasing wavelength for fixed aperture optics; though the observed performance appears linear with wavelength, rather than $\propto \lambda ^{-1}$ (Fig. 29). As with the left camera, we observed a comparable ∼15% between the R0 and R1 filters.

#### MTF Surface Maps

**Fig. 30:**
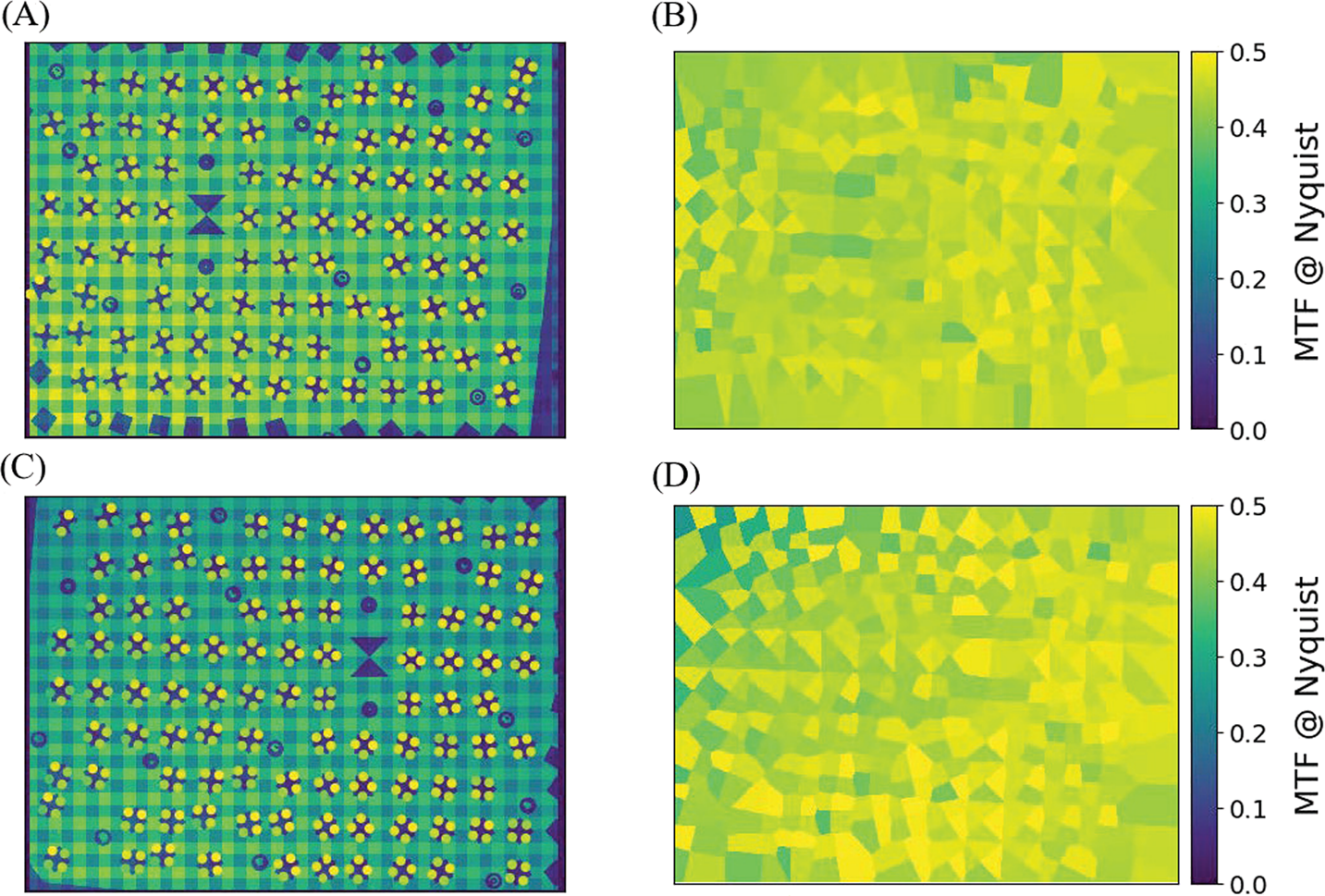
Example of MTF mapper output run on image 471TAMB(L/R)06_0056.png, a 30 ms 8-bit exposure of MTF target SN/007 at 63 mm focal length using L0/R0. (**A**) Edge map showing the identification of each edge in the image and the corresponding MTF Nyquist value. (**B**) The derived MTF Nyquist map for the right eye. Color corresponds the measured MTF constrast at the Nyquist frequency of .35 l.p./pix. (**C**) and (**D**) are identical to panels (A) and (B) except for the left eye. Note that data was sparsely sampled across the FOV, leading to artifacts introduced by the linear interpolation used to fill the frame

Because the “lensgrid” MTF mapper target provides many slant edges across the field of view, it is possible to derive surface maps of MTF values for each filter and zoom position. MTF Mapper is able to automatically identify each slant edge in the image, the number of pixels in the image, and calculate the MTF curve for each edge. From these edges, the calculated MTF values can be interpolated to create surface maps across the field of view. Fig. 30 shows an example of MTF surface maps at Nyquist generated for an 8-bit 30 ms exposure of the “lendsgrid” target (SN007) acquired at 63 mm focal length ($\mathit{zoom}_{mc}=5196$). As can be seen in the surface maps, at 63 mm the image quality does not appreciably vary spatially across the frame. Similarly, the image quality did not appreciable change across the 34 mm FOV. Table 8 contains the derived MTF values at the Nyquist frequency for an edge located at the center and edge of the field of view as a function of both filter and zoom position.

#### Comparison to MSL’s Mastcam

**Fig. 31:**
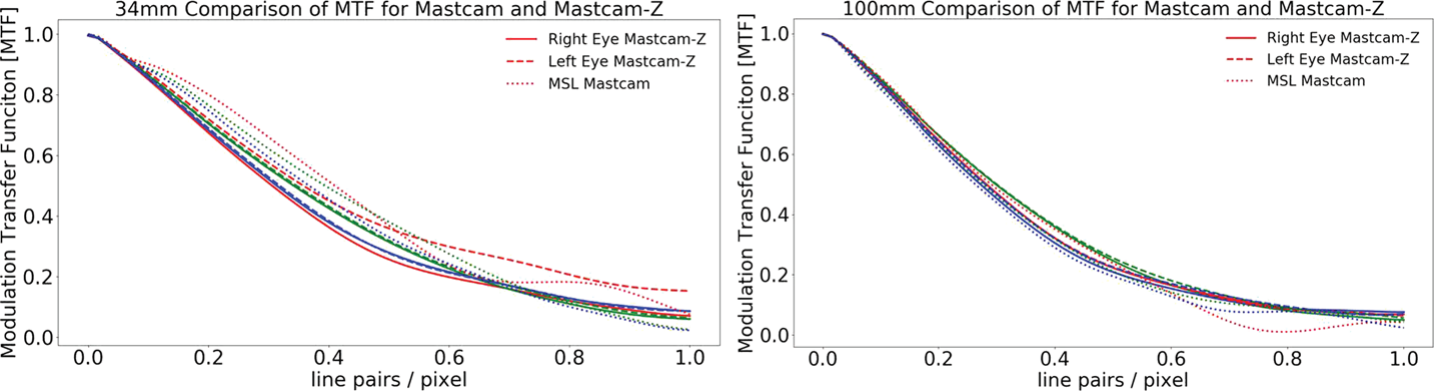
Comparison of the derived MTF between Mastcam and Mastcam-Z. MTF curves derived for 34 mm focal length (left) and 100 mm focal length (right). RGB colors correspond to the individual colors of the Mastcam-Z Bayer pattern. MTF curves for Mastcam are derived from the same images used in Bell et al. (2017). In all, we find good agreement in the MTF curves between Mastcam and Mastcam-Z

Bell et al. (2017) provided a limited analysis of the MTF performance of the Mastcam cameras on MSL—owing largely to strict time constraints that resulted in limited acquisition of MTF data during pre-flight calibration. In their analysis, they used 5 slant edges of a single target, primarily at the center of the FOV, to derive an average MTF curve in RGB from these edges using the Matlab software *sfrmat3.m* (Burns 2000, 2015). MSL Mastcam M100 and M34 were reported to have an MTF at Nyquist (47 l.p./mm or 0.35 l.p./pixel) of 0.04–0.07 and 0.06–0.10. In order to systematically compare these results with Mastcam-Z, we reprocessed the MTF images in Bell et al. (2017) using the MTF Mapper program. We find significantly improved MTF values at Nyquist for the Mastcam cameras (0.4–0.5 for both eyes) which, according to our analysis, have comparable performances to Mastcam-Z (see Fig. 31).

Similar to Mastcam-Z, Mastcam also exhibits a slight difference in the derived Nyquist MTF values between the 100 mm and 34 mm zoom positions, as well as slight color dependencies. In addition, we find a slight increase in performance of the left Mastcam-Z in comparison to the right Mastcam-Z. In general, we find good agreement to the values derived for Mastcam, which is a testament to the excellent build quality of the Mastcam-Z optical assemblies as zoomable optical assemblies typically have lower image quality as compared to fixed-focal length systems.

### Effective Resolution

The Ground Resolution Distance (GRD) is defined as the minimum distance between two point targets that allows them to be detected as separate entities in an image. The GRD is affected by the optical system used to collect light from the target scene, the detector used to form the raw image, and the electronics that process and generate the final digital product. GRD can also be affected by motion of the sensor system during image acquisition. Ground Sample Distance (GSD), or pixel scale, is the footprint projected by one image pixel onto the surface, typically assuming a flat target plane perpendicular to the boresight, and does not taken into account blurring and/or degradation by the optical system, detector, or electronics. By dividing by the distance to the target, GSD and GRD can be expressed in terms of an angular resolution. The angular resolution of an optical system is typically described via the Point Spread Function (PSF), which is defined as the imaging system’s response to a point source. The PSF is the inverse Fourier transform of the Optical Transfer Function (OTF), which specifies the ability of an optical system to transfer details from object to image at different spatial frequencies. The OTF is a complex function, but is real-valued for the case of a radially symmetric PSF (e.g., an ideal circular aperture). The magnitude of the complex OTF is the MTF. Therefore, while MTF expresses optical performance in the frequency domain the PSF describes optical performance in the spatial domain.

The theoretical limit for the fundamental resolution performance of an optical system is determined by diffraction. For an unobstructed circular aperture, the diffraction-limited PSF is described by the Airy disk and has an angular width between its principal maximum and first minimum of $\theta =1.22\frac{\lambda }{D}$, where $\lambda $ is the wavelength of light and $D$ is the diameter of the aperture. Following the Rayleigh Criterion, two point objects can be considered resolved when the first minimum of the PSF generated from one coincides with the principal maximum of the PSF generated by the other. The Rayleigh Diffraction Limit (RDL) describes the GRD for an unobstructed diffraction-limited circular aperture of diameter $D$ with infinitesimal detector elements:

8 $$ \mathit{RDL} = 1.22 \cdot \Big(\frac{\lambda }{D}\Big) \cdot d, $$

where $d$ is the distance from the sensor system to the target.

For sensor systems that are not diffraction-limited, the RDL underestimates the GRD. A more accurate measure of the GRD is to compute it using the full width half maximum (FWHM) of the measured system PSF. While accurately measuring a systems’ two dimensional PSF requires a complex test setup, measuring the MTF is considerably simpler. The inverse Fourier transform of the MTF is the Line Spread Function (LSF), which represents a one-dimensional transect of the PSF along the direction of the edge used to measure the MTF. The FWHM of the LSF is a common standard used to characterize the resolution of remote sensing and medical imaging systems (Perkins and Lees 2016). In terms of the FWHM of the LSF, the GRD is:

9 $$ \mathit{GRD} = \mathit{FWHM}_{\mathit{LSF}} \cdot d. $$

**Fig. 32:**
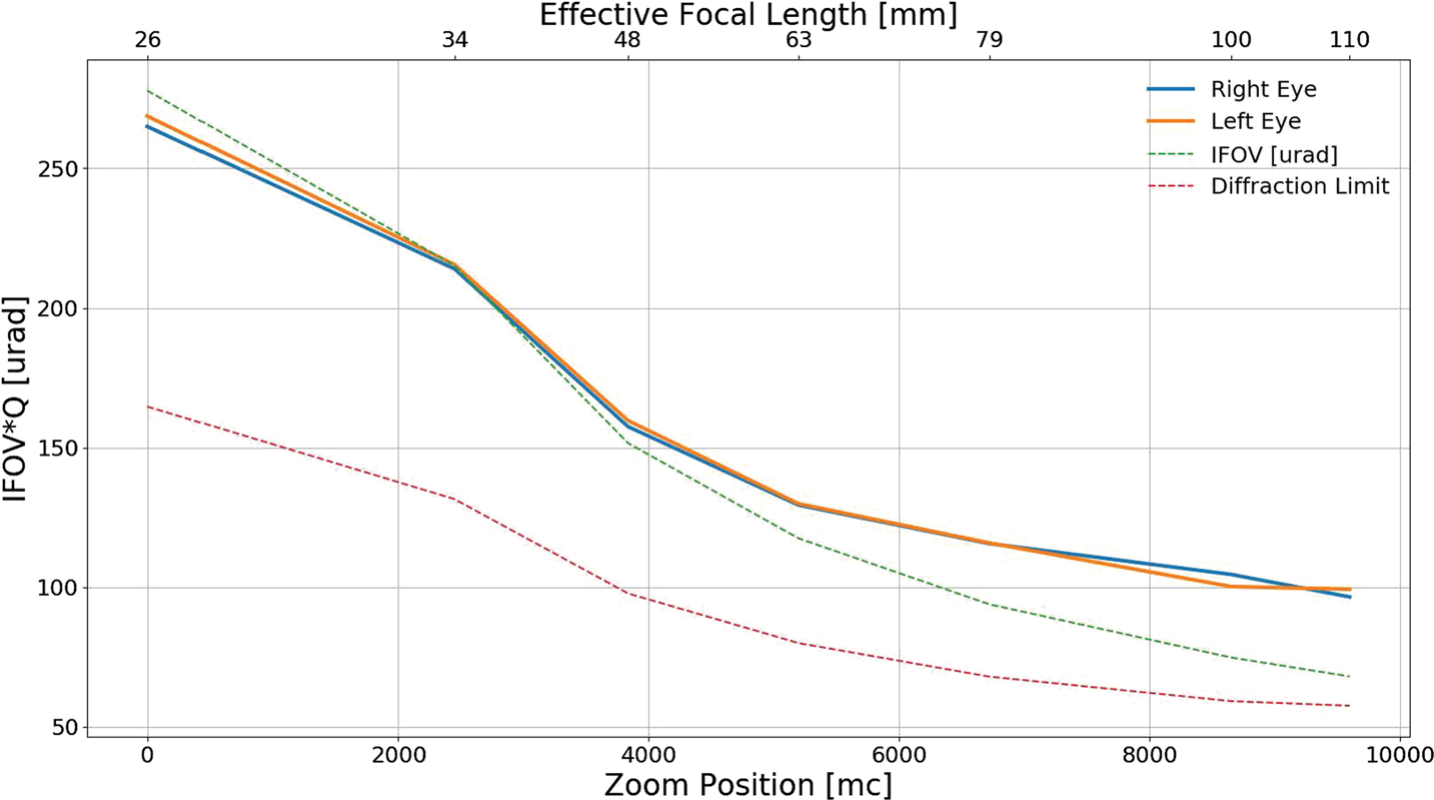
Derived angular resolution of the Mastcam-Z cameras a function of zoom. IFOV is calculated from the pixel pitch and effective focal length. $Q$ is measured as the FWHM of the LSF calculated when determining the MTF for the R0/L0 filters. The green dashed line demonstrates the GSD of a single pixel, while the red dashed line demonstrates the GRD predicted by the diffraction limit. We find that for shorter focal lengths, the pixel pitch begins to dominate the effective resolution of the Mastcam-Z cameras (though still does not meet the criteria that $Q \ll 1$), while for longer focal lengths, system contributions become the limiting factor

A common performance metric used to describe commercial remote sensing systems is the optical quality factor (Q):

10 $$ Q = \mathit{FWHM}_{\mathit{LSF}} \cdot \frac{f}{p}, $$

where $f$ is the effective focal length of the optical system and $p$ is the detector pixel pitch. The instantaneous field of view (IFOV) for each pixel is given by $\mathit{IFOV}=\frac{p}{f}$ (note that $\mathit{GSD}= \mathit{IFOV} \cdot d$). $Q$ is therefore an approximate measure of $\mathit{FWHM}_{\mathit{PSF}}$ in units of pixel IFOV, with $Q=2$ corresponding to Nyquist sampling of the PSF. In cases where $Q \ll 1$, the entire PSF is contained within a single pixel and the GSD becomes an effective measure of the GRD. Fig. 32 plots the derived angular resolution ($\theta _{\mathit{GRD}}=Q \cdot \mathit{IFOV}$) of Mastcam-Z as a function of zoom position. At short focal lengths, we find that the pixel size begins to dominate the contributions to resolution, while at longer focal lengths, system effects dominate the derived resolution. For comparison, we also plot the diffraction limited angular resolution ($\theta _{\mathit{RDL}}=\mathit{RDL}/d$), calculated from the derived Mastcam-Z focal length and f-number values vs zoom, as described in Sect. 3.4.

### Stray and Scattered Light Testing

On May 7$^{th}$, 2019, a series of tests were performed to analyze the stray light performance of the Mastcam-Z cameras during stand-alone calibration. A collimated source was used to over-illuminate the entrance aperture of the Mastcam-Z cameraheads at known illumination geometries (Fig. 10). This stray-light assembly (Sect. 3.2.2) produced a $2''$ collimated beam to approximate direct solar flux at Mars, although with a different color temperature. By measuring the signal in DN/ms of unsaturated frames collected using an ND2 filter and comparing to the signal in DN/ms of images acquired using the Mastcam solar filter on MSL, the collimator is ∼300× dimmer than the Sun at Mars. The assembly allowed for the accurate positioning of the collimator up to ${\pm} 40^{\circ }$ in azimuth and $55^{\circ }$ degrees in elevation, measured with respective to the camera boresight. By measuring the size of the beam in an unsaturated image using an ND2 filter, we are able to determine the divergence angle of the collimator to be ∼7.4 mrad.

**Fig. 33:**
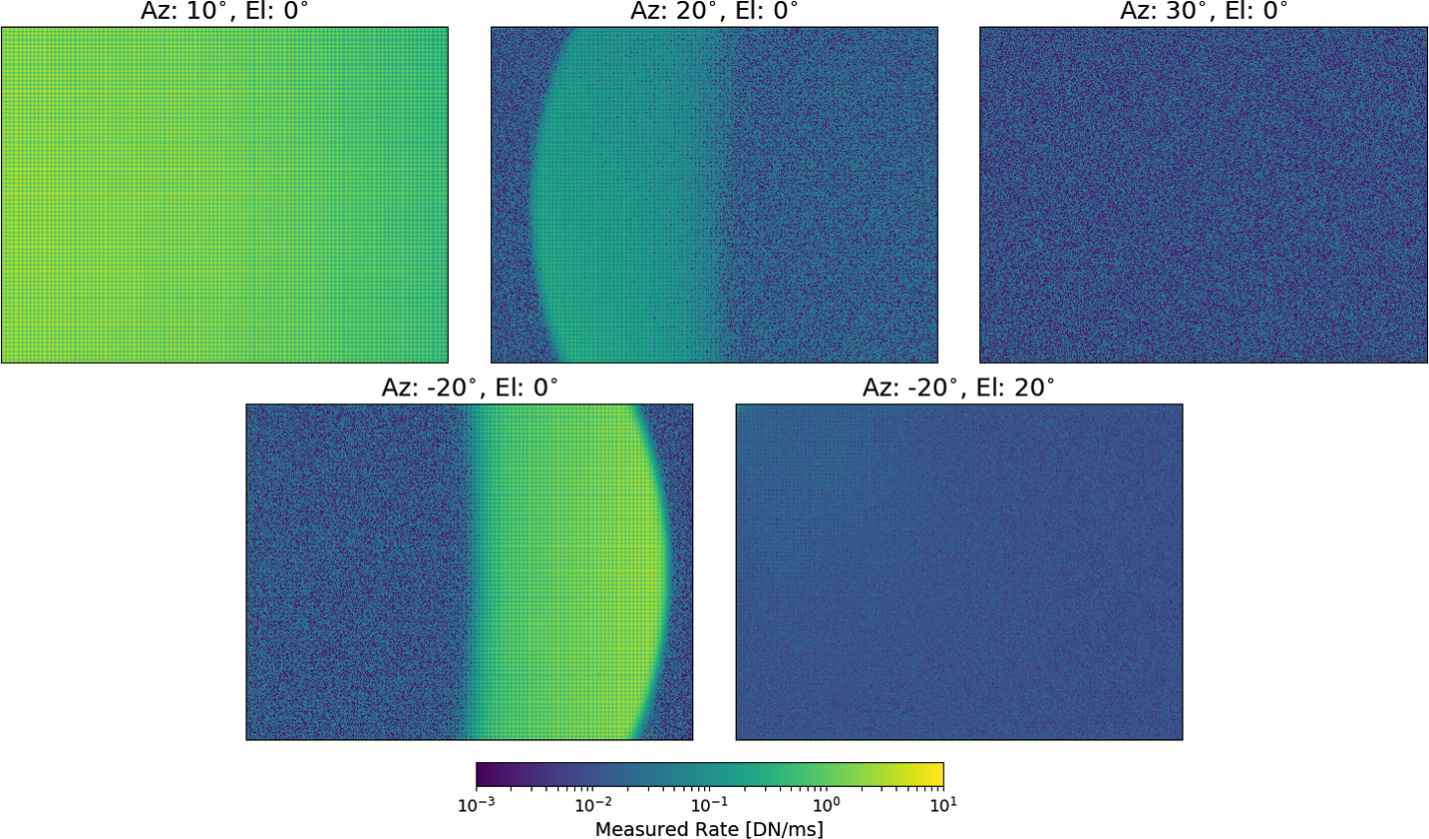
Measurements of the stray light as a function of camera position for the left Mastcam-Z. All frames were acquired at 50 ms exposure times and 100 mm zoom, with the exception of the Az: $-20^{\circ }$,El:$20^{ \circ }$ frame, which was acquired with a 10 s exposure time. Stray light is found to have a contribution to the image immediately outside of the FOV, but drops rapidly with angle. Maximum stray light fluxes of ∼4 $\frac{DN}{ms}$ are observed in the $10^{\circ }$ azimuth frame, but are found to drop to below our ability to measure by $30^{\circ }$ azimuth

Five unique positions were studied on the left Mastcam-Z at a focal length of 100 mm (FOV of $5.1^{\circ } \times 6.8^{\circ }$) and are plotted in Fig. 33. Throughout this section, each instance of an azimuth/elevation pair acts accounts for a single position of the collimator. We find that just outside the field of view, within angles of ${\sim} 20^{\circ }$, stray light can lead to a contribution in the image under bright illumination conditions. At $10^{\circ }$ azimuth and $0^{ \circ }$ elevation, we find a maximum flux of ∼4 $\frac{DN}{ms}$, equivalent to a signal of ${\sim} 1200\, \frac{DN}{ms}$ of solar stray light on Mars. This flux drops to ∼0.3 $\frac{DN}{ms}$ by $20^{\circ }$ azimuth (equivalent to ${\sim} 90\, \frac{DN}{ms}$ on Mars). No observable stray light is present by $30^{\circ }$ azimuth, as the frames were dominated by thermal noise. Interestingly, we find a significantly higher flux at $-20^{\circ }$ azimuth, comparable in magnitude to the $10^{\circ }$ azimuth case, but with a similar vignetting as its symmetric counterpart. This could suggest an asymmetry in the scattering light about the camera boresight. Indeed, a similar structure/intensity in the stray light is also observed on the right Mastcam-Z. Another possibility is that, given the accuracy of the collimator positioning is ${\sim} 5^{ \circ }$, there may be a strong dependence in the angular drop-off in stray light from 10–20^∘^ azimuth. Fluxes at $-20^{\circ }$ azimuth and $20^{\circ }$ elevation were found to be ∼0.05 DN/ms. Based on straylight considerations alone, we recommend a keep-out zone of at least ${\pm} 20^{\circ }$ for bright, solar-like, illumination sources when not observing with L7/R7. Table 9 provides the calculated estimates of the stray light rejection percentage as a function of position, using the measured stray light signal compared to the known signal of the collimator.

**Table 9:** Table of measured stray light rejection for 100 mm zoom position

Position	Total signal [DN]^a^	Integration time [ms]	Thermal signal [DN]^b^	Rejection^c^
Az: 10^∘^, El: 0^∘^	200	50	.6	1.8e-5
Az: 20^∘^, El: 0^∘^	15	50	.6	1.2e-6
Az: 30^∘^, El: 0^∘^	0.7^*d*^	50	.6	<6e-8^*e*^
Az: − 20^∘^, El: 0^∘^	200	50	.6	1.8e-5
Az: − 20^∘^, El: 20^∘^	1450	10000	124	6e-8

^a^Unless otherwise noted, the measured signal is approximated as the respective Mastcam-Z’s estimate maximum value of the bias corrected frame

^b^Thermal signal is approximated from the dark current reported in Sect. 3.3.2

^c^Rejection is calculated as the ratio of the measured signal in [DN/ms] to the measured signal of the collimator: 226500 DN/ms

^d^As no structure was observed in the frame to select a maximum, the frame average is used

^e^As the signal is consistent with the thermal noise, we place a limit of the rejection percentage to be less than that generated by thermal noise alone

**Fig. 34:**
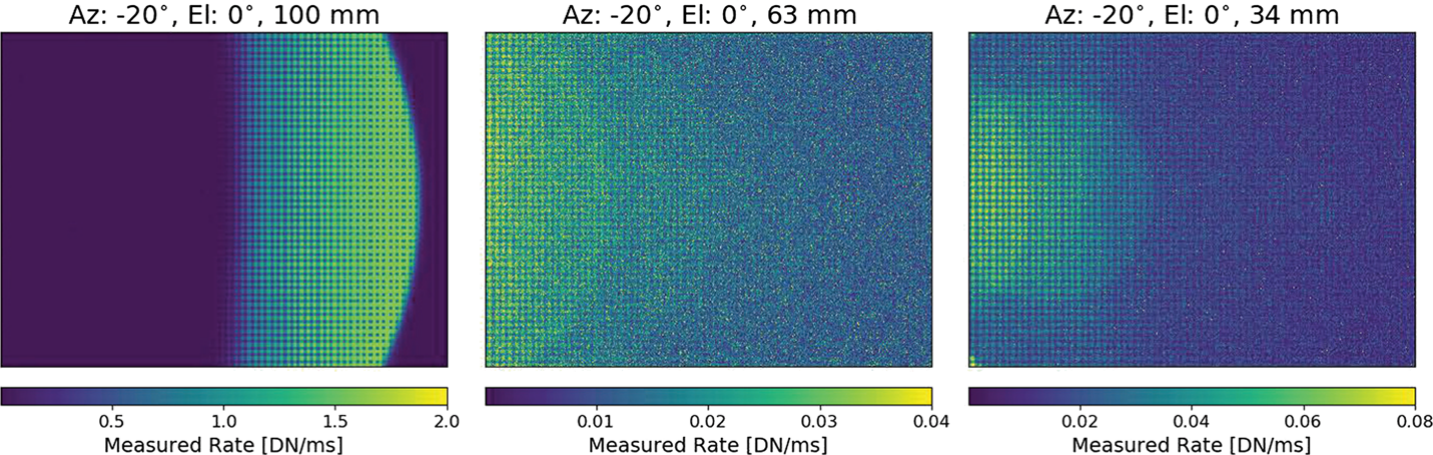
Images of the stray light acquired with the left Mastcam-Z with a collimated beam overfilling the entrance aperture at a $-20^{\circ }$ azimuth and $0^{\circ }$ elevation for three different zoom position. The 100 mm frame is taken with a 1 s integration time, while the 63 mm and 34 mm frames are taken with a 10 s integration time. We find a stronger contribution of the stray light at the 100 mm zoom position, which is largely diffuse, but changes orientation by the 63 mm focal length, and re-focuses to a lens-flare effect at 34 mm

The stray light dependence as a function of focal length was measured at the −20^∘^ azimuth × $0^{\circ }$ elevation position and is plotted in Fig. 34. We find a more uniform structure to the stray light in the 100 mm case, becoming less focused at 63 mm and refocusing to nearly an image of the collimated beam at 34 mm focal length, where the collimated input arrived at an angle $\sim 2\times $ the edge of the field of view (horizontal FOV at 34 mm is $\sim 20^{\circ }$). Furthermore, the orientation of the observed stray light structure was also a function of focal length. While subject to constant illumination conditions, the stray light structure was dominantly focused on the left side of the image at 100 mm, but then moved to the right side of the image as focal length was reduced to 63 mm and then 34 m (see Fig. 34). Not unexpectedly, these frames demonstrate that there is a zoom dependence to the stray light that results from differing positions of the lens elements. These effects will need to be studied in greater detail and at a larger number of input orientations using the Engineering Qualification Model (EQM) and during in-flight calibration on Mars to better constrain the zoom dependence of the stray light expected during surface operations. Regardless, however, stray light rejection is substantial ($>10^{-5}$) for azimuth angles exceeding $20^{\circ }$ from the boresight.

### Geometric Calibration

A geometric understanding of the Mastcam-Z cameraheads is vital for reconstructing the topography, orientation, and shape of terrains observed on Mars. Accurate geometric camera models permit imaging systems to make quantitative measurements of their spatial surroundings. While the data used to generate geometric and radiometric camera models rarely overlap, there is a rich intersection in combining the resulting data products to radiometrically analyze targets identified either at particular locations (e.g., along a bedding plane), under specific illumination and viewing geometries, or simply to combine multi-spectral data from the left and right eyes (e.g., Johnson et al. 2006a,b; Liang et al. 2020; Hayes et al. 2011). Many radiometric data products use the geometric models to linearize (or undistort) images and correct for the slight changes caused by changing focus, filter, or zoom positions. In this context, linearization refers to the geometric pre-processing that minimizes distortion in an image. It is also called rectification. Note that benefits also flow the other way, such as using radiometrically calibrated images, including bias-subtracted and flat-fielding, to increase the fidelity of stereo reconstructions.

While there is a range of 9600 motor-counts in the Mastcam-Z zoom assembly, the geometric calibration is restricted to seven “canonical positions” at effective focal lengths of 26 mm, 34 mm, 48 mm, 63 mm, 79 mm, 100 mm and 110 mm (precise motor-counts are given in Table 10). These are the primary zoom positions for flight operations, and any image taken at another zoom setting will require interpolation to determine camera model parameters. From the perspective of the geometric calibration, each of these zoom positions on the left and right Mastcam-Zs are distinct “cameras” and must be characterized individually. The focus positions and filters also have a non-negligible impact on the camera geometric properties and are generally accounted for by first-order corrections to camera models set to filters L0 and R0 at the reference focus positions of 3 meters. There is some evidence that temperature may also be a factor, and strategies to adapt models made under ambient conditions to conditions on Mars are briefly discussed in Sect. 3.11.4. The outcome of the geometric calibration includes an empirical measurement of the focal lengths and camera center locations for each Mastcam-Z camerahead at each zoom position, as well as second-order parameters including the principal points, distortion coefficients, and external position/orientation of the left and right cameras on the rover body.

**Table 10:** Parameters of the photogrammetric camera model

Nominal focal length [mm]	26 mm	34 mm	48 mm	63 mm	79 mm	100 mm	110 mm
Zoom Motor count [mc]	0000	2448	3834	5196	6720	8652	9600
Num. of Stereo image-pairs	25	25	26	25	32	32	30
Re-projection Error [pix]	0.226	0.271	0.365	0.386	0.404	0.431	0.410
Reference Focus Left^a^ [mc]	−2364	0108	1326	2214	2706	2718	2484
Reference Focus Right^a^ [mc]	−2106	0108	1278	2154	2646	2640	2406
Stereo Baseline [mm]	242.9	243.2	244.0	244.1	244.3	244.2	243.4
Stereo Toe-In angle^b^ [deg]	1.17	1.15	1.15	1.16	1.17	1.17	1.17
Relative Center Offset^c^ [mm]	−0.00	−14.0	−36.8	−46.4	−42.9	−41.8	−33.2
Focal Length Left ($FL_{l}$) [mm]	26.16	34.49	49.22	63.67	79.65	100.1	109.9
Focal Length Right ($FL_{l}$) [mm]	26.16	34.52	49.26	63.69	79.65	100.0	109.9
Horizontal FOV^d^ [deg]	25.6	19.6	13.8	10.7	8.56	6.79	6.18
Vertical FOV^d^ [deg]	19.1	14.6	10.3	7.97	6.38	5.08	4.63
$A_{l}$ focus-param. Left [m × mc]	2090 ± 780	2450 ± 10	2084 ± 32	1888 ± 234	1943 ± 80	1948 ± 21	1951 ± 58
$A_{l}$ focus-param. Right [m × mc]	2268 ± 350	2011 ± 116	2073 ± 79	1965 ± 163	1810 ± 128	1966 ± 22	1968 ± 27
$B_{l}$ focus-param. Left [m]	0.045 ± 0.24	−0.23 ± 0.01	−0.12 ± 0.04	−0.18 ± 0.15	−0.09 ± 0.04	−0.09 ± 0.01	−0.10 ± 0.03
$B_{l}$ focus-param. Right [m]	−0.004 ± 0.12	0.09 ± 0.1	−0.21 ± 0.1	−0.14 ± 0.10	−0.20 ± 0.07	−0.08 ± 0.01	−0.09 ± 0.02
$C_{l}$ focus-param. Left [mc]	−1629 ± 49	844 ± 2	2050 ± 8	2953 ± 28	3349 ± 8	3352 ± 3	3120 ± 6
$C_{l}$ focus-param. Right [mc]	−1338 ± 24	747 ± 26	2036 ± 20	2914 ± 21	3329 ± 13	3287 ± 3	3043 ± 3

^a^The reference focus positions are at the best focus of 3.0 ± 0.2 meters under ambient conditions

^b^The toe-in is taken to be the half-angle between the rays passing through the center pixels of both cameras

^c^The relative offset is the approximate range (+*X*) displacement of the camera center relative to the 26 mm camera placements

^d^These fields of view do not include optical distortion and are nearly equal for the left and right Mastcam-Zs

Geometric calibration typically consists of acquiring multiple images of known dot targets (e.g., Figs. 9 and 36), which are processed into a precise mapping between the center of each target dot in 3D-space and the centroid of its corresponding image on the 2D image plane. A pixel’s ray is nominally defined as a line passing through the camera’s center and its location on the virtual focal plane (although this definition is modified for fish-eye cameras such as the rover’s NavCams and HazCams (Maki et al. 2012)). While many camera model types have been developed over the years (Di and Li 2004)—some even characterizing each pixel individually—the computer-vision and remote-sensing communities have converged on several closely related parametric models that describe the entire image field with less than two dozen calibration parameters. For Mastcam-Z we have chosen to use two mature models that differ mostly in how their coefficient definitions are formulated; the CAHVOR (Di and Li 2004) and OpenCV (OpenCV 2005) models. The coefficients used to describe these models can be translated between one another with only minor loss of fidelity (Di and Li 2004). The CAHVOR coefficients are determined using known target positions in a metrology-dependent photogrammetric derivation. The OpenCV model coefficients, on the other hand, are determined using a pure-photogrammetric derivation that does not require known (i.e., surveyed) target positions (see below). The metrology-dependent CAHVOR method was developed by JPL and uses dot targets with precisely measured positions relative to the cameras to directly calculate the parameters of the CAHVOR camera model (Di and Li 2004; Gennery 2001, 2006; Bell et al. 2017). This method has been applied to every NASA rover-based camera system flown to Mars to-date.

The OpenCV photogrammetric method is an industry standard that uses a pure-photogrammetric approach to solve for the dot-target locations and camera parameters in a single bundle adjustment (Zhang 2000; Bradski 2000; Klopschitz et al. 2017). This approach has been used to geometrically calibrate a variety of planetary orbiters (e.g., Tulyakov et al. 2018). While the standard JPL metrology-dependent method can be more accurate for data-limited scenarios as it utilizes the true target locations, the pure-photogrammetric approach can be equally precise if enough images are used in the model construction (Di and Li 2004). The justification for calibrating Mastcam-Z using both techniques is to (a) derive coefficients for each model to support both high-heritage processing chains used on MER and MSL (CAHVOR) as well as support stereo generation software developed by Joanneum Research and Mastcam-Z Co-Is that use OpenCV and (b) validate the pure-photogrammetric approach for use in future calibration efforts.

Each calibration method starts from the idealized pinhole camera model. The pinhole model is based on the intuitive *camera obscura* in which rays of light enter through a small hole (at the camera center) and shine onto a back wall (the inverted focal-plane). A distortion model is then applied to the focal plane to account for any second-order efforts to account for the real camera’s deviation from the ideal model. The parameters that define the pinhole model and distortion comprise the intrinsic camera model. The camera model’s extrinsic parameters define the location and pointing of the focal-plane relative to a coordinate system defined on the rover. These extrinsic values can be transformed to the rover’s site and global coordinate systems on Mars. Taken together, the intrinsic and external camera model parameters give the precise ray location and direction for each pixel of the camerahead’s detector.

Data appropriate to calibrate Mastcam-Z using both the metrology-dependent and pure-photogrammetric analysis techniques were acquired during calibration. Herein, we summarize preliminary outputs of the OpenCV pure-photogrammetric analysis and compare against the preliminary outputs of the metrology-dependent JPL method. The full and final CAHVOR camera models, which will simultaneously incorporate both zoom and focus dependence and be derived using the metrology-dependent JPL technique, will be the subject of a forthcoming publication (J.N. Maki, personal communication, May 2$^{nd}$, 2020). Similarly, the full and final OpenCV model results, derived using the pure-photogrammetric approach, will be described in a separate publication that will also provide a detailed comparison to the full CAHVOR model results (C. Tate, personal communication, May 2$^{nd}$, 2020). Accordingly, the present discussion is limited to an overview of each model / derivation technique, a summary of the derived geometric camera properties, and a comparison between preliminary results of the CAHVOR and OpenCV model outputs. The full details of each technique and its implementation will be left to these future publications.

#### Camera Model Formulations

**Fig. 35:**
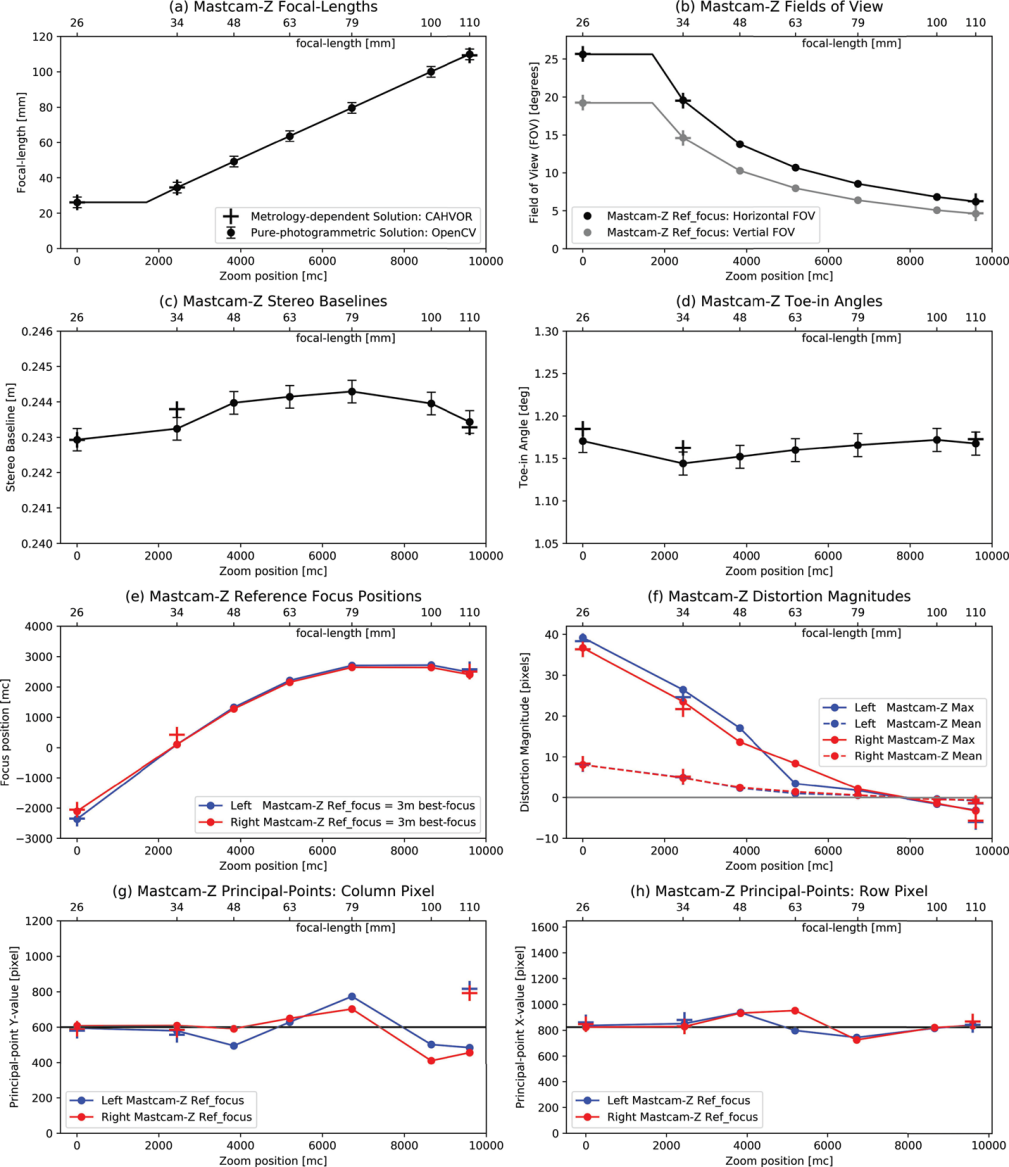
Preliminary geometric calibration results for the Mastcam-Z cameraheads. Results from the metrology-dependent CAHVOR solution are plotted using crosses while results from the pure-photogrammetric solution are shown as dots. Results obtained by the two techniques are consistent to with within error (1$\sigma $). (**a**) Focal lengths versus zoom motor-count, which are nearly identical for the left and right Mastcam-Z cameraheads. (**b**) The fields of view (FOV) in the horizontal and vertical directions. (**c**) Stereo baseline between the left and right cameraheads. (**d**) Toe-in angles, defined as the half-angle between the camerahead boresights, versus zoom motor-count. (**e**) Reference focus positions (set to 3-meters best focus) versus zoom motor-count. Note the slight asymmetry between the left and right focus mechanisms. (**f**) The maximum and mean optical distortions, calculated in a frame reference where there is zero distortion at the image center. (**g**, **h**) The column and row principal-point coordinates respectively. The black horizontal lines represent the center of the detector

The camera model results presented herein describe the intrinsic geometry of the Mastcam-Z optics, as well as the extrinsic position and pointing of each camerahead for the seven canonical zoom positions (Fig. 35 and Table 10). While these values may ultimately differ from the final parameters presented in the follow-on publications discussed above, initial results show that the output of the metrology-dependent derivation of the CAHVOR model and the pure-photogrammetric derivation of the OpenCV model are consistent to within 1-$\sigma $ error (35). For consistency with the follow-on publications, the results presented herein are limited to a general description of the Mastcam-Z geometric properties and stable solutions that are unlikely to change with further analysis (e.g., focal length, distortion magnitude, and stereo baseline / toe-in angle).

**Fig. 36:**
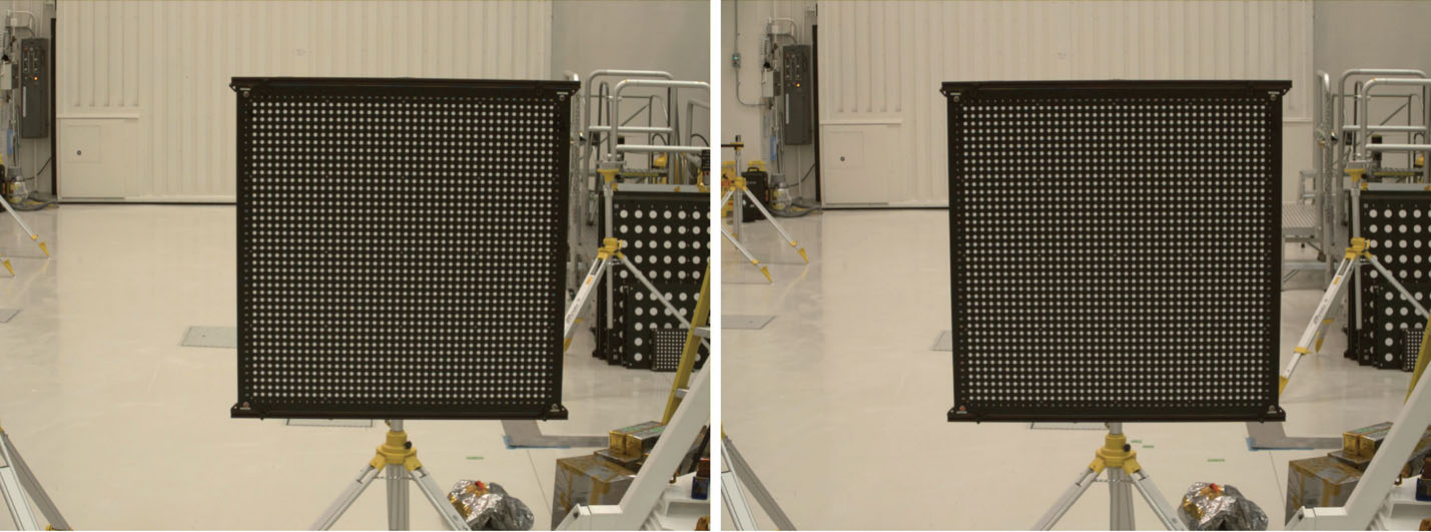
Dot-target stereo pair taken at JPL during ATLO geometric calibration. These white-balanced Mastcam-Z left and right images are at 26 mm focal length with filters L0 and R0, and the dot target is 5.3-meters from cameras. The optical distortion causes a slight warping in the rows and columns of dots, which are in precise straight lines on the physical dot target. See the section for distortion below. Careful observation will also identify the 1.17-degree toe-in angle (Z-axis rotation) and the 0.65-degree roll angle (X-axis rotation) between the left and right images

Pure-photogrammetric post-processing was performed by teams at Joanneum Research and Cornell University using the open-source OpenCV library (OpenCV 2005; Bradski 2000) and its derivatives (Klopschitz et al. 2017). Since the pure-photogrammetric method allows for a more heterogeneous dataset, inputs included all images taken of a geometric dot target (not just the surveyed target placements). The photogrammetric solutions were solved twice: first using the complete stand-alone dataset collected at MSSS and then again for the surveyed dot-target images acquired during ATLO at JPL. The first solution was used to establish a robust initial guess for intrinsic parameters that do not depend on the physical camera location. Extrinsic parameters, which describe the relative location of the cameras, changed between the stand-alone testing at MSSS and ATLO, when the cameraheads were integrated onto the rover. The second solution used the same image set as the CAHVOR analysis, allowing for easier cross-comparison with those results. Note that the intrinsic solution derived from stand-alone testing was found to be very close to the second solution derived from the ATLO dataset, which benefited from the extra constraint of stereo cross-calibration between the two Mastcam-Z cameraheads as the data collection consisted of left-right stereo pairs acquired with the cameras integrated on the rover. Note that the pure-photogrammetric method does not use the surveyed target locations until it translates and rotates the cameras into the rover’s coordinate system, for which only one surveyed dot-target location is sufficient. We refer the reader to the calibration plan and as-run procedures, available in the SOM, for a detailed description of the acquired images and target positions.

For each technique the calibration workflow starts by identifying relevant stereo image pairs, of which the ATLO dataset includes 250. Within this dataset, each of the seven canonical focal lengths has 5-7 focus positions ranging from 1-meter to infinity focus. Although the ATLO dataset is limited to four discrete dot-target placements (at 2.2, 3.3, 5.3, and 42 meters), the stereo overlap and multiple zoom positions place tight constraints on the resulting camera models. The dots in each image are identified on the dot-target grid and centroided to sub-pixel accuracy. The dot locations in the coordinate system of the target (e.g., the dot in column 1 and row 2, where the dots are 1 inch apart) and dot locations in the image coordinates (e.g., the dot was imaged at pixel ij = [300,400]) are then passed to the relevant solver to estimate the optical camera model solution for each zoom position and reference focus position. Since focus position can vary between images, a linear affine transformation is used to transform the dot’s centroided locations between the acquired focus position and the reference focus position. To verify this procedure, dot centroids were determined for a stack of images taken of a fixed target at multiple focus locations. These centroid positions were then transformed to the reference focus position. The difference between the transformed dot positions and the measured positions at the reference focus positions were consistent to within the expected sub-pixel accuracy of the centroiding algorithm itself. This supplies evidence that the focus dependence can be modeled as a linear change of the camera’s intrinsic parameters and has little impact on the extrinsic camera model.

#### OpenCV Formulation

The pure-photogrammetric method used OpenCV’s solver stereoCalibrate (OpenCV 2005), which is accurate to a single scale term and requires six spatial terms for camera placement and pointing. This necessitates another intermediate adjustment to make the photogrammetric camera solutions at various zoom and focus positions homogeneous across the whole parameter space and is achieved by exploiting the fact that several stationary dot-target placements were imaged in many zoom and focus positions, allowing for a linear correction to both scale and the relative offsets of the camera centers. The left 26-mm camera position is taken as the fixed point to which the other models are corrected because of its low re-projection error (wider-FOV cameras generally fare better in photogrammetric solutions). The other camera models are rotated, scaled, and offset for self-consistency across the entire zoom range. Note that, before scaling, the differences between the intrinsic solutions for all focal lengths (34 mm–110 mm) was less than $0.7\%$ in magnitude. The relative offsets are given in Table 10 and interpreted as the movement of the camera center with zoom. Note that the camera centers move back by −46 mm between 26 mm and 63 mm zoom positions then moved forward −33 mm between 63 mm and 110 mm zoom positions. Based on this observation and the focus position to distance curves, it appears that the optical assemblies have an inflection point around 63 mm that marks an extreme in the focus dependence. This is supported by the focal length dependence on focus, which is an order of magnitude less at 63 mm than at either 26 mm or 110 mm.

The intrinsic photogrammetric camera model is defined by the pin-hole camera calibration matrix $\mathbf{K}$ and a set of distortion parameters $\delta _{l}= (k_{1}, k_{2}, p_{1}, p_{2}, k_{3})$. The relationship between the pixel coordinates $(i,j)$ and a point in camera-centered coordinates $\mathbf{x}_{c}$ or the rover’s coordinates $\mathbf{p}$ is given as,

11 $$ \begin{aligned} s \left [ \textstyle\begin{array}{c} i \\ j \\ 1 \end{array}\displaystyle \right ] & = \left [ \textstyle\begin{array}{c@{\quad }c@{\quad }c} f_{x} & a & c_{x} \\ 0 & f_{y} & c_{y} \\ 0 & 0 & 1 \end{array}\displaystyle \right ] \left [ \textstyle\begin{array}{c} x_{c} \\ y_{c} \\ z_{c} \end{array}\displaystyle \right ] \\ s \mathbf{u}_{ij} & = \,\, \mathbf{K} \,\, \mathbf{x}_{c} \, = \,\, \mathbf{K} \,\, \mathbf{R} \, ( \mathbf{p} - \mathbf{c} ), \end{aligned} $$

where $s$ is the scale parameter, $\mathbf{R}$ is the rotation matrix, and $\mathbf{c}$ is the position vector for the camera’s center in the rover’s coordinate system. We note that throughout this paper the use of bold lowercase letters for vectors, bold uppercase letters for matrices, and nonbold letters for scalars.

The distortion correction is governed by Equation (12) below, where $\mathbf{x}_{c} \xrightarrow{} \mathbf{x''}_{c} $. This distortion model was advanced by Duane C. Brown in the 1960s (Brown 1968) and has become a standard for many photogrammetric libraries such as OpenCV (OpenCV 2005). Three radial distortion parameters ($k_{1}$, $k_{2}$ $k_{3}$) capture for the distortion radiating from the principal point ($c_{x}$, $c_{y}$), and two tangential parameters ($p_{1}$, $p_{2}$) account for the cross-field shear due to subtle misalignments in the camera lenses. The parameters $k_{3}$, $p_{1}$, and $p_{2}$ are sometimes set to zero for the Mastcam-Zs because they were found to be insignificant (to within error) and their absence provides a more direct comparison with the CAHVOR distortion model, as discussed below. The distortion terms in $\delta _{l}$ are assumed to depend only on zoom $l$.

12 $$ \begin{aligned} \mathbf{x''}_{c} & = [ x'', y'', 1 ]^{T} \\ \mathbf{x}_{c} & = [ x_{c}, y_{c}, z_{c} ]^{T} \\ \delta _{l} & = [ k_{1}, k_{2}, p_{1}, p_{2}, k_{3} ] \\ x' & = x_{c}/z_{c} \\ y' & = y_{c}/z_{c}. \\ r^{2} & = x^{\prime \,2} + x^{\prime \,2} \\ x'' & = x' (1+k_{1}r^{2}+k_{2}r^{4}+k_{3}r^{6}) + 2p_{1}x'y' + p_{2}(r^{2}+2x^{\prime \,2}) \\ y'' & = y' (1+k_{1}r^{2}+k_{2}r^{4}+k_{3}r^{6}) + 2p_{1}(r^{2}+2y^{\prime \,2}) + 2p_{2}x'y' \end{aligned} $$

Since this camera model is strictly valid for only one filter and focus position, a correction is required to transform the camera model for any filter and focus position into a reference camera state. This is accomplished by multiplying the camera model by an affine matrix. Pixel coordinates in the image frame are affine transformed to the reference filter $k_{\mathit{ref}}$, which is always $k_{\mathit{ref}}=0$ for each camera (L0/R0), and to the reference focus position $f_{\mathit{ref}}$ which depends on zoom ($l$). The reference filter and focus positions are nominally positions at which the complete camera model was solved for each focal length.

For a given focal length, filter and focus position, the pixel location $\mathbf{u}_{ij}$ and its ray or position in camera-centric space $\mathbf{x}_{c}$ is related by,

13 $$ {\mathbf{u}}_{ij,\mathit{lkf}} = \left [ \textstyle\begin{array}{c} i \\ j \\ 1 \end{array}\displaystyle \right ]_{\mathit{lkf}} $$

14 $$ {\mathbf{u}}_{ij,l} = {\mathbf{u}}_{ij,l,k=0,f=f_{l,\mathit{ref}}} = \boldsymbol{K}_{l} \,\, \mathbf{x''}_{c} $$

where $\boldsymbol{K}_{l}$ is the camera’s intrinsic matrix and $\mathbf{x''}_{c}$ is the un-distorted point using the parameters $\delta _{l}$.

15 $$ \mathbf{K}_{\mathit{lkf}} = \left [ \textstyle\begin{array}{c@{\quad }c@{\quad }c} f_{x} & a & c_{x} \\ 0 & f_{y} & c_{y} \\ 0 & 0 & 1 \end{array}\displaystyle \right ] _{\mathit{lkf}} $$

16 $$ {\boldsymbol{u}}_{\mathit{ijl}} = {\boldsymbol{u}}_{\mathit{ijl},k=0,f=f_{l,\mathit{ref}}} \cong \boldsymbol{M}_{\mathit{lkf}} {\boldsymbol{ u}}_{\mathit{ijlkf}} $$

where the affine matrix for filter and focus can be combined into one transformation,

17 $$ \boldsymbol{M}_{\mathit{lkf}} \equiv \boldsymbol{M}_{l,k\to 0, f \to f_{\mathit{ref}}} = \left [ \textstyle\begin{array}{c@{\quad }c@{\quad }c} c_{00} & c_{01} & c_{02} \\ c_{10} & c_{11} & c_{12} \\ 0 & 0 & 1 \end{array}\displaystyle \right ]_{\mathit{lkf}}. $$

The pixel locations are then described by the modified camera model,

18 $$ {\boldsymbol{u}}_{ij,\mathit{lkf}} = \boldsymbol{M}^{-1}_{\mathit{lkf}} \quad \boldsymbol{K}_{l} \quad \mathbf{x''}_{c} . $$

During photogrammetric calibration, the filter and focus transformations were applied in reverse for each calibration point or dot pixel location in the image frame. After this transformation, the dots’ coordinates were passed to the standard OpenCV function that estimates the intrinsic camera model ($\mathbf{K}_{l}$ and $\delta _{l}$). In the geometric reconstruction of the 3D scene, the camera’s intrinsic matrix can be modified by the affine transformation to account for the second-order changes introduced by changing filter and/or focus position. Although an affine transformation has six degrees of freedom (scale, offset, and shear in the x and y directions), the shear terms are insignificant ($c_{01},c_{10}\simeq 0$) and do not introduce any measurable rotation into the modified intrinsic matrix $\boldsymbol{M}^{-1}_{\mathit{lkf}} \boldsymbol{K}_{l}$.

#### CAHVOR Formulation

The CAHVOR camera model uses six vectors ($\mathbf{c}$—camera center vector, $\mathbf{a}$—camera axis unit vector, $\mathbf{H}$—horizontal information vector, $\mathbf{V}$—vertical information vector, $\mathbf{O}$—optical axis, and $\mathbf{R}$—radial lens distortion coefficients) to define the pixel coordinates $(i,j)$ where the point $\boldsymbol{p}$ intersects the focal plane and the camera center point $\mathbf{c}$ (Gennery 2001; Di and Li 2004; Gennery 2006; Bell et al. 2017). A concise summary of the CAHVOR formulation is,

19 $$ \begin{aligned} i & = \frac{ (\mathbf{p}-\mathbf{c}) \cdot \mathbf{h} }{(\mathbf{p}-\mathbf{c}) \cdot \mathbf{a} } \\ j & = \frac{ (\mathbf{p}-\mathbf{c}) \cdot \mathbf{v} }{(\mathbf{p}-\mathbf{c}) \cdot \mathbf{a} } \end{aligned} $$

or equivalently (for direct comparison with Equation 11),

20 $$ \text{s} \left [ \textstyle\begin{array}{c} i \\ j \\ 1 \end{array}\displaystyle \right ] = \left [ \textstyle\begin{array}{c} \mathbf{h}^{T} \\ \mathbf{v}^{T} \\ \mathbf{a}^{T} \end{array}\displaystyle \right ] ( \mathbf{p} - \mathbf{c} ). $$

The following conversions are used for direct comparison with the horizontal and vertical focal lengths and principal points of OpenCV’s formulation,

21 $$ \begin{aligned} f_{x} & = \mathbf{a} \cdot \mathbf{h} \\ f_{y} & = \mathbf{a} \cdot \mathbf{v} \\ p_{x} & = || \mathbf{a} \times \mathbf{h} || \\ p_{y} & = || \mathbf{a} \times \mathbf{v} ||. \end{aligned} $$

The distortion strategy for CAHVOR is similar to the OpenCV model in that it uses five parameters to modify point $\mathbf{p} \xrightarrow{} \mathbf{p'}$ to account for distortion. These parameters, however, are not identical. Only two of the five parameters are equivalent (e.i. $k_{1}$ and $k_{2}$) when all others are set to zero. The CAHVOR model uses the “optical axis” $\mathbf{O}$ to rotate or offset the center of radial distortion in the focal plane relative to the detector normal axis $\mathbf{a}$.

22 $$ \begin{aligned} \mathbf{p'} & = \mathbf{p} + \mu \mathbf{\lambda } \\ \zeta & = (\mathbf{p}-\mathbf{c}) \cdot \mathbf{o} \\ \boldsymbol{\lambda } & = (\mathbf{p}-\mathbf{c}) - \zeta \mathbf{o} \\ \tau & = ( \boldsymbol{\lambda } \cdot \boldsymbol{\lambda } ) / \zeta ^{2} \\ \mu & = k_{0} + k_{1} \tau + k_{2} \tau ^{2} \\ \mathbf{r} & = [k_{0},k_{1},k_{2}]^{T}. \end{aligned} $$

As is the case with OpenCV’s distortion, these equations must effectively be solved in reverse to un-distort, or linearize (i.e., rectify), an image starting from distorted pixels in the image and estimating the pixel locations without distortion. Although this poses a mathematical challenge, many software packages are available to accurately evaluate this conversion and move between distorted and un-distorted space.

##### Focal Length and FOV Versus Zoom

The above camera models are solved for each zoom position using a least-square minimization of dot target locations in the geometric dataset. The OpenCV pure-photogrammetric solutions for focal length and field-of-view (FOV) are given in Fig. 35(a) and 35(b). The focal lengths for the left and right Mastcam-Z’s differ by less than 0.1% at a given zoom position, and vary linearly with zoom motor-count after about 1800 motor-counts. The discontinuity between 26 mm and 34 mm is to protect the launch-lock position at 26 mm. In actuality, the transition is more gradual than depicted in Fig. 35(a). The mapping between focal length and zoom position is given as

23 $$ FL(l) = \textstyle\begin{cases} 0.01054\times l +8.77 & \text{if } l>2000 \\ 26.16 & \text{if } l< 600 \\ \end{cases} $$

where the focal length $FL$ is in millimeters and $l$ is the zoom motor-count. The range between $600 \leq l \leq 2000$ is non-linear and less constrained. The zoom Hall sensors have an indeterminacy between $0< l<200$, which should be avoided in-flight. As a result, 26 mm images can be taken at either 0 mc or between 200 mc and 600 mc with only minor changes of principal-point that can be readily corrected. More importantly, the focal length does not measurably change for $l \leq 600$.

The FOVs in the horizontal and vertical directions (Fig. 35(b)) are useful quantities in planning image sequences. The reported FOVs do not include optical distortion, which can increase the FOV by about 2% at 26 mm.

##### Extrinsic Parameters Versus Zoom

Fig. 35(c,d) shows the zoom dependence of the stereo baseline and toe-in angles, respectively. The stereo-baseline between the left and right cameraheads varies by less than 2 mm over the entire zoom range. Toe-in, defined as the half angle between the rays passing through the center pixels of the left and right cameraheads, is similarly stable across zoom with a variance of less than $0.2^{\circ }$. There is also a relative roll between the left and right Mastcam-Zs of $0.65^{\circ }$ around the boresight direction (X-axis in the camerahead reference frame). These semi-constant parameters show that the zoom mechanism is exceptionally smooth across its entire range.

##### Focus Versus Zoom

For a given zoom position, the Mastcam-Z geometric calibration handles focus dependence by transforming camera model solutions to a reference focus position via an affine transformation (Table 10 and Fig. 35(e)). For each camerahead and zoom position, the reference focus position is determined as the best-focus for a planar target at a 3-meter distance under ambient conditions. For dot-targets, the transformation estimates the dot locations that the cameras would have seen if the focus position was at its reference value. The affine coefficients are derived from an image set of stationary dot targets acquired at 16 pre-defined focus positions ranging from approximately 1-meter to infinity for each of the seven canonical zoom positions, as well as at 68 zoom positions for a constant 3 meter best focus. The selected range of focus positions effectively spans the operational range of each Mastcam-Z camerahead. When transformed to the reference focus position using the derived affine transformations, the dot target centroid locations measured at the test focus positions matched those measured from images acquired at the reference focus position to within sub-pixel accuracy (i.e., the expected accuracy of the centroid algorithm itself).

The affine transformations adjust for slight changes in focal length and principal point introduced by motions of the focus mechanism. Preliminary analysis suggests that transforming each image to a reference focus position does not jeopardize the precision of the photogrammetric solutions. While higher-order errors may arise from a slight change of camera center location for changing focus position, this translation is estimated to be $<5$ mm across the full range of the focus mechanism. Affine transformations can also relate camera models for each filter to the reference L0 and R0 camera models. Filter-to-filter affine correction matrices will be updated and improved in-flight using multi-spectral sequences of natural targets on Mars, where small mis-registrations between frames will be detectable as “fringing” in color image products.

The relation between distance and best-focus position was characterized from the stand-alone data set and validated in ATLO. Having an estimate for this relation is important for efficiently choosing autofocus ranges during flight operations. Assuming that the image distance ($ID$) of the thin lens formula ($\frac{1}{FL} = \frac{1}{ID}+\frac{1}{OD}$, where $FL$ is the focal length and $OD$ is the object distance) is linear with focus motor-count ($ID\approx A+B f$), the relation between best-focus distance ($FD_{l}$, as a proxy for $OD$) and $f$ has the functional form:

24 $$ FD_{l}(f) = \frac{A_{l} + B_{l} f}{ C_{l}-f }, $$

where the ambient values for $A_{l}$, $B_{l}$, and $C_{l}$ are given in Table 10 for each zoom position. Note that Equation (24) is primarily used as a tactical tool for determining autofocus parameters in-flight, and not for deriving any photogrammetric properties of the cameraheads. As a result, the distance $FD_{l}$ is measured in meters from the shades on the front of the lens assemblies and not from the camera center points. Note that the focus-distance relation of the cameraheads is also sensitive to filter (i.e., wavelength) and, to a greater extent, camerahead temperature (see Figs. 29–27). Over the range of temperatures of tested during the MSSS TVAC ramps ($-5^{\circ }$ C and $+50^{\circ }$ C), the 34 mm and 100 mm zoom best-focus positions changed in a linear fashion by −60 and −20 focus motor-counts, respectively, for an MTF target at 3 meters. This translates to an error of 0.25 m or $\sim 10\%$ distance error for 34 mm at 3 meters and increases for more distant targets. Although this temperature dependence can be partially removed, this level of variability will not affect tactical selection of autofocus parameters. Note that first-order corrections for temperature and filter primarily modify the $C_{l}$ parameter, leaving $A_{l}$ and $B_{l}$ mostly unchanged.

##### Optical Distortion Versus Zoom

**Fig. 37:**
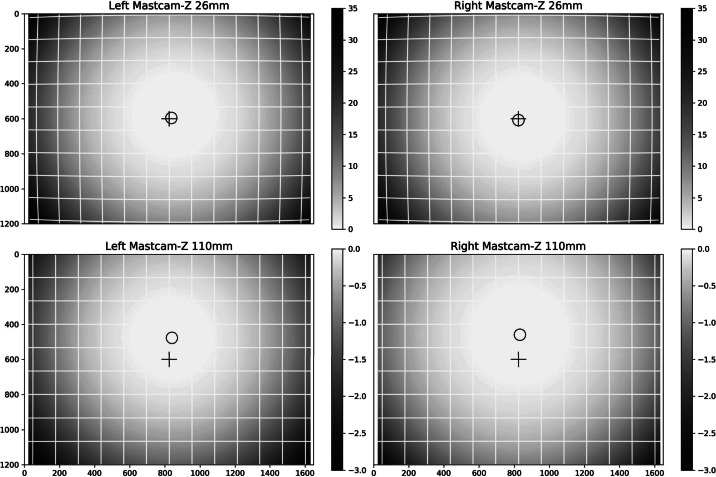
Mastcam-Z distortion maps for 26 mm and 110 mm. Grey-scale color bar depicts distortion magnitude in pixels (sum of $x$ and $y$ distortions in quadrature). The plus (+) symbols are the detector centers, while the circles (∘) are the principal-points. Note that the distortion is nearly zero in the center of the frame and grows to its maximum value in the corners

The maximum optical distortions (Figs. 37 and 35(f)) are always at the edges of the frames and primarily in the lower left of the array. Positive values of distortion displace pixels away from the frame’s center (i.e., barrel distortion), while negative values displace pixels towards the center (i.e., pincushion distortion). Notice a cross-over from barrel distortion to pincushion distortion occurs between the 79 mm and 100 mm zoom positions in Fig. 35(f).

The distortion is near zero at the images principal point and remains below 30% of its maximum value across the center half of the image’s area. Due to this linearity near the image center, the affine parameters were derived using data from this region. Observed distortion magnitudes agree with the pre-build optical modeling. Note, however, that minimizing distortion at the image center is a choice made during analysis and results in reported focal lengths representing the effective focal length at the image center. Mathematically, the lowest order radial distortion coefficient is completely correlated with the focal length. In some cases the focal length and distortion coefficients are traded off in post-processing to “balance” the distortion pattern so it is zero at an intermediate radius and has equal positive and negative extreme values. For consistency with the MSL Mastcam Calibration (see Bell et al. (2017)), however, we have chosen not to do this. Accordingly, it is relevant to note that the extrema of the reported Mastcam-Z distortion magnitudes may seem artificially high when compared to optical systems whose distortion has been “balanced”.

##### Principal-Point Versus Zoom

The principal points (Fig. 35(g,h)) can be interpreted as the location (i.e., pixel) on the detector array that intersect each camerahead’s optical axis. The observed column and row values for the principal point vary by ±200 pixels across the entirety of the zoom range. Note that the disagreement between the OpenCV and CAHVOR principal points at 110 mm seen in Fig. 35(h) are not significant, as the CAHVOR solution uses the distortion term for the optical axis $\mathbf{O}$, as opposed to a translation of the principal point, to ultimately capture a very similar distortion field as the OpenCV solution.

#### Temperature Dependence of Geometric Camera Models

The camera models were derived using data taken under ambient conditions in a laboratory and their application in Mars’ thermal environment will introduce several unconstrained uncertainties. ATLO stare tests in JPL’s TVAC chamber showed that the Mastcam-Z cameraheads’ absolute pointing can shift by ${\sim} 1^{\circ }$ over a change of $30^{\circ }$ C, although the relative position between the two cameraheads remained consistent to within error. This suggests that the observed motion is due to the mast assembly en masse, and not due to thermal warping of the Mastcam-Z mounting bracket or other factors that would measurably change the positioning of the cameraheads relative to each other. Regardless, the extrinsic parameters of the Mastcam-Z camera models will be verified and, if necessary, updated during in-flight calibration (see Sect. 5). During stand-alone testing, the thermal impact on Mastcam-Z’s intrinsic parameters was estimated from photogrammetric calibrations at 48 mm focal length taken through the TVAC window at $-5^{\circ }$ C under vacuum and $+25^{\circ }$ C at ambient pressure. Results showed a 2.5% decrease in focal length between $-5^{\circ }$ C and ambient. While it is difficult to disentangle changes in the intrinsic optical behavior of the cameras from effects caused by the TVAC window or other test conditions, it is likely that the ambient camera models will need to be empirically updated in-flight. This is best accomplished by comparison to coincident NavCam measurements, whose optics are less susceptible to thermal effects (Maki et al. 2012, 2020).

### Geoboard Imaging

**Fig. 38:**
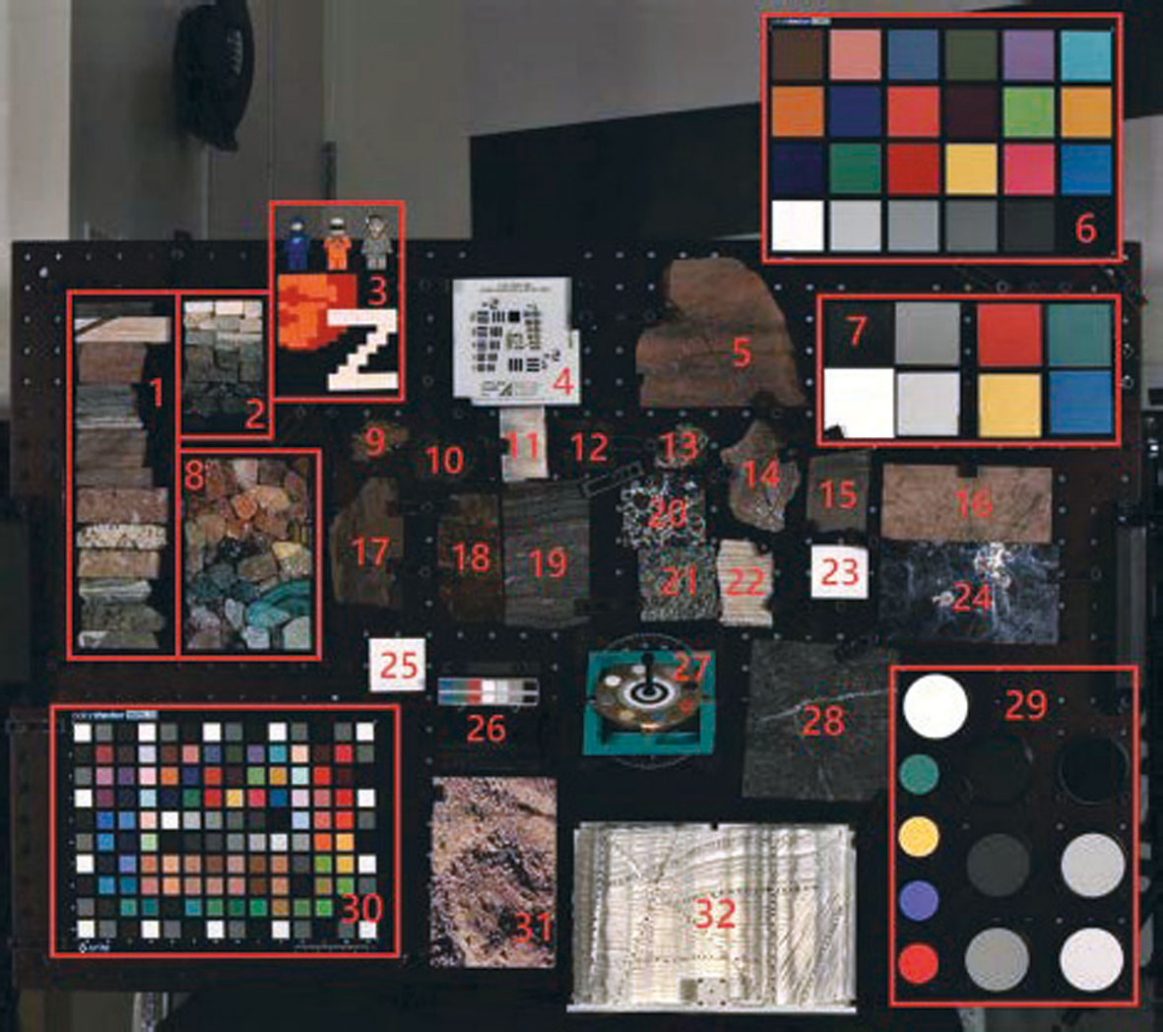
The Mastcam-Z “Geoboard” imaged under ambient pressure and temperature conditions by Mastcam-Z (34 mm focal length, R0 filter, Bayer RGB color). Geoboard targets include color standards, rock slabs, resolution targets, stereo calibration targets, and education and public outreach materials. Target labels correspond to the names and descriptions provided in Table 11. For scale, the grid holes on the board have 1 inch spacing

Independent observations of reflectance standards and well-characterized geologic samples can be used to validate instrument performance and the accuracy of the calibration pipeline. Accordingly, both Mastcam-Z cameraheads observed a “Geoboard” with a collection of color standards, rock slabs, resolution targets, stereo calibration targets, and education and public outreach (E/PO) materials during stand-alone calibration at MSSS (see Fig. 38 and Table 11). Observations of the materials on the Geoboard can be compared to laboratory measurements to constrain expected uncertainties in instrument performance.

**Table 11:** Color standards, rock slabs, resolution targets, stereo calibration targets, and education and public outreach materials (E/PO) included on the Mastcam-Z “Geoboard.” Target numbers correspond to labels shown in Fig. 38

	Category	Description
1	E/PO	Mock stratigraphic column
2	E/PO	Grayscale rocks & minerals
3	E/PO	MCZ Lego logo
4	Resolution	USAF 1951 resolution target
5	Rock (cut and polished)	Precambrian cross-bedded sandstone
6	Color standard	Xrite Color Checker calibration standards
7	Color standard	Mastcam-Z calibration target materials
8	E/PO	Rainbow rocks & minerals
9	Rock (cut)	Vesicular basalt
10	Rock (natural surface)	Weathered vesicular basalt
11	Rock (natural surface)	Selenite gypsum vein
12	Rock (natural surface)	Weathered vesicular basalt
13	Rock (cut)	Carbonate-coated basalt breccia
14	Rock (cut and polished)	Welded tuff with black glass
15	Rock (cut and polished)	AREF 082 rock slab
16	Rock (cut and polished)	AREF 247 rock slab
17	Rock (natural surface)	Weathered Columbia River Basalt
18	Rock (natural surface)	Weathered Pilbara stromatolite
19	Rock (cut and polished)	AREF 414 rock slab (Banded Iron)
20	Rock (cut and polished)	AREF 146 rock slab
21	Rock (cut and polished)	AREF 225 rock slab
22	Rock (cut)	Sulfate/carbonate varve
23	Color standard	Mars-2020 rover deck paint sample
24	Rock (cut and polished)	AREF 248 rock slab
25	Color standard	Mars-2020 rover deck paint sample
26	Color standard	Mastcam-Z secondary calibration target
27	Color standard	Mastcam-Z primary calibration target
28	Rock (cut and polished)	AREF 175 rock slab
29	Color standard	Labsphere color standard targets
30	Color standard	Xrite Color Checker calibration standards
31	Stereo	Stair-step photo target
32	Stereo	3D-printed strike/dip target

The color standards on the Mastcam-Z Geoboard included witness samples of the Mastcam-Z calibration target materials, an optically flight-like “calibration spare” of the Mastcam-Z primary calibration target, a flight-like engineering model of the Mastcam-Z secondary calibration target, and samples of the Mars-2020 rover deck paint, in addition to standards by X-Rite and Labsphere. Rock targets included AREF polished slabs from NASA, which had also been used in the calibration of the Mars Exploration Rover Pancams (Bell et al. 2006) and the Mars Science Laboratory Mastcams (Bell et al. 2017). A variety of rock targets with naturally-weathered surfaces were included (basalt weathering rinds and coatings, vesicular basalts, stromatolite textures, and fibrous gypsum veins), in addition to cut and polished samples with fine-scale textural details and mineralogic variability (banded iron, cross-bedded sandstone, welded tuff with black glass, sulfate/carbonate varve, and carbonate-coated basalt breccia). Space in the upper right of the Geoboard was dedicated to E/PO targets provided by The Planetary Society.

On May 8$^{th}$, 2019, fourteen-filter Mastcam-Z observations of the Geoboard were acquired at three zoom positions (34 mm, 63 mm and 100 mm) under a variety of lighting conditions. Images were calibrated to radiance units using the steps discussion in Sect. 4.1, and the colored calibration target witness samples (target 7 in Fig. 38) were used to find relative reflectance (Fig. 6) using the technique briefly discussed in Sect. 4.1.7 and described in more detail by Kinch et al. (2020, this journal). For each filter, the image data were fit to the laboratory spectra of the calibration target witness samples by a simple linear regression of the values derived from the 8 witness samples. Reduced data products were created for a variety of slope, ratio and band depth parameters to verify Mastcam-Z’s multispectral capabilities (e.g., Fig. 40). A complete analysis of the Geoboard images is being assembled for a separate publication (M.S. Rice, Personal Communication, May 12$^{th}$, 2020).

**Fig. 39:**
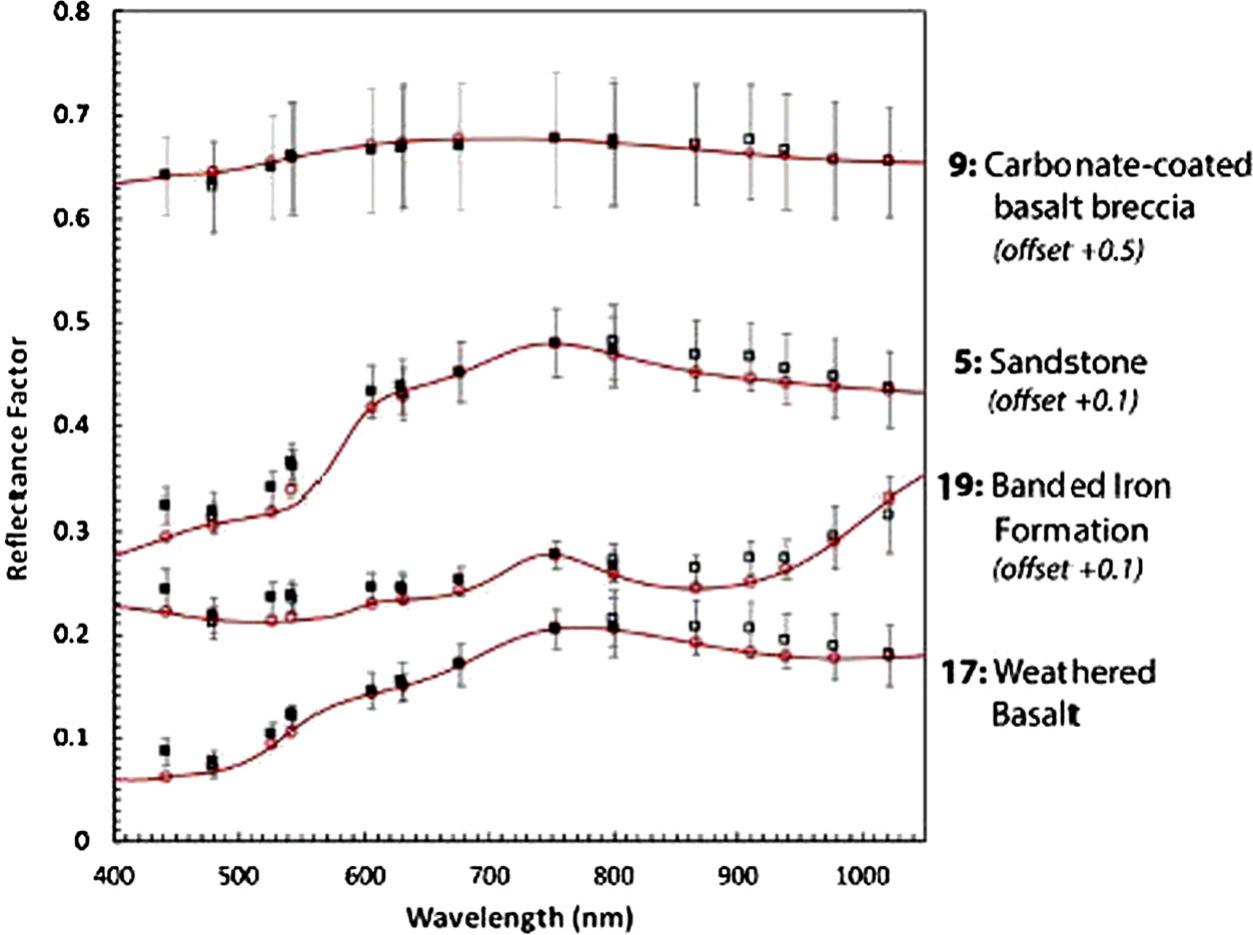
High-resolution laboratory spectra of Geoboard rock targets (solid red lines) and lab spectra convolved to Mastcam-Z bandpasses (open red circles) compared to Mastcam-Z reflectance measurements (Mastcam-Z left = filled black squares; Mastcam-Z right = open black squares). Lab spectra shown were acquired with the contact probe and were normalized to the reflectance value of the Mastcam-Z measurement at 754 nm. Some spectra have been offset for clarity. Root mean square errors between the two datasets are $<3\%$ for all rock targets shown. Error bars shown represent the standard deviation of the Mastcam-Z pixels averaged in each region of interest. Target numbers correspond to the labels on Fig. 38

Mastcam-Z spectra were extracted from the calibrated reflectance factor ($R^{*}$) images by manually selecting regions of interest (ROIs) in the left and right camera images and averaging the reflectance values for all the pixels contained in each ROI. Error bars are shown as the standard deviations of pixel values within the ROIs (Fig. 39). Reflectance profiles of the Geoboard targets were externally validated using a full-resolution laboratory spectrometer at Western Washington University (WWU) in July 2019. An ASD FieldSpec 4 high resolution spectroradiometer, with a spectral range of 350 nm–2500 nm and resolution of 3 nm in the VNIR and 8 nm in the SWIR, was used to collect spectra for the rock targets and color calibration standards. Spectralon SRM-99 was used for a white reference and 200 spectra were averaged to create each spectrum. These spectra corrected for occasional offsets at 1000 nm and 1830 nm, where detector changeovers occur. Using a custom-built goniometer (Hoza and Rice 2019), spectra were collected from a range of viewing geometries, in addition to measurements at a standard geometry using a contact probe (incidence = $23^{\circ }$, emission = $35^{\circ }$).

The spot sizes of goniometer and contact probe measurements were roughly 1 cm in diameter on the sample; care was taken to select the Mastcam-Z ROIs from the same ∼1 cm diameter region on each Geoboard target. The error bars in Fig. 39 indicate the pixel-to-pixel variability in the Mastcam-Z observation within this 1 cm spot (not the instrumental error); for example, the large error bars for the carbonate-coated basalt breccia spectrum are due to averaging the mm-scale, light-toned carbonate coatings with the darker basalt in the ROI. All rock targets on the Geoboard contain some compositional variability at scales below ∼1 cm, and targets with natural, textured surfaces have additional variations due to local incidence angles and shadowing that contribute to the error bars.

Comparisons of the high-resolution laboratory spectra and the Mastcam-Z reflectances of selected Geoboard rock targets are shown in Fig. 39. These targets exhibit varying spectral profiles largely due to iron oxide phases (Target 5, oxidized sandstone; Target 19, Banded Iron Formation; Target 17, weathered basalt; and Target 13, carbonate-coated basalt breccia). Instances where the lab data fall outside the error bars for the Mastcam-Z data (such as for the blue 442 nm narrowband filter) can be attributed to the differing illumination conditions between the Geoboard imaging and WWU lab measurements. To partially account for differences in overall reflectance due to illumination geometry, each lab spectrum was normalized to the reflectance value of the corresponding Mastcam-Z measurement at 754 nm. This first-order correction, however, does not address the non-uniform illumination across the board during calibration. Root mean square errors (RMSEs) between the two datasets are $1-2\%$ for most Geoboard rock targets. Of the targets shown in Fig. 39, the largest discrepancy between the lab and Mastcam-Z data is for the banded iron sample (RMSE = $2.9\%$), which is likely due to slight discrepancies in the proportions of hematite bands included in the two spectral averages.

**Fig. 40:**
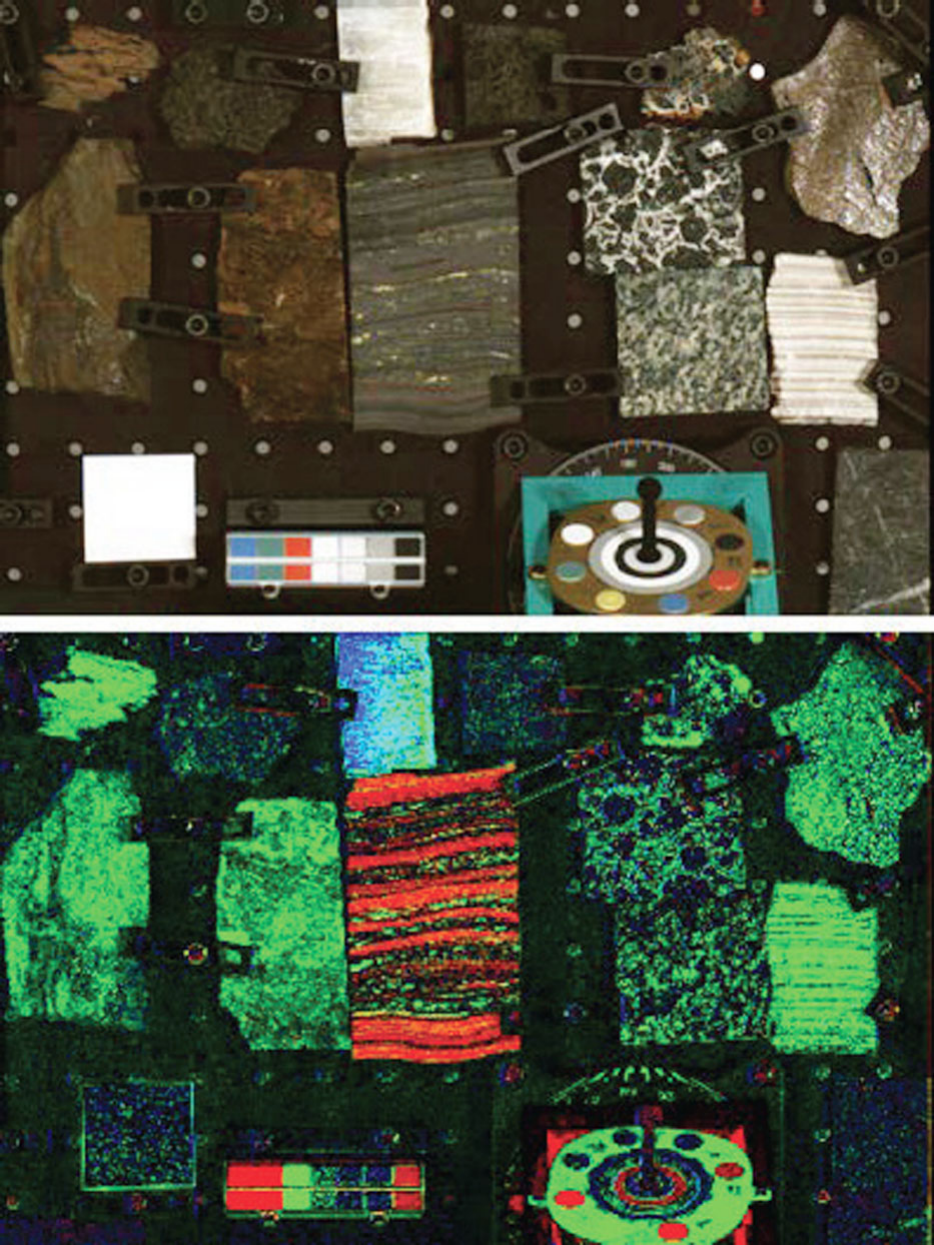
Cropped Mastcam-Z Right 63 mm zoom image of the center of the Geoboard. Above: visible color (R0 filter, Bayer RGB). Below: RGB composite of three parameters that highlight mineralogic variations among the targets. Red = 866 nm band depth (0.05 min, 0.20 max), relative to continuum wavelengths at 637 nm and 1014 nm (consistent with crystalline hematite); green = positive 474 nm to 637 nm slope (0.0 min, + 0.001 max) (consistent with Fe-oxides); blue = negative 939 nm to 978 nm slope (0.0 min, −0.0001 max) (consistent with hydration). For scale, the grid holes on the board have 1 inch spacing

Specific, quantifiable characteristics of the spectra shown in Fig. 39 can be mapped across the full Mastcam-Z field of view. Fig. 40 is an RGB composite of three spectral parameters that highlight mineralogic variations among the targets (red = 866 nm band depth, relative to continuum wavelengths at 637 nm and 1014 nm; green = positive 474 nm to 637 nm slope; blue = negative 939 nm to 978 nm slope). The slope parameter in blue highlights a drop in reflectance at 978 nm caused by a narrow $\mathrm{H}_{2}\mathrm{O}$ absorption centered at 980–1000 nm (e.g., Rice et al. 2010). This parameter distinguishes the hydrated gypsum vein (Target 11) from other Ca-sulfate samples (e.g., Target 22, a varve with anhydrite and minor gypsum alternating with carbonate). The slope parameter in green is a measure of “redness” in visible wavelengths, which is strongly influenced by Fe^3+^ absorptions at short wavelengths (e.g., Singer 1982; Bell et al. 1990), and thus distinguishes the weathered (oxidized) surfaces (e.g., Targets 9, 17, and 18) from unaltered mafics (Target 10). This “red slope” also distinguishes bright targets with minor Fe-oxides (e.g., the Ca-sulfate in Target 22) from bright targets without (e.g., Target 25, the rover deck paint). The band depth parameter in red correlates with the depth of the broad $\text{Fe}^{3+}$ absorption centered near 860 nm in crystalline hematite (e.g., Morris et al. 1985; Brown et al. 2008). This parameter distinguishes the individual hematite-bearing layers in the Banded Iron Sample (Target 19) from the other Fe-oxide-bearing rock surfaces. These parameter composites validate that, on Mars, reduced data products from a limited subset of Mastcam-Z filters can be used to map geologically significant aspects of the spectral variability.

## Data Reduction and Validation

### Radiometric Calibration Pipeline

**Fig. 41:**
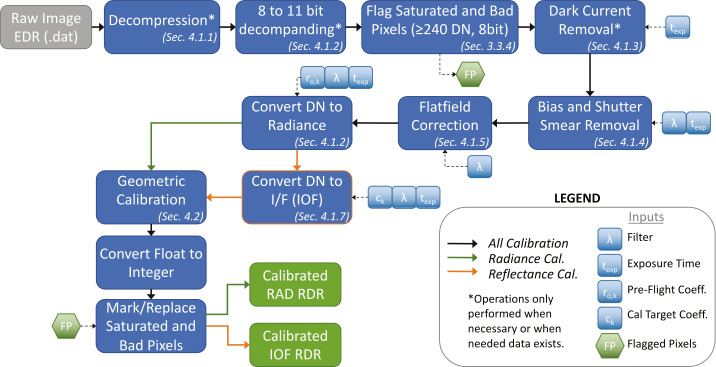
Flowchart outlining the steps in Mastcam-Z radiometric calibration pipeline

Fig. 41 provides an overview of the primary steps in the Mastcam-Z data reduction and calibration pipeline. Full-frame and subframed images are calibrated to radiance and reflectance ($I/F$) products using a variety of pre-flight and, when available, ancillary in-flight data files and coefficients. The sub-sections below will qualitatively explain each step of radiometric pipeline, explaining how the results of the pre-flight calibration described above will be used to generate reduced data products that remove image artifacts and describe calibrated scene radiance (RAD) and/or reflectance (IOF). The tactical details of the calibration pipeline, as it will be implemented in-flight, along with the associated product ID descriptions and header keywords, are still in development at the time of this publication. They will be described in detail in a follow-on publication that reviews in-flight calibration activities after landing (see Sect. 5).

#### Decompression

Similar to MSL Mastcam, raw Mastcam-Z EDRs (Engineering Data Records) can be compressed for downlink (Bell et al. 2017). In the event that they are, the first step in the calibration pipeline is to decompress the data into the proper spatial domain format. Mastcam-Z images are compressed and decompressed using standard JPEG protocols and Bayer interpolation (e.g., Malvar et al. 2004). For a detailed description of the decompression process of JPEG compressed Mastcam-Z data products, which is identical to the process used for MSL Mastcam data products, please see Sect. 5.2.1 of Bell et al. (2017). For losslessly compressed data the Huffman decoding process, described in Appendix C of Malin et al. (2013), is used to decode the images.

#### Decompanding

Companding and decompanding, blends of the words “compressing” and “expanding,” are protocols that reduce pixel values from their original 11-bits (2048 DN range) to 8-bits (256 DN range) and simultaneously rescale the image using a pre-defined look-up-table (LUT) to maximize the utility of the reduced 8-bit dynamic range (Bell et al. 2006, 2017). To maximize the valid pixel range in each 8-bit companded image, a constant DC offset is removed from the 11-bit image prior to applying the LUT. This DC offset, meant to approximate the static bias (see Sect. 3.3.3), is set to the minimum bias value expected during operations (110-120 DN). The most common companding table utilizes a square root based LUT that parallels photon arrival Poisson noise, whose standard deviation is equal to the square root of the signal. This LUT minimizes data loss by compressing from 11-bits to 8-bits in steps sizes proportional to the expected magnitude of the noise (i.e., the shot noise is not encoded). There are 31 additional LUTs that can be used, each with its own application that can be appropriate for specific operational scenarios (e.g., returning 0-255 DN of the 0-2047 DN l1-bit signal of a scene where only the dimmer pixels are of scientific interest). Decompanding is the opposite process, converting the downlinked 8-bit data into an approximation of the original 11-bit data. Together, (de)companding relates the 11-bit pixel values captured by the ADCs and the 8-bit values that are downlinked to Earth. The companding table used for 8-bit frames acquired during calibration was Table 0 (Bell et al. 2017), which has a square-root dependence (i.e., $DN_{8\mathit{bits}} = [32*(DN_{11\mathit{bits}}-DC\_\mathit{OFFSET})]^{1/2}$ in integers). More details on companding and decompanding are provided in Bell et al. (2017) and Bell et al. (2020, this journal).

#### Dark Current Removal

Dark current results from the random generation of electrons within the detector and has an exponential thermal dependence. During stand-alone testing, the dark current was measured as a function of temperature through a series of long duration exposures, with the solar filters in place, and with no incident light on the detectors (see Sect. 3.3.2). The average dark current of the central $100\times 100$ pixels was measured to be:

25 $$ \begin{aligned} DC_{L}(T) = (20.4 \pm 9.5)e^{[(0.088 \pm .016)[^{\circ}C^{-1}]T]} [e^{-}/s] \\ DC_{R}(T) = (20.6 \pm 9.2)e^{[(0.086 \pm .016)[^{\circ}C^{-1}]T]} [e^{-}/s] \end{aligned} $$

Both of these models agree well with the dark current models published for Mastcam (Bell et al. 2017). It should be noted, however, that the fit is dominated by measurements at higher temperatures, where there is greater signal, and therefore over-predicts the dark current at colder temperatures ($<+20^{\circ}$ C). We also note that the dark current is pixel-dependent, though no hot pixels were measured during stand-alone calibration. Because the dark current is <1 DN/s for temperatures $\lesssim -15^{\circ }$ C and Mastcam-Z integration times are typically in the few to tens of milliseconds, it can be safely ignored for the majority of images acquired on Mars. Dark current only needs to be accounted for in off-nominal situations such as long integration times (e.g., dusk or night-time sky imaging) or images acquired with higher than normal FPA temperatures. When the dark current model (Equation (25)) predicts <1 DN (i.e., $<16$ $e^{-}$) of signal for any given image, no correction is applied. If the model predicts >1 DN (i.e., $>16$ $e^{-}$) of signal, however, the dark current will be removed by using the model to normalize the closest dark current map. During stand-alone calibration, dark current maps were acquired at $-10^{ \circ }$ C, $-5^{\circ }$ C, $20^{\circ }$ C, $25^{\circ }$ C, $30^{\circ }$ C and $40^{\circ }$ C. During cruise, additional dark current observations will be acquired (see Sect. 5). For an observation acquired at temperature ($T$) and exposure time ($t$) with a modeled dark signal $>1DN/s$, the signal [$DN$] to be removed is:

26 $$ D_{ij,T} = D_{ij,T_{o}} \, t \: \frac{DC(T)}{DC(T_{o})}, $$

where $D_{ij,T_{o}}$ is a bias-subtracted dark current map in [$\frac{DN}{s}$] acquired at temperature $T_{o}$, and $\frac{DC(T)}{DC(T_{o})}$ is the ratio of the dark current model at temperature $T$ and $T_{o}$, respectively. Note that the scaled dark current maps are more accurate when $T$ and $T_{o}$ are close in temperature.

#### Transfer Smear, Ghost and Bias Correction

Mastcam-Z zero-second exposures show two components, a temperature-dependent static bias and temperature independent “shutter smear” that is proportional to the scene flux (see Sect. 3.3.3). Because Mastcam-Z does not have a shutter, light falling on the CCD just before integration begins is smeared across the array as it is transferred to the serial register. Both components are present in every acquired image. Ideally, removing these components is straightforward—simply subtract a zero-exposure or “shutter” frame acquired coincident with the commanded image. Alternatively, two images could be commanded at different exposure times and analysis could be conducted on the difference between them as it takes the same amount of time and data volume, ignoring compression differences, to acquire and downlink a shutter frame as it does an exposed frame. When shutter frames or multiple exposure times are not acquired, however, the transfer smear and bias must be modeled and removed.

The static bias is the easier of the two to correct. During stand-alone dark current testing, zero-second exposures were acquired at multiple temperatures with the solar filter in-place under low ambient illumination (i.e., no incoming flux on the detector). These frames are wholly composed of the static bias. An analysis of this dataset showed that there is a mild (5-10 DN) temperature dependence to the static bias (see Sect. 3.3.3) that leads to slightly higher, but more uniform (i.e., less variance), bias at colder temperatures. If no bias frame is provided, the radiometric pipeline approximates the static bias by finding the measured static bias calculated at the closest temperature point and subtracting it. During stand-alone testing, we acquired proper static bias frames at − 10^∘^ C, $-5^{\circ }$ C, $25^{\circ }$ C, $30^{\circ }$ C, and $40^{\circ }$ C. Additional static bias frames will be acquired during cruise and in-flight to cover additional temperature points. If required, additional temperature points may be derived from V&V datasets acquired in the MSSS TVAC chamber.

The second component of Mastcam-Z zero-second exposures is a combination of transfer smear and ghost image effects (see Sect. 3.3.3) whose magnitude is proportional to scene illumination. While the cause of the ghost image is not completely understood, it can be approximated as an extra non-uniform integration time across the detector. The magnitude of this extra integration time, $t_{sm,\mathit{ijk}}$ (see Equation (1)) is wavelength dependent, but analysis suggests it is temperature independent (see Sect. 3.3.3). In the lower-right hand corner of the detector, furthest from the video readout, the transfer smear and ghost can represent up to $\sim 10\%$ of the image brightness for a 6 ms L0/R0 exposure. The shape of this component can be seen in Fig. 18 and its magnitude for each filter is listed in Table 4.

In the absence of an acquired shutter frame, the transfer smear can be removed by scaling the static bias and dark subtracted frame by the ratio ($\frac{t}{t+t_{sm,\mathit{ijk}}}$), where $t$ is the commanded exposure time. This is similar to a flatfield correction (see Sect. 4.1.5):

27 $$ DN_{\mathit{cor},ijkl} = (DN_{\mathit{ijkl}} - B_{ij,T} - D_{ij,T}t/g) \frac{t}{t+t_{sm,\mathit{ijk}}}, $$

where $DN_{\mathit{ijkl}}$ is the observed image after decompression and decompanding, $D_{ij,T}$ is the appropriate dark current map in [$\frac{e^{-}}{s}$] (see Equation (26) and Sect. 4.1.3), $B_{ij,T}$ is the static bias, and $t_{sm,\mathit{ijk}}$ is the extra integration time that approximates the ghost and transfer smear. Ideally, because of the complexities associated with modeling the transfer smear and ghost, a shutter (zero-exposure) frame should be taken for each observation that requires high radiometric accuracy. In that case, both the bias and transfer smear are properly captured and removed from the frame.

Note that, when using companding to return 8-bit data, a $DC\_\mathit{OFFSET}$ term is subtracted from the frame before applying the LUT. This is meant to approximate (and remove) the bulk “bias” by subtracting a constant value close to (but less than to avoid negative values) the actual bias from the frame prior to companding it to 8-bit. This maximizes the dynamic range available to the companding process. Before calibrating any companded data, it should first be decompanded and the $DC\_\mathit{OFFSET}$ added back in (see Sect. 4.1.2).

#### Flatfield Correction

As described in Sect. 3.6, the flatfield correction ($F_{\mathit{ijkl}}$) is designed to correct for pixel-to-pixel variations as well as lower frequency (e.g., optical) variations across the scene. The flatfield correction is designed as a multiplicative correction that will bring every pixel to the same average response. Ideally, we would have flatfields for every filter and zoom configuration but, unfortunately, time constraints precluded generation of a full suite of flatfield correction maps. Rather, we have flatfield maps from stand-alone calibration that cover every filter at 34 mm, 63 mm, and 100 mm. When observations are acquired at one of these focal lengths, the pipeline uses the flatfield correction generated during stand-alone calibration. When observations require flatfields outside of these three focal lengths, however, we must generate one. In order to do so, we break the flatfield into two components: a low frequency component that accommodates smoother optically-induced variations, and a high frequency component that accommodates pixel-to-pixel responsivity variations.

The low frequency component is primarily a function of focal length, which can induce variations of ∼± 20% across the field of view as the zoom mechanism varies focal length across its full range. To account for these variations, we derived a subset of the full flatfield correction maps for L0/R0 at a series of 67 zoom motor positions covering the range of motion acquired during the continuous zoom test (see Sect. 3.6). Because of time constraints, the continuous zoom dataset was collected at 1 frame per ISOP / exposure time using 8-bit companding, and as a result the per-pixel derivations are quite noisy. The lower frequency components of the resulting flats, however, are robust. To remove high frequency noise, the resultant flatfield correction maps derived from each zoom motor position is passed through a 25-pixel median average to create a low frequency flat. At each zoom position, flatfield correction maps are calculated from a combination of two flux levels and integration times (see Sect. 3.6) to derive a per-pixel responsivity in DN/s and scale this on a per-pixel basis, as described in Sect. 3.6:

28 $$ f_{\mathit{ijk}0} = \frac{\frac{\bar{DN}}{s}_{k0,\mathit{center}}}{\frac{DN}{s}_{\mathit{ijk}0}} $$

where we use $f$ to denote that the flat has been median filtered, $\frac{\bar{DN}}{s}_{k0,\mathit{center}}$ is the per-color responsivity average over pixel dimensions ($i,j$) in the center of the array, for the given intermediate zoom, $k$, and filter $l=0$ (continuous data was only taken for R0/L0).

To bring the high frequency information back in, we use the full-resolution flatfield map for the closest canonical zoom position derived from the radiometric dataset. To remove the low frequency component of the canonical zoom’s flatfield, we then divide by the low frequency flat at the canonical zoom position such that:

29 $$ \tilde{F}_{\mathit{ijk}0} = f_{\mathit{ijk}0} \cdot f_{ij\tilde{k}0} $$

where $\tilde{k}$ denotes the closest canonical zoom position. Note that we are not removing the low frequency contribution with this ratio, we are simply scaling it to match the low frequency contribution at the closest canonical zoom. The master flat generated at that canonical zoom can then be used to correct for both the high and low frequency contributions. The final flatfield correction is the multiplication of the canonical zoom position at full resolution, $\hat{F}_{\mathit{ijkl}}$, by the median filtered flat ratio:

30 $$ \bar{F}_{\mathit{ijkl}} = \hat{F}_{\mathit{ijkl}} \cdot \tilde{F}_{\mathit{ijk}0} $$

It should be noted that the continuous zoom dataset was only collected for the R0/L0 filters. Therefore, when applying this low frequency correction to other filters, we assume that there is no spectral dependence. Because the low frequency component of the flats should result primarily from the optical throughput, this is likely a good assumption, though the filter is part of the optics and scattering from the edge of filters has been observed in a few flatfield corrections (see Fig. 21), so there may still be some factor that is not properly accounted for. Accordingly, we recommend using Martian sky radiance observations to create specifically tailored flatfield corrections if a non-canonical zoom / filter pairing becomes part of a standard imaging sequence on Mars.

To test the validation of this correction, Equations (28)–(30) were applied to a subset of the radiometric coefficient dataset to compare modeled flats to those measured at the canonical 63 mm position for each filter. We find an average difference between the measured and predicted flats with an average difference of <1% and a standard deviations of <4% for all filters, demonstrating that the routine for calculating flats at non-canonical zoom is robust and that the continuous flats taken in R0/L0 can be used to approximate the low frequency variations in other filters.

#### Convert DN to Radiance

Once Mastcam-Z images have been dark current subtracted, bias corrected, and flatfielded they can be converted to units of photon flux ($\langle L_{\lambda } \rangle _{\mathit{ijkl}}$ $[\frac{ph}{{s}\, {m}^{2}\, {sr}}]$) using Equation (2) in Sect. 3.1. The output of this calibration is the average in-band photon radiance $[\frac{ph}{{s}\, {m}^{2}\, {sr}}]$ incidence upon the entrance aperture of the camera. Photon radiance can be converted into the more common radiance units [$\frac{W}{m^{2} \, sr}$] by dividing by $\frac{ \lambda _{\mathit{eff},k}}{h\, c}$:

31 $$ \langle L_{\lambda }^{\prime } \rangle _{\mathit{ijkl}} \: \biggl[\frac{W}{m^{2} \, sr}\biggr] = \langle L_{\lambda } \rangle _{\mathit{ijkl}} \biggl[\frac{ph}{s\, m^{2}\, sr}\biggr] \frac{h\, c}{ \lambda _{eff,k}} \biggl[\frac{J}{ph}\biggr] $$

where $\lambda _{eff,k}$ is the weighted average of the wavelength and filter $k$’s spectral response (see Sect. 3.5 and Equation (5)). The multiplicative constant that converts DN to photon radiance is ($\frac{g}{A_{o}\varOmega _{l} \, r_{o,kT}\, t}$) (see Equation (2)), where $g$ is $15.6\pm 0.2$ [$\frac{e^{-}}{s}$] (left) or $15.6\pm 0.4$ [$\frac{e^{-}}{s}$] (right), respectively, and $t$ is the commanded exposure time. The optical throughput ($A_{o}\varOmega _{l}$) is given by Equation (4) (Sect. 3.4) and the radiometric coefficient $r_{o,kT}$ [$\frac{e^{-}}{ph}$] at temperature $T$ [$^{\circ }\mbox{C}$] is:

32 $$ r_{o,k}(T) = r_{o,k}(T_{\mathit{ref}}(1+\beta _{k}(T-T_{\mathit{ref}})) \, \left [ \frac{e^{-}}{ph} \right ] $$

where $r_{o,k}(T_{\mathit{ref}})$ is the ambient temperature ($T_{\mathit{ref}}=-5^{ \circ }~\mathrm{C}$) radiometric coefficient described in Sect. 3.7 and $\beta $ is the measured temperature correction term described in Sect. 3.7.1, both of which are listed in Table 6.

Note that, for 34 mm, 63 mm, or 100 mm focal lengths, radiance ($\langle L_{\lambda }^{\prime } \rangle _{\mathit{ijkl}} \, [\frac{W}{m^{2} \, sr}]$) can be directly determined by multiplying dark current subtracted, bias subtracted, and flatfielded images by the radiance radiometric coefficients presented in Table 7 and then dividing by exposure time. In addition to the weighted responsivity ($r_{o,k}\, [ \frac{ph}{e^{-}}]$), the radiance radiometric coefficient ($r_{o,kl}^{\prime }$ [$\frac{W}{\frac{DN}{s} \, m^{2}\, nm, sr}$]) folds in the optical throughput, gain, and $\frac{ph}{s}$-to-$W$ conversion into a single term:

33 $$ r_{o,kl}^{\prime } = \frac{g}{A_{o}\varOmega _{l} \, r_{o,kT}}\, \frac{h\, c}{ \int \bar{r_{k}} \lambda d\lambda }. $$

where $r_{o,kl}^{\prime }$ can be scaled to other zoom positions by scaling by the ratio of the optical throughput ($A_{o}\varOmega _{l}$, see Sect. 3.4 and Table 7). Note that $r_{o,kl}^{\prime }$ still has to be scaled for the observed temperature dependence in $r_{o,k}$ (Equation (32), Sect. 3.7.1).

$\langle L_{\lambda }^{\prime } \rangle _{\mathit{ijkl}} $ is the standard output for the Mastcam-Z calibrated radiance (RAD) reduced data products. When looking at surface targets on Mars, $\langle L_{\lambda } \rangle _{\mathit{ijkl}}$ is the integrated in-band solar reflectance:

34 $$ \langle L_{\lambda } \rangle _{\mathit{ijkl}} = \frac{ \int \bar{r_{k}} \rho _{\lambda } \, \tau _{\lambda }\, \frac{S_{o\lambda }}{\pi \, d_{\mathit{sun}}^{2}} \frac{\lambda }{hc} d\lambda }{ \int \bar{r_{k}} d\lambda } $$

where $\rho _{\lambda }$ is the target reflectance ($I/F$ at wavelength $\lambda $) within the IFOV of pixel $(i,j)$, $\tau _{\lambda }$ is the atmospheric transmission at the time of observation, $S_{o}$ is the solar flux at the top of the Earth’s atmosphere in [$\frac{W}{m^{2}\, sr\, nm}$] (AM0, (Wehrli 1985)) and $d_{\mathit{sun}}$ is the distance to the Sun in AU. The weighted average of the target $I/F$ ($\rho _{\lambda }$) and atmospheric transmission ($\tau _{\lambda }$) product can be found by dividing $\langle L_{\lambda } \rangle _{\mathit{ijkl}}$ by the weighted average of the solar spectral irradiance and filter $k$’s band-pass at the top of the Martian atmosphere:

35 $$ \begin{aligned} &F_{\mathit{sun}}= \frac{ \int \bar{r_{k}} \, \frac{S_{o\lambda }}{\pi \, d_{\mathit{Sun}}^{2}}\, \frac{\lambda }{hc} d\lambda }{ \int \bar{r_{k}} d\lambda } \\ &\langle \rho _{\lambda } \, \tau _{\lambda }\rangle _{\mathit{ijk}} = \frac{\langle L_{\lambda } \rangle _{\mathit{ijkl}}}{F_{\mathit{Sun},k}\, (\frac{1.38}{d_{\mathit{Sun}}})^{2}} = \frac{\langle L_{\lambda }^{\prime } \rangle _{\mathit{ijkl}} \, \langle \lambda \rangle _{k}}{h\, c\, F_{\mathit{Sun},k} \,(\frac{1.38}{d_{\mathit{Sun}}})^{2} } \end{aligned} $$

where $F_{\mathit{sun},k}$ is the in-band spectral irradiance at the top of the Martian atmosphere at perihelion (1.38 $\mathrm{AU}$). For MSL, an initial $I/F$ estimate for $\langle \rho _{\lambda }\, \tau _{\lambda }\rangle $ was created, and delivered to the PDS, by comparing the measured $DN$ (post bias, flat, and dark correction) to the expected $DN_{\mathit{eff}}$ from viewing a perfectly white Lambertian scatterer, ignoring the atmosphere (i.e., $\rho _{\lambda }=1$, $\tau _{\lambda }=0$):

36 $$ DN_{\mathit{eff}, kl} = \frac{A_{o}\varOmega _{l}\,t\,r_{o,k}}{g} F_{\mathit{Sun},k} \, \frac{t}{10\, \mathrm{ms}} \, \biggl(\frac{1.38 \, \mathrm{AU}}{d_{\mathit{Sun}}}\biggr)^{2} $$

Values of $DN_{eff, kl}$ for each filter at $l=100$ mm, $t=10$ ms, and $d=1.38$ AU are listed in Table 2.

#### Convert from RAD to IOF Using the Calibration Target

The problem with Equation (35)’s estimate $\langle \rho _{\lambda } \tau _{\lambda } \rangle $ is that it still includes the effects of the Martian atmosphere. In order to approximately remove $\tau _{\lambda }$ during operations on the Martian surface, images of the radiometric calibration targets will be regularly acquired with multi-spectral sequences and/or when radiometric or color accuracy is required. A laboratory-based reflectance model can then be compared to the observed reflected radiance of all color and grayscale materials on the calibration target as a function of wavelength and illumination geometry. Radiance values will be derived from the images for all color and grayscale surfaces. For each image, a plot of observed radiance versus model reflectance will show data points for the different color and grayscale materials falling along a straight line. The slope of this line is a measure of the instantaneous irradiance and provides a direct conversion factor from units of radiance to units of reflectance ($I/F$ or IOF). This procedure is similar to the procedure employed for MER Pancam (Bell et al. 2006) and MSL Mastcam (Bell et al. 2017).

Early in the mission, before significant dust deposition on the calibration target, this straight line should pass through the origin (zero reflectance should give zero observed radiance). This prediction can be used to monitor the quality of the radiance calibration. Inaccuracies in additive terms such as bias and dark current will result in a line that does not pass through the origin. This procedure was employed to process images of the Geoboard target (see Sect. 3.12) using the calibration target witness materials (Fig. 38, target 7). The results agreed with laboratory spectra of the witness samples to $< 5\%$ RMS. This is a conservative estimate of the calibration error as the non-solar color spectrum of the light sources was not taken into account, leading to larger deviations in the broadband filters, and also because it folds in errors in the laboratory spectra themselves.

As the mission progresses dust will accumulate on calibration target surfaces. The 8 circular patches will remain relatively dust-free because of the presence of the cylindrical permanent magnets underneath, but even these will experience some non-magnetic dust deposition. In order to correct for the presence of dust we will employ a method described in full detail by Kinch et al. (2015) which has been successfully employed both on MER and MSL. The dust correction procedure fits the observed radiance values to an analytical two-layer scattering model based on the work of Hapke (2012, Sect. 9.D.2). This derives a value for the thickness of deposited dust, which can be tracked as a function of time both for magnetically-protected areas and for the central grayscale rings that are less protected.

For more details on the operational use of the Mastcam-Z radiometric calibration targets, please see Kinch et al. (2020) in this journal.

### Geometric Calibration Pipeline

Precise camera models are essential for many of the rover’s navigation functions and scientific measurements in the Martian environment. The geometric calibration pipeline uses the camera models to remove geometric distortion and linearize, or rectify, downlinked images. These calibrated data products are then used to produce a wealth of reduced data products including image mosaics, digital terrain meshes, maps of illumination geometry and other derived products for engineering and science use. At least three laboratories (JPL, JR, and DLR) are developing pipelines to process Mastcam-Z images into physical measurements of the terrain. JPL is developing a next-generation system for closed-loop pointing of all cameras on the rover as well as a cloud-based tools to visualize individual images and products derived from them, such as XYZ and surface normals (Web Marsviewer), and to visualize the rover in a 3D terrain environment for planning targeting (ASTTRO). The JPL pipeline is the official Geometric Calibration Pipeline that will be used to remove image distortion within the Radiometric Calibration Pipeline and produce standard products delivered to the PDS. The JPL tools will make use of the heritage CAHVOR camera model derived from the metrology-dependent analysis. JR is developing PRoViP for stereo reconstruction and PRo3D (Barnes et al. 2018) to visualize the terrain and make geometric measurements such as strike and dip. ProViP and PRo3D will utilize the OpenCV camera model derived from the pure-photogrammetric approach. The Mastcam-Z operations team is developing a software package called Viewpoint to visualize operations and planning (Proton 2011). Similar to the JPL pipeline Viewpoint, which has its roots in software developed for MER and MSL, will make use of the CAHVOR camera model. Finally, the Mastcam-Z science team has developed various tools for their individual science programs that make use of the camera models to perform tasks such as measuring target orientations (e.g., strike and dip of bedding planes) and scale (Hayes et al. 2011), co-registering filters from both eyes into single multi-spectral image stacks (Wellington et al. 2017), or producing photometry data cubes that co-register observations from multiple image geometries (Johnson et al. 2021). These tools will make use of both the CAHVOR and OpenCV camera models, depending on the preference of the individual investigator. To ensure consistency between these various software packages, both the CAHVOR and OpenCV camera models will be validated and cross-checked during inflight calibration on Mars (see Sect. 5). Additional details on the data products that will be generated by these pipelines will be discussed in follow-on publications that present the full geometric OpenCV and CAHVOR camera model results.

## Expected in-Flight Calibration Activities

In-flight calibration and testing occurs after launch. Some activities are accomplished during instrument health check-outs during the cruise phase, while others occur during normal, routine operations on Mars. In-flight calibration is used to verify the accuracy of pre-flight calibration and identify changes in camera performance. In some cases, substantial improvements to the pre-flight calibration can be made *in-situ* on Mars (e.g., Bell et al. 2017). For Mastcam-Z, in-flight calibration activities will include verification of the observed radiometric temperature dependence (see Sect. 3.7.1) using images of the calibration target color patches, updating the bad pixel map, verifying the bias/smear model and collecting additional bias, shutter, and dark frames under various conditions, validating the stray light analysis, and updating the flatfield correction maps using images of the Martian sky. The temperature dependencies of the geometric camera model will also be tested and, if required, updated using comparisons to coincident NavCam measurements, as the NavCam optical designs are less susceptible to thermal effects (Maki et al. 2020). Multi-spectral observations of natural targets will be used to update the affine corrections between filters (see Sect. 3.11.1). Within the first month on Mars, a panorama of the rover deck, which includes a collection of surveyed fiducial and focus marks, will be acquired to verify that camera pointing and geometry have not changed since ATLO. Similarly, periodic nighttime images of bright standard stars and/or the Martian satellites, Phobos and Deimos, will be acquired to provide an additional method for verification of the accuracy and stability of the radiometric calibration. Further, imaging of well-characterized constellations will be used to verify the accuracy and stability of the geometric calibration and validate any required temperature corrections. A brief summary of expected in-flight calibration activities is described in the Calibration Plan (JPL Document D-101345), a copy of which is provided in the Supplementary Online Material of this manuscript.

## Summary

This paper describes pre-flight calibration of the Mars 2020 rover Mastcam Zoom (Mastcam-Z) Multispectral, Stereoscopic Imager (Bell et al. 2020). The results discussed herein will enable raw Mastcam-Z images to be radiometrically and geometrically calibrated following downlink to Earth. Detector parameters and optical properties have been sufficiently determined to permit conversion of raw DN values into reliable estimates of in-band radiance [$\frac{W}{\mathrm{cm}^{2}\, \mathrm{sr}}$] and reflectance ($I/F$) with absolute errors of $\sim 5\%$ ($\sim <10\%$ for images acquired at untested camera states, see Sect. 3.7), exceeding the $\pm 10\%$ radiometric accuracy requirement. Comparison between normalized reflectance ($\bar{I/F}$) and well-characterized Geoboard targets suggest a relative calibration accuracy of $<5\%$, with most targets matching laboratory measurements to an RMS error of $1-2\%$ (see Sect. 3.12). The optical quality of Mastcam-Z’s zoom lenses is excellent, with MTF values ranging from 0.26 to 0.55 across all non-solar filters and focal lengths at Nyquist sampling (47 l.p./mm or 0.35 l.p./pixel), exceeding the $\mathit{MTF}_{\mathit{Nyquist}} > 0.2$ requirement. Stray light rejection is better than $6\times 10^{-8}$ at all focal lengths for rays entering $>30^{\circ }$ off the optical axis and the geometric calibration has demonstrated that the camera’s intrinsic geometric properties are relatively stable to changes in temperature, focus, focal length, and filter position.

While time constraints necessitated that calibration only be conducted over a subset of camera state parameters, models have been derived and verified to permit interpolation to zoom and/or focus positions not evaluated during stand-alone testing. Some system properties, such as the radiometric coefficient ($r_{o,k}$), bias ($B_{ij}$), dark current ($D_{ij}$) and MTF were found to be temperature dependent. Temperature-dependent models have been developed and verified, which will allow extrapolation of these parameters to conditions on Mars. The radiometric and geometric properties derived during pre-flight calibration will be verified and, if necessary, updated during in-flight calibration activities.

**Fig. 42:**
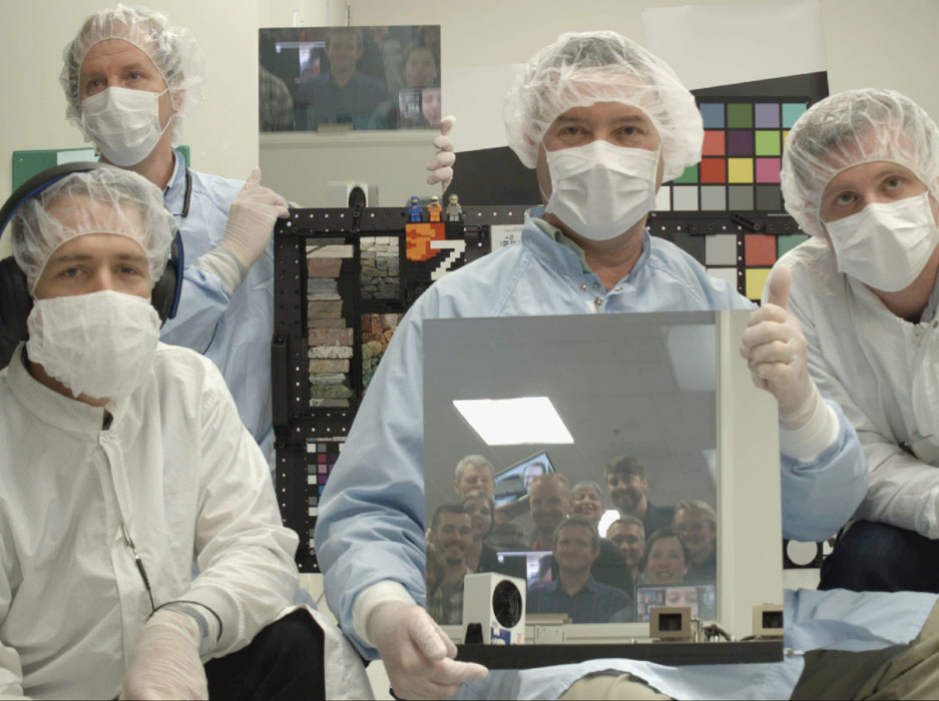
Mastcam-Z self-portrait acquired at the end of stand-alone calibration in MSSS’s clean room on May 8$^{th}$, 2019. The flight cameras can be seen in the larger mirror, held by investigation PI Jim Bell. Deputy-PI Justin Maki is holding the smaller mirror in the back. On the far right is Calibration Lead Alex Hayes and, on the left, is technician Andrew Winhold. Members of the Mastcam-Z science and engineering teams are standing next to a window in the adjacent control room, and can be seen in the mirror reflections. (This image is rotated 180-degrees and white balanced)

**Fig. 43:**
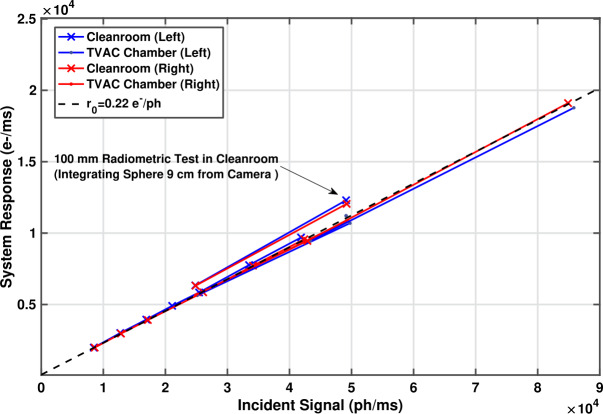
Comparison of L0/R0 radiometric results for all integrating sphere configurations from both the radiometric (Sects. 3.6 and 3.7) and photon transfer (Sect. 3.3.1) datasets obtained in the cleanroom and TVAC chamber. The x-axis shows expected signal in $\frac{ph}{ms}$, calculated using Equation (1). The y-axis shows observed response of the central $100\times100$ pixels in $\frac{e^{-}}{s}$ ($\frac{DN}{t}g$). The slope of the line is the system responsivity $r_{o,L0/R0}$. With the exception of the 100 mm radiometric dataset from the cleanroom, all observations are consistent to within $\sim 2\%$ regardless of zoom position or integrating sphere configuration

**Fig. 45:**
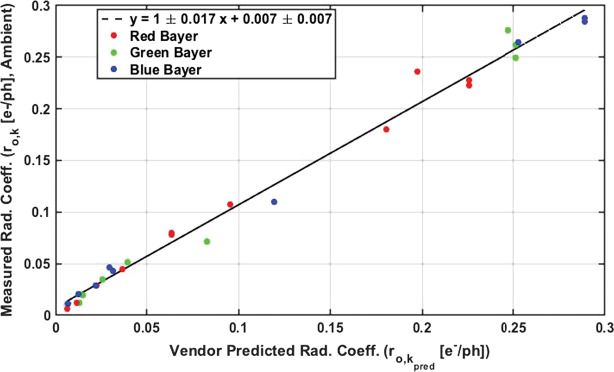
Comparison between ambient radiometric coefficients derived during pre-flight calibration (Table 12 and vendor predictions based on the multiplication of expected detector quantum efficiency, filter transmission, and modeled Mastcam-Z optical transmission. To within error, the best-fit line is consistent with a 1:1 correspondence

**Fig. 46:**
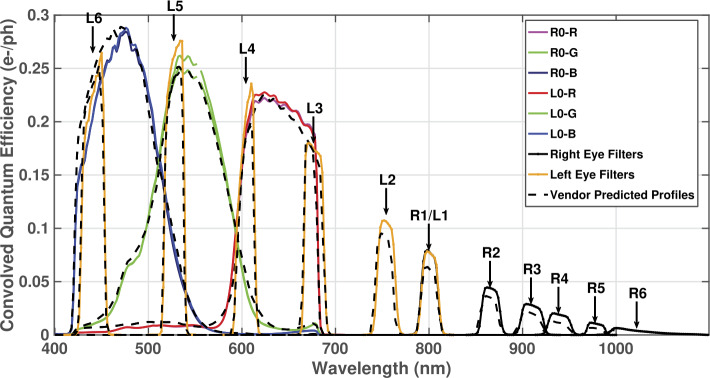
Comparison between measured Mastcam-Z spectral profiles at ambient conditions and vendor predictions, based on the multiplication of expected detector quantum efficiency, filter transmission, and modeled Mastcam-Z optical transmission. While Mastcam-Z shows slightly better efficiency for the infrared filters, the profiles are remarkably consistent

**Fig. 47:**
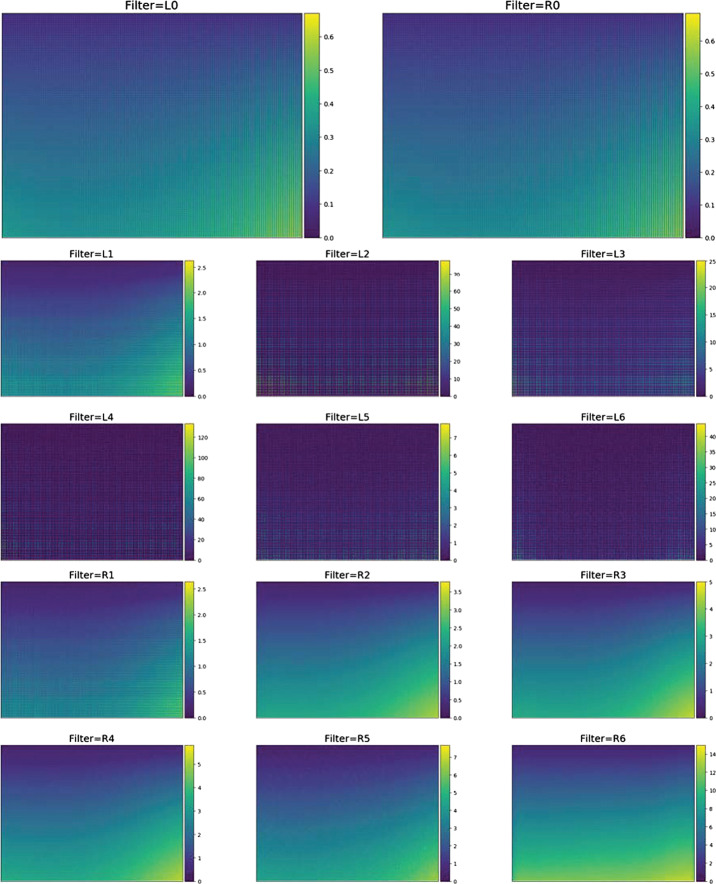
Dynamic bias as a function of filter derived from the 100 mm zoom radiometric dataset. The dynamic bias is found to be independent of zoom position. Large derived values for left eye filters result from low signal in the Bayer colors that are not illuminated depending on the narrowband filter central wavelength. For these filters, only actively illuminated pixels are averaged to calculate the values found in Table 4. See Sect. 3.3.3 for details

**Fig. 48:**
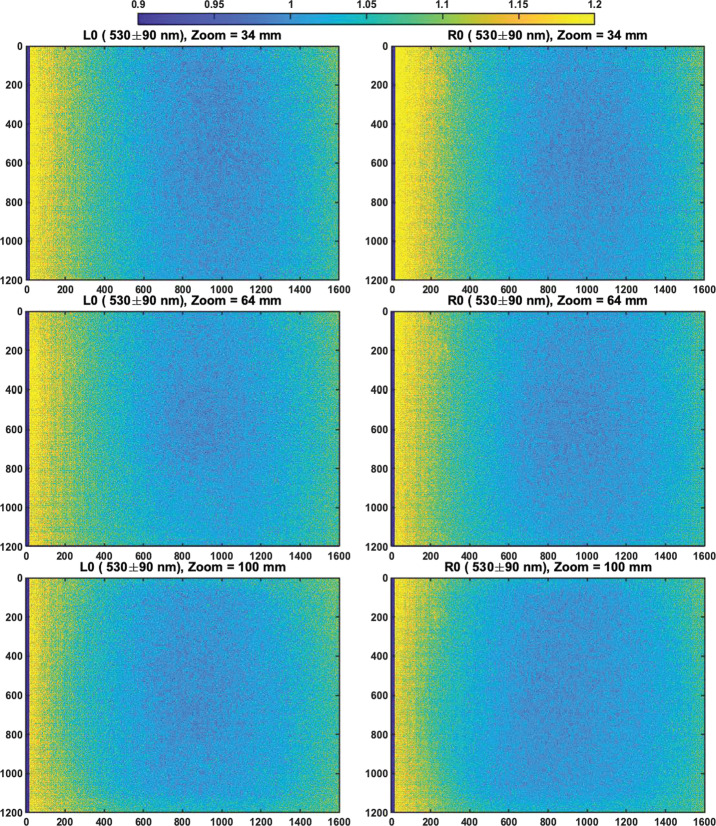
Flatfield correction maps for Filters L0/R0 at 34 mm, 63 mm, and 100 mm focal lengths. See Sect. 3.6 for details

**Fig. 49:**
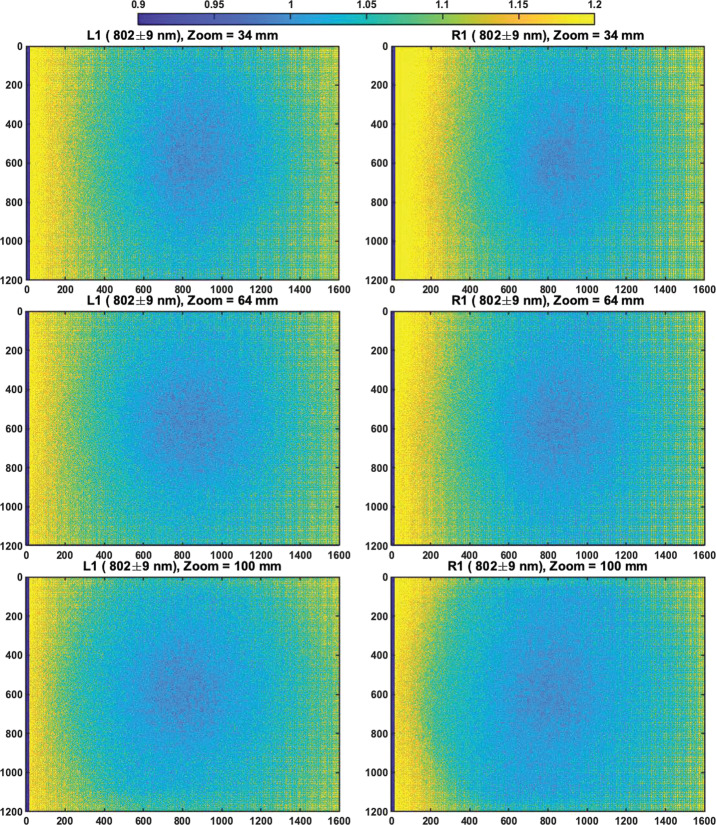
Flatfield correction maps for Filters L1/R1 at 34 mm, 63 mm, and 100 mm focal lengths. See Sect. 3.6 for details

**Fig. 50:**
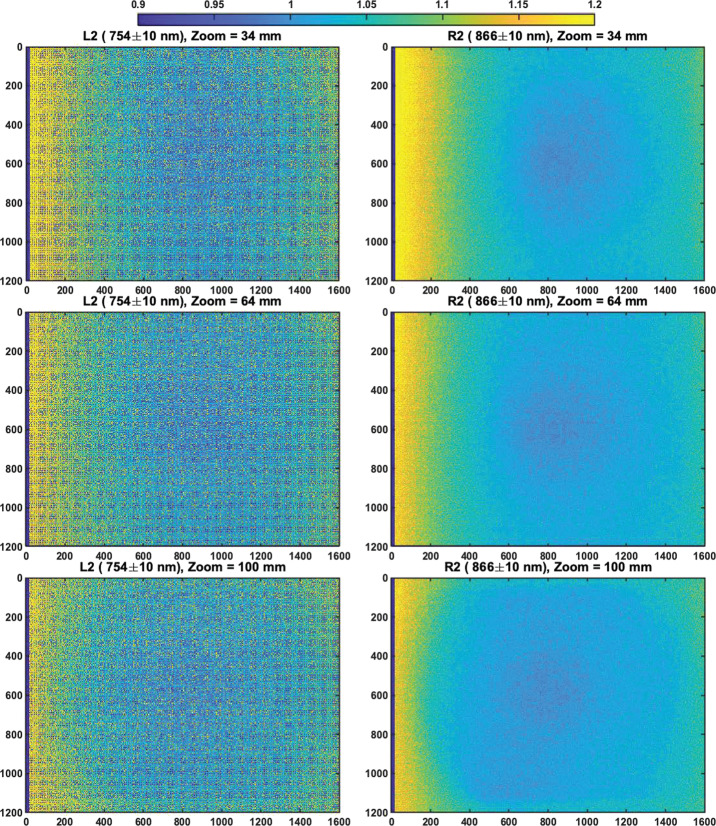
Flatfield correction maps for Filters L2/R2 at 34 mm, 63 mm, and 100 mm focal lengths. See Sect. 3.6 for details

**Fig. 51:**
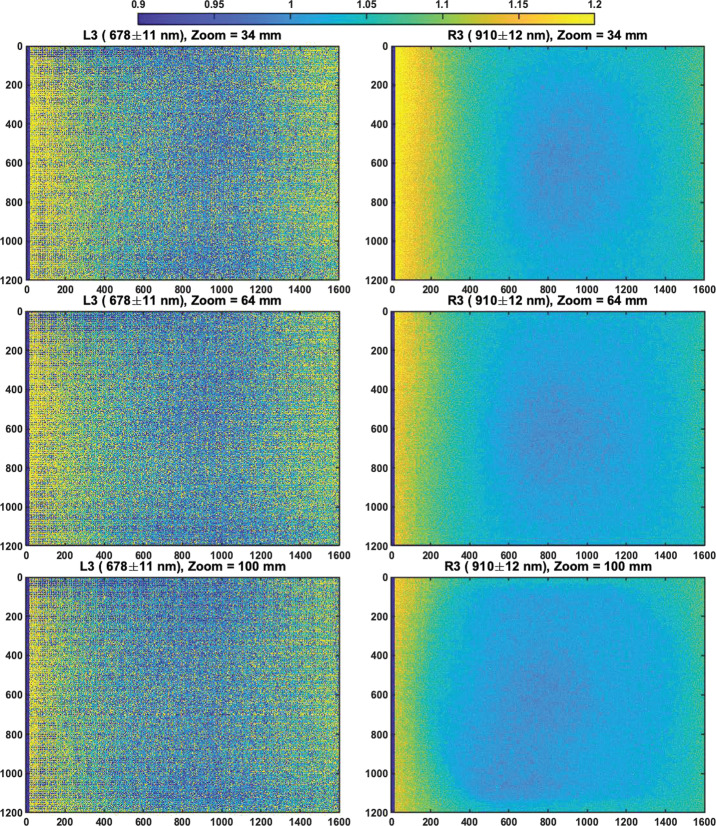
Flatfield correction maps for Filters L3/R3 at 34 mm, 63 mm, and 100 mm focal lengths. See Sect. 3.6 for details

**Fig. 52:**
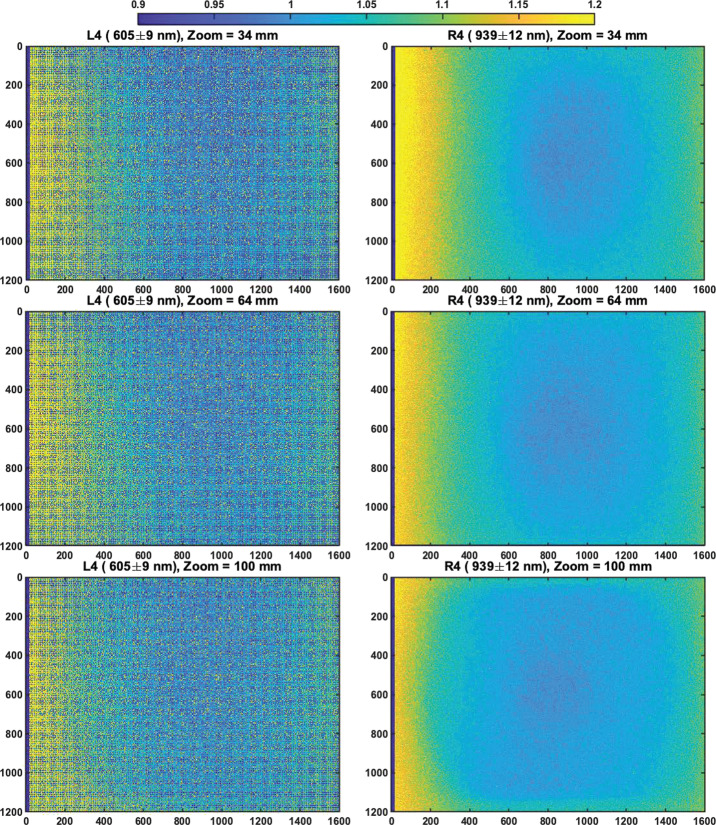
Flatfield correction maps for Filters L4/R4 at 34 mm, 63 mm, and 100 mm focal lengths. See Sect. 3.6 for details

**Fig. 53:**
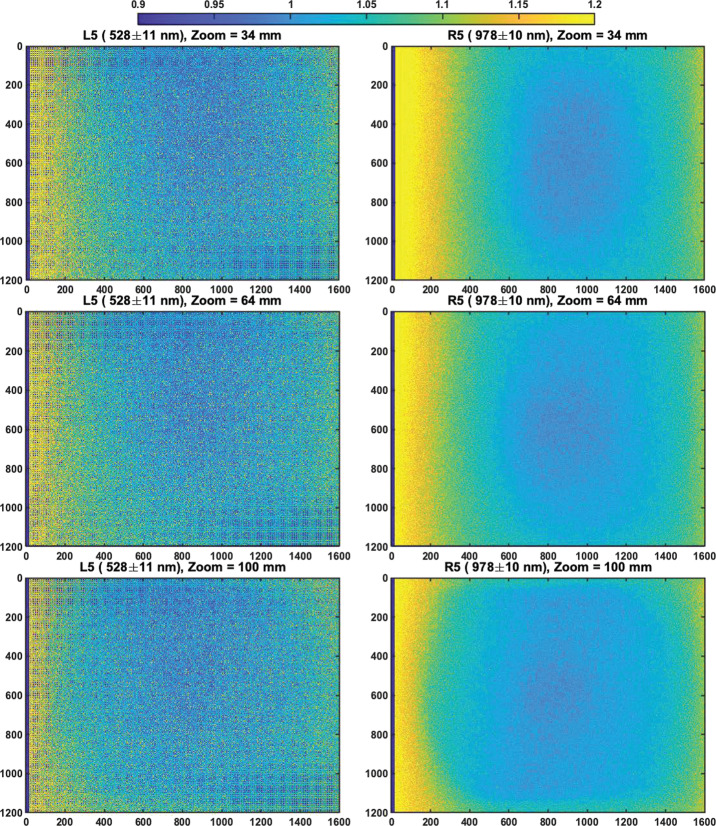
Flatfield correction maps for Filters L5/R5 at 34 mm, 63 mm, and 100 mm focal lengths. See Sect. 3.6 for details

**Fig. 54:**
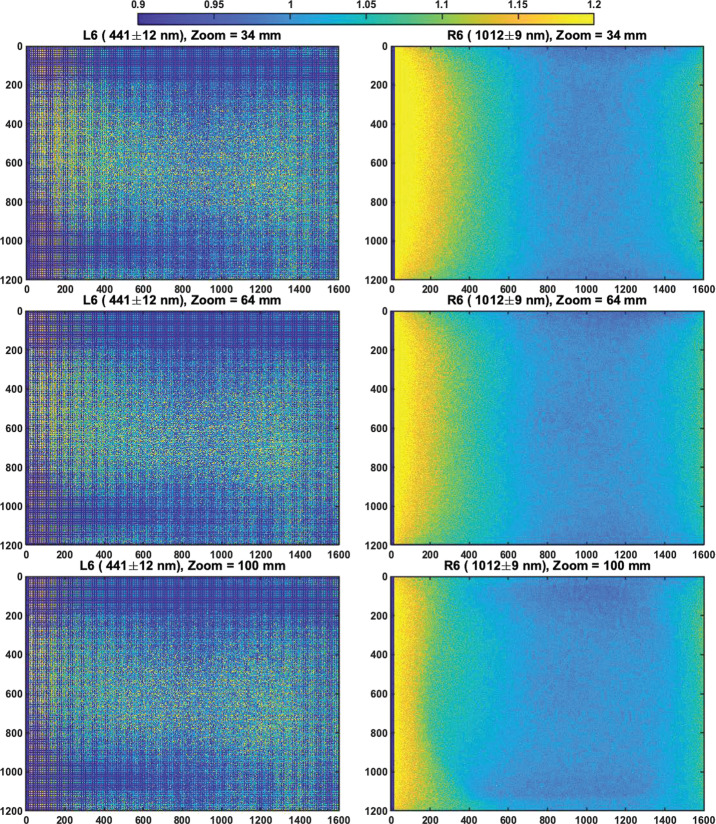
Flatfield correction maps for Filters L6/R6 at 34 mm, 63 mm, and 100 mm focal lengths. See Sect. 3.6 for details

The calibration plan, as-run procedures, image logs, and radiometric calibration analysis scripts used for stand-alone calibration are provided in the Supplementary Online Material for this manuscript. Files containing the spectral response curves for each filter ($r_{\lambda },k$, see Sect. 3.5) are also contained in the SOM. Finally, and perhaps most importantly, we would like to emphasize that the results presented herein were only made possible through the substantial efforts of a large number of students, scientists, and engineers from the Mastcam-Z team, who all came together to participate in pre-flight calibration activities (Fig. 42).

### Supplementary Information

Below are the links to the electronic supplementary material.

(ZIP 98.6 MB)

(RAR 99 kB)

(RAR 44 kB)

## Figures and Tables

**Fig. 44:**
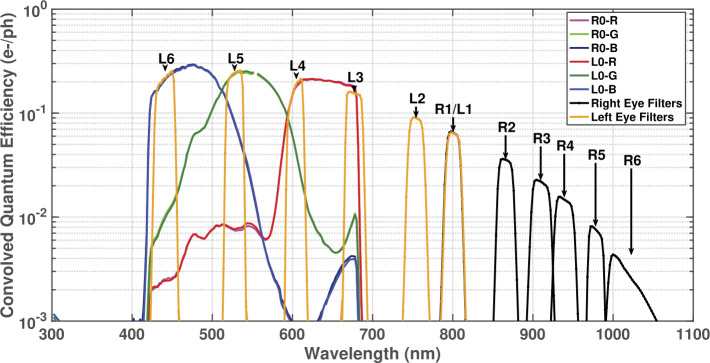
Mastcam-Z Spectral Response ($r_{\lambda ,k}$) for filters L0-L6 and R0-R6 in logarithmic space at a detector temperature of $-5^{\circ }~\mathrm{C}$. For the purposes of this plot, only the dominant RGB Bayer response is shown any given filter, except L0/R0

## Appendix A: Additional Radiometric Details

### A.1 Radiometric Coefficients at Ambient

**Table 12:** Mastcam-Z radiometric calibration coefficients ($r_{o,k}$)^a^ at $+25^{\circ}$ C

Filter	Effective wavelength ±HWHM (nm)	Red		Green		Blue	
		$r_{o,k}$ $[\frac{e^{-}}{ph}]$	*β* [10^−3^/C]	$r_{o,k}$ $[\frac{e^{-}}{ph}]$	*β* [10^−3^/C]	$r_{o,k}$ $[\frac{e^{-}}{ph}]$	*β* [10^−3^/C]
L0	530±90	0.217±0.0063	1.08±0.05	0.25±0.0068	0.18±0.06	0.276±0.0066	0±0.1
L1	800±9	0.0754±0.002	2.24±0.05	0.0497±0.0013	1.86±0.05	0.0447±0.0012	0±0.06
L2	754±10	0.103±0.0013	1.83±0.05	0.0329±0.0012	1.68±0.07	0.00104±0.0012	0±0.05
L3	677±11	0.174±0.00088	1.76±0.05	0.0118±0.00085	0.18±0.05	0.00304±0.00084	0±0.05
L4	605±9	0.226±0.00063	0.75±0.05	0.0687±0.00063	2.57±0.05	0.00218±0.00063	0±0.05
L5	528±11	0.0114±0.00039	0.54±0.05	0.267±0.00039	0±0.05	0.106±0.00039	1.28±0.05
L6	442±12	0.00515±0.00019	0±0.05	0.0183±0.00019	0±0.05	0.253±0.00019	−1±0.5
L7	590±88	-±-	0.85±0.10	-±-	0.89±0.50	-±-	0.85±0.50
R0	530±90	0.211±0.0061	1.08±0.05	0.237±0.0068	0.18±0.06	0.272±0.0066	0±0.1
R1	800±9	0.0757±0.0017	2.24±0.05	0.0489±0.0011	1.86±0.05	0.0441±0.0011	0±0.06
R2	866±10	0.0422±0.0025	3.01±0.05	0.0406±0.00085	2.88±0.05	0.0407±0.00023	2.96±0.05
R3	910±12	0.0274±0.0033	3.56±0.05	0.0271±3.2e-05	3.51±0.05	0.027±9e-05	3.53±0.05
R4	939±12	0.0189±0.0055	4.19±0.05	0.019±0.0013	4.2±0.05	0.0189±0.0004	4.18±0.05
R5	978±10	0.0105±0.00038	5.56±0.05	0.0107±0.0055	5.45±0.05	0.0104±0.0024	5.46±0.05
R6	1022±19	0.00594±0.00058	7.43±0.05	0.00609±0.0007	7.47±0.05	0.00599±0.006	7.45±0.05
R7	880±10	-±-	3.40±0.50	-±-	3.20±0.50	-±-	3.30±0.50

^a^The $r_{o,k}$ reported here are the peak response, which must be multiplied by the peak normalized $\bar{r_{\lambda ,k}}$ to obtain $r_{\lambda ,k}$

^b^These *β* coefficients are set to zero because of large relative uncertainties

**Table 13:** Mastcam-Z radiometric calibration coefficients for 34 mm and 100 mm focal lengths in units of (W/m^2^/nm/sr)/(DN/s) at $+25^{\circ}$ temperature. Note that these results can be scaled to other focal lengths by scaling for changes to optical throughput ($A_{o}\varOmega _{l}$, see Sect. 3.4)

Filter number	Effective center wavelength (nm)	Red	Green	Blue
		[(W/m^2^/nm/sr)/(DN/s)] ±1*σ* uncertainty		
*34 mm zoom*				
L0	530	4.69e-07±2.31e-08	4.52e-07±2.15e-08	4.97e-07±2.3e-08
L1	800	4.98e-06±1.7e-07	7.56e-06±2.63e-07	1.02e-05±3.63e-07
L2	754	3.64e-06±1.5e-07	1.19e-05±5.01e-07	0.000278±5.5e-06
L3	677	2.26e-06±5.61e-08	4.57e-05±1.22e-06	0.000137±1.02e-05
L4	605	2.44e-06±8.2e-08	9.77e-06±3.38e-07	0.000445±3.51e-05
L5	528	4.24e-05±1.71e-06	1.98e-06±5.1e-08	6.46e-06±1.62e-07
L6	442	8.99e-05±5.21e-06	3.6e-05±1.54e-06	2.32e-06±5.99e-08
L7	590	-±-	-±-	-±-
R0	530	4.69e-07±1.67e-08	4.61e-07±1.58e-08	4.98e-07±1.57e-08
R1	800	4.86e-06±1.19e-07	7.43e-06±1.86e-07	1.02e-05±2.56e-07
R2	866	7.42e-06±1.51e-07	7.67e-06±1.57e-07	7.64e-06±1.53e-07
R3	910	9.2e-06±2.11e-07	9.32e-06±2.18e-07	9.36e-06±2.18e-07
R4	939	1.26e-05±2.4e-07	1.26e-05±2.36e-07	1.27e-05±2.33e-07
R5	978	2.82e-05±7.02e-07	2.8e-05±7e-07	2.78e-05±6.93e-07
R6	1022	2.02e-05±3.75e-07	2.02e-05±3.81e-07	2.02e-05±3.9e-07
R7	880	-±-	-±-	-±-
*100 mm zoom*				
L0	530	4.69e-07±2.31e-08	4.52e-07±2.15e-08	4.97e-07±2.3e-08
L1	800	4.98e-06±1.7e-07	7.56e-06±2.63e-07	1.02e-05±3.63e-07
L2	754	3.64e-06±1.5e-07	1.19e-05±5.01e-07	0.000278±5.5e-06
L3	677	2.26e-06±5.61e-08	4.57e-05±1.22e-06	0.000137±1.02e-05
L4	605	2.44e-06±8.2e-08	9.77e-06±3.38e-07	0.000445±3.51e-05
L5	528	4.24e-05±1.71e-06	1.98e-06±5.1e-08	6.46e-06±1.62e-07
L6	442	8.99e-05±5.21e-06	3.6e-05±1.54e-06	2.32e-06±5.99e-08
L7	590	-±-	-±-	−±
R0	530	4.69e-07±1.67e-08	4.61e-07±1.58e-08	4.98e-07±1.57e-08
R1	800	4.86e-06±1.19e-07	7.43e-06±1.86e-07	1.02e-05±2.56e-07
R2	866	7.42e-06±1.51e-07	7.67e-06±1.57e-07	7.64e-06±1.53e-07
R3	910	9.2e-06±2.11e-07	9.32e-06±2.18e-07	9.36e-06±2.18e-07
R4	939	1.26e-05±2.4e-07	1.26e-05±2.36e-07	1.27e-05±2.33e-07
R5	978	2.82e-05±7.02e-07	2.8e-05±7e-07	2.78e-05±6.93e-07
R6	1022	2.02e-05±3.75e-07	2.02e-05±3.81e-07	2.02e-05±3.9e-07
R7	880	-±-	-±-	-±-

^a^The coefficients reported here are subject to the same temperature dependence and *β* parameters as those presented in Table 6

### A.2 TVAC Window Transmission

**Table 14:** Correction factors to account for TVAC chamber window transmission, calculated from observations of a 116-patch Macbeth Color Target imaged at uniform illumination both with and without the TVAC chamber window in place on May 1$^{st}$, 2019 (see Table 3)

Filter number	Effective center wavelength (nm)	Red	Green	Blue
		Effective window transmission ±1*σ* uncertainty		
L0	530	0.941±0.0072	0.948±0.0077	0.946±0.007
L1	802	0.92±0.0049	0.92±0.0053	0.921±0.0056
L2	754	0.921±0.0052	0.923±0.0056	0.922±0.0053
L3	678	0.926±0.0053	0.948±0.006	0.937±0.0052
L4	605	0.938±0.008	0.938±0.0073	0.938±0.0076
L5	528	0.937±0.0038	0.937±0.0039	0.938±0.0039
L6	441	0.938±0.0087	0.938±0.0087	0.938±0.0087
L7	590	-±-	-±-	-±-
R0	530	0.942±0.0049	0.949±0.007	0.953±0.013
R1	802	0.919±0.0045	0.921±0.0055	0.921±0.0053
R2	866	0.907±0.0049	0.907±0.0045	0.907±0.0052
R3	910	0.898±0.005	0.899±0.0037	0.899±0.0041
R4	939	0.901±0.0035	0.901±0.0037	0.903±0.0053
R5	978	0.898±0.0041	0.898±0.0035	0.9±0.0048
R6	1012	0.898±0.0045	0.898±0.0046	0.899±0.0051
R7	880	-±-	-±-	-±-

### A.6 Overview of Radiometric Parameters

**Table:**

Symbol	Definition
*i*	pixel row in image
*j*	pixel column in image
*k*	filter number from 0 to 7
*l*	zoom position or focal length
*f*	focus position
${mc}_{l}$	zoom position in motor-counts [mc]
${mc}_{f}$	focus position in motor-counts [mc]
${FL}_{l}$	zoom focal length [mm]
${FD}_{lf}$	distance to target for best-focus [m]
*t*	exposure time in seconds [s]
${t}_{e}$	extra exposure time due to smear and residual charge [s]
*DN*	12-bit digital-number value
*DN*0	12-bit digital-number value with *t* = 0
${DN}_{8}$	8-bit digital-number value after companding
*D*	dark rate due to dark current [DN/s]
*N*	zero-mean noise of the pixel [DN]
*T*	temperature of the focal-plane assembly [^∘^ C]
${T}_{\mathit{amb}}$	temperature at ambient calibration [^∘^ C]
${A}_{0}$	light collection area of camera [$\mathrm{m}^{2}$]
*Ω*	pixel solid-angle at the center of the array [sr]
*g*	gain as electrons per digital-number [$\frac{e^{-}}{DN}$]
*λ*	wavelength of incident light [nm]
${F}_{\mathit{ijkl}}$	flat field to correct for vignetting [unitless]
${r}_{\mathit{ijk}}(\lambda )$	bandpass efficiency [$e^{-}/ph$]
${\bar{r}_{\mathit{ijk}}(\lambda )}$	integral normalized bandpass efficiency [unitless]
${r}_{0,\mathit{ijk}}$	radiometric coefficient [$e^{-}/ph$,nm]
$\beta _{\mathit{ijk}}$	radiometric temperature coefficient [1/^∘^ C]

## Appendix B: Additional Geometric Details

**Table:**

Symbol	Definition
*i*	pixel row in image
*j*	pixel column in image
${u}_{j}$	pixel column or “x” coordinate
${v}_{i}$	pixel row or “y” coordinate
***u***	point in the camera’s pixel reference frame
$\boldsymbol{x}_{c}$	point in the camera centered reference frame
$\boldsymbol{x}_{w}$	point in the world or rover reference frame
*k*	filter number from 0 to 7
*l*	zoom position or focal length
*f*	focus position
${f}_{l,\mathit{ref}}$	zoom’s reference focus position
${mc}_{l}$	zoom position in motor-counts [mc]
${mc}_{f}$	focus position in motor-counts [mc]
${FL}_{l}$	zoom focal length [mm]
${FD}_{\mathit{lkf}}$	distance to target for best-focus [m]
$\boldsymbol{K}_{l}$	Zoom’s Intrinsic matrix for $f_{\mathit{ref}}$ and *k* = 0
$\boldsymbol{M}_{\mathit{lkf}}$	Affine transformation matrix to $f_{\mathit{ref}}$ and *k* = 0

## Appendix C: Mini-Header Example

The instrument calibration data taken in April and May of 2019 are saved as PNG images with header information encoded in the files. These mini-headers contain the values listed in the table below. The ALTO and flight images have similar information saved in the “MINI_HEADER” group of the headers.

**Table:**

Name	Example^a^	Description
filt	‘0’	Filter number 0-7
vflush	‘2047’	Num. charge-clearing vertical transfers before exposure
focus	‘2460’	Focus motor-count number
autoexposure	‘0’	Auto-exposure parameter (zero if not auto-exposed)
subsec	‘39’	Spacecraft clock fractional 1/256th of a second
compression[0]	‘0’	Companding table number
id	‘1’	GSE-assigned image ID
serno	‘173032’	System serial number
mech[1]	‘0’	Filter motor-count number (divisible by −294)
note^b^	‘ISOP = 1.0079’	Arbitrary test note^b^
initsize	‘3955264’	Initially allocated size of image in bytes
autofocus	‘0’	Auto-focus parameters (zero if not auto-focused)
status	‘1’	Instrument status
compression[1]	‘255’	Maximum pixel value (zero if not companded)
exposure	‘0.0’	Exposure time in milliseconds (divisible by 0.1)
sy	‘0’	Y-vertical start pixel (divisible by 16)
sx	‘0’	X-horizontal start pixel (divisible by 16)
sclk	‘610037823’	Spacecraft clock in epoch J2000.0 at start of exposure
dcoffset	‘110’	DC-offset pixel value subtracted from frame before companding
filter	‘0’	Filter number 0-7 (redundant with “filt”)
mech[2]	‘8652’	Zoom motor-count number (divisible by 6)
mech[0]	‘2460’	Focus motor-count number (divisible by 6, redundant with “focus”)
mode^c^	‘9’	Camerahead mode used to read-out image
side	‘0’	Not used

^a^This example mini-header comes from the image “412TAMBR01_0001.png” in the “/cal1” folder

^b^The test “note” holds information like the integrating sphere out-put (ISOP) in $\mathrm{mW}/\mathrm{cm}^{2}sr\upmu \text{m}$, the monochromator wavelength in nanometers (e.g. “WAVELENGTH = 500”), whether the cameras are looking through the TVAC window (e.g. “WINDOW = 1”), the calibration target name (e.g. “DOT40”, “MTF_SN007”, etc.), or the metrology measurement id (e.g. “METROLOGY = 001”)

^c^The “mode” parameter controls the camerahead clock speed and the video exposure mode, among other things. Mode 9 commands the clock to half-speed (20 MHz), while Mode 0 commands the clock to full speed (40 MHz)
