# Supplementary material for: Pre-Flight Calibration of the Mars 2020 Rover Mastcam Zoom (Mastcam-Z) Multispectral, Stereoscopic Imager
Source: Space Sci Rev. 2021 Feb 18;217(2):29. doi: 10.1007/s11214-021-00795-x (PMC7892537; doi:10.1007/s11214-021-00795-x)
Supplement: Supplementary file 1 — (ZIP 98.6 MB) [file 11214_2021_795_MOESM1_ESM.zip › As_Run_Log_Document.docx]

**FM Mastcam-Z As-Run Procedure Log**

| **Test Description** | **Filename and Version** | **Date and Time** | **Shifts** | **Calibration Team** |
| --- | --- | --- | --- | --- |
| V&V Ramps imaging the JR dot target from -40C to +60C |  | Thursday - Friday, April 26-27, 2019 (8 hours) |  | Mike Caplinger, Elsa Jensen, Tex Kubacki, Paul Corlies, Christian Tate, Andy Winhold |
| Dark current at -10C  Practice MTF placement test | [CalPro 441 Dark Current v2 01.docx](https://lona.sese.asu.edu/mastcam-z/File:CalPro_441_Dark_Current_v2_01.docx)  [CalPro 473 MTF v2 01.docx](https://lona.sese.asu.edu/mastcam-z/File:CalPro_473_MTF_v2_01.docx) | Friday, April 26, 2019 19:00-20:00 (1 hour)  20:00-22:00 (2 hours) | 0.5 | Jim Bell, Elsa Jensen, Tex Kubacki, Paul Corlies, Christian Tate, Andy Winhold |
| MTF Tests for 34mm, 63mm, and 100mm at -10C  Radiometric Flat Fields at -10C plus some photon transfer curves  Solar filter Radiometric Flat Fields at -10C | [CalPro 473 MTF v2 02.docx](https://lona.sese.asu.edu/mastcam-z/File:CalPro_473_MTF_v2_02.docx)  [CalPro 425 Radiometric v2 03.docx](https://lona.sese.asu.edu/mastcam-z/File:CalPro_425_Radiometric_v2_03.docx)  [CalPro 426 Radiometric v2 03.docx](https://lona.sese.asu.edu/mastcam-z/File:CalPro_426_Radiometric_v2_03.docx) | Saturday. April 27, 2019 8:45-13:20 (5 hours)  14:00-20:00 (6 hours)  18:15-20:45 (3 hours) | 1.5 | Jim Bell, Elsa Jensen, Tex Kubacki, Paul Corlies, Christian Tate, Megan Barrington, Andy Winhold |
| JR Cal. at -10C, 100+ positions at 48mm (Scene 0), fixed target tests (Scenes 1-6) | [CalPro 465-7 JR Geometric v2 04 FL48 TEMP.docx](https://lona.sese.asu.edu/mastcam-z/File:CalPro_465-7_JR_Geometric_v2_04_FL48_TEMP.docx) | Sunday, April 28, 2019 8:30-16:10 (8 hours) | 1.5 | Jim Bell, Elsa Jensen, Tex Kubacki, Paul Corlies, Megan Barrington, Christian Tate, Andy Winhold |
| JR Cal. Frames for -10C to +42C Ramp  Radiometric Flat Fields through the window at +30C plus some photon transfer curves and dark current before and after | [CalPro 468 JR Geometric v2 04.docx](https://lona.sese.asu.edu/mastcam-z/File:CalPro_468_JR_Geometric_v2_04.docx)  [CalPro 423 Radiometric v2 04.docx](https://lona.sese.asu.edu/mastcam-z/File:CalPro_423_Radiometric_v2_04.docx) | Monday, April 29, 2019 10:00-13:30 (4 hours)  18:00-23:00 (5 hours) | 2 | Justin Maki, Ken Herkenhoff, Elsa Jensen, Tex Kubacki, Paul Corlies, Christian Tate, Andy Winhold |
| MTF Tests for 34mm, 63mm, and 100mm at +28C | [CalPro 471 MTF v2 06.docx](https://lona.sese.asu.edu/mastcam-z/File:CalPro_471_MTF_v2_06.docx) | Tuesday, April 30, 2019 8:30-15:00 (7 hours) | 1.5 | Justin Maki, Elsa Jensen, Tex Kubacki, Paul Corlies, Christian Tate, Andy Winhold |
| JR Cal. at +25C, 100+ positions at 48mm (Scene 0) | [CalPro 465-7 JR Geometric v2 06 TVAC Ambient.docx](https://lona.sese.asu.edu/mastcam-z/File:CalPro_465-7_JR_Geometric_v2_06_TVAC_Ambient.docx) | Tuesday, April 30, 2019 15:00-18:30 (4 hours) | 1 | Ken Herkenhoff, Jason van Beek, Tex Kubacki, Paul Corlies, Christian Tate, Andy Winhold, Mason Starr |
| JR Cal. at +25C, fixed target tests (Scene 7 window on, Scene 9 door open) plus Macbeth imaging in non-solar filters (Scene 8 window on, Scene 10 door open)  FMs Move to MSSS Cleanroom | [CalPro 466 JR Geometric v2 07 TVAC Ambient.docx](https://lona.sese.asu.edu/mastcam-z/File:CalPro_466_JR_Geometric_v2_07_TVAC_Ambient.docx) | Wednesday, May 1, 2019 8:30-11:30 (3 hours)  12:00-15:00 (3 hours) | 1 | Justin Maki, Mike Caplinger, Elsa Jensen, Tex Kubacki, Paul Corlies, Ole Jensen, Christian Tate, Andy Winhold, |
| Photon Transfer and Dark Current for the Right MCZ FM1 for two Radiance Values | [CalPro 412 Photon v2 03.docx](https://lona.sese.asu.edu/mastcam-z/File:CalPro_412_Photon_v2_03.docx) | Wednesday, May 1, 2019, 18:00-22:00 (3 hours) | 1 | Ken Herkenhoff, Jason van Beek, Tex Kubacki, Paul Corlies, Christian Tate, Andy Winhold, Mason Starr, Megan Barrington |
| JPL Geometric Calibration for 26mm, 34mm, 48mm, and 63mm for both MCZs | [CalPro 462 Geometric v2 02.docx](https://lona.sese.asu.edu/mastcam-z/File:CalPro_462_Geometric_v2_02.docx) (first half) | Thursday, May 2, 2019, 8:30-17:00 (9 hours) | 1 | Justin Maki, Mark Thompson, Mike Caplinger, Elsa Jensen, Tex Kubacki, Paul Corlies, Ole Jensen, Christian Tate, Andy Winhold, Jason van Beek, Jesse Kuik, Evan Stanish |
| Photon Transfer and Dark Current for the Left and Right MCZs | [CalPro 412 Photon v2 05.docx](https://lona.sese.asu.edu/mastcam-z/File:CalPro_412_Photon_v2_05.docx)  [CalPro 412 Photon v2 06.docx](https://lona.sese.asu.edu/mastcam-z/File:CalPro_412_Photon_v2_06.docx) | Thursday, May 2, 2019,  17:45*-23:30 (6 hours) | 1 | Ken Herkenhoff, Jason van Beek, Katherine Winchell, Darian Dixom, Paul Corlies, Christian Tate, Andy Winhold, Megan Barrington, Emily Lakdawalla, Mason Starr |
| JPL Geometric Calibration for 79mm, 100mm, and 110mm for both MCZs as well as several JR scripts  JR Target with Metrology determining absolute scale | [CalPro 462 Geometric v2 02.docx](https://lona.sese.asu.edu/mastcam-z/File:CalPro_462_Geometric_v2_02.docx) (second half)  [CalPro 465-7 JR Geometric v2 09.docx](https://lona.sese.asu.edu/mastcam-z/File:CalPro_465-7_JR_Geometric_v2_09.docx) | Friday, May 3, 2019  8:00-14:00 (8 hours)  14:00-16:00 (2 hours) | 1 | Justin Maki, Mark Thompson, Jason van Beek, Elsa Jensen, Tex Kubacki, Katherine Winchell, Paul Corlies, Ole Jensen, Jesse Kuik, Evan Stanish, Gerhard Paar |
| MTF and Depth of Field targets | [CalPro 471 MTF v2 08.docx](https://lona.sese.asu.edu/mastcam-z/File:CalPro_471_MTF_v2_08.docx) | Friday, May 3, 2019  17:00-23:00 (6 hours) | 1 | Ken Herkenhoff, Jason van Beek, Katherine Winchell, Darian Dixom, Paul Corlies, Christian Tate, Andy Winhold, Megan Barrington, Emily Lakdawalla, Mason Starr, Tina |
| MTF Targets | [CalPro 471 MTF v2 08.docx](https://lona.sese.asu.edu/mastcam-z/File:CalPro_471_MTF_v2_08.docx) | Saturday, May 4, 2019  8:00-12:00 (4 hours) | 1 | Alex Hayes, Tex Kubacki, Paul Corlies, Katherine Winchell, Christian Tate, Evan Stanish, Ernest Cisneros, Melissa Rice, Gerhard Paar |
| JR Geometric Calibration at Ambient | [CalPro 465-7 JR Geometric v2 11.docx](https://lona.sese.asu.edu/mastcam-z/File:CalPro_465-7_JR_Geometric_v2_11.docx) | Saturday, May 4, 2019  14:00-24:00 (10 hours) | 1.5 | Alex Hayes, Ken Herkenhoff, Gerhard Paar, Jason van Beek, Darian Dixom, Paul Corlies, Christian Tate, Evan Stanish, Ernest Cisneros, Melissa Rice, Nathalie Turenne, Alexis Parkinson, Tina Seeger, Megan Barrington |
| Spectral Throughput Right at Ambient | [CalPro 433 434 R Spectral Throughput v2 02.docx](https://lona.sese.asu.edu/mastcam-z/File:CalPro_433_434_R_Spectral_Throughput_v2_02.docx) | Sunday, May 5, 2019  8:00-24:00 (16 hours) | 2 | Melissa Rice, Alex Hayes, Jim Bell, Katherine Winchell, Tex Kubacki, Paul Corlies, Ole Jensen, Christian Tate, Ernest Cisneros, Nathalie Turenne, Alexis Parkinson, Tina Seeger, Evan Stanish, Megan Barrington |
| Radiometric Flat Fields at Ambient | [CalPro 423 Radiometric v2 07.docx](https://lona.sese.asu.edu/mastcam-z/File:CalPro_423_Radiometric_v2_07.docx) | Monday, May 6, 2019  8:00-16:00 (8 hours) | 1 | Mike Caplinger, Mel Rice, Briony Horgan, Alex Hayes, Jim Bell, Tex Kubacki, Paul Corlies, Ole Jensen, Christian Tate, Alexis Parkinson, Tina Seeger, Megan Barrington |
| Spectral Throughput Left at Ambient | [CalPro 433 434 L Spectral Throughput v2 02.docx](https://lona.sese.asu.edu/mastcam-z/File:CalPro_433_434_L_Spectral_Throughput_v2_02.docx) | Monday, May 6, 2019  16:00-24:00 (8 hours) | 1 | Mel Rice, Alex Hayes, Jim Bell, Tex Kubacki, Paul Corlies, Ole Jensen, Christian Tate, Alexis Parkinson, Tina Seeger, Megan Barrington |
| Spectral Throughput Left at Ambient Second Try | [CalPro 433 434 L Spectral Throughput v2 03.docx](https://lona.sese.asu.edu/mastcam-z/File:CalPro_433_434_L_Spectral_Throughput_v2_03.docx) | Tuesday, May 7, 2019  7:30-11:30 (4 hours) | 0.5 | Briony Horgan, Alex Hayes, Jim Bell, Elsa Jensen, Chis Donaldson, Tex Kubacki, Paul Corlies, Ole Jensen, Christian Tate, Alexis Parkinson |
| Stray-light Testing | [CalPro 481 Stray Light v2 02.docx](https://lona.sese.asu.edu/mastcam-z/File:CalPro_481_Stray_Light_v2_02.docx) | Tuesday, May 7, 2019  12:30-16:30 (6 hours) | 0.5 | Alex Hayes, Jim Bell, Briony Horgan, Tex Kubacki, Paul Corlies, Ole Jensen, Christian Tate, Alexis Parkinson, Megan Barrington |
| MTF and Star Targets | [CalPro 471 MTF v2 12.docx](https://lona.sese.asu.edu/mastcam-z/File:CalPro_471_MTF_v2_12.docx) | Tuesday, May 7, 2019  17:30-22:30 (5 hours) | 0.5 | Jeff Johnson, Alex Hayes, Jim Bell, Tex Kubacki, Paul Corlies, Ole Jensen, Angela Magee, Christian Tate, Nathalie Turenne, Megan Barrington |
| JR Geometric Calibration at Ambient Intrinsic for 100mm and 26mm | [CalPro 465-7 JR Geometric v2 13.docx](https://lona.sese.asu.edu/mastcam-z/File:CalPro_465-7_JR_Geometric_v2_13.docx) | Tuesday, May 7, 2019  23:20-01:00 (2 hours) | 0.5 | Jeff Johnson, Alex Hayes, Jim Bell, Angela Magee, Jason Van Beek, Paul Corlies, Ole Jensen, Christian Tate, Nathalie Turenne |
| Geoboard and EPO Imaging | [CalPro 491 Sample Observations v2 03.docx](https://lona.sese.asu.edu/mastcam-z/File:CalPro_491_Sample_Observations_v2_03.docx) | Wednesday, May 8, 2019  8:00-15:30 (8 hours) | 1 | Mike Caplinger, Briony Horgan, Jeff Johnson, Alex Hayes, Jim Bell, Justin Maki, Elsa Jensen, Chis Donaldson, Angela Magee, Tex Kubacki, Paul Corlies, Ole Jensen, Christian Tate, Alexis Parkinson, Megan Barrington |

* Near term schedule

*** First time run and uncertain duration estimate

Updated: Mid-day Wednesday, May 1, 2019

Testing accomplished: 57 hours

Testing scheduled: 100 hours

**Percent completed: 36%**

Updated: End-of-day Thursday, May 2, 2019

Testing accomplished: 75 hours

Testing scheduled: 80 hours

**Percent completed: 48%**

Updated: Mid-Day Friday, May 3, 2019

Testing accomplished: 85 hours

Testing scheduled: 74 hours

**Percent completed: 53%**

Updated: End-of-Day Saturday, May 4, 2019

Testing accomplished: 107 hours

Testing scheduled: 52 hours

**Percent completed: 67%**

Updated: Mid-Day Sunday, May 5, 2019

Testing accomplished: 122 hours

Testing scheduled: 47 hours

**Percent completed: 72%**

Updated: Mid-Day Monday, May 6, 2019

Testing accomplished: 130 hours

Testing scheduled: 32 hours

**Percent completed: 80%**

Updated: Mid-Day Tuesday, May 7, 2019

Testing accomplished: 149 hours

Testing scheduled: 18 hours

**Percent completed: 90%**

Updated: Mid-Day Wednesday, May 7, 2019

Testing accomplished: 164 hours

**Percent completed: 100%**

**ATLO Wishlist**

- **Pointing cross-calibration**
- **RGB cross-calibration to Mastcam-Z**

**EQM Wishlist**

- **Off axis spectral throughput shits**
- **Sutter smear**
