# Supplementary material for: Pre-Flight Calibration of the Mars 2020 Rover Mastcam Zoom (Mastcam-Z) Multispectral, Stereoscopic Imager
Source: Space Sci Rev. 2021 Feb 18;217(2):29. doi: 10.1007/s11214-021-00795-x (PMC7892537; doi:10.1007/s11214-021-00795-x)
Supplement: Supplementary file 1 — (ZIP 98.6 MB) [file 11214_2021_795_MOESM1_ESM.zip › CalPro_412_L_Photon_v2_05.pdf]

Date 5/2 Time 17:47 Initials [illegible]

Photon Transfer Procedure for Left Mastcam-Z Ambient Testing at MSSS (Pro. 4.1.2)

[Procedure version 2.05, prepared by the Mastcam-Z calibration team at Cornell University]

These measurements are performed on the camera and at the temperature designated below as specified in the Mastcam-Z Calibration Plan,

Unit Under Test:

Left FM __X__ **Right FM** ______ **EQM** _____ **Other** ____________

These measurements are performed at temperature:

-35° C ______ **-10°C** ______ **+5°C** ______ **Ambient** __X__ **Other** ________

These measurements are performed at:

MSSS __X__ **ASU** ______ **Other** ________

Date 5/2/19 **Start Time** 17:50 **End Time** 21:00

Estimated Duration __5.0 hours__

Scheduled Start Time 16:00 **Sch. End Time** 23:59

Calibration Lead [L] KEN HERKENHOFF **Documentarian [D]** EMILY, MEGAN, JON

Camera Operator [[redacted]] JASON, KW, DD **Technician [T]** CHRISTIAN Tate

Data Validator [V] MASON Starr **Other** ____________

Date 5/2 Time 17:50 Initials [illegible]

Change Log

Version	Name	Change
v1_01<br>4 June 2018	C. Tate	(first draft)
v1_27<br>30 Oct. 2018	C. Tate	Procedure edits prior to EQM testing
v1_29<br>1 Dec. 2018	C. Tate	Procedure edits after EQM testing
v2_04<br>2 May 2019	C. Tate	Updated after running similar procedures
v2_05<br>2 May 2019	K. Herkenhoff	Approved version prior to FM testing

Document Approval

Approved by James Bell Date
Mastcam-Z PI
Arizona State University

[signature] 5/6/19
Approved by Alexander Hayes Date
Mastcam-Z Calibration Working Group Lead, Cornell University

[signature] 5/2/19
Approved by Justin Maki Date
Mastcam-Z Deputy PI and Investigation Scientist, Jet Propulsion Laboratory

[signature] 5/2/19
Approved by: Date
Ken Herkenhoff
Mastcam-Z Co-Investigator, USGS

[signature] 5/3/19
Approved by Christian Tate Date
Procedure Author
Cornell University

Table of Contents

PHOTON TRANSFER PROCEDURE FOR LEFT MASTCAM-Z AMBIENT TESTING AT MSSS (PRO. 4.1.2) **1**

- Change Log 2
- Document Approval 2
- Test Description 4
- Software Preparation 4
  - *Table 1. File naming convention for the camera script prefixes and frame filenames: "AAABBBBCDD"* *4*
- Hardware Installation 6
  - *Figure 1. ASU Floor Plan for Geometric Testing in the TVAC Chamber. The MSSS Floor Plan allows for similar target and source placements relative to the chamber window.* *6*
  - *Table 2. Exposure times in milliseconds for each integrating sphere radiance value in Table 3* *8*
  - *Table 3. The nominal integrating sphere output radiance values.* *8*

MASTCAM-Z TESTS **9**

- Dark Current with the Right and Left Mastcam-Zs 9
- Center the Integrating Sphere on the Left Mastcam-Z 10
- Radiance Value 1 for the Left Mastcam-Z 11
- Radiance Value 2 for the Left Mastcam-Z 12
- Radiance Value 3 for the Left Mastcam-Z 13
- Radiance Value 4 for the Left Mastcam-Z 14
- Radiance Value 5 for the Left Mastcam-Z 15
- Radiance Value 6 for the Left Mastcam-Z 16
- Radiance Value 7 for the Left Mastcam-Z 17
- Radiance Value 8 for the Left Mastcam-Z 18
- Data Validation 19
- Dark Current with the Right and Left Mastcam-Zs 20

SHUTDOWN PROCEDURE **21**

Test Description

Excerpt from the Calibration Plan 4.2,

> The objectives of these tests are to derive flat field images as well as the coefficients to allow a conversion from reduced (bias, dark, and flat field corrected) DN/s to absolute radiometric response (W/cm^2/sr per filter) for (a) the R, G, and B microfilters of the Bayer Pattern Filter detectors in each camera head (clear filter), (b) the 14 non-solar Mastcam-Z spectral filters "Science Filters", and, if time permits, (c) the two Mastcam-Z neutral density solar filters; and to provide an estimate of the uncertainty in these coefficients and, at Priority 2, their temperature dependence. This test builds off the Section 4.3 – Spectral Throughput Calibration to accurately account for the filter spectral response in the conversion. The requirement of knowing the relative response on the shape of the spectral throughput to ±5% combined with the absolute Radiance accuracy of the integration sphere at ±5% still allows the ±10% absolute radiometric calibration requirement to be met.

Software Preparation

The software and files required for this test are prepared well in advance of test day. This checklist ensures that the following are present, debugged, and executable: (1) all fast-look scripts, (2) automated header generation of all relevant camera parameters, target positioning, and metadata, (3) all camera scripts that command the camera unit, and (4) the directories/file-paths pointing to the data repositories of this specific test.

Table 1. File naming convention for the camera script prefixes and frame filenames: "AAABBBBCDD"

Code	Name	Example	Value
"AAA"	Calibration Plan Section	"411" = Cal. Plan 4.1.1 chapter 4, section 1, subsection 1	412
"BBBB"	Location of test or ASU Chamber temperature	"MSSS" = test at MSSS, "TN10" = ASU TVAC -10C, …	TAMB
"C"	Camera unit under test	"L" = Left Mastcam-Z, "R" = Right Mastcam-Z, "E" =EQM, "C" =COTS	L
"DD"	Part of test (radiance value)	"00" = test set up, "01" = first radiance value …	00-08

Date 5/2 Time 17:50 Initial [illegible]

1. **[D]** [illegible] Look up the daily calibration schedule and record the scheduled start and end time of this test on the cover page of this document. Also fill out and double-check the other information on the cover page.
2. **[D]** [illegible] Ensure that all supplemental manuals are on hand. These are,
   - ~~Labsphere_Manual~~,
   - Validator_Manual, Documentarian_Manual
   - MastcamZCalPlan
3. **[D]** [illegible] Ensure that the Image Log is present and ready to use. Find and open the Google Sheets file "Image_Log_42". There is a link on the Wiki.
4. **[V]** [illegible] Check that all Calgorithms fast-look and validation scripts are present, up-to-date, and ready to analyze test output. Find and open the "Radiometric_Calibration_42_Validation" Jupyter notebook. There is a link on the Wiki.
5. **[O]** [illegible] Check that all camera scripts required for this test are present, up-to-date and ready to command the ground support equipment (GSE). These are,
   - **412TAMBL00 - 412TAMBL08** ~~43~~ 423TAMBL00
   - **441TEMPR03, 441TEMPL03**
6. **[O,V,D, L]** Notes:

START DELAYED DUE TO GEOMETRIC TESTING

Hardware Installation

This procedure is for the ambient TVAC chamber testing at MSSS. Figure 1 shows the nominal layout of the TVAC chamber, workspace, Mastcam-Zs, ground support equipment (GSE), targets, sources, and other equipment necessary for this test if it happens at ASU. Although MSSS' cleanroom is different than ASU's, the placement of the targets and sources relative to the chamber window is similar.

Figure 1. ASU Floor Plan for Geometric Testing in the TVAC Chamber. The MSSS Floor Plan allows for similar target and source placements relative to the chamber window.

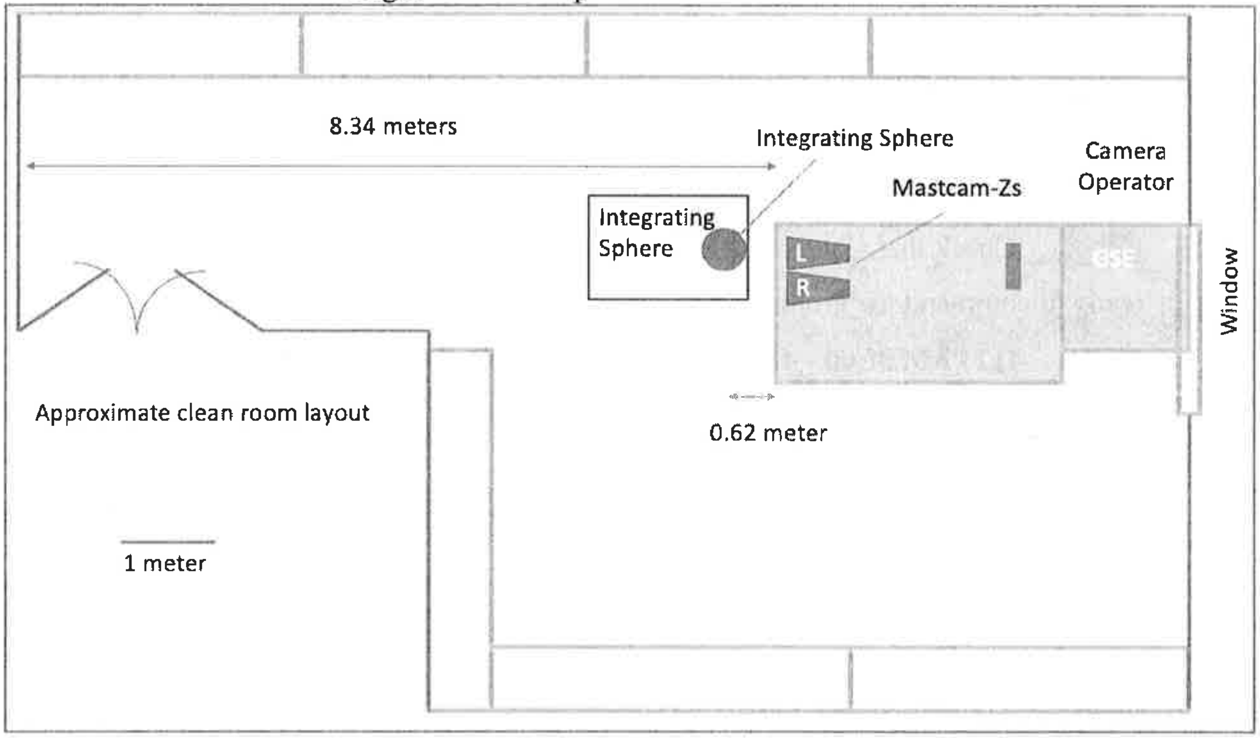

7. [T,O, L] [initialed] Ensure that all personnel in the cleanroom are following the cleanroom practices for electrostatic discharge, proper clothing and other safety concerns.
8. [T] [initialed] Double check that ionized air is flowing over the Mastcam-Zs.
9. [T] [initialed] Verify that the thermocouples are turned on and properly reading out.
10. [O,T] [initialed] Ensure that the camera unit and GSE wires are secure, kink-free, and do not present tripping hazards when the lights are turned off.
11. ~~[O,D] [initialed] Check the camera temperature and ensure nominal operation.~~

Date 5/2 Time 18:19 Initial [illegible]

11. **[O,D]** [illegible] Check the camera temperature and ensure nominal operation.
12. **[D]** [illegible] Record the following environmental information:
    - Cleanroom temperature [illegible] 68°F pressure N/A humidity 66%
13. **[O,D, L]** Notes:

____

____

____

14. **[D,T]** [illegible] Take time-stamped pictures of the integrating sphere and the whole test/GSE set-up.
15. **[T]** [illegible] Power on the integrating sphere. Follow the procedure in "Labsphere_Manual". Record the time the lamp is turned on 18:19. The bulb and radiometer take about 20 minutes to warm up and stabilize, but that can happen while we continue to center the sphere on the Mastcam-Z boresight.
16. **[D,T]** [illegible] Record the exact readout value of the integrating sphere's radiance: 6 mW/cm2/sr.
17. **[T,O, L]** [illegible] Confirm that the camera systems and GSEs are powered on and ready for use.
18. **[D, L]** Notes:

LEFT ON ONLY.

____

____

Date 5/2 Time 18:33 Initial [illegible]

Table 2. Exposure times in milliseconds for each integrating sphere radiance value in Table 3 at **100 mm focal length. The estimated duration is 13 minutes.** (Note that each sequence of exposure times begins and ends with a zero-time exposure in order to evaluate bias evolution.)

	**Time0**	**Time1**	**Time2**	**Time3**	**Time4**	**Time5**	**Time6**	**Time7**	**Time8**
**Radiance 1**	0.0	3.0	4.0	6.0	10.0	22.0	30.0	60.0	80.0
**Radiance 2**	0.0	3.0	4.0	6.0	10.0	14.0	22.0	40.0	80.0
**Radiance 3**	0.0	2.0	3.0	4.0	6.0	10.0	22.0	30.0	40.0
**Radiance 4**	0.0	2.0	3.0	4.0	6.0	10.0	14.0	22.0	30.0
**Radiance 5**	0.0	1.0	1.5	2.0	3.0	6.0	10.0	14.0	22.0
**Radiance 6**	0.0	0.5	1.0	2.0	3.0	4.0	6.0	10.0	14.0
**Radiance 7**	0.0	0.5	1.0	1.5	2.0	3.0	4.0	10.0	14.0
**Radiance 8**	0.0	0.5	1.0	1.5	2.0	2.5	3.0	6.0	10.0

Table 3. The nominal integrating sphere output radiance values.
If the pre-test reveals that a scaling is necessary, then the scaled radiance values are recorded here and used for the remainder of the photon transfer testing. (Note, these radiance values are in units of 1.0-2.4 micron band integrated spectral radiance mW/cm2/sr, true radiance is about a factor of two higher.)

**IS Output Radiances**	**Nominal Radiance [mW/cm2/sr]**	**Scaled Radiance [mW/cm2/sr]**
✓ Radiance 1	1.0	
✓ Radiance 2	2.0	
✓ Radiance 3	3.0	
✓ Radiance 4	4.0	
✓ Radiance 5	5.0	
✓ Radiance 6	6.0	
✓ Radiance 7	8.0	
✓ Radiance 8	10.0	

Date 5/2 Time 19:00 Initial [illegible]

Mastcam-Z Tests

Dark Current with the Right and Left Mastcam-Zs

19. **[T]** [illegible] Turn off the lights and minimize the room's ambient light.
20. **[D]** [illegible] Record the following temperatures:
    - Camera CCD temp 24.[illegible]°C Optics temp N/A

SKIP 21. ~~**[D,T]** [illegible] Take digital pictures of the integrating sphere's position and the~~ whole test/GSE set-up.

22. **[O]** Load and execute camera script **441TEMPL03**, which captures 5 dark frames through filter 7 at the exposure times 0.0, 10.0, 20.0, and 100 seconds. The estimated duration is 12 minutes.
23. **[O]** Load and execute camera script **441TEMPR03**, which captures 5 dark frames through filter 7 at the exposure times 0.0, 10.0, 20.0, and 100 seconds. The estimated duration is 12 minutes.
24. **[D]** [illegible] Record image names and parameters in Image Log.
25. ~~**[T]** ____ Lights on~~. SKIP
26. **[D, L]** Notes: CLOSED WINDOW SHADE.
    DID THIS TEST AFTER ALIGNMENT

Date 5/2 Time 18:38 Initial [illegible]

Center the Integrating Sphere on the Left Mastcam-Z

27. **[T]** [illegible] Move the integrating sphere ~~close to~~ 64cm from Mastcam-Z's boresight.
28. **[T]** [illegible] Remove the integrating sphere's port cover, OPEN SLIT TO RADIANCE 1.
29. **[O]** [illegible] Insert the note "ISOP=[radiance]" and execute camera script **423TAMBL00**. This script captures one auto-exposure at 40% full-well for filter 0 at 79 mm focal length (because we want to center it in 100mm).
30. **[V,O,T]** [illegible] Open images, and if the images show that the integrating sphere is not centered, center the integrating sphere disc in the frame. Recapture **423TAMBL00** frames if necessary.
31. **[D]** [illegible] Record image names and parameters in the image Log.
32. **[T]** [illegible] Take digital pictures of the integrating sphere's position and the whole test/GSE set-up. 20 3/16" HEIGHT ABOVE FLOOR
33. **[T]** [illegible] Visually estimate the distance from the integrating sphere and the Mastcam-Z's sunshade. This distance is approximately 68 cm.
34. **[T]** [illegible] Lights off
35. **[D, L]** Notes:
DID THIS BEFORE DARK CURRENT
STARTED AT 1 mWcm^{-2}sr^{-1}

Date 5/2 Time 19:15 Initial [initials]

Radiance Value 1 for the Left Mastcam-Z

REMOVE PORT COVER

36. **[D]** [initials] Record temperature information:
    - Camera CCD temp 24.0°C Optics temp N/A
37. **[D,T]** [initials] Set the integrating sphere output to this test's radiance value defined in Table 3. ADJUSTED DOWN
38. **[D,T]** [initials] Record exact integrating sphere readout value 1.000[illegible] mW/cm2/sr.

SKIP 39. ~~**[T]** ____ Take time-stamped digital pictures of the setup and integrating sphere readout.~~

40. **[O]** [initials] Insert the note "ISOP=[radiance]" and execute camera script **412TAMBL01**, which captures 10 frames for 9 exposure times with filter 0 at focus 3 m at 100mm focal length. The estimated duration is 10 minutes.
41. **[D,T]** [initials] Record exact integrating sphere readout value 1.0[illegible]03 mW/cm2/sr.
42. **[D]** [initials] Record image names and parameters in the Image Log.
43. **[D, L]** Notes: MOVE TO 110 mm FOCAL LENGTH DELETED FOCAL LENGTH COMMANDS FROM SCRIPTS.

Date 5/2 Time 19:28 Initial Ea

Radiance Value 2 for the Left Mastcam-Z

44. **[D]** Ea Record temperature information:
    - Camera CCD temp 24.0°C Optics temp N/A
45. **[D,T]** Ea Set the integrating sphere output to this test's radiance value defined in Table 3.
46. **[D,T]** Ea Record exact integrating sphere readout value 2.0045 mW/cm2/sr.

SKIP ~~47. **[T]** ____ Take time-stamped~~ digital pictures of the setup and integrating sphere readout.

48. **[O]** Ea Insert the note "ISOP=[radiance]" and execute camera script **412TAMBL02**, which captures 10 frames for 9 exposure times with filter 0 at focus 3 m at ~~100~~ 110 mm focal length. The estimated duration is 10 minutes.
49. **[D,T]** Ea Record exact integrating sphere readout value 2.0[illegible]6 mW/cm2/sr.
50. **[D]** Ea Record image names and parameters in the Image Log.
51. **[D, L]** Notes: ____________________

Date 5/2 Time 20:3[illegible] Initial [illegible]

Radiance Value 3 for the Left Mastcam-Z

52. **[D]** [initials] Record temperature information:
    - Camera CCD temp 23.6°C Optics temp N/A
53. **[D,T]** [initials] Set the integrating sphere output to this test's radiance value defined in Table 3.
54. **[D,T]** [initials] Record exact integrating sphere readout value 3.016 mW/cm2/sr.

SKIP ~~55. **[T]** ____ Take time-stamped digital pic~~tures of the setup and integrating sphere readout.

56. **[O]** [initials] Insert the note "ISOP=[radiance]" and execute camera script **412TAMBL03**, which captures 10 frames for 9 exposure times with filter 0 at focus 3 m at 100mm focal length. The estimated duration is 10 minutes.
57. **[D,T]** [initials] Record exact integrating sphere readout value 3.029 mW/cm2/sr.
58. **[D]** ____ Record image names and parameters in the Image Log.
59. **[D, L]** Notes: →89

Date 5/2 Time 9:41 Initial [initials]

Radiance Value 4 for the Left Mastcam-Z

60. **[D]** [initials] Record temperature information:
    - Camera CCD temp 24.0°C Optics temp N/A
61. **[D,T]** [initials] Set the integrating sphere output to this test's radiance value defined in Table 3.
62. **[D,T]** [initials] Record exact integrating sphere readout value 4.015 mW/cm2/sr.

SKIP 63. ~~**[T]** ____ Take time-stamped digital pictur~~es of the setup and integrating sphere readout.

64. **[O]** [initials] Insert the note "ISOP=[radiance]" and execute camera script **412TAMBL04**, which captures 10 frames for 9 exposure times with filter 0 at focus 3 m at ~~100~~ 110 mm focal length. The estimated duration is 10 minutes.
65. **[D,T]** [initials] Record exact integrating sphere readout value 4.019 mW/cm2/sr.
66. **[D]** [initials] Record image names and parameters in the Image Log.
67. **[D, L]** Notes: 110 mm F.L.

Date 5/2 Time 9:54 Initial [initials]

Radiance Value 5 for the Left Mastcam-Z ~~5.0~~ 5.0

68. **[D]** [initials] Record temperature information:
    - Camera CCD temp 24.0°C Optics temp N/A
69. **[D,~~T~~]** [initials] Set the integrating sphere output to this test's radiance value defined in Table 3.
70. **[D,T]** [initials] Record exact integrating sphere readout value 5.0063 mW/cm2/sr.

SKIP ~~71. [T] ___ Take time-stamped dig~~ital pictures of the setup and integrating sphere readout.

72. **[O]** [initials] Insert the note "ISOP=[radiance]" and execute camera script **412TAMBL05**, which captures 10 frames for 9 exposure times with filter 0 at focus 3 m at ~~100~~ 110mm focal length. The estimated duration is 10 minutes.
73. **[D,T]** [initials] Record exact integrating sphere readout value 5.011 mW/cm2/sr.
74. **[D]** [initials] Record image names and parameters in the Image Log.
75. **[D, L]** Notes: 110mm F.L.

Date 5/2 Time 20:04 Initial Za

Radiance Value 6 for the Left Mastcam-Z 6.0

76. **[D]** Za Record temperature information:
    - Camera CCD temp 23.9°C Optics temp N/A
77. **[D,T]** Za Set the integrating sphere output to this test's radiance value defined in Table 3.
78. **[D,T]** Za Record exact integrating sphere readout value 6.019 mW/cm2/sr.
79. SKIP ~~79. **[T]** ____ Take time-stamped dig~~ital pictures of the setup and integrating sphere readout.
80. **[O]** Za Insert the note "ISOP=[radiance]" and execute camera script **412TAMBL06**, which captures 10 frames for 9 exposure times with filter 0 at focus 3 m at ~~100~~ 110mm focal length. The estimated duration is 10 minutes.
81. **[D,T]** Za Record exact integrating sphere readout value 6.017 mW/cm2/sr.
82. **[D]** Za Record image names and parameters in the Image Log.
83. **[D, L]** Notes: →89

Radiance Value 7 for the Left Mastcam-Z 8.0

84. **[D]** [illegible] Record temperature information:
    - Camera CCD temp 23.9°C Optics temp N/A
85. **[D,T]** [illegible] Set the integrating sphere output to this test's radiance value defined in Table 3.
86. **[D,T]** [illegible] Record exact integrating sphere readout value 8.032 mW/cm2/sr.

SKIP ~~87. [T] ____ Take~~ time-stamped digital pictures of the setup and integrating sphere readout.

88. **[O]** [illegible] Insert the note "ISOP=[radiance]" and execute camera script **412TAMBL07**, which captures 10 frames for 9 exposure times with filter 0 at focus 3 m at ~~100~~ 110mm focal length. The estimated duration is 10 minutes.
89. **[D,T]** [illegible] Record exact integrating sphere readout value 8.042 mW/cm2/sr.
90. **[D]** [illegible] Record image names and parameters in the Image Log.
91. **[D, L]** Notes: →89

Date 5/2 Time 20:27 Initial [signature]

Radiance Value 8 for the Left Mastcam-Z 10.0

92. **[D]** [initials] Record temperature information:
    - Camera CCD temp 23.9°C Optics temp N/A
93. **[D,T]** [initials] Set the integrating sphere output to this test's radiance value defined in Table 3.
94. **[D,T]** [initials] Record exact integrating sphere readout value 10.042 mW/cm2/sr.

SKIP ~~95. **[T]** ____ Take time-stam~~ped digital pictures of the setup and integrating sphere readout.

96. **[O]** [initials] Insert the note "ISOP=[radiance]" and execute camera script **412TAMBL08**, which captures 10 frames for 9 exposure times with filter 0 at focus 3 m at ~~100~~ 110mm focal length. The estimated duration is 10 minutes.
97. **[D,T]** [initials] Record exact integrating sphere readout value ~~10.085~~ 10.105 mW/cm2/sr.
98. **[D]** [initials] Record image names and parameters in the Image Log.
99. **[D, L]** Notes: →179
    23.7°C

Date 5/2 Time 20:49 Initial [illegible]

Data Validation

100. **[V]** [illegible] Upload data to server.

101. **[V]** [illegible] Run the Photon Transfer Jupyter notebook on the acquired data for the Right and Left Mastcam-Z. This analysis can take place while the test continues.

- Create preliminary photon transfer curves.
- Create preliminary flat-field images and radiometric coefficients for each filter.
- Save results in the calibration records.

102. **[V,D, L]** Notes: PROBLEM WITH #6 DATASET

Date 5/2 Time 20:50 Initial [illegible]

Dark Current with the Right and Left Mastcam-Zs

~~103. [T] ____ Turn off the integrating sphere lamp.~~

~~104. [T] ____ Turn off the lights and minimize the room's ambient light.~~

105. **[D]** [illegible] Record the following temperatures:
    - Camera CCD temp ~~23.5°C~~ 23.4°C Optics temp N/A

SKIP 106. **[D,T]** ____ Take digital pictures of the integrating sphere's position, and the whole test/GSE set-up.

107. [[redacted]] Load and execute camera script **441TEMPL03**, which captures 5 dark frames through filter 7 at the exposure times 0.0, 10.0, 20.0, and 100.0 seconds. The estimated duration is 12 minutes.

108. [[redacted]] Load and execute camera script **441TEMPR03**, which captures 5 dark frames through filter 7 at the exposure times 0.0, 10.0, 20.0, and 100.0 seconds. The estimated duration is 12 minutes. SKIP

109. **[D]** [illegible] Record image names and parameters in Image Log.

110. **[T]** [illegible] Cover the integrating sphere exit port window.

111. **[D, L]** Notes: SPHERE LAMP LEFT ON FOR RIGHT CAMERA TESTING
23.4°C

LEFT CAMERA POWERED OFF.

Date 5/2 Time ______ Initial ______

Shutdown Procedure

Continued in next procedure

SKIP

112. **[D,T]** ____ Take digital pictures of this page and the test setup.
113. **[D,O]** ____ Review entries in Image Log, GSE command log, and image headers.
114. **[D, L]** ____ Review calibration procedure and ensure that each task is initialed.
115. **[D, L]** Notes: ____

____

____

116. **[V, L]** ____ Before making the decision to break down the test setup, ensure that adequate data were acquired for the test requirements. See “MastcamZCalPlan” for these requirements.
117. **[V]** Notes: ____

____

____

Data Validator (signature) ____________________

Date ______________ Time ______________

118. **[V, L]** ____ Give the go/no-go decision. Have enough data been acquired to fulfill test requirements? See “MastcamZCalPlan” for these requirements.
119. **[D, L]** ____ Update the Log Document.
120. **[L]** Notes: ____

____

____

Calibration Lead (signature) ____________________

Date ______________ Time ______________

121. [O, L] ____ Ensure that the camera and GSE are in a safe state.
122. [O,D] ____ Review the Image Log with the documentarian. Exchange high-fives.
123. [O] Notes: ____

____

____

Camera Operator (signature) ______________________

Date ______________ Time ______________

124. [T] ____ If the next test does not require the integrating sphere, position it away from the chamber or bench. Otherwise, be sure not to move it. The next test is ___________.
125. [T] ____ Ensure that all other test equipment is safely put away.
126. [T] Notes: ____

____

____

Technician (signature) ______________________

Date ______________ Time ______________

127. [D, L] ____ Double-check this procedure and ensure that the top of each page is initialed with the time and date.
128. [D] ____ Photo-scan this document, save it on the cloud, and file the hardcopy in the Log Binder. Upload the digital pictures taken during this test in the appropriate archive on the cloud. The required links are on the Wiki.
129. [D] ____ Double-check that every required cell the Image Log is accurately filled. When this is complete, print the Image Log and file it the Log Binder after this document.
130. [D] Notes: ____

____

____

Documentarian (signature) ______________________

Date ______________ Time ______________

SKIP
