# Supplementary material for: Pre-Flight Calibration of the Mars 2020 Rover Mastcam Zoom (Mastcam-Z) Multispectral, Stereoscopic Imager
Source: Space Sci Rev. 2021 Feb 18;217(2):29. doi: 10.1007/s11214-021-00795-x (PMC7892537; doi:10.1007/s11214-021-00795-x)
Supplement: Supplementary file 1 — (ZIP 98.6 MB) [file 11214_2021_795_MOESM1_ESM.zip › CalPro_412_R_Photon_v2_03.pdf]

Photon Transfer Procedure for Mastcam-Z Ambient Testing at MSSS (Pro. 4.1.2)

[Procedure version 2.03, prepared by the Mastcam-Z calibration team at Cornell University]

These measurements are performed on the camera and at the temperature designated below as specified in the Mastcam-Z Calibration Plan,

Unit Under Test:

Left FM __X__ Right FM __X__ EQM____ Other________

These measurements are performed at temperature:

-35° C____ -10°C____ +5°C____ Ambient__X__ Other______

These measurements are performed at,

MSSS__X__ ASU____ Other______

Date 5/1/19 **Start Time** 18:40 **End Time** 20:40

Estimated Duration __5.0 hours__

Scheduled Start Time 16:00 **Sch. End Time** 21:00

Calibration Lead [L] HERKENHOFF **Documentarian [D]** CORLIES

Camera Operator [O] VAN BEEK, KW **Technician [T]** WINHOLD, MS

Data Validator [V] TATE, M **Other** ______

Date 5/1/19 Time 16:03 Initials [illegible]

Change Log

Version	Name	Change
v1_01<br>4 June 2018	C. Tate	(first draft)
v1_27<br>30 Oct. 2018	C. Tate	Procedure edits prior to EQM testing
v1_29<br>1 Dec. 2018	C. Tate	Procedure edits after EQM testing
v2_03<br>1 May 2019	C. Tate	Approved version prior to FM testing

Document Approval

Approved by James Bell — Date
Mastcam-Z PI
Arizona State University

[signature] 5/6/19
Approved by Alexander Hayes — Date
Mastcam-Z Calibration Working Group Lead, Cornell University

[signature] 5/1/19
Approved by Justin Maki — Date
Mastcam-Z Deputy PI and Investigation Scientist, Jet Propulsion Laboratory

[signature] 5/1/19
Approved by: — Date
Ken Herkenhoff
Mastcam-Z Co-Investigator, USGS

[signature] 1-5-19
Approved by Christian Tate — Date
Procedure Author
Cornell University

Table of Contents

PHOTON TRANSFER PROCEDURE FOR MASTCAM-Z AMBIENT TESTING AT MSSS (PRO. 4.1.2) **1**

Change Log 2
Document Approval 2
Test Description 4
Software Preparation 4
Table 1. File naming convention for the camera script prefixes and frame filenames: "AAABBBBCDD" *4*
Hardware Installation 6
Figure 1. ASU Floor Plan for Geometric Testing in the TVAC Chamber. The MSSS Floor Plan allows for similar target and source placements relative to the chamber window. *6*
Table 2. Exposure times in milliseconds for each integrating sphere radiance value in Table 3 .... *8*
Table 3. The nominal integrating sphere output radiance values. *8*

MASTCAM-Z TESTS **9**

Dark Current with the Right and Left Mastcam-Zs 9
Center the Integrating Sphere on the Left Mastcam-Z 10
Radiance Value 1 for the Left Mastcam-Z 11
Radiance Value 2 for the Left Mastcam-Z 12
Radiance Value 3 for the Left Mastcam-Z 13
Radiance Value 4 for the Left Mastcam-Z 14
Radiance Value 5 for the Left Mastcam-Z 15
Radiance Value 6 for the Left Mastcam-Z 16
Radiance Value 7 for the Left Mastcam-Z 17
Radiance Value 8 for the Left Mastcam-Z 18
Data Validation 19
Dark Current with the Right and Left Mastcam-Zs 20

SHUTDOWN PROCEDURE **21**

Date 5/1/19 Time 16:05 Initial [illegible]

Test Description

Excerpt from the Calibration Plan 4.2,

> The objectives of these tests are to derive flat field images as well as the coefficients to allow a conversion from reduced (bias, dark, and flat field corrected) DN/s to absolute radiometric response (W/cm^2/sr per filter) for (a) the R, G, and B microfilters of the Bayer Pattern Filter detectors in each camera head (clear filter), (b) the 14 non-solar Mastcam-Z spectral filters "Science Filters", and, if time permits, (c) the two Mastcam-Z neutral density solar filters; and to provide an estimate of the uncertainty in these coefficients and, at Priority 2, their temperature dependence. This test builds off the Section 4.3 – Spectral Throughput Calibration to accurately account for the filter spectral response in the conversion. The requirement of knowing the relative response on the shape of the spectral throughput to ±5% combined with the absolute Radiance accuracy of the integration sphere at ±5% still allows the ±10% absolute radiometric calibration requirement to be met.

Software Preparation

The software and files required for this test are prepared well in advance of test day. This checklist ensures that the following are present, debugged, and executable: (1) all fast-look scripts, (2) automated header generation of all relevant camera parameters, target positioning, and metadata, (3) all camera scripts that command the camera unit, and (4) the directories/file-paths pointing to the data repositories of this specific test.

Table 1. File naming convention for the camera script prefixes and frame filenames: "AAABBBBCDD"

Code	Name	Example	Value
"AAA"	Calibration Plan Section	"411" = Cal. Plan 4.1.1 chapter 4, section 1, subsection 1	412
"BBBB"	Location of test or ASU Chamber temperature	"MSSS" = test at MSSS, "TN10" = ASU TVAC -10C, …	TAMB
"C"	Camera unit under test	"L" = Left Mastcam-Z, "R" = Right Mastcam-Z, "E" =EQM, "C" =COTS	R/L
"DD"	Part of test (radiance value)	"00" = test set up, "01" = first radiance value …	00-08

1. **[D]** [illegible] Look up the daily calibration schedule and record the scheduled start and end time of this test on the cover page of this document. Also fill out and double-check the other information on the cover page.
2. **[D]** CX Ensure that all supplemental manuals are on hand. These are,
   - ~~Labsphere_Manual,~~
   - Validator_Manual, Documentarian_Manual
   - MastcamZCalPlan
3. **[D]** [illegible] Ensure that the Image Log is present and ready to use. Find and open the Google Sheets file "Image_Log_42". There is a link on the Wiki.
4. **[V]** [illegible] Check that all Calgorithms fast-look and validation scripts are present, up-to-date, and ready to analyze test output. Find and open the "Radiometric_Calibration_42_Validation" Jupyter notebook. There is a link on the Wiki.
5. **[O]** [illegible] Check that all camera scripts required for this test are present, up-to-date and ready to command the ground support equipment (GSE). These are,
   - **412TAMBR00 - 412TAMBR08**
   - **412TAMBL00 - 412TAMBL08**
   - **441TEMPR03, 441TEMPL03**
6. **[O,V,D, L]** Notes:

LATE START DUE TO CLEAN ROOM SETUP, METROLOGY WALKTHROUGH, INITIALIZATION

Hardware Installation

This procedure is for the ambient TVAC chamber testing at MSSS. Figure 1 shows the nominal layout of the TVAC chamber, workspace, Mastcam-Zs, ground support equipment (GSE), targets, sources, and other equipment necessary for this test if it happens at ASU. Although MSSS' cleanroom is different than ASU's, the placement of the targets and sources relative to the chamber window is similar.

Figure 1. ASU Floor Plan for Geometric Testing in the TVAC Chamber. The MSSS Floor Plan allows for similar target and source placements relative to the chamber window.

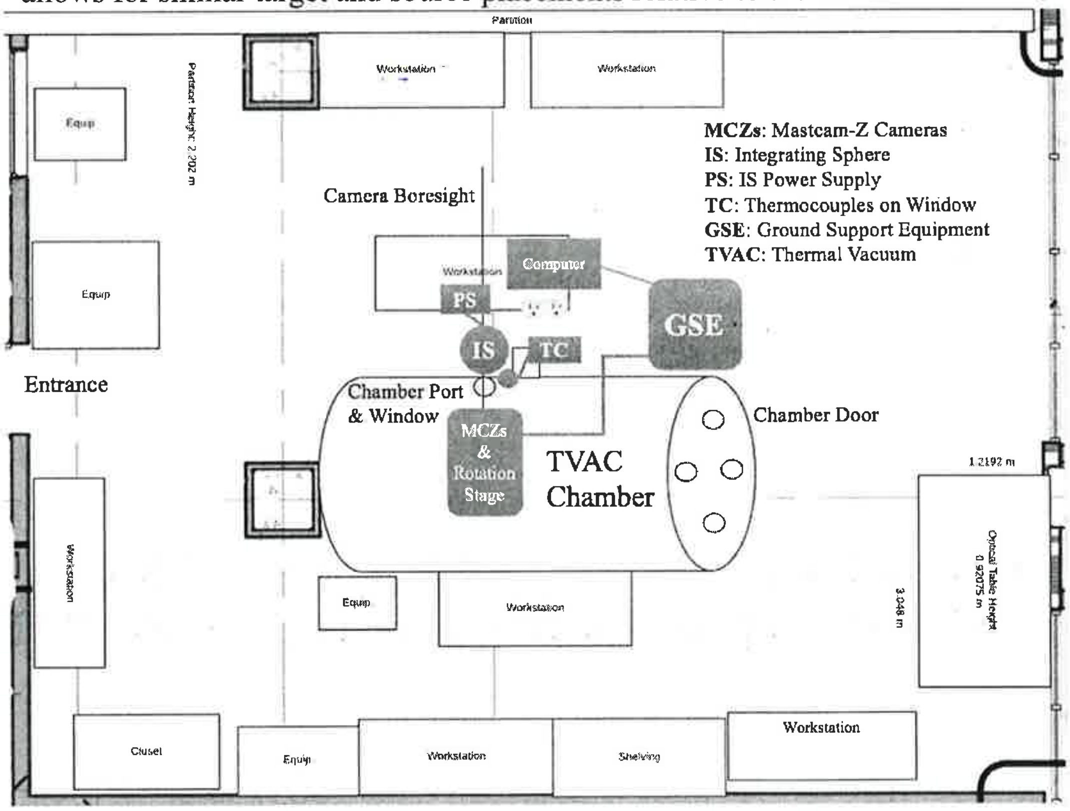

7. [T,O, L] ✓ Ensure that all personnel in the cleanroom are following the cleanroom practices for electrostatic discharge, proper clothing and other safety concerns.
8. ~~[T] ____ Double check that nitrogen is flowing over the Mastcam-Zs.~~ SKIP
9. [T] ✓ Verify that the thermocouples are turned on and properly reading out.
10. [O,T] ✓ Ensure that the camera unit and GSE wires are secure, kink-free, and do not present tripping hazards when the lights are turned off.

Date 5/1 Time 18:52 Initial [initials]

11. **[O,D]** [initials] Check the camera temperature and ensure nominal operation. L 21.3, R 21.9°C
12. **[D]** [initials] Record the following environmental information:
    - Cleanroom temperature 67.6°F pressure N/A humidity 55%
13. **[O,D, L]** Notes:

    LEFT CAMERA POWER OFF.

14. **[D,T]** [initials] Take time-stamped pictures of the integrating sphere and the whole test/GSE set-up. RAN RT. CAMERA DARKCURRENT → STEPS 19, 20, 21, 23
15. **[T]** [initials] Power on the integrating sphere. Record the time the lamp is turned on 19:07. WARMUP 20 mins
16. **[D,T]** [initials] Record the exact readout value of the integrating sphere's radiance: 1.0004 mW/cm2/sr.
17. **[T,O, L]** ~~Confirm that the camera systems and GSEs are powered on and ready for use.~~ SKIP
18. **[D, L]** Notes:

    111 24.2° 132

Date 5/1 Time ______ Initial ______

Table 2. Exposure times in milliseconds for each integrating sphere radiance value in Table 3 at **100 mm focal length. The estimated duration is 13 minutes.** (Note that each sequence of exposure times begins and ends with a zero-time exposure in order to evaluate bias evolution.)

	**Time0**	**Time1**	**Time2**	**Time3**	**Time4**	**Time5**	**Time6**	**Time7**	**Time8**
**Radiance 1**	0.0	3.0	4.0	6.0	10.0	22.0	30.0	60.0	80.0
**Radiance 2**	0.0	3.0	4.0	6.0	10.0	14.0	22.0	40.0	80.0
**Radiance 3**	0.0	2.0	3.0	4.0	6.0	10.0	22.0	30.0	40.0
**Radiance 4**	0.0	2.0	3.0	4.0	6.0	10.0	14.0	22.0	30.0
**Radiance 5**	0.0	1.0	1.5	2.0	3.0	6.0	10.0	14.0	22.0
**Radiance 6**	0.0	0.5	1.0	2.0	3.0	4.0	6.0	10.0	14.0
**Radiance 7**	0.0	0.5	1.0	1.5	2.0	3.0	4.0	10.0	14.0
**Radiance 8**	0.0	0.5	1.0	1.5	2.0	2.5	3.0	6.0	10.0

Table 3. The nominal integrating sphere output radiance values.

If the pre-test reveals that a scaling is necessary, then the scaled radiance values are recorded here and used for the remainder of the photon transfer testing. (Note, these radiance values are in units of 1.0-2.4 micron band integrated spectral radiance mW/cm2/sr, true radiance is about a factor of two higher.)

	**IS Output Radiances**	**Nominal Radiance [mW/cm2/sr]**	**Scaled Radiance [mW/cm2/sr]**
✓	Radiance 1	1.0	
	Radiance 2	2.0	
	Radiance 3	3.0	
	Radiance 4	4.0	
✓	Radiance 5	5.0	
	Radiance 6	6.0	
	Radiance 7	8.0	
	Radiance 8	10.0	

Date 5/1 Time 18:54 Initial Ea

Mastcam-Z Tests

Dark Current with the Right ~~and Left~~ Mastcam-Zs

19. **[T]** Ea Turn off the lights and minimize the room's ambient light.
20. **[D]** Ea Record the following temperatures:
    - Camera CCD temp 21.9°C   Optics temp N/A
21. **[D,T]** Ea Take digital pictures of the integrating sphere's position and the whole test/GSE set-up. CAMERA POWERED ON
22. SKIP **[[redacted]]** ~~Load and execute camera script **441TEMPL03**, which captures 5 dark frames through filter 7 at the exposure times 0.0, 10.0, 20.0, and 100 seconds. The estimated duration is 12 minutes.~~
23. 2X **[O]** Load and execute camera script **441TEMPR03**, which captures 5 dark frames through filter 7 at the exposure times 0.0, 10.0, 20.0, and 100 seconds. The estimated duration is 12 minutes.
24. **[D]** Ea Record image names and parameters in Image Log.
25. **[T]** Ea Lights on.
26. **[D, L]** Notes: 24.5°C 1st
    25.1°C 2nd 154
    DIFFERENCE BETWEEN DARK FRAMES SHOWS NO SIGNIFICANT STRUCTURE AND AVERAGE ~~EN~~ DARK CURRENT SIMILAR, CONSISTENT WITH SMALL CHANGE IN CCD TEMPERATURE.

Date 5/1/19 Time 17:29 Initial [initials]

Center the Integrating Sphere on the ~~Left~~ RIGHT Mastcam-Z

27. **[T]** [initials] Move the integrating sphere close to Mastcam-Z's boresight.
28. **[O]** [initials] Insert the note "ISOP=[radiance]" and execute camera script **423TAMB~~L~~R00**. OPEN EXIT PORT (REMOVE COVER) This script captures one auto-exposure at 40% full-well ~~and one bias frame~~ for filter 0 at 26 mm focal length. REMOVE EXIT PORT COVER, F.L.=79mm,
29. **[V,O,T]** [initials] Open images, and if the images show that the integrating sphere is not centered, center the integrating sphere disc in the frame. Recapture **423TAMB~~L~~R00** frames if necessary. REPEATED @ 79mm, 100mm
30. **[D]** [initials] Record image names and parameters in the image Log.
31. **[T]** [initials] Take digital pictures of the integrating sphere's position and the whole test/GSE set-up.
32. **[T]** [initials] Visually estimate the distance from the integrating sphere and the Mastcam-Z's sunshade. This distance is approximately 36 cm.
33. **[T]** [initials] Lights off
34. **[D, L]** Notes:

24.8°C, 25.0°C, 25.2°C, 25.3°C
→ 12
SPHERE WAS LOWERED TO 19 7/8" ABOVE FLOOR TO CENTER EXIT PORT IN FOV.

Date 5/1/19 Time 19:50 Initial [illegible]

Radiance Value 1 for the ~~Left~~ RIGHT Mastcam-Z

35. **[D]** [illegible] Record temperature information:
    - Camera CCD temp 25.0°C Optics temp N/A
36. **[D,T]** [illegible] Set the integrating sphere output to this test's radiance value defined in Table 3.
37. **[D,T]** [illegible] Record exact integrating sphere readout value 1.0079 mW/cm2/sr.
38. SKIP ~~**[T]**~~ ____ ~~Take~~ time-stamped digital pictures of the setup and integrating sphere ~~readout~~. LIGHTS OUT
39. **[O]** [illegible] Insert the note "ISOP=[radiance]" and execute camera script **412TAMB~~L~~R01**, which captures 10 frames for 9 exposure times with filter 0 at focus 3 m at 100mm focal length. The estimated duration is ~~22~~ 9 minutes.
40. **[D,T]** [illegible] Record exact integrating sphere readout value 1.0051 mW/cm2/sr.
41. **[D]** [illegible] Record image names and parameters in the Image Log.
42. **[D, L]** Notes: ~~[illegible]~~ 25.0°C
    → 89

Date ______ Time ______ Initial ______

Radiance Value 2 for the Left Mastcam-Z

43. **[D]** ____ Record temperature information:
    - Camera CCD temp _______________ Optics temp _______________
44. **[D,T]** ____ Set the integrating sphere output to this test's radiance value defined in Table 3.
45. **[D,T]** ____ Record exact integrating sphere readout value ___________ mW/cm2/sr.
46. **[T]** ____ Take time-stamped digital pictures of the setup and integrating sphere readout.
47. **[O]** ____ Insert the note "ISOP=[radiance]" and execute camera script **412TAMBL02**, which captures 10 frames for 9 exposure times with filter 0 at focus 3 m at 100mm focal length. The estimated duration is 22 minutes.
48. **[D,T]** ____ Record exact integrating sphere readout value ___________ mW/cm2/sr.
49. **[D]** ____ Record image names and parameters in the Image Log.
50. **[D, L]** Notes: ____

____

____

SKIP

Date ______ Time ______ Initial ______

Radiance Value 3 for the Left Mastcam-Z

51. **[D]** ____ Record temperature information:
    - Camera CCD temp ______________ Optics temp ______________
52. **[D,T]** ____ Set the integrating sphere output to this test's radiance value defined in Table 3.
53. **[D,T]** ____ Record exact integrating sphere readout value __________ mW/cm2/sr.
54. **[T]** ____ Take time-stamped digital pictures of the setup and integrating sphere readout.
55. **[O]** ____ Insert the note "ISOP=[radiance]" and execute camera script **412TAMBL03**, which captures 10 frames for 9 exposure times with filter 0 at focus 3 m at 100mm focal length. The estimated duration is 22 minutes.
56. **[D,T]** ____ Record exact integrating sphere readout value __________ mW/cm2/sr.
57. **[D]** ____ Record image names and parameters in the Image Log.
58. **[D, L]** Notes: ____

____

____

SKIP

Date ______ Time ______ Initial ______

Radiance Value 4 for the Left Mastcam-Z

59. **[D]** ____ Record temperature information:
    - Camera CCD temp ______________ Optics temp ______________
60. **[D,T]** ____ Set the integrating sphere output to this test's radiance value defined in Table 3.
61. **[D,T]** ____ Record exact integrating sphere readout value __________ mW/cm2/sr.
62. **[T]** ____ Take time-stamped digital pictures of the setup and integrating sphere readout.
63. **[O]** ____ Insert the note "ISOP=[radiance]" and execute camera script **412TAMBL04**, which captures 10 frames for 9 exposure times with filter 0 at focus 3 m at 100mm focal length. The estimated duration is 22 minutes.
64. **[D,T]** ____ Record exact integrating sphere readout value __________ mW/cm2/sr.
65. **[D]** ____ Record image names and parameters in the Image Log.
66. **[D, L]** Notes: ____

____

____

SKIP

Date 5/1/19 Time 20:0[illegible] Initial [illegible]

Radiance Value 5 for the ~~Left~~ RIGHT Mastcam-Z

67. **[D]** [initials] Record temperature information:
    - Camera CCD temp 25.0 Optics temp N/A
68. **[D,T]** [initials] Set the integrating sphere output to this test's radiance value defined in Table 3.
69. **[D,T]** [initials] Record exact integrating sphere readout value 5.0013 mW/cm2/sr.
70. ~~**[T]** ____ Take time-stamped~~ digital pictures of the ~~setup and~~ integrating sphere readout.
71. SKIP **[O]** ~~[initials] Insert the note "ISOP=[radiance]"~~ STET and execute camera script **412TAMB~~L~~R05**, which captures 10 frames for 9 exposure times with filter 0 at focus 3 m at 100mm focal length. The estimated duration is ~~22~~ 9 minutes.
72. **[D,T]** [initials] Record exact integrating sphere readout value 5.0088 mW/cm2/sr.
73. **[D]** [initials] Record image names and parameters in the Image Log.
74. **[D, L]** Notes: TURN OFF SPHERE

    25.1°C → 1179

Radiance Value 6 for the Left Mastcam-Z

75. **[D]** ____ Record temperature information:
    - Camera CCD temp _______________ Optics temp _______________
76. **[D,T]** ____ Set the integrating sphere output to this test's radiance value defined in Table 3.
77. **[D,T]** ____ Record exact integrating sphere readout value ___________ mW/cm2/sr.
78. **[T]** ____ Take time-stamped digital pictures of the setup and integrating sphere readout.
79. **[O]** ____ Insert the note "ISOP=[radiance]" and execute camera script **412TAMBL06**, which captures 10 frames for 9 exposure times with filter 0 at focus 3 m at 100mm focal length. The estimated duration is 22 minutes.
80. **[D,T]** ____ Record exact integrating sphere readout value ___________ mW/cm2/sr.
81. **[D]** ____ Record image names and parameters in the Image Log.
82. **[D, L]** Notes: ____

____

____

SKIP

Date _____ Time _____ Initial _____

Radiance Value 7 for the Left Mastcam-Z

83. **[D]** ____ Record temperature information:
    - Camera CCD temp ______________ Optics temp ______________
84. **[D,T]** ____ Set the integrating sphere output to this test's radiance value defined in Table 3.
85. **[D,T]** ____ Record exact integrating sphere readout value __________ mW/cm2/sr.
86. **[T]** ____ Take time-stamped digital pictures of the setup and integrating sphere readout.
87. **[O]** ____ Insert the note "ISOP=[radiance]" and execute camera script **412TAMBL07**, which captures 10 frames for 9 exposure times with filter 0 at focus 3 m at 100mm focal length. The estimated duration is 22 minutes.
88. **[D,T]** ____ Record exact integrating sphere readout value __________ mW/cm2/sr.
89. **[D]** ____ Record image names and parameters in the Image Log.
90. **[D, L]** Notes: ____

____

____

SKIP

Radiance Value 8 for the Left Mastcam-Z

SKIP

91. **[D]** ____ Record temperature information:
    - Camera CCD temp ______________ Optics temp ______________
92. **[D,T]** ____ Set the integrating sphere output to this test's radiance value defined in Table 3.
93. **[D,T]** ____ Record exact integrating sphere readout value __________ mW/cm2/sr.
94. **[T]** ____ Take time-stamped digital pictures of the setup and integrating sphere readout.
95. **[O]** ____ Insert the note "ISOP=[radiance]" and execute camera script **412TAMBL08**, which captures 10 frames for 9 exposure times with filter 0 at focus 3 m at 100mm focal length. The estimated duration is 22 minutes.
96. **[D,T]** ____ Record exact integrating sphere readout value __________ mW/cm2/sr.
97. **[D]** ____ Record image names and parameters in the Image Log.
98. **[D, L]** Notes: ____

____

____

Data Validation

99. [V] ~~Zen~~ Upload data to server.

100. [V] Zen Run the Photon Transfer Jupyter notebook on the acquired data for the Right and Left Mastcam-Z. This analysis can take place while the test continues.

- Create preliminary photon transfer curves.
- Create preliminary flat-field images and radiometric coefficients for each filter.
- Save results in the calibration records.

101. [V,D, L] Notes: GAIN = 16.0 e⁻/DN, READ NOISE = 21.7 e⁻

Date 5/1/19 Time 20:15 Initial [illegible]

Dark Current with the Right ~~and Left~~ Mastcam-Zs

102. **[T]** [illegible] Turn off the lights and minimize the room's ambient light.
103. **[D]** [illegible] Record the following temperatures:
    - Camera CCD temp ~~25.8°C~~ 24.8 Optics temp N/A

SKIP 104. **[D,T]** ____ ~~Take digital~~ pictures of the integrating sphere's position, and the whole test/GSE set-up.

SKIP 105. **[O]** Load and execute camera script **441TEMPL03**, which captures 5 dark frames through filter 7 at the exposure times 0.0, 10.0, 20.0, and 100.0 seconds. The estimated duration is 12 minutes.

✓ 106. **[O]** Load and execute camera script **441TEMPR03**, which captures 5 dark frames through filter 7 at the exposure times 0.0, 10.0, 20.0, and 100.0 seconds. The estimated duration is 12 minutes.

107. **[D]** [illegible] Record image names and parameters in Image Log.
108. **[T]** [illegible] ~~Un~~cover the port window.
109. **[D, L]** Notes: TURN LIGHTS ON IN ROOM
    25.1°C
    → 176

Date 5/1/19 Time 20:33 Initial [illegible]

Shutdown Procedure

110. **[D,T]** [illegible] Take digital pictures of this page and the test setup.
111. **[D,O]** [illegible] Review entries in Image Log, GSE command log, and image headers.
112. **[D, L]** [illegible] Review calibration procedure and ensure that each task is initialed.
113. **[D, L]** Notes: THE SPHERE WAS LOWERED TO 19 7/8" ABOVE FLOOR TO CENTER IN FOV

114. **[V, L]** [illegible] Before making the decision to break down the test setup, ensure that adequate data were acquired for the test requirements. See "MastcamZCalPlan" for these requirements.
115. **[V]** Notes: All good

Data Validator (signature) Christian [illegible]

Date 5-1-19 Time 8:40 (20:40)

116. **[V, L]** [illegible] Give the go/no-go decision. Have enough data been acquired to fulfill test requirements? See "MastcamZCalPlan" for these requirements.
117. **[D, L]** [illegible] Update the Log Document.
118. **[L]** Notes: SOME OF US HAVE BEEN HERE SINCE 7:30 THIS MORNING, SO WE QUIT BEFORE 9PM.

Calibration Lead (signature) [illegible]

Date 5/1/19 Time 20:50

119. **[O, L]** JVB Ensure that the camera and GSE are in a safe state.
120. **[O,D]** JVB Review the Image Log with the documentarian. Exchange high-fives.
121. **[**[redacted]**]** Notes: Nothing of note.

Camera Operator (signature)

Date 5-1-19 Time 9:08 pm

122. **[T]** AW If the next test does not require the integrating sphere, position it away from the chamber or bench. Otherwise, be sure not to move it. The next test is METROLOGY
123. **[T]** AW Ensure that all other test equipment is safely put away.
124. **[T]** Notes: KAPTON TAPE MARKS FEET OF INT. SPHERE CART PLACEMENT NEAR CH3.

Technician (signature)

Date 5/1/19 Time 21:03

125. **[D, L]** PMC Double-check this procedure and ensure that the top of each page is initialed with the time and date.
126. **[D]** PMC Photo-scan this document, save it on the cloud, and file the hardcopy in the Log Binder. Upload the digital pictures taken during this test in the appropriate archive on the cloud. The required links are on the Wiki.
127. **[D]** PMC Double-check that every required cell the Image Log is accurately filled. When this is complete, print the Image Log and file it the Log Binder after this document.
128. **[D]** Notes:

Documentarian (signature)

Date 5/2/19 Time 8:37AM
