# Supplementary material for: Pre-Flight Calibration of the Mars 2020 Rover Mastcam Zoom (Mastcam-Z) Multispectral, Stereoscopic Imager
Source: Space Sci Rev. 2021 Feb 18;217(2):29. doi: 10.1007/s11214-021-00795-x (PMC7892537; doi:10.1007/s11214-021-00795-x)
Supplement: Supplementary file 1 — (ZIP 98.6 MB) [file 11214_2021_795_MOESM1_ESM.zip › CalPro_412_R_Photon_v2_06.pdf]

Photon Transfer Procedure for Right Mastcam-Z Ambient Testing at MSSS (Pro. 4.1.2)

[Procedure version 2.06, prepared by the Mastcam-Z calibration team at Cornell University]

These measurements are performed on the camera and at the temperature designated below as specified in the Mastcam-Z Calibration Plan,

Unit Under Test:

Left FM ~~X~~ **Right FM** X **EQM**____ **Other**____

These measurements are performed at temperature:

-35° C____ **-10°C**____ **+5°C**____ **Ambient** X **Other**____

These measurements are performed at:

MSSS X **ASU**____ **Other**____

Date 5/2/19 **Start Time** 21:00 **End Time** 22:50

Estimated Duration **3.0 hours**

Scheduled Start Time____ **Sch. End Time**____

Calibration Lead [L] KEN Herkenhoff **Documentarian [D]** MEGAN, EMILY Lakdawalla

Camera Operator [O] ~~DARIAN~~, ~~CHRIS~~ JASON, KW, DD **Technician [T]** CHRISTIAN Tate

Data Validator [V] MASON Starr **Other**____

Change Log

Version	Name	Change
v1_01<br>4 June 2018	C. Tate	(first draft)
v1_27<br>30 Oct. 2018	C. Tate	Procedure edits prior to EQM testing
v1_29<br>1 Dec. 2018	C. Tate	Procedure edits after EQM testing
v2_04<br>2 May 2019	C. Tate	Updated after running similar procedures
v2_06<br>2 May 2019	K. Herkenhoff	Approved version prior to FM testing

Document Approval

Approved by James Bell Date
Mastcam-Z PI
Arizona State University

5/6/19
Approved by Alexander Hayes Date
Mastcam-Z Calibration Working Group
Lead, Cornell University

Approved by Justin Maki Date
Mastcam-Z Deputy PI and Investigation
Scientist, Jet Propulsion Laboratory

5/2/19
Approved by: Date
Ken Herkenhoff
Mastcam-Z Co-Investigator, USGS

5/3/19
Approved by Christian Tate Date
Procedure Author
Cornell University

Table of Contents

PHOTON TRANSFER PROCEDURE FOR RIGHT MASTCAM-Z AMBIENT TESTING AT MSSS (PRO. 4.1.2) **1**

CHANGE LOG 2
DOCUMENT APPROVAL 2
TEST DESCRIPTION 4
SOFTWARE PREPARATION 4
Table 1. File naming convention for the camera script prefixes and frame filenames: "AAABBBBCDD" *4*
HARDWARE INSTALLATION 6
Figure 1. ASU Floor Plan for Geometric Testing in the TVAC Chamber. The MSSS Floor Plan allows for similar target and source placements relative to the chamber window. *6*
Table 2. Exposure times in milliseconds for each integrating sphere radiance value in Table 3 .... *8*
Table 3. The nominal integrating sphere output radiance values. *8*

MASTCAM-Z TESTS **9**

DARK CURRENT WITH THE RIGHT AND RIGHT MASTCAM-ZS 9
CENTER THE INTEGRATING SPHERE ON THE RIGHT MASTCAM-Z 10
RADIANCE VALUE 1 FOR THE RIGHT MASTCAM-Z 11
RADIANCE VALUE 2 FOR THE RIGHT MASTCAM-Z 12
RADIANCE VALUE 3 FOR THE RIGHT MASTCAM-Z 13
RADIANCE VALUE 4 FOR THE RIGHT MASTCAM-Z 14
RADIANCE VALUE 5 FOR THE RIGHT MASTCAM-Z 15
RADIANCE VALUE 6 FOR THE RIGHT MASTCAM-Z 16
RADIANCE VALUE 7 FOR THE RIGHT MASTCAM-Z 17
RADIANCE VALUE 8 FOR THE RIGHT MASTCAM-Z 18
DATA VALIDATION 19
DARK CURRENT WITH THE RIGHT AND RIGHT MASTCAM-ZS 20

SHUTDOWN PROCEDURE **21**

Test Description

Excerpt from the Calibration Plan 4.2,

> The objectives of these tests are to derive flat field images as well as the coefficients to allow a conversion from reduced (bias, dark, and flat field corrected) DN/s to absolute radiometric response ($W/cm^2/sr$ per filter) for (a) the R, G, and B microfilters of the Bayer Pattern Filter detectors in each camera head (clear filter), (b) the 14 non-solar Mastcam-Z spectral filters "Science Filters", and, if time permits, (c) the two Mastcam-Z neutral density solar filters; and to provide an estimate of the uncertainty in these coefficients and, at Priority 2, their temperature dependence. This test builds off the Section 4.3 – Spectral Throughput Calibration to accurately account for the filter spectral response in the conversion. The requirement of knowing the relative response on the shape of the spectral throughput to ±5% combined with the absolute Radiance accuracy of the integration sphere at ±5% still allows the ±10% absolute radiometric calibration requirement to be met.

Software Preparation

The software and files required for this test are prepared well in advance of test day. This checklist ensures that the following are present, debugged, and executable: (1) all fast-look scripts, (2) automated header generation of all relevant camera parameters, target positioning, and metadata, (3) all camera scripts that command the camera unit, and (4) the directories/file-paths pointing to the data repositories of this specific test.

Table 1. File naming convention for the camera script prefixes and frame filenames: "AAABBBBCDD"

Code	Name	Example	Value
"AAA"	Calibration Plan Section	"411" = Cal. Plan 4.1.1 chapter 4, section 1, subsection 1	412
"BBBB"	Location of test or ASU Chamber temperature	"MSSS" = test at MSSS, "TN10" = ASU TVAC -10C, …	TAMB
"C"	Camera unit under test	"L" = Right Mastcam-Z, "R" = Right Mastcam-Z, "E" =EQM, "C" =COTS	R
"DD"	Part of test (radiance value)	"00" = test set up, "01" = first radiance value …	00-08

Date 5/2 Time 21:00 Initial [illegible]

1. **[D]** [illegible] Look up the daily calibration schedule and record the scheduled start and end time of this test on the cover page of this document. Also fill out and double-check the other information on the cover page.
2. **[D]** ____ Ensure that all supplemental manuals are on hand. These are,
   - Labsphere_Manual,
   - Validator_Manual, Documentarian_Manual
   - MastcamZCalPlan
3. **[D]** [illegible] Ensure that the Image Log is present and ready to use. Find and open the Google Sheets file "Image_Log_42". There is a link on the Wiki.
4. **[V]** [illegible] Check that all Calgorithms fast-look and validation scripts are present, up-to-date, and ready to analyze test output. Find and open the "Radiometric_Calibration_42_Validation" Jupyter notebook. There is a link on the Wiki.
5. **[O]** [illegible] Check that all camera scripts required for this test are present, up-to-date and ready to command the ground support equipment (GSE). These are,
   - **412TAMBR00 - 412TAMBR08** 423TAMBR00
   - **441TEMPR03, 441TEMPL03**
6. **[O,V,D, L]** Notes:

RUNNING SCRIPTS NOT RUN LAST NIGHT

Hardware Installation

This procedure is for the ambient TVAC chamber testing at MSSS. Figure 1 shows the nominal layout of the TVAC chamber, workspace, Mastcam-Zs, ground support equipment (GSE), targets, sources, and other equipment necessary for this test if it happens at ASU. Although MSSS' cleanroom is different than ASU's, the placement of the targets and sources relative to the chamber window is similar.

Figure 1. ASU Floor Plan for Geometric Testing in the TVAC Chamber. The MSSS Floor Plan allows for similar target and source placements relative to the chamber window.

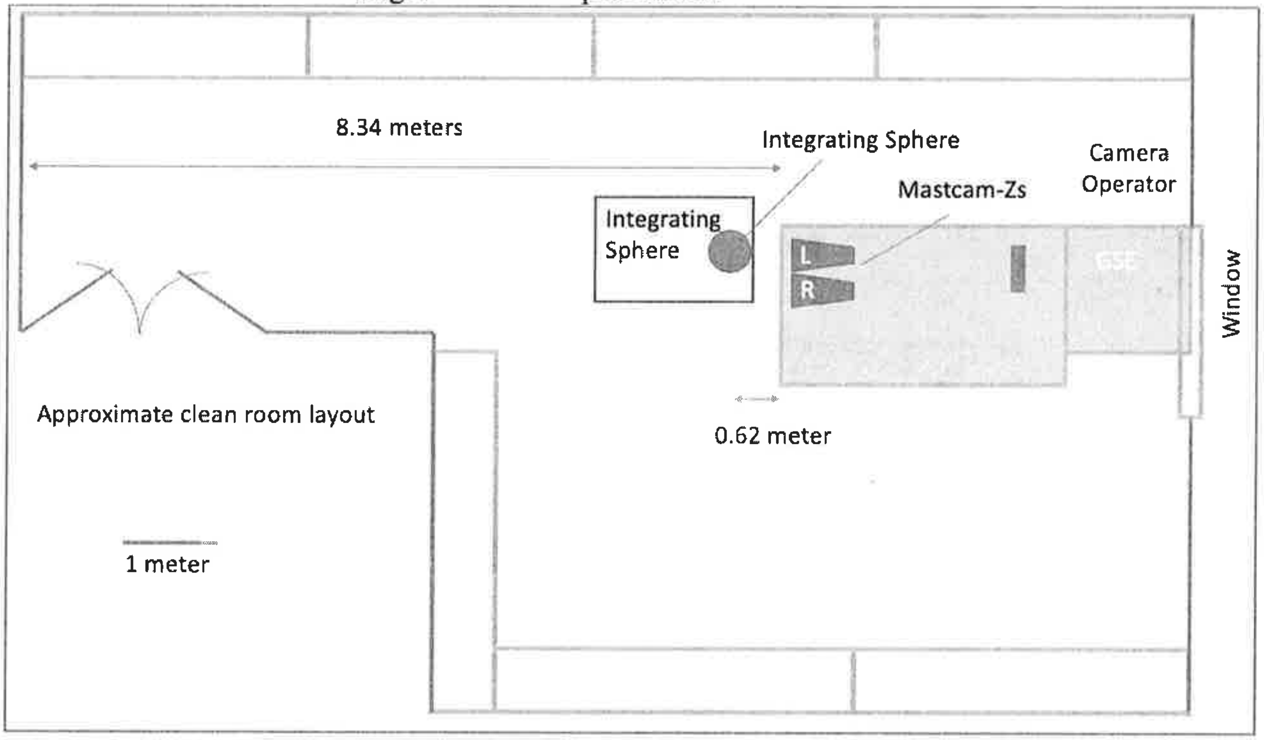

7. **[T,O, L]** ___ Ensure that all personnel in the cleanroom are following the cleanroom practices for electrostatic discharge, proper clothing and other safety concerns.
8. **[T]** ___ Double check that ionized air is flowing over the Mastcam-Zs.
9. **[T]** ___ Verify that the thermocouples are turned on and properly reading out.
10. **[O,T]** ___ Ensure that the camera unit and GSE wires are secure, kink-free, and do not present tripping hazards when the lights are turned off.
11. **[O,D]** ___ Check the camera temperature and ensure nominal operation.

12. **[D]** [illegible] Record the following environmental information:
    - Cleanroom temperature 65.8°F pressure _______ humidity 65%
13. **[O,D, L]** Notes:

DROVE FOCAL LENGTH TO 79 mm

____

____

SKIP

14. **[D,T]** ____ Take time-stamped pictures of the integrating sphere and the whole test/GSE set-up.

SKIP

15. **[T]** ____ Power on the integrating sphere. Follow the procedure in "Labsphere_Manual". Record the time the lamp is turned on ____________. The bulb and radiometer take about 20 minutes to warm up and stabilize, but that can happen while we continue to center the sphere on the Mastcam-Z boresight.
16. **[D,T]** ____ Record the exact readout value of the integrating sphere's radiance: __________ mW/cm2/sr.
17. **[T,O, L]** ____ Confirm that the camera systems and GSEs are powered on and ready for use.
18. **[D, L]** Notes:

____

____

____

Date 5/2 Time 21:08 Initial [illegible]

Table 2. Exposure times in milliseconds for each integrating sphere radiance value in Table 3 at **100 mm focal length. The estimated duration is 13 minutes.** (Note that each sequence of exposure times begins and ends with a zero-time exposure in order to evaluate bias evolution.)

	Time0	Time1	Time2	Time3	Time4	Time5	Time6	Time7	Time8
**Radiance 1**	0.0	3.0	4.0	6.0	10.0	22.0	30.0	60.0	80.0
**Radiance 2**	0.0	3.0	4.0	6.0	10.0	14.0	22.0	40.0	80.0
**Radiance 3**	0.0	2.0	3.0	4.0	6.0	10.0	22.0	30.0	40.0
**Radiance 4**	0.0	2.0	3.0	4.0	6.0	10.0	14.0	22.0	30.0
**Radiance 5**	0.0	1.0	1.5	2.0	3.0	6.0	10.0	14.0	22.0
**Radiance 6**	0.0	0.5	1.0	2.0	3.0	4.0	6.0	10.0	14.0
**Radiance 7**	0.0	0.5	1.0	1.5	2.0	3.0	4.0	10.0	14.0
**Radiance 8**	0.0	0.5	1.0	1.5	2.0	2.5	3.0	6.0	10.0

Table 3. The nominal integrating sphere output radiance values.
If the pre-test reveals that a scaling is necessary, then the scaled radiance values are recorded here and used for the remainder of the photon transfer testing. (Note, these radiance values are in units of 1.0-2.4 micron band integrated spectral radiance mW/cm2/sr, true radiance is about a factor of two higher.)

IS Output Radiances	Nominal Radiance [mW/cm2/sr]	Scaled Radiance [mW/cm2/sr]
~~Radiance 1~~	~~1.0~~	
Radiance 2	2.0	
Radiance 3	3.0	
Radiance 4	4.0	
~~Radiance 5~~	~~5.0~~	
Radiance 6	6.0	
Radiance 7	8.0	
Radiance 8	10.0	

Date 5/2 Time 21:08 Initial [illegible]

Mastcam-Z Tests

Dark Current with the ~~Right and~~ Right Mastcam-Z~~s~~

STET ~~SKIP~~

CLOSED PORT COVER

19. **[T]** [illegible] Turn off the lights and minimize the room's ambient light.
20. **[D]** [illegible] Record the following temperatures:
    - Camera CCD temp 23.7°C   Optics temp ______________
21. SKIP ~~**[D,T]** ____ Take digital pictures of the integrating sphere's position~~ and the whole test/GSE set-up.
22. [[redacted]] Load and execute camera script **441TEMPL03**, which captures 5 dark frames through filter 7 at the exposure times 0.0, 10.0, 20.0, and 100 seconds. The estimated duration is 12 minutes. SKIP
23. **[O]** Load and execute camera script **441TEMPR03**, which captures 5 dark frames through filter 7 at the exposure times 0.0, 10.0, 20.0, and 100 seconds. The estimated duration is 12 minutes.
24. **[D]** [illegible] Record image names and parameters in Image Log.
25. ~~**[T]** ____ Lights on.~~ SKIP
26. **[D, L]** Notes: ~~USE DARK DATA TAKEN AFTER LEFT CAMERA PHOTON TRANSFER RUN ON~~ 5/2/19
    NEED DARKS, IGNORE SLASH

23.8°C

Date 5/2 Time 21:08 Initial [illegible]

Center the Integrating Sphere on the Right Mastcam-Z

27. **[T]** ____ Move the integrating sphere close to Mastcam-Z's boresight.
28. **[T]** ____ Remove the integrating sphere's port cover.
29. **[O]** ____ Insert the note "ISOP=[radiance]" and execute camera script **423TAMB~~L~~R00**. This script captures one auto-exposure at 40% full-well for filter 0 at 79 mm focal length (because we want to center it in 100mm).
30. **[V,O,T]** ____ Open images, and if the images show that the integrating sphere is not centered, center the integrating sphere disc in the frame. Recapture **423TAMB~~L~~R00** frames if necessary.
31. **[D]** ____ Record image names and parameters in the image Log.
32. **[T]** ____ Take digital pictures of the integrating sphere's position and the whole test/GSE set-up.
33. **[T]** ____ Visually estimate the distance from the integrating sphere and the Mastcam-Z's sunshade. This distance is approximately ~~[illegible]~~ 64 cm
34. **[T]** ____ Lights off
35. **[D, L]** Notes:

MOVE TO 110mm FOCAL LENGTH

Date ______ Time ______ Initial ______

Radiance Value 1 for the Right Mastcam-Z

SKIP

36. **[D]** ____ Record temperature information:
    - Camera CCD temp ______________ Optics temp ______________
37. **[D,T]** ____ Set the integrating sphere output to this test's radiance value defined in Table 3.
38. **[D,T]** ____ Record exact integrating sphere readout value __________ mW/cm2/sr.
39. **[T]** ____ Take time-stamped digital pictures of the setup and integrating sphere readout.
40. **[O]** ____ Insert the note "ISOP=[radiance]" and execute camera script **412TAMBR01**, which captures 10 frames for 9 exposure times with filter 0 at focus 3 m at 100mm focal length. The estimated duration is 10 minutes.
41. **[D,T]** ____ Record exact integrating sphere readout value __________ mW/cm2/sr.
42. **[D]** ____ Record image names and parameters in the Image Log.
43. **[D, L]** Notes: ____________________________________

DONE LAST NIGHT 5/1/19

Radiance Value 2 for the Right Mastcam-Z 2.0

44. **[D]** [illegible] Record temperature information:
    - Camera CCD temp 23.8°C Optics temp ______
45. **[D,T]** [illegible] Set the integrating sphere output to this test's radiance value defined in Table 3.
46. **[D,T]** [illegible] Record exact integrating sphere readout value 2.011 mW/cm2/sr.
47. ~~**[T]** ____ Take time-stamped digital pic~~tures of the setup and integrating sphere readout.
48. **[O]** [illegible] Insert the note "ISOP=[radiance]" and execute camera script **412TAMBR02**, which captures 10 frames for 9 exposure times with filter 0 at focus 3 m at ~~100~~ 110mm focal length. The estimated duration is 10 minutes.
49. **[D,T]** [illegible] Record exact integrating sphere readout value 2.022 mW/cm2/sr.
50. **[D]** [illegible] Record image names and parameters in the Image Log.
51. **[D, L]** Notes: → 89

24.0°C

Date 5/2/19 Time 21:45 Initial [illegible]

Radiance Value 3 for the Right Mastcam-Z 3.0

52. **[D]** [illegible] Record temperature information:
    - Camera CCD temp 24.0°C Optics temp ______
53. **[D,T]** [illegible] Set the integrating sphere output to this test's radiance value defined in Table 3.
54. **[D,T]** [illegible] Record exact integrating sphere readout value 3.001 mW/cm2/sr.

SKIP 55. ~~**[T]** ____ Take time-stamped digital pictures of the setup and integrating sphere readout.~~

56. **[O]** [illegible] Insert the note "ISOP=[radiance]" and execute camera script **412TAMBR03**, which captures 10 frames for 9 exposure times with filter 0 at focus 3 m at ~~100~~ 110mm focal length. The estimated duration is 10 minutes.
57. **[D,T]** [illegible] Record exact integrating sphere readout value 3.001 mW/cm2/sr.
58. **[D]** [illegible] Record image names and parameters in the Image Log.
59. **[D, L]** Notes: ______

Radiance Value 4 for the Right Mastcam-Z 4.0

60. **[D]** [illegible] Record temperature information:
    - Camera CCD temp 24.0 Optics temp ______
61. **[D,T]** [illegible] Set the integrating sphere output to this test's radiance value defined in Table 3.
62. **[D,T]** [illegible] Record exact integrating sphere readout value 4.019 mW/cm2/sr.

SKIP ~~63. **[T]** ____ Take time-stamped digital pic~~tures of the setup and integrating sphere readout.

64. **[O]** [illegible] Insert the note "ISOP=[radiance]" and execute camera script **412TAMBR04**, which captures 10 frames for 9 exposure times with filter 0 at focus 3 m at ~~100~~ 110 mm focal length. The estimated duration is 10 minutes.
65. **[D,T]** [illegible] Record exact integrating sphere readout value 4.025 mW/cm2/sr.
66. **[D]** [illegible] Record image names and parameters in the Image Log.
67. **[D, L]** Notes: PHOTON TRANSFER PLOTS LOOK GOOD.

→89

Date ______ Time ______ Initial ______

Radiance Value 5 for the Right Mastcam-Z

SKIP

68. **[D]** ____ Record temperature information:
    - Camera CCD temp ______________ Optics temp ______________
69. **[D,T]** ____ Set the integrating sphere output to this test's radiance value defined in Table 3.
70. **[D,T]** ____ Record exact integrating sphere readout value __________ mW/cm2/sr.
71. **[T]** ____ Take time-stamped digital pictures of the setup and integrating sphere readout.
72. **[O]** ____ Insert the note "ISOP=[radiance]" and execute camera script **412TAMBR05**, which captures 10 frames for 9 exposure times with filter 0 at focus 3 m at 100mm focal length. The estimated duration is 10 minutes.
73. **[D,T]** ____ Record exact integrating sphere readout value __________ mW/cm2/sr.
74. **[D]** ____ Record image names and parameters in the Image Log.
75. **[D, L]** Notes: ______________________________

DONE LAST NIGHT 5/1/19

Date 5/2 Time 22:07 Initial [initials]

Radiance Value 6 for the Right Mastcam-Z 6.0

76. **[D]** [initials] Record temperature information:
    - Camera CCD temp 24.0°C Optics temp N/A
77. **[D,T]** [initials] Set the integrating sphere output to this test's radiance value defined in Table 3.
78. **[D,T]** [initials] Record exact integrating sphere readout value 6.086 mW/cm2/sr.
79. ~~**[T]** ____ Take time-stamped digital pic~~tures of the setup and integrating sphere readout.
80. **[O]** [initials] Insert the note "ISOP=[radiance]" and execute camera script **412TAMBR06,** which captures 10 frames for 9 exposure times with filter 0 at focus 3 m at ~~100~~ 48mm focal length. The estimated duration is 10 minutes.
81. **[D,T]** [initials] Record exact integrating sphere readout value 6.088 mW/cm2/sr.
82. **[D]** [initials] Record image names and parameters in the Image Log.
83. **[D, L]** Notes: —89

Date 5/2 Time 22:17 Initial [illegible]

Radiance Value 7 for the Right Mastcam-Z 8.0

84. **[D]** [illegible] Record temperature information:
    - Camera CCD temp ~~24~~ 23.9° Optics temp N/A
85. **[D,T]** [illegible] Set the integrating sphere output to this test's radiance value defined in Table 3.
86. **[D,T]** [illegible] Record exact integrating sphere readout value 8.067 mW/cm2/sr.
87. SKIP ~~87. [T] ___ Take time-stamped digi~~tal pictures of the setup and integrating sphere readout.
88. **[O]** [illegible] Insert the note "ISOP=[radiance]" and execute camera script **412TAMBR07**, which captures 10 frames for 9 exposure times with filter 0 at focus 3 m at ~~100~~ 110 mm focal length. The estimated duration is 10 minutes.
89. **[D,T]** [illegible] Record exact integrating sphere readout value 8.062 mW/cm2/sr.
90. **[D]** [illegible] Record image names and parameters in the Image Log.
91. **[D, L]** Notes: ______

24.0°C

Date 5/2 Time 22:28 Initials [illegible]

Radiance Value 8 for the Right Mastcam-Z 10.0

92. **[D]** [illegible] Record temperature information:
    - Camera CCD temp 24.0 Optics temp N/A
93. **[D,T]** [illegible] Set the integrating sphere output to this test's radiance value defined in Table 3.
94. **[D,T]** [illegible] Record exact integrating sphere readout value 10.023 mW/cm2/sr.

SKIP ~~95. **[T]** ____ Take time-stamped~~ digital pictures of the setup and integrating sphere readout.

96. **[[redacted]]** [illegible] Insert the note "ISOP=[radiance]" and execute camera script **412TAMBR08**, which captures 10 frames for 9 exposure times with filter 0 at focus 3 m at 100mm focal length. The estimated duration is 10 minutes.
97. **[D,T]** [illegible] Record exact integrating sphere readout value 10.018 mW/cm2/sr.
98. **[D]** [illegible] Record image names and parameters in the Image Log.
99. **[D, L]** Notes: →179
    24.0°C

Data Validation

100. **[V]** [illegible] Upload data to server.
101. **[V]** [illegible] Run the Photon Transfer Jupyter notebook on the acquired data for the Right and Right Mastcam-Z. This analysis can take place while the test continues.
    - Create preliminary photon transfer curves.
    - Create preliminary flat-field images and radiometric coefficients for each filter.
    - Save results in the calibration records.
102. **[V,D, L]** Notes: DATA LOOK GOOD!

Dark Current with the Right and Right Mastcam-Zs

103. **[T]** [illegible] Turn off the integrating sphere lamp.
104. **[T]** [illegible] Turn off the lights and minimize the room's ambient light.
105. **[D]** [illegible] Record the following temperatures:
   - Camera CCD temp 23.9°C Optics temp N/A

SKIP

~~106. **[D,T]** ___ Take digital pictures of the integrating sphere's position, and the whole test/GSE set-up.~~

~~107. **[O]** Load and execute camera script **441TEMPL03**, which captures 5 dark frames through filter 7 at the exposure times 0.0, 10.0, 20.0, and 100.0 seconds. The estimated duration is 12 minutes.~~

108. **[O]** Load and execute camera script **441TEMPR03**, which captures 5 dark frames through filter 7 at the exposure times 0.0, 10.0, 20.0, and 100.0 seconds. The estimated duration is 12 minutes.
109. **[D]** [illegible] Record image names and parameters in Image Log.
110. **[T]** [illegible] Cover the integrating sphere exit port window.
111. **[D, L]** Notes: TURN LIGHTS ON

23.8°C

Shutdown Procedure

SKIP ~~112. [D,T] ____ Take digital pic~~tures of this page and the test setup.

113. [D,O] MB Review entries in Image Log, GSE command log, and image headers.
114. [D, L] MB Review calibration procedure and ensure that each task is initialed.
115. [D, L] Notes: ______________________________

116. [V, L] ~~Zu~~ Before making the decision to break down the test setup, ensure that adequate data were acquired for the test requirements. See "MastcamZCalPlan" for these requirements.
117. [V] Notes: ALL PLOTS GOOD EXCEPT LEFT CAMERA RADIANCE LEVEL 6. DARK CURRENT SAME WHEN LAMP ON AS WHEN OFF.

Data Validator (signature) [signature]

Date 5.2.19 Time 11:00

118. [V, L] ~~Zu~~ Give the go/no-go decision. Have enough data been acquired to fulfill test requirements? See "MastcamZCalPlan" for these requirements.
119. [D, L] MB Update the Log Document.
120. [L] Notes: ______________________________

Calibration Lead (signature) [signature]

Date 5/2/19 Time 23:01

121. **[O, L]** KW Ensure that the camera and GSE are in a safe state.
122. **[O,D]** KW Review the Image Log with the documentarian. Exchange high-fives.
123. **[O]** Notes: CAMERAS HOMED, POWER OFF

Camera Operator (signature) [signature]

Date 5/02/19 Time 11:04 PM

124. **[T]** [initials] If the next test does not require the integrating sphere, position it away from the chamber or bench. Otherwise, be sure not to move it. The next test is JPL Geometric
125. **[T]** [initials] Ensure that all other test equipment is safely put away.
126. **[T]** Notes:

Technician (signature) [signature]

Date 5/2/19 Time 23:00

127. **[D, L]** MB Double-check this procedure and ensure that the top of each page is initialed with the time and date.
128. **[D]** MB Photo-scan this document, save it on the cloud, and file the hardcopy in the Log Binder. Upload the digital pictures taken during this test in the appropriate archive on the cloud. The required links are on the Wiki. (later)
129. **[D]** MB Double-check that every required cell the Image Log is accurately filled. When this is complete, print the Image Log and file it the Log Binder after this document.
130. **[D]** Notes:

Documentarian (signature) Megan Barrington

Date 2 May 2019 Time 10:59 pm
