# Supplementary material for: Pre-Flight Calibration of the Mars 2020 Rover Mastcam Zoom (Mastcam-Z) Multispectral, Stereoscopic Imager
Source: Space Sci Rev. 2021 Feb 18;217(2):29. doi: 10.1007/s11214-021-00795-x (PMC7892537; doi:10.1007/s11214-021-00795-x)
Supplement: Supplementary file 1 — (ZIP 98.6 MB) [file 11214_2021_795_MOESM1_ESM.zip › CalPro_423_Radiometric_v2_04.pdf]

Radiometric Calibration Procedure for Mastcam-Z Ambient TVAC Testing at MSSS (Pro. 4.2.3)

[Procedure version 2.04, prepared by the Mastcam-Z calibration team at Cornell University]

These measurements are performed on the camera and at the temperature designated below as specified in the Mastcam-Z Calibration Plan,

Unit Under Test:

Left FM X **Right FM** X **EQM**_____ **Other**_____

These measurements are performed at temperature:

-35° C_____ **-10°C** X **+5°C**_____ **Ambient**_____ **Other**_____

These measurements are performed at,

MSSS X **ASU**_____ **Other**_____

Date 4/29 **Start Time** 18:~~[illegible]~~20 **End Time** 23:00

Estimated Duration 4.5 hours

Scheduled Start Time 18:00 **Sch. End Time** ~~[illegible]:00~~ 22:00

Calibration Lead [L] HERKENHOFF **Documentarian [D]** CORLIES

Camera Operator [O] VAN BEEK, DIXON **Technician [T]** WINHOLD

Data Validator [V] TATE **Other** _____

Date 4/29 Time 18:00 Initials [signature]

Change Log

Version	Name	Change
v1_01<br>17 Sep 2018	C. Tate	(first draft)
v1_20<br>1 Nov 2018	C. Tate	Procedure edits prior to EQM testing
v1_23<br>10 Dec. 2018	C. Tate	Procedure edits after EQM testing
v2_0~~3~~4<br>29 April 2019	C. Tate	Approved version prior to FM testing

Document Approval

Approved by James Bell — Date
Mastcam-Z PI
Arizona State University

[signature] 5/6/19
Approved by Alexander Hayes — Date
Mastcam-Z Calibration Working Group
Lead, Cornell University

Approved by Justin Maki — Date
Mastcam-Z Deputy PI and Investigation
Scientist, Jet Propulsion Laboratory

[signature] 4/29
Approved by Christian Tate — Date
Procedure Author
Cornell University

[signature]
Approved by — Date
Kenneth Herkenhoff — 4-29-19

Table of Contents

RADIOMETRIC CALIBRATION PROCEDURE FOR MASTCAM-Z AMBIENT TVAC TESTING AT MSSS (PRO. 4.2.3) 1

CHANGE LOG 2
DOCUMENT APPROVAL 2
TEST DESCRIPTION 4
SOFTWARE PREPARATION 4
Table 1. File naming convention for the camera script prefixes and frame filenames: "AAABBBBCDD" 4
HARDWARE INSTALLATION 6
Figure 1. ASU Floor Plan for Geometric Testing in the TVAC Chamber. The MSSS Floor Plan allows for similar target and source placements relative to the chamber window. 6
Table 2. The Nominal Radiance Values (calibrated integrating sphere output). 8

RIGHT AND LEFT MASTCAM-Z TESTS 9

CENTER THE INTEGRATING SPHERE .... 9 (Darks)
RADIANCE VALUE 1 FOR THE RIGHT MASTCAM-ZS 11
RADIANCE VALUE 1 FOR THE LEFT MASTCAM-ZS 12
RADIANCE VALUE 2 FOR THE LEFT MASTCAM-Z 13
RADIANCE VALUE 2 FOR THE RIGHT MASTCAM-Z 14
DATA VALIDATION .... 15 (Darks)

SHUTDOWN PROCEDURE 17

Test Description

Excerpt from the Calibration Plan 4.2,

> The objectives of these tests are to derive flat field images as well as the coefficients to allow a conversion from reduced (bias, dark, and flat field corrected) DN/s to absolute radiometric response (W/cm^2/sr per filter) for (a) the R, G, and B microfilters of the Bayer Pattern Filter detectors in each camera head (clear filter), (b) the 14 non-solar Mastcam-Z spectral filters "Science Filters", and, if time permits, (c) the two Mastcam-Z neutral density solar filters; and to provide an estimate of the uncertainty in these coefficients and, at Priority 2, their temperature dependence. This test builds off the Section 4.3 – Spectral Throughput Calibration to accurately account for the filter spectral response in the conversion. The requirement of knowing the relative response on the shape of the spectral throughput to ±5% combined with the absolute Radiance accuracy of the integration sphere at ±5% still allows the ±10% absolute radiometric calibration requirement to be met.

Software Preparation

The software and files required for this test are prepared well in advance of test day. This checklist ensures that the following are present, debugged, and executable: (1) all fast-look scripts, (2) automated header generation of all relevant camera parameters, target positioning, and metadata, (3) all camera scripts that command the camera unit, and (4) the directories/file-paths pointing to the data repositories of this specific test.

Table 1. File naming convention for the camera script prefixes and frame filenames: "AAABBBBCDD"

Code	Name	Example	Value
"AAA"	Calibration Plan Section	"411" = Cal. Plan 4.1.1 chapter 4, section 1, subsection 1	423
"BBBB"	Location of test or ASU Chamber temperature	"MSSS" = test at MSSS, "TN10" = ASU TVAC -10C, …	TAMB
"C"	Camera unit under test	"L" = Left Mastcam-Z, "R" = Right Mastcam-Z, "E" =EQM, "C" =COTS	R/L
"DD"	Part of test (radiance value)	"00" = test set up, "01" = first radiance value …	00-08

Date 4/29 Time 18:20 Initial [illegible]

1. **[D]** ✓ Look up the daily calibration schedule and record the scheduled start and end time of this test on the cover page of this document. Also fill out and double-check the other information on the cover page.
2. **[D]** CX Ensure that all supplemental manuals are on hand. These are,
   - ~~Labsphere_Manual,~~ [illegible]
   - Validator_Manual, Documentarian_Manual
   - MastcamZCalPlan
3. **[D]** ✓ Ensure that the Image Log is present and ready to use. Find and open the Google Sheets file "Image_Log_42". There is a link on the Wiki.
4. **[V]** [illegible] Check that all Calgorithms fast-look and validation scripts are present, up-to-date, and ready to analyze test output. Find and open the "Radiometric_Calibration_42_Validation" Jupyter notebook. There is a link on the Wiki.
5. **[O]** [illegible] Check that all camera scripts required for this test are present, up-to-date and ready to command the ground support equipment (GSE). These are,
   - **412TAMBR00 - 412TAMBR08** and **423TAMBR00 - 423TAMBR06**
   - **412TAMBL00 - 412TAMBL08** and **423TAMBL00 - 423TAMBL06**
   - **441TEMPR03 and 441TEMPL03**
6. **[O,V,D, L]** Notes:

ADDED DARK CURRENT SEQUENCE BEFORE THIS RADIOMETRIC TEST DUE TO SLOWLY CHANGING CAMERA TEMPERATURES. GOAL IS TO ALLOW RADIOMETRY IMAGES TO BE DARK-CORRECTED VIA INTERPOLATION OF DATA ACQUIRED BEFORE AND AFTER RADIOMETRY.

Hardware Installation

This procedure is for the ambient TVAC chamber testing at MSSS. Figure 1 shows the nominal layout of the TVAC chamber, workspace, Mastcam-Zs, ground support equipment (GSE), targets, sources, and other equipment necessary for this test if it happens at ASU. Although MSSS' cleanroom is different than ASU's, the placement of the targets and sources relative to the chamber window is similar.

Figure 1. ASU Floor Plan for Geometric Testing in the TVAC Chamber. The MSSS Floor Plan allows for similar target and source placements relative to the chamber window.

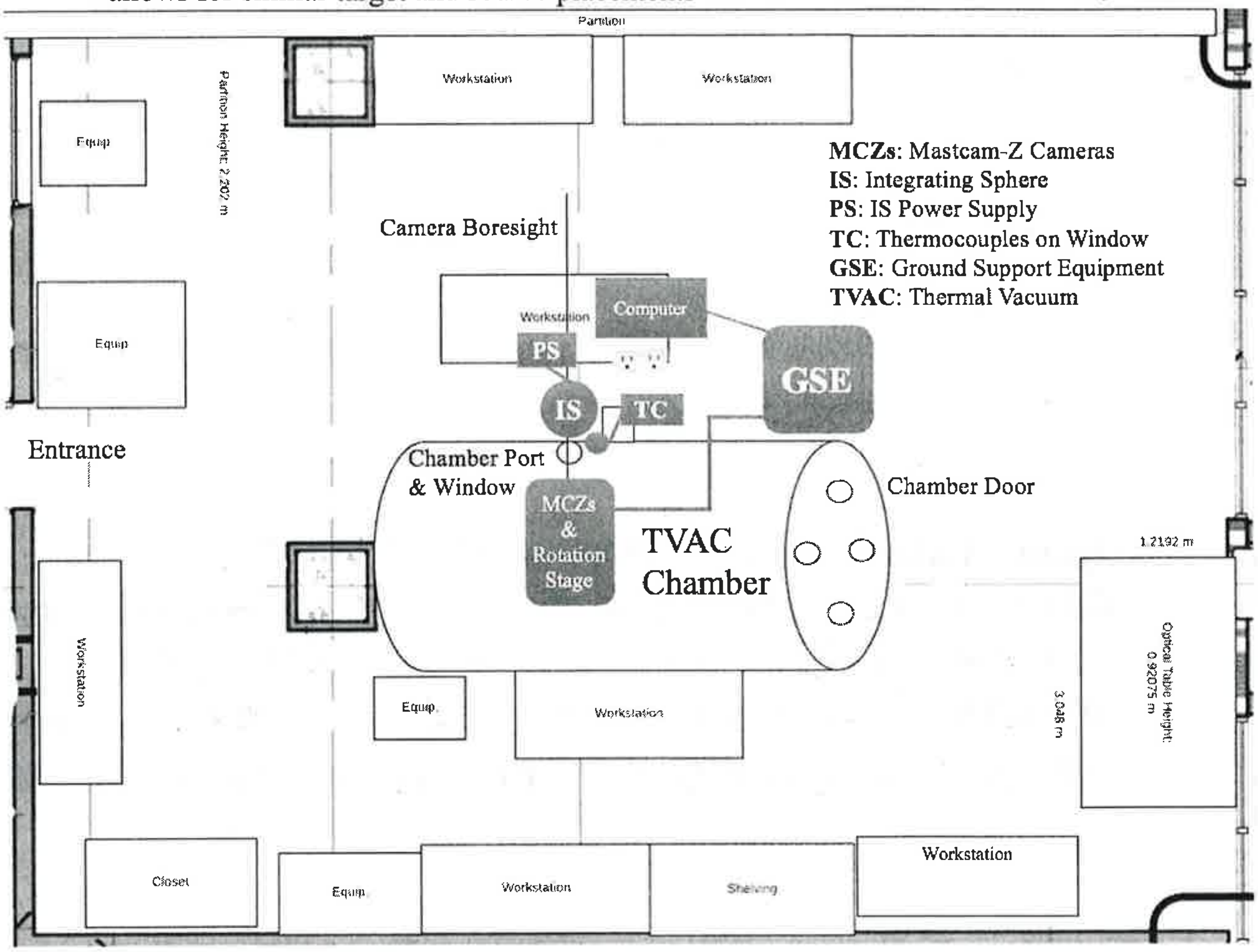

7. [T,O, L] ___ Ensure that all personnel in the cleanroom are following the cleanroom practices for electrostatic discharge, proper clothing and other safety concerns.
8. [T] ___ Double check that nitrogen is flowing over the Mastcam-Zs or the window port.

9. **[O,T]** ____ If not already done, mate the Right and Left Mastcam-Zs into the GSE. Follow the procedure in "MastcamZ_GSE_Manual".
10. **[T]** ____ Verify that the thermocouples are turned on and properly reading out.
11. **[O,T]** ____ Ensure that the camera unit and GSE wires are secure, kink-free, and do not present tripping hazards when the lights are turned off.
12. **[[redacted],D]** ____ Check the camera temperature and ensure nominal operation.
13. **[D]** ____ Record the following environmental information:
    - Cleanroom temperature 24.8 pressure ~~24.8~~ humidity 44
14. **[O,D, L]** Notes:

____

____

____

15. **[D,T]** ____ Take time-stamped pictures of this page, the integrating sphere, and the whole test/GSE set-up.
16. **[T]** ____ Power on the integrating sphere. Follow the procedure in "Labsphere_Manual". Record the time the lamp is turned on 18:17.
17. **[D,T]** ____ Record the exact readout value of the integrating sphere's radiance: -0.1479 mW/cm2/sr.
18. **[T,O, L]** ____ Confirm that the camera systems and GSEs are powered on and ready for use. Follow the procedure in "MastcamZ_GSE_Manual".
19. **[D, L]** Notes:

ABOVE RECORDED DURING DARK

____

____

Table 2. The Nominal Radiance Values (calibrated integrating sphere output).

**IS Output Radiances**	**Nominal Radiance [mW/cm2/sr]**
Radiance 1	5.0
Radiance 2	10.0

Date 4/29 Time 8:22 Initial [illegible]

Right and Left Mastcam-Z Tests

Dark Current with the Right and Left Mastcam-Zs

20. **[T]** ___ Cover the port window and turn off the lights.
21. **[D]** ___ Record the following temperatures:
    i. Chamber temp ambient Port temp N/A
    ii. Camera CCD temp R: 30.2° L: 29.6 °C Optics temp N/A
22. **[D,T]** ___ Take digital pictures of the geometric target's position, and the whole test/GSE set-up.
23. **[O]** Load and execute camera script **441TEMPR03**, which captures 5 dark frames through filter 7 at the exposure times 0.0, 10.0, 20.0, and 100 seconds. The estimated duration is 10 minutes.
24. **[O]** Load and execute camera script **441TEMPL03**, which captures 5 dark frames through filter 7 at the exposure times 0.0, 10.0, 20.0, and 100 seconds. The estimated duration is 10 minutes.
25. **[D]** ___ Record image names and parameters in Image Log.
26. **[T]** ___ Uncover the port window.
27. **[D, L]** Notes: LAST SUFFIX 88 FOR BOTH CAMERAS

Center the Integrating Sphere

28. **[T]** ___ Move integrating sphere output as close to the chamber window as possible centered on the Right Mastcam-Z boresight. ~1 cm between
29. **[T]** ___ Turn off lights. LIGHTS NEXT DOOR ON, ILLUMINATING CHAMBER
30. **[O]** ___ Insert the note "ISOP=[radiance]" and execute camera script **423TAMBR00**. This script captures one auto-exposure at 40% full-well and one bias frame for filter 0 at 26 mm focal length. 4.978 $mW cm^{-2} sr^{-1}$

31. **[V,O,T]** [illegible] Open images, and if the images show that the integrating sphere is not centered, center the integrating sphere disc in the frame. Recapture **423TAMBR00** frames if necessary.
32. **[D]** [illegible] Record image names and parameters in the image Log.
33. **[O,D,V]** [illegible] Approximate the full-well percentage of the center pixels of the image and verify that they are about 40% (or DN 155). SKIP
34. **[T]** [illegible] Lights off
35. **[D, L]** Notes:

Date 4/29 Time 9:02 Initial [illegible]

Radiance Value 1 for the Right Mastcam-Zs

36. **[T]** [initials] Set integrating sphere output to the radiance value 1 defined in Table 2.
37. **[D,T]** [initials] Record exact integrating sphere readout value 4.9863 mW/cm2/sr.
38. **[D]** [initials] Record temperature information:
    - Chamber temp ambient Port temp ~~[illegible]~~ N/A
    - Camera CCD temp 30.9°C Optics temp N/A
39. **[D,T]** [initials] Take time-stamped digital pictures of the setup and integrating sphere readout.
40. **[O]** [initials] Insert the note "ISOP=[radiance]" and execute camera script **423TAMBR02**, which captures 5 frames for ~~40% and~~ TBC 80% full-well and ~~10 bias frames~~ TBC with the 7 non-solar filters at three focal lengths. The estimated duration is 40 minutes.
41. **[D,T]** [initials] Record exact integrating sphere readout value 4.9863 → 4.9888 INTERMITTENTLY mW/cm2/sr.
42. **[O]** [initials] Insert the note "ISOP=[radiance]" and execute camera script **41~~3~~2TAMBR05**, which captures 10 frames for 9 exposure times with filter 0 at 100mm focal length. The estimated duration is 10 minutess.
43. **[D,T]** ____ Record exact integrating sphere readout value 4.9888 mW/cm2/sr.
44. **[D]** [initials] Record image names and parameters in the Image Log.
45. **[D, L]** Notes: FOCAL LENGTHS = 34, 63, 100 mm
DECIDED TO GO TO RADIANCE VALUE 2 FOR RIGHT EYE BEFORE MOVING TO LEFT ~~EE~~ EYE

LEFT TEST IMAGE WITH SPHERE = 4.9838 mWcm⁻²sr⁻¹

RIGHT EYE SPHERE IMAGES NOT PERFECTLY CENTERED, BUT DEEMED CLOSE ENOUGH.

RAN LEFT EYE PHOTON TRANSFER SERIES RUN DURING RT. RAD.
→ 412TAMBLØ5, WITH INITIAL FLUX OF 4.9888 mW/cm²/sr, EVEN THOUGH SPHERE OUTPUT NOT WELL CENTERED: STEPS 56-59
→ ALSO 412TAMBLO8 AT RADIANCE 2.
GO TO PG. ~~[illegible]~~ 14.

Date 4/29 Time 21:00 Initial ____

Radiance Value 1 for the Left Mastcam-Zs

46. **[T]**____ Pull the integrating sphere away from the cameras and point it into a dark corner of the room before taking another radiance reading. Record exact integrating sphere readout value 4.9514 mW/cm2/sr.
47. **[T]**____ Center the integrating sphere for the Left Mastcam-Z. Do not change the radiance value.
48. **[O]**____ Insert the note "ISOP=[radiance]" and execute camera script **423TAMBL00**. This script captures one auto-exposure at 40% full-well and one bias frame for filter 0 at 26 mm focal length.
49. **[V,O,T]**____ Open images, and if the images show that the integrating sphere is not centered, center the integrating sphere disc in the frame. Recapture **423TAMBL00** frames if necessary.
50. **[D]**____ Record image names and parameters in the image Log.
51. **[D,T]**____ Record exact integrating sphere readout value ~~[illegible]~~ 5.0013 mW/cm2/sr.
52. **[D]**____ Record temperature information:
    - Chamber temp ambient Port temp N/A
    - Camera CCD temp ~~29.8°~~ 29.8° Optics temp N/A
53. **[D,T]**____ Take time-stamped digital pictures of the setup and integrating sphere readout.
54. **[[redacted]]**____ Insert the note "ISOP=[radiance]" and execute camera script **423TAMBL02**, which captures 5 frames for 40% and 80% full-well and 10 bias frames with the 7 non-solar filters at three focal lengths. The estimated duration is 40 minutes.
55. **[D,T]**____ Record exact integrating sphere readout value 5.0187 mW/cm2/sr.
56. **[O]**____ Insert the note "ISOP=[radiance]" and execute camera script **41~~3~~2TAMBL05**, which captures 10 frames for 9 exposure times with filter 0 at 100mm focal length. The estimated duration is 10 minutess.
57. **[D,T]**____ Record exact integrating sphere readout value ________ mW/cm2/sr.
58. **[D]**____ Record image names and parameters in the Image Log.
59. **[D, L]** Notes: IMAGE NOT WELL CENTERED, TAKEN DURING RT. RADIOMETRY

Date 4/29 Time ~~20:03~~ 20:40 Initial [illegible]

____

____

Radiance Value 2 for the Left Mastcam-Z

SKIP 60. **[T]** ~~Set~~ integrating sphere output to the radiance value 2 defined in Table 2.

61. **[D,T]** ___ Record exact integrating sphere readout value 9.9183 mW/cm2/sr.

62. **[D]** ___ Record temperature information:
    - Chamber temp ambient Port temp N/A
    - Camera CCD temp 29.8°C Optics temp N/A

63. **[D,T]** ___ Take time-stamped digital pictures of the setup and integrating sphere readout.

64. **[O]** ___ Insert the note "ISOP=[radiance]" and execute camera script **423TAMBL02**, which captures 5 frames for 40% and 80% full-well and 10 bias frames with the 7 non-solar filters at three focal lengths. The estimated duration is 40 minutes.

65. **[D,T]** ___ Record exact integrating sphere readout value 9.9976 mW/cm2/sr.

RAN IN PARALLEL WITH RT. RADIOMETRY @ RAD. VALUE 2. → 66. **[O]** ___ Insert the note "ISOP=[radiance]" and execute camera script **41~~3~~2TAMBL08**, which captures 10 frames for 9 exposure times with filter 0 at 100mm focal length. The estimated duration is 10 minutess.

→ 67. **[O]** ___ If time permits, insert the note "ISOP=[radiance]" and execute camera script **423TAMBL05**, which captures 5 frames for 80% ~~and 80%~~ full-well ~~and 5 bias frames~~ with filters 0 and 1 at seven focal lengths. These images are compa~~ou~~nded at 8-bits. The estimated duration is 12 minutes.

68. **[D,T]** ___ Record exact integrating sphere readout value 10.003 mW/cm2/sr.

69. **[D]** ___ Record image names and parameters in the Image Log.

70. **[D, L]** Notes: GO TO STEP 47, THEN 61. WHEN CENTERED INSERTED RT. CAMERA STEPS 81

____

Date 4/29 Time 20:03 Initial [illegible]

Radiance Value 2 for the Right Mastcam-Z

SKIP

71. **[T]** ____ Pull the integrating sphere away from the cameras and point it into a dark corner of the room before taking another radiance reading. Record exact integrating sphere readout value __________ mW/cm2/sr.
72. **[T]** ✓ Center the integrating sphere for the Right Mastcam-Z. Do not change the radiance value. ALREADY DONE

SKIP, RAN LATER

73. **[O]** ____ Insert the note "ISOP=[radiance]" and execute camera script **423TAMBR00**. This script captures one auto-exposure at 40% full-well and one bias frame for filter 0 at 26 mm focal length.
74. **[V,O,T]** ____ Open images, and if the images show that the integrating sphere is not centered, center the integrating sphere disc in the frame. Recapture **423TAMBR00** frames if necessary.
75. **[D]** ____ Record image names and parameters in the image Log.
76. **[D,T]** [initials] Record exact integrating sphere readout value 10.005 mW/cm2/sr.
77. **[D]** [initials] Record temperature information:
    - Chamber temp ambient Port temp N/A
    - Camera CCD temp 30.6 C Optics temp N/A

SKIP

78. **[D,T]** [initials] Take time-stamped digital pictures of the setup and integrating sphere readout.
79. **[O]** [initials] Insert the note "ISOP=[radiance]" and execute camera script **423TAMBR02**, which captures 5 frames for 40% and 80% full-well and 10 bias frames with the 7 non-solar filters at three focal lengths. The estimated duration is 40 minutes.
80. **[D,T]** [initials] Record exact integrating sphere readout value 10.003 mW/cm2/sr.
81. **[O]** [initials] Insert the note "ISOP=[radiance]" and execute camera script **41~~3~~2TAMBR08**, which captures 10 frames for 9 exposure times with filter 0 at 100mm focal length. The estimated duration is 10 minute~~s~~s.
82. **[O]** [initials] If time permits, insert the note "ISOP=[radiance]" and execute camera script **423TAMBR05**, which captures 5 frames for ~~40% and~~ 80% full-well ~~and 5 bias frames~~

RADIANCE = 9.9202

with filters 0 and 1 at seven focal lengths. These images are compounded at 8-bits. The estimated duration is 12 minutes.

83. **[D,T]** [illegible] Record exact integrating sphere readout value 9.9202 mW/cm2/sr.
84. **[D]** ____ Record image names and parameters in the Image Log.
85. **[D, L]** Notes: DONE DURING LEFT EYE RADIOMETRY

Data Validation

86. **[T]** CB Lights on
87. **[V]** CB Upload data to server.
88. **[V]** CB Run the "Radiometric_Calibration_42_Validation" Jupyter notebook on the acquired data for the Right ✓ and Left ✓ Mastcam-Z with the window off. This analysis can take place while the test continues.
    - Create preliminary flat-field images and radiometric coefficients for each filter.
    - Save results in the calibration records.
89. **[V,D, L]** Notes: The Right flats and photon transfer curves look good. [illegible]

Date 4/29 Time 22:37 Initial [illegible]

Dark Current with the Right and Left Mastcam-Zs

90. **[T]** ___ Cover the port window and turn off the lights.
91. **[D]** ___ Record the following temperatures:
    iii. Chamber temp ambient Port temp N/A
    iv. Camera CCD temp L: 29.5, R: 30.1 Optics temp N/A
92. **[D,T]** ___ Take digital pictures of the geometric target's position, and the whole test/GSE set-up.
93. **[O]** Load and execute camera script **441TEMPR03**, which captures 5 dark frames through filter 7 at the exposure times 0.0, 10.0, 20.0, and 100 seconds. The estimated duration is 10 minutes.
94. **[O]** Load and execute camera script **441TEMPL03**, which captures 5 dark frames through filter 7 at the exposure times 0.0, 10.0, 20.0, and 100 seconds. The estimated duration is 10 minutes.
95. **[D]** ___ Record image names and parameters in Image Log.
96. **[T]** ___ Uncover the port window.
97. **[D, L]** Notes: L: 89
    R: 89

Shutdown Procedure

98. **[D,T]** CX Take digital pictures of this page and the test setup.
99. **[D,O]** CX Review entries in Image Log, GSE command log, and image headers.
100. **[D, L]** ____ Review calibration procedure and ensure that each task is initialed.
101. **[D, L]** Notes: DRY N PURGE TURNED BACK ON.

102. **[V, L]** [illegible] Before making the decision to break down the test setup, ensure that adequate data were acquired for the test requirements. See "MastcamZCalPlan" for these requirements.
103. **[V]** Notes: ____

Data Validator (signature) [illegible signature]

Date 4-29-19 Time 10:54

104. **[V, L]** [illegible] Give the go/no-go decision. Have enough data been acquired to fulfill test requirements? See "MastcamZCalPlan" for these requirements.
105. **[D, L]** [illegible] Update the Log Document.
106. **[L]** Notes: UPDATED TAG-UP START TIME TO 8:30 PDT TOMORROW.

Calibration Lead (signature) [illegible signature]

Date 4/29/19 Time 23:01

107. [[redacted], L] ____ Ensure that the camera and GSE are in a safe state.
108. **[O,D]** ____ Review the Image Log with the documentarian. Exchange high-fives.
109. **[O]** Notes: No Concerns

Camera Operator (signature) ______________

Date 4-29-19 Time 11:02

110. **[T]** ____ If the next test does not require the integrating sphere, position it away from the chamber or bench. Otherwise, be sure not to move it. The next test is MTF.
111. **[T]** ____ Ensure that all other test equipment is safely put away.
112. **[T]** Notes: GN2 VALVE TURNED 1/4 TURN CW SO THE VALVE WAS ⊥ TO WALL. GAS COULD BE HEARD FLOWING ~11PM (HAD BEEN CLOSED ~6PM FOR TESTING) FOR CHAMBER

Technician (signature) ______________

Date 4/29/19 Time 23:10

113. **[D, L]** ____ Double-check this procedure and ensure that the top of each page is initialed with the time and date.
114. **[D]** ____ Photo-scan this document, save it on the cloud, and file the hardcopy in the Log Binder. Upload the digital pictures taken during this test in the appropriate archive on the cloud. The required links are on the Wiki.
115. **[D]** ____ Double-check that every required cell the Image Log is accurately filled. When this is complete, print the Image Log and file it the Log Binder after this document.
116. **[D]** Notes: ______________

Documentarian (signature) ______________

Date 4-29-19 Time 10:57
