# Supplementary material for: Pre-Flight Calibration of the Mars 2020 Rover Mastcam Zoom (Mastcam-Z) Multispectral, Stereoscopic Imager
Source: Space Sci Rev. 2021 Feb 18;217(2):29. doi: 10.1007/s11214-021-00795-x (PMC7892537; doi:10.1007/s11214-021-00795-x)
Supplement: Supplementary file 1 — (ZIP 98.6 MB) [file 11214_2021_795_MOESM1_ESM.zip › CalPro_423_Radiometric_v2_04_sup.pdf]

Re prin[illegible]

~~Shutdown Procedure~~

—

I. S. - Window reflection effect.

4-27-19

5.333	4:24
5.323	4:29
5.332	4:32
5.196	
5.193	2.4% drop
5.198	
5,193	
5.318	4:36
5.323	4:38
5.321	4:40
5.318	~~5:~~ 4:54
5.311	5:01
5.313	5:05
10.029	5:40
10.287	6:17
10.28	6:48
9.985	3.1%
9.985	6:50
10.304	
10.30	
10.302	7:25
10.297	7:48
10.292	8:00
10.0299	9
9.9801	
9.9776	

[illegible]age headers.
[illegible] initialed.

[illegible]nsure that [illegible]CalPlan" for these not in

I.S. looking into the dark room (i.e. not in front of the window)

Data Validator (sig[illegible]

Date ____

7. **[V, L]** ____ Give the go/[illegible]ired to fulfill test requirements? See "Mast[illegible]
8. **[D, L]** ____ Update the L[illegible]
9. **[L]** Notes: ____

Calibration Lead [illegible]

Date ____
