# Supplementary material for: Pre-Flight Calibration of the Mars 2020 Rover Mastcam Zoom (Mastcam-Z) Multispectral, Stereoscopic Imager
Source: Space Sci Rev. 2021 Feb 18;217(2):29. doi: 10.1007/s11214-021-00795-x (PMC7892537; doi:10.1007/s11214-021-00795-x)
Supplement: Supplementary file 1 — (ZIP 98.6 MB) [file 11214_2021_795_MOESM1_ESM.zip › CalPro_423_Radiometric_v2_07.pdf]

Radiometric Calibration Procedure for Mastcam-Z Ambient Testing at MSSS (Pro. 4.2.3)

[Procedure version 2.07, prepared by the Mastcam-Z calibration team at Cornell University]

These measurements are performed on the camera and at the temperature designated below as specified in the Mastcam-Z Calibration Plan,

Unit Under Test:

Left FM X **Right FM** X **EQM**____ **Other**________

These measurements are performed at temperature:

-35° C____ **-10°C** X **+5°C**____ **Ambient**____ **Other**________

These measurements are performed at,

MSSS X **ASU**____ **Other**____

Date 5/6/2019 **Start Time** 7:30 **End Time** 17:30

Estimated Duration 5.0 hours

Scheduled Start Time 7:30 **Sch. End Time** 15:30

Calibration Lead [L] Jim Bell, Melissa Rice, Alex Hayes **Documentarian [D]** Alexis Parkinson

Camera Operator [O] Tex **Technician [T]** Elsa, Christian

Data Validator [V] Tina Seeger, Briony Horgan **Other** ________

Date 5/6 Time 8a Initials CT

Change Log

Version	Name	Change
v1_01<br>17 Sep 2018	C. Tate	(first draft)
v1_20<br>1 Nov 2018	C. Tate	Procedure edits prior to EQM testing
v1_23<br>10 Dec. 2018	C. Tate	Procedure edits after EQM testing
v2_07<br>6 May 2019	C. Tate	Approved version prior to FM testing

Document Approval

5/6/19
Approved by James Bell Date
Mastcam-Z PI
Arizona State University

05/06/19
Approved by Alexander Hayes Date
Mastcam-Z Calibration Working Group Lead, Cornell University

Approved by Justin Maki Date
Mastcam-Z Deputy PI and Investigation Scientist, Jet Propulsion Laboratory

5/6/19
Approved by Ken Herkenhoff Date
Mastcam-Z Co-Investigator
USGS

5/6/19
Approved by Date

5/6/19
Approved by Christian Tate Date
Procedure Author
Cornell University

Date 5/6 Time 8am Initial [illegible]

Table of Contents

RADIOMETRIC CALIBRATION PROCEDURE FOR MASTCAM-Z AMBIENT TESTING AT MSSS (PRO. 4.2.3) **1**

Change Log 2
Document Approval 2
Test Description 4
Software Preparation 4
Table 1. File naming convention for the camera script prefixes and frame filenames: "AAABBBBCDD" *4*
Hardware Installation 6
Figure 1. Floor Plan for Radiometric Testing in the MSSS cleanroom. *6*
Table 2. The Nominal Radiance Values (calibrated integrating sphere output). *8*

LEFT AND RIGHT MASTCAM-Z TESTS **9**

Dark Current with the Right and Left Mastcam-Zs 9
Center the Integrating Sphere on the Left Mastcam-Z 10
Radiance Value 1 for the Left Mastcam-Z 11
Radiance Value 2 for the Left Mastcam-Z 12
Data Validation 13
Center the Integrating Sphere on the Right Mastcam-Z 14
Radiance Value 2 for the Right Mastcam-Z 15
Radiance Value 1 for the Right Mastcam-Z 16
Data Validation 17
Dark Current with the Right and Left Mastcam-Zs 18

SHUTDOWN PROCEDURE **19**

Test Description

Excerpt from the Calibration Plan 4.2,

> The objectives of these tests are to derive flat field images as well as the coefficients to allow a conversion from reduced (bias, dark, and flat field corrected) DN/s to absolute radiometric response ($W/cm^2/sr$ per filter) for (a) the R, G, and B microfilters of the Bayer Pattern Filter detectors in each camera head (clear filter), (b) the 14 non-solar Mastcam-Z spectral filters "Science Filters", and, if time permits, (c) the two Mastcam-Z neutral density solar filters; and to provide an estimate of the uncertainty in these coefficients and, at Priority 2, their temperature dependence. This test builds off the Section 4.3 – Spectral Throughput Calibration to accurately account for the filter spectral response in the conversion. The requirement of knowing the relative response on the shape of the spectral throughput to ±5% combined with the absolute Radiance accuracy of the integration sphere at ±5% still allows the ±10% absolute radiometric calibration requirement to be met.

Software Preparation

The software and files required for this test are prepared well in advance of test day. This checklist ensures that the following are present, debugged, and executable: (1) all fast-look scripts, (2) automated header generation of all relevant camera parameters, target positioning, and metadata, (3) all camera scripts that command the camera unit, and (4) the directories/file-paths pointing to the data repositories of this specific test.

Table 1. File naming convention for the camera script prefixes and frame filenames: "AAABBBBCDD"

Code	Name	Example	Value
"AAA"	Calibration Plan Section	"411" = Cal. Plan 4.1.1 chapter 4, section 1, subsection 1	423
"BBBB"	Location of test or ASU Chamber temperature	"MSSS" = test at MSSS, "TN10" = ASU TVAC -10C, …	TAMB
"C"	Camera unit under test	"L" = Left Mastcam-Z, "R" = Right Mastcam-Z, "E" =EQM, "C" =COTS	R/L
"DD"	Part of test (radiance value)	"00" = test set up, "01" = first radiance value …	00-08

Date 5/6 Time 9:00 Initial MK

1. **[D]** MK Look up the daily calibration schedule and record the scheduled start and end time of this test on the cover page of this document. Also fill out and double-check the other information on the cover page.
2. **[D]** MK Ensure that all supplemental manuals are on hand. These are,
   - ~~Labsphere_Manual,~~
   - Validator_Manual, Documentarian_Manual
   - MastcamZCalPlan
3. **[D]** MK Ensure that the Image Log is present and ready to use. Find and open the Google Sheets file "Image_Log_42". There is a link on the Wiki.
4. **[V]** MK Check that all Calgorithms fast-look and validation scripts are present, up-to-date, and ready to analyze test output. Find and open the "Radiometric_Calibration_42_Validation" Jupyter notebook. There is a link on the Wiki.
5. **[O]** MK Check that all camera scripts required for this test are present, up-to-date and ready to command the ground support equipment (GSE). These are,
   - **412TAMBR00 - 412TAMBR08** and **423TAMBR00 - 423TAMBR06**
   - **412TAMBL00 - 412TAMBL08** and **423TAMBL00 - 423TAMBL06**
   - **441TEMPR03 and 441TEMPL03**
6. **[O,V,D, L]** Notes:

____

____

____

Date 5/6 Time 9:00 Initial MR

Hardware Installation

This procedure is for the ambient cleanroom testing at MSSS. Figure 1 shows the nominal layout of the cleanroom chamber, workspace, Mastcam-Zs, ground support equipment (GSE), targets, sources, and other equipment necessary.

Figure 1. Floor Plan for Radiometric Testing in the MSSS cleanroom.

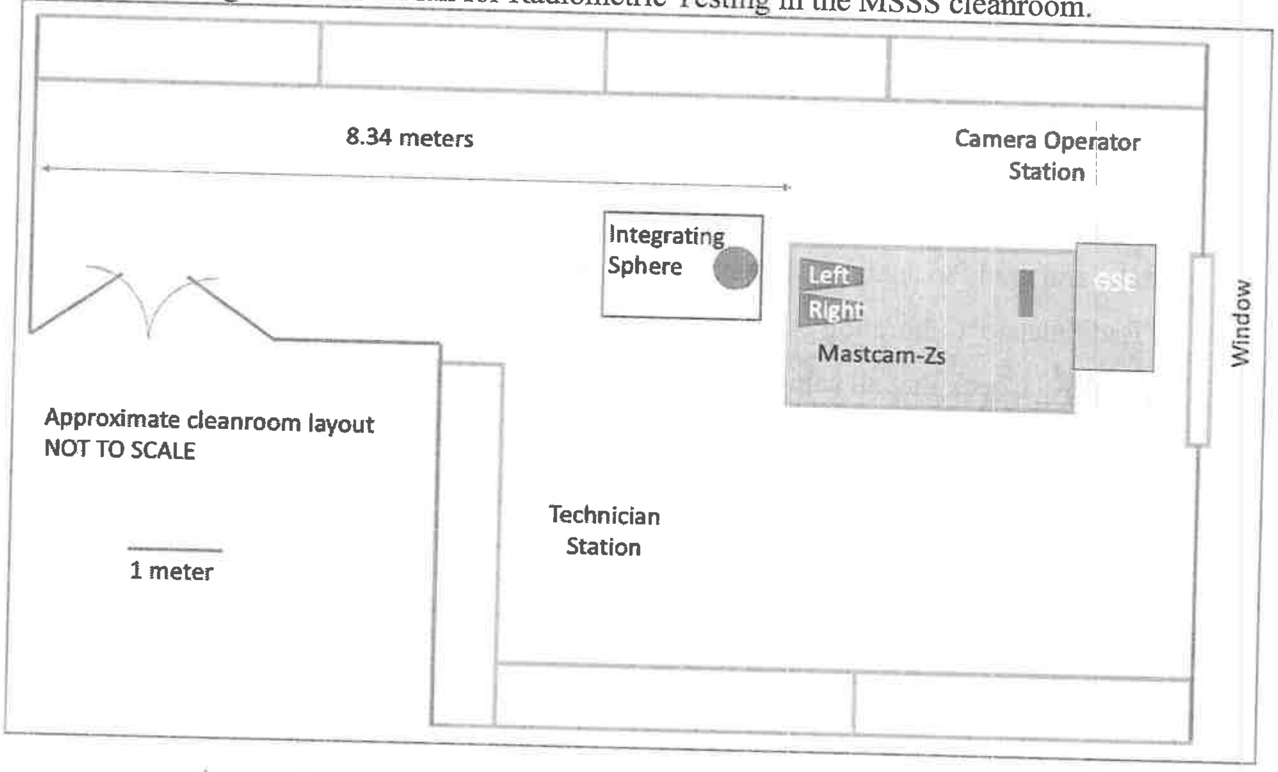

7. **[T,O, L]** MR Ensure that all personnel in the cleanroom are following the cleanroom practices for electrostatic discharge, proper clothing and other safety concerns.
8. **[T]** MR Double check that nitrogen is flowing over the Mastcam-Zs or the window port.
9. **[O,T]** MR If not already done, mate the Right and Left Mastcam-Zs into the GSE. Follow the procedure in "MastcamZ_GSE_Manual".
10. **[T]** MR Verify that the thermocouples are turned on and properly reading out.
11. **[O,T]** MR Ensure that the camera unit and GSE wires are secure, kink-free, and do not present tripping hazards when the lights are turned off.
12. **[O,D]** MR Check the camera temperature and ensure nominal operation.
13. **[D]** N/A Record the following environmental information:

Date 5/6 Time 9:15 Initial MR

- Cleanroom temperature N/A pressure ______ humidity ______

14. [O,D, L] Notes:

____

____

____

15. [D,T] MR Take time-stamped pictures of this page, the integrating sphere, and the whole test/GSE set-up.
16. [T] MR Power on the integrating sphere. Follow the procedure in "Labsphere_Manual". Record the time the lamp is turned on 9:20.
17. [D,T] MR Record the exact readout value of the integrating sphere's radiance: 5.021 mW/cm2/sr.
18. [T,O, L] MR Confirm that the camera systems and GSEs are powered on and ready for use. Follow the procedure in "MastcamZ_GSE_Manual".
19. [D, L] Notes:

____

____

____

Table 2. The Nominal Radiance Values (calibrated integrating sphere output).

IS Output Radiances	Nominal Radiance [mW/cm2/sr]
Radiance 1	5.0
Radiance 2	10.0

Date 5/6 Time 16:05 Initial MR

Left and Right Mastcam-Z Tests

Do at end of L

Dark Current with the Right and Left Mastcam-Zs

20. [T] ____ ~~Cover the port window and turn off the lights.~~
21. [D] MR Record temperature information:
    - Left Mastcam-Z CCD temp 22.2
    - Right Mastcam-Z CCD temp 24.9
22. [D,T] ____ ~~Take digital pictures of the geometric target's position, and the whole test/GSE set-up.~~
23. [O] Load and execute camera script **441TEMPR03**, which captures 5 dark frames through filter 7 at the exposure times 0.0, 10.0, 20.0, and 100 seconds. The estimated duration is 12 minutes.
24. [O] Load and execute camera script **441TEMPL03**, which captures 5 dark frames through filter 7 at the exposure times 0.0, 10.0, 20.0, and 100 seconds. The estimated duration is 12 minutes.
25. [D] MR Record image names and parameters in Image Log.
26. [T] ____ ~~Uncover the port window.~~
27. [D, L] Notes:____________________

441 TEMPR00 → room dark next : command L0+R0 40s integration at 26mm autofocus off

" LC0 → go to Data Validation on pg 17

Date 5/6 Time 9:15 Initial MR

Center the Integrating Sphere on the Left Mastcam-Z

28. **[T]** MR With the assistance of MSSS hardware person, move the integrating sphere output as close to the Left Mastcam-Z as possible centered on the Mastcam-Z's boresight.
29. ~~**[T]** ____ Turn off lights.~~
30. **[O]** MR Capture a test image with filter 0 at 26mm to get the sphere centered. Save the image as **423TAMBL00**.
31. **[V,O,T]** MR Open images, and if the images show that the integrating sphere is not centered, center the integrating sphere disc in the frame. Recapture **423TAMBL00** frames if necessary.
32. **[D]** MR Record image names and parameters in the image Log.
33. ~~**[T]** MR Lights off~~
34. **[D, L]** Notes:

Caplinger positioned sphere ~9cm from L camera
Final test image is centered, filling FOV
except small portion of 4 corners

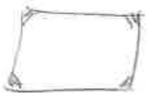

Radiance Value 1 for the Left Mastcam-Z

35. **[T]** MK Set the integrating sphere output to Radiance 1 found in Table 2.
36. **[D,T]** MK Record exact integrating sphere readout value 5.021 mW/cm2/sr.
37. **[D]** MR Record the following temperatures:
    - Left Camera CCD temp 23.2
    - Right Camera CCD temp ______
38. **[D,T]** MK Take time-stamped digital pictures of the setup and integrating sphere readout.
39. **[O]** MK Insert the note "ISOP=[radiance]" and execute camera script **423TAMBL02**, which captures 5 frames for 40% and 80% full-well and 5 bias frames with the 7 non-solar filters at three focal lengths. The estimated duration is about 40 minutes.
40. **[D,T]** MK Record exact integrating sphere readout value 5.024 mW/cm2/sr.

41. **[O]** MR Insert the note "ISOP=[radiance]" and execute camera script **423TAMBL05**, which captures 5 frames for 40% and 80% full-well and 5 bias frames for filters 0 and 1 at seven focal lengths. The estimated duration is 15 minutes.
42. **[D,T]** MR Record exact integrating sphere readout value 5.034 mW/cm2/sr.

43. **[O]** SKIP Insert the note "ISOP=[radiance]" and execute camera script **412TAMBL05**, which captures 10 frames for 9 exposure times with filter 0 at 100mm focal length. The estimated duration is 12 minutes. ~~05~~ 06 X SKIP (come back to this if time at end of R Lactivities)
44. **[D,T]** MR Record exact integrating sphere readout value 5.031 mW/cm2/sr.
45. **[D]** ____ Record image names and parameters in the Image Log.
46. **[D, L]** Notes: ______

insert continuous zoom here: ~~[illegible]~~ 423TAMBL07

MK

sphere readout 5.029

Date 5/6 Time 11:15 Initial MR

Radiance Value 2 for the Left Mastcam-Z

47. **[T]** MR Set the integrating sphere output to Radiance 2 found in Table 2.
48. **[D,T]** MR Record exact integrating sphere readout value 10.08 mW/cm2/sr.
49. **[D]** ____ Record the following temperatures:
    - Left Camera CCD temp ____________ Not recorded
    - Right Camera CCD temp ____________
50. **[D,T]** ____ Take time-stamped digital pictures of the setup and integrating sphere readout.

51. **[O]** MR Insert the note "ISOP=[radiance]" and execute camera script **423TAMBL02**, which captures 5 frames for 40% and 80% full-well and 5 bias frames with the 7 non-solar filters at three focal lengths. The estimated duration is about 40 minutes.
52. **[D,T]** MR Record exact integrating sphere readout value 10.065 mW/cm2/sr.

updating exp times

53. **[O]** MR Insert the note "ISOP=[radiance]" and execute camera script **423TAMBL05**, which captures 5 frames for 40% and 80% full-well and 5 bias frames for filters 0 and 1 at seven focal lengths. The estimated duration is 15 minutes.
54. **[D,T]** CO Record exact integrating sphere readout value 10.057 mW/cm2/sr.

55. **[O]** CO If time permits, insert the note "ISOP=[radiance]" and execute camera script **423TAMBL~~07~~** 08, which captures 80% full-well frames for filter 0 at 70 focal lengths. The estimated duration is ~~15~~ 20 minutes.
56. **[D,T]** CO Record exact integrating sphere readout value 10.055 mW/cm2/sr.

Skip - can do if there's time to kill before moving sphere to R

57. **[O]** CO Insert the note "ISOP=[radiance]" and execute camera script **412TAMBL08**, which captures 10 frames for 9 exposure times with filter 0 at 100mm focal length. The estimated duration is 12 minutes. X
58. **[D,T]** CO Record exact integrating sphere readout value " mW/cm2/sr.

Date 5/6 Time 12p Initial ct

59. **[D]** ct Record image names and parameters in the Image Log.

60. **[D, L]** Notes: ______

Insert here:
→ ~~continuous zoom test~~
→ ~~dark~~ room dark
→ ~~dark~~

1 cont zoom ✓
2 sphere off ✓
3 camera dark
4 room dark
5 move sphere to R

could do here for R room

Data Validation

61. **[T]** ct Lights on

62. **[V]** ct Upload data to server.

63. **[V]** ct Run the "Radiometric_Calibration_42_Validation" Jupyter notebook on the acquired data for the Right and Left Mastcam-Z with the window off. This analysis can take place while the test continues.
    - Create preliminary flat-field images and radiometric coefficients for each filter.
    - Save results in the calibration records.

64. **[V,D, L]** Notes: ______

Date 5/6 Time 1:00 Initial MK

Center the Integrating Sphere on the Right Mastcam-Z

65. **[T]** MK Do not change the integrating sphere output. ~~changed back to S,~~ will fix that first
66. **[T]** MK With the assistance of MSSS hardware person, move integrating sphere output as close to the Right Mastcam-Z as possible centered on the Mastcam-Z's boresight.
67. **[T]** MK ~~Turn off lights~~.
68. **[O]** MK Capture a test image with filter 0 at 26mm to get the sphere centered. Save the image as **423TAMBR00**.
69. **[V,O,T]** MK Open images, and if the images show that the integrating sphere is not centered, center the integrating sphere disc in the frame. Recapture **423TAMBR00** frames if necessary.
70. **[D]** MK Record image names and parameters in the image Log.
71. **[T]** MK Lights off
72. **[D, L]** Notes:

___

___

___

13:13
Date 5/6 Time ~~13~~ Initial MR

Radiance Value 2 for the Right Mastcam-Z ← doing ~~Value 1 this first~~

73. **[T]** MR Do not change the integrating sphere output.
74. **[D,T]** MR Record exact integrating sphere readout value 10.005 mW/cm2/sr.
75. **[D]** ____ Record the following temperatures:
    - Left Camera CCD temp 21.8
    - Right Camera CCD temp 24.6
76. **[D,T]** MR Take time-stamped digital pictures of the setup and integrating sphere readout.
77. **[O]** MR Insert the note "ISOP=[radiance]" and execute camera script **423TAMBR02**, which captures 5 frames for 40% and 80% full-well and 5 bias frames with the 7 non-solar filters at three focal lengths. The estimated duration is about 40 minutes.
78. **[D,T]** MR Record exact integrating sphere readout value 10.035 mW/cm2/sr.
79. **[O]** MR Insert the note "ISOP=[radiance]" and execute camera script **423TAMBR05**, which captures 5 frames for 40% and 80% full-well and 5 bias frames for filters 0 and 1 at seven focal lengths. The estimated duration is 15 minutes.
80. **[D,T]** MR Record exact integrating sphere readout value 10. 042 mW/cm2/sr.
81. **[O]** MR If time permits, insert the note "ISOP=[radiance]" and execute camera script **423TAMBR~~07~~** 08, which captures 80% full-well frames for filter 0 at 70 focal lengths. The estimated duration is 15 minutes.
82. **[D,T]** MR Record exact integrating sphere readout value 10.04 mW/cm2/sr.
83. **[D]** ____ Record image names and parameters in the Image Log.
84. **[D, L]** Notes: ____________________

Radiance Value 1 for the Right Mastcam-Z ← ~~doing the first~~

85. **[T]** MR Set the integrating sphere output to Radiance 1 found in Table 2.
86. **[D,T]** MR Record exact integrating sphere readout value ~~4.9988~~ 5.0038 mW/cm2/sr.
87. **[D]** MR Record the following temperatures:
    - Left Camera CCD temp ____________
    - Right Camera CCD temp 25.4
88. **[D,T]** X Take time-stamped digital pictures of the setup and integrating sphere readout.

89. **[O]** MR Insert the note "ISOP=[radiance]" and execute camera script **423TAMBR02**, which captures 5 frames for 40% and 80% full-well and 5 bias frames with the 7 non-solar filters at three focal lengths. The estimated duration is about 40 minutes.
90. **[D,T]** MR Record exact integrating sphere readout value 5.0312 mW/cm2/sr.

5.0212 at start of ↓

91. **[O]** MR Insert the note "ISOP=[radiance]" and execute camera script **423TAMBR05**, which captures 5 frames for 40% and 80% full-well and 5 bias frames for filters 0 and 1 at seven focal lengths. The estimated duration is 15 minutes.
92. **[D,T]** MR Record exact integrating sphere readout value 5.0387 mW/cm2/sr.

93. **[D]** ~~CO~~ Record image names and parameters in the Image Log.
94. **[D, L]** Notes: ____________

MR 423TAMBR07

sphere readout 5.0262 mW/cm2/sr

→ go back to L and R camera darks after this

Date 5/6 Time 4:25 Initial MR

Data Validation

95. **[T]** MR Lights on
96. **[V]** MR Upload data to server.
97. **[V]** MR Run the "Radiometric_Calibration_42_Validation" Jupyter notebook on the acquired data for the Right and Left Mastcam-Z with the window off. This analysis can take place while the test continues.
    - Create preliminary flat-field images and radiometric coefficients for each filter.
    - Save results in the calibration records.
98. **[V,D, L]** Notes: ____

____

____

Dark Current with the Right and Left Mastcam-Zs

Skip

99. **[T]** ____ Cover the port window and turn off the lights.
100. **[D]** ____ Record temperature information:
    - Left Mastcam-Z CCD temp ____________
    - Right Mastcam-Z CCD temp ____________
101. **[D,T]** ____ Take digital pictures of the geometric target's position, and the whole test/GSE set-up.
102. **[O]** Load and execute camera script **441TEMPR03**, which captures 5 dark frames through filter 7 at the exposure times 0.0, 10.0, 20.0, and 100 seconds. The estimated duration is 12 minutes.
103. **[O]** Load and execute camera script **441TEMPL03**, which captures 5 dark frames through filter 7 at the exposure times 0.0, 10.0, 20.0, and 100 seconds. The estimated duration is 12 minutes.
104. **[D]** ____ Record image names and parameters in Image Log.
105. **[T]** ____ Uncover the port window.
106. **[D, L]** Notes:____

____

____

Shutdown Procedure

107. [D,T] [illegible] Take digital pictures of this page and the test setup.
108. [D,O] [illegible] Review entries in Image Log, GSE command log, and image headers.
109. [D, L] [illegible] Review calibration procedure and ensure that each task is initialed.
110. [D, L] Notes: ____________________

111. [V, L] [illegible] Before making the decision to break down the test setup, ensure that adequate data were acquired for the test requirements. See "MastcamZCalPlan" for these requirements.
112. [V] Notes: ____________________

Data Validator (signature) [illegible]

Date 5/6/19 Time 4:30 pm

113. [V, L] [illegible] Give the go/no-go decision. Have enough data been acquired to fulfill test requirements? See "MastcamZCalPlan" for these requirements.
114. [D, L] [illegible] Update the Log Document.
115. [L] Notes: ____________________

Calibration Lead (signature) [illegible]

Date 5/6/19 Time 4:30

Date 5/6 Time 5p Initial CT

116. [O, L] CT Ensure that the camera and GSE are in a safe state.
117. [O,D] CT Review the Image Log with the documentarian. Exchange high-fives.
118. [O] Notes: ______

Camera Operator (signature) Elsa Jensen

Date 5/6/19 Time 5pm

119. [T] CT If the next test does not require the integrating sphere, position it away from the chamber or bench. Otherwise, be sure not to move it. The next test is _______.
120. [T] CT Ensure that all other test equipment is safely put away.
121. [T] Notes: ______

Technician (signature) Christina Dat

Date 5/6/19 Time 5:00pm

122. [D, L] CT Double-check this procedure and ensure that the top of each page is initialed with the time and date.
123. [D] CT Photo-scan this document, save it on the cloud, and file the hardcopy in the Log Binder. Upload the digital pictures taken during this test in the appropriate archive on the cloud. The required links are on the Wiki.
124. [D] CT ~~Double~~-check that every required cell the Image Log is accurately filled. When this is complete, print the Image Log and file it the Log Binder after this document.
125. [D] Notes: ______

Documentarian (signature) Christina D

Date 5/6/19 Time 5:00pm
