# Supplementary material for: Pre-Flight Calibration of the Mars 2020 Rover Mastcam Zoom (Mastcam-Z) Multispectral, Stereoscopic Imager
Source: Space Sci Rev. 2021 Feb 18;217(2):29. doi: 10.1007/s11214-021-00795-x (PMC7892537; doi:10.1007/s11214-021-00795-x)
Supplement: Supplementary file 1 — (ZIP 98.6 MB) [file 11214_2021_795_MOESM1_ESM.zip › CalPro_425_Radiometric_v2_03.pdf]

Date 4/27 Time 2:00p Initials [illegible]

Radiometric Calibration Procedure for Mastcam-Z TVAC Testing at MSSS (Pro. 4.2.5)

[Procedure version 2.03, prepared by the Mastcam-Z calibration team at Cornell University]

These measurements are performed on the camera and at the temperature designated below as specified in the Mastcam-Z Calibration Plan,

Unit Under Test:

Left FM __X__ **Right FM** __X__ **EQM**____ **Other**________

These measurements are performed at temperature:

-35° C______ **-10°C** __X__ **+5°C**______ **Ambient**_____ **Other**________

These measurements are performed at,

MSSS __X__ **ASU**______ **Other**______

Date 4/27/2019 **Start Time** 2:00pm **End Time** 8:00pm

Estimated Duration __4.5 hours__

Scheduled Start Time 11:00 AM **Sch. End Time** 8:00pm

Calibration Lead [L] JIM BELL **Documentarian [D]** PAUL Corlies

Camera Operator [O] ELSA, TEX Kubacki **Technician [T]** Christian, ANDY Winhold

Data Validator [V] PAUL Corlies **Other** ________

Date 4/27 Time 2p Initials [illegible]

Change Log

Version	Name	Change
v1_01<br>17 Sep 2018	C. Tate	(first draft)
v1_20<br>1 Nov 2018	C. Tate	Procedure edits prior to EQM testing
v1_23<br>10 Dec. 2018	C. Tate	Procedure edits after EQM testing
v2_03<br>27 April 2019	C. Tate	Approved version prior to FM testing

Document Approval

x [signature] 5/6/19

Approved by James Bell — Date
Mastcam-Z PI
Arizona State University

x [signature] 5/6/19

Approved by Alexander Hayes — Date
Mastcam-Z Calibration Working Group
Lead, Cornell University

Approved by Justin Maki — Date
Mastcam-Z Deputy PI and Investigation
Scientist, Jet Propulsion Laboratory

[signature] 4-29-19

Approved by Christian Tate — Date
Procedure Author
Cornell University

Approved by — Date

Table of Contents

RADIOMETRIC CALIBRATION PROCEDURE FOR MASTCAM-Z TVAC TESTING AT MSSS (PRO. 4.2.5) 1

- CHANGE LOG 2
- DOCUMENT APPROVAL 2
- TEST DESCRIPTION 4
- SOFTWARE PREPARATION 4
  - *Table 1. File naming convention for the camera script prefixes and frame filenames: "AAABBBBCDD" 4*
- HARDWARE INSTALLATION 6
  - *Figure 1. ASU Floor Plan for Geometric Testing in the TVAC Chamber. The MSSS Floor Plan allows for similar target and source placements relative to the chamber window. 6*
  - *Table 2. The Nominal Radiance Values (calibrated integrating sphere output). 8*

RIGHT AND LEFT MASTCAM-Z TESTS 9

- CENTER THE INTEGRATING SPHERE 9
- RADIANCE VALUE 1 FOR THE RIGHT MASTCAM-ZS 10
- RADIANCE VALUE 1 FOR THE LEFT MASTCAM-ZS 11
- RADIANCE VALUE 2 FOR THE LEFT MASTCAM-Z 12
- RADIANCE VALUE 2 FOR THE RIGHT MASTCAM-Z 13
- DATA VALIDATION 14

SHUTDOWN PROCEDURE 15

Test Description

Excerpt from the Calibration Plan 4.2,

> The objectives of these tests are to derive flat field images as well as the coefficients to allow a conversion from reduced (bias, dark, and flat field corrected) DN/s to absolute radiometric response (W/cm^2/sr per filter) for (a) the R, G, and B microfilters of the Bayer Pattern Filter detectors in each camera head (clear filter), (b) the 14 non-solar Mastcam-Z spectral filters "Science Filters", and, if time permits, (c) the two Mastcam-Z neutral density solar filters; and to provide an estimate of the uncertainty in these coefficients and, at Priority 2, their temperature dependence. This test builds off the Section 4.3 – Spectral Throughput Calibration to accurately account for the filter spectral response in the conversion. The requirement of knowing the relative response on the shape of the spectral throughput to ±5% combined with the absolute Radiance accuracy of the integration sphere at ±5% still allows the ±10% absolute radiometric calibration requirement to be met.

Software Preparation

The software and files required for this test are prepared well in advance of test day. This checklist ensures that the following are present, debugged, and executable: (1) all fast-look scripts, (2) automated header generation of all relevant camera parameters, target positioning, and metadata, (3) all camera scripts that command the camera unit, and (4) the directories/file-paths pointing to the data repositories of this specific test.

Table 1. File naming convention for the camera script prefixes and frame filenames: "AAABBBBCDD"

Code	Name	Example	Value
"AAA"	Calibration Plan Section	"411" = Cal. Plan 4.1.1 chapter 4, section 1, subsection 1	423, 412
"BBBB"	Location of test or ASU Chamber temperature	"MSSS" = test at MSSS, "TN10" = ASU TVAC -10C, …	TAMB
"C"	Camera unit under test	"L" = Left Mastcam-Z, "R" = Right Mastcam-Z, "E" =EQM, "C" =COTS	R/L
"DD"	Part of test (radiance value)	"00" = test set up, "01" = first radiance value …	00-08

1. **[D]** ✓ Look up the daily calibration schedule and record the scheduled start and end time of this test on the cover page of this document. Also fill out and double-check the other information on the cover page.
2. **[D]** ✓ Ensure that all supplemental manuals are on hand. These are,
   - Labsphere_Manual,
   - Validator_Manual, Documentarian_Manual
   - MastcamZCalPlan
3. **[D]** ✓ Ensure that the Image Log is present and ready to use. Find and open the Google Sheets file "Image_Log_42" (47). There is a link on the Wiki.
4. **[V]** ex Check that all Calgorithms fast-look and validation scripts are present, up-to-date, and ready to analyze test output. Find and open the "Radiometric_Calibration_42_Validation" (47) Jupyter notebook. There is a link on the Wiki.
5. **[O]** ✓ Check that all camera scripts required for this test are present, up-to-date and ready to command the ground support equipment (GSE). These are,
   - **413TN10R00 - 413TN10R08** and **425TN10R00 - 425TN10R06**
   - **413TN10L00 - 413TN10L08** and **425TN10L00 - 425TN10L06**
6. **[O,V,D, L]** Notes:

____

____

____

Hardware Installation

This procedure is for the ambient TVAC chamber testing at MSSS. Figure 1 shows the nominal layout of the TVAC chamber, workspace, Mastcam-Zs, ground support equipment (GSE), targets, sources, and other equipment necessary for this test if it happens at ASU. Although MSSS' cleanroom is different than ASU's, the placement of the targets and sources relative to the chamber window is similar.

Figure 1. ASU Floor Plan for Geometric Testing in the TVAC Chamber. The MSSS Floor Plan allows for similar target and source placements relative to the chamber window.

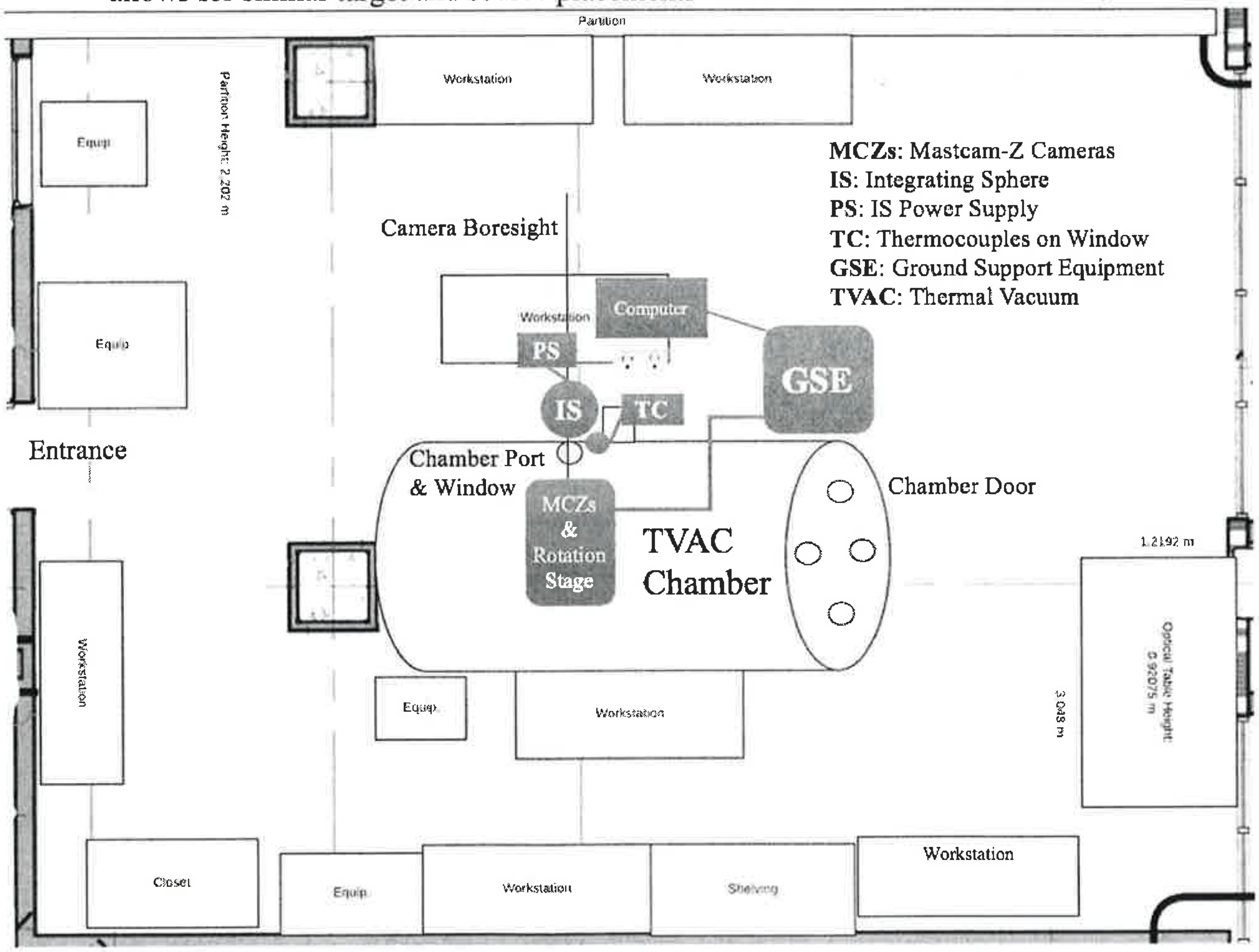

7. [T,O, L] ✓ Ensure that all personnel in the cleanroom are following the cleanroom practices for electrostatic discharge, proper clothing and other safety concerns.
8. [T] ✓ Double check that nitrogen is flowing over the Mastcam-Zs or the window port.

Date 4/27/19 Time 2:15p Initial JFB

9. **[O,T]** ✓ If not already done, mate the Right and Left Mastcam-Zs into the GSE. Follow the procedure in "MastcamZ_GSE_Manual".
10. **[T]** ✓ Verify that the thermocouples are turned on and properly reading out.
11. **[O,T]** ✓ Ensure that the camera unit and GSE wires are secure, kink-free, and do not present tripping hazards when the lights are turned off.
12. **[O,D]** ✓ Check the camera temperature and ensure nominal operation. -8.4°C
13. **[D]** ✓ Record the following environmental information: 2:20 pm
    - Cleanroom temperature 25°C pressure amb. humidity 38%
14. **[O,D, L]** Notes:

____

____

____

15. **[D,T]** [illegible] Take time-stamped pictures of this page, the integrating sphere, and the whole test/GSE set-up.
16. **[T]** ✓ Power on the integrating sphere. Follow the procedure in "Labsphere_Manual". Record the time the lamp is turned on 2:00 pm.
17. **[D,T]** ✓ Record the exact readout value of the integrating sphere's radiance: 5.52 mW/cm2/sr.
18. **[T,O, L]** ✓ Confirm that the camera systems and GSEs are powered on and ready for use. Follow the procedure in "MastcamZ_GSE_Manual".
19. **[D, L]** Notes:

Port is 3 mm from the window

____

____

NOTE: Make sure electronics is properly rotated on sphere so tech can see the readout panel

Date 4/27 Time 2:20 Initial JF

Table 2. The Nominal Radiance Values (calibrated integrating sphere output).

IS Output Radiances	Nominal Radiance [mW/cm2/sr]	
Radiance 1	5.0	5.2
Radiance 2	10.0	10.2

Date 7/27 Time 2:25 Initial JFB

Right and Left Mastcam-Z Tests

Center the Integrating Sphere

20. **[T]** ✓ Move integrating sphere output as close to the chamber window as possible centered on the Right Mastcam-Z boresight.
21. **[O]** ✓ Insert the note "ISOP=[radiance]" (5.52) and execute camera script **425TN10R00**. This script captures one auto-exposure at 40% full-well and one bias frame for filter 0 at 26 mm focal length.
22. **[V,O,T]** ✓ Open images, and if the images show that the integrating sphere is not centered, center the integrating sphere disc in the frame. Recapture **425TN10R00** frames if necessary.
23. **[D]** ✓ Record image names and ~~parameters in the image Log.~~
24. **[O,D,V]** ✓ Approximate the full-well percentage of the center pixels of the image and verify that they are about 40% (or DN 155).
    → Red, Green, or Blue?
25. **[T]** ✓ Lights off
26. **[D, L]** Notes:

MODS:

1) Choose sub-frame for auto-exposure
   672 624 304 224 = sub-frame parameters
   ⇒ change scripts later!

Radiance Value 1 for the Right Mastcam-Zs

27. **[T]** ✓ Set integrating sphere output to the radiance value 1 defined in Table 2.
28. **[D,T]** ✓ Record exact integrating sphere readout value 5.288 mW/cm2/sr.
29. **[D]** ok Record temperature information:
    - Chamber temp N/A Port temp N/A
    - Camera CCD temp -3.3 °C Optics temp N/A
30. **[D,T]** ✓ Take time-stamped digital pictures of the setup and integrating sphere readout. ✓
31. **[O]** ✓ Insert the note "ISOP=[radiance]" and execute camera script **425TN10R02**, which captures 5 frames for 40% and 80% full-well and 10 bias frames with the 7 non-solar filters at three focal lengths. The estimated duration is 35 minutes.
32. **[D,T]** ✓ Record exact integrating sphere readout value ~~5.290~~ 5.321 mW/cm2/sr.
33. **[redacted]** ✓ Insert the note "ISOP=[radiance]" and execute camera script **413TN10R05**, which captures 10 frames for 9 exposure times with filter 0 at 100mm focal length. The estimated duration is 9 minutes.
34. **[D,T]** ✓ Record exact integrating sphere readout value 5.283 mW/cm2/sr.
35. **[D]** ✓ Record image names and parameters in the Image Log.
36. **[D, L]** Notes: Run step #33 first, started at 3:27 pm
    Run step #31 second, start at 3:49 pm
    completed final script at 4:30pm, camera at -2.1°C

Kludge: Set auto-exposure threshold to 1%, then
Target DN = ~~110~~ 102 hits ≈ 40% full well mark
DN = 155 " ≈ 80% " ⟸ Too high?

Run photon transfer test first, use to tweak the 40% and 80% flux levels...

NOTE
sphere 5.333 at end of last script.
moving sphere back away from window, sphere now reads 5.193
moving sphere close again: 5.318

2.4% change in radiance due to window reflection

Date 4/27 Time 4:35 Initial JFB

Radiance Value 1 for the Left Mastcam-Zs

37. **[T]** ✓ Center the integrating sphere for the Left Mastcam-Z. Do not change the radiance value.
38. **[O]** ✓ Insert the note "ISOP=[radiance]" and execute camera script **425TN10L00**. This script captures one auto-exposure at 40% full-well and one bias frame for filter 0 at 26 mm focal length.
39. **[V,O,T]** ✓ Open images, and if the images show that the integrating sphere is not centered, center the integrating sphere disc in the frame. Recapture **425TN10L00** frames if necessary.
40. **[D]** ✓ Record image names and parameters in the image Log.
41. **[D,T]** ✓ Record exact integrating sphere readout value 5.318 mW/cm2/sr.
42. **[D]** ✓ Record temperature information:
    - Chamber temp N/A Port temp N/A
    - Camera CCD temp -5.3°C Optics temp N/A
43. **[D,T]** ✓ Take time-stamped digital pictures of the setup and integrating sphere readout.
44. **[[redacted]]** ✓ Insert the note "ISOP=[radiance]" and execute camera script **425TN10L02**, which captures 5 frames for 40% and 80% full-well and 10 bias frames with the 7 non-solar filters at three focal lengths. The estimated duration is ~~35 minutes~~.
45. **[D,T]** ✓ Record exact integrating sphere readout value 5.321 mW/cm2/sr.
46. **[O]** ✓ Insert the note "ISOP=[radiance]" and execute camera script **413TN10L03**, which captures 10 frames for 9 exposure times with filter 0 at 100mm focal length. The estimated duration is 9 minutes.
47. **[D,T]** ct Record exact integrating sphere readout value 5.318 mW/cm2/sr.
48. **[D]** ct Record image names and parameters in the Image Log.
49. **[D, L]** Notes: STARTED STEP #46 AT 4:40pm ended AT 4:50, camera at -4.8°C
STARTED STEP #44 AT 4:54pm ended AT 5:34, cam at -4.6°C
sphere @ 5.316

Radiance Value 2 for the Left Mastcam-Z

50. **[T]** ✓ Set integrating sphere output to the radiance value 2 defined in Table 2.
51. **[D,T]** ✓ Record exact integrating sphere readout value 10.029 mW/cm2/sr.
52. **[D]** ✓ Record temperature information:
    - Chamber temp N/A Port temp N/A
    - Camera CCD temp -5.1°C Optics temp N/A
53. **[D,T]** ✓ Take time-stamped digital pictures of the setup and integrating sphere readout.
54. **[O]** ✓ Insert the note "ISOP=[radiance]" and execute camera script **425TN10L02**, which captures 5 frames for 40% and 80% full-well and 10 bias frames with the 7 non-solar filters at three focal lengths. The estimated duration is 35 minutes.
55. **[D,T]** ✓ Record exact integrating sphere readout value 10.29 mW/cm2/sr.
56. **[O]** ✓ Insert the note "ISOP=[radiance]" and execute camera script **413TN10L08**, which captures 10 frames for 9 exposure times with filter 0 at 100mm focal length. The estimated duration is 9 minutes.
57. **[O]** ✓ If time permits, insert the note "ISOP=[radiance]" and execute camera script **425TN10L05**, which captures 5 frames for 40% and 80% full-well and 5 bias frames with filters 0 and 1 at seven focal lengths. These images are ~~compounded~~ companded at 8-bits. The estimated duration is 12 minutes.
58. **[D,T]** ✓ Record exact integrating sphere readout value 10.284 mW/cm2/sr.
59. **[D]** ✓ Record image names and parameters in the Image Log.
60. **[D, L]** Notes:

Started #56 at 5:40 pm, CCD @ -5.1°C, done at 5:50
Started #54 at 5:52 pm, CCD @ -5.4°C, done at 6:32
Started #57 at 6:35 pm, CCD @ -5.7°C, done at 6:46

Left camera homed and powered off at 6:50pm

Radiance Value 2 for the Right Mastcam-Z

61. **[T]** ✓ Center the integrating sphere for the Right Mastcam-Z. Do not change the radiance value.
62. **[[redacted]]** ✓ Insert the note "ISOP=[radiance]" and execute camera script **425TN10R00**. This script captures one auto-exposure at 40% full-well and one bias frame for filter 0 at 26 mm focal length.
63. **[V,O,T]** ✓ Open images, and if the images show that the integrating sphere is not centered, center the integrating sphere disc in the frame. Recapture **425TN10R00** frames if necessary.
64. **[D]** ✓ Record image names and parameters in the image Log.
65. **[D,T]** ✓ Record exact integrating sphere readout value 10.30 mW/cm2/sr.
66. **[D]** ✓ Record temperature information:
    - Chamber temp N/A Port temp N/A
    - Camera CCD temp -5.5°C Optics temp N/A
67. **[D,T]** ✓ Take time-stamped digital pictures of the setup and integrating sphere readout.
68. **[O]** ✓ Insert the note "ISOP=[radiance]" and execute camera script **425TN10R02**, which captures 5 frames for 40% and 80% full-well and 10 bias frames with the 7 non-solar filters at three focal lengths. The estimated duration is 35 minutes.
69. **[D,T]** ✓ Record exact integrating sphere readout value 10.290 mW/cm2/sr.
70. **[[redacted]]** CX Insert the note "ISOP=[radiance]" and execute camera script **413TN10R08**, which captures 10 frames for 9 exposure times with filter 0 at 100mm focal length. The estimated duration is 9 minutes.
71. **[[redacted]]** ✓ If time permits, insert the note "ISOP=[radiance]" and execute camera script **425TN10R05**, which captures 5 frames for 40% and 80% full-well and 5 bias frames with filters 0 and 1 at seven focal lengths. These images are compounded at 8-bits. The estimated duration is 12 minutes.
72. **[D,T]** ✓ Record exact integrating sphere readout value 10.30 mW/cm2/sr.
73. **[D]** ✓ Record image names and parameters in the Image Log.

Sphere was at 9.99 when pulled away from window.

Date 4/27 Time 658 Initial [illegible]

74. **[D, L]** Notes: Started #70 at 6:54pm, CCD @ -5.5°C. Done: 7:02
#68 at 7:05 pm, CCD@ -4.4°C. Done: 7:45pm
#71 at 7:50 pm, CCD@ -3.4°C. Done: 8:00 pm

Data Validation

75. **[T]** ✓ Lights on
76. **[V]** ✓ Upload data to server.
77. **[V]** ✓ Run the "Radiometric_Calibration_42_Validation" Jupyter notebook on the acquired data for the Right Mastcam-Z with the window off. This analysis can take place while the test continues.
    - Create preliminary flat-field images and radiometric coefficients for each filter.
    - Save results in the calibration records.
78. **[V,D, L]** Notes: ____________________

Date 4/27 Time ______ Initial JHB

Shutdown Procedure

79. **[D,T]** ✓ Take digital pictures of this page and the test setup.
80. **[D,O]** ✓ Review entries in Image Log, GSE command log, and image headers.
81. **[D, L]** ✓ Review calibration procedure and ensure that each task is initialed.
82. **[D, L]** Notes: ____________________

83. **[V, L]** ✓ Before making the decision to break down the test setup, ensure that adequate data were acquired for the test requirements. See "MastcamZCalPlan" for these requirements.
84. **[V]** Notes: ____________________

Data Validator (signature) [signature]

Date 4-27-19 Time 8:02 PM

85. **[V, L]** ✓ Give the go/no-go decision. Have enough data been acquired to fulfill test requirements? See "MastcamZCalPlan" for these requirements.
86. **[D, L]** ✓ Update the Log Document.
87. **[L]** Notes: ____________________

Calibration Lead (signature) [signature]

Date 4/27/19 Time 8:00pm

88. [[redacted], L] ✓ Ensure that the camera and GSE are in a safe state.
89. [[redacted],D] ✓ Review the Image Log with the documentarian. Exchange high-fives.
90. [[redacted]] Notes: ______

Camera Operator (signature) Elsa Jensen

Date 4/27/19 Time 8:05 pm

91. [T] ✓ If the next test does not require the integrating sphere, position it away from the chamber or bench. Otherwise, be sure not to move it. The next test is VR Target.
92. [T] ✓ Ensure that all other test equipment is safely put away.
93. [T] Notes: ______

Technician (signature) Christina [illegible]

Date 4-27 Time 8:08

94. [D, L] ✓ Double-check this procedure and ensure that the top of each page is initialed with the time and date.
95. [D] CX Photo-scan this document, save it on the cloud, and file the hardcopy in the Log Binder. Upload the digital pictures taken during this test in the appropriate archive on the cloud. The required links are on the Wiki.
96. [D] ✓ Double-check that every required cell the Image Log is accurately filled. When this is complete, print the Image Log and file it the Log Binder after this document.
97. [D] Notes: ______

Documentarian (signature) [illegible]

Date 4/27/2019 Time 8:03 PM
