# Supplementary material for: Pre-Flight Calibration of the Mars 2020 Rover Mastcam Zoom (Mastcam-Z) Multispectral, Stereoscopic Imager
Source: Space Sci Rev. 2021 Feb 18;217(2):29. doi: 10.1007/s11214-021-00795-x (PMC7892537; doi:10.1007/s11214-021-00795-x)
Supplement: Supplementary file 1 — (ZIP 98.6 MB) [file 11214_2021_795_MOESM1_ESM.zip › CalPro_426_Radiometric_v2_03.pdf]

Solar Radiometric Calibration Procedure for Mastcam-Z TVAC Testing at MSSS (Pro. 4.2.6)

[Procedure version 2.03, prepared by the Mastcam-Z calibration team at Cornell University]

These measurements are performed on the camera and at the temperature designated below as specified in the Mastcam-Z Calibration Plan,

Unit Under Test:

Left FM X **Right FM** X **EQM**_____ **Other**_____

These measurements are performed at temperature:

-35° C_____ **-10°C** X **+5°C**_____ **Ambient**_____ **Other**_____

These measurements are performed at,

MSSS X **ASU**_____ **Other**_____

Date 4/28/2019 **Start Time** 6:15 pm **End Time** 8:45 pm

Estimated Duration 1.0 hour

Scheduled Start Time — **Sch. End Time** —

Calibration Lead [L] Jim Bell **Documentarian [D]** Megan Barington

Camera Operator [[redacted]**]** Tex/Kathryn **Technician [T]** Christian, Andy Winhold

Data Validator [V] Paul Corlies **Other**_____

Change Log

Version	Name	Change
v1_01<br>17 Sep 2018	C. Tate	(first draft)
v1_20<br>1 Nov 2018	C. Tate	Procedure edits prior to EQM testing
v1_23<br>10 Dec. 2018	C. Tate	Procedure edits after EQM testing
v2_03<br>28 April 2019	C. Tate	Approved version prior to FM testing

Document Approval

x 5/6/19
Approved by James Bell Date
Mastcam-Z PI
Arizona State University

x 5/6/19
Approved by Alexander Hayes Date
Mastcam-Z Calibration Working Group Lead, Cornell University

Approved by Justin Maki Date
Mastcam-Z Deputy PI and Investigation Scientist, Jet Propulsion Laboratory

4/28/19
Approved by Christian Tate Date
Procedure Author
Cornell University

Approved by Date

Table of Contents

SOLAR RADIOMETRIC CALIBRATION PROCEDURE FOR MASTCAM-Z TVAC TESTING AT MSSS (PRO. 4.2.6)............ 1

Change Log........ 2
Document Approval 2
Test Description........ 4
Software Preparation 4
Table 1. File naming convention for the camera script prefixes and frame filenames: "AAABBBBCDD"............ 4
Hardware Installation 6
Figure 1. ASU Floor Plan for Geometric Testing in the TVAC Chamber. The MSSS Floor Plan allows for similar target and source placements relative to the chamber window. 6
Table 2. The Nominal Radiance Values (calibrated integrating sphere output). 8

RIGHT AND LEFT MASTCAM-Z TESTS........ 9

Center the Integrating Sphere........ 9
Radiance Value 1 for the Right and Left Mastcam-Zs 10
Radiance Value 2 for the Right and Left Mastcam-Zs 11
Data Validation........ 12

SHUTDOWN PROCEDURE 13

Test Description

Excerpt from the Calibration Plan 4.2,

> The objectives of these tests are to derive flat field images as well as the coefficients to allow a conversion from reduced (bias, dark, and flat field corrected) DN/s to absolute radiometric response (W/cm^2/sr per filter) for (a) the R, G, and B microfilters of the Bayer Pattern Filter detectors in each camera head (clear filter), (b) the 14 non-solar Mastcam-Z spectral filters "Science Filters", and, if time permits, (c) the two Mastcam-Z neutral density solar filters; and to provide an estimate of the uncertainty in these coefficients and, at Priority 2, their temperature dependence. This test builds off the Section 4.3 – Spectral Throughput Calibration to accurately account for the filter spectral response in the conversion. The requirement of knowing the relative response on the shape of the spectral throughput to ±5% combined with the absolute Radiance accuracy of the integration sphere at ±5% still allows the ±10% absolute radiometric calibration requirement to be met.

Software Preparation

The software and files required for this test are prepared well in advance of test day. This checklist ensures that the following are present, debugged, and executable: (1) all fast-look scripts, (2) automated header generation of all relevant camera parameters, target positioning, and metadata, (3) all camera scripts that command the camera unit, and (4) the directories/file-paths pointing to the data repositories of this specific test.

Table 1. File naming convention for the camera script prefixes and frame filenames: "AAABBBBCDD"

Code	Name	Example	Value
"AAA"	Calibration Plan Section	"411" = Cal. Plan 4.1.1 chapter 4, section 1, subsection 1	426
"BBBB"	Location of test or ASU Chamber temperature	"MSSS" = test at MSSS, "TN10" = ASU TVAC -10C, …	TN10
"C"	Camera unit under test	"L" = Left Mastcam-Z, "R" = Right Mastcam-Z, "E" =EQM, "C" =COTS	R/L
"DD"	Part of test (radiance value)	"00" = test set up, "01" = first radiance value …	00-02

1. **[D]** N/a Look up the daily calibration schedule and record the scheduled start and end time of this test on the cover page of this document. Also fill out and double-check the other information on the cover page.
2. **[D]** ✓ Ensure that all supplemental manuals are on hand. These are,
   - Labsphere_Manual,
   - Validator_Manual, Documentarian_Manual
   - MastcamZCalPlan
3. **[D]** ✓ Ensure that the Image Log is present and ready to use. Find and open the Google Sheets file "Image_Log_42". There is a link on the Wiki.
4. **[V]** ✓ Check that all Calgorithms fast-look and validation scripts are present, up-to-date, and ready to analyze test output. Find and open the "Radiometric_Calibration_42_Validation" Jupyter notebook. There is a link on the Wiki.
5. **[O]** ✓ Check that all camera scripts required for this test are present, up-to-date and ready to command the ground support equipment (GSE). These are,
   - **426TN10R00 - 426TN10R06**
   - **426TN10L00 - 426TN10L06**
6. **[[redacted],V,D, L]** Notes:

____

____

____

Hardware Installation

This procedure is for the ambient TVAC chamber testing at MSSS. Figure 1 shows the nominal layout of the TVAC chamber, workspace, Mastcam-Zs, ground support equipment (GSE), targets, sources, and other equipment necessary for this test if it happens at ASU. Although MSSS' cleanroom is different than ASU's, the placement of the targets and sources relative to the chamber window is similar.

Figure 1. ASU Floor Plan for Geometric Testing in the TVAC Chamber. The MSSS Floor Plan allows for similar target and source placements relative to the chamber window.

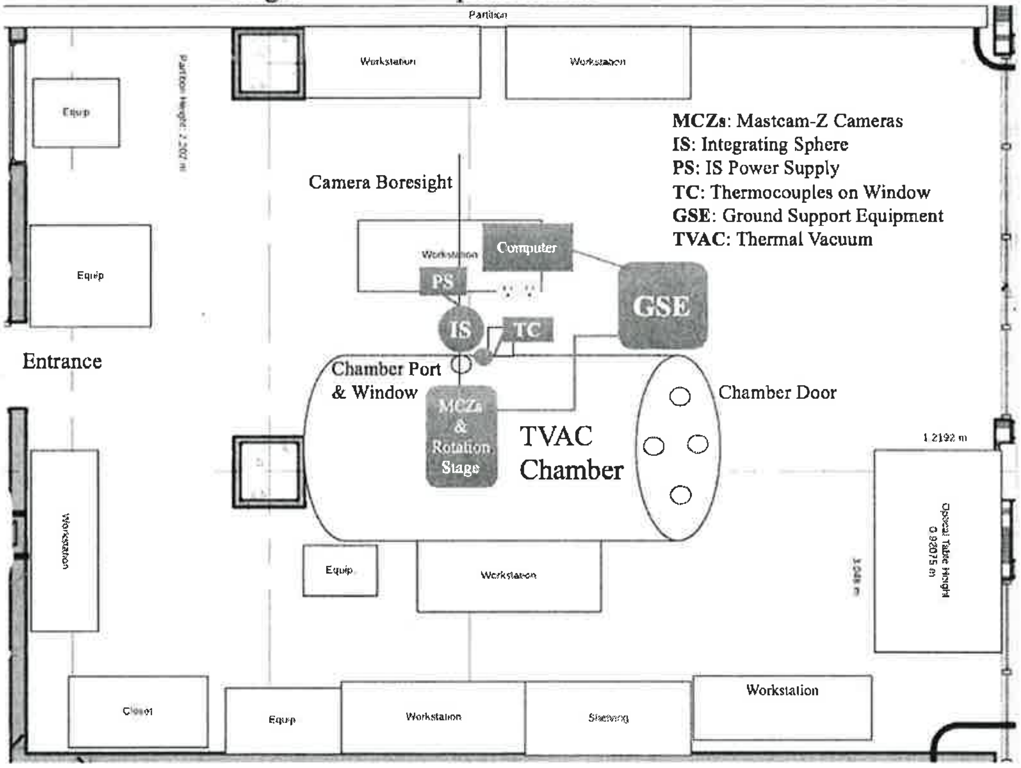

7. [T,O, L] ✓ Ensure that all personnel in the cleanroom are following the cleanroom practices for electrostatic discharge, proper clothing and other safety concerns.
8. [T] ✓ Double check that nitrogen is flowing over the Mastcam-Zs or the window port.
9. [[redacted],T] ✓ If not already done, mate the Right and Left Mastcam-Zs into the GSE. Follow the procedure in "MastcamZ_GSE_Manual".
10. [T] ✓ Verify that the thermocouples are turned on and properly reading out.

11. [O,T] ✓ Ensure that the camera unit and GSE wires are secure, kink-free, and do not present tripping hazards when the lights are turned off.
12. [O,D] ✓ Check the camera temperature and ensure nominal operation.
13. [D] n/a Record the following environmental information:
    - Cleanroom temperature N/A pressure N/A humidity N/A
14. [[redacted],D, L] Notes:

____

____

____

15. [D,T] [illegible] Take time-stamped pictures of this page, the integrating sphere, and the whole test/GSE set-up.
16. [T] ✓ Power on the integrating sphere. Follow the procedure in "Labsphere_Manual". Record the time the lamp is turned on 5:30 pm.
17. [D,T] [illegible] Record the exact readout value of the integrating sphere's radiance: 9.72 ~~[illegible]~~ mW/cm2/sr. INITIAL
18. [T,O, L] ✓ Confirm that the camera systems and GSEs are powered on and ready for use. Follow the procedure in "MastcamZ_GSE_Manual".
19. [D, L] Notes:

____

____

____

Table 2. The Nominal Radiance Values (calibrated integrating sphere output).

IS Output Radiances	Nominal Radiance [mW/cm2/sr]
Radiance 1	60.0
Radiance 2	80.0

Right and Left Mastcam-Z Tests

FILL FOV @ 100 MM

~0.3 M

Center the Integrating Sphere

20. **[T]** ✓ Move integrating sphere output as close to the chamber window as possible centered on the Right Mastcam-Z boresight.
21. **[O]** ✓ Capture test image at 100mm with both cameras with filter 0. Insert the note "ISOP=[radiance]" and call the images **426TN10R00** and **426TN10L00**.
22. **[V,O,T]** ✓ Open images, and if the images show that the integrating sphere is not centered, center the integrating sphere disc in both frames. Recapture **426TN10R00** and **426TN10L00** frames if necessary.
23. **[D]** ✓ Record image names and parameters in the image Log.
24. **[T]** N/A Lights off
25. **[D, L]** Notes:

focus at ∞

set sphere at 10 mW level (do not illuminate cameras with full lamp)

Distance to window 18.5 cm for first test image

" 12 cm 2nd

" 4.5 cm 3rd ...

12 cm Final

Testing 1, 10, ~~120~~ 60 sec. Levels: 400 DN or so... ok!

Date 4/28 Time 6:45 Initial JFB

Radiance Value 1 for the Right and Left Mastcam-Zs

26. **[T]** ✓ Set integrating sphere output to the radiance value 1 defined in Table 2.
27. **[D,T]** ✓ Record exact integrating sphere readout value 78.622 mW/cm2/sr.
28. **[D]** ~~CX~~ Record temperature information:
    - Chamber temp ~ -4°C Port temp N/A
    - Camera CCD temp (R) -3.6° ~~Optics~~ CCD temp (L) = -5.1°C
29. **[D,T]** ✓ Take time-stamped digital pictures of the setup and integrating sphere readout.
30. [[redacted]] ✓ Insert the note "ISOP=[radiance]" and execute camera script **426TN10R02**, which captures 5 frames at approximately 10% and 20% full-well and 5 bias frames with the solar filter at 100mm focal length and focus at infinity. The estimated duration is 18 minutes.
31. **[O]** ~~CX~~ Insert the note "ISOP=[radiance]" and execute camera script **426TN10L02**, which captures 5 frames at approximately 10% and 20% full-well and 5 bias frames with the solar filter at 100mm focal length and focus at infinity. The estimated duration is 8 minutes.
32. **[D,T]** ~~CX~~ Record exact integrating sphere readout value 79.25 mW/cm2/sr.
33. **[D]** ~~CX~~ Record image names and parameters in the Image Log.
34. **[D, L]** Notes: 76.383 sphere level for step #30 (end: ?)
    79.250 " for step #31

Right
→ 10 x 60 sec exposures in R8 ~400 DNs or so
~~[illegible]~~ 5 x 0 sec bias frames at end
then turn off one extra bulb (#1)
then 5 x 60 sec exposures at 50% flux level. (44.474)

Left
then 10x60 sec exposures in L8 (79.075 sphere) ~250 DNs or so
5x 0 sec bias frames (CCD @ -5.1°C)
Then 5x60 sec @ 50% Lamp = 47.041 sphere
47.066 last (78.651 @ end sphere)

Then 60 sec darks (lights off) x 3 + bias x 5
for both cameras
Temps: L = -5.3°C R = -3.7°C

End @ 7:55 pm

Radiance Value 2 for the Right and Left Mastcam-Zs

Skip

35. **[T]** ____ Set integrating sphere output to the radiance value 1 defined in Table 2.
36. **[D,T]** ____ Record exact integrating sphere readout value __________ mW/cm2/sr.
37. **[D]** ____ Record temperature information:
    - Chamber temp ________________ Port temp ______________
    - Camera CCD temp ______________ Optics temp ______________
38. **[D,T]** ____ Take time-stamped digital pictures of the setup and integrating sphere readout.
39. **[O]** ____ Insert the note "ISOP=[radiance]" and execute camera script **426TN10R02**, which captures 5 frames at approximately 10% and 20% full-well and 5 bias frames with the solar filter at 100mm focal length and focus at infinity. The estimated duration is 18 minutes.
40. **[O]** ____ Insert the note "ISOP=[radiance]" and execute camera script **426TN10L02**, which captures 5 frames at approximately 10% and 20% full-well and 5 bias frames with the solar filter at 100mm focal length and focus at infinity. The estimated duration is 8 minutes.
41. **[D,T]** ____ Record exact integrating sphere readout value __________ mW/cm2/sr.
42. **[D]** ____ Record image names and parameters in the Image Log.
43. **[D, L]** Notes: ____

____

____

Data Validation

44. **[T]** ✓ Lights on
45. **[V]** ✓ Upload data to server.
46. **[V]** N/A Run the "Radiometric_Calibration_42_Validation" Jupyter notebook on the acquired data for the Right Mastcam-Z with the window off. This analysis can take place while the test continues.
    - Create preliminary flat-field images and radiometric coefficients for each filter.
    - Save results in the calibration records.
47. **[V,D, L]** Notes: All good!

Date 4/29 Time 8:30 Initial [illegible]

Shutdown Procedure

48. **[D,T]** [illegible] Take digital pictures of this page and the test setup.
49. **[D,O]** [illegible] Review entries in Image Log, GSE command log, and image headers.
50. **[D, L]** [illegible] Review calibration procedure and ensure that each task is initialed.
51. **[D, L]** Notes: ______________________________________

52. **[V, L]** [illegible] Before making the decision to break down the test setup, ensure that adequate data were acquired for the test requirements. See "MastcamZCalPlan" for these requirements.
53. **[V]** Notes: All good

Data Validator (signature) [signature]

Date 4/28/19 Time 8:10

54. **[V, L]** ____ Give the go/no-go decision. Have enough data been acquired to fulfill test requirements? See "MastcamZCalPlan" for these requirements.
55. **[D, L]** ____ Update the Log Document.
56. **[L]** Notes: ______________________________________

Calibration Lead (signature) [signature]

Date 4/28/19 Time 8pm

57. [[redacted], L] CT Ensure that the camera and GSE are in a safe state.
58. [[redacted],D] CT Review the Image Log with the documentarian. Exchange high-fives.
59. [[redacted]] Notes: ____

Camera Operator (signature) x [signature] Tex

Date 5/7/19 Time 1pm

60. [T] CT If the next test does not require the integrating sphere, position it away from the chamber or bench. Otherwise, be sure not to move it. The next test is __________.
61. [T] CT Ensure that all other test equipment is safely put away.
62. [T] Notes: ____

Technician (signature) [signature]

Date April 29 2019 Time 8:38 am

63. [D, L] CT Double-check this procedure and ensure that the top of each page is initialed with the time and date.
64. [D] CT Photo-scan this document, save it on the cloud, and file the hardcopy in the Log Binder. Upload the digital pictures taken during this test in the appropriate archive on the cloud. The required links are on the Wiki.
65. [D] CT Double-check that every required cell the Image Log is accurately filled. When this is complete, print the Image Log and file it the Log Binder after this document.
66. [D] Notes: ____

Documentarian (signature) x [signature] Megan & Christian

Date 5/5/19 Time 3:00pm
