# Supplementary material for: Pre-Flight Calibration of the Mars 2020 Rover Mastcam Zoom (Mastcam-Z) Multispectral, Stereoscopic Imager
Source: Space Sci Rev. 2021 Feb 18;217(2):29. doi: 10.1007/s11214-021-00795-x (PMC7892537; doi:10.1007/s11214-021-00795-x)
Supplement: Supplementary file 1 — (ZIP 98.6 MB) [file 11214_2021_795_MOESM1_ESM.zip › CalPro_433_434_L_Spectral_Throughput_v2_02.pdf]

Left Spec.
~~Mastcam-Z JR Geometric Calibration (Pro. 4.6~~.5-7)

Date 5/4 Time 7a Initials [illegible]

Spectral Throughput Procedure for the Right Mastcam-Z for Ambient at MSSS

(Pro. 4.3.3-4)

[Procedure version 2.02, prepared by the Mastcam-Z calibration team at Cornell University]

These measurements are performed on the camera and at the temperature designated below as specified in the Mastcam-Z Calibration Plan,

Unit Under Test:

Left FM _____ **Right FM** __X__ **EQM**_____ **Other**_____

These measurements are performed at temperature:

-35°C_____ **- 10°C**_____ **+5°C**_____ **Ambient**__X__ **Other**_____

These measurements are performed at,

MSSS_____ **ASU**__X__ **Other**_____

Date 5/4/19 **Start Time** 7:30 **End Time** [illegible]

Estimated Duration 6.0 hours

Scheduled Start Time 7:30 **Sch. End Time** 13:30

Calibration Lead [L] Alex Hayes Melissa Rice **Documentarian [D]** Alexis Parkinson

Camera Operator [O] Tex Kubacki **Technician [T]** Paul Carlos Ernest Cisneros

Data Validator [V] Ole Jensen **Other** Jim Bell

Change Log

Version	Name	Change
v1_01<br>17 Sep 2018	C. Tate	(first draft)
v1_11<br>1 Nov 2018	C. Tate	Procedure edits prior to EQM testing
v1_13<br>1 Dec. 2018	C. Tate	Procedure edits after EQM testing
v2_02<br>5 May 2019	C. Tate	Approved version prior to FM testing

Document Approval

5/6/19

Approved by James Bell — Date
Mastcam-Z PI
Arizona State University

05/04/19

Approved by Alexander Hayes — Date
Mastcam-Z Calibration Working Group Lead, Cornell University

Approved by Justin Maki — Date
Mastcam-Z Deputy PI and Investigation Scientist, Jet Propulsion Laboratory

5/5/19

Approved by Ken Herkenhoff — Date
Mastcam-Z Co-Investigator
USGS

Approved by — Date

Approved by Christian Tate — Date
Procedure Author
Cornell University

Table of Contents

SPECTRAL THROUGHPUT PROCEDURE FOR THE RIGHT MASTCAM-Z FOR AMBIENT AT MSSS 1
CHANGE LOG 2
DOCUMENT APPROVAL 2
TEST DESCRIPTION 3
SOFTWARE PREPARATION 5
Table 1. File naming convention for the camera script prefixes and frame filenames: "AAABBBBCDD" 5
HARDWARE PREPARATION 7
Figure 1. MSSS Floor Plan for Geometric Testing in the Cleanroom. 7
MONOCHROMATOR WAVELENGTHS 10
*Table 2. Wavelengths for the Right Mastcam-Z filters **0-7** 10*
IN-BAND AND OUT-OF-BAND MEASUREMENTS FOR FILTER 0 OF THE RIGHT MASTCAM-Z 11
DATA VALIDATION 13
IN-BAND AND OUT-OF-BAND MEASUREMENTS FOR FILTER 1 OF THE RIGHT MASTCAM-Z 14
DATA VALIDATION 16
IN-BAND AND OUT-OF-BAND MEASUREMENTS FOR FILTER 2 OF THE RIGHT MASTCAM-Z 17
DATA VALIDATION 19
IN-BAND AND OUT-OF-BAND MEASUREMENTS FOR FILTER 3 OF THE RIGHT MASTCAM-Z 20
DATA VALIDATION 22
IN-BAND AND OUT-OF-BAND MEASUREMENTS FOR FILTER 4 OF THE RIGHT MASTCAM-Z 23
DATA VALIDATION 25
IN-BAND AND OUT-OF-BAND MEASUREMENTS FOR FILTER 5 OF THE RIGHT MASTCAM-Z 26
DATA VALIDATION 28
IN-BAND AND OUT-OF-BAND MEASUREMENTS FOR FILTER 6 OF THE RIGHT MASTCAM-Z 29
DATA VALIDATION 32
TIME CHECK 1 32
IN-BAND MEASUREMENTS FOR FILTER 7 OF THE RIGHT MASTCAM-Z 33
DATA VALIDATION 34
SHUTDOWN PROCEDURE 35

Test Description

Excerpt from the Calibration Plan 4.3,

> The objective of this test is to measure spectral transmission (spectral response functions) for each filter on each Mastcam-Z camera's filter wheel in a through-system sense—that

is, the spectral transmission through the optics, filter, and microlenses/Bayer Pattern Filters of the CCD detector.

Measurements from the vendor as well as experience with MSL has demonstrated little dependence with temperature; however, it is highly desirable to conduct these tests over a range of appropriate temperatures in a thermal vacuum chamber at Mars atmospheric pressure (or lower) to fully characterize the filters' thermal response.

Software Preparation

The software and files required for this test are prepared well in advance of test day. This checklist ensures that the following are present, debugged, and executable: (1) all fast-look scripts, (2) automated header generation of all relevant camera parameters, target positioning, and metadata, (3) all camera scripts that command the camera unit, and (4) the directories/file-paths pointing to the data repositories of this specific test.

Table 1. File naming convention for the camera script prefixes and frame filenames: "AAABBBBCDD"

Code	Name	Example	Value
"AAA"	Calibration Plan Section	"433" = Cal. Plan 4.3.3 chapter 4, section 3, subsection 3	433/434
"BBBB"	Location of test or ASU Chamber temperature	"MSSS" = test at MSSS, "TN10" = ASU TVAC -10C, …	TAMB
"C"	Camera unit under test	"L" = Left Mastcam-Z, "R" = Right Mastcam-Z, "E" =EQM, "C" =COTS	R
"DD"	Part of test (radiance value)	"00" = test set up, "01" = first radiance value …	00-07

1. **[D]** A64 Look up the daily calibration schedule and record the scheduled start and end time of this test on the cover page of this document. Also fill out and double-check the other information on the cover page.
2. **[D]** A64 Ensure that all supplemental manuals are on hand. These are,
   - Monochromator_Manual
   - Validator_Manual, Documentarian_Manual
   - MastcamZCalPlan
3. **[D]** A64 Ensure that the Image Log is present and ready to use. Find and open the Google Sheets file "Image_Log_43". There is a link on the Wiki.
4. **[V]** A64 Check that all Calgorithms fast-look and validation scripts are present, up-to-date, and ready to analyze test output. Find and open the "Spectral_Throughput_43_Validation" Jupyter notebook. There is a link on the Wiki.

5. **[O]** AGH Check that all camera scripts required for this test are present, up-to-date and ready to command the ground support equipment (GSE). These are,
    - **433TAMBR00 - 433TAMBR02**
    - **433TAMBR10 - 433TAMBR12**
    - **433TAMBR20 - 433TAMBR22**
    - **433TAMBR30 - 433TAMBR32**
    - **433TAMBR40 - 433TAMBR42**
    - **433TAMBR50 - 433TAMBR52**
    - **433TAMBR60 - 433TAMBR62**
    - **434TAMBR70 - 434TAMBR72**
6. **[O,V,D, L]** Notes:

____

____

____

Hardware Preparation

This procedure is for the ambient cleanroom testing at MSSS. Figure 1 shows the nominal layout of the cleanroom chamber, workspace, Mastcam-Zs, ground support equipment (GSE), targets, sources, and other equipment necessary.

Figure 1. MSSS Floor Plan for Geometric Testing in the Cleanroom.

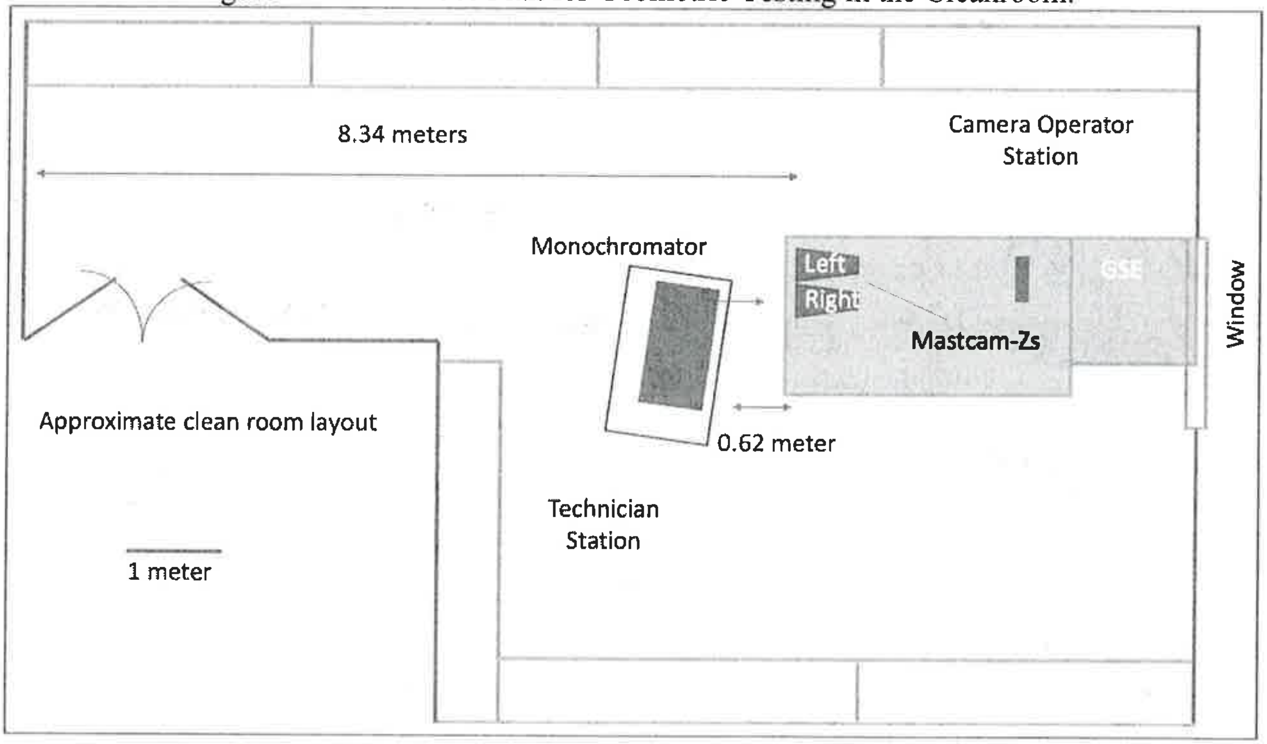

7. [T,O, L] A64 Ensure that all personnel in the cleanroom are following the cleanroom practices for electrostatic discharge and proper clothing.
8. [T] A64 Double check that the ionizers are flowing over the Mastcam-Zs.
9. [O,T] A64 If not already done, power on the Right Mastcam-Z and the GSE.
10. [O,T] A64 Ensure that the lights, monochromator, Mastcam-Zs and GSE wires are secure, kink-free, and do not present tripping hazards when the lights are turned off.
11. [O,D] A64 Check the camera temperature and ensure nominal operation. Record the following temperatures:
    - Left Mastcam-Z CCD temp 23.0
    - Right Mastcam-Z CCD temp 24.8

12. **[D]** AbH Record the following environmental information:
    - Cleanroom temperature 67.8°F pressure N/A humidity 54%
13. **[O,D, L]** Notes:

____

Ensure attenuator is not installed on radiometer

____

14. **[D,T]** AbH Take of the monochromator and the whole test/GSE set-up.
15. **[T]** AbH Power on the monochromator and radiometer, which takes about 40-120 minutes to warm up and stabilize. Follow the procedure in "Monochromator_Manual".
    - The monochromator was turned on at 8:00am.
16. **[T]** AbH Make sure that the monochromator's secondary filter-while in the clear position (i.e. that the ND2 filter **is not** in place.) ALSO CHECK ATTENUATOR ON RADIOMETER
17. **[T]** AbH Confirm that the entrance and exit slits are at 600 microns (i.e. that no one has changed them).
18. **[T]** AbH After the monochromator and radiometer have been on for at least ~~10~~ 60 minutes, perform a full radiometer scan between 300 and 1100 nm in 2 nm increments with a 2 second dwell-time at each wavelength. Follow the procedure in "Monochromator_Manual". Be sure to save file as **"mono_scan_g123_300nm_1100nm_2nm_" + "YYMMDD_hhmm"** where the last numbers encode the scan's date and start time. The estimated duration is 20 minutes.
    - Scan started at 12:16pm and completed at ______________.

19. **[T]** ____ After the monochromator and radiometer have been on for at least 60 minutes, perform a full radiometer scan between 300 and 1100 nm in 2 nm increments with a 2 second dwell-time at each wavelength. Follow the procedure in "Monochromator_Manual". Be sure to save file as **"mono_scan_g123_300nm_1100nm_2nm_" + "YYMMDD_hhmm"** where the last numbers encode the scan's date and start time. The estimated duration is 20 minutes.
    - Scan started at ______________ and completed at ______________.

20. **[D,T]** AGH Take of the monochromator and the whole test/GSE set-up.
21. **[T,D, L]** Notes:

____

____

____

Monochromator Wavelengths

Table 2. Wavelengths for the Right Mastcam-Z filters **0-7**

Filter	Peak Wavelength [nm]	Exposure In-band [ms]	Start In-band	Stop In-band	Step-size In-band	Minutes for In-band Scan	Exposure Out-band [ms]	Start Out-band	Stop Out-band	Step-size Out	Minutes for Out-band Scan
**R0**	625 ~~600~~ [illegible]	~~1.5~~ 0.4	400	710	2	19.2	300	302	1100 [illegible]	4	25.0
**R1**	~~800~~ 798	~~30~~ 0.5	780	826	2	3.9	1500	302	1100 [illegible]	6	23.6
**R2**	866	60	840	890	2	4.3	1500	302	1100 [illegible]	6	37.6
**R3**	908	75	884	940	2	4.7	1500	302	1100 [illegible]	6	23.6
**R4**	936	120	914	960	2	4.3	1500	302	1100	6	21.5
**R5**	975	300	952	1000	2	5.2	3000	302	1100	6	33.6
**R6**	1000	300	980	1100	2	11.2	3000	302	1100	6	33.6
**R7**	880	80000	868	892	4	48.4	160,000	340	1100	20	316

OUT-B. WAIT TIME = 5 + 10 X EXPOSURE

SECOND RUN:

 Date 5/5 Time 23:06 Initial [illegible]

In-band and Out-of-band Measurements for Filter 0 of the Right Mastcam-Z

Center slit in frame and set focus before lights off!

22. [T] AGH Lights off
23. [T] AGH Set the monochromator to the filter's peak wavelength given in Table 2.
24. [O,T,D] AGH Load and execute prefix **433TAMBR00**, which helps to find the optimal sub-frame, focus position, and exposure time. The GUI's note field should have "GRATING=G123,WAVELENGTH=[nanometers]".
25. [O,T] AGH Position the monochromator as need to aim the slit near the camera's boresight. Rerun script **433TAMBR00** as needed.
26. [L,O,D] AGH Record the following values,
    - sub-frame window: center x: 960 wx: 10 — sub-frame: 944 x ~~464~~ 416 32x208
    - focus position: 666
    - exposure time: 0.4 ~~[illegible]~~

ADDED: closed shutter and collected 10 frames w/ sub-frame and exposure for data collect.

27. [L,O] AGH Update the **433TAMBR~~00~~ 01/02** scripts for this sub-frame, focus position, and exposure time values.
28. [T] AGH Set up a monochromator scan for the filter's wait-time and **in-band** start, stop and step-size wavelengths given in Table 2.
29. [O,T,L] AGH Confirm that the script and monochromator scan have the same start, stop and step-size wavelengths. Set the dwell-time to at least 5 seconds between image sets (or to another appropriate value).
30. [O,T,D] [illegible] ~~Load and execute~~ prefix **433TAMBR01**, which captures 10 frames and 3 bias frames at the 100 mm focal length for each wavelength in the scan. Push continue in the GUI whenever the monochromator steps to the next wavelength position. The last wavelength position is for dark frames. The GUI's note field should have "GRATING=G123,WAVELENGTH=[nanometers]". The estimated duration is given in Table 2. WINDOW TOO HIGH?
31. [T] AGH Close the lamp shutter. The last block of images in the script is for dark frames. ✓
32. [D] AGH Double check that all image names, suffixes, and other parameters are recorded in Image log. ✓ ~~[illegible]~~ 57

✓33. **[O,V]** A6H Make sure that the filter **0**'s transmission was measured to no more that 1% on both sides of its transition curve. Notes: GOOD,

34. **[T]** A6H Set up a monochromator scan for the filter's wait-time and **out-band** start, stop and step-size wavelengths given in Table 2.

35. **[O,T,L]** A6H Confirm that the script and monochromator scan have the same start, stop and step-size wavelengths. Set the dwell-time to at least 5 seconds between image sets (or to another appropriate value).

36. **[O,T,D]** A6H Load and execute prefix **433TAMBR02**, which captures 3 frames and 1 bias frame at the 100 mm focal length for each wavelength in the scan. Push continue in the GUI whenever the monochromator steps to the next wavelength position. The last wavelength position is for dark frames. The GUI's note field should have "GRATING=G123,WAVELENGTH=[nanometers]". The estimated duration is given in Table 2.

37. **[T]** A6H Close the lamp shutter. The last block of images in the script is for dark frames.

38. **[D]** A6H Double check that all image names, suffixes, and other parameters are recorded in Image log.

39. **[O,V]** Notes: ______

Data Validation

40. [V] AH Upload data to server.
41. [V] AH Run the "Spectral_Throughput_43_Validation" Jupyter notebook on the acquired data. This analysis can take place while the test continues.
    - Create preliminary spectral throughput curves for the filter.
    - Save results in the calibration records.
42. [V,D, L] Notes: ____

____

____

In-band and Out-of-band Measurements for Filter 1 of the Right Mastcam-Z

43. [T] ____ Set the monochromator to the filter's peak wavelength given in Table 2. 798, 800, 802
44. [[redacted],T,D] ____ Load and execute prefix **433TAMBR10**, which helps to find the optimal sub-frame, focus position, and exposure time. The GUI's note field should have "GRATING=G123,WAVELENGTH=[nanometers]".
45. [L,O,D] ____ Record the following values.
    - sub-frame window: ~~944, 416, 32, 208~~ 944, ~~432~~ 384, 32, 224 (preferably same as filter 0)
    - focus position: 666
    - exposure time: 0.5 (798) 0.6 (800) 0.6 (~~802~~ 796)
46. [L,O] ____ If necessary, update the scripts **433TAMBR11** and **433TAMBR12** for these sub-frame, focus position, and exposure time values.
47. [T] ____ Set up a monochromator scan for the filter's wait-time and **in-band** start, stop and step-size wavelengths given in Table 2.
48. [O,T,L] ____ Confirm that the script and monochromator scan have the same start, stop and step-size wavelengths. Set the dwell-time to at least 5 seconds between image sets (or to another appropriate value).
49. [[redacted],T,D] ____ Load and execute prefix **433TAMBR11**, which captures 10 frames and 3 bias frames at the 100 mm focal length for each wavelength in the scan. Push continue in the GUI whenever the monochromator steps to the next wavelength position. The last wavelength position is for dark frames. The GUI's note field should have "GRATING=G123,WAVELENGTH=[nanometers]". The estimated duration is given in Table 2.
50. [T] ____ Close the lamp shutter. The last block of images in the script is for dark frames.
51. [D] ____ Double check that all image names, suffixes, and other parameters are recorded in Image log.
52. [[redacted],V] ____ Make sure that the filter **1**'s transmission was measured to no more that 1% on both sides of its transition curve. Notes: ____________________

53. **[T]** [illegible] Set up a monochromator scan for the filter's wait-time and **out-band** start, stop and step-size wavelengths given in Table 2.
54. **[O,T,L]** [illegible] Confirm that the script and monochromator scan have the same start, stop and step-size wavelengths. Set the dwell-time to at least 5 seconds between image sets (or to another appropriate value).
55. **[O,T,D]** [illegible] Load and execute prefix **433TAMBR12**, which captures 3 frames and 1 bias frame at the 100 mm focal length for each wavelength in the scan. Push continue in the GUI whenever the monochromator steps to the next wavelength position. The last wavelength position is for dark frames. The GUI's note field should have "GRATING=G123,WAVELENGTH=[nanometers]". The estimated duration is given in Table 2.
56. **[T]** [illegible] Close the lamp shutter. The last block of images in the script is for dark frames.
57. **[D]** [illegible] Double check that all image names, suffixes, and other parameters are recorded in Image log.
58. **[O,V]** Notes: 17 mins 25.6°C

Data Validation

59. **[V]** [illegible] Upload data to server.
60. **[V]** [illegible] Run the "Spectral_Throughput_43_Validation" Jupyter notebook on the acquired data. This analysis can take place while the test continues.
    - Create preliminary spectral throughput curves for the filter.
    - Save results in the calibration records.
61. **[V,D, L]** Notes: ____

____

____

In-band and Out-of-band Measurements for Filter 2 of the Right Mastcam-Z

OPEN LAMP SHUTTER

62. **[T]** ____ Set the monochromator to the filter's peak wavelength given in Table 2.
63. **[[redacted],T,D]** ____ Load and execute prefix **433TAMBR20**, which helps to find the optimal sub-frame, focus position, and exposure time. The GUI's note field should have "GRATING=G123,WAVELENGTH=[nanometers]".
64. **[L,O,D]** ____ Record the following values,
    - sub-frame window: 944,384,32,208 (preferably same as filter 0)
    - focus position: ~~666~~ 606
    - exposure time: @864=0.8, 866=0.8, @868nm=0.8

    RUN TEST IMAGE AGAIN WITH NEW SUB-FRAME
65. **[L,O]** ____ If necessary, update the scripts **433TAMBR21** and **433TAMBR22** for these sub-frame, focus position, and exposure time values.
66. **[T]** ____ Set up a monochromator scan for the filter's wait-time and **in-band** start, stop and step-size wavelengths given in Table 2.
67. **[[redacted],T,L]** ____ Confirm that the script and monochromator scan have the same start, stop and step-size wavelengths. Set the dwell-time to at least 5 seconds between image sets (or to another appropriate value).
68. **[[redacted],T,D]** ____ Load and execute prefix **433TAMBR21**, which captures 10 frames and 3 bias frames at the 100 mm focal length for each wavelength in the scan. Push continue in the GUI whenever the monochromator steps to the next wavelength position. The last wavelength position is for dark frames. The GUI's note field should have "GRATING=G123,WAVELENGTH=[nanometers]". The estimated duration is given in Table 2.
69. **[T]** ____ Close the lamp shutter. The last block of images in the script is for dark frames.
70. **[D]** ____ Double check that all image names, suffixes, and other parameters are recorded in Image log.
71. **[O,V]** ____ Make sure that the filter **2**'s transmission was measured to no more that 1% on both sides of its transition curve. Notes: 25.5°C

OPEN LAMP SHUTTER

72. **[T]** ______ Set up a monochromator scan for the filter's wait-time and **out-band** start, stop and step-size wavelengths given in Table 2.
73. **[O,T,L]** ______ Confirm that the script and monochromator scan have the same start, stop and step-size wavelengths. Set the dwell-time to at least 5 seconds between image sets (or to another appropriate value).
74. **[[redacted],T,D]** ______ Load and execute prefix **433TAMBR22**, which captures 3 frames and 1 bias frame at the 100 mm focal length for each wavelength in the scan. Push continue in the GUI whenever the monochromator steps to the next wavelength position. The last wavelength position is for dark frames. The GUI's note field should have "GRATING=G123,WAVELENGTH=[nanometers]". The estimated duration is given in Table 2.
75. **[T]** ______ Close the lamp shutter. The last block of images in the script is for dark frames.
76. **[D]** ______ Double check that all image names, suffixes, and other parameters are recorded in Image log.
77. **[O,V]** Notes: OPEN LAMP SHUTTER
25.5°C

Data Validation

78. **[V]** [illegible] Upload data to server.
79. **[V]** [illegible] Run the "Spectral_Throughput_43_Validation" Jupyter notebook on the acquired data. This analysis can take place while the test continues.
    - Create preliminary spectral throughput curves for the filter.
    - Save results in the calibration records.
80. **[V,D,L]** Notes: GOOD.

In-band and Out-of-band Measurements for Filter 3 of the Right Mastcam-Z

OPEN LAMP SHUTTER

81. **[T]** Set the monochromator to the filter's peak wavelength given in Table 2.
82. **[[redacted],T,D]** Load and execute prefix **433TAMBR30**, which helps to find the optimal sub-frame, ~~focus position~~, and exposure time. The GUI's note field should have "GRATING=G123,WAVELENGTH=[nanometers]".
83. **[L,O,D]** Record the following values,
    - sub-frame window: 944 384 32 208 (preferably same as filter 0)
    - focus position: 606
    - exposure time: @906=1.0, @908=1.1, @904=1.0
84. **[L,O]** If necessary, update the scripts **433TAMBR31** and **433TAMBR32** for these sub-frame, focus position, and exposure time values.
85. **[T]** Set up a monochromator scan for the filter's wait-time and **in-band** start, stop and step-size wavelengths given in Table 2.
86. **[O,T,L]** Confirm that the script and monochromator scan have the same start, stop and step-size wavelengths. Set the dwell-time to at least 5 seconds between image sets (or to another appropriate value).
87. **[O,T,D]** Load and execute prefix **433TAMBR31**, which captures 10 frames and 3 bias frames at the 100 mm focal length for each wavelength in the scan. Push continue in the GUI whenever the monochromator steps to the next wavelength position. The last wavelength position is for dark frames. The GUI's note field should have "GRATING=G123,WAVELENGTH=[nanometers]". The estimated duration is given in Table 2.
88. **[T]** Close the lamp shutter. The last block of images in the script is for dark frames.
89. **[D]** Double check that all image names, suffixes, and other parameters are recorded in Image log.
90. **[O,V]** Make sure that the filter **3**'s transmission was measured to no more that 1% on both sides of its transition curve. Notes: OPEN LAMP SHUTTER
GUI BUG — COUNTS
25.3°C 359

91. **[T]** ___ Set up a monochromator scan for the filter's wait-time and **out-band** start, stop and step-size wavelengths given in Table 2.
92. **[O,T,L]** ___ Confirm that the script and monochromator scan have the same start, stop and step-size wavelengths. Set the dwell-time to at least 5 seconds between image sets (or to another appropriate value).
93. **[O,T,D]** ___ Load and execute prefix **433TAMBR32**, which captures 3 frames and 1 bias frame at the 100 mm focal length for each wavelength in the scan. Push continue in the GUI whenever the monochromator steps to the next wavelength position. The last wavelength position is for dark frames. The GUI's note field should have "GRATING=G123,WAVELENGTH=[nanometers]". The estimated duration is given in Table 2.
94. **[T]** ___ Close the lamp shutter. The last block of images in the script is for dark frames.
95. **[D]** ___ Double check that all image names, suffixes, and other parameters are recorded in Image log.
96. **[O,V]** Notes: OPEN LAMP SHUTTER
LIGHTS ON
25.2°C 5

97. [T] [illegible] Perform a full radiometer scan between 300 and 1100 nm in 2 nm increments with a 2 second dwell-time at each wavelength. Follow the procedure in "Monochromator_Manual". Be sure to save file as "**mono_scan_g123_300nm_1100nm_2nm_**" + "**YYMMDD_hhmm**" where the last numbers encode the scan's date and start time. The estimated duration is 20 minutes.

- Scan started at 19:27 and completed at 19:54.

STET

REMOVE RADIOMETER

Data Validation

98. [V] [illegible] Upload data to server.
99. [V] [illegible] Run the "Spectral_Throughput_43_Validation" Jupyter notebook on the acquired data. This analysis can take place while the test continues.
    - Create preliminary spectral throughput curves for the filter.
    - Save results in the calibration records.
100. [V,D, L] Notes: GOOD.

In-band and Out-of-band Measurements for Filter 4 of the Right Mastcam-Z

101. **[T]** [illegible] Set the monochromator to the filter's peak wavelength given in Table 2. 934 nm
102. **[O,T,D]** [illegible] Load and execute prefix **433TAMBR40**, which helps to find the optimal sub-frame, focus position, and exposure time. The GUI's note field should have "GRATING=G123,WAVELENGTH=[nanometers]".
103. **[L,O,D]** [illegible] Record the following values,
   - sub-frame window: 944, ~~384~~ 368, 32, 208 (preferably same as filter 0)
   - focus position: 606 3
   - exposure time: @934 = 1.4, @936 = 1.4, @938 = 1.4, @940 = 1.4
104. **[L,O]** [illegible] If necessary, update the scripts **433TAMBR41** and **433TAMBR42** for these sub-frame, focus position, and exposure time values.
105. **[T]** [illegible] Set up a monochromator scan for the filter's wait-time and **in-band** start, stop and step-size wavelengths given in Table 2.
106. **[O,T,L]** [illegible] Confirm that the script and monochromator scan have the same start, stop and step-size wavelengths. Set the dwell-time to at least 5 seconds between image sets (or to another appropriate value).
107. **[O,T,D]** [illegible] Load and execute prefix **433TAMBR41**, which captures 10 frames and 3 bias frames at the 100 mm focal length for each wavelength in the scan. Push continue in the GUI whenever the monochromator steps to the next wavelength position. The last wavelength position is for dark frames. The GUI's note field should have "GRATING=G123,WAVELENGTH=[nanometers]". The estimated duration is given in Table 2. ABORTED FIRST (4-nm) RUN, RESTARTED WITH 6-nm STEPS
108. **[T]** [illegible] Close the lamp shutter. The last block of images in the script is for dark frames.
109. **[D]** [illegible] Double check that all image names, suffixes, and other parameters are recorded in Image log.
110. **[O,V]** [illegible] Make sure that the filter **4**'s transmission was measured to no more that 1% on both sides of its transition curve. Notes: OPEN LAMP SHUTTER

____

____

111. **[T]** [illegible] Set up a monochromator scan for the filter's wait-time and **out-band** start, stop and step-size wavelengths given in Table 2.
112. **[O,T,L]** [illegible] Confirm that the script and monochromator scan have the same start, stop and step-size wavelengths. Set the dwell-time to at least 5 seconds between image sets (or to another appropriate value).
113. **[O,T,D]** [illegible] Load and execute prefix **433TAMBR42**, which captures 3 frames and 1 bias frame at the 100 mm focal length for each wavelength in the scan. Push continue in the GUI whenever the monochromator steps to the next wavelength position. The last wavelength position is for dark frames. The GUI's note field should have "GRATING=G123,WAVELENGTH=[nanometers]". The estimated duration is given in Table 2.
114. **[T]** [illegible] Close the lamp shutter. The last block of images in the script is for dark frames.
115. **[D]** [illegible] Double check that all image names, suffixes, and other parameters are recorded in Image log.
116. **[O,V]** Notes: ____

____

____

Data Validation

117. **[V]** [illegible] Upload data to server.
118. **[V]** [illegible] Run the "Spectral_Throughput_43_Validation" Jupyter notebook on the acquired data. This analysis can take place while the test continues.
    - Create preliminary spectral throughput curves for the filter.
    - Save results in the calibration records.
119. **[V,D, L]** Notes: GOOD

In-band and Out-of-band Measurements for Filter 5 of the Right Mastcam-Z

OPEN LAMP SHUTTER

120. **[T]** ___ Set the monochromator to the filter's peak wavelength given in Table 2. 973
121. **[O,T,D]** ___ Load and execute prefix **433TAMBR50**, which helps to find the optimal sub-frame, focus position, and exposure time. The GUI's note field should have "GRATING=G123,WAVELENGTH=[nanometers]".
122. **[L,O,D]** ___ Record the following values,
    - sub-frame window: 944, 368, 32, 208 (preferably same as filter 0)
    - focus position: ~~606~~ ~~546~~ 606
    - exposure time: @ 973 = 1.2 ms, @ 975 nm = 2.2, @ 971 = 2.4
123. **[L,O]** ___ If necessary, update the scripts **433TAMBR51** and **433TAMBR52** for these sub-frame, focus position, and exposure time values.
124. **[T]** ___ Set up a monochromator scan for the filter's wait-time and **in-band** start, stop and step-size wavelengths given in Table 2.
125. **[O,T,L]** ___ Confirm that the script and monochromator scan have the same start, stop and step-size wavelengths. Set the dwell-time to at least 5 seconds between image sets (or to another appropriate value).
126. **[O,T,D]** ___ Load and execute prefix **433TAMBR51**, which captures 10 frames and 3 bias frames at the 100 mm focal length for each wavelength in the scan. Push continue in the GUI whenever the monochromator steps to the next wavelength position. The last wavelength position is for dark frames. The GUI's note field should have "GRATING=G123,WAVELENGTH=[nanometers]". The estimated duration is given in Table 2.
127. **[T]** ___ Close the lamp shutter. The last block of images in the script is for dark frames.
128. **[D]** ___ Double check that all image names, suffixes, and other parameters are recorded in Image log. 25.3°C
129. **[O,V]** ___ Make sure that the filter **5**'s transmission was measured to no more that 1% on both sides of its transition curve. Notes: NO RED PIXELS

5 sec. ok? Y

 Date 5/5 Time 21:25 Initial [illegible]

OPEN LAMP SHUTTER

130. **[T]** [illegible] Set up a monochromator scan for the filter's wait-time and **out-band** start, stop and step-size wavelengths given in Table 2.

131. **[O,T,L]** [illegible] Confirm that the script and monochromator scan have the same start, stop and step-size wavelengths. Set the dwell-time to at least 5 seconds between image sets (or to another appropriate value).

132. **[O,T,D]** [illegible] Load and execute prefix **433TAMBR52**, which captures 3 frames and 1 bias frame at the 100 mm focal length for each wavelength in the scan. Push continue in the GUI whenever the monochromator steps to the next wavelength position. The last wavelength position is for dark frames. The GUI's note field should have "GRATING=G123,WAVELENGTH=[nanometers]". The estimated duration is given in Table 2.

133. **[T]** [illegible] Close the lamp shutter. The last block of images in the script is for dark frames.

134. **[D]** [illegible] Double check that all image names, suffixes, and other parameters are recorded in Image log.

135. **[O,V]** Notes: OPEN LAMP SHUTTER

25.0°C 547

Data Validation

136. [V] Za Upload data to server.
137. [V] Za Run the "Spectral_Throughput_43_Validation" Jupyter notebook on the acquired data. This analysis can take place while the test continues.
    - Create preliminary spectral throughput curves for the filter.
    - Save results in the calibration records.
138. [V,D, L] Notes: GOOD

In-band and Out-of-band Measurements for Filter 6 of the Right Mastcam-Z

139. **[T]** [initials] Set the monochromator to the filter's peak wavelength given in Table 2. 998
140. **[O,T,D]** [initials] Load and execute prefix **433TAMBR60**, which helps to find the optimal sub-frame, focus position, and exposure time. The GUI's note field should have "GRATING=G123,WAVELENGTH=[nanometers]".
141. **[L,O,D]** [initials] Record the following values,
    - sub-frame window: 944, 368, 32, 208 (preferably same as filter 0)
    - focus position: 606
    - exposure time: @ 998 = 4.3 msec, @1000 = 3.9, @1002 = 4.0
142. **[L,O]** [initials] If necessary, update the scripts **433TAMBR61** and **433TAMBR62** for these sub-frame, focus position, and exposure time values. 3.9 msec
143. **[T]** [initials] Set up a monochromator scan for the filter's wait-time and **in-band** start, stop and step-size wavelengths given in Table 2.
144. **[O,T,L]** [initials] Confirm that the script and monochromator scan have the same start, stop and step-size wavelengths. Set the dwell-time to at least 5 seconds between image sets (or to another appropriate value).
145. **[[redacted],T,D]** [initials] Load and execute prefix **433TAMBR61**, which captures 10 frames and 3 bias frames at the 100 mm focal length for each wavelength in the scan. Push continue in the GUI whenever the monochromator steps to the next wavelength position. The last wavelength position is for dark frames. The GUI's note field should have "GRATING=G123,WAVELENGTH=[nanometers]". The estimated duration is given in Table 2. ABORTED RESTARTED W/PROPER INTEGRARATION TIME, AFTER HALTING PROCESS
146. **[T]** [initials] Close the lamp shutter. The last block of images in the script is for dark frames. 306-1049, 25.5°C
147. **[D]** [initials] Double check that all image names, suffixes, and other parameters are recorded in Image log.
148. **[[redacted],V]** [initials] Make sure that the filter **6**'s transmission was measured to no more that 1% on both sides of its transition curve. Notes: OPEN LAMP SHUTTER

149. **[T]** [initials] Set up a monochromator scan for the filter's wait-time and **out-band** start, stop and step-size wavelengths given in Table 2.

150. **[[redacted],T,L]** [initials] Confirm that the script and monochromator scan have the same start, stop and step-size wavelengths. Set the dwell-time to at least 5 seconds between image sets (or to another appropriate value).

151. **[O,T,D]** [initials] Load and execute prefix **433TAMBR62**, which captures 3 frames and 1 bias frame at the 100 mm focal length for each wavelength in the scan. Push continue in the GUI whenever the monochromator steps to the next wavelength position. The last wavelength position is for dark frames. The GUI's note field should have "GRATING=G123,WAVELENGTH=[nanometers]". The estimated duration is given in Table 2.

152. **[T]** [initials] Close the lamp shutter. The last block of images in the script is for dark frames.

153. **[D]** [initials] Double check that all image names, suffixes, and other parameters are recorded in Image log.

154. **[O,V]** Notes: OPEN LAMP SHUTTER

~~LIGHTS~~
✓ UPDATE TABLE G2 ~~[illegible]~~ G3 BOUNDARY TO 700nm
✓ RE-RUN RØ IN-BAND ONLY
✓ LIGHTS ON
✓ RADIOMETER SCAN WITH G2/G3 @ 700nm
RESTORE TABLE TO DEFAULT
RADIOMETER ~~[illegible]~~ SCAN
~~GO TO LEFT~~
~~REMOVE RADIOMETER~~

 Date 5/5 Time 23:57 Initial [illegible]

UPDATE TABLE
RE-RUN R∅ IN-BAND ← DEFAULT GRATING ~~RADIOMETER SCAN~~
RESTORE TABLE

155. **[T]** [illegible] Perform a full radiometer scan between 300 and 1100 nm in 2 nm increments with a 2 second dwell-time at each wavelength. Follow the procedure in "Monochromator_Manual". Be sure to save file as "**mono_scan_g123_300nm_1100nm_2nm_**" + "**YYMMDD_hhmm**" where the last numbers encode the scan's date and start time. The estimated duration is 20 minutes.

- Scan started at 23:38 and completed at 00:06.

~~UPDATE TABLES~~

HEIGHTS ABOVE FLOOR OF:

~~BOTTOM OF ORANGE STAND TOP =~~

TOP OF MONOCHROMATOR TO CEILING (4 CORNERS)

TOP OF OPTICAL BENCH " " (CENTER) = 1.940 m

Data Validation

156. **[V]** [illegible] Upload data to server.
157. **[V]** [illegible] Run the "Spectral_Throughput_43_Validation" Jupyter notebook on the acquired data. This analysis can take place while the test continues.
    - Create preliminary spectral throughput curves for the filter.
    - Save results in the calibration records.
158. **[V,D, L]** Notes: GOOD.

Time Check 1

IF MORE THAN 1 HOUR AHEAD OF SCHEDULED END, CONTINUE WITH THE CONTINUOUS TESTS, OTHERWISE SKIP TO THE NEXT TIME CHECK

Scheduled End Time	Current Time	Time Ahead of Scheduled End
-	=	

159. **[D, L]** ____ Record the time in the table above and determine if there is time for more testing.
160. **[D, L]** Notes:

BEHIND SCHEDULE

~~161. [T] ____ Lights off~~ SKIP

In-band Measurements for Filter 7 of the Right Mastcam-Z

SKIP

162. **[T]** ____ Set the monochromator to the filter's peak wavelength given in Table 2.
163. **[O,T,D]** ____ Load and execute prefix **433TAMBR70**, which helps to find the optimal sub-frame, focus position, and exposure time. The GUI's note field should have "GRATING=G123,WAVELENGTH=[nanometers],WINDOW=1".
164. **[L,O,D]** ____ Record the following values,
    - sub-frame window: ______________________ (preferably same as filter 0)
    - focus position: ___________
    - exposure time: ___________
165. **[L,[redacted]]** ____ Update the **433TAMBR71** script for these sub-frame, focus position, and exposure time values. Be sure to add "WINDOW=1" to the note.
166. **[T]** ____ Set up a monochromator scan for the filter's wait-time and **in-band** start, stop and step-size wavelengths given in Table 2.
167. **[[redacted],T,L]** ____ Confirm that the script and monochromator scan have the same start, stop and step-size wavelengths. Set the dwell-time to at least 5 seconds between image sets (or to another appropriate value).
168. **[O,T,D]** ____ Load and execute prefix **433TAMBR71**, which captures 10 frames and 3 bias frames at the 100 mm focal length for each wavelength in the scan. Push continue in the GUI whenever the monochromator steps to the next wavelength position. The last wavelength position is for dark frames. The GUI's note field should have "GRATING=G123,WAVELENGTH=[nanometers], WINDOW=1". The estimated duration is given in Table 2.
169. **[T]** ____ Close the lamp shutter. The last block of images in the script is for dark frames.
170. **[D]** ____ Double check that all image names, suffixes, and other parameters are recorded in Image log.
171. **[O,V]** ____ Make sure that the filter 7's transmission was measured to no more that 1% on both sides of its transition curve. Notes: ______________________________

RESET MONOCHROMATOR G2/G3 TO ~~DEFAULT~~ 658 nm

172. **[T]** [illegible] Perform a full radiometer scan between 300 and 1100 nm in 2 nm increments with a 2 second dwell-time at each wavelength. Follow the procedure in "Monochromator_Manual". Be sure to save file as "**mono_scan_g123_300nm_1100nm_2nm_" + "YYMMDD_hhmm**" where the last numbers encode the scan's date and start time. The estimated duration is 15 minutes.

- Scan started at 00:12 and ~~completed~~ STOPPED at 00:36.

~~FAILED, RESTART~~ THROUGH → 928nm WHEN FAILED.
HUMIDITY = 49% FOR FIRST TIME.

Data Validation

173. **[V]** ____ Upload data to server.
174. **[V]** ____ Run the "Spectral_Throughput_43_Validation" Jupyter notebook on the acquired data. This analysis can take place while the test continues.
    - Create preliminary spectral throughput curves for the filter.
    - Save results in the calibration records.
175. **[V,D, L]** Notes: ____________________

Shutdown Procedure

176. **[D,T]** [illegible] Take digital pictures of the test setup. IMAGES 1-2.
177. **[D,O]** [illegible] Review entries in Image Log, GSE command log, and image headers.
178. **[D, L]** [illegible] Review calibration procedure and ensure that each task is initialed.
179. **[D, L]** Notes: TURNED OFF MONOCHROMATOR.

180. **[V, L]** [illegible] Before making the decision to break down the test setup, ensure that adequate data were acquired for the test requirements. See "MastcamZCalPlan" for these requirements.
181. **[V]** Notes:

Data Validator (signature) [illegible]

Date 05-MAY-19 Time 23:58

182. **[V, L]** [illegible] Give the go/no-go decision. Have enough data been acquired to fulfill test requirements? See "MastcamZCalPlan" for these requirements.
183. **[D, L]** [illegible] Update the Log Document.
184. **[L]** Notes:

Calibration Lead (signature) [illegible]

Date 5/6/19 Time 00:41

185. **[[redacted], L]** EJ Ensure that the camera and GSE are in a safe state.
186. **[O,D]** EJ Review the Image Log with the documentarian. Exchange high-fives.
187. **[[redacted]]** Notes: ____

____

____

Camera Operator (signature) Elsa Jensen

Date 5/5/19 Time 11:59pm

188. **[T]** [illegible] If the next test does not require the monochrometer, position it away from the chamber or bench. Otherwise, be sure not to move it. The next test is ___________ .
189. **[T]** [illegible] Ensure that all other test equipment is safely put away.
190. **[T]** Notes: ____

____

____

Technician (signature) [illegible]

Date 5/6/19 Time 12:53pm

191. **[D, L]** [illegible] Double-check this procedure and ensure that the top of each page has valid data, time and initials.

SKIP 192. **[D]** ____ Photo-scan this document, save it on the cloud, and file the hard-copy in the Log Binder. Upload the digital pictures taken during this test in the appropriate archive on the cloud. The required links are on the Wiki.

193. **[D]** [illegible] Double-check that every required cell the Image Log is accurately filled. When this is complete, print the Image Log and file it the Log Binder after this document.
194. **[D]** Notes: ____

____

____

Documentarian (signature) [illegible]

Date 5/5 2019 Time 11:57p.m.
