# Supplementary material for: Pre-Flight Calibration of the Mars 2020 Rover Mastcam Zoom (Mastcam-Z) Multispectral, Stereoscopic Imager
Source: Space Sci Rev. 2021 Feb 18;217(2):29. doi: 10.1007/s11214-021-00795-x (PMC7892537; doi:10.1007/s11214-021-00795-x)
Supplement: Supplementary file 1 — (ZIP 98.6 MB) [file 11214_2021_795_MOESM1_ESM.zip › CalPro_433_434_L_Spectral_Throughput_v2_02_copy.pdf]

Spectral Throughput Procedure for the Left Mastcam-Z for Ambient Testing at MSSS

(Pro. 4.3.3-4)

[Procedure version 2.02, prepared by the Mastcam-Z calibration team at Cornell University]

These measurements are performed on the camera and at the temperature designated below as specified in the Mastcam-Z Calibration Plan,

Unit Under Test:

Left FM __X__ Right FM ______ EQM_____ Other____________

These measurements are performed at temperature:

-35°C______ - 10°C______ +5°C______ Ambient__X__ Other__________

These measurements are performed at,

MSSS______ ASU__X__ Other________

Date ~~5/4/19~~ 5/6/19 **Start Time** ~~8:00am~~ 16:45 **End Time** ________

Estimated Duration 6.0 hours

Scheduled Start Time 7:30 am **Sch. End Time** 13:30

Calibration Lead [L] Alex Hayes / Melissa Rice **Documentarian [D]** Alexis Parkinson

Camera Operator [[redacted]] Tex **Technician [T]** Christian Tate / Paul / Ernest

Data Validator [V] Paul Corlies / Ole **Other** ________

Change Log

Version	Name	Change
v1_01<br>17 Sep 2018	C. Tate	(first draft)
v1_11<br>1 Nov 2018	C. Tate	Procedure edits prior to EQM testing
v1_13<br>1 Dec. 2018	C. Tate	Procedure edits after EQM testing
v2_02<br>5 May 2019	C. Tate	Approved version prior to FM testing

Document Approval

[signature] 5/5/19
Approved by James Bell — Date
Mastcam-Z PI
Arizona State University

[signature] 5/4/19
Approved by Alexander Hayes — Date
Mastcam-Z Calibration Working Group Lead, Cornell University

Approved by Justin Maki — Date
Mastcam-Z Deputy PI and Investigation Scientist, Jet Propulsion Laboratory

[signature] 5/5/19
Approved by Ken Herkenhoff — Date
Mastcam-Z Co-Investigator
USGS

Approved by — Date
Melissa Rice, Co-I
W

[signature] 5/5/19
Approved by Christian Tate — Date
Procedure Author
Cornell University

Table of Contents

SPECTRAL THROUGHPUT PROCEDURE FOR THE LEFT MASTCAM-Z FOR AMBIENT TESTING AT MSSS 1

CHANGE LOG 2
DOCUMENT APPROVAL 2
TEST DESCRIPTION 3
SOFTWARE PREPARATION 5
Table 1. File naming convention for the camera script prefixes and frame filenames: "AAABBBBCDD" 5
HARDWARE PREPARATION 7
Figure 1. MSSS Floor Plan for Geometric Testing in the Cleanroom. 7
MONOCHROMATOR WAVELENGTHS 10
*Table 2. Wavelengths for the Left Mastcam-Z filters **0-7** 10*
IN-BAND AND OUT-OF-BAND MEASUREMENTS FOR FILTER 0 OF THE LEFT MASTCAM-Z 11
DATA VALIDATION 13
IN-BAND AND OUT-OF-BAND MEASUREMENTS FOR FILTER 1 OF THE LEFT MASTCAM-Z 14
DATA VALIDATION 16
IN-BAND AND OUT-OF-BAND MEASUREMENTS FOR FILTER 2 OF THE LEFT MASTCAM-Z 17
DATA VALIDATION 19
IN-BAND AND OUT-OF-BAND MEASUREMENTS FOR FILTER 3 OF THE LEFT MASTCAM-Z 20
DATA VALIDATION 22
IN-BAND AND OUT-OF-BAND MEASUREMENTS FOR FILTER 4 OF THE LEFT MASTCAM-Z 23
DATA VALIDATION 25
IN-BAND AND OUT-OF-BAND MEASUREMENTS FOR FILTER 5 OF THE LEFT MASTCAM-Z 26
DATA VALIDATION 28
IN-BAND AND OUT-OF-BAND MEASUREMENTS FOR FILTER 6 OF THE LEFT MASTCAM-Z 29
DATA VALIDATION 32
TIME CHECK 1 32
IN-BAND MEASUREMENTS FOR FILTER 7 OF THE LEFT MASTCAM-Z 33
DATA VALIDATION 34

SHUTDOWN PROCEDURE 35

Test Description

Excerpt from the Calibration Plan 4.3,

> The objective of this test is to measure spectral transmission (spectral response functions) for each filter on each Mastcam-Z camera's filter wheel in a through-system sense—that

is, the spectral transmission through the optics, filter, and microlenses/Bayer Pattern Filters of the CCD detector.

Measurements from the vendor as well as experience with MSL has demonstrated little dependence with temperature; however, it is highly desirable to conduct these tests over a range of appropriate temperatures in a thermal vacuum chamber at Mars atmospheric pressure (or lower) to fully characterize the filters' thermal response.

Software Preparation

The software and files required for this test are prepared well in advance of test day. This checklist ensures that the following are present, debugged, and executable: (1) all fast-look scripts, (2) automated header generation of all relevant camera parameters, target positioning, and metadata, (3) all camera scripts that command the camera unit, and (4) the directories/file-paths pointing to the data repositories of this specific test.

Table 1. File naming convention for the camera script prefixes and frame filenames: "AAABBBBCDD"

Code	Name	Example	Value
"AAA"	Calibration Plan Section	"433" = Cal. Plan 4.3.3 chapter 4, section 3, subsection 3	433/434
"BBBB"	Location of test or ASU Chamber temperature	"MSSS" = test at MSSS, "TN10" = ASU TVAC -10C, …	TAMB
"C"	Camera unit under test	"L" = Left Mastcam-Z, "R" = Right Mastcam-Z, "E" =EQM, "C" =COTS	L
"DD"	Part of test (radiance value)	"00" = test set up, "01" = first radiance value …	00-99

1. **[D]** [initials] Look up the daily calibration schedule and record the scheduled start and end time of this test on the cover page of this document. Also fill out and double-check the other information on the cover page.
2. **[D]** ____ Ensure that all supplemental manuals are on hand. These are,
   - Monochromator_Manual,
   - Validator_Manual, Documentarian_Manual
   - MastcamZCalPlan
3. **[D]** [initials] Ensure that the Image Log is present and ready to use. Find and open the Google Sheets file "Image_Log_43". There is a link on the Wiki.
4. **[V]** [initials] Check that all Calgorithms fast-look and validation scripts are present, up-to-date, and ready to analyze test output. Find and open the "Spectral_Throughput_43_Validation" Jupyter notebook. There is a link on the Wiki.

5. [O] Check that all camera scripts required for this test are present, up-to-date and ready to command the ground support equipment (GSE). These are,
   - **433TAMBL00 - 433TAMBL02**
   - **433TAMBL10 - 433TAMBL12**
   - **433TAMBL20 - 433TAMBL22**
   - **433TAMBL30 - 433TAMBL32**
   - **433TAMBL40 - 433TAMBL42**
   - **433TAMBL50 - 433TAMBL52**
   - **433TAMBL60 - 433TAMBL62**
   - **434TAMBL70 - 434TAMBL72**
6. **[O,V,D, L]** Notes:

___

___

___

Hardware Preparation

This procedure is for the ambient cleanroom testing at MSSS. Figure 1 shows the nominal layout of the cleanroom chamber, workspace, Mastcam-Zs, ground support equipment (GSE), targets, sources, and other equipment necessary.

Figure 1. MSSS Floor Plan for Geometric Testing in the Cleanroom.

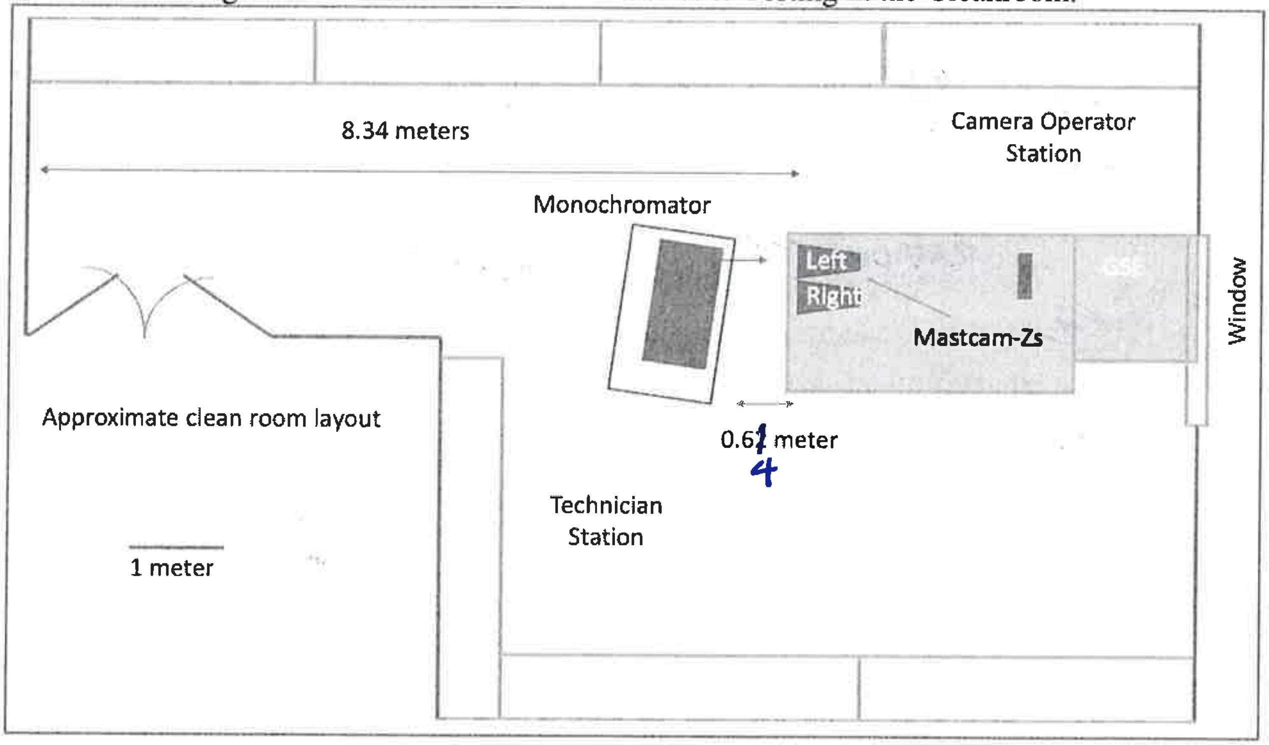

7. [T,O, L] ____ Ensure that all personnel in the cleanroom are following the cleanroom practices for electrostatic discharge and proper clothing.
8. [T] ____ Double check that the ionizers are flowing over the Mastcam-Zs.
9. [O,T] ____ If not already done, power on the Left Mastcam-Z and the GSE.
10. [O,T] ____ Ensure that the lights, monochromator, Mastcam-Zs and GSE wires are secure, kink-free, and do not present tripping hazards when the lights are turned off.
11. [O,D] ____ Check the camera temperature and ensure nominal operation. Record the following temperatures:
    - Left Mastcam-Z CCD temp 21.4°C

- Right Mastcam-Z CCD temp 21.6°C

12. **[D]** [illegible] Record the following environmental information:
    - Cleanroom temperature 67.2°F pressure _______ humidity 66%

13. **[[redacted],D, L]** Notes:

____

____

____

14. **[D,T]** [illegible] Take ^PIX^ of the monochromator and the whole test/GSE set-up.

15. **[T]** [illegible] Power on the monochromator and radiometer, which takes about 40-120 minutes to warm up and stabilize. Follow the procedure in "Monochromator_Manual".
    - The ~~monochromator~~ RADIOMETER was turned on at 14:55.

16. **[T]** [illegible] Make sure that the monochromator's secondary filter-while in the clear position (i.e. that the ND2 filter **is not** in place.)

17. **[T]** [illegible] Confirm that the entrance and exit slits are at 600 microns (i.e. that no one has changed them).

18. **[T]** ____ After the monochromator and radiometer have been on for at least 10 minutes, perform a full radiometer scan between 300 and 1100 nm in 2 nm increments with a 2 second dwell-time at each wavelength. Follow the procedure in "Monochromator_Manual". Be sure to save file as **"mono_scan_g123_300nm_1100nm_2nm_" + "YYMMDD_hhmm"** where the last numbers encode the scan's date and start time. The estimated duration is 20 minutes.
    - Scan started at ____________ and completed at ____________.

SKIP

TURN ON MONOCHROMATOR

19. **[T]** [illegible] After the monochromator and radiometer have been on for at least 60 minutes, perform a full radiometer scan between 300 and 1100 nm in 2 nm increments with a 2 second dwell-time at each wavelength. Follow the procedure in "Monochromator_Manual". Be sure to save file as

GRATING 2/3 CROSSOVER = 652 nm

"**mono_scan_g123_300nm_1100nm_2nm_**" + "**YYMMDD_hhmm**" where the last numbers encode the scan's date and start time. The estimated duration is 20 minutes.

- Scan started at 18:38 and completed at 19:05.

20. ~~**[D,T]** ____ Take of the monochromator and the whole test/GSE set-up.~~ P/X

21. **[T,D, L]** Notes:

~~AUTOFOCUS ON NEWPORT LOGO.~~

~~OPEN SLIT COVER~~

✓ REMOVE RADIOMETER, LIGHTS OFF

GO TO STEP 81.

DISTANCE TO CEILING FROM TOP OF MONOCHROMATOR = 1.833 m

Monochromator Wavelengths

Table 2. Wavelengths for the Left Mastcam-Z filters **0-7**

Filter	Peak Wavelength [nm]	Exposure In-band [ms]	Start In-band	Stop In-band	Step-size In-band	Minutes for In-band Scan	Exposure Out-band [ms]	Start Out-band	Stop Out-band	Step-size Out	Minutes for Out-band Scan
**L0**	625 ~~600~~ [illegible] ~~666~~	1.5	400	710	2	19.2	X ~~300~~	302	1100 4	4	25.0
**L1**	718 ~~800~~	30	780	826	2	3.9	1500	302	1100	6	23.6
**L2**	750	15	730	772	2	3.6	1500	302	1100	6	23.6
**L3**	676	15	654	698	2	3.7	1500	302	1100	6	23.6
**L4**	~~600~~	15	578	620	2	3.6	1500	302	1100	6	23.6
**L5**	527	30	500	550	2	4.2	1200	302	1100	6	33.6
**L6**	450	150	410	470	2	5.4	1500	302	1100	6	23.6
**L7**	590	20000	490	690	8	46.9	~~40000~~	~~340~~	~~1100~~	~~20~~	~~82.6~~

↑ factor of 10 too large

↑ multiply in-band by ~~100~~ 50

		IN	OUT
R0	666 nm	✓	✓
R1	800 nm	✓	✓
R2	866 nm	✓	✓
R3	908 nm	✓	✓
R4	936 nm	✓	✓
R5	975 nm	✓	✓
R6	1000 nm		
R7	880 nm		

Date 5/6 Time ______ Initial ______

5/7 Monochromator Lamps + Power Meter/Radiometer 7:23am

In-band and Out-of-band Measurements for Filter 0 of the Left Mastcam-Z

22. **[T]** Fla Lights off
23. **[T]** Th Set the monochromator to the filter's peak wavelength given in Table 2. 625 nm
24. **[[redacted],T,D]** ____ Load and execute prefix **433TAMBL00**, which helps to find the optimal sub-frame, focus position, and exposure time. The GUI's note field should have "GRATING=G123,WAVELENGTH=[nanometers]". AUTO EXP 120 — Full Frame 20 100 mm near auto auto exposure,
25. **[[redacted],T]** ____ Position the monochromator as need to aim the slit near the camera's boresight. Rerun script **433TAMBL00** as needed.
26. **[L,O,D]** ____ Record the following values,
    - sub-frame window: ______________
    - focus position: ______________
    - exposure time: @ 625 = ______________
27. **[L,O]** ____ Update the **433TAMBL**** scripts for this sub-frame, focus position, and exposure time values.
28. **[T]** ____ Set up a monochromator scan for the filter's wait-time and **in-band** start, stop and step-size wavelengths given in Table 2.
29. **[[redacted],T,L]** ____ Confirm that the script and monochromator scan have the same start, stop and step-size wavelengths. Set the dwell-time to at least 5 seconds between image sets (or to another appropriate value).
30. **[[redacted],T,D]** ____ Load and execute prefix **433TAMBL01**, which captures 10 frames and 3 bias frames at the 100 mm focal length for each wavelength in the scan. Push continue in the GUI whenever the monochromator steps to the next wavelength position. The last wavelength position is for dark frames. The GUI's note field should have "GRATING=G123,WAVELENGTH=[nanometers]". The estimated duration is given in Table 2.
31. **[T]** ____ Close the lamp shutter. The last block of images in the script is for dark frames.
32. **[D]** ____ Double check that all image names, suffixes, and other parameters are recorded in Image log.

See (V2)

33. **[O,V]** ____ Make sure that the filter **0**'s transmission was measured to no more that 1% on both sides of its transition curve. Notes: ____

34. **[T]** ____ Set up a monochromator scan for the filter's wait-time and **out-band** start, stop and step-size wavelengths given in Table 2.
35. **[O,T,L]** ____ Confirm that the script and monochromator scan have the same start, stop and step-size wavelengths. Set the dwell-time to at least 5 seconds between image sets (or to another appropriate value).
36. **[O,T,D]** ____ Load and execute prefix **433TAMBL02**, which captures 3 frames and 1 bias frame at the 100 mm focal length for each wavelength in the scan. Push continue in the GUI whenever the monochromator steps to the next wavelength position. The last wavelength position is for dark frames. The GUI's note field should have "GRATING=G123,WAVELENGTH=[nanometers]". The estimated duration is given in Table 2.
37. **[T]** ____ Close the lamp shutter. The last block of images in the script is for dark frames.
38. **[D]** ____ Double check that all image names, suffixes, and other parameters are recorded in Image log.
39. **[O,V]** Notes: ____

see V2

Data Validation

40. **[V]** ____ Upload data to server.
41. **[V]** ____ Run the "Spectral_Throughput_43_Validation" Jupyter notebook on the acquired data. This analysis can take place while the test continues.
    - Create preliminary spectral throughput curves for the filter.
    - Save results in the calibration records.
42. **[V,D, L]** Notes: ____

____

____

See VZ

In-band and Out-of-band Measurements for Filter 1 of the Left Mastcam-Z

43. **[T]** ____ Set the monochromator to the filter's peak wavelength given in Table 2.
44. **[[redacted],T,D]** ____ Load and execute prefix **433TAMBL10**, which helps to find the optimal sub-frame, focus position, and exposure time. The GUI's note field should have "GRATING=G123,WAVELENGTH=[nanometers]".
45. **[L,O,D]** ____ Record the following values,
    - sub-frame window: ________________________ (preferably same as filter 0)
    - focus position: ____________
    - exposure time: ____________

46. **[L,O]** ____ If necessary, update the scripts **433TAMBL11** and **433TAMBL12** for these sub-frame, focus position, and exposure time values.
47. **[T]** ____ Set up a monochromator scan for the filter's wait-time and **in-band** start, stop and step-size wavelengths given in Table 2.
48. **[[redacted],T,L]** ____ Confirm that the script and monochromator scan have the same start, stop and step-size wavelengths. Set the dwell-time to at least 5 seconds between image sets (or to another appropriate value).
49. **[[redacted],T,D]** ____ Load and execute prefix **433TAMBL11**, which captures 10 frames and 3 bias frames at the 100 mm focal length for each wavelength in the scan. Push continue in the GUI whenever the monochromator steps to the next wavelength position. The last wavelength position is for dark frames. The GUI's note field should have "GRATING=G123,WAVELENGTH=[nanometers]". The estimated duration is given in Table 2.
50. **[T]** ____ Close the lamp shutter. The last block of images in the script is for dark frames.
51. **[D]** ____ Double check that all image names, suffixes, and other parameters are recorded in Image log.
52. **[[redacted],V]** ____ Make sure that the filter **1**'s transmission was measured to no more that 1% on both sides of its transition curve. Notes: ______________________________________

____

____

See V2

Date ______ Time ______ Initial ______

53. **[T]** ____ Set up a monochromator scan for the filter's wait-time and **out-band** start, stop and step-size wavelengths given in Table 2.
54. **[O,T,L]** ____ Confirm that the script and monochromator scan have the same start, stop and step-size wavelengths. Set the dwell-time to at least 5 seconds between image sets (or to another appropriate value).
55. **[O,T,D]** ____ Load and execute prefix **433TAMBL12**, which captures 3 frames and 1 bias frame at the 100 mm focal length for each wavelength in the scan. Push continue in the GUI whenever the monochromator steps to the next wavelength position. The last wavelength position is for dark frames. The GUI's note field should have "GRATING=G123,WAVELENGTH=[nanometers]". The estimated duration is given in Table 2.
56. **[T]** ____ Close the lamp shutter. The last block of images in the script is for dark frames.
57. **[D]** ____ Double check that all image names, suffixes, and other parameters are recorded in Image log.
58. **[O,V]** Notes: ____

____

____

See V2

Data Validation

59. **[V]** ____ Upload data to server.
60. **[V]** ____ Run the "Spectral_Throughput_43_Validation" Jupyter notebook on the acquired data. This analysis can take place while the test continues.
    - Create preliminary spectral throughput curves for the filter.
    - Save results in the calibration records.
61. **[V,D, L]** Notes: ____

____

____

See V2

Date ______ Time ______ Initial ______

In-band and Out-of-band Measurements for Filter 2 of the Left Mastcam-Z

62. **[T]** ____ Set the monochromator to the filter's peak wavelength given in Table 2.
63. **[O,T,D]** ____ Load and execute prefix **433TAMBL20**, which helps to find the optimal sub-frame, focus position, and exposure time. The GUI's note field should have "GRATING=G123,WAVELENGTH=[nanometers]".
64. **[L,O,D]** ____ Record the following values,
    - sub-frame window: ________________________ (preferably same as filter 0)
    - focus position: ____________
    - exposure time: ____________
65. **[L,O]** ____ If necessary, update the scripts **433TAMBL21** and **433TAMBL22** for these sub-frame, focus position, and exposure time values.
66. **[T]** ____ Set up a monochromator scan for the filter's wait-time and **in-band** start, stop and step-size wavelengths given in Table 2.
67. **[O,T,L]** ____ Confirm that the script and monochromator scan have the same start, stop and step-size wavelengths. Set the dwell-time to at least 5 seconds between image sets (or to another appropriate value).
68. **[[redacted],T,D]** ____ Load and execute prefix **433TAMBL21**, which captures 10 frames and 3 bias frames at the 100 mm focal length for each wavelength in the scan. Push continue in the GUI whenever the monochromator steps to the next wavelength position. The last wavelength position is for dark frames. The GUI's note field should have "GRATING=G123,WAVELENGTH=[nanometers]". The estimated duration is given in Table 2.
69. **[T]** ____ Close the lamp shutter. The last block of images in the script is for dark frames.
70. **[D]** ____ Double check that all image names, suffixes, and other parameters are recorded in Image log.
71. **[O,V]** ____ Make sure that the filter **2**'s transmission was measured to no more that 1% on both sides of its transition curve. Notes: ______________________________

See V2

____

____

72. **[T]** ____ Set up a monochromator scan for the filter's wait-time and **out-band** start, stop and step-size wavelengths given in Table 2.
73. **[[redacted],T,L]** ____ Confirm that the script and monochromator scan have the same start, stop and step-size wavelengths. Set the dwell-time to at least 5 seconds between image sets (or to another appropriate value).
74. **[O,T,D]** ____ Load and execute prefix **433TAMBL22**, which captures 3 frames and 1 bias frame at the 100 mm focal length for each wavelength in the scan. Push continue in the GUI whenever the monochromator steps to the next wavelength position. The last wavelength position is for dark frames. The GUI's note field should have "GRATING=G123,WAVELENGTH=[nanometers]". The estimated duration is given in Table 2.
75. **[T]** ____ Close the lamp shutter. The last block of images in the script is for dark frames.
76. **[D]** ____ Double check that all image names, suffixes, and other parameters are recorded in Image log.
77. **[O,V]** Notes: ____

____

____

See V2

Data Validation

78. [V] ____ Upload data to server.
79. [V] ____ Run the "Spectral_Throughput_43_Validation" Jupyter notebook on the acquired data. This analysis can take place while the test continues.
    - Create preliminary spectral throughput curves for the filter.
    - Save results in the calibration records.
80. [V,D, L] Notes: ____

____

____

REPEAT L3 IN-BAND ONLY WITH GRATING 2/3 WAVELENGTH = 700nm

GO TO STEP 97.

In-band and Out-of-band Measurements for Filter 3 of the Left Mastcam-Z

81. **[T]** [initials] Set the monochromator to the filter's peak wavelength given in Table 2. 674 nm
82. **[O,T,D]** [initials] Load and execute prefix **433TAMBL30**, which helps to find the optimal sub-frame, focus position, and exposure time. The GUI's note field should have "GRATING=G123,WAVELENGTH=[nanometers]". 4 sec. exposure! @676 (BAD ALIGNMENT)
83. **[L,O,D]** [initials] Record the following values.
    - sub-frame window: ~~800, 528~~ 912, 528 32, 208
    - focus position: ~~666 666~~ 726
    - exposure time: @674 = 0.4 @676 = 0.4, @672nm = 0.4

84. **[L,O]** [initials] If necessary, update the scripts **433TAMBL31** and **433TAMBL32** for these sub-frame, focus position, and exposure time values.
85. **[T]** [initials] Set up a monochromator scan for the filter's wait-time and **in-band** start, stop and step-size wavelengths given in Table 2.
86. **[O,T,L]** [initials] Confirm that the script and monochromator scan have the same start, stop and step-size wavelengths. Set the dwell-time to at least 5 seconds between image sets (or to another appropriate value).
87. **[O,T,D]** [initials] Load and execute prefix **433TAMBL31**, which captures 10 frames and 3 bias frames at the 100 mm focal length for each wavelength in the scan. Push continue in the GUI whenever the monochromator steps to the next wavelength position. The last wavelength position is for dark frames. The GUI's note field should have "GRATING=G123,WAVELENGTH=[nanometers]". The estimated duration is given in Table 2.
88. **[T]** [initials] Close the lamp shutter. The last block of images in the script is for dark frames.
89. **[D]** [initials] Double check that all image names, suffixes, and other parameters are recorded in Image log.
90. **[O,V]** [initials] Make sure that the filter **3**'s transmission was measured to no more that 1% on both sides of its transition curve. Notes: 24.8°C

OPEN LAMP SHUTTER

91. **[T]** [illegible] Set up a monochromator scan for the filter's wait-time and **out-band** start, stop and step-size wavelengths given in Table 2.
92. **[O,T,L]** [illegible] Confirm that the script and monochromator scan have the same start, stop and step-size wavelengths. Set the dwell-time to at least ~~5~~ 4 seconds between image sets (or to another appropriate value).
93. **[O,T,D]** [illegible] Load and execute prefix **433TAMBL32**, which captures 3 frames and 1 bias frame at the 100 mm focal length for each wavelength in the scan. Push continue in the GUI whenever the monochromator steps to the next wavelength position. The last wavelength position is for dark frames. The GUI's note field should have "GRATING=G123,WAVELENGTH=[nanometers]". The estimated duration is given in Table 2.
94. **[T]** [illegible] Close the lamp shutter. The last block of images in the script is for dark frames.
95. **[D]** [illegible] Double check that all image names, suffixes, and other parameters are recorded in Image log.
96. **[O,V]** Notes: OPEN LAMP SHUTTER

GRATING 2/3 CROSSOVER WAVELENGTH = 700nm

97. **[T]** ____ Perform a full radiometer scan between 300 and 1100 nm in 2 nm increments with a 2 second dwell-time at each wavelength. Follow the procedure in "Monochromator_Manual". Be sure to save file as "**mono_scan_g123_300nm_1100nm_2nm_**" + "**YYMMDD_hhmm**" where the last numbers encode the scan's date and start time. The estimated duration is 20 minutes.
    - Scan started at ____________ and completed at _____________.

Data Validation

98. **[V]** [initials] Upload data to server.
99. **[V]** [initials] Run the "Spectral_Throughput_43_Validation" Jupyter notebook on the acquired data. This analysis can take place while the test continues.
    - Create preliminary spectral throughput curves for the filter.
    - Save results in the calibration records.
100. **[V,D, L]** Notes: GOOD

In-band and Out-of-band Measurements for Filter 4 of the Left Mastcam-Z

101. **[T]** ____ Set the monochromator to the filter's peak wavelength given in Table 2. 600 nm
102. **[O,T,D]** ____ Load and execute prefix **433TAMBL40**, which helps to find the optimal sub-frame, focus position, and exposure time. The GUI's note field should have "GRATING=G123,WAVELENGTH=[nanometers]".
103. **[L,O,D]** ____ Record the following values,
    - sub-frame window: 912, 528, 32, 208 (preferably same as filter 0)
    - focus position: 726
    - exposure time: @600nm = 0.7, @602 = 0.6, @604 = 0.5, @606 = 0.5, @608 = 0.5, @610 = 0.5
104. **[L,O]** ____ If necessary, update the scripts **433TAMBL41** and **433TAMBL42** for these sub-frame, focus position, and exposure time values.
105. **[T]** ____ Set up a monochromator scan for the filter's wait-time and **in-band** start, stop and step-size wavelengths given in Table 2.
106. **[O,T,L]** ____ Confirm that the script and monochromator scan have the same start, stop and step-size wavelengths. Set the dwell-time to at least 5 seconds between image sets (or to another appropriate value).
107. **[[redacted],T,D]** ____ Load and execute prefix **433TAMBL41**, which captures 10 frames and 3 bias frames at the 100 mm focal length for each wavelength in the scan. Push continue in the GUI whenever the monochromator steps to the next wavelength position. The last wavelength position is for dark frames. The GUI's note field should have "GRATING=G123,WAVELENGTH=[nanometers]". The estimated duration is given in Table 2.
108. **[T]** ____ Close the lamp shutter. The last block of images in the script is for dark frames.
109. **[D]** ____ Double check that all image names, suffixes, and other parameters are recorded in Image log.
110. **[[redacted],V]** ____ Make sure that the filter **4**'s transmission was measured to no more that 1% on both sides of its transition curve. Notes: GOOD.

OPEN LAMP SHUTTER

111. **[T]** [initials] Set up a monochromator scan for the filter's wait-time and **out-band** start, stop and step-size wavelengths given in Table 2.

112. **[O,T,L]** [initials] Confirm that the script and monochromator scan have the same start, stop and step-size wavelengths. Set the dwell-time to at least ~~6~~ 4 seconds between image sets (or to another appropriate value).

113. **[[redacted],T,D]** [initials] Load and execute prefix **433TAMBL42**, which captures 3 frames and 1 bias frame at the 100 mm focal length for each wavelength in the scan. Push continue in the GUI whenever the monochromator steps to the next wavelength position. The last wavelength position is for dark frames. The GUI's note field should have "GRATING=G123,WAVELENGTH=[nanometers]". The estimated duration is given in Table 2.

114. **[T]** [initials] Close the lamp shutter. The last block of images in the script is for dark frames.

115. **[D]** [initials] Double check that all image names, suffixes, and other parameters are recorded in Image log.

116. **[O,V]** Notes: ______________________________

Data Validation

117. [V] ~~Upload~~ Upload data to server.
118. [V] ~~Run~~ the "Spectral_Throughput_43_Validation" Jupyter notebook on the acquired data. This analysis can take place while the test continues.
    - Create preliminary spectral throughput curves for the filter.
    - Save results in the calibration records.
119. [V,D, L] Notes: OPEN LAMP SHUTTER
STRANGE PLOT, BLUE RESPONSE SO LOW
THAT NEGATIVE AFTER BIAS SUBTRACTION.

In-band and Out-of-band Measurements for Filter 5 of the Left Mastcam-Z

120. **[T]** [illegible] Set the monochromator to the filter's peak wavelength given in Table 2. 527 nm
121. **[O,T,D]** ____ Load and execute prefix **433TAMBL50**, which helps to find the optimal sub-frame, focus position, and exposure time. The GUI's note field should have "GRATING=G123,WAVELENGTH=[nanometers]".
122. **[L,O,D]** [illegible] Record the following values,
    - sub-frame window: 912, 528, 32, 208
    - focus position: 726
    - exposure time: @527=0.7, @529 nm=0.7, @531=0.7, @533=0.7
123. **[L,O]** [illegible] If necessary, update the scripts **433TAMBL51** and **433TAMBL52** for these sub-frame, focus position, and exposure time values.
124. **[T]** [illegible] Set up a monochromator scan for the filter's wait-time and **in-band** start, stop and step-size wavelengths given in Table 2.
125. **[O,T,L]** [illegible] Confirm that the script and monochromator scan have the same start, stop and step-size wavelengths. Set the dwell-time to at least 5 seconds between image sets (or to another appropriate value).
126. **[O,T,D]** [illegible] Load and execute prefix **433TAMBL51**, which captures 10 frames and 3 bias frames at the 100 mm focal length for each wavelength in the scan. Push continue in the GUI whenever the monochromator steps to the next wavelength position. The last wavelength position is for dark frames. The GUI's note field should have "GRATING=G123,WAVELENGTH=[nanometers]". The estimated duration is given in Table 2. DIDN'T WAIT EXTRA SECOND BEFORE IMAGING, ESP. 544
127. **[T]** [illegible] Close the lamp shutter. The last block of images in the script is for dark frames.
128. **[D]** [illegible] Double check that all image names, suffixes, and other parameters are recorded in Image log.
129. **[O,V]** [illegible] Make sure that the filter **5**'s transmission was measured to no more that 1% on both sides of its transition curve. Notes: GOOD.

Date 5/6 Time 21:33 Initial

OPEN LAMP SHUTTER

130. **[T]** ____ Set up a monochromator scan for the filter's wait-time and **out-band** start, stop and step-size wavelengths given in Table 2.
131. **[O,T,L]** ____ Confirm that the script and monochromator scan have the same start, stop and step-size wavelengths. Set the dwell-time to at least 5 seconds between image sets (or to another appropriate value).
132. **[O,T,D]** ____ Load and execute prefix **433TAMBL52**, which captures 3 frames and 1 bias frame at the 100 mm focal length for each wavelength in the scan. Push continue in the GUI whenever the monochromator steps to the next wavelength position. The last wavelength position is for dark frames. The GUI's note field should have "GRATING=G123,WAVELENGTH=[nanometers]". The estimated duration is given in Table 2.
133. **[D]** ____ Double check that all image names, suffixes, and other parameters are recorded in Image log.
134. **[[redacted],V]** Notes: ____________________

Data Validation

135. **[V]** Zu Upload data to server.
136. **[V]** ____ Run the "Spectral_Throughput_43_Validation" Jupyter notebook on the acquired data. This analysis can take place while the test continues.
    - Create preliminary spectral throughput curves for the filter.
    - Save results in the calibration records.
137. **[V,D, L]** Notes: OPEN LAMP SHUTTER

Date 5/6 Time 21:49 Initial ___

In-band and Out-of-band Measurements for Filter 6 of the Left Mastcam-Z

138. **[T]** ___ Set the monochromator to the filter's peak wavelength given in Table 2 450 nm
139. **[O,T,D]** ___ Load and execute prefix **433TAMBL60**, which helps to find the optimal sub-frame, focus position, and exposure time. The GUI's note field should have "GRATING=G123,WAVELENGTH=[nanometers]".
140. **[L,[redacted],D]** ___ Record the following values,
    - sub-frame window: 912, 528, 32, 208 (preferably same as filter 0)
    - focus position: 726
    - exposure time: @ 450 nm = 1.5, @ 452 = 1.8, @ 448 = 1.6 ms
141. **[L,O]** ___ If necessary, update the scripts **433TAMBL61** and **433TAMBL62** for these sub-frame, focus position, and exposure time values.
142. **[T]** ___ Set up a monochromator scan for the filter's wait-time and **in-band** start, stop and step-size wavelengths given in Table 2.
143. **[O,T,L]** ___ Confirm that the script and monochromator scan have the same start, stop and step-size wavelengths. Set the dwell-time to at least 5 seconds between image sets (or to another appropriate value).
144. **[O,T,D]** ___ Load and execute prefix **433TAMBL61**, which captures 10 frames and 3 bias frames at the 100 mm focal length for each wavelength in the scan. Push continue in the GUI whenever the monochromator steps to the next wavelength position. The last wavelength position is for dark frames. The GUI's note field should have "GRATING=G123,WAVELENGTH=[nanometers]". The estimated duration is given in Table 2.
145. **[T]** ___ Close the lamp shutter. The last block of images in the script is for dark frames.
146. **[D]** ___ Double check that all image names, suffixes, and other parameters are recorded in Image log.
147. **[O,V]** ___ Make sure that the filter **6**'s transmission was measured to no more that 1% on both sides of its transition curve. Notes: GOOD.

 Date 5/6 Time 20:02 Initial ______

OPEN LAMP SHUTTER

______

148. **[T]**______ Set up a monochromator scan for the filter's wait-time and **out-band** start, stop and step-size wavelengths given in Table 2.

149. **[[redacted],T,L]**______ Confirm that the script and monochromator scan have the same start, stop and step-size wavelengths. Set the dwell-time to at least 5 seconds between image sets (or to another appropriate value).

150. **[O,T,D]**______ Load and execute prefix **433TAMBL62**, which captures 3 frames and 1 bias frame at the 100 mm focal length for each wavelength in the scan. Push continue in the GUI whenever the monochromator steps to the next wavelength position. The last wavelength position is for dark frames. The GUI's note field should have "GRATING=G123,WAVELENGTH=[nanometers]". The estimated duration is given in Table 2.

151. **[T]**______ Close the lamp shutter. The last block of images in the script is for dark frames.

152. **[D]**______ Double check that all image names, suffixes, and other parameters are recorded in Image log.

153. **[[redacted],V]** Notes: OPEN LAMP SHUTTER

______

______

GRATING 2/3
CROSSOVER WAVELENGTH = 652 nm (SHOWING 658)

154. **[T]** [illegible] Perform a full radiometer scan between 300 and 1100 nm in 2 nm increments with a 2 second dwell-time at each wavelength. Follow the procedure in "Monochromator_Manual". Be sure to save file as "**mono_scan_g123_300nm_1100nm_2nm_" + "YYMMDD_hhmm**" where the last numbers encode the scan's date and start time. The estimated duration is 15 minutes.

- Scan started at ~~20:22~~ 22:22 and completed at ~~20:51~~ 22:51.

155. **[T]** [illegible] Lights on

Data Validation

156. **[V]** [illegible] Upload data to server.
157. **[V]** [illegible] Run the "Spectral_Throughput_43_Validation" Jupyter notebook on the acquired data. This analysis can take place while the test continues.
    - Create preliminary spectral throughput curves for the filter.
    - Save results in the calibration records.
158. **[V,D, L]** Notes: OK, BUT STEP IN BLUE RESPONSE AT ABOUT 590 nm.

Time Check 1

IF MORE THAN 1.0 HOUR AHEAD OF SCHEDULED END, CONTINUE WITH THE CONTINUOUS TESTS, OTHERWISE SKIP TO THE NEXT TIME CHECK

Scheduled End Time	Current Time	Time Ahead of Scheduled End
-	=	

159. **[D, L]** ____ Record the time in the table above and determine if there is time for more testing.
160. **[D, L]** Notes: BEHIND SCHEDULE
161. **[T]** ____ Lights off

In-band Measurements for Filter 7 of the Left Mastcam-Z

SKIP

162. **[T]** ____ Set the monochromator to the filter's peak wavelength given in Table 2.
163. **[O,T,D]** ____ Load and execute prefix **433TAMBL70**, which helps to find the optimal sub-frame, focus position, and exposure time. The GUI's note field should have "GRATING=G123,WAVELENGTH=[nanometers],WINDOW=1".
164. **[L,O,D]** ____ Record the following values,
    - sub-frame window: ______________________ (preferably same as filter 0)
    - focus position: ____________
    - exposure time: ____________
165. **[L,O]** ____ Update the **433TAMBL71** script for these sub-frame, focus position, and exposure time values. Be sure to add "WINDOW=1" to the note.
166. **[T]** ____ Set up a monochromator scan for the filter's wait-time and **in-band** start, stop and step-size wavelengths given in Table 2.
167. **[O,T,L]** ____ Confirm that the script and monochromator scan have the same start, stop and step-size wavelengths. Set the dwell-time to at least 5 seconds between image sets (or to another appropriate value).
168. **[[redacted],T,D]** ____ Load and execute prefix **433TAMBL71**, which captures 10 frames and 3 bias frames at the 100 mm focal length for each wavelength in the scan. Push continue in the GUI whenever the monochromator steps to the next wavelength position. The last wavelength position is for dark frames. The GUI's note field should have "GRATING=G123,WAVELENGTH=[nanometers], WINDOW=1". The estimated duration is given in Table 2.
169. **[T]** ____ Close the lamp shutter. The last block of images in the script is for dark frames.
170. **[D]** ____ Double check that all image names, suffixes, and other parameters are recorded in Image log.
171. **[O,V]** ____ Make sure that the filter 7's transmission was measured to no more that 1% on both sides of its transition curve. Notes: ______________________________

CHANGE GRATING 2/3 CROSSOVER WAVELENGTH TO 700nm.

172. **[T]** [illegible] Perform a full radiometer scan between 300 and 1100 nm in 2 nm increments with a 2 second dwell-time at each wavelength. Follow the procedure in "Monochromator_Manual". Be sure to save file as "**mono_scan_g123_300nm_1100nm_2nm_**" + "**YYMMDD_hhmm**" where the last numbers encode the scan's date and start time. The estimated duration is 15 minutes.

- Scan started at ~~20:58~~ 22:58 and completed at ~~23:27~~ 23:27.

GO TO ~~[illegible]~~ STEP 22

Data Validation

173. **[V]** ____ Upload data to server.
174. **[V]** ____ Run the "Spectral_Throughput_43_Validation" Jupyter notebook on the acquired data. This analysis can take place while the test continues.
    - Create preliminary spectral throughput curves for the filter.
    - Save results in the calibration records.
175. **[V,D, L]** Notes: ____________________

Shutdown Procedure

Skip Procedure not Completed

176. **[D,T]** ____ Take digital pictures of the test setup.
177. **[D,O]** ____ Review entries in Image Log, GSE command log, and image headers.
178. **[D, L]** ____ Review calibration procedure and ensure that each task is initialed.
179. **[D, L]** Notes: ____

____

____

180. **[V, L]** ____ Before making the decision to break down the test setup, ensure that adequate data were acquired for the test requirements. See "MastcamZCalPlan" for these requirements.
181. **[V]** Notes: ____

____

____

Data Validator (signature) ______________________

Date ______________ Time ______________

182. **[V, L]** ____ Give the go/no-go decision. Have enough data been acquired to fulfill test requirements? See "MastcamZCalPlan" for these requirements.
183. **[D, L]** ____ Update the Log Document.
184. **[L]** Notes: ____

____

____

Calibration Lead (signature) ______________________

Date ______________ Time ______________

185. **[O, L]** ____ Ensure that the camera and GSE are in a safe state.
186. **[O,D]** ____ Review the Image Log with the documentarian. Exchange high-fives.
187. **[O]** Notes: ____
____
____

Camera Operator (signature) ________________________

Date ______________ Time ______________

188. **[T]** ____ If the next test does not require the monochrometer, position it away from the chamber or bench. Otherwise, be sure not to move it. The next test is ___________ _.
189. **[T]** ____ Ensure that all other test equipment is safely put away.
190. **[T]** Notes: ____
____
____

Technician (signature) ____________________________

Date ______________ Time ______________

191. **[D, L]** ____ Double-check this procedure and ensure that the top of each page has valid data, time and initials.
192. **[D]** ____ Photo-scan this document, save it on the cloud, and file the hard-copy in the Log Binder. Upload the digital pictures taken during this test in the appropriate archive on the cloud. The required links are on the Wiki.
193. **[D]** ____ Double-check that every required cell the Image Log is accurately filled. When this is complete, print the Image Log and file it the Log Binder after this document.
194. **[D]** Notes: ____
____
____

Documentarian (signature) __________________________

Date ______________ Time ______________

(V2)

In-band and Out-of-band Measurements for Filter 0 of the Left Mastcam-Z

Radiometer Scan
Start 8:32am
Finish 8:59

22. **[T]** AGH Lights off
23. **[T]** AGH Set the monochromator to the filter's peak wavelength given in Table 2.
24. **[[redacted],T,D]** AGH Load and execute prefix **433TAML00**, which helps to find the optimal sub-frame, focus position, and exposure time. The GUI's note field should have "GRATING=G123,WAVELENGTH=[nanometers]".
25. **[[redacted],T]** AGH Position the monochromator as need to aim the slit near the camera's boresight. Rerun script **433TAMBL00** as needed.
26. **[L,O,D]** AGH Record the following values,
    - sub-frame window: 912 512 32 208
    - focus position: 726
    - exposure time: 0.4msec

can iterate here w/ slight peak shift (±2nm)

→ room dark

27. **[L,O]** AGH Update the **433TAMBL**** scripts for this sub-frame, focus position, and exposure time values.
28. **[T]** AGH Set up a monochromator scan for the filter's wait-time and **in-band** start, stop and step-size wavelengths given in Table 2.
29. **[O,T,L]** AGH Confirm that the script and monochromator scan have the same start, stop and step-size wavelengths. Set the dwell-time to at least 5 seconds between image sets (or to another appropriate value).
30. **[O,T,D]** AGH Load and execute prefix **433TAMBL01**, which captures 10 frames and 3 bias frames at the 100 mm focal length for each wavelength in the scan. Push continue in the GUI whenever the monochromator steps to the next wavelength position. The last wavelength position is for dark frames. The GUI's note field should have "GRATING=G123,WAVELENGTH=[nanometers]". The estimated duration is given in Table 2.

start: 9:09
end: 9:30
(+restart)

31. **[T]** AGH Close the lamp shutter. The last block of images in the script is for dark frames.

552, 554, 556

V2

32. **[D]** A6W Double check that all image names, suffixes, and other parameters are recorded in Image log.
33. **[O,V]** A6W Make sure that the filter **0**'s transmission was measured to no more that 1% on both sides of its transition curve. Notes: ______________________________

34. **[T]** A6W Set up a monochromator scan for the filter's wait-time and **out-band** start, stop and step-size wavelengths given in Table 2.
35. **[O,T,L]** A6W Confirm that the script and monochromator scan have the same start, stop and step-size wavelengths. Set the dwell-time to at least 5 seconds between image sets (or to another appropriate value).
36. **[O,T,D]** A6W Load and execute prefix **433TAMBL02**, which captures 3 frames and 1 bias frame at the 100 mm focal length for each wavelength in the scan. Push continue in the GUI whenever the monochromator steps to the next wavelength position. The last wavelength position is for dark frames. The GUI's note field should have "GRATING=G123,WAVELENGTH=[nanometers]". The estimated duration is given in Table 2.
37. **[T]** A6W Close the lamp shutter. The last block of images in the script is for dark frames.
38. **[D]** A6W Double check that all image names, suffixes, and other parameters are recorded in Image log.
39. **[O,V]** Notes: Redo of 552, 554, 556, to check a spike in data.

Data Validation

40. **[V]** _AC_ Upload data to server.
41. **[V]** _AC_ Run the "Spectral_Throughput_43_Validation" Jupyter notebook on the acquired data. This analysis can take place while the test continues.
    - Create preliminary spectral throughput curves for the filter.
    - Save results in the calibration records.
42. **[V,D, L]** Notes: ____

____

____

In-band and Out-of-band Measurements for Filter 1 of the Left Mastcam-Z

43. **[T]** ____ Set the monochromator to the filter's peak wavelength given in Table 2.
44. **[O,T,D]** ____ Load and execute prefix **433TAMBL10**, which helps to find the optimal sub-frame, focus position, and exposure time. The GUI's note field should have "GRATING=G123,WAVELENGTH=[nanometers]".
45. **[L,O,D]** ____ Record the following values,
    - sub-frame window: Same (preferably same as filter 0)
    - focus position: 726
    - exposure time: 0.6 msec
46. **[L,[redacted]]** ____ If necessary, update the scripts **433TAMBL11** and **433TAMBL12** for these sub-frame, focus position, and exposure time values.
47. **[T]** ____ Set up a monochromator scan for the filter's wait-time and **in-band** start, stop and step-size wavelengths given in Table 2.
48. **[O,T,L]** ____ Confirm that the script and monochromator scan have the same start, stop and step-size wavelengths. Set the dwell-time to at least 5 seconds between image sets (or to another appropriate value).
49. **[O,T,D]** ____ Load and execute prefix **433TAMBL11**, which captures 10 frames and 3 bias frames at the 100 mm focal length for each wavelength in the scan. Push continue in the GUI whenever the monochromator steps to the next wavelength position. The last wavelength position is for dark frames. The GUI's note field should have "GRATING=G123,WAVELENGTH=[nanometers]". The estimated duration is given in Table 2.
50. **[T]** ____ Close the lamp shutter. The last block of images in the script is for dark frames.
51. **[D]** ____ Double check that all image names, suffixes, and other parameters are recorded in Image log.
52. **[O,V]** ____ Make sure that the filter **1**'s transmission was measured to no more that 1% on both sides of its transition curve. Notes: ______________________________

____

____

53. **[T]** A614 Set up a monochromator scan for the filter's wait-time and **out-band** start, stop and step-size wavelengths given in Table 2.
54. **[O,T,L]** A614 Confirm that the script and monochromator scan have the same start, stop and step-size wavelengths. Set the dwell-time to at least 5 seconds between image sets (or to another appropriate value).
55. **[O,T,D]** A614 Load and execute prefix **433TAMBL12**, which captures 3 frames and 1 bias frame at the 100 mm focal length for each wavelength in the scan. Push continue in the GUI whenever the monochromator steps to the next wavelength position. The last wavelength position is for dark frames. The GUI's note field should have "GRATING=G123,WAVELENGTH=[nanometers]". The estimated duration is given in Table 2.
56. **[T]** A614 Close the lamp shutter. The last block of images in the script is for dark frames.
57. **[D]** A614 Double check that all image names, suffixes, and other parameters are recorded in Image log.
58. **[O,V]** Notes: ____

____

____

Data Validation

59. **[V]** AbH Upload data to server.
60. **[V]** AbH Run the "Spectral_Throughput_43_Validation" Jupyter notebook on the acquired data. This analysis can take place while the test continues.
    - Create preliminary spectral throughput curves for the filter.
    - Save results in the calibration records.
61. **[V,D, L]** Notes: ____

____

____

In-band and Out-of-band Measurements for Filter 2 of the Left Mastcam-Z

62. **[T]** AGH Set the monochromator to the filter's peak wavelength given in Table 2.
63. **[[redacted],T,D]** AGH Load and execute prefix **433TAMBL20**, which helps to find the optimal sub-frame, focus position, and exposure time. The GUI's note field should have "GRATING=G123,WAVELENGTH=[nanometers]".
64. **[L,O,D]** AGH Record the following values,
    - sub-frame window: 912 496 32 208 (preferably same as filter 0)
    - focus position: 726 912 480 32 240 ← in out → auto focus
    - exposure time: 0.4 sec
65. **[L,[redacted]]** AGH If necessary, update the scripts **433TAMBL21** and **433TAMBL22** for these sub-frame, focus position, and exposure time values.
66. **[T]** AGH Set up a monochromator scan for the filter's wait-time and **in-band** start, stop and step-size wavelengths given in Table 2.
67. **[[redacted],T,L]** AGH Confirm that the script and monochromator scan have the same start, stop and step-size wavelengths. Set the dwell-time to at least 5 seconds between image sets (or to another appropriate value).
68. **[[redacted],T,D]** AGH Load and execute prefix **433TAMBL21**, which captures 10 frames and 3 bias frames at the 100 mm focal length for each wavelength in the scan. Push continue in the GUI whenever the monochromator steps to the next wavelength position. The last wavelength position is for dark frames. The GUI's note field should have "GRATING=G123,WAVELENGTH=[nanometers]". The estimated duration is given in Table 2.
69. **[T]** AGH Close the lamp shutter. The last block of images in the script is for dark frames.
70. **[D]** AGH Double check that all image names, suffixes, and other parameters are recorded in Image log.
71. **[O,V]** AGH Make sure that the filter **2**'s transmission was measured to no more that 1% on both sides of its transition curve. Notes: ______________________________

____

____

72. **[T]** ____ Set up a monochromator scan for the filter's wait-time and **out-band** start, stop and step-size wavelengths given in Table 2.
73. **[O,T,L]** ____ Confirm that the script and monochromator scan have the same start, stop and step-size wavelengths. Set the dwell-time to at least 5 seconds between image sets (or to another appropriate value).
74. **[O,T,D]** ____ Load and execute prefix **433TAMBL22**, which captures 3 frames and 1 bias frame at the 100 mm focal length for each wavelength in the scan. Push continue in the GUI whenever the monochromator steps to the next wavelength position. The last wavelength position is for dark frames. The GUI's note field should have "GRATING=G123,WAVELENGTH=[nanometers]". The estimated duration is given in Table 2.
75. **[T]** ____ Close the lamp shutter. The last block of images in the script is for dark frames.
76. **[D]** ____ Double check that all image names, suffixes, and other parameters are recorded in Image log.
77. **[O,V]** Notes: ____

____

____

Data Validation

78. **[V]** AGL Upload data to server.
79. **[V]** AGL Run the "Spectral_Throughput_43_Validation" Jupyter notebook on the acquired data. This analysis can take place while the test continues.
    - Create preliminary spectral throughput curves for the filter.
    - Save results in the calibration records.
80. **[V,D, L]** Notes: ____

____

____

In-band and Out-of-band Measurements for Filter 3 of the Left Mastcam-Z

81. **[T]** _____ Set the monochromator to the filter's peak wavelength given in Table 2.
82. **[O,T,D]** _____ Load and execute prefix **433TAMBL30**, which helps to find the optimal sub-frame, focus position, and exposure time. The GUI's note field should have "GRATING=G123,WAVELENGTH=[nanometers]".
83. **[L,[redacted],D]** _____ Record the following values,
    - sub-frame window: 912 496 32 208
    - focus position: 726
    - exposure time: 0.5
84. **[L,[redacted]]** _____ If necessary, update the scripts **433TAMBL31** and **433TAMBL32** for these sub-frame, focus position, and exposure time values.
85. **[T]** _____ Set up a monochromator scan for the filter's wait-time and **in-band** start, stop and step-size wavelengths given in Table 2.
86. **[O,T,L]** _____ Confirm that the script and monochromator scan have the same start, stop and step-size wavelengths. Set the dwell-time to at least 5 seconds between image sets (or to another appropriate value).
87. **[[redacted],T,D]** _____ Load and execute prefix **433TAMBL31**, which captures 10 frames and 3 bias frames at the 100 mm focal length for each wavelength in the scan. Push continue in the GUI whenever the monochromator steps to the next wavelength position. The last wavelength position is for dark frames. The GUI's note field should have "GRATING=G123,WAVELENGTH=[nanometers]". The estimated duration is given in Table 2.
88. **[T]** _____ Close the lamp shutter. The last block of images in the script is for dark frames.
89. **[D]** _____ Double check that all image names, suffixes, and other parameters are recorded in Image log.
90. **[O,V]** _____ Make sure that the filter **3**'s transmission was measured to no more that 1% on both sides of its transition curve. Notes: ______________________________

V2

____

____

91. **[T]** ____ Set up a monochromator scan for the filter's wait-time and **out-band** start, stop and step-size wavelengths given in Table 2.
92. **[O,T,L]** ____ Confirm that the script and monochromator scan have the same start, stop and step-size wavelengths. Set the dwell-time to at least 5 seconds between image sets (or to another appropriate value).
93. **[O,T,D]** ____ Load and execute prefix **433TAMBL32**, which captures 3 frames and 1 bias frame at the 100 mm focal length for each wavelength in the scan. Push continue in the GUI whenever the monochromator steps to the next wavelength position. The last wavelength position is for dark frames. The GUI's note field should have "GRATING=G123,WAVELENGTH=[nanometers]". The estimated duration is given in Table 2.
94. **[T]** ____ Close the lamp shutter. The last block of images in the script is for dark frames.
95. **[D]** ____ Double check that all image names, suffixes, and other parameters are recorded in Image log.
96. **[O,V]** Notes: ____

____

____

In Band Only

97. **[T]** ____ Perform a full radiometer scan between 300 and 1100 nm in 2 nm increments with a 2 second dwell-time at each wavelength. Follow the procedure in "Monochromator_Manual". Be sure to save file as **"mono_scan_g123_300nm_1100nm_2nm_" + "YYMMDD_hhmm"** where the last numbers encode the scan's date and start time. The estimated duration is 20 minutes.
    - Scan started at ______________ and completed at _______________.

Data Validation

98. **[V]** _MW_ Upload data to server.
99. **[V]** _MW_ Run the "Spectral_Throughput_43_Validation" Jupyter notebook on the acquired data. This analysis can take place while the test continues.
    - Create preliminary spectral throughput curves for the filter.
    - Save results in the calibration records.

100. **[V,D, L]** Notes: ____

____

____

In-band and Out-of-band Measurements for Filter 4 of the Left Mastcam-Z

101. **[T]** A6H Set the monochromator to the filter's peak wavelength given in Table 2.
102. **[O,T,D]** A6H Load and execute prefix **433TAMBL40**, which helps to find the optimal sub-frame, focus position, and exposure time. The GUI's note field should have "GRATING=G123,WAVELENGTH=[nanometers]".
103. **[L,O,D]** A6H Record the following values,
    - sub-frame window: 912 496 32 20 (preferably same as filter 0)
    - focus position: 726 Rerun at 6km
    - exposure time: ~~0.8 msec~~ 0.4
104. **[L,O]** A6H If necessary, update the scripts **433TAMBL41** and **433TAMBL42** for these sub-frame, focus position, and exposure time values.
105. **[T]** A6H Set up a monochromator scan for the filter's wait-time and **in-band** start, stop and step-size wavelengths given in Table 2.
106. **[O,T,L]** A6H Confirm that the script and monochromator scan have the same start, stop and step-size wavelengths. Set the dwell-time to at least 5 seconds between image sets (or to another appropriate value).
107. **[O,T,D]** A6H Load and execute prefix **433TAMBL41**, which captures 10 frames and 3 bias frames at the 100 mm focal length for each wavelength in the scan. Push continue in the GUI whenever the monochromator steps to the next wavelength position. The last wavelength position is for dark frames. The GUI's note field should have "GRATING=G123,WAVELENGTH=[nanometers]". The estimated duration is given in Table 2.
108. **[T]** A6H Close the lamp shutter. The last block of images in the script is for dark frames.
109. **[D]** A6H Double check that all image names, suffixes, and other parameters are recorded in Image log.
110. **[O,V]** A6H Make sure that the filter **4**'s transmission was measured to no more that 1% on both sides of its transition curve. Notes: ______________________________

____

____

111. **[T]** ____ Set up a monochromator scan for the filter's wait-time and **out-band** start, stop and step-size wavelengths given in Table 2.
112. **[O,T,L]** ____ Confirm that the script and monochromator scan have the same start, stop and step-size wavelengths. Set the dwell-time to at least 5 seconds between image sets (or to another appropriate value).
113. **[O,T,D]** ____ Load and execute prefix **433TAMBL42**, which captures 3 frames and 1 bias frame at the 100 mm focal length for each wavelength in the scan. Push continue in the GUI whenever the monochromator steps to the next wavelength position. The last wavelength position is for dark frames. The GUI's note field should have "GRATING=G123,WAVELENGTH=[nanometers]". The estimated duration is given in Table 2.
114. **[T]** ____ Close the lamp shutter. The last block of images in the script is for dark frames.
115. **[D]** ____ Double check that all image names, suffixes, and other parameters are recorded in Image log.
116. **[O,V]** Notes: ____

____

____

In-Band Only

Data Validation

117. **[V]** AGH Upload data to server.
118. **[V]** AGH Run the "Spectral_Throughput_43_Validation" Jupyter notebook on the acquired data. This analysis can take place while the test continues.
    - Create preliminary spectral throughput curves for the filter.
    - Save results in the calibration records.
119. **[V,D, L]** Notes: ____

____

____

In-band and Out-of-band Measurements for Filter 5 of the Left Mastcam-Z

120. **[T]** ____ Set the monochromator to the filter's peak wavelength given in Table 2.
121. **[O,T,D]** ____ Load and execute prefix **433TAMBL50**, which helps to find the optimal sub-frame, focus position, and exposure time. The GUI's note field should have "GRATING=G123,WAVELENGTH=[nanometers]".
122. **[L,O,D]** ____ Record the following values,
    - sub-frame window: 912 496 32 208
    - focus position: 726
    - exposure time: 0.7
123. **[L,O]** ____ If necessary, update the scripts **433TAMBL51** and **433TAMBL52** for these sub-frame, focus position, and exposure time values.
124. **[T]** ____ Set up a monochromator scan for the filter's wait-time and **in-band** start, stop and step-size wavelengths given in Table 2.
125. **[O,T,L]** ____ Confirm that the script and monochromator scan have the same start, stop and step-size wavelengths. Set the dwell-time to at least 5 seconds between image sets (or to another appropriate value).
126. **[O,T,D]** ____ Load and execute prefix **433TAMBL51**, which captures 10 frames and 3 bias frames at the 100 mm focal length for each wavelength in the scan. Push continue in the GUI whenever the monochromator steps to the next wavelength position. The last wavelength position is for dark frames. The GUI's note field should have "GRATING=G123,WAVELENGTH=[nanometers]". The estimated duration is given in Table 2.
127. **[T]** ____ Close the lamp shutter. The last block of images in the script is for dark frames.
128. **[D]** ____ Double check that all image names, suffixes, and other parameters are recorded in Image log.
129. **[O,V]** ____ Make sure that the filter **5**'s transmission was measured to no more that 1% on both sides of its transition curve. Notes: ______________________________

V2

Date ______ Time ______ Initial ______

____

____

130. **[T]** ____ Set up a monochromator scan for the filter's wait-time and **out-band** start, stop and step-size wavelengths given in Table 2.

131. **[O,T,L]** ____ Confirm that the script and monochromator scan have the same start, stop and step-size wavelengths. Set the dwell-time to at least 5 seconds between image sets (or to another appropriate value).

132. **[O,T,D]** ____ Load and execute prefix **433TAMBL52**, which captures 3 frames and 1 bias frame at the 100 mm focal length for each wavelength in the scan. Push continue in the GUI whenever the monochromator steps to the next wavelength position. The last wavelength position is for dark frames. The GUI's note field should have "GRATING=G123,WAVELENGTH=[nanometers]". The estimated duration is given in Table 2.

133. **[D]** ____ Double check that all image names, suffixes, and other parameters are recorded in Image log.

134. **[O,V]** Notes: ____

____

____

In-Band Only

Data Validation

135. **[V]** ____ Upload data to server.
136. **[V]** ____ Run the "Spectral_Throughput_43_Validation" Jupyter notebook on the acquired data. This analysis can take place while the test continues.
    - Create preliminary spectral throughput curves for the filter.
    - Save results in the calibration records.
137. **[V,D, L]** Notes: ____

____

____

In-band and Out-of-band Measurements for Filter 6 of the Left Mastcam-Z

138. **[T]** A6K Set the monochromator to the filter's peak wavelength given in Table 2.
139. **[O,T,D]** A6K Load and execute prefix **433TAMBL60**, which helps to find the optimal sub-frame, focus position, and exposure time. The GUI's note field should have "GRATING=G123,WAVELENGTH=[nanometers]".
140. **[L,O,D]** A6K Record the following values,
    - sub-frame window: 912496 32 208 (preferably same as filter 0)
    - focus position: 726
    - exposure time: ~~1.3~~ 1.6
141. **[L,[redacted]]** A6K If necessary, update the scripts **433TAMBL61** and **433TAMBL62** for these sub-frame, focus position, and exposure time values.
142. **[T]** A6K Set up a monochromator scan for the filter's wait-time and **in-band** start, stop and step-size wavelengths given in Table 2.
143. **[O,T,L]** A6K Confirm that the script and monochromator scan have the same start, stop and step-size wavelengths. Set the dwell-time to at least 5 seconds between image sets (or to another appropriate value).
144. **[O,T,D]** A6K Load and execute prefix **433TAMBL61**, which captures 10 frames and 3 bias frames at the 100 mm focal length for each wavelength in the scan. Push continue in the GUI whenever the monochromator steps to the next wavelength position. The last wavelength position is for dark frames. The GUI's note field should have "GRATING=G123,WAVELENGTH=[nanometers]". The estimated duration is given in Table 2.
145. **[T]** A6K Close the lamp shutter. The last block of images in the script is for dark frames.
146. **[D]** A6K Double check that all image names, suffixes, and other parameters are recorded in Image log.
147. **[O,V]** A6K Make sure that the filter **6**'s transmission was measured to no more that 1% on both sides of its transition curve. Notes: ______________________

Included one room dark

V2

____

____

148. **[T]** ____ Set up a monochromator scan for the filter's wait-time and **out-band** start, stop and step-size wavelengths given in Table 2.

149. **[O,T,L]** ____ Confirm that the script and monochromator scan have the same start, stop and step-size wavelengths. Set the dwell-time to at least 5 seconds between image sets (or to another appropriate value).

150. **[O,T,D]** ____ Load and execute prefix **433TAMBL62**, which captures 3 frames and 1 bias frame at the 100 mm focal length for each wavelength in the scan. Push continue in the GUI whenever the monochromator steps to the next wavelength position. The last wavelength position is for dark frames. The GUI's note field should have "GRATING=G123,WAVELENGTH=[nanometers]". The estimated duration is given in Table 2.

151. **[T]** ____ Close the lamp shutter. The last block of images in the script is for dark frames.

152. **[D]** ____ Double check that all image names, suffixes, and other parameters are recorded in Image log.

153. **[O,V]** Notes: ____

____

____

In-Band Only

154. **[T]** AGH Perform a full radiometer scan between 300 and 1100 nm in 2 nm increments with a 2 second dwell-time at each wavelength. Follow the procedure in "Monochromator_Manual". Be sure to save file as "**mono_scan_g123_300nm_1100nm_2nm_**" + "**YYMMDD_hhmm**" where the last numbers encode the scan's date and start time. The estimated duration is 15 minutes.

- Scan started at 12:21pm and completed at ____________.

155. **[T]** AGH Lights on

Data Validation

156. **[V]** ____ Upload data to server.
157. **[V]** ____ Run the "Spectral_Throughput_43_Validation" Jupyter notebook on the acquired data. This analysis can take place while the test continues.
    - Create preliminary spectral throughput curves for the filter.
    - Save results in the calibration records.
158. **[V,D, L]** Notes: ____

____

____

Time Check 1

IF MORE THAN 1.0 HOUR AHEAD OF SCHEDULED END, CONTINUE WITH THE CONTINUOUS TESTS, OTHERWISE SKIP TO THE NEXT TIME CHECK

Scheduled End Time	Current Time	Time Ahead of Scheduled End
-	=	

159. **[D, L]** ____ Record the time in the table above and determine if there is time for more testing.
160. **[D, L]** Notes:

____

____

____

161. **[T]** ____ Lights off

In-band Measurements for Filter 7 of the Left Mastcam-Z

162. **[T]** ____ Set the monochromator to the filter's peak wavelength given in Table 2.
163. **[O,T,D]** ____ Load and execute prefix **433TAMBL70**, which helps to find the optimal sub-frame, focus position, and exposure time. The GUI's note field should have "GRATING=G123,WAVELENGTH=[nanometers],WINDOW=1".
164. **[L,O,D]** ____ Record the following values,
    - sub-frame window: ________________________ (preferably same as filter 0)
    - focus position: ____________
    - exposure time: ____________
165. **[L,O]** ____ Update the **433TAMBL71** script for these sub-frame, focus position, and exposure time values. Be sure to add "WINDOW=1" to the note.
166. **[T]** ____ Set up a monochromator scan for the filter's wait-time and **in-band** start, stop and step-size wavelengths given in Table 2.
167. **[O,T,L]** ____ Confirm that the script and monochromator scan have the same start, stop and step-size wavelengths. Set the dwell-time to at least 5 seconds between image sets (or to another appropriate value).
168. **[O,T,D]** ____ Load and execute prefix **433TAMBL71**, which captures 10 frames and 3 bias frames at the 100 mm focal length for each wavelength in the scan. Push continue in the GUI whenever the monochromator steps to the next wavelength position. The last wavelength position is for dark frames. The GUI's note field should have "GRATING=G123,WAVELENGTH=[nanometers], WINDOW=1". The estimated duration is given in Table 2.
169. **[T]** ____ Close the lamp shutter. The last block of images in the script is for dark frames.
170. **[D]** ____ Double check that all image names, suffixes, and other parameters are recorded in Image log.
171. **[[redacted],V]** ____ Make sure that the filter **7**'s transmission was measured to no more that 1% on both sides of its transition curve. Notes: ______________________________

____

____

172. **[T]** ____ Perform a full radiometer scan between 300 and 1100 nm in 2 nm increments with a 2 second dwell-time at each wavelength. Follow the procedure in "Monochromator_Manual". Be sure to save file as "**mono_scan_g123_300nm_1100nm_2nm_" + "YYMMDD_hhmm**" where the last numbers encode the scan's date and start time. The estimated duration is 15 minutes.

- Scan started at ______________ and completed at ______________.

Data Validation

173. **[V]** ____ Upload data to server.

174. **[V]** ____ Run the "Spectral_Throughput_43_Validation" Jupyter notebook on the acquired data. This analysis can take place while the test continues.

- Create preliminary spectral throughput curves for the filter.
- Save results in the calibration records.

175. **[V,D, L]** Notes: ____

____

____

Shutdown Procedure

176. **[D,T]** ____ Take digital pictures of the test setup.
177. **[D,O]** ____ Review entries in Image Log, GSE command log, and image headers.
178. **[D, L]** ____ Review calibration procedure and ensure that each task is initialed.
179. **[D, L]** Notes: ____

____

____

180. **[V, L]** ____ Before making the decision to break down the test setup, ensure that adequate data were acquired for the test requirements. See "MastcamZCalPlan" for these requirements.
181. **[V]** Notes: ____

____

____

Data Validator (signature) ____________________

Date ______________ Time ______________

182. **[V, L]** ____ Give the go/no-go decision. Have enough data been acquired to fulfill test requirements? See "MastcamZCalPlan" for these requirements.
183. **[D, L]** ____ Update the Log Document.
184. **[L]** Notes: ____

____

____

Calibration Lead (signature) ____________________

Date ______________ Time ______________

185. **[O, L]** ____ Ensure that the camera and GSE are in a safe state.
186. **[O,D]** ____ Review the Image Log with the documentarian. Exchange high-fives.
187. **[O]** Notes: ____

____

____

Camera Operator (signature) ______________________

Date ______________ Time ______________

188. **[T]** ____ If the next test does not require the monochrometer, position it away from the chamber or bench. Otherwise, be sure not to move it. The next test is ___________.
189. **[T]** ____ Ensure that all other test equipment is safely put away.
190. **[T]** Notes: ____

____

____

Technician (signature) ______________________

Date ______________ Time ______________

191. **[D, L]** ____ Double-check this procedure and ensure that the top of each page has valid data, time and initials.
192. **[D]** ____ Photo-scan this document, save it on the cloud, and file the hard-copy in the Log Binder. Upload the digital pictures taken during this test in the appropriate archive on the cloud. The required links are on the Wiki.
193. **[D]** ____ Double-check that every required cell the Image Log is accurately filled. When this is complete, print the Image Log and file it the Log Binder after this document.
194. **[D]** Notes: ____

____

____

Documentarian (signature) ______________________

Date ______________ Time ______________
