# Supplementary material for: Pre-Flight Calibration of the Mars 2020 Rover Mastcam Zoom (Mastcam-Z) Multispectral, Stereoscopic Imager
Source: Space Sci Rev. 2021 Feb 18;217(2):29. doi: 10.1007/s11214-021-00795-x (PMC7892537; doi:10.1007/s11214-021-00795-x)
Supplement: Supplementary file 1 — (ZIP 98.6 MB) [file 11214_2021_795_MOESM1_ESM.zip › CalPro_441_Dark_Current_v2_01.pdf]

Dark Current Procedure for The Right Mastcam-Z Testing at ASU (Pro. 4.4.1)

[Procedure version 2.01, prepared by the Mastcam-Z calibration team at Cornell University]

These measurements are performed on the camera and at the temperature designated below as specified in the Mastcam-Z Calibration Plan,

Unit Under Test:

Left FM X **Right FM** X **EQM**_____ **Other**_____

These measurements are performed at temperature:

-35°C_____ **-10°C** X **+5°C**_____ **Ambient**_____ **Other** TEMP

These measurements are performed at,

MSSS X **ASU** ~~X~~ **Other**_____

Date 4-26-2019 **Start Time** 7:00 pm **End Time** 7:30 pm

Estimated Duration 2 hours

Scheduled Start Time NA **Sch. End Time** NA

Calibration Lead [L] ~~Christian Tate~~ Jim Bell **Documentarian [D]** Christian Tate

Camera Operator [O] Tex and Jason **Technician [T]** Andrew Winhold

Data Validator [V] Paul Corlies **Other**_____

Table of Contents

DARK CURRENT PROCEDURE FOR THE RIGHT MASTCAM-Z TESTING AT ASU (PRO. 4.4.1) **1**

TEST DESCRIPTION 3

SOFTWARE PREPARATION 3

HARDWARE INSTALLATION 4

Figure 1. ASU Floor Plan for Geometric Testing in the TVAC Chamber. The MSSS Floor Plan allows for similar target and source placements relative to the chamber window. 4

Table 1. Dark Exposure times in ***milliseconds*** *for part "01"* 5

Table 2. Dark Exposure times in ***milliseconds*** *for part "02"* 5

Table 3. Dark Exposure times in ***milliseconds*** *for part "04"* 5

DARK CURRENT TEST FOR THE RIGHT MASTCAM-Z AT TEMPERATURE 1 6

DARK CURRENT TEST FOR THE LEFT MASTCAM-Z AT TEMPERATURE 1 6

DARK CURRENT TEST FOR THE RIGHT MASTCAM-Z AT TEMPERATURE 2 7

DARK CURRENT TEST FOR THE LEFT MASTCAM-Z AT TEMPERATURE 2 7

DARK CURRENT TEST FOR THE RIGHT MASTCAM-Z AT TEMPERATURE 3 8

DARK CURRENT TEST FOR THE LEFT MASTCAM-Z AT TEMPERATURE 3 8

SHUTDOWN PROCEDURE **9**

Test Description

Excerpt from the Calibration Plan 4.4:

> The objective of the dark current testing is to determine the dark current as a function of temperature as well as acquire the dark current data needed for all ambient and thermal testing. This data will be acquired during one of the thermal cycles of the chamber during radiometric calibrations. Tests will consist of taking a series of exposures of increasing durations (up to five minutes in length) at 10 temperatures while the chamber warms with the solar filters in place and chamber window blocked. These exposures will also be used to determine the bad pixel mask (either dead or hot pixels) for each camera.

Software Preparation

The software and files required for this test are prepared well in advance of test day. This checklist ensures that the following are present, debugged, and executable: (1) all fast-look scripts, (2) automated header generation of all relevant camera parameters, target positioning, and metadata, (3) all camera scripts that command the camera unit, and (4) the directories/file-paths pointing to the data repositories of this specific test.

1. **[D]** Look up the daily calibration schedule and record the scheduled start and end time of this test on the cover page of this document. Also fill out and double-check the other information on the cover page. CT
2. **[D]** Ensure that all supplemental manuals are on hand. These are, CT
    - Validator_Manual, Documentarian_Manual,
    - MastcamZCalPlan
3. **[D]** Ensure that the Image Log is present and ready to use. CT
4. **[V]** Check that all Calgorithms (cal) fast-look and validation scripts are present, up-to-date, and ready to analyze test output. CT
5. ~~**[V]** Create and go to test directory **"441TEMP"**~~ CT
6. **[O]** Check that all camera scripts required for this test are present, up-to-date and ready to command the ground support equipment (GSE). These are, ST
    - **441TEMPR00 - 441TEMPR04**
    - **441TEMPL00 - 441TEMPL04**

7. **[O,V,D, L]** Notes:

___

___

___

Date 5-1 Time 8:50 Initial CT

Hardware Installation

This procedure is for the ambient TVAC chamber testing at MSSS. Figure 1 shows the nominal layout of the TVAC chamber, workspace, Mastcam-Zs, ground support equipment (GSE), targets, sources, and other equipment necessary for this test if it happens at ASU. Although MSSS' cleanroom is different than ASU's, the placement of the targets and sources relative to the chamber window is similar.

Figure 1. ASU Floor Plan for Geometric Testing in the TVAC Chamber. The MSSS Floor Plan allows for similar target and source placements relative to the chamber window.

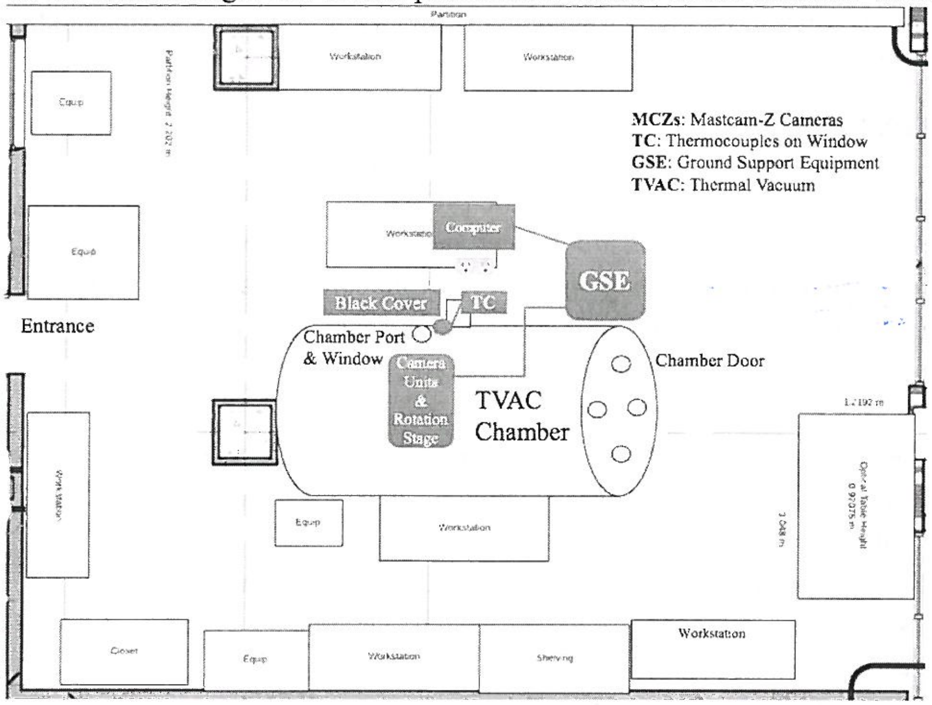

8. **[T,O, L]** CT Ensure that all personnel in the cleanroom are following the cleanroom practices for electrostatic discharge, proper clothing and other safety concerns.
9. **[T]** N/A Double check that nitrogen is flowing over the Mastcam-Zs or the window port.
10. **[O,T]** N/A If not already done, mate the Right and Left Mastcam-Zs into the GSE. Follow the procedure in "MastcamZ_GSE_Manual".
11. **[T]** CT Verify that the thermocouples are turned on and properly reading out.
12. **[O,T]** CT Ensure that the camera unit and GSE wires are secure, kink-free, and do not present tripping hazards when the lights are turned off.
13. **[O,D]** CT Check the camera temperature and ensure nominal operation.

Date 5-2 Time 8:51 Initial CT

14. **[D]** CX Record the following environmental information: Not recorded

- Cleanroom temperature [illegible] pressure _______ humidity _______

15. **[O,D, L]** Notes:

Environmentals not recorded CT

Table 1. Dark Exposure times in **milliseconds** for part **"01"**

Time0	Time1	Time2	Time3
0	1000	10,000	20,000

Table 2. Dark Exposure times in **milliseconds** for part **"02"**

Time0
100,000

Table 3. Dark Exposure times in **milliseconds** for part **"04"**

Time0	Time1
0	1000

Mike C. supplied an EST-safe black plastic bag to rubber band in-front of the chamber window.

Date 4-26 Time 7:003 Initial CT

Dark Current Test for the Right Mastcam-Z at Temperature 1

16. **[D]** Record climate information:
    - TVAC temperature ~-10 pressure 1.5 e-7 torr
    - Camera CCD temperature -5°C other temperatures -10°C stage
17. **[O]** Load and execute camera script **441TEMPR03**, which captures 5 dark frames through filter 7 at the exposure times 0.0, ~~1.0,~~ and ~~2.0~~ seconds. The estimated duration is 1 minute. 0, 10, 20, 100 s
18. **[D]** Record climate information:
    - TVAC temperature ______ Same
    - Camera CCD temperature ______ other temperatures ______

Dark Current Test for the Left Mastcam-Z at Temperature 1

19. **[D]** Record climate information:
    - TVAC temperature ~-10 pressure 1.5e-7 torr
    - Camera CCD temperature -5 other temperatures -10°C stage
20. **[O]** Load and execute camera script **441TEMPR03,** (L) which captures 5 dark frames through filter 7 at the exposure times 0.0, 1.0, and 2.0 seconds. The estimated duration is 1 minute. 0, 10, 20, 100 s
21. **[D]** Record climate information:
    - TVAC temperature ______ Same
    - Camera CCD temperature ______ other temperatures ______

Test terminated after the first temperature measurment for/on on both FMs. We plan to take the next dark current measurement at the end of this temperature's testing (i.e. after the radiametric and geometric work). CT

The next dark current measurements might be at -10°C if Mike C. descides to change it.

Date 4/28 Time 8:05 Initial JFB

Dark Current Test for the Right Mastcam-Z at Temperature 2

22. **[D]** Record climate information:
    - TVAC temperature ~-10 pressure 1.5 e-7
    - Camera CCD temperature -9.6°C other temperatures ______
23. **[O]** Load and execute camera script **441TEMPR03**, which captures 5 dark frames through filter 7 at the exposure times ~~0.0, 1.0, and 2.0 seconds~~ 0, 10, 20, 100 s. The estimated duration is 1 minute.
    INCORRECT - Christian: fix ✓CG
24. **[D]** Record climate information:
    - TVAC temperature ______
    - Camera CCD temperature -5.8°C other temperatures ______

START AT 8:07 AM. Sphere exit port covered

Dark Current Test for the Left Mastcam-Z at Temperature 2

25. **[D]** Record climate information:
    - TVAC temperature ~-10 pressure 1.5 e-7
    - Camera CCD temperature -11.3°C other temperatures ______
26. **[O]** Load and execute camera script **441TEMPR03**, which captures 5 dark frames through filter 7 at the exposure times ~~0.0, 1.0, and 2.0 seconds~~ 0, 10, 20, 100 s. The estimated duration is 1 minute.
    INCORRECT: Christian: fix ✓CG
27. **[D]** Record climate information:
    - TVAC temperature ______
    - Camera CCD temperature -7.2°C other temperatures ______

START AT 8:10 AM. Sphere exit port covered.

* Procedure descriptions incorrect.

Date 5-1 Time 8:45 Initial cf

Skip

Dark Current Test for the Right Mastcam-Z at Temperature 2

28. **[D]** Record climate information:
    - TVAC temperature _______ pressure _______
    - Camera CCD temperature _______ other temperatures ______________
29. **[O]** Load and execute camera script **441TEMPR03**, which captures 5 dark frames through filter 7 at the exposure times 0.0, 1.0, and 2.0 seconds. The estimated duration is 1 minute.
30. **[D]** Record climate information:
    - TVAC temperature _______
    - Camera CCD temperature _______ other temperatures ______________

Dark Current Test for the Left Mastcam-Z at Temperature 2

31. **[D]** Record climate information:
    - TVAC temperature _______ pressure _______
    - Camera CCD temperature _______ other temperatures ______________
32. **[O]** Load and execute camera script **441TEMPR03,** which captures 5 dark frames through filter 7 at the exposure times 0.0, 1.0, and 2.0 seconds. The estimated duration is 1 minute.
33. **[D]** Record climate information:
    - TVAC temperature _______
    - Camera CCD temperature _______ other temperatures ______________

Date 5-1 Time 8:57 Initial CT

Shutdown Procedure

1. **[D,T]** CT Take digital pictures of this page and the test setup.
2. **[D,O]** CT Review entries in Image Log, GSE command log, and image headers.
3. **[D, L]** CT Review calibration procedure and ensure that each task is initialed.
4. **[D, L]** Notes: __________

5. **[V, L]** CT Before making the decision to break down the test setup, ensure that adequate data were acquired for the test requirements. See "MastcamZCalPlan" for these requirements.
6. **[V]** Notes: The results are what we expect

Data Validator (signature) __________

Date __________ Time __________

7. **[V, L]** CT Give the go/no-go decision. Have enough data been acquired to fulfill test requirements? See "MastcamZCalPlan" for these requirements.
8. **[D, L]** CT Update the Log Document.
9. **[L]** Notes: __________

Calibration Lead (signature) Christian Tate

Date 5-1-19 Time 8:57 am

10. [O, L] ____ Ensure that the camera and GSE are in a safe state.
11. [O,D] ____ Review the Image Log with the documentarian. Exchange high-fives.
12. [O] Notes: ____

Camera Operator (signature) ________________________

Date ______________ Time ______________

13. [T] [illegible] If the next test does not require the target, position it away from the chamber or bench. Otherwise, be sure not to move it. The next test is MTF.
14. [T] ____ Ensure that all other test equipment is safely put away.
15. [T] Notes: Removed the black bag

Technician (signature) ________________________

Date ______________ Time ______________

16. [D, L] ____ Double-check this procedure and ensure that the top of each page has valid data, time and initials.
17. [D] ____ Photo-scan this document, save it on the cloud, and file the hard-copy in the Log Binder. Upload the digital pictures taken during this test in the appropriate archive on the cloud. The required links are on the Wiki.
18. [D] ____ Double-check that every required cell the Image Log is accurately filled. When this is complete, print the Image Log and file it the Log Binder after this document.
19. [D] Notes: ____

Documentarian (signature) ________________________

Date ______________ Time ______________

MTF Calibration Procedure for Mastcam-Z TVAC Testing (Pro. 4.7.2)

[Procedure version 2.01, prepared by the Mastcam-Z calibration team at Cornell University.]

These measurements are performed on the camera and at the temperature designated below as specified in the Calibration Plan (Document #),

Unit Under Test:

R FM __X__ L FM __X__ EQM_____ Other__________________

Test Performed at Temperature:

-35°C_______ - 10°C____X_____ +5°C______ Ambient _______ Other _______

These measurements are performed at,

MSSS____X___ ASU________ Other_________

Date __________________ Start Time ______________ End Time _____________

Estimated Duration ___2.5 hours___

Scheduled Start Time ______________ Sch. End Time__________

**Calibration Lead [L]**	________________	**Documentarian [D]**	________________
**Camera Operator [O]**	________________	**Technician [T]**	________________
**Data Validator [V]**	________________	**Metrologist [M]**	________________
**Other**	________________		

Table of Contents

MTF CALIBRATION PROCEDURE FOR MASTCAM-Z TVAC TESTING (PRO. 4.7.2) **1**

DOCUMENT APPROVAL 2

TEST DESCRIPTION 3

SOFTWARE PREPARATION 4

Table 1. File naming convention for the camera script prefixes and frame filenames: "AAABBBBCDD" 4

HARDWARE INSTALLATION 5

Table 2. The nominal target placement scenes for the geometric testing 7

SCENE 1 FOR THE RIGHT MASTCAM-Z 8

SCENE 1 FOR THE LEFT MASTCAM-Z 9

SHUTDOWN PROCEDURE **10**

Document Approval

____

Approved by James Bell Date
Mastcam-Z PI
Arizona State University

____

Approved by Alexander Hayes Date
Mastcam-Z Calibration Working Group
Lead, Cornell University

____

Approved by Justin Maki Date
Mastcam-Z Deputy PI and Investigation
Scientist, Jet Propulsion Laboratory

____

Approved by Christian Tate Date
Procedure Author
Cornell University

____

Approved by: Date

Test Description

Excerpt from the Calibration Plan 4.7

The objective of this test is to image well-characterized bar targets at multiple focus and zoom positions in order to characterize the Modulation Transfer Function (MTF) and depth of field of each camera. Targets should be imaged at ~50% full well using the Bayer RGB (priority 1), 805 nm, (priority 2), and remaining non-solar filters (priority 3). Obtain a minimum of 3 images of each target per filter, focus, and zoom position. Multiple images are needed to reduce errors in determining target locations in the image plane.

MTF is an effective means of specifying the resolution of an optical system. Resolution is defined as the minimum feature size of an object that can be distinguished by an imaging system. The Point Spread Function (PSF) is the inverse Fourier Transform of the MTF—the PSF describes optical performance in the spatial domain while the MTF expresses optical performance in the frequency domain. Images of the bar targets and knife edge or point source targets at various zoom and focus positions will be used to determine PSF, depth of field, and MTF. The bar target shall consist of a chart containing horizontal, vertical, and diagonal lines and bars of varying thicknesses as well as circular dots of various sub- and super-pixel sizes.

In addition to determining the optical performance of the optomechanical assemblies, the images collected during MTF/PSF calibration will also be used to determine the numerical value and repeatability of the stepper motor counts for the Hall Effect sensors used to measure the position of the focus group and two moving zoom groups in the optical zoom assemblies. This will determine the relationship between stepper motor count for each focus/zoom group, working distance, and pixel scale. Owing to thickness variations between spectral filters, focus shifts may occur and images would ideally be obtained using all non-solar filters.

Note that the Mastcam-Z instrument has an onboard focus merge algorithm that selects and merges the best-focus portions of a scene using a focus stack (or z-stack) of multiple images (up to 16) at varying focal positions. While usually only the best-focus or merged product is saved to file, the raw frames for the focus stack can also be saved to file without appreciably increasing the observation or product generation time. For tests where images at multiple focus positions are desired, such as during MTF/PSF Calibrations, the entire focus stack of images will be written to file. Since images at multiple focus positions are acquired irrespective of whether or not they are written to memory, saving multiple focus positions per zoom setting does not require additional test time (outside of the time required to write the files to disk in the GSE). Multiple focus position images shall be saved during both Stand-Alone and ATLO MTF/PSF testing.

Software Preparation

The software and files required for this test are prepared well in advance of test day. This checklist ensures that the following are present, debugged, and executable: (1) all fast look scripts, (2) automated header generation of all relevant camera parameters, target positioning, and metadata, (3) all camera scripts that command the camera unit, and (4) the directories/file-paths pointing to the data repositories of this specific test.

Table 1. File naming convention for the camera script prefixes and frame filenames: "AAABBBBCDD"

Code	Name	Example	Value(s)
"AAA"	Calibration Plan Section	"411" = Cal. Plan 4.1.1 chapter 4, section 1, subsection 1	473, 492
"BBBB"	Location of test or ASU TVAC temperature	"MSSS" = test at MSSS, "ATLO" = test at JPL ATLO, "TN10" = ASU TVAC -10C, …	TN10
"C"	Camera unit under test	"L" = Left Mastcam-Z, "R" = Right Mastcam-Z, "E" =EQM, "C" =COTS	R/L
"DD"	Part of test	"00" = test set up, "01" = first radiance level …	00-12

1. **[D]** ____ Look up the daily calibration schedule and record the scheduled start and end time of this test on the cover page of this document. Also, fill out and double-check the other information on the cover page.
2. **[D]** ____ Ensure that all supplemental manuals are on hand. These are,
   - Validator_Manual, Documentarian_Manual,
   - MastcamZCalPlan
3. **[D]** ____ Ensure that the Image Log is present and ready to use. Find and open the Google Sheets file "Image_Log_46". There is a link on the Wiki.
4. **[V]** ____ Check that all *Calgorithms* fast-look and validation scripts are present, up-to-date, and ready to analyze test output. Find and open the "Geometric_Calibration_46_Validation" Jupyter notebook. There is a link on the Wiki.
5. **[O]** ____ Check that all camera scripts required for this test are present, up-to-date and ready to command the ground support equipment (GSE). These are,

- **473TN10R00 - 473TN10R06, 492TN10R01 - 492TN10R12**
- **473TN10L00 - 473TN10L06, 492TN10L01 - 492TN10L12**

6. **[O,V,D, L]** Notes:

____

____

____

Hardware Installation

This procedure is for the ambient TVAC chamber testing at MSSS. Figure 1 shows the nominal layout of the TVAC chamber, workspace, Mastcam-Zs, ground support equipment (GSE), targets, sources, and other equipment necessary for this test if it happens at ASU. Although MSSS' cleanroom is different than ASU's, the placement of the targets and sources relative to the chamber window is similar.

Figure 1. ASU Floor Plan for Geometric Testing in the TVAC Chamber. The MSSS Floor Plan allows for similar target and source placements relative to the chamber window.

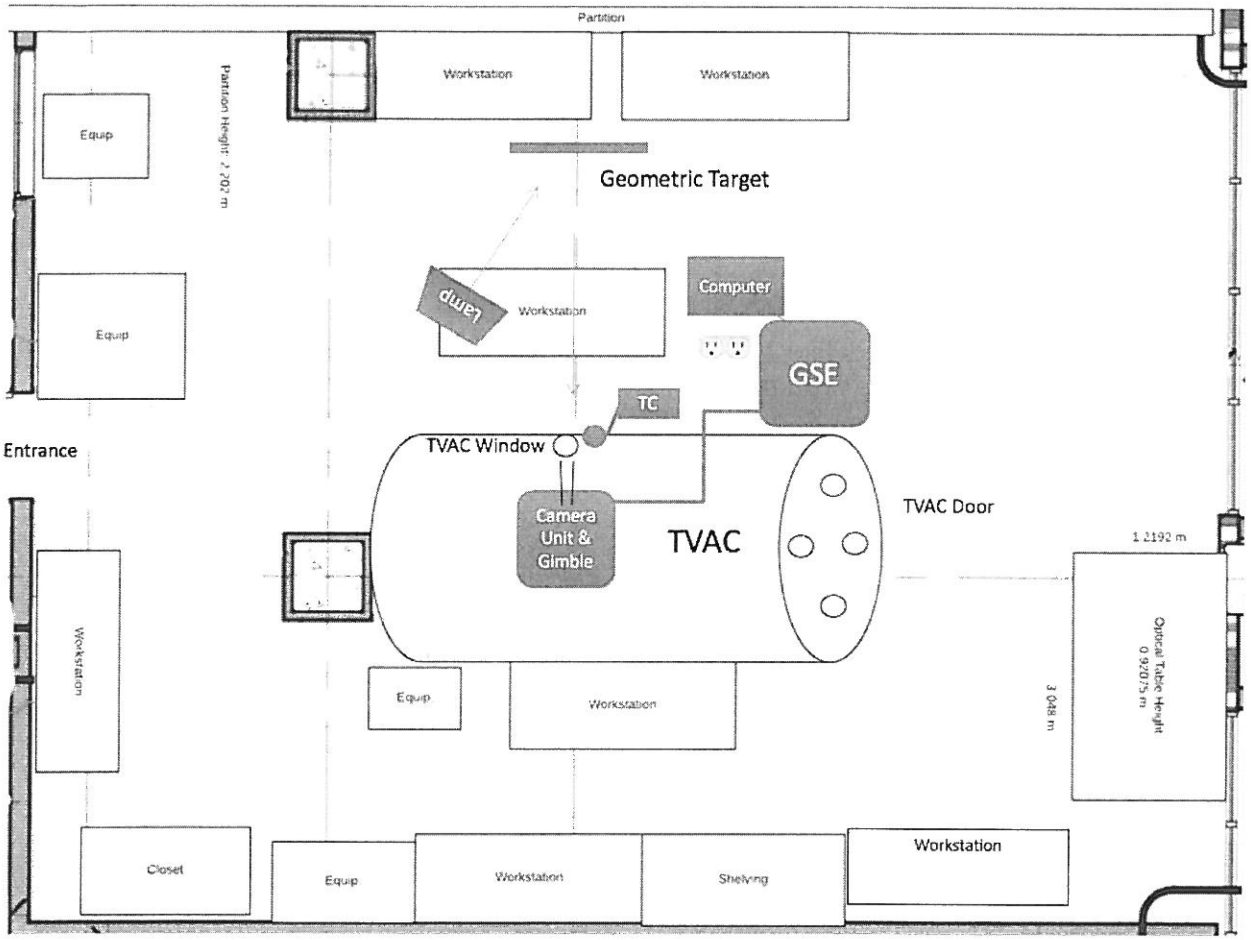

7. **[T,O, L]** ____ Ensure that all personnel in the cleanroom are following the cleanroom practices for electrostatic discharge, proper clothing, and other safety concerns. See "ESD_Manual" and "Cleanroom_Manual".
8. **[T]** ____ Double check that nitrogen is flowing over the Mastcam-Zs or the window port.
9. **[O,T]** ____ If not already done, mate the Right Mastcam-Z into the GSE. Follow the procedure in "MastcamZ_GSE_Manual".
10. **[T]** ____ Verify that the thermocouples are turned on and properly reading out.
11. **[T]** ____ Install the lamps and position them 1 meter from the geometric target out of the camera's field of view (FOV). Power them on.

12. **[O,T]** ____ Ensure that the camera unit and GSE wires are secure, kink-free, and do not present tripping hazards when the lights are turned off.
13. **[O,D]** ____ Check the camera temperature and ensure nominal operation.
14. **[D]** ____ Record the following environmental information:
    - Cleanroom temperature _______ pressure _______ humidity _______
15. **[O,D, L]** Notes:

____

____

____

Table 2. The nominal target placement scenes for the geometric testing.

	Target Placements	Notes
**Scene 1**	MTF_SN005 (Side A) at 2 meters centered on the 63mm boresight	
**Scene 2**	MTF_SN005 (Side A) at 3 meters centered on the 100mm boresight	
**Scene 3**	MTF_SN007 (Side A) at 3 meters centered on the 34mm boresight	
**Scene 4**	MTF_SN007 (Side A) at 2 meters centered on the 26mm boresight	

Date ______ Time ______ Initials ______

Scene 1 for the Right Mastcam-Z

See Next Page

16. **[M,T]** Position the MTF target to Scene 1 as described in Table 2.
17. **[M]** Measure and record the location of the MTF target.

____

____

____

18. **[D]** Record climate information:
    - TVAC temp ______________ Port temp ______________
    - Camera CCD temp ______________ Optics temp ______________
19. **[D,T]** Take digital pictures of the setup and MTF target.
20. **[O]** Capture one autofocused frame at 63mm with filter 0, and rsync data to the validator.
21. **[V,T]** Load the image in MTF Mapper to find the correct target position. Recapture an autofocused frame if necessary.
22. **[O]** Load and execute camera script **473TN10R03**, which autofocus and takes 3 frames at 63mm focal length with filter 0. Insert note "TARGET=MTF_SN005".
23. **[O]** Load and execute camera script **492TN10R06**, which autofocus and takes three frames at 63mm focal length with filters 0-6. Insert note "TARGET=MTF_SN005". The duration is 7 minutes.
24. **[D]** Record image names and parameters in Image Log.
25. **[V]** Run fast-look script to verify that required data were obtained.
26. **[D, L]** Notes: ____

____

____

Date 4-26 Time 7:20 Initials CS

Test Images to refine the future target placement procedures. CS

Scene 1 for the Right Mastcam-Z

14. **[M,T]** Position the MTF target to Scene 1 as described in Table 2.
15. **[M]** Measure and record the location of the MTF target.
This is the first MTF data set!

16. **[D]** Record climate information:
    - TVAC temp -5/-10 Port temp N/A
    - Camera CCD temp -5 Optics temp N/A
17. **[D,T]** Take digital pictures of the setup and MTF target.
18. **[O]** Capture one autofocused frame at 63mm with filter 0, and rsync data to the validator.
19. **[V,T]** Load the image in MTF Mapper to find the correct target position. Recapture an autofocused frame if necessary. 473TEMPR03
20. ~~**[O]** Load and execute camera script **472TAMBR03**, which autofocus and takes 3 frames at 63mm focal length with filter 0.~~ ~~492TEMPR06~~ 492TN10R06
21. **[O]** Load and execute camera script ~~**491TAMBR06**~~, which autofocus and takes three frames at 63mm focal length with filters 0-6. The duration is 7 minutes.
22. **[D]** Record image names and parameters in Image Log.
23. **[V]** Run fast-look script to verify that required data were obtained.
24. **[D, L]** Notes:
Ran

TARGET= MT~~F~~-SN005

The prefixes we ran are recorded in the Image Log.

Test terminated because we came to the end of our shift at 8:15 pm. CS

Another reason to stop ~~[illegible]~~ early is that we did not have an IR light source set up to image anything other than filter R0.

CS

Shutdown Procedure

1. **[D,T]** ____ Take digital pictures of this page and the test setup.
2. **[D,O]** ____ Review entries in Image Log, GSE command log, and image headers.
3. **[D, L]** ____ Review calibration procedure and ensure that each task is initialed.
4. **[D, L]** Notes: ____

 ____

 ____

5. **[V, L]** ____ Before making the decision to break down the test setup, ensure that adequate data were acquired for the test requirements. See "MastcamZCalPlan" for these requirements.
6. **[V]** Notes: ____

 ____

 ____

Data Validator (signature) ______________________

Date ______________ Time ______________

7. **[V, L]** ____ Give the go/no-go decision. Have enough data been acquired to fulfill test requirements? See "MastcamZCalPlan" for these requirements.
8. **[D, L]** ____ Update the Log Document.
9. **[L]** Notes: ____

 ____

 ____

Calibration Lead (signature) ______________________

Date ______________ Time ______________

10. **[O, L]** ____ Ensure that the camera and GSE are in a safe state.
11. **[O,D]** ____ Review the Image Log with the documentarian. Exchange high-fives.
12. **[O]** Notes: ____

____

____

Camera Operator (signature) ________________________

Date ______________ Time ______________

13. **[T]** ____ If the next test does not require the target, position it away from the chamber or bench. Otherwise, be sure not to move it. The next test is ______________.
14. **[T]** ____ Ensure that all other test equipment is safely put away.
15. **[T]** Notes: ____

____

____

Technician (signature) ____________________________

Date ______________ Time ______________

16. **[D, L]** ____ Double-check this procedure and ensure that the top of each page has valid data, time and initials.
17. **[D]** ____ Photo-scan this document, save it on the cloud, and file the hard-copy in the Log Binder. Upload the digital pictures taken during this test in the appropriate archive on the cloud. The required links are on the Wiki.
18. **[D]** ____ Double-check that every required cell the Image Log is accurately filled. When this is complete, print the Image Log and file it the Log Binder after this document.
19. **[D]** Notes: ____

____

____

Documentarian (signature) __________________________

Date ______________ Time ______________

Date 4/27 Time 8:30 Initials JGB

MTF Calibration Procedure for Mastcam-Z TVAC Testing (Pro. 4.7.~~2~~ 3)

[Procedure version ~~2.21~~ v2.02, prepared by the Mastcam-Z calibration team at Cornell University.]

These measurements are performed on the camera and at the temperature designated below as specified in the Calibration Plan (Document #),

Unit Under Test:

R FM _X_ L FM _X_ EQM___ Other___

Test Performed at Temperature:

-35°C___ - 10°C_X_ +5°C___ Ambient___ Other___

These measurements are performed at,

MSSS_X_ ASU___ Other___

Date 4/27/2019 **Start Time** 8:45 AM **End Time** ___

Estimated Duration 2.5 hours

Scheduled Start Time 8:30 AM **Sch. End Time**___

Calibration Lead [L] Jim **Documentarian [D]** Jim

Camera Operator [O] Tex **Technician [T]** Christian

Data Validator [V] Paul **Metrologist [M]** Christian

Other ANDY

Date 4/27 Time 830 Initials JFB

Table of Contents

MTF CALIBRATION PROCEDURE FOR MASTCAM-Z AMBIENT TVAC TESTING (PRO. 4.7.2) 1

TEST DESCRIPTION 3

SOFTWARE PREPARATION 4

Table 1. File naming convention for the camera script prefixes and frame filenames: "AAABBBBCDD" 4

HARDWARE INSTALLATION .... **ERROR! BOOKMARK NOT DEFINED.**

*Figure 1. ASU Floor Plan for Geometric Testing in the TVAC Chamber. The MSSS Floor Plan allows for similar target and source placements relative to the chamber window. .... **Error! Bookmark not defined.***

Table 2. The nominal target placement scenes for the geometric testing 7

SCENE 1 FOR THE RIGHT MASTCAM-Z 8

Document Approval

[signature] 4/27/19	
Approved by James Bell Date<br>Mastcam-Z PI<br>Arizona State University	Approved by Alexander Hayes Date<br>Mastcam-Z Calibration Working Group Lead, Cornell University
	[signature: Christian Tate]
Approved by Justin Maki Date<br>Mastcam-Z Deputy PI and Investigation Scientist, Jet Propulsion Laboratory	Approved by Christian Tate Date<br>Procedure Author<br>Cornell University
Approved by: Date	

Date 4/27 Time 845 Initials JCB

Test Description

Excerpt from the Calibration Plan 4.7

The objective of this test is to image well-characterized bar targets at multiple focus and zoom positions in order to characterize the Modulation Transfer Function (MTF) and depth of field of each camera. Targets should be imaged at ~50% full well using the Bayer RGB (priority 1), 805 nm, (priority 2), and remaining non-solar filters (priority 3). Obtain a minimum of 3 images of each target per filter, focus, and zoom position. Multiple images are needed to reduce errors in determining target locations in the image plane.

MTF is an effective means of specifying the resolution of an optical system. Resolution is defined as the minimum feature size of an object that can be distinguished by an imaging system. The Point Spread Function (PSF) is the inverse Fourier Transform of the MTF—the PSF describes optical performance in the spatial domain while the MTF expresses optical performance in the frequency domain. Images of the bar targets and knife edge or point source targets at various zoom and focus positions will be used to determine PSF, depth of field, and MTF. The bar target shall consist of a chart containing horizontal, vertical, and diagonal lines and bars of varying thicknesses as well as circular dots of various sub- and super-pixel sizes.

In addition to determining the optical performance of the optomechanical assemblies, the images collected during MTF/PSF calibration will also be used to determine the numerical value and repeatability of the stepper motor counts for the Hall Effect sensors used to measure the position of the focus group and two moving zoom groups in the optical zoom assemblies. This will determine the relationship between stepper motor count for each focus/zoom group, working distance, and pixel scale. Owing to thickness variations between spectral filters, focus shifts may occur and images would ideally be obtained using all non-solar filters.

Note that the Mastcam-Z instrument has an onboard focus merge algorithm that selects and merges the best-focus portions of a scene using a focus stack (or z-stack) of multiple images (up to 16) at varying focal positions. While usually only the best-focus or merged product is saved to file, the raw frames for the focus stack can also be saved to file without appreciably increasing the observation or product generation time. For tests where images at multiple focus positions are desired, such as during MTF/PSF Calibrations, the entire focus stack of images will be written to file. Since images at multiple focus positions are acquired irrespective of whether or not they are written to memory, saving multiple focus positions per zoom setting does not require additional test time (outside of the time required to write the files to disk in the GSE). Multiple focus position images shall be saved during both Stand-Alone and ATLO MTF/PSF testing.

Date 4/27 Time 8:45 Initials [illegible]

Software Preparation

The software and files required for this test are prepared well in advance of test day. This checklist ensures that the following are present, debugged, and executable: (1) all fast look scripts, (2) automated header generation of all relevant camera parameters, target positioning, and metadata, (3) all camera scripts that command the camera unit, and (4) the directories/file-paths pointing to the data repositories of this specific test.

Table 1. File naming convention for the camera script prefixes and frame filenames: "AAABBBBCDD"

Code	Name	Example	Value(s)
"AAA"	Calibration Plan Section	"411" = Cal. Plan 4.1.1 chapter 4, section 1, subsection 1	473, 492
"BBBB"	Location of test or ASU TVAC temperature	"MSSS" = test at MSSS, "ATLO" = test at JPL ATLO, "TN10" = ASU TVAC -10C, …	TN10
"C"	Camera unit under test	"L" = Left Mastcam-Z, "R" = Right Mastcam-Z, "E" =EQM, "C" =COTS	R/L
"DD"	Part of test	"00" = test set up, "01" = first radiance level …	00-12

1. **[D]** _✓_ Look up the daily calibration schedule and record the scheduled start and end time of this test on the cover page of this document. Also, fill out and double-check the other information on the cover page.
2. **[D]** _✓_ Ensure that all supplemental manuals are on hand. These are,
   - Validator_Manual, Documentarian_Manual,
   - MastcamZCalPlan
3. **[D]** _✓_ Ensure that the Image Log is present and ready to use. Find and open the Google Sheets file "Image_Log_46" (47). There is a link on the Wiki.
4. **[V]** _✓_ Check that all *Calgorithms* fast-look and validation scripts are present, up-to-date, and ready to analyze test output. Find and open the "Geometric_Calibration_46_Validation" Jupyter notebook. There is a link on the Wiki.
5. **[O]** _✓_ Check that all camera scripts required for this test are present, up-to-date and ready to command the ground support equipment (GSE). These are,

- **473TN10R00 - 473TN10R06, 492TN10R01 - 492TN10R12**
- **473TN10L00 - 473TN10L06, 492TN10L01 - 492TN10L12**

6. **[O,V,D, L]** Notes:

Hardware Installation

This procedure is for the ambient TVAC chamber testing at MSSS. Figure 1 shows the nominal layout of the TVAC chamber, workspace, Mastcam-Zs, ground support equipment (GSE), targets, sources, and other equipment necessary for this test if it happens at ASU. Although MSSS' cleanroom is different than ASU's, the placement of the targets and sources relative to the chamber window is similar.

Date 4/27 Time 850 Initials JTP

Figure 1. ASU Floor Plan for Geometric Testing in the TVAC Chamber. The MSSS Floor Plan allows for similar target and source placements relative to the chamber window.

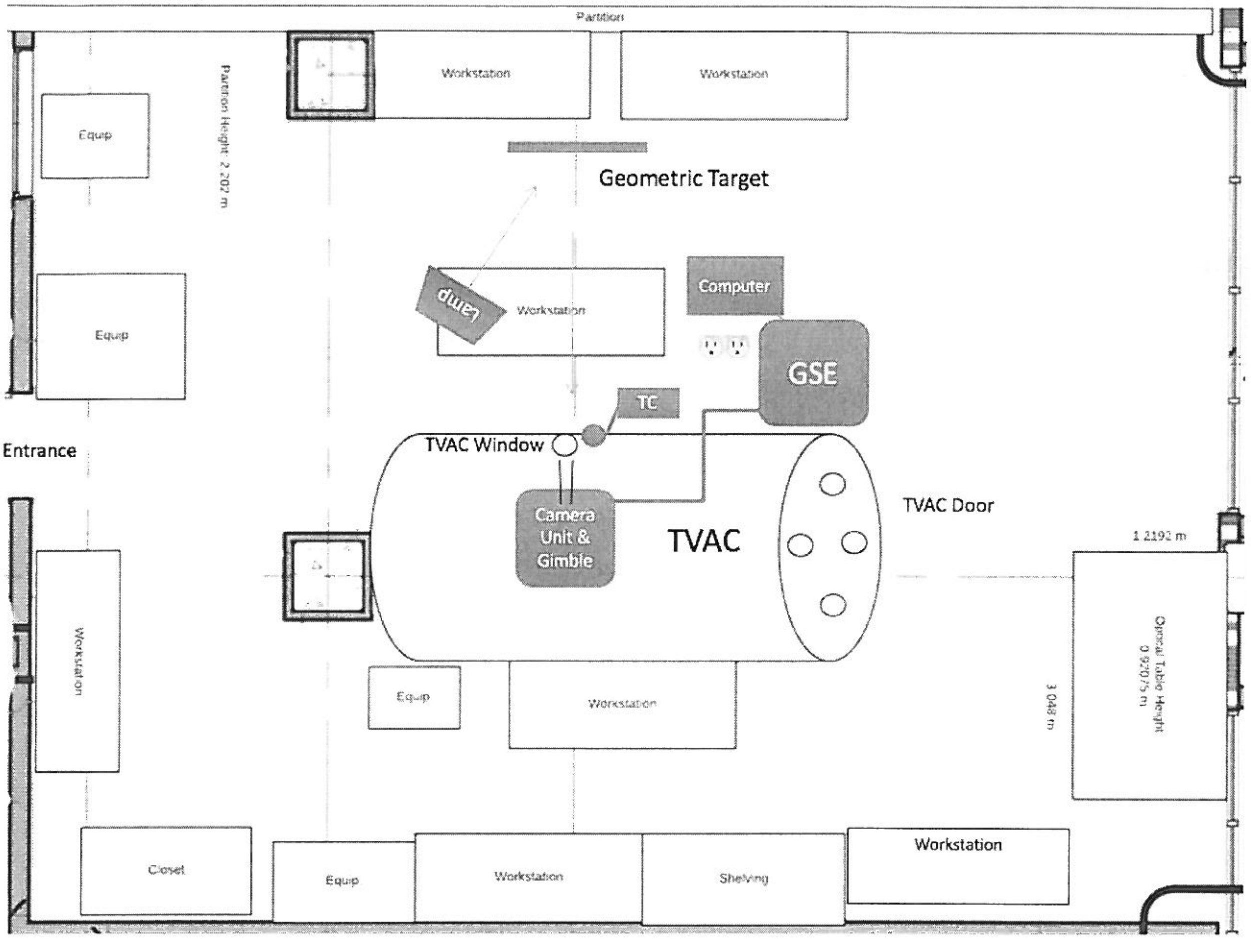

7. **[T,O, L]** ✓ Ensure that all personnel in the cleanroom are following the cleanroom practices for electrostatic discharge, proper clothing, and other safety concerns. See "ESD_Manual" and "Cleanroom_Manual".
8. **[T]** ✓ Double check that nitrogen is flowing over the Mastcam-Zs or the window port.
9. **[O,T]** ✓ If not already done, mate the Right Mastcam-Z into the GSE. Follow the procedure in "MastcamZ_GSE_Manual".
10. **[T]** ✓ Verify that the thermocouples are turned on and properly reading out.
11. **[T]** ✓ Install the lamps and position them 1 meter from the geometric target out of the camera's field of view (FOV). Power them on.

Date 4/27 Time 850 Initials JFO

12. **[O,T]** ✓ Ensure that the camera unit and GSE wires are secure, kink-free, and do not present tripping hazards when the lights are turned off.
13. **[O,D]** ✓ Check the camera temperature and ensure nominal operation.
14. **[D]** ____ Record the following environmental information:
    - Cleanroom temperature 26.8 pressure AMB humidity 38% [illegible]
15. **[O,D, L]** Notes:

photo of setup by Christina

~~[illegible]~~ C camera Temp -7.7, -5.7 L,R

Table 2. The nominal target placement scenes for the geometric testing.

	Target Placements	Notes
**Scene 1**	MTF_SN005 (Side A) at 2 meters centered on the 63mm boresight	1.65m from window To Target
**Scene 2**	MTF_SN005 (Side A) at 3 meters centered on the 100mm boresight	2.92m distance
**Scene 3**	MTF_SN007 (Side A) at 3 meters centered on the 34mm boresight	
**Scene 4**	MTF_SN007 (Side A) at 2 meters centered on the 26mm boresight	NIX – out of Time

1.65

Date 4/27 Time 920 Initials JFB

Scene 1 for the Right Mastcam-Z

✓ 16. **[M,T]** Position the MTF target to Scene 1 as described in Table 2.

✓ 17. **[M]** Measure and record the location of the MTF target.

Tweaking, Taking Test images

Distance 1.65 m

✓ 18. **[D]** Record climate information:

- TVAC temp ______ Port temp ______
- Camera CCD temp -3.4°C Optics temp ______

✓ 19. **[D,T]** Take digital pictures of the setup and MTF target.

✓ 20. **[O]** Capture one autofocused frame at 63mm with filter 0, and rsync data to the validator.

✓ 21. **[V,T]** Load the image in MTF Mapper to find the correct target position. Recapture an autofocused frame if necessary.

Skipping due to time X 22. **[O]** Load and execute camera script **473TN10R03**, which autofocus and takes 3 frames at 63mm focal length with filter 0. Insert note "TARGET=MTF_SN005".

✓ 23. **[O]** Load and execute camera script **492TN10R06**, which autofocus and takes three frames at 63mm focal length with filters 0-6. Insert note "TARGET=MTF_SN005". The duration is 7 minutes. *

✓ 24. **[D]** Record image names and parameters in Image Log.

✓ 25. **[V]** Run fast-look script to verify that required data were obtained.

✓ 26. **[D, L]** Notes:

Script aborted first time – Target bumped

Camera Temp – 2.94 for 2nd run of script.

Procedure change

* Change Insert Note To Before Load and execute...

– Auto focus failed on filter 6 (1000 nm)

→ Re-running with new af parameters for filter 6 only

– range was too small for longest-wave filter

– GSE operators homed mechanisms, powered off Right camera at 9:38 AM

Date 4/27 Time 9:40 Initials JFB

Scene 1 for the Left Mastcam-Z

✓ 27. **[M,T]** Position the MTF target to Scene 1 as described in Table 2.

28. **[M]** Measure and record the location of the MTF target.

Taking Test Images
Starting Camera Temp = -10°C
Distance = 1.65 m

✓ 29. **[D]** Record climate information:

- TVAC temp ______ Port temp ______
- Camera CCD temp -10°C Optics temp ______

✓ 30. **[D,T]** Take digital pictures of the setup and MTF target.

✓ 31. **[O]** Capture one autofocused frame at 63mm with filter 0, and rsync data to the validator.

✓ 32. **[V,T]** Load the image in MTF Mapper to find the correct target position. Recapture an autofocused frame if necessary.

SKIP X 33. **[O]** Load and execute camera script **473TN10L03**, which autofocus and takes 3 frames at 63mm focal length with filter 0. Insert note "TARGET=MTF_SN005".

✓ 34. **[O]** Load and execute camera script **492TN10L06**, which autofocus and takes three frames at 63mm focal length with filters 0-6. Insert note "TARGET=MTF_SN005". The duration is 7 minutes.

✓ 35. **[D]** Record image names and parameters in Image Log.

36. **[V]** Run fast-look script to verify that required data were obtained.

37. **[D, L]** Notes:

Script started at 9:54 am, Camera Temp at -6.0°C
finished at 10:01 am -5.2°C

All files transferred
Quick-look looks good!

Date 4/27 Time 10:08 Initials S.D.

Scene 2 for the Left Mastcam-Z

✓38. **[M,T]** Position the MTF target to Scene 2 as described in Table 2.

✓39. **[M]** Measure and record the location of the MTF target.

Target is 2.92m distance

✓ 40. **[D]** Record climate information:

- TVAC temp ______ Port temp ______
- Camera CCD temp -5.41 Optics temp ______

✓ 41. **[D,T]** Take digital pictures of the setup and MTF target.

✓42. **[O]** Capture one autofocused frame at 100mm with filter 0, and rsync data to the validator.

✓43. **[V,T]** Load the image in MTF Mapper to find the correct target position. Recapture an autofocused frame if necessary.

SKIP ✗44. **[O]** Load and execute camera script **473TN10L04**, which autofocus and takes 3 frames at 100mm focal length with filter 0. Insert note "TARGET=MTF_SN005".

✓45. **[O]** Load and execute camera script **492TN10L07**, which autofocus and takes three frames at 100mm focal length with filters 0-6. Insert note "TARGET=MTF_SN005". The duration ~~is 7 minutes~~.

✓46. **[D]** Record image names and parameters in Image Log.

✓47. **[V]** Run fast-look script to verify that required data were obtained.

✓48. **[D, L]** Notes:

Script started at 10:17. Aborted, Target bumped

Script re-started at 10:25 camera at -5.0

Script ~~completed at~~ aborted at 10:32, autofocus failed on filter 6.

⇒ af parameters expanded, filter 6 part of script re-run 10:33

failed again. re-setting parameters, re-running 10:36

failed again, manually diagnosing focus

af not working well for 440nm filter

focus set to 2730 manually for 5-stack on filter 6

filter 6, re-run script ~~at~~ without af, start at 11:03

Left camera homed and powered off at 11:07 am

Date 4/27 Time 11:08 Initials JFB

Scene 2 for the Right Mastcam-Z

✓49. **[M,T]** Position the MTF target to Scene 2 as described in Table 2.
✓50. **[M]** Measure and record the location of the MTF target.
DISTANCE TO TARGET = 3.01 m

✓51. **[D]** Record climate information:
- TVAC temp ______ Port temp ______
- Camera CCD temp −4.6°C Optics temp ______

✓52. **[D,T]** Take digital pictures of the setup and MTF target.
✓53. **[O]** Capture one autofocused frame at 100mm with filter 0, and rsync data to the validator.
✓54. **[V,T]** Load the image in MTF Mapper to find the correct target position. Recapture an autofocused frame if necessary.
SKIP ✗55. **[O]** Load and execute camera script ~~**473TN10R04**, which autofocus and takes 3 frames at 100mm focal length with filter 0. Insert~~ note "TARGET=MTF_SN005".
56. **[O]** Load and execute camera script **492TN10R07**, which autofocus and takes three frames at 100mm focal length with filters 0-6. Insert note "TARGET=MTF_SN005". The duration is 7 minutes.
✓57. **[D]** Record image names and parameters in Image Log.
58. **[V]** Run fast-look script to verify that required data were obtained.
59. **[D, L]** Notes: STARTED SCRIPT AT 12:00 CAMERA AT −2.5°C

autofocus not behaving well. Tex calling Caplinger... (11:20 AM)
Caplinger assessing (11:30 AM)
hypothesis: Too much scattered light onto Target from people moving around The room. NEW RULE: Everyone either seated, or out of The room, when acquiring data.
⇒ working well with folks in room STATIONARY!

<u>Scene 3 for the Right Mastcam-Z</u>

✓ 60. **[M,T]** Position the MTF target to Scene 3 as described in Table 2.

✓ 61. **[M]** Measure and record the location of the MTF target.

2.95m To Target

62. **[D]** Record climate information:
    - TVAC temp ______________ Port temp ______________
    - Camera CCD temp -3.1°C Optics temp ______________

✓ 63. **[D,T]** Take digital pictures of the setup and MTF target.

✓ 64. **[O]** Capture one autofocused frame at 34mm with filter 0, and rsync data to the validator.

✓ 65. **[V,T]** Load the image in MTF Mapper to find the correct target position. Recapture an autofocused frame if necessary.

SKIP X 66. **[O]** Load and execute camera script **473TN10R02**, which autofocus and takes 3 frames at 34mm focal length with filter 0. Insert note "TARGET=MTF_SN007".

67. **[O]** Load and execute camera script **492TN10R05** [illegible], which autofocus and takes three frames at 34mm focal length with filters 0-6. Insert note "TARGET=MTF_SN007". The duration is 7 minutes.

✓ 68. **[D]** Record image names and parameters in Image Log.

69. **[V]** Run fast-look script to verify that required data were obtained.

70. **[D, L]** Notes: ______________

Started script at 12:36 pm

script failed at on filter 4 → need to widen af range.
re-started at 12:36 pm, camera at -3.0°C

Date 4/27 Time 1:00p Initials JFB

Scene 3 for the Left Mastcam-Z

✓ 71. **[M,T]** Position the MTF target to Scene 3 as described in Table 2.

72. **[M]** Measure and record the location of the MTF target.

distance = 2.89m

73. **[D]** Record climate information:
- TVAC temp ________ Port temp ________
- Camera CCD temp -5.6 °C Optics temp ________

✓ 74. **[D,T]** Take digital pictures of the setup and MTF target.

✓ 75. **[O]** Capture one autofocused frame at 34mm with filter 0, and rsync data to the validator.

✓ 76. **[V,T]** Load the image in MTF Mapper to find the correct target position. Recapture an autofocused frame if necessary.

SKIP ✗ 77. **[O]** Load and execute camera script **473TN10L02**, which autofocus and takes 3 frames at 34mm focal length with filter 0. Insert note "TARGET=MTF_SN007".

✓ 78. **[O]** Load and execute camera script **492TN10L05**, which autofocus and takes three frames at 34mm focal length with filters 0-6. Insert note "TARGET=MTF_SN007". The duration is 7 minutes.

79. **[D]** Record image names and parameters in Image Log.

80. **[V]** Run fast-look script to verify that required data were obtained.

81. **[D, L]** Notes:

script started at 1:05pm, camera -5.6°C
script completed at 1:15pm -4.1°C

Validated!

Date 4/27 Time ______ Initials JFN

Scene 4 for the Left Mastcam-Z

82. **[M,T]** Position the MTF target to Scene 4 as described in Table 2.
83. **[M]** Measure and record the location of the MTF target.

____

____

____

84. **[D]** Record climate information:
    - TVAC temp ______________ Port temp ______________
    - Camera CCD temp ______________ Optics temp ______________
85. **[D,T]** Take digital pictures of the setup and MTF target.
86. **[O]** Capture one autofocused frame at 26mm with filter 0, and rsync data to the validator.
87. **[V,T]** Load the image in MTF Mapper to find the correct target position. Recapture an autofocused frame if necessary.
88. **[O]** Load and execute camera script **473TN10L02**, which autofocus and takes 3 frames at 26mm focal length with filter 0. Insert note "TARGET=MTF_SN07".
89. **[O]** Load and execute camera script **492TN10L05**, which autofocus and takes three frames at 26mm focal length with filters 0-6. Insert note "TARGET=MTF_SN007". The duration is 7 minutes.
90. **[D]** Record image names and parameters in Image Log.
91. **[V]** Run fast-look script to verify that required data were obtained.
92. **[D, L]** Notes: ____

____

____

Date 4/27 ______ Time ______ Initials JKS ______

Scene 4 for the Right Mastcam-Z

93. **[M,T]** Position the MTF target to Scene 4 as described in Table 2.
94. **[M]** Measure and record the location of the MTF target.

____

____

____

95. **[D]** Record climate information:
    - TVAC temp ______________ Port temp ______________
    - Camera CCD temp ______________ Optics temp ______________
96. **[D,T]** Take digital pictures of the setup and MTF target.
97. **[O]** Capture one autofocused frame at 26mm with filter 0, and rsync data to the validator.
98. **[V,T]** Load the image in MTF Mapper to find the correct target position. Recapture an autofocused frame if necessary.
99. **[O]** Load and execute camera script **473TN10R02**, which autofocus and takes 3 frames at 26mm focal length with filter 0. Insert note "TARGET=MTF_SN007".
100. **[O]** Load and execute camera script **492TN10R05**, which autofocus and takes three frames at 26mm focal length with filters 0-6. Insert note "TARGET=MTF_SN007". The duration is 7 minutes.
101. **[D]** Record image names and parameters in Image Log.
102. **[V]** Run fast-look script to verify that required data were obtained.
103. **[D, L]** Notes: ____

____

____

Date 4/27 Time JFB Initials 1:15pm

Shutdown Procedure

✓1. **[D,T]** ____ Take digital pictures of this page and the test setup.
✓2. **[D,O]** ____ Review entries in Image Log, GSE command log, and image headers.
✓3. **[D, L]** ____ Review calibration procedure and ensure that each task is initialed.
4. **[D, L]** Notes: ____

✓5. **[V, L]** JFB Before making the decision to break down the test setup, ensure that adequate data were acquired for the test requirements. See "MastcamZCalPlan" for these requirements.
6. **[V]** Notes: ____

Data Validator (signature) [signature]

Date 4/27/19 Time 1:15 pm

✓7. **[V, L]** JFB Give the go/no-go decision. Have enough data been acquired to fulfill test requirements? See "MastcamZCalPlan" for these requirements.
8. **[D, L]** ____ Update the Log Document.
9. **[L]** Notes: ____

Calibration Lead (signature) [signature]

Date 4/27/19 Time 1:20pm

10. **[O, L]** ✓ Ensure that the camera and GSE are in a safe state.
11. **[O,D]** ____ Review the Image Log with the documentarian. Exchange high-fives. ✓
12. **[O]** Notes: ______________________________

Camera Operator (signature) [signature]

Date 4/27 Time 1:20

13. **[T]** ✓ If the next test does not require the target, position it away from the chamber or bench. Otherwise, be sure not to move it. The next test is INT. Sphere.
14. **[T]** ✓ Ensure that all other test equipment is safely put away.
15. **[T]** Notes: ______________________________

Technician (signature) [signature]

Date 4-27 Time 1:45

16. **[D, L]** ✓ Double-check this procedure and ensure that the top of each page has valid data, time and initials.
17. **[D]** ____ Photo-scan this document, save it on the cloud, and file the hard-copy in the Log Binder. Upload the digital pictures taken during this test in the appropriate archive on the cloud. The required links are on the Wiki.
18. **[D]** ✓ Double-check that every required cell the Image Log is accurately filled. When this is complete, print the Image Log and file it the Log Binder after this document.
19. **[D]** Notes: ______________________________

Documentarian (signature) [signature]

Date 4-27 Time 1:22

Date 4/27 Time 2:00p Initials [illegible]

Radiometric Calibration Procedure for Mastcam-Z TVAC Testing at MSSS (Pro. 4.2.5)

[Procedure version 2.03, prepared by the Mastcam-Z calibration team at Cornell University]

These measurements are performed on the camera and at the temperature designated below as specified in the Mastcam-Z Calibration Plan,

Unit Under Test:

Left FM __X__ Right FM __X__ EQM____ Other________

These measurements are performed at temperature:

-35° C_____ -10°C__X__ +5°C_____ Ambient____ Other________

These measurements are performed at,

MSSS__X__ ASU_____ Other_____

Date 4/27/2019 **Start Time** 2:00pm **End Time** 8:00pm

Estimated Duration __4.5 hours__

Scheduled Start Time 11:00AM **Sch. End Time** 8:00pm

Calibration Lead [L] JIM **Documentarian [D]** PAUL

Camera Operator [O] ELSA, TEX **Technician [T]** Christian, ANDY

Data Validator [V] PAUL **Other** ________

Date 4/27 Time 2p Initials JFB

Change Log

Version	Name	Change
v1_01<br>17 Sep 2018	C. Tate	(first draft)
v1_20<br>1 Nov 2018	C. Tate	Procedure edits prior to EQM testing
v1_23<br>10 Dec. 2018	C. Tate	Procedure edits after EQM testing
v2_03<br>27 April 2019	C. Tate	Approved version prior to FM testing

Document Approval

Approved by James Bell Date
Mastcam-Z PI
Arizona State University

Approved by Alexander Hayes Date
Mastcam-Z Calibration Working Group
Lead, Cornell University

Approved by Justin Maki Date
Mastcam-Z Deputy PI and Investigation
Scientist, Jet Propulsion Laboratory

Christian Tate 4-29-19

Approved by Christian Tate Date
Procedure Author
Cornell University

Approved by Date

Table of Contents

RADIOMETRIC CALIBRATION PROCEDURE FOR MASTCAM-Z TVAC TESTING AT MSSS (PRO. 4.2.5)....................... 1

CHANGE LOG..2
DOCUMENT APPROVAL 2
TEST DESCRIPTION..4
SOFTWARE PREPARATION 4
Table 1. File naming convention for the camera script prefixes and frame filenames: "AAABBBBCDD"............4
HARDWARE INSTALLATION 6
Figure 1. ASU Floor Plan for Geometric Testing in the TVAC Chamber. The MSSS Floor Plan allows for similar target and source placements relative to the chamber window. 6
Table 2. The Nominal Radiance Values (calibrated integrating sphere output). 8

RIGHT AND LEFT MASTCAM-Z TESTS 9

CENTER THE INTEGRATING SPHERE...9
RADIANCE VALUE 1 FOR THE RIGHT MASTCAM-ZS..10
RADIANCE VALUE 1 FOR THE LEFT MASTCAM-ZS..11
RADIANCE VALUE 2 FOR THE LEFT MASTCAM-Z 12
RADIANCE VALUE 2 FOR THE RIGHT MASTCAM-Z 13
DATA VALIDATION...14

SHUTDOWN PROCEDURE 15

Test Description

Excerpt from the Calibration Plan 4.2,

> The objectives of these tests are to derive flat field images as well as the coefficients to allow a conversion from reduced (bias, dark, and flat field corrected) DN/s to absolute radiometric response (W/cm^2/sr per filter) for (a) the R, G, and B microfilters of the Bayer Pattern Filter detectors in each camera head (clear filter), (b) the 14 non-solar Mastcam-Z spectral filters "Science Filters", and, if time permits, (c) the two Mastcam-Z neutral density solar filters; and to provide an estimate of the uncertainty in these coefficients and, at Priority 2, their temperature dependence. This test builds off the Section 4.3 – Spectral Throughput Calibration to accurately account for the filter spectral response in the conversion. The requirement of knowing the relative response on the shape of the spectral throughput to ±5% combined with the absolute Radiance accuracy of the integration sphere at ±5% still allows the ±10% absolute radiometric calibration requirement to be met.

Software Preparation

The software and files required for this test are prepared well in advance of test day. This checklist ensures that the following are present, debugged, and executable: (1) all fast-look scripts, (2) automated header generation of all relevant camera parameters, target positioning, and metadata, (3) all camera scripts that command the camera unit, and (4) the directories/file-paths pointing to the data repositories of this specific test.

Table 1. File naming convention for the camera script prefixes and frame filenames: "AAABBBBCDD"

Code	Name	Example	Value
"AAA"	Calibration Plan Section	"411" = Cal. Plan 4.1.1 chapter 4, section 1, subsection 1	423, 412
"BBBB"	Location of test or ASU Chamber temperature	"MSSS" = test at MSSS, "TN10" = ASU TVAC -10C, …	TAMB
"C"	Camera unit under test	"L" = Left Mastcam-Z, "R" = Right Mastcam-Z, "E" =EQM, "C" =COTS	R/L
"DD"	Part of test (radiance value)	"00" = test set up, "01" = first radiance value …	00-08

1. **[D]** ____ Look up the daily calibration schedule and record the scheduled start and end time of this test on the cover page of this document. Also fill out and double-check the other information on the cover page.
2. **[D]** ____ Ensure that all supplemental manuals are on hand. These are,
   - Labsphere_Manual,
   - Validator_Manual, Documentarian_Manual
   - MastcamZCalPlan
3. **[D]** ____ Ensure that the Image Log is present and ready to use. Find and open the Google Sheets file "Image_Log_42". There is a link on the Wiki.
4. **[V]** ____ Check that all Calgorithms fast-look and validation scripts are present, up-to-date, and ready to analyze test output. Find and open the "Radiometric_Calibration_42_Validation" Jupyter notebook. There is a link on the Wiki.
5. **[O]** ____ Check that all camera scripts required for this test are present, up-to-date and ready to command the ground support equipment (GSE). These are,
   - **413TN10R00 - 413TN10R08** and **425TN10R00 - 425TN10R06**
   - **413TN10L00 - 413TN10L08** and **425TN10L00 - 425TN10L06**
6. **[O,V,D, L]** Notes:

____

____

____

Hardware Installation

This procedure is for the ambient TVAC chamber testing at MSSS. Figure 1 shows the nominal layout of the TVAC chamber, workspace, Mastcam-Zs, ground support equipment (GSE), targets, sources, and other equipment necessary for this test if it happens at ASU. Although MSSS' cleanroom is different than ASU's, the placement of the targets and sources relative to the chamber window is similar.

Figure 1. ASU Floor Plan for Geometric Testing in the TVAC Chamber. The MSSS Floor Plan allows for similar target and source placements relative to the chamber window.

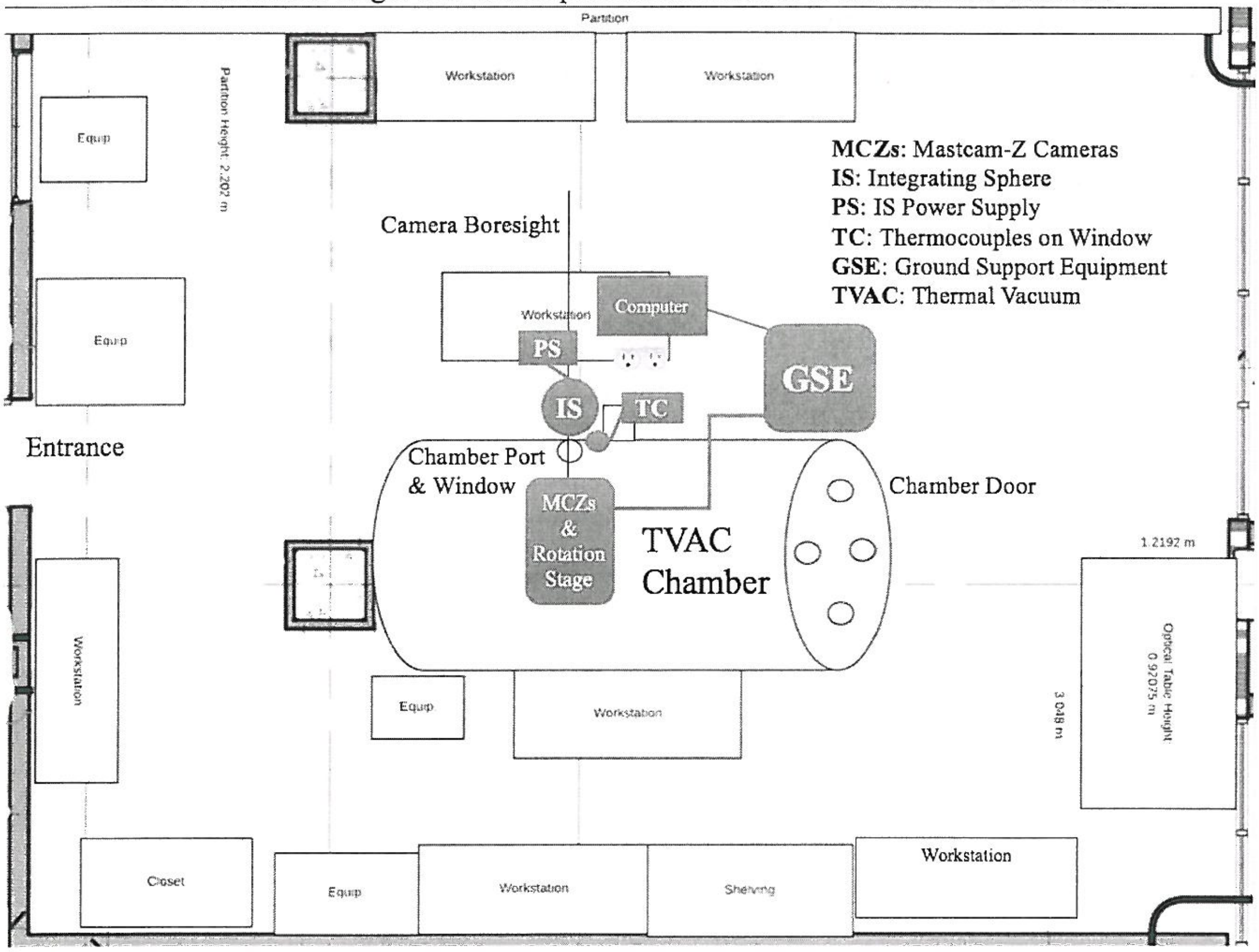

7. **[T,O, L]** ✓ Ensure that all personnel in the cleanroom are following the cleanroom practices for electrostatic discharge, proper clothing and other safety concerns.
8. **[T]** ✓ Double check that nitrogen is flowing over the Mastcam-Zs or the window port.

Date 4/22/19 Time 2:15p Initial JFB

9. [O,T] ✓ If not already done, mate the Right and Left Mastcam-Zs into the GSE. Follow the procedure in "MastcamZ_GSE_Manual".
10. [T] ✓ Verify that the thermocouples are turned on and properly reading out.
11. [O,T] ✓ Ensure that the camera unit and GSE wires are secure, kink-free, and do not present tripping hazards when the lights are turned off.
12. [O,D] ✓ Check the camera temperature and ensure nominal operation. -8.4°C 2:20pm
13. [D] ✓ Record the following environmental information:
    - Cleanroom temperature 25°C pressure [illegible] amb. humidity 38%
14. [O,D, L] Notes:

____

____

____

15. [D,T] ____ Take time-stamped pictures of this page, the integrating sphere, and the whole test/GSE set-up.
16. [T] ✓ Power on the integrating sphere. Follow the procedure in "Labsphere_Manual". Record the time the lamp is turned on 2:00 pm.
17. [D,T] ✓ Record the exact readout value of the integrating sphere's radiance: 5.52 mW/cm2/sr.
18. [T,O, L] ✓ Confirm that the camera systems and GSEs are powered on and ready for use. Follow the procedure in "MastcamZ_GSE_Manual".
19. [D, L] Notes:

Port is 3 mm from the window

____

____

NOTE: Make sure electronics is properly rotated on sphere so Tech can see the readout panel

Table 2. The Nominal Radiance Values (calibrated integrating sphere output).

IS Output Radiances	Nominal Radiance [mW/cm2/sr]	
Radiance 1	5.0	5.2
Radiance 2	10.0	10.2

Right and Left Mastcam-Z Tests

Center the Integrating Sphere

20. **[T]** ✓ Move integrating sphere output as close to the chamber window as possible centered on the Right Mastcam-Z boresight.
21. **[O]** ✓ Insert the note "ISOP=[radiance]" (5.52) and execute camera script **425TN10R00**. This script captures one auto-exposure at 40% full-well and one bias frame for filter 0 at 26 mm focal length.
22. **[V,O,T]** ✓ Open images, and if the images show that the integrating sphere is not centered, center the integrating sphere disc in the frame. Recapture **425TN10R00** frames if necessary.
23. **[D]** ✓ Record image names and ~~parameters in the image Log.~~
24. **[O,D,V]** ✓ Approximate the full-well percentage of the center pixels of the image and verify that they are about 40% (or DN 155). → Red, Green, or Blue?
25. **[T]** ✓ Lights off
26. **[D, L]** Notes:

MODS:

1) Choose sub-frame for auto-exposure
672 624 304 224 = sub-frame parameters

⇒ change scripts later!

Date 4/27 Time 3:15 Initial JHB

Radiance Value 1 for the Right Mastcam-Zs

27. **[T]** ✓ Set integrating sphere output to the radiance value 1 defined in Table 2.
28. **[D,T]** ✓ Record exact integrating sphere readout value 5.288 mW/cm2/sr.
29. **[D]** ____ Record temperature information:
    - Chamber temp ________ Port temp ________
    - Camera CCD temp -3.3 °C Optics temp ________
30. **[D,T]** ✓ Take time-stamped digital pictures of the setup and integrating sphere readout.
31. **[O]** ✓ Insert the note "ISOP=[radiance]" and execute camera script **425TN10R02**, which captures 5 frames for 40% and 80% full-well and 10 bias frames with the 7 non-solar filters at three focal lengths. The estimated duration is 35 minutes.
32. **[D,T]** ✓ Record exact integrating sphere readout value ~~5.290~~ 5.321 mW/cm2/sr.
33. **[O]** ✓ Insert the note "ISOP=[radiance]" and execute camera script **413TN10R05**, which captures 10 frames for 9 exposure times with filter 0 at 100mm focal length. The estimated duration is 9 minutes.
34. **[D,T]** ✓ Record exact integrating sphere readout value 5.283 mW/cm2/sr.
35. **[D]** ✓ Record image names and parameters in the Image Log.
36. **[D, L]** Notes: Run Step #33 first, started at 3:27 pm
    Run Step #31 second, start at 3:49 pm
    completed final script at 4:30pm, camera at -2.1°C

Kludge: Set auto-exposure threshold to 1%, then
Target DN = ~~110~~ 102 hits ≈ 40% full well mark
DN = 155 " ≈ 80% " ⇐ Too high?

Run photon transfer test first, use to tweak the 40% and 80% flux levels...

NOTE
sphere 5.333 at end of last script.
moving sphere back away from window, sphere now reads 5.193
moving sphere close again: 5.318

2.4% change in radiance due to window reflection

Date 7/27 Time 4:35 Initial JFB

Radiance Value 1 for the Left Mastcam-Zs

37. **[T]** ✓ Center the integrating sphere for the Left Mastcam-Z. Do not change the radiance value.
38. **[O]** ✓ Insert the note "ISOP=[radiance]" and execute camera script **425TN10L00.** This script captures one auto-exposure at 40% full-well and one bias frame for filter 0 at 26 mm focal length.
39. **[V,O,T]** ✓ Open images, and if the images show that the integrating sphere is not centered, center the integrating sphere disc in the frame. Recapture **425TN10L00** frames if necessary.
40. **[D]** ✓ Record image names and parameters in the image Log.
41. **[D,T]** ✓ Record exact integrating sphere readout value 5.318 mW/cm2/sr.
42. **[D]** ✓ Record temperature information:
    - Chamber temp ________ Port temp ________
    - Camera CCD temp -5.3°C Optics temp ________
43. **[D,T]** ✓ Take time-stamped digital pictures of the setup and integrating sphere readout.
44. **[O]** ✓ Insert the note "ISOP=[radiance]" and execute camera script **425TN10L02,** which captures 5 frames for 40% and 80% full-well and 10 bias frames with the 7 non-solar filters at three focal lengths. The estimated duration is ~~35 minutes~~.
45. **[D,T]** ✓ Record exact integrating sphere readout value 5.321 mW/cm2/sr.
46. **[O]** ✓ Insert the note "ISOP=[radiance]" and execute camera script **413TN10L05,** which captures 10 frames for 9 exposure times with filter 0 at 100mm focal length. The estimated duration is 9 minutes.
47. **[D,T]** ____ Record exact integrating sphere readout value 5.318 mW/cm2/sr.
48. **[D]** ____ Record image names and parameters in the Image Log.
49. **[D, L]** Notes: STARTED STEP #46 AT 4:40pm ended AT 4:50, camera at -4.8°C STARTED STEP #44 AT 4:54pm ended AT 5:34, CAM at -4.6°C sphere @ 5.316

Radiance Value 2 for the Left Mastcam-Z

50. **[T]** ✓ Set integrating sphere output to the radiance value 2 defined in Table 2.
51. **[D,T]** ✓ Record exact integrating sphere readout value 10.029 mW/cm2/sr.
52. **[D]** ✓ Record temperature information:
    - Chamber temp ________ Port temp ________
    - Camera CCD temp -5.1°C Optics temp ________
53. **[D,T]** ✓ Take time-stamped digital pictures of the setup and integrating sphere readout.
54. **[O]** ✓ Insert the note "ISOP=[radiance]" and execute camera script **425TN10L02**, which captures 5 frames for 40% and 80% full-well and 10 bias frames with the 7 non-solar filters at three focal lengths. The estimated duration is 35 minutes.
55. **[D,T]** ✓ Record exact integrating sphere readout value 10.29 mW/cm2/sr.
56. **[O]** ✓ Insert the note "ISOP=[radiance]" and execute camera script **413TN10L08**, which captures 10 frames for 9 exposure times with filter 0 at 100mm focal length. The estimated duration is 9 minutes.
57. **[O]** ✓ If time permits, insert the note "ISOP=[radiance]" and execute camera script **425TN10L05**, which captures 5 frames for 40% and 80% full-well and 5 bias frames with filters 0 and 1 at seven focal lengths. These images are ~~compounded~~ companded at 8-bits. The estimated duration is 12 minutes.
58. **[D,T]** ✓ Record exact integrating sphere readout value 10.284 mW/cm2/sr.
59. **[D]** ✓ Record image names and parameters in the Image Log.
60. **[D, L]** Notes:

Started #56 at 5:40 pm, CCD @ -5.1°C, done at 5:50
Started #54 at 5:52 pm, CCD @ -5.4°C, done at 6:32
Started #57 at 6:35 pm, CCD @ -5.7°C, done at 6:46

Left camera homed and powered off at 6:50pm

Date 4/27 Time ______ Initial JFB

Radiance Value 2 for the Right Mastcam-Z

61. **[T]** ✓ Center the integrating sphere for the Right Mastcam-Z. Do not change the radiance value.
62. **[O]** ✓ Insert the note "ISOP=[radiance]" and execute camera script **425TN10R00**. This script captures one auto-exposure at 40% full-well and one bias frame for filter 0 at 26 mm focal length.
63. **[V,O,T]** ✓ Open images, and if the images show that the integrating sphere is not centered, center the integrating sphere disc in the frame. Recapture **425TN10R00** frames if necessary.
64. **[D]** ✓ Record image names and parameters in the image Log.
65. **[D,T]** ✓ Record exact integrating sphere readout value 10.30 mW/cm2/sr.
66. **[D]** ✓ Record temperature information:
    - Chamber temp __________ Port temp __________
    - Camera CCD temp -5.5°C Optics temp __________
67. **[D,T]** ✓ Take time-stamped digital pictures of the setup and integrating sphere readout.
68. **[O]** ✓ Insert the note "ISOP=[radiance]" and execute camera script **425TN10R02**, which captures 5 frames for 40% and 80% full-well and 10 bias frames with the 7 non-solar filters at three focal lengths. The estimated duration is 35 minutes.
69. **[D,T]** ✓ Record exact integrating sphere readout value 10.290 mW/cm2/sr.
70. **[O]** ____ Insert the note "ISOP=[radiance]" and execute camera script **413TN10R08**, which captures 10 frames for 9 exposure times with filter 0 at 100mm focal length. The estimated duration is 9 minutes.
71. **[O]** ✓ If time permits, insert the note "ISOP=[radiance]" and execute camera script **425TN10R05**, which captures 5 frames for 40% and 80% full-well and 5 bias frames with filters 0 and 1 at seven focal lengths. These images are compounded at 8-bits. The estimated duration is 12 minutes.
72. **[D,T]** ✓ Record exact integrating sphere readout value 10.30 mW/cm2/sr.
73. **[D]** ✓ Record image names and parameters in the Image Log.

Sphere was at 9.99 when pulled away from window.

Date 4/27 Time 656 Initial [illegible]

74. **[D, L]** Notes: Started #70 at 6:54 pm, CCD @ -5.5°C. Done: 7:02
#68 at 7:05 pm, CCD @ -4.4°C. Done: 7:45 pm
#71 at 7:50 pm, CCD @ -3.4°C. Done: 8:00 pm

Data Validation

75. **[T]** ✓ Lights on
76. **[V]** ✓ Upload data to server.
77. **[V]** ✓ Run the "Radiometric_Calibration_42_Validation" Jupyter notebook on the acquired data for the Right Mastcam-Z with the window off. This analysis can take place while the test continues.
    - Create preliminary flat-field images and radiometric coefficients for each filter.
    - Save results in the calibration records.
78. **[V,D, L]** Notes: ______

Date 4/27 Time ______ Initial JFB

Shutdown Procedure

79. **[D,T]** ✓ Take digital pictures of this page and the test setup.
80. **[D,O]** ✓ Review entries in Image Log, GSE command log, and image headers.
81. **[D, L]** ✓ Review calibration procedure and ensure that each task is initialed.
82. **[D, L]** Notes: ____________________

83. **[V, L]** ✓ Before making the decision to break down the test setup, ensure that adequate data were acquired for the test requirements. See "MastcamZCalPlan" for these requirements.
84. **[V]** Notes: ____________________

Data Validator (signature) [signature]

Date 4-27-19 Time 8:02 PM

85. **[V, L]** ✓ Give the go/no-go decision. Have enough data been acquired to fulfill test requirements? See "MastcamZCalPlan" for these requirements.
86. **[D, L]** ✓ Update the Log Document.
87. **[L]** Notes: ____________________

Calibration Lead (signature) [signature]

Date 4/27/19 Time 8:00pm

88. **[O, L]** ✓ Ensure that the camera and GSE are in a safe state.
89. **[O,D]** ✓ Review the Image Log with the documentarian. Exchange high-fives.
90. **[O]** Notes: ____

Camera Operator (signature) Elsa Jensen

Date 4/27/19 Time 8:05 pm

91. **[T]** ✓ If the next test does not require the integrating sphere, position it away from the chamber or bench. Otherwise, be sure not to move it. The next test is IR Target.
92. **[T]** ✓ Ensure that all other test equipment is safely put away.
93. **[T]** Notes: ____

Technician (signature) Christian [illegible]

Date 4-27 Time 8:08

94. **[D, L]** ✓ Double-check this procedure and ensure that the top of each page is initialed with the time and date.
95. **[D]** ____ Photo-scan this document, save it on the cloud, and file the hardcopy in the Log Binder. Upload the digital pictures taken during this test in the appropriate archive on the cloud. The required links are on the Wiki.
96. **[D]** ✓ Double-check that every required cell the Image Log is accurately filled. When this is complete, print the Image Log and file it the Log Binder after this document.
97. **[D]** Notes: ____

Documentarian (signature) [illegible]

Date 4/27/2019 Time 8:03 PM

Date 4/28 Time 8:30 Initials JFB

JR Geometric Calibration Procedure for the Right and Left Mastcam-Z TVAC Testing at MSSS (Pro. 4.6.5-8)

[Procedure version 2.04, prepared by the Mastcam-Z calibration team at Cornell University]

These measurements are performed on the camera and at the temperature designated below as specified in the Mastcam-Z Calibration Plan,

Unit Under Test:

Left FM X **Right FM** X **EQM**_____ **Other**_____

These measurements are performed at temperature:

-35°C_____ **-10°C** X **+5°C**_____ **Ambient**_____ **Other**_____

These measurements are performed at,

MSSS X **ASU**_____ **Other**_____

Date 4/28/2019 **Start Time** _____ **End Time** _____

Estimated Duration 8.0 hours

Scheduled Start Time 830 **Sch. End Time** 1730

Calibration Lead [L] JIM **Documentarian [D]** Megan

Camera Operator [O] Tex, Elsa **Technician [T]** Andy, Christian

Data Validator [V] Paul **Metrologist [M]** Informal, Christian

Other _____

Change Log

Version	Name	Change
v1_01<br>26 Sep 2018	C. Tate	(first draft)
v1_07<br>1 Nov 2018	C. Tate	Procedure edits prior to EQM testing
V1_07-JR<br>8 Nov 2018	G. Paar	Distances more precisely reflected, change mode from v06 to v07 kept, fixed focus consistently at 2 tables & figure automatically referenced
v1_10<br>13 Dec. 2018	C. Tate	Procedure edits after EQM testing
v2_04<br>28 April 2019	C. Tate	Approved version prior to FM testing

Document Approval

X

Approved by James Bell Date
Mastcam-Z PI
Arizona State University

Approved by Alexander Hayes Date
Mastcam-Z Calibration Working Group
Lead, Cornell University

Approved by Justin Maki Date
Mastcam-Z Deputy PI and Investigation
Scientist, Jet Propulsion Laboratory

Christian Tate 4-30-19
Approved by Christian Tate Date
Procedure Author
Cornell University

Approved by: Date

Date 4/28 Time 830 Initials JFB

Table of Contents

JR GEOMETRIC CALIBRATION PROCEDURE FOR THE RIGHT AND LEFT MASTCAM-Z TVAC TESTING AT MSSS (PRO. 4.6.5-8) 1

CHANGE LOG 2
DOCUMENT APPROVAL 2
TEST DESCRIPTION 4
SOFTWARE PREPARATION 4
Table 1. File naming convention for the camera script prefixes and frame filenames: "AAABBBBCDD" 4
HARDWARE INSTALLATION 6
Figure 1. ASU Floor Plan for Geometric Testing in the TVAC Chamber. The MSSS Floor Plan allows for similar target and source placements relative to the chamber window. 6
EXPLANATION OF THE SEMI-RANDOM ORIENTATIONS 8
Figure 2. An example of the camera's FOV (black) and JR dot target's semi-random positions (red) 8
100+ TARGET POSITIONS FOR THE 48MM RIGHT AND LEFT MASTCAM-ZS 9
DATA VALIDATION 11
FIXED TARGET POSITIONS FOR THE 34MM RIGHT MASTCAM-Z (SCENE 1) 12
FIXED TARGET POSITIONS FOR THE 63MM RIGHT MASTCAM-Z (SCENE 2) 14
FIXED TARGET POSITIONS FOR THE 100MM RIGHT MASTCAM-Z (SCENE 3) 16
FIXED TARGET POSITIONS FOR THE 34MM LEFT MASTCAM-Z (SCENE 4) 18
FIXED TARGET POSITIONS FOR THE 63MM LEFT MASTCAM-Z (SCENE 5) 20
FIXED TARGET POSITIONS FOR THE 100MM LEFT MASTCAM-Z (SCENE 6) 22
DATA VALIDATION 24
TIME CHECK 1 24
FIXED TARGET POSITIONS FOR THE 26MM RIGHT MASTCAM-Z (SCENE 7) 25
FIXED TARGET POSITIONS FOR THE 26MM LEFT MASTCAM-Z (SCENE 8) 27
TIME CHECK 2 29
100+ TARGET POSITIONS FOR THE 63MM RIGHT AND LEFT MASTCAM-ZS 30
DATA VALIDATION 32

SHUTDOWN PROCEDURE 33

Test Description

Excerpt from the Calibration Plan 4.6

The objective of Geometric Calibration is to characterize the geometric distortion introduced by the Mastcam-Z optics into its images, and measure the effective focal length and field of view at each focus and zoom position. As the range of zoom positions available to Mastcam-Z represent a continuum, measurements will be acquired at a finite number of zoom settings and then interpolated to characterize distortion and other geometric parameters across the full zoom range. Targets should be imaged at ~50% full well using the Bayer RGB/805 nm (priority 1) and remaining non-solar filters (priority 3). The calibration data will be used to generate a geometric model for each camera. The camera models may exhibit wavelength dependence, so an attempt to measure the effect overall filters is desired (although not required).

Software Preparation

The software and files required for this test are prepared in advance of test day. This checklist ensures that the following are present, debugged, and executable: (1) all fast look scripts, (2) automated header generation of all relevant camera parameters, target positioning, and metadata, (3) all camera scripts that command the camera unit, and (4) the directories/file-paths pointing to the data repositories of this specific test.

Table 1. File naming convention for the camera script prefixes and frame filenames: "AAABBBBCDD"

Code	Name	Example	Value(s)
"AAA"	Calibration Plan Section	"465" = Cal. Plan 4.6.5 chapter 4, section 6, subsection 5	465-8
"BBBB"	Location of test or ASU Chamber temperature	"ATLO" = test at JPL ATLO, "TN10" = MSSS TVAC -10C, …	TEMP
"C"	Camera unit under test	"L" = Left Mastcam-Z, "R" = Right Mastcam-Z, "E" =EQ "C" =COTS	L/R
"DD"	Part of test	"00" = test set up, "01" = first part,…	00-13

1. **[D]** ✓ Look up the daily calibration schedule and record the scheduled start and end time of this test on the cover page of this document. Also, fill out and double-check the other information on the cover page.
2. **[D]** ✓ Ensure that all supplemental manuals are on hand. These are,
   - Validator_Manual, Documentarian_Manual, MastcamZ_Data_Manual,
   - MastcamZCalPlan ~~47~~ OK
3. **[D]** ✓ Ensure that the Image Log is present and ready to use. Find and open the Google Sheets file "Image_Log_46". There is a link on the Wiki.
4. **[V]** ✓ Check that all *Calgorithms* fast-look and validation scripts are present, up-to-date, and ready to analyze test output. Find and open the "Geometric_Calibration_46_Validation" Jupyter notebook. There is a link on the Wiki.
5. **[O]** ✓ Check that all camera scripts required for this test are present, up-to-date and ready to command the ground support equipment (GSE). These are,
   - **465TEMPR01 - 465TEMPR09, 465TEMPL01 - 465TEMPL09**
   - **466TEMPR01 - 466TEMPR13, 466TEMPL01 - 466TEMPL13**
   - **467TEMPR01 - 467TEMPR04, 467TEMPL01 - 467TEMPL04**

6. **[O,V,D, L]** Notes:

___

___

___

Date 4/28 Time 840 Initials [illegible]

Hardware Installation

This procedure is for the ambient TVAC chamber testing at MSSS. Figure 1 shows the nominal layout of the TVAC chamber, workspace, Mastcam-Zs, ground support equipment (GSE), targets, sources, and other equipment necessary for this test if it happens at ASU. Although MSSS' cleanroom is different than ASU's, the placement of the targets and sources relative to the chamber window is similar.

Figure 1. ASU Floor Plan for Geometric Testing in the TVAC Chamber. The MSSS Floor Plan allows for similar target and source placements relative to the chamber window.

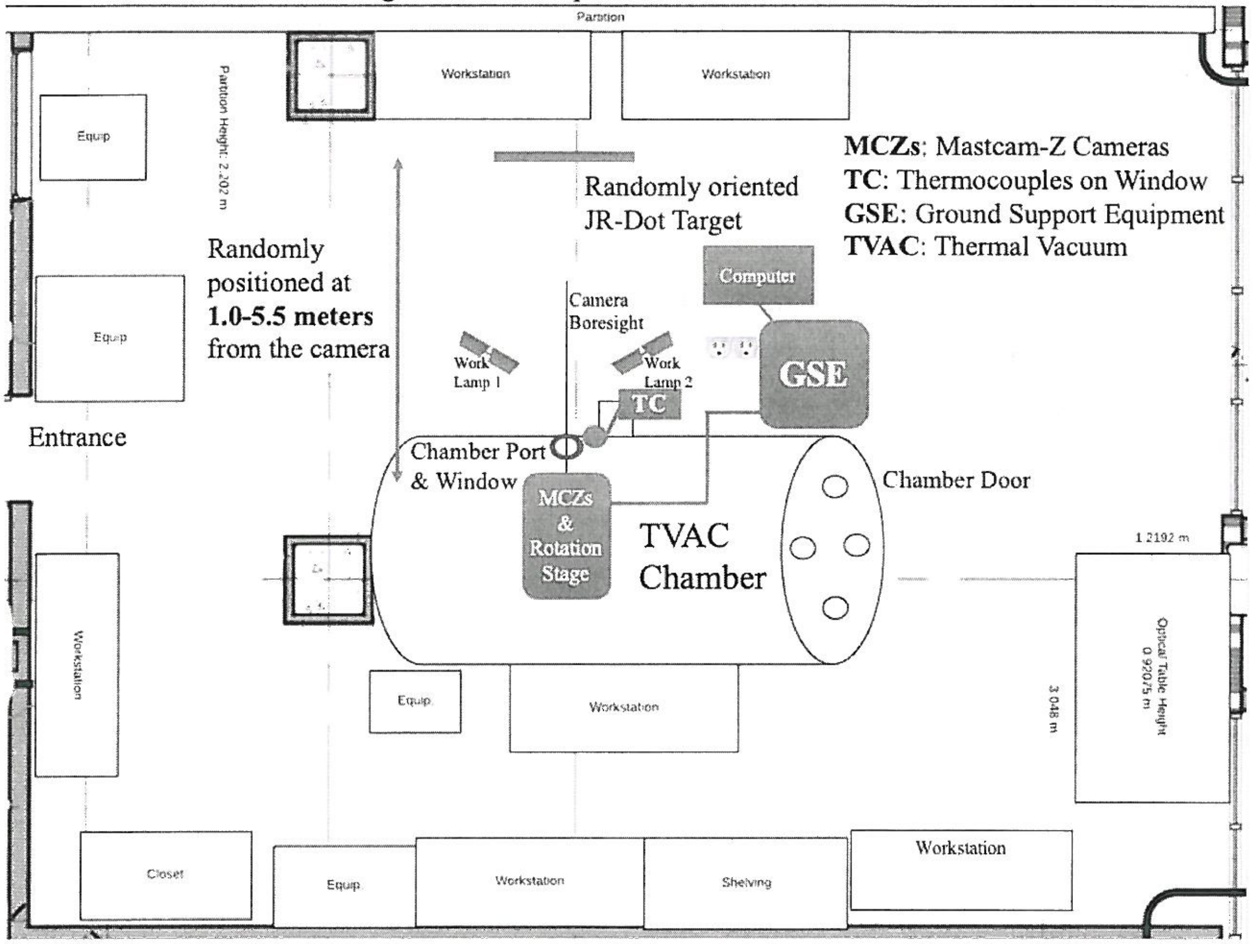

7. **[T,O, L]** ✓ Ensure that all personnel in the cleanroom are following the cleanroom practices for electrostatic discharge, proper clothing, and other safety concerns. See "ESD_Manual" and "Cleanroom_Manual".

Date 4/28 Time 845 Initials JFB

8. **[T]** ✓ Double check that nitrogen is flowing over the Mastcam-Zs or the window port.
9. **[O,T]** ✓ If not already done, mate the Right Mastcam-Z into the GSE. Follow the procedure in "MastcamZ_GSE_Manual".
10. **[T]** ✓ Verify that the thermocouples are turned on and properly reading out.
11. **[T]** ✓ Position the JR dot target approximately **2 meters** from the cameras
12. **[T]** ✓ Install the lamps and position them about 1 meter from the geometric target out of the camera's field of view (FOV). Power them on.
13. **[O,T]** ✓ Ensure that the camera unit and GSE wires are secure, kink-free, and do not present tripping hazards when the lights are turned off.
14. **[O,D]** ✓ Check the camera temperature and ensure nominal operation.
15. **[D]** ✓ Record the following environmental information:
    - Cleanroom temperature 26.6°C pressure amb humidity 41%
16. **[O,D, L]** Notes:

____

____

____

Date 4/28 Time 845 Initials JHB

Explanation of the Semi-Random Orientations

Figure 2. An example of the camera's FOV (black) and JR dot target's semi-random positions (red)

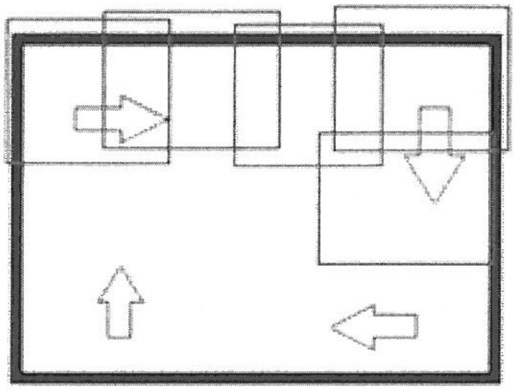

Figure 2 shows the desired orientations for the following tests that ask for a certain number of "semi-random orientations" of the JR dot target. The blue arrows show the motion of the target across the camera's FOV for optimal coverage. Note that some frames should be taken with the JR dot target rotated 90-degrees around the camera's line-of-sight.

17. **[O,T, L]** ✓ Capture a test frame with both cameras at 48mm to confirm that the chamber window does not clip the FOV (save images with prefixes **465TEMPR00** and **465TEMPL00**). If there is clipping, the next section will be taken at 63mm instead of 48mm.

18. **[D, L]** Notes:____

Date 4/28 Time 845 Initials JFB

100+ Target Positions for the 48mm Right and Left Mastcam-Zs

19. **[T]** ✓ Position the JR dot target approximately **2 meters** from the camera. Adjust lights accordingly.
20. **[D]** ✓ Record the following temperatures:
    - Chamber temp ________ Port temp ________
    - Camera CCD temp -4.1°C Optics temp ________
21. **[D,T]** ✓ Take digital pictures of the geometric target's position, and the whole test/GSE set-up.
22. **[O,T]** ✓ Capture test frames to find a standard exposure time for the 100 positions at ~~3~~ 3 OK meters focus. Save these test frames with the prefix name **465TEMPR00**, and update "var1" in the script **465TEMPR03** once this exposure time is found. 100 msec UPDATE NOTE *
23. ✓ **[V,O,T]** Evaluate whether the target's dots are in-focus enough for discrimination. If the dots are too out-of-focus for JR's algorithm, move the target back.
24. **[O,T]** ✓ Capture test frames to find a standard exposure time for the 100 positions at ~~3~~ 3 ok meters focus. Save these test frames with the prefix name **465TEMPL00**, and update "var1" in the script **465TEMPL03** once this exposure time is found. 100 msec
25. ✓ **[V,O,T]** Evaluate whether the target's dots are in-focus enough for discrimination. If the dots are too out-of-focus for JR's algorithm, move the target back.
26. ✓ **[O,T]** ✓ Load and begin the script **465TEMPR03**, which captures 1 frame with filter 0 at **48mm** focal length one frame at a time, with a pause command between each frame. CCD: -4.3°C
27. ✓ **[O,T]** ✓ Load and begin the script **465TEMPL03**, which captures 1 frame with filter 0 at **48mm** focal length one frame at a time, with a pause command between each frame. CCD: -5.7°C Then
28. **[O,T]** ✓ Capture **10 images** of the JR dot target in semi-random orientations (see Figure 2) normal to the camera approximately **1-meter** distance covering each edge of the camera's FOV. STARTS AT 9:07 am

* Be explicit — move between images
FIRST TWO PAIRS AT SAME POSITION.

29. **[O,T]** ✓ Capture **20 images** of the JR dot target in semi-random orientations (see Figure 2) normal to the camera approximately **2-meter** distance covering each edge of the camera's FOV. R CCD = −4.5°C, L = −5.7°C
30. **[O,T]** ✓ Capture **20 images** of the JR dot target in semi-random orientations (see Figure 2) normal to the camera approximately **3-meter** distance covering each edge of the camera's FOV. R CCD: −4.6°C L CCD: −5.9°C
31. **[O,T]** ✓ Capture **20 images** of the JR dot target in semi-random orientations (see Figure 2) normal to the camera approximately **4-meter** distance covering each edge of the camera's FOV. R CCD: −4.5°C L CCD: −5.9°C
32. **[O,T]** ✓ Capture **20 images** of the JR dot target in semi-random orientations (see Figure 2) normal to the camera approximately **5-meter** distance covering each edge of the camera's FOV. R CCD: −4.4°C L CCD: −5.8°C
33. **[O,T]** ____ Capture **20 images** of the JR dot target in semi-random orientations (see Figure 2) normal to the camera as distant as possible covering each edge of the camera's FOV. ✗ SKIP. TIME... *

NOT USEFUL. WE HAVE TO FOLLOW A SPECIFIC PROCEDURE

34. **[O,T, L]** ✓ After more than 100 (150 would be perfect) usable frames have been captured, stop the prefix script.
35. **[D]** ✓ Record image names and parameters in Image Log.
36. **[D, L]** Notes: Test end at 11:09 AM; Final CCD Temps: R: −4.5°C L: −5.9°C

– Total ≈ 141 to 143 image pairs at all positions.

* Documentarian needs to work with Technician to correct out-of-sync Left/Right pairs somewhere around/after pair #140...

Date 4/26 Time ~~11:10~~ 11:10 Initials JSP

Data Validation

37. **[V]** ✓ Run the "Geometric_46_Validation" Jupyter notebook on the acquired data for for the Right and Left Mastcam-Zs. This analysis can take place while the test continues.

38. **[V,D, L]** Notes:

Good sampling of FOV in all distances!

ADDED #141 (Right) AND #143 (Left) "Group photo" shot to flush last JR Target image out of The GSE buffer

JIM, Tex, Christian, Andy...

Left camera homed and powered off at 11:25 AM

Date 4/28 Time 11:15 Initials SAB

OK

Fixed Target Positions for the 34mm Right Mastcam-Z (Scene 1)

39. **[M]** N/a If available, install the metrology equipment.
40. **[T]** ✓ Position the JR dot target approximately **1.7 meter** from the camera.
41. **[D]** ✓ Record the following temperatures:
    - Chamber temp ______ Port temp ______
    - Camera CCD temp −4.6°C Optics temp ______
42. **[D,T]** ✓ Take digital pictures of the geometric target's position, and the whole test/GSE set-up. *ADD: SET focal length To 34mm
43. **[O,T]** ✓ Capture test frames to finely position the target to fill the **34mm** FOV and approximately centered in the 26mm FOV. Save these test frames with the prefix name **466TEMPR00**. ADD: Drive focus To 1.7 m
44. **[V,O,T]** ✓ Evaluate whether the target's dots are in-focus enough for discrimination.
45. **[M]** N/A Measure the locations of the geometric target and the camera.
46. **[M,D]** N/a Record the location measurements in the Image Log and tables below.

Target Location	Metrology ID#
Reference	
Top-Left Nest	
Top-Right Nest	
Bottom-Left Nest	
Bottom-Right Nest	

Camera/Chamber Location	Metrology ID#
Reference	
Nest 1	
Nest 2	
Nest 3	

47. **[M,D, L]** Notes: Distance To Target: ~~1.7M~~ 1.43M

NOTE DEFINE: Distance from Front of Bottom of chamber port To Target center.
NOT To cameras.

* NEEDS DIAGRAM

48. **[O,T]** ✓ Load and execute the script **466TEMPR06**, which captures Z-stacks of 16 focus distances (from 1 meter to infinity) for filter 0 with seven focal lengths. The estimated duration is 10 minutes.
49. **[D]** Record image names and parameters in Image Log. ✓
50. **[O,T]** ✓ Load and execute the script **466TEMPR07**, which captures frames at 2-m focus distance for filter 0 with seven focal lengths in reverse. The estimated duration is 3 minutes.
51. **[O,T]** ✓ Load and execute the script **466TEMPR09**, which captures Z-stacks of 16 focus distances (from 1 meter to infinity) for each non-solar filter with the **34mm** focal length. The estimated duration is 10 minutes.
52. **[O,T]** ✓ If time permits, load and execute the script **467TEMPR01**, which auto-exposes and captures frames for ~140 ~~focal~~ lengths with filter 0 at a focus distance of 3 meters. The estimated duration is ~~12~~ 20 minutes.
53. **[D]** ✓ Record image names and parameters in Image Log.
54. **[D, L]** Notes:

STARTED #48 AT 11:32, CCD AT -4.5°C, finished 11:48, CCD -4.0°C
#50 AT 11:50, CCD AT -4.2°C, finished 11:54, CCD=-4.1°C
#51 AT 11:56, CCD AT -4.2°C, finished 12:12, CCD= -3.7°C
#52 AT 12:16, CCD @ -3.9°C, finished 12:25, CCD= ?

Fixed Target Positions for the 63mm Right Mastcam-Z (Scene 2)

55. **[T]** ✓ Position the JR dot target approximately **3 meters** from the camera.
56. **[D]** ✓ Record the following temperatures:
    - Chamber temp ______________ Port temp ______________
    - Camera CCD temp -3.7°C Optics temp ______________
57. **[D,T]** ✓ Take digital pictures of the geometric target's position, and the whole test/GSE set-up.
58. **[O,T]** ✓ Capture test frames to finely position the target to fill the **63mm** FOV and approximately centered in the 34mm FOV. Save these test frames with the prefix name **466TEMPR00**. *ADD: Drive focus to 3 M.
59. **[M]** N/a Measure the locations of the geometric target and the camera.
60. **[M,D]** ✓ Record the location measurements in the Image Log and tables below.

Target Location	Metrology ID#
Reference	
Top-Left Nest	
Top-Right Nest	
Bottom-Left Nest	
Bottom-Right Nest	

Camera/Chamber Location	Metrology ID#
Reference	
Nest 1	
Nest 2	
Nest 3	

61. **[M,D, L]** Notes: ______________________________
DISTANCE TO TARGET: 2.469 M
____

62. **[O,T]** ✓ Load and execute the script **466TEMPR06**, which captures Z-stacks of 16 focus distances (from 1 meter to infinity) for filter 0 with seven focal lengths. The estimated duration is ~~10~~ 16 minutes.
63. **[D]** ✓ Record image names and parameters in Image Log.

64. **[O,T]** ✓ Load and execute the script **466TEMPR10**, which captures Z-stacks of 16 focus distances (from 1 meter to infinity) for each non-solar filter with the **63mm** focal length. The estimated duration is 10 minutes.
65. **[O,T]** ✓ If time permits, load and execute the script **467TEMPR01**, which auto-exposes and captures frames for ~140 focal lengths with filter 0 at a focus distance of 3 meters. The estimated duration is ~~12~~ 20 minutes.
66. **[D]** ____ Record image names and parameters in Image Log.
67. **[D, L]** Notes: Started #62 at 12:52, CCD = -4.3°, Finished: 1:08, CCD: -3.9°C
#64 @ 1:11, CCD = -4.1°C, " 1:29, CCD: -3.7°C
#65 @ 1:33, CCD = -4.0°C, 1:51, CCD: -3.6°C

Fixed Target Positions for the 100mm Right Mastcam-Z (Scene 3)

68. **[T]** ✓ Position the JR dot target approximately **5 meters** from the camera.
69. **[D]** ✓ Record the following temperatures:
    - Chamber temp _______________ Port temp _______________
    - Camera CCD temp _______________ Optics temp _______________
70. **[D,T]** ____ Take digital pictures of the geometric target's position, and the whole test/GSE set-up. * SET 100mm f/L, set focus To ~5m
71. **[O,T]** ____ Capture test frames to finely position the target to fill the **100mm** FOV and approximately centered in the 63mm FOV. Save these test frames with the prefix name * **466TEMP~~L00~~**. R00
72. **[V,O,T]** Evaluate whether the target's dots are in-focus enough for discrimination.
73. **[M]** N/A Measure the locations of the geometric target and the camera.
74. **[M,D]** ✓ Record the location measurements in the Image Log and tables below.

Target Location	Metrology ID#
Reference	
Top-Left Nest	
Top-Right Nest	
Bottom-Left Nest	
Bottom-Right Nest	

Camera/Chamber Location	Metrology ID#
Reference	
Nest 1	
Nest 2	
Nest 3	

75. **[M,D, L]** Notes: Distance = 4.30 m

76. **[O,T]** ____ Load and execute the script **466TEMP~~L11~~** R11, which captures Z-stacks of 16 focus distances (from 1 meter to infinity) for each non-solar filter with the **100mm** focal length. The estimated duration is ~~10~~ 23 minutes.

Date 4/28 Time 2:45 Initials JFD

77. **[D]** ✓ Record image names and parameters in Image Log.
78. **[O,T]** ✓ Load and execute the script **467TEMPL~~04~~** R04, which auto-exposes and captures frames for ~70 focal lengths with filter 0 at a focus distance of 5 meters. The estimated duration is 8 minutes.
79. **[O,T]** ____ If time permits, load and execute the script **466TEMP~~L06~~** R06, which captures Z-stacks of 16 focus distances (from 1 meter to infinity) for filter 0 with seven focal lengths. The estimated duration is 10 minutes.
80. **[D]** ✓ Record image names and parameters in Image Log.
81. **[D, L]** Notes: Started #76 at 1:57pm, CCD: -3.7°C; Finished: 2:20, CCD: -3.7°C
#78 2:22 -3.8°C 2:33 -3.6°C
#79 2:34 -3.6°C

homed and powered down right camera at 2:52 pm

Fixed Target Positions for the 34mm Left Mastcam-Z (Scene 4)

82. **[M]** N/A If available, install the metrology equipment.
83. **[T]** ✓ Position the JR dot target approximately **1.7 meter** from the camera.
84. **[D]** ✓ Record the following temperatures:
    - Chamber temp ______ Port temp ______
    - Camera CCD temp -6.1°C Optics temp ______
85. **[D,T]** ✓ Take digital pictures of the geometric target's position, and the whole test/GSE set-up.
86. **[O,T]** ✓ Capture test frames to finely position the target to fill the **34mm** FOV and approximately centered in the 26mm FOV. Save these test frames with the prefix name **466TEMPL00**.
87. **[V,O,T]** ✓ Evaluate whether the target's dots are in-focus enough for discrimination.
88. **[M]** ✓ Measure the locations of the geometric target and the camera.
89. **[M,D]** ✓ Record the location measurements in the Image Log and tables below.

Target Location	Metrology ID#
Reference	
Top-Left Nest	
Top-Right Nest	
Bottom-Left Nest	
Bottom-Right Nest	

Camera/Chamber Location	Metrology ID#
Reference	
Nest 1	
Nest 2	
Nest 3	

90. **[M,D, L]** Notes: DISTANCE = 1.61 M

91. **[O,T]** ✓ Load and execute the script **466TEMPL06**, which captures Z-stacks of 16 focus distances (from 1 meter to infinity) for filter 0 with seven focal lengths. The estimated duration is 10 minutes.
92. **[D]** Record image names and parameters in Image Log.
93. **[O,T]** ✓ Load and execute the script **466TEMPL07**, which captures frames at 2-m focus distance for filter 0 with seven focal lengths in reverse. The estimated duration is 3 minutes.
94. **[O,T]** ✓ Load and execute the script **466TEMPL09**, which captures Z-stacks of 16 focus distances (from 1 meter to infinity) for each non-solar filter with the **34mm** focal length. The estimated duration is 10 minutes.
95. **[O,T]** ✓ If time permits, load and execute the script **467TEMPL01**, which auto-exposes and captures frames for ~140 focal lengths with filter 0 at a focus distance of 3 meters. The estimated duration is ~~12~~ 20 minutes.
96. **[D]** ✓ Record image names and parameters in Image Log.
97. **[D, L]** Notes: STARTED #91 @ 2:57 CCD@ -5.8°; FINISHED @ 3:12, CCD@ -5.0°C
    #93 3:14 -5.1° 3:18 -5.0°C
    #94 3:19 -5.0° 3:34 -4.7°C
    #95 3:35 -4.7°C 3:54 -4.8°C

Date 4/28 Time 3:57 Initials JAB

Fixed Target Positions for the 63mm Left Mastcam-Z (Scene 5) OK

98. **[T]** ✓ Position the JR dot target approximately **3 meters** from the camera.
99. **[D]** ✓ Record the following temperatures:
    - Chamber temp ______________ Port temp ______________
    - Camera CCD temp -5.2°C Optics temp ______________
100. **[D,T]** ✓ Take digital pictures of the geometric target's position, and the whole test/GSE set-up. *Set f/L to 63mm and focus to 3m
101. **[O,T]** ✓ Capture test frames to finely position the target to fill the **63mm** FOV and approximately centered in the 34mm FOV. Save these test frames with the prefix name **466TEMPL00**.
102. **[M]** N/A Measure the locations of the geometric target and the camera.
103. **[M,D]** ✓ Record the location measurements in the Image Log and tables below.

Target Location	Metrology ID#
Reference	
Top-Left Nest	
Top-Right Nest	
Bottom-Left Nest	
Bottom-Right Nest	

Camera/Chamber Location	Metrology ID#
Reference	
Nest 1	
Nest 2	
Nest 3	

104. **[M,D, L]** Notes: DISTANCE = 2.82M

105. **[O,T]** ✓ Load and execute the script **466TEMPL06**, which captures Z-stacks of 16 focus distances (from 1 meter to infinity) for filter 0 with seven focal lengths. The estimated duration is 10 minutes.
106. **[D]** ✓ Record image names and parameters in Image Log.

Date 4/28 Time 4:20p Initials JFB

107. **[O,T]** ✓ Load and execute the script **466TEMPL10**, which captures Z-stacks of 16 focus distances (from 1 meter to infinity) for each non-solar filter with the **63mm** focal length. The estimated duration is 10 minutes.
108. **[O,T]** ✓ If time permits, load and execute the script **467TEMPL01**, which auto-exposes and captures frames for ~140 focal lengths with filter 0 at a focus distance of 3 meters. The estimated duration is ~~12~~ 20 minutes.
109. **[D]** ✓ Record image names and parameters in Image Log.
110. **[D, L]** Notes: START #105 @ 4:05pm, CCD @ -5.2°; Finished @ 4:21, CCD @ -4.7°
#107 4:23pm -4.8°, 4:39pm -4.7°
#108 4:43pm -5.0° ~~4~~5:02pm -4.7°

Date 4/28 Time 5:10 Initials JH

OK

Fixed Target Positions for the 100mm Left Mastcam-Z (Scene 6)

111. **[T]** ✓ Position the JR dot target approximately **5 meters** from the camera.
112. **[D]** ✓ Record the following temperatures:
    - Chamber temp ______________ Port temp ______________
    - Camera/CCD temp -4.9°C Optics temp ______________
113. **[D,T]** ✓ Take digital pictures of the geometric target's position, and the whole test/GSE set-up. * Set f/l to 100mm and focus to 5 meters
114. **[O,T]** ✓ Capture test frames to finely position the target to fill the **100mm** FOV and approximately centered in the 63mm FOV. Save these test frames with the prefix name **466TEMPL00**.
115. **[V,O,T]** Evaluate whether the target's dots are in-focus enough for discrimination.
116. **[M]** N/A Measure the locations of the geometric target and the camera.
117. **[M,D]** ✓ Record the location measurements in the Image Log and tables below.

Target Location	Metrology ID#
Reference	
Top-Left Nest	
Top-Right Nest	
Bottom-Left Nest	
Bottom-Right Nest	

Camera/Chamber Location	Metrology ID#
Reference	
Nest 1	
Nest 2	
Nest 3	

118. **[M,D, L]** Notes: ______________
DISTANCE = 4.38 M
____

119. **[O,T]** ✓ Load and execute the script **466TEMPL11**, which captures Z-stacks of 16 focus distances (from 1 meter to infinity) for each non-solar filter with the **100mm** focal length. The estimated duration is 10 minutes.

120. **[D]** ✓ Record image names and parameters in Image Log.

121. **[O,T]** ✓ Load and execute the script **467TEMPL04**, which auto-exposes and captures frames for ~70 focal lengths with filter 0 at a focus distance of 5 meters. The estimated duration is 8 minutes.

122. **[O,T]** ✓ If time permits, load and execute the script **466TEMPL06**, which captures Z-stacks of 16 focus distances (from 1 meter to infinity) for filter 0 with seven focal lengths. The estimated duration is ~~10~~ 20 minutes.

123. **[D]** ✓ Record image names and parameters in Image Log.

124. **[D, L]** Notes: start #119 @ 5:22, CCD = -5.1°; Finish @ 5:34, CCD = -4.6°
#121 5:41 -4.8°; 5:52 -4.7°
#122 5:55 -4.8° 6:11 -4.5°

Data Validation

125. **[V]** ____ Run the "Geometric_46_Validation" Jupyter notebook on the acquired data for for the Right and Left Mastcam-Zs. This analysis can take place while the test continues.
126. **[V,D, L]** Notes: ____________________

Time Check 1

NO TIME ON 4/28

X

IF MORE THAN 2.0 HOURS AHEAD OF SCHEDULED END, CONTINUE. OTHERWISE, SKIP TO THE FIXED TARGET TESTS.

Scheduled End Time	Current Time	Time Ahead of Scheduled End
-	=	

127. **[D, L]** ____ Record the time in the table above and determine if there is time for more testing.
128. **[D, L]** Notes: ____________________

Date ______ Time ______ Initials ______

Skip

Fixed Target Positions for the 26mm Right Mastcam-Z (Scene 7)

129. **[T]** ____ Position the JR dot target approximately **1.2 meters** from the camera.
130. **[D]** ____ Record the following temperatures:
    - Chamber temp ______________ Port temp ______________
    - Camera CCD temp ______________ Optics temp ______________
131. **[D,T]** ____ Take digital pictures of the geometric target's position, and the whole test/GSE set-up.
132. **[O,T]** ____ Capture test frames to finely position the target to be filled and centered in the 26mm FOV. Save these test frames with the prefix name **466TEMPR00**.
133. **[V,O,T]** ____ Evaluate whether the target's dots are in-focus enough for discrimination.
134. **[D]** ____ Record the target distance measurement(s) in the Image Log.
135. **[D]** ____ Record image names and parameters in Image Log.
136. **[D, L]** Notes:____

____

____

137. **[M]** ____ Measure the locations of the geometric target and the camera.
138. **[M,D]** ____ Record the location measurements in the Image Log and tables below.

Skip

Target Location	Metrology ID#
Reference	
Top-Left Nest	
Top-Right Nest	
Bottom-Left Nest	
Bottom-Right Nest	

Camera/Chamber Location	Metrology ID#
Reference	
Nest 1	
Nest 2	
Nest 3	

139. **[M,D, L]** Notes: ____
____
____

140. **[O,T]** ____ Load and execute the script **466TEMPR13**, which captures Z-stacks of 16 focus distances (from 1 meter to infinity) for each non-solar filter with the **26mm** focal length. The estimated duration is 10 minutes.
141. **[D]** Record image names and parameters in Image Log.
142. **[O,T]** ____ If time permits, load and execute the script **466TEMPR06**, which captures Z-stacks of 16 focus distances (from 1 meter to infinity) for filter 0 with seven focal lengths. The estimated duration is 10 minutes.
143. **[D]** Record image names and parameters in Image Log.
144. **[D, L]** Notes:____
____
____

Fixed Target Positions for the 26mm Left Mastcam-Z (Scene 8)

Skip

145. **[T]** ____ Position the JR dot target approximately **1.2 meters** from the camera.
146. **[D]** ____ Record the following temperatures:
    - Chamber temp ______________ Port temp ______________
    - Camera CCD temp ______________ Optics temp ______________
147. **[D,T]** ____ Take digital pictures of the geometric target's position, and the whole test/GSE set-up.
148. **[O,T]** ____ Capture test frames to finely position the target to be filled and centered in the 26mm FOV. Save these test frames with the prefix name **466TEMPR00**.
149. **[V,O,T]** ____ Evaluate whether the target's dots are in-focus enough for discrimination.
150. **[D]** ____ Record the target distance measurement(s) in the Image Log.
151. **[D]** ____ Record image names and parameters in Image Log.
152. **[D, L]** Notes:____

    ____

    ____

153. **[M]** ____ Measure the locations of the geometric target and the camera.
154. **[M,D]** ____ Record the location measurements in the Image Log and tables below.

Date ______ Time ______ Initials ______

Skip

Target Location	Metrology ID#
Reference	
Top-Left Nest	
Top-Right Nest	
Bottom-Left Nest	
Bottom-Right Nest	

Camera/Chamber Location	Metrology ID#
Reference	
Nest 1	
Nest 2	
Nest 3	

155. **[M,D, L]** Notes: ____

____

____

156. **[O,T]** ____ Load and execute the script **466TEMPR13**, which captures Z-stacks of 16 focus distances (from 1 meter to infinity) for each non-solar filter with the **26mm** focal length. The estimated duration is 10 minutes.
157. **[D]** Record image names and parameters in Image Log.
158. **[O,T]** ____ If time permits, load and execute the script **466TEMPR06**, which captures Z-stacks of 16 focus distances (from 1 meter to infinity) for filter 0 with seven focal lengths. The estimated duration is 10 minutes.
159. **[D]** Record image names and parameters in Image Log.
160. **[D, L]** Notes: ____

____

____

Time Check 2 NO TIME ON 4/28

IF MORE THAN 1.0 HOUR AHEAD OF SCHEDULED END, CONTINUE.

OTHERWISE, SKIP TO THE SHUTDOWN PROCEDURE.

Scheduled End Time	Current Time	Time Ahead of Scheduled End
-	=	

161. **[D, L]** ____ Record the time in the table above and determine if there is time for more testing

162. **[D, L]** Notes: ____

____

____

100+ Target Positions for the 63mm Right and Left Mastcam-Zs Skip

163. **[T]** ____ Position the JR dot target approximately **3 meters** from the camera. Adjust lights accordingly.
164. **[D]** ____ Record the following temperatures:
    - Chamber temp ______________ Port temp ______________
    - Camera CCD temp ______________ Optics temp ______________
165. **[D,T]** ____ Take digital pictures of the geometric target's position, and the whole test/GSE set-up.
166. **[O,T]** ____ Capture test frames to find a standard exposure time for the 100 positions at 3 meters focus. Save these test frames with the prefix name **465TEMPR00**, and update "var1" in the script **465TEMPR04** once this exposure time is found.
167. **[V,O,T]** Evaluate whether the target's dots are in-focus enough for discrimination. If the dots are too out-of-focus for JR's algorithm, move the target back.
168. **[O,T]** ____ Capture test frames to find a standard exposure time for the 100 positions at 3 meters focus. Save these test frames with the prefix name **465TEMPL00**, and update "var1" in the script **465TEMPL04** once this exposure time is found.
169. **[V,O,T]** Evaluate whether the target's dots are in-focus enough for discrimination. If the dots are too out-of-focus for JR's algorithm, move the target back.
170. **[O,T]** ____ Load and begin the script **465TEMPR04**, which captures frames with filter 0 at **63mm** focal length one frame at a time, with a pause command between each frame.
171. **[O,T]** ____ Load and begin the script **465TEMPL04**, which captures frames with filter 0 at **63mm** focal length one frame at a time, with a pause command between each frame.
172. **[O,T]** ____ Capture **10 images** of the JR dot target in semi-random orientations (see Figure 2) normal to the camera approximately **1-meter** distance covering each edge of the camera's FOV.

Skip

173. **[O,T]** ____ Capture **20 images** of the JR dot target in semi-random orientations (see Figure 2) normal to the camera approximately **2-meter** distance covering each edge of the camera's FOV.
174. **[O,T]** ____ Capture **20 images** of the JR dot target in semi-random orientations (see Figure 2) normal to the camera approximately **3-meter** distance covering each edge of the camera's FOV.
175. **[O,T]** ____ Capture **20 images** of the JR dot target in semi-random orientations (see Figure 2) normal to the camera approximately **4-meter** distance covering each edge of the camera's FOV.
176. **[O,T]** ____ Capture **20 images** of the JR dot target in semi-random orientations (see Figure 2) normal to the camera approximately **5-meter** distance covering each edge of the camera's FOV.
177. **[O,T]** ____ Capture **20 images** of the JR dot target in semi-random orientations (see Figure 2) normal to the camera as distant as possible covering each edge of the camera's FOV.
178. **[O,T, L]** ____ After more than 100 usable frames have been captured, stop the prefix script.
179. **[D]** ____ Record image names and parameters in Image Log.
180. **[D, L]** Notes:____

____

____

Data Validation

181. **[V]** ____ Run the "Geometric_46_Validation" Jupyter notebook on the acquired data for the Right and Left Mastcam-Zs. This analysis can take place while the test continues.
182. **[V,D, L]** Notes: ____

    ____

    ____

Shutdown Procedure

183. **[D,T]** ____ Take digital pictures of this page and the test setup.
184. **[D,O]** ____ Review entries in Image Log, GSE command log, and image headers.
185. **[D, L]** ____ Review calibration procedure and ensure that each task is initialed.
186. **[D, L]** Notes: ____

____

____

187. **[V, L]** ____ Before making the decision to break down the test setup, ensure that adequate data were acquired for the test requirements. See "MastcamZCalPlan" for these requirements.
188. **[V]** Notes: ____

____

____

Data Validator (signature) [signature]

Date 4/28/19 Time 8:16 PM

189. **[V, L]** ____ Give the go/no-go decision. Have enough data been acquired to fulfill test requirements? See "MastcamZCalPlan" for these requirements.
190. **[D, L]** ____ Update the Log Document.
191. **[L]** Notes: ____

____

____

Calibration Lead (signature) [signature]

Date 4/28/19 Time 6:00 pm

192. **[O, L]** ____ Ensure that the camera and GSE are in a safe state.
193. **[O,D]** ____ Review the Image Log with the documentarian. Exchange high-fives.
194. **[O]** Notes: ____

____

____

Camera Operator (signature) ______________________

Date ______________ Time ______________

195. **[T]** ____ If the next test does not require the target, position it away from the chamber or bench. Otherwise, be sure not to move it. The next test is ________________.
196. **[T]** ____ Ensure that all other test equipment is safely put away.
197. **[T]** Notes: ____

____

____

Technician (signature) ______________________

Date ______________ Time ______________

198. **[D, L]** ____ Double-check this procedure and ensure that the top of each page has valid data, time and initials.
199. **[D]** ____ Photo-scan this document, save it on the cloud, and file the hard-copy in the Log Binder. Upload the digital pictures taken during this test in the appropriate archive on the cloud. The required links are on the Wiki.
200. **[D]** ____ Double-check that every required cell the Image Log is accurately filled. When this is complete, print the Image Log and file it the Log Binder after this document.
201. **[D]** Notes: ____

____

____

Documentarian (signature) ______________________

Date ______________ Time ______________

Solar Radiometric Calibration Procedure for Mastcam-Z TVAC Testing at MSSS (Pro. 4.2.6)

[Procedure version 2.03, prepared by the Mastcam-Z calibration team at Cornell University]

These measurements are performed on the camera and at the temperature designated below as specified in the Mastcam-Z Calibration Plan,

Unit Under Test:

Left FM __X__ Right FM __X__ EQM____ Other________

These measurements are performed at temperature:

-35° C____ -10°C__X__ +5°C____ Ambient____ Other________

These measurements are performed at,

MSSS__X__ ASU____ Other______

Date 4/28/2019 **Start Time** 6:15 pm **End Time** ______

Estimated Duration **1.0 hour**

Scheduled Start Time — **Sch. End Time** —

**Calibration Lead [L]** Jim	**Documentarian [D]** Megan
**Camera Operator [O]** Tex/Kathryn	**Technician [T]** Christian, Andy
**Data Validator [V]** Paul	**Other** ______

Change Log

Version	Name	Change
v1_01<br>17 Sep 2018	C. Tate	(first draft)
v1_20<br>1 Nov 2018	C. Tate	Procedure edits prior to EQM testing
v1_23<br>10 Dec. 2018	C. Tate	Procedure edits after EQM testing
v2_03<br>28 April 2019	C. Tate	Approved version prior to FM testing

Document Approval

Approved by James Bell Date
Mastcam-Z PI
Arizona State University

Approved by Alexander Hayes Date
Mastcam-Z Calibration Working Group
Lead, Cornell University

Approved by Justin Maki Date
Mastcam-Z Deputy PI and Investigation
Scientist, Jet Propulsion Laboratory

[signature] 4/28
Approved by Christian Tate Date
Procedure Author
Cornell University

Approved by Date

Table of Contents

SOLAR RADIOMETRIC CALIBRATION PROCEDURE FOR MASTCAM-Z TVAC TESTING AT MSSS (PRO. 4.2.6)............ 1

CHANGE LOG.. 2
DOCUMENT APPROVAL 2
TEST DESCRIPTION.. 4
SOFTWARE PREPARATION 4
Table 1. File naming convention for the camera script prefixes and frame filenames: "AAABBBBCDD"............ 4
HARDWARE INSTALLATION 6
Figure 1. ASU Floor Plan for Geometric Testing in the TVAC Chamber. The MSSS Floor Plan allows for similar target and source placements relative to the chamber window. 6
Table 2. The Nominal Radiance Values (calibrated integrating sphere output). 8

RIGHT AND LEFT MASTCAM-Z TESTS 9

CENTER THE INTEGRATING SPHERE... 9
RADIANCE VALUE 1 FOR THE RIGHT AND LEFT MASTCAM-ZS 10
RADIANCE VALUE 2 FOR THE RIGHT AND LEFT MASTCAM-ZS 11
DATA VALIDATION.. 12

SHUTDOWN PROCEDURE 13

Test Description

Excerpt from the Calibration Plan 4.2,

> The objectives of these tests are to derive flat field images as well as the coefficients to allow a conversion from reduced (bias, dark, and flat field corrected) DN/s to absolute radiometric response ($W/cm^2/sr$ per filter) for (a) the R, G, and B microfilters of the Bayer Pattern Filter detectors in each camera head (clear filter), (b) the 14 non-solar Mastcam-Z spectral filters "Science Filters", and, if time permits, (c) the two Mastcam-Z neutral density solar filters; and to provide an estimate of the uncertainty in these coefficients and, at Priority 2, their temperature dependence. This test builds off the Section 4.3 – Spectral Throughput Calibration to accurately account for the filter spectral response in the conversion. The requirement of knowing the relative response on the shape of the spectral throughput to ±5% combined with the absolute Radiance accuracy of the integration sphere at ±5% still allows the ±10% absolute radiometric calibration requirement to be met.

Software Preparation

The software and files required for this test are prepared well in advance of test day. This checklist ensures that the following are present, debugged, and executable: (1) all fast-look scripts, (2) automated header generation of all relevant camera parameters, target positioning, and metadata, (3) all camera scripts that command the camera unit, and (4) the directories/file-paths pointing to the data repositories of this specific test.

Table 1. File naming convention for the camera script prefixes and frame filenames: "AAABBBBCDD"

Code	Name	Example	Value
"AAA"	Calibration Plan Section	"411" = Cal. Plan 4.1.1 chapter 4, section 1, subsection 1	426
"BBBB"	Location of test or ASU Chamber temperature	"MSSS" = test at MSSS, "TN10" = ASU TVAC -10C, …	TN10
"C"	Camera unit under test	"L" = Left Mastcam-Z, "R" = Right Mastcam-Z, "E" =EQM, "C" =COTS	R/L
"DD"	Part of test (radiance value)	"00" = test set up, "01" = first radiance value …	00-02

1. **[D]** N/a Look up the daily calibration schedule and record the scheduled start and end time of this test on the cover page of this document. Also fill out and double-check the other information on the cover page.
2. **[D]** ✓ Ensure that all supplemental manuals are on hand. These are,
   - Labsphere_Manual,
   - Validator_Manual, Documentarian_Manual
   - MastcamZCalPlan
3. **[D]** ✓ Ensure that the Image Log is present and ready to use. Find and open the Google Sheets file "Image_Log_42". There is a link on the Wiki.
4. **[V]** ✓ Check that all Calgorithms fast-look and validation scripts are present, up-to-date, and ready to analyze test output. Find and open the "Radiometric_Calibration_42_Validation" Jupyter notebook. There is a link on the Wiki.
5. **[O]** ✓ Check that all camera scripts required for this test are present, up-to-date and ready to command the ground support equipment (GSE). These are,
   - **426TN10R00 - 426TN10R06**
   - **426TN10L00 - 426TN10L06**
6. **[O,V,D, L]** Notes:

___

___

___

Hardware Installation

This procedure is for the ambient TVAC chamber testing at MSSS. Figure 1 shows the nominal layout of the TVAC chamber, workspace, Mastcam-Zs, ground support equipment (GSE), targets, sources, and other equipment necessary for this test if it happens at ASU. Although MSSS' cleanroom is different than ASU's, the placement of the targets and sources relative to the chamber window is similar.

Figure 1. ASU Floor Plan for Geometric Testing in the TVAC Chamber. The MSSS Floor Plan allows for similar target and source placements relative to the chamber window.

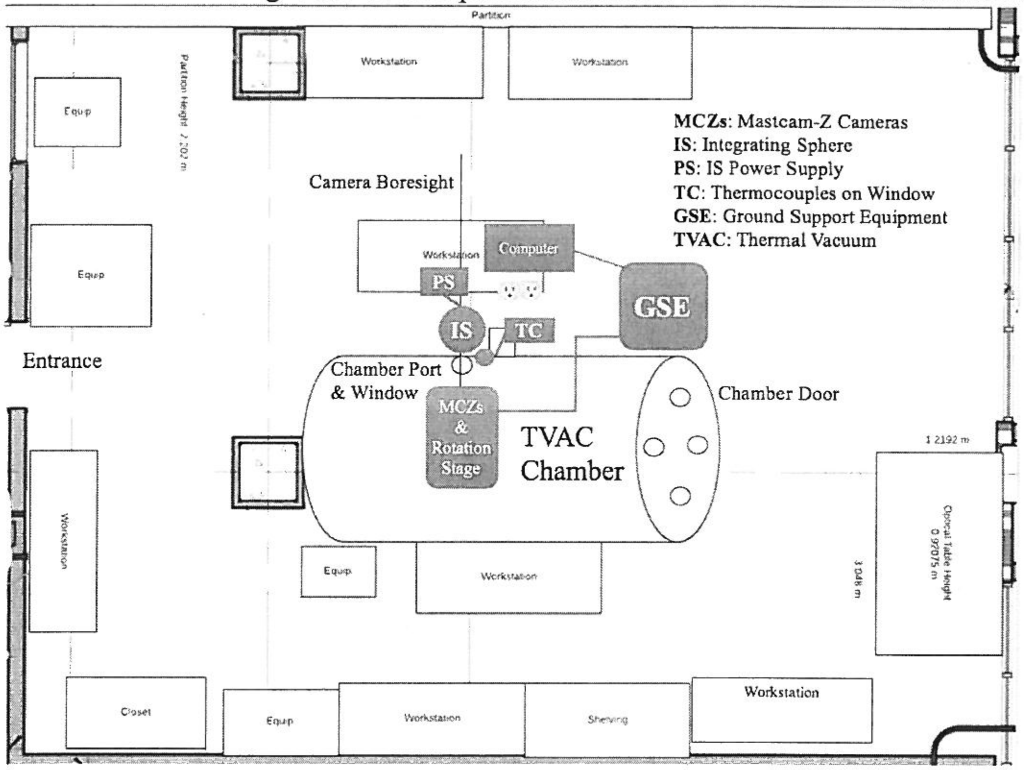

7. **[T,O, L]** ✓ Ensure that all personnel in the cleanroom are following the cleanroom practices for electrostatic discharge, proper clothing and other safety concerns.
8. **[T]** ✓ Double check that nitrogen is flowing over the Mastcam-Zs or the window port.
9. **[O,T]** ✓ If not already done, mate the Right and Left Mastcam-Zs into the GSE. Follow the procedure in "MastcamZ_GSE_Manual".
10. **[T]** ✓ Verify that the thermocouples are turned on and properly reading out.

11. **[O,T]** ✓ Ensure that the camera unit and GSE wires are secure, kink-free, and do not present tripping hazards when the lights are turned off.
12. **[O,D]** ✓ Check the camera temperature and ensure nominal operation.
13. **[D]** n/a Record the following environmental information:
    - Cleanroom temperature _______ pressure _______ humidity _______
14. **[O,D, L]** Notes:

____

____

____

15. **[D,T]** ____ Take time-stamped pictures of this page, the integrating sphere, and the whole test/GSE set-up.
16. **[T]** ✓ Power on the integrating sphere. Follow the procedure in "Labsphere_Manual". Record the time the lamp is turned on 5:30pm.
17. **[D,T]** ____ Record the exact readout value of the integrating sphere's radiance: 9.72 [illegible] mW/cm2/sr. INITIAL
18. **[T,O, L]** ✓ Confirm that the camera systems and GSEs are powered on and ready for use. Follow the procedure in "MastcamZ_GSE_Manual".
19. **[D, L]** Notes:

____

____

____

Table 2. The Nominal Radiance Values (calibrated integrating sphere output).

**IS Output Radiances**	**Nominal Radiance [mW/cm2/sr]**
Radiance 1	60.0
Radiance 2	80.0

Right and Left Mastcam-Z Tests

FILL FOV @ 100 MM

Center the Integrating Sphere

~0.3M

20. **[T]** ✓ Move integrating sphere output as close to the chamber window as possible centered on the Right Mastcam-Z boresight.
21. **[O]** ✓ Capture test image at 100mm with both cameras with filter 0. Insert the note "ISOP=[radiance]" and call the images **426TN10R00** and **426TN10L00**.
22. **[V,O,T]** ✓ Open images, and if the images show that the integrating sphere is not centered, center the integrating sphere disc in both frames. Recapture **426TN10R00** and **426TN10L00** frames if necessary.
23. **[D]** ✓ Record image names and parameters in the image Log.
24. **[T]** N/A Lights off
25. **[D, L]** Notes:

focus at ∞

set sphere at 10 mW level (do not illuminate cameras with full lamp)

Distance to window 18.5 cm for first test image

" 12 cm 2nd

" 4.5 cm 3rd...

12 cm Final

Testing 1, 10, ~~[illegible]~~ 60 sec. Levels: 400 DN or so... OK!

Date 4/28 Time 6:45 Initial JFB

Radiance Value 1 for the Right and Left Mastcam-Zs

26. **[T]** ✓ Set integrating sphere output to the radiance value 1 defined in Table 2.
27. **[D,T]** ✓ Record exact integrating sphere readout value 78.622 mW/cm2/sr.
28. **[D]** ____ Record temperature information:
    - Chamber temp ________ Port temp ________
    - Camera CCD temp (R) − 3.6° ~~Optics~~ CCD temp (L) = −5.1°C
29. **[D,T]** ✓ Take time-stamped digital pictures of the setup and integrating sphere readout.
30. **[O]** ✓ Insert the note "ISOP=[radiance]" and execute camera script **426TN10R02**, which captures 5 frames at approximately 10% and 20% full-well and 5 bias frames with the solar filter at 100mm focal length and focus at infinity. The estimated duration is 18 minutes.
31. **[O]** ____ Insert the note "ISOP=[radiance]" and execute camera script **426TN10L02**, which captures 5 frames at approximately 10% and 20% full-well and 5 bias frames with the solar filter at 100mm focal length and focus at infinity. The estimated duration is 8 minutes.
32. **[D,T]** ____ Record exact integrating sphere readout value ________ mW/cm2/sr.
33. **[D]** ____ Record image names and parameters in the Image Log.
34. **[D, L]** Notes: 76.383 sphere level for step #30 (end: ?)
    79.250 " for step #31

Right
→ 10 x 60 sec exposures in R8 ~400 DNs or so
~~[illegible]~~ 5 x 0 sec bias frames at end
then turn off one extra bulb (#1)
Then 5 x 60 sec exposures at 50% flux level. (44.474)

Left
then 10x60 sec exposures in L8 (79.075 sphere) ~250 DNs or so
5x 0 sec bias frames (CCD @ −5.1°C)
Then 5x60 sec @ 50% Lamp = 47.041 sphere, 47.066 last (78.651 @ end sphere)

Then 60 sec darks (lights off) x 3 + bias x 5
for both cameras
Temps: L = −5.3°C R = −3.7°C

End @ 7:55 pm

Radiance Value 2 for the Right and Left Mastcam-Zs

35. **[T]** ____ Set integrating sphere output to the radiance value 1 defined in Table 2.
36. **[D,T]** ____ Record exact integrating sphere readout value __________ mW/cm2/sr.
37. **[D]** ____ Record temperature information:
    - Chamber temp ________________ Port temp ______________
    - Camera CCD temp ______________ Optics temp ______________
38. **[D,T]** ____ Take time-stamped digital pictures of the setup and integrating sphere readout.
39. **[O]** ____ Insert the note "ISOP=[radiance]" and execute camera script **426TN10R02**, which captures 5 frames at approximately 10% and 20% full-well and 5 bias frames with the solar filter at 100mm focal length and focus at infinity. The estimated duration is 18 minutes.
40. **[O]** ____ Insert the note "ISOP=[radiance]" and execute camera script **426TN10L02**, which captures 5 frames at approximately 10% and 20% full-well and 5 bias frames with the solar filter at 100mm focal length and focus at infinity. The estimated duration is 8 minutes.
41. **[D,T]** ____ Record exact integrating sphere readout value __________ mW/cm2/sr.
42. **[D]** ____ Record image names and parameters in the Image Log.
43. **[D, L]** Notes: ____

____

____

Data Validation

44. **[T]** ✓ Lights on
45. **[V]** ✓ Upload data to server.
46. **[V]** N/A Run the "Radiometric_Calibration_42_Validation" Jupyter notebook on the acquired data for the Right Mastcam-Z with the window off. This analysis can take place while the test continues.
    - Create preliminary flat-field images and radiometric coefficients for each filter.
    - Save results in the calibration records.
47. **[V,D, L]** Notes: ____

____

____

Shutdown Procedure

48. **[D,T]** ____ Take digital pictures of this page and the test setup.
49. **[D,O]** ____ Review entries in Image Log, GSE command log, and image headers.
50. **[D, L]** ____ Review calibration procedure and ensure that each task is initialed.
51. **[D, L]** Notes: ____

52. **[V, L]** ____ Before making the decision to break down the test setup, ensure that adequate data were acquired for the test requirements. See "MastcamZCalPlan" for these requirements.
53. **[V]** Notes: ____

Data Validator (signature) [signature]

Date 4/28/19 Time 8:10

54. **[V, L]** ____ Give the go/no-go decision. Have enough data been acquired to fulfill test requirements? See "MastcamZCalPlan" for these requirements.
55. **[D, L]** ____ Update the Log Document.
56. **[L]** Notes: ____

Calibration Lead (signature) [signature]

Date 4/28/19 Time 8pm

57. **[O, L]** ____ Ensure that the camera and GSE are in a safe state.
58. **[O,D]** ____ Review the Image Log with the documentarian. Exchange high-fives.
59. **[O]** Notes: ____________________

Camera Operator (signature) ____________________

Date ____________ Time ____________

60. **[T]** ____ If the next test does not require the integrating sphere, position it away from the chamber or bench. Otherwise, be sure not to move it. The next test is __________.
61. **[T]** ____ Ensure that all other test equipment is safely put away.
62. **[T]** Notes: ____________________

Technician (signature) Christian Tate

Date April 29, 2019 Time 8:38 am

63. **[D, L]** ____ Double-check this procedure and ensure that the top of each page is initialed with the time and date.
64. **[D]** ____ Photo-scan this document, save it on the cloud, and file the hardcopy in the Log Binder. Upload the digital pictures taken during this test in the appropriate archive on the cloud. The required links are on the Wiki.
65. **[D]** ____ Double-check that every required cell the Image Log is accurately filled. When this is complete, print the Image Log and file it the Log Binder after this document.
66. **[D]** Notes: ____________________

Documentarian (signature) ____________________

Date ____________ Time ____________

JR Geometric Calibration Procedure for the Right and Left Mastcam-Z TVAC Ramping at MSSS (Pro. 4.6.8)

[Procedure version 2.04, prepared by the Mastcam-Z calibration team at Cornell University]

These measurements are performed on the camera and at the temperature designated below as specified in the Mastcam-Z Calibration Plan,

Unit Under Test:

Left FM __X__ Right FM __X__ EQM_____ Other____________

These measurements are performed at temperature:

-35°C______ -10°C_______ +5°C______ Ambient______ Other_-10 to +35 ramp

These measurements are performed at,

MSSS__X__ ASU_______ Other________

Date 4-29-2019 **Start Time** 10:05 **End Time** ____________

Estimated Duration _3.0 hours_

Scheduled Start Time 9:00 **Sch. End Time**__________

Calibration Lead [L] Justin Maki **Documentarian [D]** Christian Tate

Camera Operator [O] Tex & Elsa **Technician [T]** Andy Windholtd

Data Validator [V] Paul Corlies **Metrologist [M]** ____________

Other ____________________

Change Log

Version	Name	Change
v1_01<br>26 Sep 2018	C. Tate	(first draft)
v1_07<br>1 Nov 2018	C. Tate	Procedure edits prior to EQM testing
V1_07-JR<br>8 Nov 2018	G. Paar	Distances more precisely reflected, change mode from v06 to v07 kept, fixed focus consistently at 2 tables & figure automatically referenced
v1_10<br>13 Dec. 2018	C. Tate	Procedure edits after EQM testing
v2_04<br>29 April 2019	C. Tate	Approved version prior to FM testing

Document Approval

X

Approved by James Bell Date
Mastcam-Z PI
Arizona State University

Approved by Alexander Hayes Date
Mastcam-Z Calibration Working Group
Lead, Cornell University

x [signature] 4/30/19

Approved by Justin Maki Date
Mastcam-Z Deputy PI and Investigation
Scientist, Jet Propulsion Laboratory

[signature]

Approved by Christian Tate Date
Procedure Author
Cornell University

Approved by: Date

Table of Contents

JR GEOMETRIC CALIBRATION PROCEDURE FOR THE RIGHT AND LEFT MASTCAM-Z TVAC RAMPING AT MSSS (PRO. 4.6.8) 1

CHANGE LOG 2
DOCUMENT APPROVAL 2
TEST DESCRIPTION 4
SOFTWARE PREPARATION 4
Table 1. File naming convention for the camera script prefixes and frame filenames: "AAABBBBCDD" 4
HARDWARE INSTALLATION 6
Figure 1. ASU Floor Plan for Geometric Testing in the TVAC Chamber. The MSSS Floor Plan allows for similar target and source placements relative to the chamber window. 6
TARGET METROLOGY 8
TEST SCRIPTS THE RIGHT AND LEFT MASTCAM-ZS 9
TEMP AT RIGHT AND LEFT MASTCAM-ZS 10
TEMP AT RIGHT AND LEFT MASTCAM-ZS 11
TEMP AT RIGHT AND LEFT MASTCAM-ZS 12
TEMP AT RIGHT AND LEFT MASTCAM-ZS 13
TEMP AT RIGHT AND LEFT MASTCAM-ZS 14
TEMP AT RIGHT AND LEFT MASTCAM-ZS 15
TEMP AT RIGHT AND LEFT MASTCAM-ZS 16
TEMP AT RIGHT AND LEFT MASTCAM-ZS 17
TEMP AT RIGHT AND LEFT MASTCAM-ZS 18
TEMP AT RIGHT AND LEFT MASTCAM-ZS 19
TEMP AT RIGHT AND LEFT MASTCAM-ZS 20
TEMP AT RIGHT AND LEFT MASTCAM-ZS 21
TEMP AT RIGHT AND LEFT MASTCAM-ZS 22
TEMP AT RIGHT AND LEFT MASTCAM-ZS 23
TEMP AT RIGHT AND LEFT MASTCAM-ZS 24
TEMP AT RIGHT AND LEFT MASTCAM-ZS 25
TEMP AT RIGHT AND LEFT MASTCAM-ZS 26
TEMP AT RIGHT AND LEFT MASTCAM-ZS 27
TEMP AT RIGHT AND LEFT MASTCAM-ZS 28
TEMP AT RIGHT AND LEFT MASTCAM-ZS 29
TEMP AT RIGHT AND LEFT MASTCAM-ZS 30
TEMP AT RIGHT AND LEFT MASTCAM-ZS 31
TEMP AT RIGHT AND LEFT MASTCAM-ZS 32
DATA VALIDATION 33
DARK CURRENT WITH THE RIGHT AND LEFT MASTCAM-ZS 34
DATA VALIDATION 35

SHUTDOWN PROCEDURE 36

Test Description

Excerpt from the Calibration Plan 4.6

> The objective of Geometric Calibration is to characterize the geometric distortion introduced by the Mastcam-Z optics into its images, and measure the effective focal length and field of view at each focus and zoom position. As the range of zoom positions available to Mastcam-Z represent a continuum, measurements will be acquired at a finite number of zoom settings and then interpolated to characterize distortion and other geometric parameters across the full zoom range. Targets should be imaged at ~50% full well using the Bayer RGB/805 nm (priority 1) and remaining non-solar filters (priority 3). The calibration data will be used to generate a geometric model for each camera. The camera models may exhibit wavelength dependence, so an attempt to measure the effect overall filters is desired (although not required).

Software Preparation

The software and files required for this test are prepared in advance of test day. This checklist ensures that the following are present, debugged, and executable: (1) all fast look scripts, (2) automated header generation of all relevant camera parameters, target positioning, and metadata, (3) all camera scripts that command the camera unit, and (4) the directories/file-paths pointing to the data repositories of this specific test.

Table 1. File naming convention for the camera script prefixes and frame filenames: "AAABBBBCDD"

Code	Name	Example	Value(s)
"AAA"	Calibration Plan Section	"465" = Cal. Plan 4.6.5 chapter 4, section 6, subsection 5	468, 473, 441
"BBBB"	Location of test or ASU Chamber temperature	"ATLO" = test at JPL ATLO, "TN10" = MSSS TVAC -10C, …	TEMP
"C"	Camera unit under test	"L" = Left Mastcam-Z, "R" = Right Mastcam-Z, "E" =EQ "C" =COTS	L/R
"DD"	Part of test	"00" = test set up, "01" = first part,…	00-13

1. **[D]** CO Look up the daily calibration schedule and record the scheduled start and end time of this test on the cover page of this document. Also, fill out and double-check the other information on the cover page.
2. **[D]** CO Ensure that all supplemental manuals are on hand. These are,
   - Validator_Manual, Documentarian_Manual, MastcamZ_Data_Manual,
   - MastcamZCalPlan
3. **[D]** CO Ensure that the Image Log is present and ready to use. Find and open the Google Sheets file "Image_Log_46". There is a link on the Wiki.
4. **[V]** CO Check that all *Calgorithms* fast-look and validation scripts are present, up-to-date, and ready to analyze test output. Find and open the "Geometric_Calibration_46_Validation" Jupyter notebook. There is a link on the Wiki.
5. **[O]** CO Check that all camera scripts required for this test are present, up-to-date and ready to command the ground support equipment (GSE). These are,
   - **473TEMPR01 - 473TEMPR13**
   - **473TEMPL01 - 473TEMPL13**
   - **441TEMPR03 and 441TEMPL03**

6. **[O,V,D, L]** Notes:

____

____

____

Hardware Installation

This procedure is for the ambient TVAC chamber testing at MSSS. Figure 1 shows the nominal layout of the TVAC chamber, workspace, Mastcam-Zs, ground support equipment (GSE), targets, sources, and other equipment necessary for this test if it happens at ASU. Although MSSS' cleanroom is different than ASU's, the placement of the targets and sources relative to the chamber window is similar.

Figure 1. ASU Floor Plan for Geometric Testing in the TVAC Chamber. The MSSS Floor Plan allows for similar target and source placements relative to the chamber window.

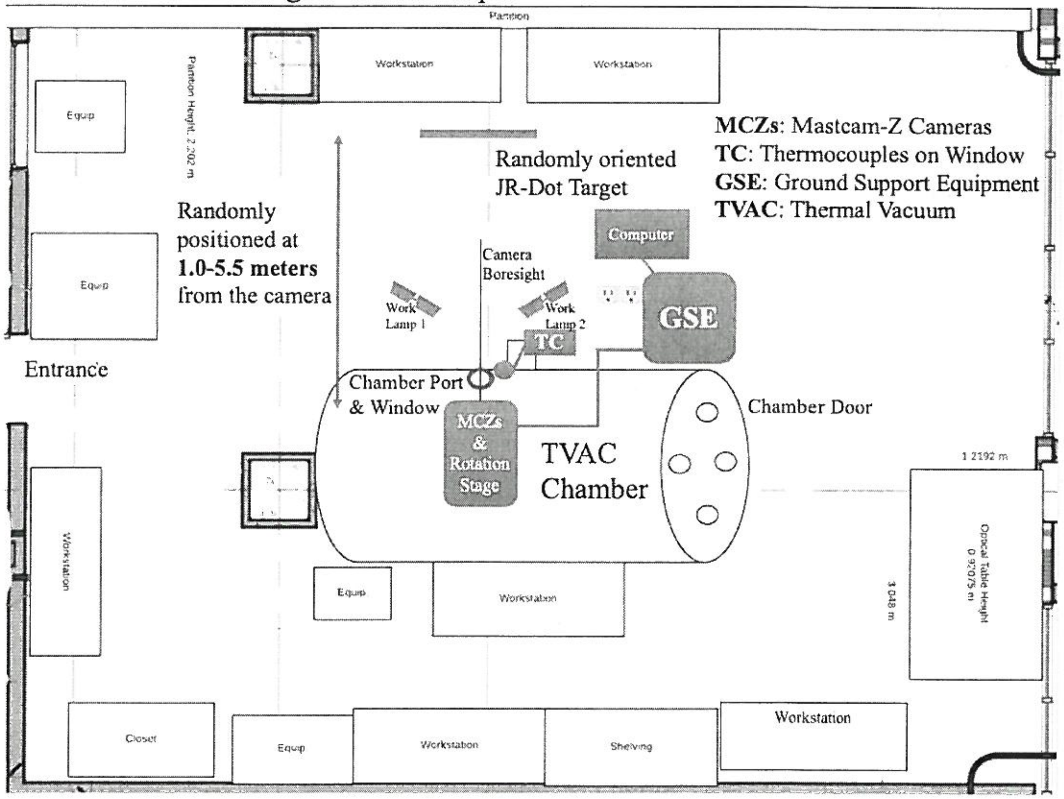

7. **[T,O, L]** ~~___~~ Ensure that all personnel in the cleanroom are following the cleanroom practices for electrostatic discharge, proper clothing, and other safety concerns. See "ESD_Manual" and "Cleanroom_Manual".
8. **[T]** ____ Double check ~~that nitrogen~~ air ionizer on is flowing over the Mastcam-Zs or the window port.
9. **[O,T]** ____ If ~~not already~~ done, mate the Right Mastcam-Z into the GSE. Follow the procedure in "MastcamZ_GSE_Manual".
10. **[T]** ____ Verify that the thermocouples are turned on and properly reading out.

MCZ CH PRT Temp: Right -5.3°C Left -6.4°C

TVAC Chamber: Aux. Plat -10.81°C Main +5.49°C Shroud = -11.92 Cold = -143.68°C

Date 4/29 Time 10:45 AM Initials JM

11. **[T]** ____ Position the JR dot target approximately **3 meters** from the cameras 2.07 bar
12. **[T]** ____ Install the lamps and ~~position them about 1 meter from the geometric target out of the camera's field of view (FOV).~~ Power them on. no lamps
13. **[O,T]** ____ Ensure that the camera unit and GSE wires are secure, kink-free, and do not present tripping hazards when the lights are turned off. ✓ ok
14. **[O,D]** ____ Check the camera temperature and ensure nominal operation.
15. **[D]** ____ Record the following environmental information:
    - Cleanroom temperature 22.8°C pressure earth ambient humidity ~~38%~~ 37% RH
16. **[O,D, L]** Notes:

17. **[O,T, L]** JM Capture a test frame with both cameras at 34mm focused at 3 meters with filter 0 to center the target in both cameras. Save images with prefixes **468TEMPR00** and **468TEMPL00**. 4 test images acquired
18. **[D, L]** Notes: positioned target for optimal dot coverages.

10:40 AM "Fog" seen in (auto focus images)
10:45 AM Caplinger wiped the window
10:57 AM ramp started
11:02 AM CH temp: L: -4.6°C
R: -3.7°C

Aux. platen: -3.1°C
main 5.49°
shroud -10.81°C
cold plate -143.83°C

Suffixes: 468TEMPR/L
0-4
Suffices: 473TEMPR/L
0-15

Target ~~Metrology~~ setup

19. **[T]** ____ Once the target is centered at approximately ~~3~~ 2.07 meters **meters** from both cameras, use lidar to measure the target position. ✓
20. **[D]** ____ Record the following temperatures:
    - Chamber temp ______________ Port temp ______________
    - Camera CCD temp ______________ Optics temp ______________
21. **[D,T]** ✓ Take digital pictures of the geometric target's position, and the whole test/GSE set-up. Andy acquired: 2.07 meters from
22. **[M]** ____ Measure the locations of the geometric target and the camera.
23. **[M,D]** ____ Record the location measurements in the Image Log and tables below.

Target Location	~~Metrology ID#~~
Reference	
Top-Left Nest	
Top-Right Nest	
Bottom-Left Nest	
Bottom-Right Nest	

Camera/Chamber Location	~~Metrology ID#~~
Reference	
Nest 1	
Nest 2	
Nest 3	

24. **[M,D, L]** Notes: no metrology for ramp test

Date 4/29 Time 10:56 Initials JM

Test Scripts the Right and Left Mastcam-Zs ✓ #1 10:56 AM

25. **[D]** ____ Record the following temperatures:
    - Chamber temp ______________ Port temp ______________
    - Camera CCD temp R: -3.8 Optics temp L: -2.5
26. **[D,T]** ✓ ~~Take digital pictures of the geometric target's position, and the whole test/GSE set-up.~~ Andy, step 21
27. **[O,T]** ✓ Load and execute script **473TEMPR09**, which captures autofocused frames for three focal lengths with filter 0. Insert the note "TARGET=JR_DOT". The estimated duration is 4 minutes.
28. **[O,T]** ✓ Load and execute script **473TEMPL09**, which captures autofocused frames for three focal lengths with filter 0. Insert the note "TARGET=JR_DOT". The estimated duration is 4 minutes.
29. **[V,O,T]** Evaluate whether the target's dots are in-focus enough for discrimination.
30. **[D]** ____ Record image names and parameters in Image Log.
31. **[D, L]** Notes: ____

    image suffix: 16-27

Temp at Right and Left Mastcam-Zs

#2 11:06 AM

32. **[D]** ____ Record the following temperatures:
    - Chamber temp ______________ Port temp ______________
    - Camera CCD temp R: +1 Optics temp L: +2
33. ~~**[D,T]** ____ Take digital pictures of the geometric target's position, and the whole test/GSE set-up.~~
34. **[O,T]** ✓ Load and execute script **473TEMPR09**, which captures autofocused frames for three focal lengths with filter 0. Insert the note "TARGET=JR_DOT". The estimated duration is 4 minutes.
35. **[O,T]** ✓ Load and execute script **473TEMPL09**, which captures autofocused frames for three focal lengths with filter 0. Insert the note "TARGET=JR_DOT". The estimated duration is 4 minutes.
36. **[V,O,T]** Evaluate whether the target's dots are in-focus enough for discrimination.
37. **[D]** ____ Record image names and parameters in Image Log.
38. **[D, L]** Notes: image suffix: 28 - 39

Date 4/29 Time 11:15AM Initials [illegible]

Temp at Right and Left Mastcam-Zs

#3

~11:15AM

39. **[D]** ____ Record the following temperatures:
    - Chamber temp ______________ Port temp ______________
    - Camera CCD temp R: 5.3 Optics temp L: 5.8
40. **[D,T]** ____ ~~Take digital pictures of the geometric target's position, and the whole test/GSE set-up.~~
41. **[O,T]** ✓ Load and execute script **473TEMPR09**, which captures autofocused frames for three focal lengths with filter 0. Insert the note "TARGET=JR_DOT". The estimated duration is 4 minutes.
42. **[O,T]** ✓ Load and execute script **473TEMPL09**, which captures autofocused frames for three focal lengths with filter 0. Insert the note "TARGET=JR_DOT". The estimated duration is 4 minutes.
43. **[V,O,T]** Evaluate whether the target's dots are in-focus enough for discrimination.
44. **[D]** ____ Record image names and parameters in Image Log.
45. **[D, L]** Notes: image suffix: 40-51

Temp at Right and Left Mastcam-Zs

#4 ~11:20 AM

46. **[D]** ____ Record the following temperatures:
    - Chamber temp ______________ Port temp ______________
    - Camera CCD temp R: 11.7 Optics temp L: 11.2
47. ~~**[D,T]** ____ Take digital pictures of the geometric target's position, and the whole test/GSE set-up.~~
48. **[O,T]** ✓ Load and execute script **473TEMPR09**, which captures autofocused frames for three focal lengths with filter 0. Insert the note "TARGET=JR_DOT". The estimated duration is 4 minutes.
49. **[O,T]** ✓ Load and execute script **473TEMPL09**, which captures autofocused frames for three focal lengths with filter 0. Insert the note "TARGET=JR_DOT". The estimated duration is 4 minutes.
50. **[V,O,T]** Evaluate whether the target's dots are in-focus enough for discrimination.
51. **[D]** ____ Record image names and parameters in Image Log.
52. **[D, L]** Notes: image suffix: 52 - 63

Date 4/29 Time 11:30AM Initials JM

Temp at Right and Left Mastcam-Zs

#5 ~11:30AM

53. **[D]** ✓ Record the following temperatures:
    - Chamber temp ______________ Port temp ______________
    - Camera CCD temp R: 18.1°C Optics temp L: 17.7 °C
54. **[D,T]** ____ Take ~~digital pictures of the geometric target's position, and the whole test/GSE set-up.~~
55. **[O,T]** ✓ Load and execute script **473TEMPR09**, which captures autofocused frames for three focal lengths with filter 0. Insert the note "TARGET=JR_DOT". The estimated duration is 4 minutes.
56. **[O,T]** ✓ Load and execute script **473TEMPL09**, which captures autofocused frames for three focal lengths with filter 0. Insert the note "TARGET=JR_DOT". The estimated duration is 4 minutes.
57. **[V,O,T]** Evaluate whether the target's dots are in-focus enough for discrimination.
58. **[D]** ____ Record image names and parameters in Image Log.
59. **[D, L]** Notes:

image suffix: 64-75

#6 ~11:37 AM

Temp at Right and Left Mastcam-Zs

60. **[D]** ____ Record the following temperatures:
    - Chamber temp ____[illegible]____ Port temp ________
    - ~~Camera~~ Right CCD temp ____26.1 C____ ~~Optics~~ Left temp ____25.8 °C____
61. ~~**[D,T]** ____ Take digital pictures of the geometric target's position, and the whole test/GSE set-up.~~
62. **[O,T]** ✓ Load and execute script **473TEMPR09**, which captures autofocused frames for three focal lengths with filter 0. Insert the note "TARGET=JR_DOT". The estimated duration is 4 minutes.
63. **[O,T]** ✓ Load and execute script **473TEMPL09**, which captures autofocused frames for three focal lengths with filter 0. Insert the note "TARGET=JR_DOT". The estimated duration is 4 minutes.
64. **[V,O,T]** Evaluate whether the target's dots are in-focus enough for discrimination.
65. **[D]** ____ Record image names and parameters in Image Log.
66. **[D, L]** Notes: 76-87

    image suffix: 87 both

Date 4/29 Time 11:40AM Initials JM

#7 ~ 11:40 AM

Temp at Right and Left Mastcam-Zs

67. **[D]** ____ Record the following temperatures:
    - Chamber temp ______________ Port temp ______________
    - Camera CCD temp Left 32.2 ~~Optics~~ Right temp 31.4

68. **[D,T]** ~~____ Take digital pictures of the geometric target's position, and the whole test/GSE set-up.~~

69. **[O,T]** ✓ Load and execute script **473TEMPR09**, which captures autofocused frames for three focal lengths with filter 0. Insert the note "TARGET=JR_DOT". The estimated duration is 4 minutes.

70. **[O,T]** ✓ Load and execute script **473TEMPL09**, which captures autofocused frames for three focal lengths with filter 0. Insert the note "TARGET=JR_DOT". The estimated duration is 4 minutes.

71. **[V,O,T]** Evaluate whether the target's dots are in-focus enough for discrimination.

72. **[D]** ____ Record image names and parameters in Image Log.

73. **[D, L]** Notes: 88-99

image suffix: 99 both.

Date 4/29 Time 11:50 AM Initials [illegible]

Temp at Right and Left Mastcam-Zs #8

74. **[D]** ____ Record the following temperatures:

11:50 AM

- Chamber temp ______________ Port temp ______________
- Camera CCD temp ______________ Optics temp ______________

75. **[D,T]** ~~____ Take digital pictures of the geometric target's position, and the whole test/GSE set-up.~~

76. **[O,T]** √ Load and execute script **473TEMPR09**, which captures autofocused frames for three focal lengths with filter 0. Insert the note "TARGET=JR_DOT". The estimated duration is 4 minutes.

77. **[O,T]** √ Load and execute script **473TEMPL09**, which captures autofocused frames for three focal lengths with filter 0. Insert the note "TARGET=JR_DOT". The estimated duration is 4 minutes.

78. **[V,O,T]** Evaluate whether the target's dots are in-focus enough for discrimination.

79. **[D]** ____ Record image names and parameters in Image Log.

80. **[D, L]** Notes: image suffix: 111 both

Date 4/29 Time 11:55AM Initials gn

#9 ✓11:55AM

Temp at Right and Left Mastcam-Zs

81. **[D]** ____ Record the following temperatures:
    - Chamber temp ______________ Port temp ______________
    - Camera CCD temp R: 38.2°C Optics temp L: 36.7°C
82. **[D,T]** ____ ~~Take digital pictures of the geometric target's position, and the whole test/GSE set-up.~~
83. **[O,T]** ✓ Load and execute script **473TEMPR09**, which captures autofocused frames for three focal lengths with filter 0. Insert the note "TARGET=JR_DOT". The estimated duration is 4 minutes.
84. **[O,T]** ✓ Load and execute script **473TEMPL09**, which captures autofocused frames for three focal lengths with filter 0. Insert the note "TARGET=JR_DOT". The estimated duration is 4 minutes.
85. **[V,O,T]** Evaluate whether the target's dots are in-focus enough for discrimination.
86. **[D]** ____ Record image names and parameters in Image Log.
87. **[D, L]** Notes: image suffix: 123 both

Date 4/29 Time 11:59 AM Initials JM

Temp at Right and Left Mastcam-Zs #10 11:59 AM

88. **[D]** ____ Record the following temperatures:
    - Chamber temp ______________ Port temp ______________
    - Camera CCD temp R: 40.0°C Optics temp L: 38.4°C
89. **[D,T]** ____ Take digital pictures of the geometric target's position, and the whole test/GSE set-up.
90. **[O,T]** ____ Load and execute script **473TEMPR09**, which captures autofocused frames for three focal lengths with filter 0. Insert the note "TARGET=JR_DOT". The estimated duration is 4 minutes.
91. **[O,T]** ____ Load and execute script **473TEMPL09**, which captures autofocused frames for three focal lengths with filter 0. Insert the note "TARGET=JR_DOT". The estimated duration is 4 minutes.
92. **[V,O,T]** Evaluate whether the target's dots are in-focus enough for discrimination.
93. **[D]** ____ Record image names and parameters in Image Log.
94. **[D, L]** Notes:
    image suffix: 135 both

Temp at Right and Left Mastcam-Zs #11

95. **[D]** ____ Record the following temperatures:
   - Chamber temp (Aux. platen) 39.35°C Port temp ______________
   - Camera CCD temp ______________ Optics temp ______________
96. **[D,T]** ____ Take digital pictures of the geometric target's position, and the whole test/GSE set-up.
97. **[O,T]** ____ Load and execute script **473TEMPR09**, which captures autofocused frames for three focal lengths with filter 0. Insert the note "TARGET=JR_DOT". The estimated duration is 4 minutes.
98. **[O,T]** ____ Load and execute script **473TEMPL09**, which captures autofocused frames for three focal lengths with filter 0. Insert the note "TARGET=JR_DOT". The estimated duration is 4 minutes.
99. **[V,O,T]** Evaluate whether the target's dots are in-focus enough for discrimination.
100. **[D]** ____ Record image names and parameters in Image Log.
101. **[D, L]** Notes: image suffix: 1~~5~~7 both

/

Temp at Right and Left Mastcam-Zs #12

102. **[D]** ____ Record the following temperatures:
    - Chamber temp ______________ Port temp ______________
    - Camera CCD temp R: 41.8° C Optics temp L: 40.3 °C
103. **[D,T]** ____ Take digital pictures of the geometric target's position, and the whole test/GSE set-up.
104. **[O,T]** ____ Load and execute script **473TEMPR09**, which captures autofocused frames for three focal lengths with filter 0. Insert the note "TARGET=JR_DOT". The estimated duration is 4 minutes.
105. **[O,T]** ____ Load and execute script **473TEMPL09**, which captures autofocused frames for three focal lengths with filter 0. Insert the note "TARGET=JR_DOT". The estimated duration is 4 minutes.
106. **[V,O,T]** Evaluate whether the target's dots are in-focus enough for discrimination.
107. **[D]** ____ Record image names and parameters in Image Log.
108. **[D, L]** Notes:____

Date 4/29 Time 12:25 pm Initials JM

Temp at Right and Left Mastcam-Zs #13

109. **[D]** ____ Record the following temperatures:
   - Chamber temp aux platen: 38.58°C Port temp ____________
   - Camera CCD temp 42.4 °C Optics temp 40.8 °C
110. **[D,T]** ____ Take digital pictures of the geometric target's position, and the whole test/GSE set-up.
111. **[O,T]** ✓ Load and execute script **473TEMPR09**, which captures autofocused frames for three focal lengths with filter 0. Insert the note "TARGET=JR_DOT". The estimated duration is 4 minutes.
112. **[O,T]** ✓ Load and execute script **473TEMPL09**, which captures autofocused frames for three focal lengths with filter 0. Insert the note "TARGET=JR_DOT". The estimated duration is 4 minutes.
113. **[V,O,T]** Evaluate whether the target's dots are in-focus enough for discrimination.
114. **[D]** ____ Record image names and parameters in Image Log.
115. **[D, L]** Notes: image suffix: 171

 Date 4/29 Time 12:33 pm Initials JV

Temp at Right and Left Mastcam-Zs #14

116. **[D]** ____ Record the following temperatures: ✓
   - Chamber temp aux. plate 38.7 Port temp ______________
   - Camera CCD temp ______________ Optics temp ______________

117. **[D,T]** ____ ~~Take digital pictures of the geometric target's position, and the whole test/GSE set-up.~~

118. **[O,T]** ____ Load and execute script **473TEMPR09**, which captures autofocused frames for three focal lengths with filter 0. Insert the note "TARGET=JR_DOT". The estimated duration is 4 minutes.

119. **[O,T]** ____ Load and execute script **473TEMPL09**, which captures autofocused frames for three focal lengths with filter 0. Insert the note "TARGET=JR_DOT". The estimated duration is 4 minutes.

120. **[V,O,T]** Evaluate whether the target's dots are in-focus enough for discrimination.

121. **[D]** ____ Record image names and parameters in Image Log.

122. **[D, L]** Notes:

R 172 ← aborted/terminated early ~12:38 pm

L 175

↳ Tex reports that the camera has returned a mechanism health flag. He ~~believes~~ says that this appears to be a known issue ~~[illegible]~~ and will confer with Caplinger.

→ focus mech position at 0, backlash adds uncertainty, throws a mech fault homed mechanism, status flag clears continue, all nominal)

Date 4/29 Time 12:54pm Initials gm

Temp at Right and Left Mastcam-Zs

#15

123. **[D]** ____ Record the following temperatures:
    - Chamber temp ______________ Port temp ______________
    - Camera CCD temp 43.2 Optics temp 41.3
124. **[D,T]** ____ ~~Take digital pictures of the geometric target's position, and the whole test/GSE set-up.~~
125. **[O,T]** ✓ Load and execute script **473TEMPR09**, which captures autofocused frames for three focal lengths with filter 0. Insert the note "TARGET=JR_DOT". The estimated duration is 4 minutes.
126. **[O,T]** ✓ Load and execute script **473TEMPL09**, which captures autofocused frames for three focal lengths with filter 0. Insert the note "TARGET=JR_DOT". The estimated duration is 4 minutes.
127. **[V,O,T]** Evaluate whether the target's dots are in-focus enough for discrimination.
128. **[D]** ____ Record image names and parameters in Image Log.
129. **[D, L]** Notes:

R: 184

L 187

declare Ramp complete

Temp at Right and Left Mastcam-Zs

130. **[D]** ____ Record the following temperatures:
    - Chamber temp ______________ Port temp ______________
    - Camera CCD temp ______________ Optics temp ______________
131. **[D,T]** ____ Take digital pictures of the geometric target's position, and the whole test/GSE set-up.
132. **[O,T]** ____ Load and execute script **473TEMPR09**, which captures autofocused frames for three focal lengths with filter 0. Insert the note "TARGET=JR_DOT". The estimated duration is 4 minutes.
133. **[O,T]** ____ Load and execute script **473TEMPL09**, which captures autofocused frames for three focal lengths with filter 0. Insert the note "TARGET=JR_DOT". The estimated duration is 4 minutes.
134. **[V,O,T]** Evaluate whether the target's dots are in-focus enough for discrimination.
135. **[D]** ____ Record image names and parameters in Image Log.
136. **[D, L]** Notes:____

____

____

skip

Temp at Right and Left Mastcam-Zs

137. **[D]** ____ Record the following temperatures:
   - Chamber temp _______________ Port temp _______________
   - Camera CCD temp _______________ Optics temp _______________
138. **[D,T]** ____ Take digital pictures of the geometric target's position, and the whole test/GSE set-up.
139. **[O,T]** ____ Load and execute script **473TEMPR09**, which captures autofocused frames for three focal lengths with filter 0. Insert the note "TARGET=JR_DOT". The estimated duration is 4 minutes.
140. **[O,T]** ____ Load and execute script **473TEMPL09**, which captures autofocused frames for three focal lengths with filter 0. Insert the note "TARGET=JR_DOT". The estimated duration is 4 minutes.
141. **[V,O,T]** Evaluate whether the target's dots are in-focus enough for discrimination.
142. **[D]** ____ Record image names and parameters in Image Log.
143. **[D, L]** Notes:____

____

____

skip

Temp at Right and Left Mastcam-Zs

144. **[D]** ____ Record the following temperatures:
    - Chamber temp ______________ Port temp ______________
    - Camera CCD temp ______________ Optics temp ______________
145. **[D,T]** ____ Take digital pictures of the geometric target's position, and the whole test/GSE set-up.
146. **[O,T]** ____ Load and execute script **473TEMPR09**, which captures autofocused frames for three focal lengths with filter 0. Insert the note "TARGET=JR_DOT". The estimated duration is 4 minutes.
147. **[O,T]** ____ Load and execute script **473TEMPL09**, which captures autofocused frames for three focal lengths with filter 0. Insert the note "TARGET=JR_DOT". The estimated duration is 4 minutes.
148. **[V,O,T]** Evaluate whether the target's dots are in-focus enough for discrimination.
149. **[D]** ____ Record image names and parameters in Image Log.
150. **[D, L]** Notes:____

____

____

skip

Temp at Right and Left Mastcam-Zs

151. **[D]** ____ Record the following temperatures:
    - Chamber temp ______________ Port temp ______________
    - Camera CCD temp ______________ Optics temp ______________
152. **[D,T]** ____ Take digital pictures of the geometric target's position, and the whole test/GSE set-up.
153. **[O,T]** ____ Load and execute script **473TEMPR09**, which captures autofocused frames for three focal lengths with filter 0. Insert the note "TARGET=JR_DOT". The estimated duration is 4 minutes.
154. **[O,T]** ____ Load and execute script **473TEMPL09**, which captures autofocused frames for three focal lengths with filter 0. Insert the note "TARGET=JR_DOT". The estimated duration is 4 minutes.
155. **[V,O,T]** Evaluate whether the target's dots are in-focus enough for discrimination.
156. **[D]** ____ Record image names and parameters in Image Log.
157. **[D, L]** Notes:____

____

____

skip

Temp at Right and Left Mastcam-Zs

158. **[D]** ____ Record the following temperatures:
   - Chamber temp ______________ Port temp ______________
   - Camera CCD temp ______________ Optics temp ______________
159. **[D,T]** ____ Take digital pictures of the geometric target's position, and the whole test/GSE set-up.
160. **[O,T]** ____ Load and execute script **473TEMPR09**, which captures autofocused frames for three focal lengths with filter 0. Insert the note "TARGET=JR_DOT". The estimated duration is 4 minutes.
161. **[O,T]** ____ Load and execute script **473TEMPL09**, which captures autofocused frames for three focal lengths with filter 0. Insert the note "TARGET=JR_DOT". The estimated duration is 4 minutes.
162. **[V,O,T]** Evaluate whether the target's dots are in-focus enough for discrimination.
163. **[D]** ____ Record image names and parameters in Image Log.
164. **[D, L]** Notes:____

____

____

skip

Temp at Right and Left Mastcam-Zs

165. **[D]** ____ Record the following temperatures:
   - Chamber temp _______________ Port temp _______________
   - Camera CCD temp _______________ Optics temp _______________
166. **[D,T]** ____ Take digital pictures of the geometric target's position, and the whole test/GSE set-up.
167. **[O,T]** ____ Load and execute script **473TEMPR09**, which captures autofocused frames for three focal lengths with filter 0. Insert the note "TARGET=JR_DOT". The estimated duration is 4 minutes.
168. **[O,T]** ____ Load and execute script **473TEMPL09**, which captures autofocused frames for three focal lengths with filter 0. Insert the note "TARGET=JR_DOT". The estimated duration is 4 minutes.
169. **[V,O,T]** Evaluate whether the target's dots are in-focus enough for discrimination.
170. **[D]** ____ Record image names and parameters in Image Log.
171. **[D, L]** Notes:____

____

____

skip

Date ______ Time ______ Initials ______

Temp at Right and Left Mastcam-Zs

172. **[D]** ____ Record the following temperatures:
    - Chamber temp _______________ Port temp _______________
    - Camera CCD temp _______________ Optics temp _______________
173. **[D,T]** ____ Take digital pictures of the geometric target's position, and the whole test/GSE set-up.
174. **[O,T]** ____ Load and execute script **473TEMPR09**, which captures autofocused frames for three focal lengths with filter 0. Insert the note "TARGET=JR_DOT". The estimated duration is 4 minutes.
175. **[O,T]** ____ Load and execute script **473TEMPL09**, which captures autofocused frames for three focal lengths with filter 0. Insert the note "TARGET=JR_DOT". The estimated duration is 4 minutes.
176. **[V,O,T]** Evaluate whether the target's dots are in-focus enough for discrimination.
177. **[D]** ____ Record image names and parameters in Image Log.
178. **[D, L]** Notes:____

____

____

skip

Temp at Right and Left Mastcam-Zs

179. **[D]** ____ Record the following temperatures:
   - Chamber temp _______________ Port temp _______________
   - Camera CCD temp _______________ Optics temp _______________
180. **[D,T]** ____ Take digital pictures of the geometric target's position, and the whole test/GSE set-up.
181. **[O,T]** ____ Load and execute script **473TEMPR09**, which captures autofocused frames for three focal lengths with filter 0. Insert the note "TARGET=JR_DOT". The estimated duration is 4 minutes.
182. **[O,T]** ____ Load and execute script **473TEMPL09**, which captures autofocused frames for three focal lengths with filter 0. Insert the note "TARGET=JR_DOT". The estimated duration is 4 minutes.
183. **[V,O,T]** Evaluate whether the target's dots are in-focus enough for discrimination.
184. **[D]** ____ Record image names and parameters in Image Log.
185. **[D, L]** Notes:____

____

____

skip

Temp at Right and Left Mastcam-Zs

186. **[D]** ____ Record the following temperatures:
    - Chamber temp ______________ Port temp ______________
    - Camera CCD temp ______________ Optics temp ______________
187. **[D,T]** ____ Take digital pictures of the geometric target's position, and the whole test/GSE set-up.
188. **[O,T]** ____ Load and execute script **473TEMPR09**, which captures autofocused frames for three focal lengths with filter 0. Insert the note "TARGET=JR_DOT". The estimated duration is 4 minutes.
189. **[O,T]** ____ Load and execute script **473TEMPL09**, which captures autofocused frames for three focal lengths with filter 0. Insert the note "TARGET=JR_DOT". The estimated duration is 4 minutes.
190. **[V,O,T]** Evaluate whether the target's dots are in-focus enough for discrimination.
191. **[D]** ____ Record image names and parameters in Image Log.
192. **[D, L]** Notes:____

____

____

skip

Date 4/29 Time 13:08 Initials [illegible]

Target distance 2.06 m ✓ unchanged

Data Validation

193. **[V]** ✓ Run the "Geometric_46_Validation" Jupyter notebook on the acquired data for the Right and Left Mastcam-Zs. This analysis can take place while the test continues.

194. **[V,D, L]** Notes: Paul is running a variant of this analysis software
Paul reports data are nominal

end of ramp test

Test image: 44

Date 4/29 Time 13:14 Initials gn

Dark Current with the Right and Left Mastcam-Zs

195. **[T]** ✓ Cover the port window and turn off the lights.

196. **[D]** ____ Record the following temperatures:

- Chamber temp ______________ Port temp ______________
- Camera CCD temp R = 43.0 °C ~~Optics~~ temp L = 41.3 °C

197. **[D,T]** ✓ Take digital pictures of the ~~geometric target's position~~ setup, and the whole test/GSE set-up.

198. ✓ **[O]** Load and execute camera script **441TEMPR03**, which captures 5 dark frames through filter 7 at the exposure times 0.0, 1.0, 2.0, and 100 seconds. The estimated duration is 9 minute. (0 10. 20.0 100.0)

199. ✓ **[O]** Load and execute camera script **441TEMPL03**, which captures 5 dark frames through filter 7 at the exposure times 0.0, 1.0, 2.0, and 100 seconds. The estimated duration is 9 minute. 0, 10.0, 20.0, 100.0

200. **[D]** ____ Record image names and parameters in Image Log.

201. **[D, L]** Notes: img suffix: 66

Data Validation

202. **[V]** ✓ Run the "Dark_41_Validation" Jupyter notebook on the acquired data for the Right and Left Mastcam-Zs. This analysis can take place while the test continues.

203. **[V,D, L]** Notes: ok per paul

Shutdown Procedure

204. **[D,T]** ✓ Take digital pictures of this page and the test setup.
205. **[D,O]** ____ Review entries in Image Log, GSE command log, and image headers.
206. **[D, L]** ____ Review calibration procedure and ensure that each task is initialed.
207. **[D, L]** Notes: ______________________________

208. **[V, L]** ✓ Before making the decision to break down the test setup, ensure that adequate data were acquired for the test requirements. See "MastcamZCalPlan" for these requirements.
209. **[V]** Notes: ok per Paul analysis

Data Validator (signature) [signature]

Date 4-29-19 Time 1:54 PM

210. **[V, L]** ✓ Give the go/no-go decision. Have enough data been acquired to fulfill test requirements? See "MastcamZCalPlan" for these requirements.
211. **[D, L]** ____ Update the Log ~~Document~~. procedure is the log
212. **[L]** Notes: ______________________________

Calibration Lead (signature) [signature]

Date 4/29/19 Time 13:55

213. **[O, L]** ✓ Ensure that the camera and GSE are in a safe state. off & safe per Tex
214. **[O,D]** ✓ Review the Image Log with the documentarian. Exchange high-fives.
215. **[O]** Notes: high fives were exchanged

Camera Operator (signature) [signature]

Date 4/29/19 Time 13:56

216. **[T]** ✓ If the next test does not require the target, position it away from the chamber or bench. Otherwise, be sure not to move it. The next test is radiometric cal
Andy moved target
217. **[T]** ✓ Ensure that all other test equipment is safely put away.
218. **[T]** Notes: ____

Technician (signature) [signature]

Date 4/29/19 Time 13:58

219. **[D, L]** ____ Double-check this procedure and ensure that the top of each page has valid data, time and initials.
220. **[D]** ____ Photo-scan this document, save it on the cloud, and file the hard-copy in the Log Binder. Upload the digital pictures taken during this test in the appropriate archive on the cloud. The required links are on the Wiki.
221. **[D]** CT Double-check that every required cell the Image Log is accurately filled. When this is complete, print the Image Log and file it the Log Binder after this document.
222. **[D]** Notes: ____

Documentarian (signature) Christian Tate

Date 4-29-19 Time 15:08

Date 4/29 Time 18:00 Initials [illegible]

Radiometric Calibration Procedure for Mastcam-Z Ambient TVAC Testing at MSSS (Pro. 4.2.3)

[Procedure version 2.04, prepared by the Mastcam-Z calibration team at Cornell University]

These measurements are performed on the camera and at the temperature designated below as specified in the Mastcam-Z Calibration Plan,

Unit Under Test:

Left FM X **Right FM** X **EQM**____ **Other**____

These measurements are performed at temperature:

-35° C____ **-10°C** X **+5°C**____ **Ambient**____ **Other**____

These measurements are performed at,

MSSS X **ASU**____ **Other**____

Date 4/29 **Start Time** 18:~~[illegible]~~20 **End Time** 23:00

Estimated Duration 4.5 hours

Scheduled Start Time 18:00 **Sch. End Time** ~~20:00~~ 22:00

Calibration Lead [L] HERKENHOFF **Documentarian [D]** CORLIES

Camera Operator [O] VAN BEEK, DIXON **Technician [T]** WINHOLD

Data Validator [V] TATE **Other**____

Change Log

Version	Name	Change
v1_01<br>17 Sep 2018	C. Tate	(first draft)
v1_20<br>1 Nov 2018	C. Tate	Procedure edits prior to EQM testing
v1_23<br>10 Dec. 2018	C. Tate	Procedure edits after EQM testing
v2_03<br>29 April 2019	C. Tate	Approved version prior to FM testing

Document Approval

Approved by James Bell — Date
Mastcam-Z PI
Arizona State University

Approved by Alexander Hayes — Date
Mastcam-Z Calibration Working Group
Lead, Cornell University

Approved by Justin Maki — Date
Mastcam-Z Deputy PI and Investigation
Scientist, Jet Propulsion Laboratory

[signature] 4/29
Approved by Christian Tate — Date
Procedure Author
Cornell University

[signature]
Approved by — Date
Kenneth Herkenhoff 4-29-19

Date 4/29 Time 18:00 Initial [illegible]

Table of Contents

RADIOMETRIC CALIBRATION PROCEDURE FOR MASTCAM-Z AMBIENT TVAC TESTING AT MSSS (PRO. 4.2.3) 1

CHANGE LOG 2
DOCUMENT APPROVAL 2
TEST DESCRIPTION 4
SOFTWARE PREPARATION 4
Table 1. File naming convention for the camera script prefixes and frame filenames: "AAABBBBCDD" 4
HARDWARE INSTALLATION 6
Figure 1. ASU Floor Plan for Geometric Testing in the TVAC Chamber. The MSSS Floor Plan allows for similar target and source placements relative to the chamber window. 6
Table 2. The Nominal Radiance Values (calibrated integrating sphere output). 8

RIGHT AND LEFT MASTCAM-Z TESTS 9

CENTER THE INTEGRATING SPHERE .... 9 ← Darks
RADIANCE VALUE 1 FOR THE RIGHT MASTCAM-ZS 11
RADIANCE VALUE 1 FOR THE LEFT MASTCAM-ZS 12
RADIANCE VALUE 2 FOR THE LEFT MASTCAM-Z 13
RADIANCE VALUE 2 FOR THE RIGHT MASTCAM-Z 14
DATA VALIDATION .... 15 ← Darks

SHUTDOWN PROCEDURE 17

Test Description

Excerpt from the Calibration Plan 4.2,

> The objectives of these tests are to derive flat field images as well as the coefficients to allow a conversion from reduced (bias, dark, and flat field corrected) DN/s to absolute radiometric response (W/cm^2/sr per filter) for (a) the R, G, and B microfilters of the Bayer Pattern Filter detectors in each camera head (clear filter), (b) the 14 non-solar Mastcam-Z spectral filters "Science Filters", and, if time permits, (c) the two Mastcam-Z neutral density solar filters; and to provide an estimate of the uncertainty in these coefficients and, at Priority 2, their temperature dependence. This test builds off the Section 4.3 – Spectral Throughput Calibration to accurately account for the filter spectral response in the conversion. The requirement of knowing the relative response on the shape of the spectral throughput to ±5% combined with the absolute Radiance accuracy of the integration sphere at ±5% still allows the ±10% absolute radiometric calibration requirement to be met.

Software Preparation

The software and files required for this test are prepared well in advance of test day. This checklist ensures that the following are present, debugged, and executable: (1) all fast-look scripts, (2) automated header generation of all relevant camera parameters, target positioning, and metadata, (3) all camera scripts that command the camera unit, and (4) the directories/file-paths pointing to the data repositories of this specific test.

Table 1. File naming convention for the camera script prefixes and frame filenames: "AAABBBBCDD"

Code	Name	Example	Value
"AAA"	Calibration Plan Section	"411" = Cal. Plan 4.1.1 chapter 4, section 1, subsection 1	423
"BBBB"	Location of test or ASU Chamber temperature	"MSSS" = test at MSSS, "TN10" = ASU TVAC -10C, ...	TAMB
"C"	Camera unit under test	"L" = Left Mastcam-Z, "R" = Right Mastcam-Z, "E" =EQM, "C" =COTS	R/L
"DD"	Part of test (radiance value)	"00" = test set up, "01" = first radiance value ...	00-08

1. **[D]** ✓ Look up the daily calibration schedule and record the scheduled start and end time of this test on the cover page of this document. Also fill out and double-check the other information on the cover page.
2. **[D]** ____ Ensure that all supplemental manuals are on hand. These are,
    - Labsphere_Manual,
    - Validator_Manual, Documentarian_Manual
    - MastcamZCalPlan
3. **[D]** ✓ Ensure that the Image Log is present and ready to use. Find and open the Google Sheets file "Image_Log_42". There is a link on the Wiki.
4. **[V]** [illegible] Check that all Calgorithms fast-look and validation scripts are present, up-to-date, and ready to analyze test output. Find and open the "Radiometric_Calibration_42_Validation" Jupyter notebook. There is a link on the Wiki.
5. **[O]** [illegible] Check that all camera scripts required for this test are present, up-to-date and ready to command the ground support equipment (GSE). These are,
    - **412TAMBR00 - 412TAMBR08** and **423TAMBR00 - 423TAMBR06**
    - **412TAMBL00 - 412TAMBL08** and **423TAMBL00 - 423TAMBL06**
    - **441TEMPR03 and 441TEMPL03**
6. **[O,V,D, L]** Notes:

ADDED DARK CURRENT SEQUENCE BEFORE THIS RADIOMETRIC TEST DUE TO SLOWLY CHANGING CAMERA TEMPERATURES. GOAL IS TO ALLOW RADIOMETRY IMAGES TO BE DARK-CORRECTED VIA INTERPOLATION OF DATA ACQUIRED BEFORE AND AFTER RADIOMETRY.

Hardware Installation

This procedure is for the ambient TVAC chamber testing at MSSS. Figure 1 shows the nominal layout of the TVAC chamber, workspace, Mastcam-Zs, ground support equipment (GSE), targets, sources, and other equipment necessary for this test if it happens at ASU. Although MSSS' cleanroom is different than ASU's, the placement of the targets and sources relative to the chamber window is similar.

Figure 1. ASU Floor Plan for Geometric Testing in the TVAC Chamber. The MSSS Floor Plan allows for similar target and source placements relative to the chamber window.

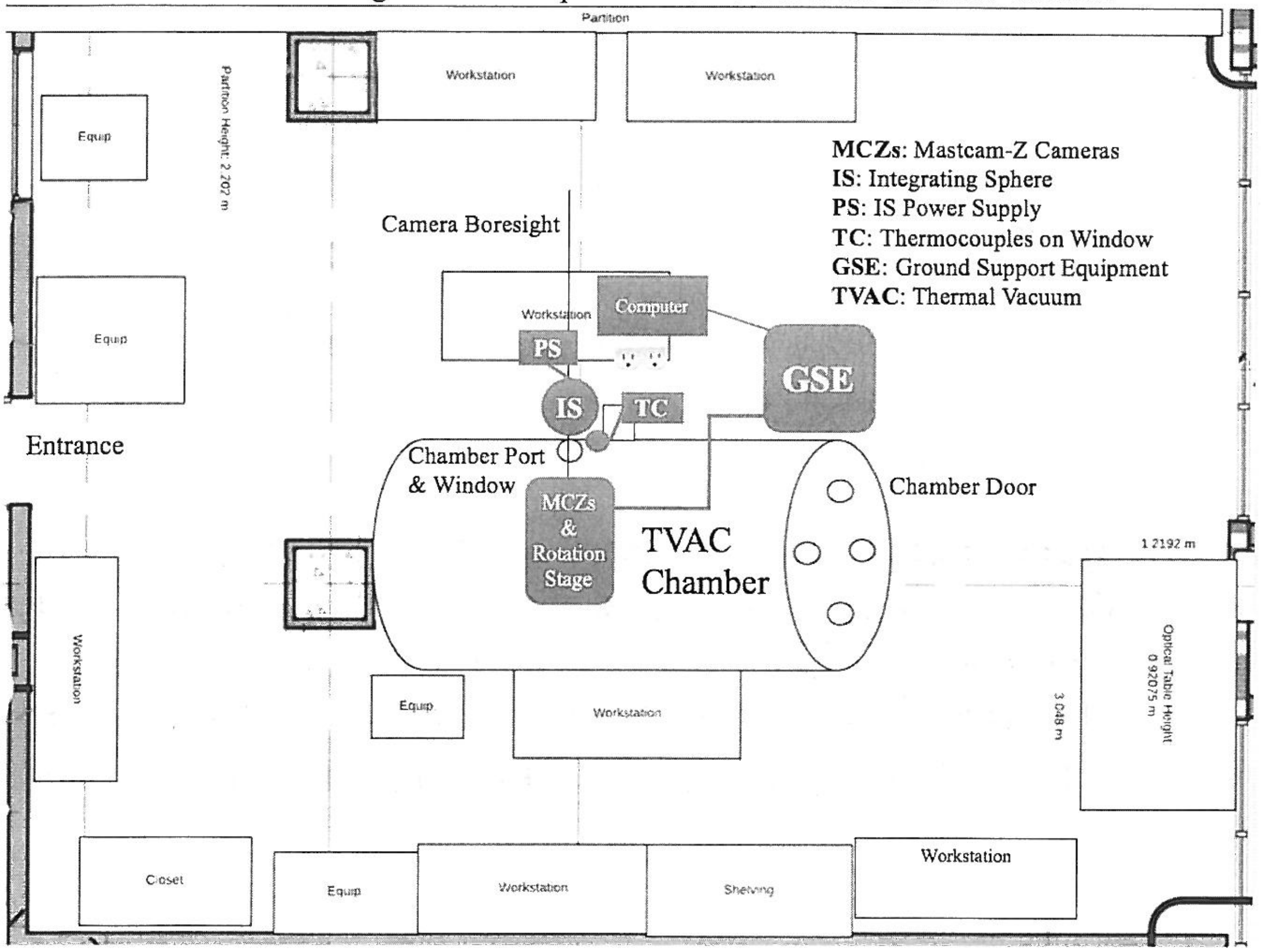

7. **[T,O, L]** ___ Ensure that all personnel in the cleanroom are following the cleanroom practices for electrostatic discharge, proper clothing and other safety concerns.
8. **[T]** ___ Double check that nitrogen is flowing over the Mastcam-Zs or the window port.

9. **[O,T]** CT If not already done, mate the Right and Left Mastcam-Zs into the GSE. Follow the procedure in "MastcamZ_GSE_Manual".
10. **[T]** CT Verify that the thermocouples are turned on and properly reading out.
11. **[O,T]** CT Ensure that the camera unit and GSE wires are secure, kink-free, and do not present tripping hazards when the lights are turned off.
12. **[O,D]** CT Check the camera temperature and ensure nominal operation.
13. **[D]** CT Record the following environmental information:
    - Cleanroom temperature 24.8 pressure ~~24.8~~ humidity 44
14. **[O,D, L]** Notes:

___

___

___

15. **[D,T]** CT Take time-stamped pictures of this page, the integrating sphere, and the whole test/GSE set-up.
16. **[T]** CT Power on the integrating sphere. Follow the procedure in "Labsphere_Manual". Record the time the lamp is turned on 18:17.
17. **[D,T]** [illegible] Record the exact readout value of the integrating sphere's radiance: -0.1479 mW/cm2/sr.
18. **[T,O, L]** CT Confirm that the camera systems and GSEs are powered on and ready for use. Follow the procedure in "MastcamZ_GSE_Manual".
19. **[D, L]** Notes:

ABOVE RECORDED DURING DARK

___

___

Date 4/29 Time 18:48 Initial ZC

Table 2. The Nominal Radiance Values (calibrated integrating sphere output).

IS Output Radiances	Nominal Radiance [mW/cm2/sr]
Radiance 1	5.0
Radiance 2	10.0

Right and Left Mastcam-Z Tests

Dark Current with the Right and Left Mastcam-Zs

20. **[T]** [initials] Cover the port window and turn off the lights.
21. **[D]** [initials] Record the following temperatures:
    i. Chamber temp ________ Port temp ________
    ii. Camera CCD temp R: 30.2° L: 29.6 °C Optics temp ________
22. **[D,T]** [initials] Take digital pictures of the geometric target's position, and the whole test/GSE set-up.
23. **[O]** Load and execute camera script **441TEMPR03**, which captures 5 dark frames through filter 7 at the exposure times 0.0, 10.0, 20.0, and 100 seconds. The estimated duration is 10 minutes.
24. **[O]** Load and execute camera script **441TEMPL03**, which captures 5 dark frames through filter 7 at the exposure times 0.0, 10.0, 20.0, and 100 seconds. The estimated duration is 10 minutes.
25. **[D]** [initials] Record image names and parameters in Image Log.
26. **[T]** [initials] Uncover the port window.
27. **[D, L]** Notes: LAST SUFFIX 88 FOR BOTH CAMERAS

Center the Integrating Sphere

28. **[T]** [initials] Move integrating sphere output as close to the chamber window as possible centered on the Right Mastcam-Z boresight. ~1 cm between
29. **[T]** [initials] Turn off lights. LIGHTS NEXT DOOR ON, ILLUMINATING CHAMBER
30. **[O]** [initials] Insert the note "ISOP=[radiance]" and execute camera script **423TAMBR00**. This script captures one auto-exposure at 40% full-well and one bias frame for filter 0 at 26 mm focal length. 4.998 $mW cm^{-2} sr^{-1}$

31. **[V,O,T]** Ze Open images, and if the images show that the integrating sphere is not centered, center the integrating sphere disc in the frame. Recapture **423TAMBR00** frames if necessary.
32. **[D]** Ze Record image names and parameters in the image Log.
33. **[O,D,V]** ____ Approximate the full-well percentage of the center pixels of the image and verify that they are about 40% (or DN 155). SKIP
34. **[T]** Ze Lights off
35. **[D, L]** Notes:

___

___

___

Date 4/29 Time 9:02 Initial [initials]

Radiance Value 1 for the Right Mastcam-Zs

36. **[T]** [initials] Set integrating sphere output to the radiance value 1 defined in Table 2.
37. **[D,T]** [initials] Record exact integrating sphere readout value 4.9863 mW/cm2/sr.
38. **[D]** [initials] Record temperature information:
    - Chamber temp ________________ Port temp ______________
    - Camera CCD temp 30.9°C Optics temp ______________
39. **[D,T]** [initials] Take time-stamped digital pictures of the setup and integrating sphere readout.
40. **[O]** [initials] Insert the note "ISOP=[radiance]" and execute camera script **423TAMBR02**, which captures 5 frames for ~~40% and~~ TBC 80% full-well and ~~10 bias frames~~ TBC with the 7 non-solar filters at three focal lengths. The estimated duration is 40 minutes.
41. **[D,T]** [initials] Record exact integrating sphere readout value 4.9863 → 4.9888 INTERMITTENTLY mW/cm2/sr.
42. **[O]** [initials] Insert the note "ISOP=[radiance]" and execute camera script **41~~3~~2TAMBR05**, which captures 10 frames for 9 exposure times with filter 0 at 100mm focal length. The estimated duration is 10 minutess.
43. **[D,T]** ____ Record exact integrating sphere readout value __________ mW/cm2/sr.
44. **[D]** ____ Record image names and parameters in the Image Log.
45. **[D, L]** Notes: FOCAL LENGTHS = 34, 63, 100 mm
DECIDED TO GO TO RADIANCE VALUE 2 FOR RIGHT EYE BEFORE MOVING TO LEFT ~~EE~~ EYE

LEFT TEST IMAGE WITH SPHERE = 4.9838 mW cm^{-2} sr^{-1}

RIGHT EYE SPHERE IMAGES NOT PERFECTLY CENTERED, BUT DEEMED CLOSE ENOUGH.

RAN LEFT EYE PHOTON TRANSFER SERIES RUN DURING RT. RAD.
→ 412TAMBL05, WITH INITIAL FLUX OF 4.9888 mW/cm²/sr, EVEN THOUGH SPHERE OUTPUT NOT WELL CENTERED: STEPS 56-59
→ ALSO 412TAMBL08 AT RADIANCE 2.
GO TO PG. 14.

Date 4/29 Time 21:00 Initial Z

Radiance Value 1 for the Left Mastcam-Zs

46. **[T]** Z Pull the integrating sphere away from the cameras and point it into a dark corner of the room before taking another radiance reading. Record exact integrating sphere readout value 4.9514 mW/cm2/sr.
47. **[T]** Z Center the integrating sphere for the Left Mastcam-Z. Do not change the radiance value.
48. **[O]** Z Insert the note "ISOP=[radiance]" and execute camera script **423TAMBL00**. This script captures one auto-exposure at 40% full-well and one bias frame for filter 0 at 26 mm focal length.
49. **[V,O,T]** Z Open images, and if the images show that the integrating sphere is not centered, center the integrating sphere disc in the frame. Recapture **423TAMBL00** frames if necessary.
50. **[D]** ____ Record image names and parameters in the image Log.
51. **[D,T]** ~~Z~~ Record exact integrating sphere readout value ~~4.9183~~ 5.0013 mW/cm2/sr.
52. **[D]** ~~Z~~ Record temperature information:
    - Chamber temp ________________ Port temp ______________
    - Camera CCD temp ~~29.8°~~ 29.8° Optics temp ______________
53. **[D,T]** ~~Z~~ Take time-stamped digital pictures of the setup and integrating sphere readout.
54. **[O]** Z Insert the note "ISOP=[radiance]" and execute camera script **423TAMBL02**, which captures 5 frames for 40% and 80% full-well and 10 bias frames with the 7 non-solar filters at three focal lengths. The estimated duration is 40 minutes.
55. **[D,T]** Z Record exact integrating sphere readout value 5.0187 mW/cm2/sr.
56. **[O]** Z Insert the note "ISOP=[radiance]" and execute camera script **41~~3~~2TAMBL05**, which captures 10 frames for 9 exposure times with filter 0 at 100mm focal length. The estimated duration is 10 minutess.
57. **[D,T]** Z Record exact integrating sphere readout value __________ mW/cm2/sr.
58. **[D]** Z Record image names and parameters in the Image Log.
59. **[D, L]** Notes: IMAGE NOT WELL CENTERED, TAKEN DURING RT. RADIOMETRY

Date 4/29 Time ~~20:03~~ 20:40 Initial [initials]

____

____

Radiance Value 2 for the Left Mastcam-Z

SKIP 60. **[T]** ____ Set integrating sphere output to the radiance value 2 defined in Table 2.

61. **[D,T]** [initials] Record exact integrating sphere readout value 9.9183 mW/cm2/sr.

62. **[D]** ____ Record temperature information:

- Chamber temp ________________ Port temp ______________
- Camera CCD temp 29.8°C Optics temp ______________

63. **[D,T]** [initials] Take time-stamped digital pictures of the setup and integrating sphere readout.

64. **[O]** [initials] Insert the note "ISOP=[radiance]" and execute camera script **423TAMBL02**, which captures 5 frames for 40% and 80% full-well and 10 bias frames with the 7 non-solar filters at three focal lengths. The estimated duration is 40 minutes.

65. **[D,T]** [initials] Record exact integrating sphere readout value 9.9976 mW/cm2/sr.

RAN IN PARALLEL WITH RT. RADIOMETRY @ RAD. VALUE 2. → 66. **[O]** [initials] Insert the note "ISOP=[radiance]" and execute camera script **4~~1~~23TAMBL08**, which captures 10 frames for 9 exposure times with filter 0 at 100mm focal length. The estimated duration is 10 minutess.

~~SEE PG. 11~~ 67. **[O]** [initials] If time permits, insert the note "ISOP=[radiance]" and execute camera script **423TAMBL05**, which captures 5 frames for ~~4~~80% ~~and 80%~~ full-well ~~and 5 bias frames~~ with filters 0 and 1 at seven focal lengths. These images are comp~~ou~~ anded at 8-bits. The estimated duration is 12 minutes.

68. **[D,T]** [initials] Record exact integrating sphere readout value 10.003 mW/cm2/sr.

69. **[D]** ____ Record image names and parameters in the Image Log.

70. **[D, L]** Notes: GO TO STEP 47, THEN 61. WHEN CENTERED INSERTED RT. CAMERA STEPS 81

____

Date 4/29 Time 20:03 Initial [illegible]

Radiance Value 2 for the Right Mastcam-Z

71. **[T]** ____ Pull the integrating sphere away from the cameras and point it into a dark corner of the room before taking another radiance reading. Record exact integrating sphere readout value __________ mW/cm2/sr. SKIP
72. **[T]** ✓ Center the integrating sphere for the Right Mastcam-Z. Do not change the radiance value. ALREADY DONE
73. **[O]** ____ Insert the note "ISOP=[radiance]" and execute camera script **423TAMBR00**. This script captures one auto-exposure at 40% full-well and one bias frame for filter 0 at 26 mm focal length.
74. **[V,O,T]** ____ Open images, and if the images show that the integrating sphere is not centered, center the integrating sphere disc in the frame. Recapture **423TAMBR00** frames if necessary.
75. **[D]** ____ Record image names and parameters in the image Log.

SKIP, RAN LATER (items 73–75)

76. **[D,T]** [initials] Record exact integrating sphere readout value 10.005 mW/cm2/sr.
77. **[D]** ____ Record temperature information:
    - Chamber temp ________________ Port temp ______________
    - Camera CCD temp 30.6 °C Optics temp ______________
78. **[D,T]** ____ Take time-stamped digital pictures of the setup and integrating sphere readout. SKIP
79. **[O]** [initials] Insert the note "ISOP=[radiance]" and execute camera script **423TAMBR02**, which captures 5 frames for 40% and 80% full-well and 10 bias frames with the 7 non-solar filters at three focal lengths. The estimated duration is 40 minutes.
80. **[D,T]** [initials] Record exact integrating sphere readout value 10.003 mW/cm2/sr.
81. **[O]** [initials] Insert the note "ISOP=[radiance]" and execute camera script **41~~3~~2TAMBR08**, which captures 10 frames for 9 exposure times with filter 0 at 100mm focal length. The estimated duration is 10 minute~~s~~s.
82. **[O]** [initials] If time permits, insert the note "ISOP=[radiance]" and execute camera script **423TAMBR05**, which captures 5 frames for ~~40% and~~ 80% full-well ~~and 5 bias frames~~

RADIANCE = 9.9202

with filters 0 and 1 at seven focal lengths. These images are compaunded at 8-bits. The estimated duration is 12 minutes.

83. **[D,T]** [illegible] Record exact integrating sphere readout value 9.9202 mW/cm2/sr.

84. **[D]** ____ Record image names and parameters in the Image Log.

85. **[D, L]** Notes: DONE DURING LEFT EYE RADIOMETRY

Data Validation

86. **[T]** ____ Lights on

87. **[V]** ____ Upload data to server.

88. **[V]** [illegible] Run the "Radiometric_Calibration_42_Validation" Jupyter notebook on the acquired data for the Right ✓ and Left ✓ Mastcam-Z with the window off. This analysis can take place while the test continues.

- Create preliminary flat-field images and radiometric coefficients for each filter.
- Save results in the calibration records.

89. **[V,D, L]** Notes: The Right flats and photon transfer curves look good. [illegible]

Dark Current with the Right and Left Mastcam-Zs

90. **[T]** En Cover the port window and turn off the lights.
91. **[D]** ____ Record the following temperatures:
    iii. Chamber temp ______________ Port temp ______________
    iv. Camera CCD temp L: 29.5, R: 30.1 Optics temp ______________
92. **[D,T]** En Take digital pictures of the geometric target's position, and the whole test/GSE set-up.
93. **[O]** Load and execute camera script **441TEMPR03**, which captures 5 dark frames through filter 7 at the exposure times 0.0, 10.0, 20.0, and 100 seconds. The estimated duration is 10 minutes.
94. **[O]** Load and execute camera script **441TEMPL03**, which captures 5 dark frames through filter 7 at the exposure times 0.0, 10.0, 20.0, and 100 seconds. The estimated duration is 10 minutes.
95. **[D]** CO Record image names and parameters in Image Log.
96. **[T]** ____ Uncover the port window.
97. **[D, L]** Notes: L: 89
    R: 89

Date 4/29 Time 22:50 Initial [illegible]

Shutdown Procedure

98. **[D,T]** CS Take digital pictures of this page and the test setup.
99. **[D,O]** CS Review entries in Image Log, GSE command log, and image headers.
100. **[D, L]** ____ Review calibration procedure and ensure that each task is initialed.
101. **[D, L]** Notes: DRY N PURGE TURNED BACK ON.

102. **[V, L]** [illegible] Before making the decision to break down the test setup, ensure that adequate data were acquired for the test requirements. See "MastcamZCalPlan" for these requirements.
103. **[V]** Notes: ____

Data Validator (signature) [illegible signature]

Date 4-29-19 Time 10:54

104. **[V, L]** [illegible] Give the go/no-go decision. Have enough data been acquired to fulfill test requirements? See "MastcamZCalPlan" for these requirements.
105. **[D, L]** [illegible] Update the Log Document.
106. **[L]** Notes: UPDATED TAG-UP START TIME TO 8:30 PDT TOMORROW.

Calibration Lead (signature) [illegible signature]

Date 4/29/19 Time 23:01

107. **[O, L]** JVB Ensure that the camera and GSE are in a safe state.
108. **[O,D]** ____ Review the Image Log with the documentarian. Exchange high-fives.
109. **[O]** Notes: No Concerns

Camera Operator (signature) [signature]

Date 4-29-19 Time 11:02

110. **[T]** AW If the next test does not require the integrating sphere, position it away from the chamber or bench. Otherwise, be sure not to move it. The next test is _______.
111. **[T]** AW Ensure that all other test equipment is safely put away.
112. **[T]** Notes: GN2 VALVE TURNED 1/4 TURN CW SO THE VALVE WAS ⊥ TO WALL. GAS COULD BE HEARD FLOWING ~11pm (HAD BEEN CLOSED ~6pm FOR TESTING) FOR CHAMBER

Technician (signature) [signature]

Date 4/29/19 Time 23:10

113. **[D, L]** ____ Double-check this procedure and ensure that the top of each page is initialed with the time and date.
114. **[D]** ____ Photo-scan this document, save it on the cloud, and file the hardcopy in the Log Binder. Upload the digital pictures taken during this test in the appropriate archive on the cloud. The required links are on the Wiki.
115. **[D]** CJD Double-check that every required cell the Image Log is accurately filled. When this is complete, print the Image Log and file it the Log Binder after this document.
116. **[D]** Notes: ____________

Documentarian (signature) [signature]

Date 4-29-19 Time 10:57

Date 4/30 Time 8:30 Initials [illegible]

MTF Calibration Procedure for Mastcam-Z Ambient TVAC Testing (Pro. 4.7.1)

[Procedure version 2.06, prepared by the Mastcam-Z calibration team at Cornell University.]

These measurements are performed on the camera and at the temperature designated below as specified in the Calibration Plan (Document #),

Unit Under Test:

R FM X **L FM** X **EQM**____ **Other**____

Test Performed at Temperature:

-35°C____ **- 10°C**____ **+5°C**____ **Ambient** X **Other** ____

These measurements are performed at,

MSSS X **ASU**____ **Other**____

Date 4/30/19 **Start Time** 8:30 AM **End Time** ____

Estimated Duration 4.0 hours

Scheduled Start Time 8:30 AM **Sch. End Time**____

**Calibration Lead [L]**	Maki	**Documentarian [D]**	Christian
**Camera Operator [O]**	Tex, Elsa	**Technician [T]**	Andy
**Data Validator [V]**	Paul	**Metrologist [M]**	N/A
**Other**			

Date 5/30 Time 9:04 Initials gm

Change Log

Version	Name	Change
v1_01<br>1 Oct 2018	C. Tate	(first draft)
v2_03<br>30 April 2019	C. Tate	Approved version prior to FM testing

Document Approval

Approved by James Bell Date
Mastcam-Z PI
Arizona State University

Approved by Alexander Hayes Date
Mastcam-Z Calibration Working Group Lead, Cornell University

4/30/19

Approved by Justin Maki Date
Mastcam-Z Deputy PI and Investigation Scientist, Jet Propulsion Laboratory

Approved by Christian Tate Date
Procedure Author
Cornell University

Approved by: Date

Date 5/30 Time 9:04 Initials [illegible]

Table of Contents

MTF CALIBRATION PROCEDURE FOR MASTCAM-Z AMBIENT TVAC TESTING (PRO. 4.7.1) 1
CHANGE LOG 2
DOCUMENT APPROVAL 2
TEST DESCRIPTION 3
SOFTWARE PREPARATION 4
Table 1. File naming convention for the camera script prefixes and frame filenames: "AAABBBBCDD" 4
HARDWARE INSTALLATION 5
Table 2. The nominal target placement scenes for the geometric testing 7
SCENE 3 FOR THE LEFT MASTCAM-Z 8
SCENE 3 FOR THE RIGHT MASTCAM-Z 9
SCENE 1 FOR THE LEFT MASTCAM-Z 10
SCENE 1 FOR THE RIGHT MASTCAM-Z 11
SCENE 2 FOR THE LEFT MASTCAM-Z 12
SCENE 2 FOR THE RIGHT MASTCAM-Z 13
TIME CHECK 1 14
SCENE 3 Z-STACK FOR THE LEFT MASTCAM-Z 15
SCENE 3 Z-STACK FOR THE RIGHT MASTCAM-Z 16
SHUTDOWN PROCEDURE **17**

Test Description

Excerpt from the Calibration Plan 4.7

The objective of this test is to image well-characterized bar targets at multiple focus and zoom positions in order to characterize the Modulation Transfer Function (MTF) and depth of field of each camera. Targets should be imaged at ~50% full well using the Bayer RGB (priority 1), 805 nm, (priority 2), and remaining non-solar filters (priority 3). Obtain a minimum of 3 images of each target per filter, focus, and zoom position. Multiple images are needed to reduce errors in determining target locations in the image plane.

MTF is an effective means of specifying the resolution of an optical system. Resolution is defined as the minimum feature size of an object that can be distinguished by an imaging system. The Point Spread Function (PSF) is the inverse Fourier Transform of the MTF—the PSF describes optical performance in the spatial domain while the MTF expresses optical performance in the frequency domain. Images of the bar targets and knife edge or point source targets at various zoom and focus positions will be used to determine PSF, depth of field, and MTF. The bar target shall consist of a chart containing horizontal, vertical, and diagonal lines and bars of varying thicknesses as well as circular dots of various sub- and super-pixel sizes.

In addition to determining the optical performance of the optomechanical assemblies, the images collected during MTF/PSF calibration will also be used to determine the numerical value and repeatability of the stepper motor counts for the Hall Effect sensors used to measure the position of the focus group and two moving zoom groups in the optical zoom assemblies. This will determine the relationship between stepper motor count for each focus/zoom group, working distance, and pixel scale. Owing to thickness variations between spectral filters, focus shifts may occur and images would ideally be obtained using all non-solar filters.

Note that the Mastcam-Z instrument has an onboard focus merge algorithm that selects and merges the best-focus portions of a scene using a focus stack (or z-stack) of multiple images (up to 16) at varying focal positions. While usually only the best-focus or merged product is saved to file, the raw frames for the focus stack can also be saved to file without appreciably increasing the observation or product generation time. For tests where images at multiple focus positions are desired, such as during MTF/PSF Calibrations, the entire focus stack of images will be written to file. Since images at multiple focus positions are acquired irrespective of whether or not they are written to memory, saving multiple focus positions per zoom setting does not require additional test time (outside of the time required to write the files to disk in the GSE). Multiple focus position images shall be saved during both Stand-Alone and ATLO MTF/PSF testing.

Software Preparation

The software and files required for this test are prepared well in advance of test day. This checklist ensures that the following are present, debugged, and executable: (1) all fast look scripts, (2) automated header generation of all relevant camera parameters, target positioning, and metadata, (3) all camera scripts that command the camera unit, and (4) the directories/file-paths pointing to the data repositories of this specific test.

Table 1. File naming convention for the camera script prefixes and frame filenames: "AAABBBBCDD"

Code	Name	Example	Value(s)
"AAA"	Calibration Plan Section	"411" = Cal. Plan 4.1.1 chapter 4, section 1, subsection 1	471, 491
"BBBB"	Location of test or ASU TVAC temperature	"MSSS" = test at MSSS, "ATLO" = test at JPL ATLO, "TN10" = ASU TVAC -10C, …	TAMB
"C"	Camera unit under test	"L" = Left Mastcam-Z, "R" = Right Mastcam-Z, "E" =EQM, "C" =COTS	R/L
"DD"	Part of test	"00" = test set up, "01" = first radiance level …	00-12

Date 4/30 Time 9:05 Initials JR

1. **[D]** ____ Look up the daily calibration schedule and record the scheduled start and end time of this test on the cover page of this document. Also, fill out and double-check the other information on the cover page. N/A
2. **[D]** ✓ Ensure that all supplemental manuals are on hand. These are,
   - Validator_Manual, Documentarian_Manual,
   - MastcamZCalPlan
3. **[D]** ✓ Ensure that the Image Log is present and ready to use. Find and open the Google Sheets file "Image_Log_46". The duration is 2 minutes. There is a link on the Wiki.
4. **[V]** ✓ Check that all *Calgorithms* fast-look and validation scripts are present, up-to-date, and ready to analyze test output. Find and open the "Geometric_Calibration_47_Validation" Jupyter notebook. There is a link on the Wiki.
5. **[O]** ✓ Check that all camera scripts required for this test are present, up-to-date and ready to command the ground support equipment (GSE). These are,
   - **471TAMBR00 - 471TAMBR13, 491TAMBR00 - 491TAMBR12**
   - **471TAMBL00 - 471TAMBL13, 491TAMBL00 - 491TAMBL12**
6. **[O,V,D, L]** Notes: scripts confirmed

Hardware Installation

This procedure is for the ambient TVAC chamber testing at MSSS. Figure 1 shows the nominal layout of the TVAC chamber, workspace, Mastcam-Zs, ground support equipment (GSE), targets, sources, and other equipment necessary for this test if it happens at ASU. Although MSSS' cleanroom is different than ASU's, the placement of the targets and sources relative to the chamber window is similar.

MSSS see photos

Figure 1. A~~SU~~ Floor Plan for Geometric Testing in the TVAC Chamber. The MSSS Floor Plan allows for similar target and source placements relative to the chamber window.

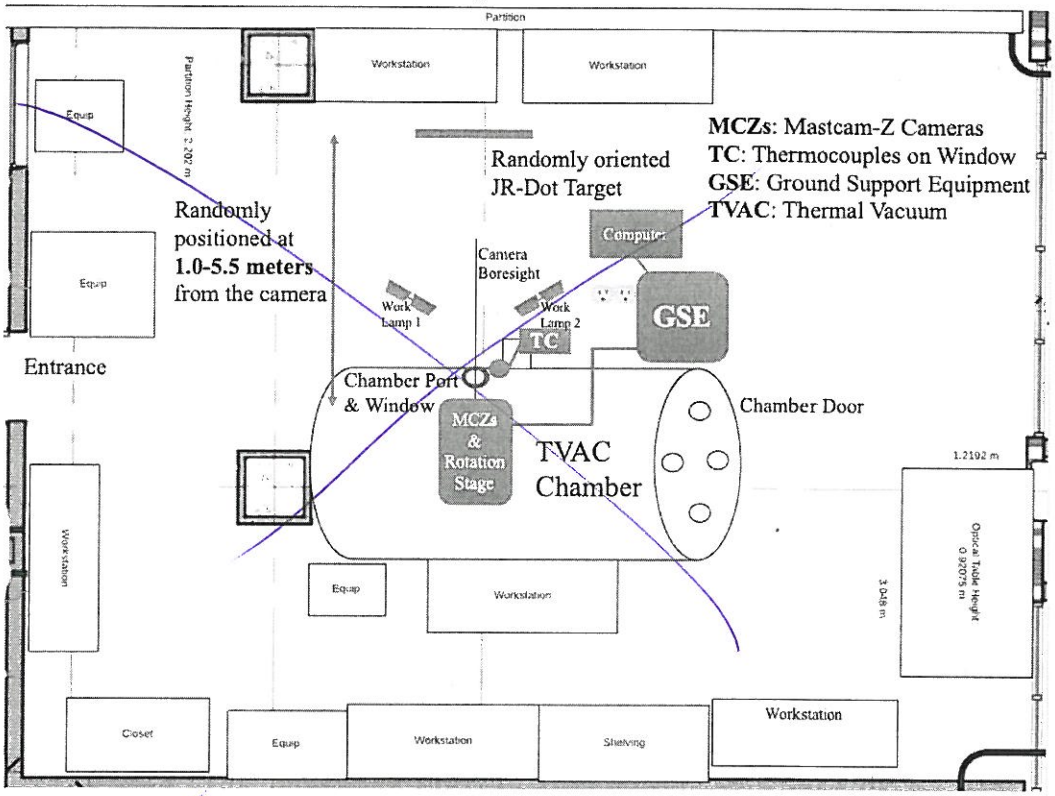

7. **[T,O, L]** ✓ Ensure that all personnel in the cleanroom are following the cleanroom practices for electrostatic discharge, proper clothing, and other safety concerns. See "ESD_Manual" and "Cleanroom_Manual". The duration is 2 minutes.
8. **[T]** ____ ~~Double check that nitrogen is flowing over the Mastcam-Zs or the window port.~~ skip, not required used air ionizer
9. **[O,T]** ____ ~~If not already done, mate the Right Mastcam-Z into the GSE.~~ done already
10. **[T]** ✓ Verify that the thermocouples are turned on and properly reading out.
11. **[T]** ✓ Install the lamps and position them about 1 meter from the geometric target out of the camera's field of view (FOV). Power them on.
12. **[O,T]** ✓ Ensure that the camera unit and GSE wires are secure, kink-free, and do not present tripping hazards when the lights are turned off.
13. **[O,D]** ✓ Check the camera temperature and ensure nominal operation. L: 25.6 °C R: 26.5 C
14. **[D]** ✓ Record the following environmental information:
    - Cleanroom temperature ~~26.~~ 25.6 °C pressure atm. humidity 48 % RH

Date 4/30 Time 10:06 Initials [illegible]

15. **[O,D, L]** Notes:

N/A

Table 2. The nominal target placement scenes for the geometric testing.

	**Target Placements**	**Notes**
**Scene 1**	Small MTF_SN005 (Side A) target at about 2 meters centered on the 63mm boresight	
**Scene 2**	Small MTF_SN005 (Side A) target at about 3 meters centered on the 100mm boresight	
**Scene 3**	Large MTF_SN007 (Side A) target at about 3 meters centered on the 34mm boresight	
**Scene 4**	Large MTF_SN007 (Side A) target at about 2 meters centered on the 26mm boresight	

Date 4/30 Time 10:08 Initials [illegible]

Scene 3 for the Left Mastcam-Z

SN 007

16. **[M,T]** Position the MTF target to Scene 3 as described in Table 2.
17. **[M]** Measure and record the location of the MTF target.

distance = 2.5 meters

18. **[D]** Record climate information:
    - TVAC temp 2 Port temp ______
    - Camera CCD temp L 25.6 °C ~~Optics~~ temp R 26.5 °C
19. **[D,T]** Take digital pictures of the setup and MTF target. ✓
20. **[O]** Capture a tests frame at 34mm with filter 0, and rsync data to the validator. Use prefix **471TAMBL00.** ✓
21. **[V,T]** Load the image in MTF Mapper to find the correct target position. Recapture an autofocused frame if necessary. ✓
22. **[O]** Load and execute camera script **491TAMBL05**, which autofocuses and captures 3 frames at 34mm focal length with filters 0 through 6. Insert note "TARGET=MTF_SN007". The estimated duration is 8 minutes. 10:18 AM ✓
23. **[D]** Record image names and parameters in Image Log. ✓
24. **[V]** Run fast-look script to verify that the required data were obtained. ✓
25. **[D, L]** Notes:

2 autos, + 2 manuals,
filter 3,4,5 shows faint contrast in center slanted edges

Test image: 9:41 AM, suffix 0 → reacquired with different autoexposure settings

Was: DN thres: 180
pix frac. 10%

IS: DN thres: 150
pix frac: 1%

O_2 sensor: 19.8 %

Temperature: 29.2°
final suffix: 27

final: 55 → still seeing MTF dropout in center of image, but appear minor.

Date 4/30 Time 10:08 Initials [illegible]

Scene 3 for the Right Mastcam-Z

SN 007

26. **[M,T]** Position the MTF target to Scene 3 as described in Table 2.
27. **[M]** Measure and record the location of the MTF target.

test image, suffix 0
distance = 2.7 meter

28. **[D]** Record climate information: RH % 51%
    - ~~TVAC~~ temp Room: 23.9°C ~~Port temp~~
    - Camera ~~CCD temp~~ L: 29.3°C ~~Optic~~s temp R: 29.3°C
29. **[D,T]** Take digital pictures of the setup and MTF target.
30. ✓ **[O]** Capture a tests frame at 34mm with filter 0, and rsync data to the validator. Use prefix **471TAMBR00.** 10:59 AM
31. ✓ **[V,T]** Load the image in MTF Mapper to find the correct target position. Recapture an autofocused frame if necessary.
32. ✓ **[O]** Load and execute camera script **491TAMBR05**, which autofocuses and captures 3 frames at 34mm focal length with filters 0 through 6. Insert note "TARGET=MTF_SN007". The estimated duration is 8 minutes. → with autoexpose mode
33. ✓ **[D]** Record image names and parameters in Image Log.
34. ✓ **[V]** Run fast-look script to verify that the required data were obtained.
35. **[D, L]** Notes:

filter 6 autofocus failed (out of range)
reacquiring filter 6 only (i
(suffix = 31)

Date 4/30 Time 11:54 AM Initials [illegible]

Scene 1 for the ~~Left~~ Right Mastcam-Z SN 005

36. **[M,T]** Position the MTF target to Scene 1 as described in Table 2.
37. **[M]** Measure and record the location of the MTF target.
distance = 1.4 meters
1.25 m.
2.1 meters from camera

38. **[D]** Record climate information:
    - ~~TVAC~~ Room temp 24.6 °C ~~Port temp~~ Room RH 49 %
    - Camera CCD temp R = 30.3 °C Optics ~~temp~~ N/A
39. ✓ **[D,T]** Take digital pictures of the setup and MTF target.
40. ✓ **[O]** Capture a tests frame at 63mm with filter 0, and rsync data to the validator. Use prefix **471TAMBL00.**
41. ✓ **[V,T]** Load the image in MTF Mapper to find the correct target position. Recapture an autofocused frame if necessary.
42. ✓ **[O]** Load and execute camera script **491TAMB~~L~~R06**, which autofocuses and captures 3 frames at 63mm focal length with filters 0 through 6. Insert note "TARGET=MTF_SN005". The estimated duration is 8 minutes.
43. ✓ **[V]** Run fast-look script ~~to~~ verify that the required data were obtained.
44. **[D, L]** Notes: validated

Time check: 12:08 PM

3 scenes out of 8

2 hours for 3 scenes

Date 4/30 Time 12:20 pm Initials [illegible]

Scene 1 for the ~~Right~~ Left Mastcam-Z   SN 005

45. **[M,T]** Position the MTF target to Scene 1 as described in Table 2.
46. **[M]** Measure and record the location of the MTF target.

target dot = 2.01 m

47. **[D]** Record climate information:
    - ~~TVAC~~ Room temp 25.0 °C ~~Port temp~~ Room RH 46%
    - Camera C~~CD temp~~ L = 29.7 °C O~~ptics temp~~ N/A
48. ✓ **[D,T]** Take digital pictures of the setup and MTF target.
49. ✓ **[O]** Capture a tests frame at 63mm with filter 0, and rsync data to the validator. Use prefix **471TAMB~~R~~L00.**
50. ✓ **[V,T]** Load the image in MTF Mapper to find the correct target position. Recapture an autofocused frame if necessary.
51. ✓ **[O]** Load and execute camera script **491TAMB~~R~~L06**, which autofocuses and captures 3 frames at 63mm focal length with filters 0 through 6. Insert note "TARGET=MTF_SN005". The estimated duration is 8 minutes.
52. ✓ **[V]** Run fast-look script to verify that the required data were obtained.
53. ✓ **[D, L]** Notes: Validated

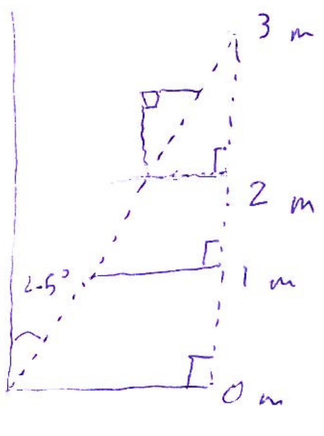

Date 4/29 Time 13:00 Initials gn

Scene 2 for the Left Mastcam-Z

54. **[M,T]** Position the MTF target to Scene 2 as described in Table 2.
55. **[M]** Measure and record the location of the MTF target.

 distance = 3.3 meters

56. **[D]** Record climate information: RH = 46 %
    - ~~TVAC~~ Room temp 25.0 °C ~~Port temp~~ ______
    - Camera CCD temp 29.8 °C Optics temp ______
57. **[D,T]** Take digital pictures of the setup and MTF target.
58. **[O]** Capture a tests frame at 100mm with filter 0, and rsync data to the validator. Use prefix **~~471~~ 491TAMBL00.**
59. **[V,T]** Load the image in MTF Mapper to find the correct target position. Recapture an autofocused frame if necessary.
60. **[O]** Load and execute camera script **~~4791~~ 491TAMBL07**, which autofocuses and captures 3 frames at 100mm focal length with filters 0 through 6. Insert note "TARGET=MTF_SN005". The estimated duration is 8 minutes.
61. **[D]** Record image names and parameters in Image Log.
62. **[V]** Run fast-look script to verify that the required data were obtained.
63. **[D, L]** Notes:

 filter 6 autofocus fail (432 nm)
 filter 6 " "

success suffix: 39

Date 4/30 Time 13:16 Initials [illegible]

Scene 2 for the Right Mastcam-Z

✓ 64. **[M,T]** Position the MTF target to Scene 2 as described in Table 2.

65. **[M]** Measure and record the location of the MTF target.

test image (13) 3.3 meters

✓ 66. **[D]** Record climate information:

- ~~TVAC~~ Room temp 24.8°C ~~Port temp~~ RH 46%
- ~~Camera CCD~~ R temp 30.2°C Optics temp ______

✓ 67. **[D,T]** Take digital pictures of the setup and MTF target.

✓ 68. **[O]** Capture a tests frame at 100mm with filter 0, and rsync data to the validator. Use prefix **471TAMBR00.**

✓ 69. **[V,T]** Load the image in MTF Mapper to find the correct target position. Recapture an autofocused frame if necessary.

✓ 70. **[O]** Load and execute camera script **491TAMBR07**, which autofocuses and captures 3 frames at 100mm focal length with filters 0 through 6. Insert note "TARGET=MTF_SN005". The estimated duration is 8 minutes.

✓ 71. **[D]** Record image names and parameters in Image Log.

✓ 72. **[V]** Run fast-look script to verify that the required data were obtained.

73. **[D, L]** Notes: validated

last suffix: 27

filters: 5 lower MTF → 975 nm
6 lower MTF → 1013 nm
long exposure times, higher dark current
→ re-run filters 5 & 6 with lights
repositioned lights closer to target
now 1.5 m away from target
last suffix: 35

Date 4/30 Time 14:00 Initials [illegible]

Time Check 1

IF MORE THAN 1.0 HOUR AHEAD OF SCHEDULED END, CONTINUE. OTHERWISE, SKIP TO THE SHUTDOWN PROCEDURE.

Scheduled End Time	Current Time	Time Ahead of Scheduled End
12:30 pm -	2:05 m =	N/A

74. **[D, L]** ✓ Record the time in the table above and determine if there is time for more testing

75. **[D, L]** Notes: defer Z-style testing to ambient cleanroom

Date ______ Time ______ Initials ______

Scene 3 Z-Stack for the Left Mastcam-Z

skip

76. **[M,T]** Position the MTF target to Scene 3 as described in Table 2.
77. **[M]** Measure and record the location of the MTF target.

____

____

____

78. **[D]** Record climate information:
    - TVAC temp ______________ Port temp ______________
    - Camera CCD temp ______________ Optics temp ______________
79. **[D,T]** Take digital pictures of the setup and MTF target.
80. **[O]** Capture a tests frame at 34mm with filter 0, and rsync data to the validator. Use prefix **471TAMBL00.**
81. **[V,T]** Load the image in MTF Mapper to find the correct target position. Recapture an autofocused frame if necessary.
82. **[O]** Autofocus once the target is properly placed.
83. **[O,V]** From the autofocus results, update the following script to capture a z-stack of 16 frames in 24 motor-count increments centered on the best focus position.
84. **[O]** Load and execute camera script **471TAMBL06**, which captures a z-stack of 16 frames at 34mm focal length with filter 0. Insert note "TARGET=MTF_SN007". The duration is 2 minutes.
85. **[D]** Record image names and parameters in Image Log.
86. **[V]** Run fast-look script to verify that the required data were obtained.
87. **[D, L]** Notes: ____

____

____

Date ______ Time ______ Initials ______

skip

Scene 3 Z-Stack for the Right Mastcam-Z

88. **[M,T]** Position the MTF target to Scene 3 as described in Table 2.
89. **[M]** Measure and record the location of the MTF target.

____

____

____

90. **[D]** Record climate information:
    - TVAC temp ______________ Port temp ______________
    - Camera CCD temp ______________ Optics temp ______________
91. **[D,T]** Take digital pictures of the setup and MTF target.
92. **[O]** Capture a tests frame at 34mm with filter 0, and rsync data to the validator. Use prefix **471TAMBR00.**
93. **[V,T]** Load the image in MTF Mapper to find the correct target position. Recapture an autofocused frame if necessary.
94. **[O]** Autofocus once the target is properly placed.
95. **[O,V]** From the autofocus results, update the following script to capture a z-stack of 16 frames in 24 motor-count increments centered on the best focus position.
96. **[O]** Load and execute camera script **471TAMBR06**, which captures a z-stack of 16 frames at 34mm focal length with filter 0. Insert note "TARGET=MTF_SN007". The duration is 2 minutes.
97. **[D]** Record image names and parameters in Image Log.
98. **[V]** Run fast-look script to verify that the required data were obtained.
99. **[D, L]** Notes: ____

____

____

Date 4/30 Time 14:07 Initials [signature]

Shutdown Procedure

skip

100. **[D,T]** ____ Take digital pictures of this page and the test setup.
101. **[D,O]** ✓ Review entries in Image Log, GSE command log, and image headers.
102. **[D, L]** ____ Review calibration procedure and ensure that each task is initialed.
103. **[D, L]** Notes: check marks in lieu of initials

104. **[V, L]** ✓ Before making the decision to break down the test setup, ensure that adequate data were acquired for the test requirements. See "MastcamZCalPlan" for these requirements.
105. **[V]** Notes: ______

Data Validator (signature) [signature]

Date 4/30/19 Time 2:08 PM

106. **[V, L]** ✓ Give the go/no-go decision. Have enough data been acquired to fulfill test requirements? See "MastcamZCalPlan" for these requirements.
107. **[D, L]** ____ Update the Log ~~Document~~. "as-run"
108. **[L]** Notes: ______

Calibration Lead (signature) [signature]

Date 4/30/19 Time 14:08

Date 4/30 Time 14:16 Initials [illegible]

mech. homed

109. **[O, L]** ✓ Ensure that the camera and GSE are in a safe state. camera heads powered
110. **[O,D]** ✓ Review the Image Log with the documentarian. Exchange high-fives.
111. **[O]** Notes: ______

Camera Operator (signature) [signature]

Date 4/30/19 Time 14:15

112. **[T]** ✓ If the next test does not require the target, position it away from the chamber or bench. Otherwise, be sure not to move it. The next test is JR
113. **[T]** ✓ Ensure that all other test equipment is safely put away.
114. **[T]** Notes: ______

Technician (signature) [signature]

Date 4-30-19 Time 2:15

115. **[D, L]** ✓ Double-check this procedure and ensure that the top of each page has valid data, time and initials.
116. **[D]** ____ Photo-scan this document, save it on the cloud, and file the hard-copy in the Log Binder. Upload the digital pictures taken during this test in the appropriate archive on the cloud. The required links are on the Wiki.
117. **[D]** ✓ Double-check that every required cell the Image Log is accurately filled. When this is complete, print the Image Log and file it the Log Binder after this document.
118. **[D]** Notes: ______

Documentarian (signature) [signature]

Date 4/30/19 Time 14:27

JR Geometric Calibration Procedure for the Right and Left Mastcam-Z Ambient TVAC Testing at MSSS (Pro. 4.6.5-7)

[Procedure version 2.06, prepared by the Mastcam-Z calibration team at Cornell University]

These measurements are performed on the camera and at the Temperature designated below as specified in the Mastcam-Z Calibration Plan,

Unit Under Test:

Left FM X **Right FM** X **EQM**____ **Other**________

These measurements are performed at Temperature:

-35°C____ **-10°C**____ **+5°C**____ **Ambient** X **Other**________

These measurements are performed at,

MSSS X **ASU**____ **Other**____

Date 4/30 **Start Time** 15:40 **End Time** 18:00

Estimated Duration 4.0 hours

Scheduled Start Time 15:00 **Sch. End Time** 19:00

Calibration Lead [L] HERKENHOFF **Documentarian [D]** WINHOLD, VAN BEEK

Camera Operator [O] VAN BEEK, TK, KW **Technician [T]** TATE

Data Validator [V] CORLIES **Metrologist [M]** N/A

Other ________

Change Log

Version	Name	Change
v1_01<br>26 Sep 2018	C. Tate	(first draft)
v1_07<br>1 Nov 2018	C. Tate	Procedure edits prior to EQM testing
V1_07-JR<br>8 Nov 2018	G. Paar	Distances more precisely reflected, change mode from v06 to v07 kept, fixed focus consistently at 2 tables & figure automatically referenced
v1_10<br>13 Dec. 2018	C. Tate	Procedure edits after EQM testing
v2_05<br>30 April 2019	C. Tate	Approved version prior to FM testing

Document Approval

Approved by James Bell Date
Mastcam-Z PI
Arizona State University

Approved by Alexander Hayes Date
Mastcam-Z Calibration Working Group
Lead, Cornell University

Approved by Justin Maki Date
Mastcam-Z Deputy PI and Investigation
Scientist, Jet Propulsion Laboratory

Approved by Christian Tate Date
Procedure Author
Cornell University

[signature] 4/30/19
Approved by: Date
KEN HERKENHOFF

Table of Contents

JR GEOMETRIC CALIBRATION PROCEDURE FOR THE RIGHT AND LEFT MASTCAM-Z AMBIENT TVAC TESTING AT MSSS (PRO. 4.6.5-7)....... 1

CHANGE LOG....... 2

DOCUMENT APPROVAL....... 2

TEST DESCRIPTION....... 4

SOFTWARE PREPARATION....... 4

Table 1. File naming convention for the camera script prefixes and frame filenames: "AAABBBBCDD"....... 4

HARDWARE INSTALLATION....... 6

Figure 1. ASU Floor Plan for Geometric Testing in the TVAC Chamber. The MSSS Floor Plan allows for similar target and source placements relative to the chamber window....... 6

FIXED TARGET POSITIONS FOR THE 63MM RIGHT AND LEFT MASTCAM-Z (SCENE 7)....... 8

DATA VALIDATION....... 14

TIME CHECK 1....... **ERROR! BOOKMARK NOT DEFINED.**

EXPLANATION OF THE SEMI-RANDOM ORIENTATIONS....... 8

Figure 2. An example of the camera's FOV (black) and JR dot target's semi-random positions (red)....... 8

100+ TARGET POSITIONS FOR THE 48MM RIGHT AND LEFT MASTCAM-ZS....... 8

DATA VALIDATION....... 10

SHUTDOWN PROCEDURE....... 15

Test Description

Excerpt from the Calibration Plan 4.6

> The objective of Geometric Calibration is to characterize the geometric distortion introduced by the Mastcam-Z optics into its images and measure the effective focal length and field of view at each focus and zoom position. As the range of zoom positions available to Mastcam-Z represent a continuum, measurements will be acquired at a finite number of zoom settings and then interpolated to characterize distortion and other geometric parameters across the full zoom range. Targets should be imaged at ~50% full well using the Bayer RGB/805 nm (priority 1) and remaining non-solar filters (priority 3). The calibration data will be used to generate a geometric model for each camera.

Software Preparation

The software and files required for this test are prepared in advance of test day. This checklist ensures that the following are present, debugged, and executable: (1) all fast look scripts, (2) automated header generation of all relevant camera parameters, target positioning, and metadata, (3) all camera scripts that command the camera unit, and (4) the directories/file-paths pointing to the data repositories of this specific test.

Table 1. File naming convention for the camera script prefixes and frame filenames: "AAABBBBCDD"

Code	Name	Example	Value(s)
"AAA"	Calibration Plan Section	"465" = Cal. Plan 4.6.5 chapter 4, section 6, subsection 5	465-7
"BBBB"	Location of test or ASU Chamber Temperature	"ATLO" = test at JPL ATLO, "TN10" = MSSS TVAC -10C, ...	TAMB
"C"	Camera unit under test	"L" = Left Mastcam-Z, "R" = Right Mastcam-Z, "E" =EQ "C" =COTS	L/R
"DD"	Part of test	"00" = test set up, "01" = first part,...	00-13

1. **[D]** [illegible] Look up the daily calibration schedule and record the scheduled start and end time of this test on the cover page of this document. Also, fill out and double-check the other information on the cover page.

2. **[D]** ____ Ensure that all supplemental manuals are on hand. These are,
    - Validator_Manual, Documentarian_Manual, MastcamZ_Data_Manual,
    - MastcamZCalPlan
3. **[D]** [illegible] Ensure that the Image Log is present and ready to use. Find and open the Google Sheets file "Image_Log_46". There is a link on the Wiki.
4. **[V]** [illegible] Check that all *Calgorithms* fast-look and validation scripts are present, up-to-date, and ready to analyze test output. Find and open the "Geometric_Calibration_46_Validation" Jupyter notebook. There is a link on the Wiki.
5. **[O]** [illegible] Check that all camera scripts required for this test are present, up-to-date and ready to command the ground support equipment (GSE). These are,
    - **465TAMBR01 - 465TAMBR09, 465TAMBL01 - 465TAMBL09**
    - **466TAMBR01 - 466TAMBR13, 466TAMBL01 - 466TAMBL13**
    - **467TAMBR01 - 467TAMBR04, 467TAMBL01 - 467TAMBL04**
6. **[O,V,D, L]** Notes:

Date 4/30/19 Time 15:51 Initials ZW

Hardware Installation

This procedure is for the ambient TVAC chamber testing at MSSS. Figure 1 shows the nominal layout of the TVAC chamber, workspace, Mastcam-Zs, ground support equipment (GSE), targets, sources, and other equipment necessary for this test if it happens at ASU. Although MSSS' cleanroom is different than ASU's, the placement of the targets and sources relative to the chamber window is similar.

Figure 1. ASU Floor Plan for Geometric Testing in the TVAC Chamber. The MSSS Floor Plan allows for similar target and source placements relative to the chamber window.

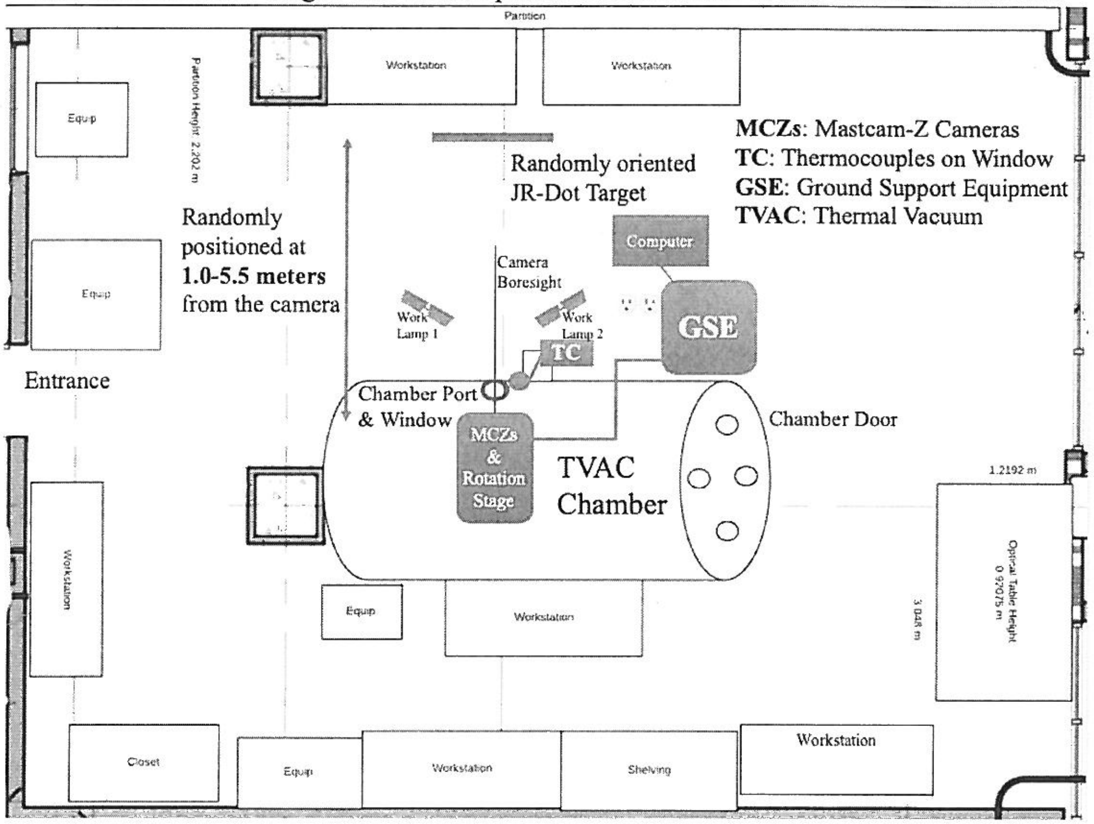

7. **[T,O, L]** ZW Ensure that all personnel in the cleanroom are following the cleanroom practices for electrostatic discharge, proper clothing, and other safety concerns. See "ESD_Manual" and "Cleanroom_Manual".
8. **[T]** ZW Double check that nitrogen is flowing over the Mastcam-Zs or the window port.

Date 4/30 Time ______ Initials Ec

9. **[O,T]** ____ If not already done, mate the Right Mastcam-Z into the GSE. Follow the procedure in "MastcamZ_GSE_Manual". SKIP
10. **[T]** Em Verify that the thermocouples are turned on and properly reading out.
11. **[T]** Ec Position the JR dot target approximately **3 meters** from the cameras
12. **[T]** Em Install the lamps and position them about 1 meter from the geometric target out of the camera's field of view (FOV). Power them on.
13. **[O,T]** Ec Ensure that the camera unit and GSE wires are secure, kink-free, and do not present tripping hazards when the lights are turned off.
14. **[O,D]** Ec Check the camera Temperature and ensure nominal operation.
15. **[D]** Ec Record the following environmental information:
    - Cleanroom Temperature 25.0 pressure _______ humidity 45%
16. **[O,D, L]** Notes:

L: 26.8 R: 27.4°C

Date 4/30 Time 16:00 Initials [illegible]

Explanation of the Semi-Random Orientations

Figure 2. An example of the camera's FOV (black) and JR dot target's semi-random positions (red)

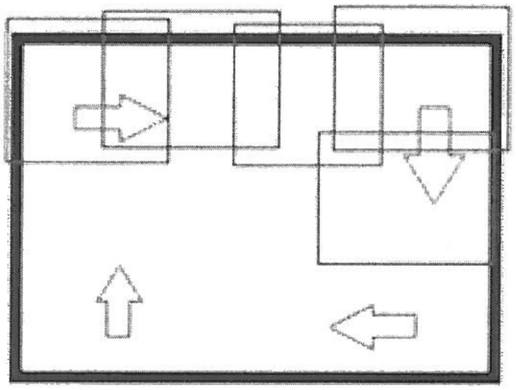

Figure 2 shows the desired orientations for the following tests that ask for a certain number of "semi-random orientations" of the JR dot target. The blue arrows show the motion of the target across the camera's FOV for optimal coverage. Note that some frames should be taken with the JR dot target rotated 90-degrees around the camera's line-of-sight.

100+ Target Positions for the 48mm Right and Left Mastcam-Zs

17. **[T]** [illegible] Position the JR dot target approximately **3 meters** from the camera. Adjust lights accordingly.
18. **[D]** [illegible] Record the following Temperatures:
    - Chamber Temp ________ Port Temp ________
    - Camera CCD Temp 26.8, 27.4°C Optics Temp ________
19. **[D,T]** [illegible] Take digital pictures of the geometric target's position, and the whole test/GSE set-up.
20. **[O,T]** [illegible] Capture test frames to find a standard exposure time for the 100 positions at 3 meters focus. Save these test frames with the prefix name **465TAMBR00**, and update "var1" in the script **465TAMBR03** once this exposure time is found. 143 ms
21. ✓ **[V,O,T]** Evaluate whether the target's dots are in-focus enough for discrimination. If the dots are too out-of-focus for JR's algorithm, move the target back.

22. **[O,T]** [illegible] Capture test frames to find a standard exposure time for the 100 positions at 3 meters focus. Save these test frames with the prefix name **465TAMBL00**, and update "var1" in the script **465TAMBL03** once this exposure time is found. 151 ms
23. **[V,O,T]** Evaluate whether the target's dots are in-focus enough for discrimination. If the dots are too out-of-focus for JR's algorithm, move the target back.

24. **[O,T]** [illegible] Load and begin the script **465TAMBR03**, which captures frames with filter 0 at **48mm** focal length one frame at a time, with a pause command between each frame.
25. **[O,T]** [illegible] Load and begin the script **465TAMBL03**, which captures frames with filter 0 at **48mm** focal length one frame at a time, with a pause command between each frame.

26. **[O,T]** [illegible] Capture **10 images** of the JR dot target in semi-random orientations (see Figure 2) normal to the camera approximately **1-meter** distance covering each edge of the camera's FOV.
27. **[O,T]** [illegible] Capture **20 images** of the JR dot target in semi-random orientations (see Figure 2) normal to the camera approximately **2-meter** distance covering each edge of the camera's FOV. Adjust lighting if necessary, to keep the frames at approximately 50% full-well.
28. **[O,T]** [illegible] Capture **20 images** of the JR dot target in semi-random orientations (see Figure 2) normal to the camera approximately **3-meter** distance covering each edge of the camera's FOV. Adjust lighting if necessary, to keep the frames at approximately 50% full-well. 9 IMAGES TAKEN AT 3.5 m
29. **[O,T]** [illegible] Capture **20 images** of the JR dot target in semi-random orientations (see Figure 2) normal to the camera approximately **4-meter** distance covering each edge of the camera's FOV. Adjust lighting if necessary, to keep the frames at approximately 50% full-well. NEED L SIDE OF RT EYE + R SIDE OF LEFT EYE.
30. **[O,T]** [illegible] Capture **30 images** of the JR dot target in semi-random orientations (see Figure 2) normal to the camera approximately **5-meter** distance covering each edge of the camera's FOV. Adjust lighting if necessary, to keep the frames at approximately 50% full-well.

Date 4/30 Time 17:20 Initials [illegible]

✓31. **[V,O,T]** Evaluate whether the target's dots are evenly distributed over each fields of view.

32. **[O,T, L]** [illegible] After more than 100 usable frames have been captured, stop the prefix script.

33. **[D]** [illegible] Record image names and parameters in Image Log.

34. **[D, L]** Notes: 144 MAX SUFFIX

Data Validation

35. **[V]** [illegible] Run the "Geometric_46_Validation" Jupyter notebook on the acquired data for for the Right and Left Mastcam-Zs. This analysis can take place while the test continues.

36. **[V,D, L]** Notes: COVERED ALL CORNERS, BOTH EYES

Date 4/30 Time 17:23 Initials [illegible]

Fixed Target Positions for the 63mm Right and Left Mastcam-Z (Scene 7)

37. **[T]** [illegible] Position the JR dot target approximately **3 meters** from the camera.
38. **[D]** [illegible] Record the following Temperatures:
    - Chamber Temp 29.8°C Port Temp ____________
    - Camera CCD Temp 29.9, 30.7°C Optics Temp ____________
39. **[D,T]** [illegible] Take digital pictures of the geometric target's position, and the whole test/GSE set-up.
40. **[O,T]** [illegible] Capture test frames to finely position the target centered in the 34mm FOV of both cameras. Save these test frames with the prefix name **466TAMBR00** and **466TAMBL00**.

SKIP {
41. **[M]** ____ Measure the locations of the geometric target and the camera.
42. **[M,D]** ____ Record the location measurements in the Image Log and tables below.

Target Location	Metrology ID#
Reference	
Top-Left Nest	
Top-Right Nest	
Bottom-Left Nest	
Bottom-Right Nest	

Camera/Chamber Location	Metrology ID#
Reference	0.38 m Camera-Port dist.
Nest 1	
Nest 2	
Nest 3	

43. **[M,D, L]** Notes: 2.08 m PORT-TARGET DISTANCE

BREAK DURING DEBUG LEFT CAMERA GUI

SKIP

44. ~~[O,T] ____ For **466TAMBL06/466TAMBR06** and **466TAMBL10/466TAMBR10**, delete the first four long distance filter positions of each 16 Z-stack.~~

Skipping until tomorrow

45. **[O,T]** ____ Load and execute the script **466TAMBL06**, which captures Z-stacks of 12 focus distances (from 1 meter to infinity) for filter 0 with seven focal lengths. The estimated duration is 10 minutes.
46. **[O,T]** ____ Load and execute the script **466TAMBR06**, which captures Z-stacks of 12 focus distances (from 1 meter to infinity) for filter 0 with seven focal lengths. The estimated duration is 10 minutes.
47. **[D]** ____ Record image names and parameters in Image Log.
48. **[D, L]** Notes:____

____

____

49. **[O,T]** ____ Load and execute the script **466TAMBL10**, which captures Z-stacks of 12 focus distances (from 1 meter to infinity) for each non-solar filter with the **63mm** focal length. The estimated duration is 10 minutes.
50. **[O,T]** ____ Load and execute the script **466TAMBR10**, which captures Z-stacks of 12 focus distances (from 1 meter to infinity) for each non-solar filter with the **63mm** focal length. The estimated duration is 10 minutes.
51. **[D]** ____ Record image names and parameters in Image Log.
52. **[D, L]** Notes:____

____

____

Date 4/30 Time 18:09 Initials CT

Skip

53. **[O,T]** ____ If time permits, load and execute the script **467TAMBL01**, which auto-exposes and captures frames for ~140 focal lengths with filter 0 at a focus distance of 3 meters. The estimated duration is 16 minutes.
54. **[O,T]** ____ If time permits, load and execute the script **467TAMBR01**, which auto-exposes and captures frames for ~140 focal lengths with filter 0 at a focus distance of 3 meters. The estimated duration is 16 minutes.
55. **[D]** ____ Record image names and parameters in Image Log.
56. **[D, L]** Notes:____

____

____

57. [T] ____ **Be sure that the target does not move. Ensure that proper warning is posted for the people who may come into the room in our absence. Post a DO NOT MOVE sign.**

Date 4/30 Time 6:10 Initials [illegible]

Data Validation

Skip

58. **[V]** ____ Run the "Geometric_46_Validation" Jupyter notebook on the acquired data for for the Right and Left Mastcam-Zs. This analysis can take place while the test continues.
59. **[V,D, L]** Notes: ______________________________

Date 4/30 Time 6:10 Initials [illegible]

Shutdown Procedure

60. **[D,T]** [illegible] Take digital pictures of the test setup.
61. **[D,O]** [illegible] Review entries in Image Log, GSE command log, and image headers.
62. **[D, L]** [illegible] Review calibration procedure and ensure that each task is initialed.
63. **[D, L]** Notes: __________

SKIP

64. **[V, L]** ____ ~~Before making the decision to break down the test setup, ensure that adequate data were acquired for the test requirements. See "MastcamZCalPlan" for these requirements.~~
65. **[V]** Notes: __________

Data Validator (signature) [illegible]

Date 4/30/19 Time 6:15 PM

66. **[V, L]** [illegible] Give the go/no-go decision. Have enough data been acquired to fulfill test requirements? See "MastcamZCalPlan" for these requirements.
67. **[D, L]** [illegible] Update the Log Document.
68. **[L]** Notes: PROCEDURE HALTED DUE TO PROBLEM WITH LEFT CAMERA GUI

Calibration Lead (signature) [illegible]

Date 4/30/19 Time 18:15

69. **[O, L]** TK Ensure that the camera and GSE are in a safe state.
70. **[O,D]** TK Review the Image Log with the documentarian. Exchange high-fives.
71. **[O]** Notes: ______

Camera Operator (signature) [signature]

Date 5/1/2019 Time 11:38

72. **[T]** CT If the next test does not require the target, position it away from the chamber or bench. Otherwise, be sure not to move it. The next test is JR geo. cal. (same)
73. **[T]** CT Ensure that all other test equipment is safely put away.
74. **[T]** Notes: ______

Technician (signature) Christian Tate

Date 4-30-19 Time 6:12

75. **[D, L]** [initials] Double-check this procedure and ensure that the top of each page has valid data, time and initials.
76. **[D]** ____ Photo-scan this document, save it on the cloud, and file the hard-copy in the Log Binder. Upload the digital pictures taken during this test in the appropriate archive on the cloud. The required links are on the Wiki. Skip
77. **[D]** ____ Double-check that every required cell the Image Log is accurately filled. When this is complete, print the Image Log and file it the Log Binder after this document. Skip
78. **[D]** Notes: ______

Documentarian (signature) [signature]

Date 4/30/19 Time 6:15

Date 5/1 Time 8:30 Initials [illegible]

JR Geometric Calibration Procedure for the Right and Left Mastcam-Z Ambient TVAC Testing at MSSS (Pro. 4.6.6)

[Procedure version 2.07, prepared by the Mastcam-Z calibration team at Cornell University]

These measurements are performed on the camera and at the Temperature designated below as specified in the Mastcam-Z Calibration Plan,

Unit Under Test:

Left FM X **Right FM** X **EQM**____ **Other**________

These measurements are performed at Temperature:

-35°C____ **-10°C**____ **+5°C**____ **Ambient** X **Other**________

These measurements are performed at,

MSSS X **ASU**____ **Other**____

Date 5/1/19 **Start Time** 8:30 **End Time** ________

Estimated Duration 3.0 hours

Scheduled Start Time ________ **Sch. End Time**________

Calibration Lead [L] Maki **Documentarian [D]** Christian, Megan (shadow)

Camera Operator [O] Elsa/Tex, Angela shadow **Technician [T]** Andy

Data Validator [V] Paul, Ole (shadow) **Metrologist [M]** N/A

Other ________

Change Log

Version	Name	Change
v1_01<br>26 Sep 2018	C. Tate	(first draft)
v1_07<br>1 Nov 2018	C. Tate	Procedure edits prior to EQM testing
V1_07-JR<br>8 Nov 2018	G. Paar	Distances more precisely reflected, change mode from v06 to v07 kept, fixed focus consistently at 2 tables & figure automatically referenced
v1_10<br>13 Dec. 2018	C. Tate	Procedure edits after EQM testing
v2_07<br>1 May 2019	C. Tate	Approved version prior to FM testing

Document Approval

Approved by James Bell Date
Mastcam-Z PI
Arizona State University

Approved by Alexander Hayes Date
Mastcam-Z Calibration Working Group Lead, Cornell University

[signature] 5/1/19
Approved by Justin Maki Date
Mastcam-Z Deputy PI and Investigation Scientist, Jet Propulsion Laboratory

[signature] 5/1/19
Approved by: Date
Ken Herkenhoff
Mastcam-Z Co-Investigator, USGS

[signature] 5/1/19
Approved by Christian Tate Date
Procedure Author
Cornell University

Table of Contents

JR GEOMETRIC CALIBRATION PROCEDURE FOR THE RIGHT AND LEFT MASTCAM-Z AMBIENT TVAC TESTING AT MSSS (PRO. 4.6.6)....... 1

CHANGE LOG....... 2
DOCUMENT APPROVAL....... 2
TEST DESCRIPTION....... 4
SOFTWARE PREPARATION....... 4
Table 1. File naming convention for the camera script prefixes and frame filenames: "AAABBBBCDD"....... 4
HARDWARE INSTALLATION....... 6
Figure 1. ASU Floor Plan for Geometric Testing in the TVAC Chamber. The MSSS Floor Plan allows for similar target and source placements relative to the chamber window....... 6
FIXED TARGET POSITIONS FOR THE 63MM RIGHT AND LEFT MASTCAM-Z (SCENE 7, WINDOW ON)....... 8
DATA VALIDATION....... 10
OPEN CHAMBER DOOR....... 10
FIXED TARGET POSITIONS FOR THE 63MM RIGHT AND LEFT MASTCAM-Z (SCENE 8, WINDOW OFF)....... 11
DATA VALIDATION....... 13
REMOVE TEST EQUIPMENT....... 13

SHUTDOWN PROCEDURE....... 14

Test Description

Excerpt from the Calibration Plan 4.6

> The objective of Geometric Calibration is to characterize the geometric distortion introduced by the Mastcam-Z optics into its images and measure the effective focal length and field of view at each focus and zoom position. As the range of zoom positions available to Mastcam-Z represent a continuum, measurements will be acquired at a finite number of zoom settings and then interpolated to characterize distortion and other geometric parameters across the full zoom range. Targets should be imaged at ~50% full well using the Bayer RGB/805 nm (priority 1) and remaining non-solar filters (priority 3). The calibration data will be used to generate a geometric model for each camera.

Software Preparation

The software and files required for this test are prepared in advance of test day. This checklist ensures that the following are present, debugged, and executable: (1) all fast look scripts, (2) automated header generation of all relevant camera parameters, target positioning, and metadata, (3) all camera scripts that command the camera unit, and (4) the directories/file-paths pointing to the data repositories of this specific test.

Table 1. File naming convention for the camera script prefixes and frame filenames: "AAABBBBCDD"

Code	Name	Example	Value(s)
"AAA"	Calibration Plan Section	"465" = Cal. Plan 4.6.5 chapter 4, section 6, subsection 5	466
"BBBB"	Location of test or ASU Chamber Temperature	"ATLO" = test at JPL ATLO, "TN10" = MSSS TVAC -10C, …	TAMB
"C"	Camera unit under test	"L" = Left Mastcam-Z, "R" = Right Mastcam-Z, "E" =EQ "C" =COTS	L/R
"DD"	Part of test	"00" = test set up, "01" = first part,…	00-13

1. **[D]** ✓ Look up the daily calibration schedule and record the scheduled start and end time of this test on the cover page of this document. Also, fill out and double-check the other information on the cover page.

Date 5/1 Time 8:48 Initials [illegible]

2. **[D]** ✓ Ensure that all supplemental manuals are on hand. These are,
   - ✓Validator_Manual, ✓Documentarian_Manual, ~~MastcamZ_Data_Manual~~,
   - MastcamZCalPlan✓
3. **[D]** ✓ Ensure that the Image Log is present and ready to use. Find and open the Google Sheets file "Image_Log_46". There is a link on the Wiki.
4. **[V]** ✓ Check that all *Calgorithms* fast-look and validation scripts are present, up-to-date, and ready to analyze test output. Find and open the "Geometric_Calibration_46_Validation" Jupyter notebook. There is a link on the Wiki.
5. **[O]** ✓ Check that all camera scripts required for this test are present, up-to-date and ready to command the ground support equipment (GSE). These are,
   - **466TAMBR01 - 466TAMBR13, 466TAMBL01 - 466TAMBL13**

6. **[O,V,D, L]** Notes:

Date 5/1 Time 8:54 Initials [illegible]

Hardware Installation

This procedure is for the ambient TVAC chamber testing at MSSS. Figure 1 shows the nominal layout of the TVAC chamber, workspace, Mastcam-Zs, ground support equipment (GSE), targets, sources, and other equipment necessary for this test if it happens at ASU. Although MSSS' cleanroom is different than ASU's, the placement of the targets and sources relative to the chamber window is similar.

map is out of date

Figure 1. ASU Floor Plan for Geometric Testing in the TVAC Chamber. The MSSS Floor Plan allows for similar target and source placements relative to the chamber window.

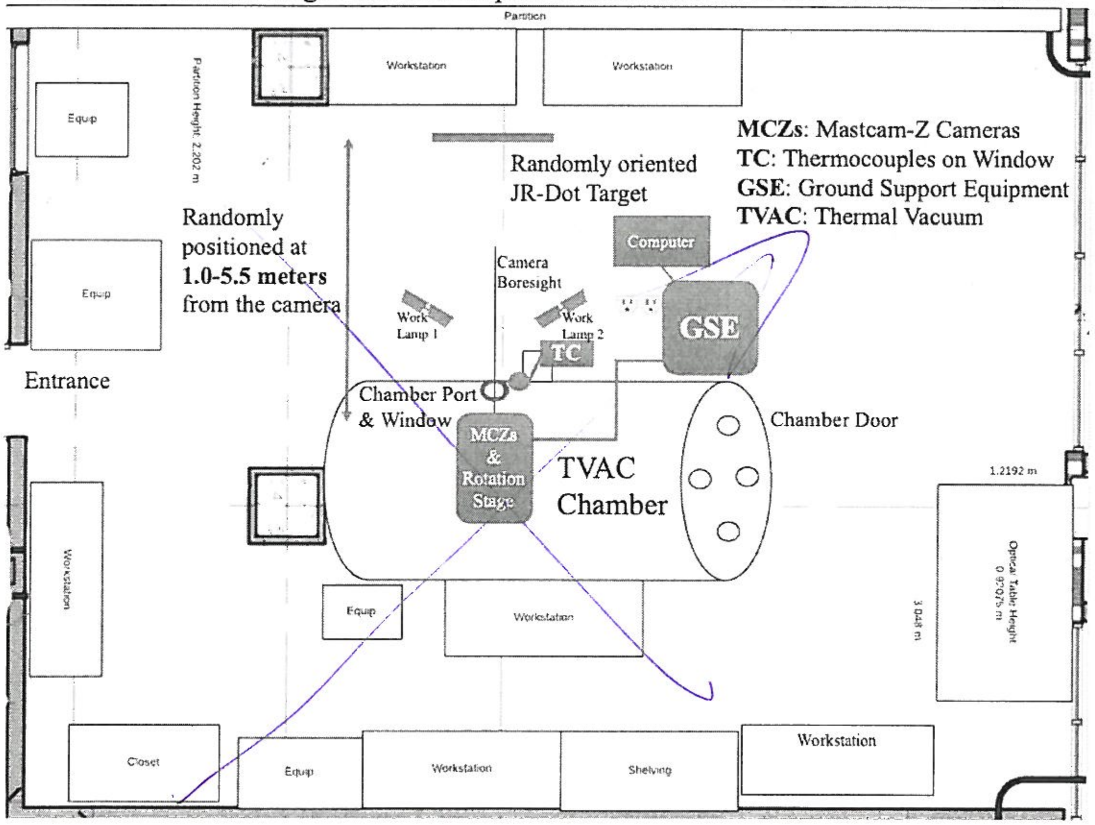

7. **[T,O, L]** ✓ Ensure that all personnel in the cleanroom are following the cleanroom practices for electrostatic discharge, proper clothing, and other safety concerns. See "ESD_Manual" and "Cleanroom_Manual".
8. **[T]** ✓ Double check that nitrogen is flowing over the Mastcam-Zs or the window port.

air ionizer

Date 5/1 Time 8:59 Initials [illegible]

skip → done

9. **[O,T]** ____ If not already done, mate the Right Mastcam-Z into the GSE. Follow the procedure in "MastcamZ_GSE_Manual".

Aux platen: 29.27 °C

10. **[T]** ✓ Verify that the thermocouples are turned on and properly reading out.
11. **[T]** ____ If necessary, position the JR dot target approximately **3 meters** from the cameras. done
12. **[T]** ✓ Place the Macbeth target in front of the JR dot target.
13. **[T]** ✓ Install the lamps and position them about 1 meter from the geometric target out of the camera's field of view (FOV). Power them on. Right Camera
14. **[O,T]** ✓ Ensure that the camera unit and GSE wires are secure, kink-free, and do not present tripping hazards when the lights are turned off.
15. **[O,D]** ✓ Check the camera Temperature and ensure nominal operation. L 29.4 °C R 30.2 °C
16. **[D]** ✓ Record the following environmental information:
    - Cleanroom Temperature 25.5 °C ~~pressure~~ n/a humidity 38%
17. **[O,D, L]** Notes:

____

____

____

Date 5/1 Time 9:30 Initials [illegible]

Fixed Target Positions for the 63mm Right and Left Mastcam-Z (Scene 7, window on)

18. **[T]** ____ ~~Position the JR dot target approximately **3 meters** from the camera.~~
19. **[D]** ____ Record the following ~~Temperatures:~~
    - ~~Chamber Temp ____________ Port Temp ____________~~
    - ~~Camera CCD Temp ____________ Optics Temp ____________~~
20. **[D,T]** ✓ Take digital pictures of the geometric target's position, and the whole test/GSE set-up.
21. **[O,T]** ✓ Capture test frames to finely position the target centered in the ~~34mm~~ FOV of both cameras. Save these test frames with the prefix name **466TAMBR00** and **466TAMBL00**. Make sure that the Macbeth target is fully visible and properly illuminated through the window (the whole target may have to move up). 48 mm suffix: 2
22. **[M]** ✓ Measure the locations of the geometric target and the camera.
23. **[M,D]** ✓ Record the location measurements in the Image Log and tables below.

Target Location	Metrology ID#
Reference	
Top-Left Nest	
Top-Right Nest	
Bottom-Left Nest	
Bottom-Right Nest	

Camera/Chamber Location	Metrology ID#
Reference	
Nest 1	
Nest 2	
Nest 3	

24. **[M,D, L]** Notes: distance = 1.63 cm from bar to target (Macbeth)

Sui

Date 5/1 Time 9:30 Initials [illegible]

25. **[O,T]** ✓ Load and execute the script **466TAMBL10** (9), which captures Z-stacks of 16 focus distances (from 1 meter to infinity) for each non-solar filter with the ~~**63mm**~~ (43) (34mm) focal length. The estimated duration is 10 minutes.

26. **[O,T]** ✓ Load and execute the script **466TAMBR10** (9), which captures Z-stacks of 16 focus distances (from 1 meter to infinity) for each non-solar filter with the ~~**63mm**~~ (34mm) focal length. The estimated duration is 10 minutes.

27. **[D]** ✓ Record image names and parameters in Image Log.

28. **[D, L]** Notes:____

29. **[T]** ✓ Remove the Macbeth target. Take note of the placement. ✓

L=29.9 °C

suffix: 118 R=30.7 °C

30. **[O,T]** ✓ Load and execute the script **466TAMBL06**, which captures Z-stacks of 16 focus distances (from 1 meter to infinity) for filter 0 with seven focal lengths. The estimated duration is 10 minutes.

31. **[O,T]** ✓ Load and execute the script **466TAMBR06**, which captures Z-stacks of 16 focus distances (from 1 meter to infinity) for filter 0 with seven focal lengths. The estimated duration is 10 minutes.

32. **[D]** ✓ Record image names and parameters in Image Log.

33. **[D, L]** Notes:____

suffix: 118 L = 30.1 °C

R = 30.8 °C

Data Validation

34. **[V]** ✓ Run the "Geometric_46_Validation" Jupyter notebook on the acquired data for for the Right and Left Mastcam-Zs. This analysis can take place while the test continues.
35. **[V,D, L]** Notes: ____________________

35.a. ✓ Power off camera heads

36. **[T]** ✓ **Be sure that the target and lamps do not move. Ensure that proper warning is posted for the people who may come into the room in our absence. Post a DO NOT MOVE sign.**

Open Chamber Door

37. **[O,T]** ✓ Call in the hardware team to open the door WITHOUT moving the target, lights, or Mastcam-Zs.
38. **[O,T, D, L]** Notes: Acquire image of setup 10:28 AM

Date 5/1 Time 10:32 Initials [illegible]

Fixed Target Positions for the 63mm Right and Left Mastcam-Z (Scene 8, window off)

39. **[O,T]** ✓ Load and execute the script **466TAMBL06**, which captures Z-stacks of 16 focus distances (from 1 meter to infinity) for filter 0 with seven focal lengths. Insert note "Window=0". The estimated duration is ~~10~~ 15 minutes.
40. **[O,T]** ✓ Load and execute the script **466TAMBR06**, which captures Z-stacks of 16 focus distances (from 1 meter to infinity) for filter 0 with seven focal lengths. Insert note "Window=0". The estimated duration is ~~10~~ 15 minutes.
41. **[D]** ✓ Record image names and parameters in Image Log.
42. **[D, L]** Notes: Validated per validator
    Note: "window=0" not recorded in image header

43. **[O,T]** ✓ If time permits, load and execute the script **466TAMBL07**, which seven focal lengths in reverse order (110, 100, …) for 3 meter focus with 0 filter. Insert note "Window=0". The estimated duration is 3 minutes.
44. **[O,T]** ✓ If time permits, load and execute the script **466TAMBR07**, which seven focal lengths in reverse order (110, 100, …) for 3 meter focus with 0 filter. Insert note "Window=0". The estimated duration is 3 minutes.
45. **[D]** ✓ Record image names and parameters in Image Log.
46. **[D, L]** Notes: final suffix = 6

L = 30.0° C
R = 30.8° C

✓ 46a. validated per validator

47. **[T]** ✓ Insert the Macbeth target in approximately the same place.

48. **[O,T]** ✓ Load and execute the script **466TAMBL10**, which captures Z-stacks of 16 focus distances (from 1 meter to infinity) for each non-solar filter with the **63mm** focal length. Insert note "Window=0". The estimated duration is 10 minutes.

49. **[O,T]** ✓ Load and execute the script **466TAMBR10**, which captures Z-stacks of 16 focus distances (from 1 meter to infinity) for each non-solar filter with the **63mm** focal length. Insert note "Window=0". The estimated duration is 10 minutes.

50. **[D]** ✓ Record image names and parameters in Image Log.

51. **[D, L]** Notes: Validated per validator

Data Validation

52. **[V]** ✓ Run the "Geometric_46_Validation" Jupyter notebook on the acquired data for for the Right and Left Mastcam-Zs. This analysis can take place while the test continues.
53. **[V,D, L]** Notes: validated
shadow on JR target

Remove Test Equipment

54. **[T]** ✓ Carefully remove the target, lights and other test equipment from the chamber room.
55. **[T, L]** Notes: ____

Date 5/1 Time 11:49 Initials [signature]

Shutdown Procedure

56. **[D,T]** ✓ Take digital pictures of the test setup. "tons" of pictures
√57. **[D,O]** ✓ Review entries in Image Log, GSE command log, and image headers.
58. **[D, L]** ✓ Review calibration procedure and ensure that each task is initialed.
59. **[D, L]** Notes: ____

60. **[V, L]** ✓ Before making the decision to break down the test setup, ensure that adequate data were acquired for the test requirements. See "MastcamZCalPlan" for these requirements.
61. **[V]** Notes: ____

Data Validator (signature) [signature]

Date 5/1/19 Time 11:35 AM

62. **[V, L]** ✓ Give the go/no-go decision. Have enough data been acquired to fulfill test requirements? See "MastcamZCalPlan" for these requirements.
63. **[D, L]** ____ Update the Log Document.
64. **[L]** Notes: ____

Calibration Lead (signature) [signature]

Date 5/1/19 Time 11:35 AM

Date 5/1 Time 11:49 Initials [illegible]

perform prior to step 56.

65. [O, L] ✓ Ensure that the camera and GSE are in a safe state.
66. [O,D] ✓ Review the Image Log with the documentarian. Exchange high-fives.
67. [O] Notes: ____________________

Camera Operator (signature) [signature]

Date 5/1/2019 Time 11:38

68. [T] ✓ If the next test does not require the target, position it away from the chamber or bench. Otherwise, be sure not to move it. The next test is going to be. in the cleanroom
69. [T] ✓ Ensure that all other test equipment is safely put away.
70. [T] Notes: None

Technician (signature) [signature]

Date 5/1/19 Time 11:50

71. [D, L] ✓ Double-check this procedure and ensure that the top of each page has valid data, time and initials.
72. [D] ____ Photo-scan this document, save it on the cloud, and file the hard-copy in the Log Binder. Upload the digital pictures taken during this test in the appropriate archive on the cloud. The required links are on the Wiki.
73. [D] ____ Double-check that every required cell the Image Log is accurately filled. When this is complete, print the Image Log and file it the Log Binder after this document.
74. [D] Notes: ____________________

Documentarian (signature) Megan Barrington

Date 1 May 2019 Time 12:33 pm

Photon Transfer Procedure for Mastcam-Z Ambient Testing at MSSS (Pro. 4.1.2)

[Procedure version 2.03, prepared by the Mastcam-Z calibration team at Cornell University]

These measurements are performed on the camera and at the temperature designated below as specified in the Mastcam-Z Calibration Plan,

Unit Under Test:

Left FM __X__ Right FM __X__ EQM_____ Other__________

These measurements are performed at temperature:

-35° C______ -10°C______ +5°C______ Ambient __X__ Other________

These measurements are performed at,

MSSS __X__ ASU______ Other________

Date 5/1/19 **Start Time** 18:40 **End Time** 20:40

Estimated Duration __5.0 hours__

Scheduled Start Time 16:00 **Sch. End Time** 21:00

Calibration Lead [L] HERKENHOFF **Documentarian [D]** CORLIES

Camera Operator [O] VAN BEEK, KW **Technician [T]** WINHOLD, MS

Data Validator [V] TATE, M **Other** ________

Change Log

Version	Name	Change
v1_01<br>4 June 2018	C. Tate	(first draft)
v1_27<br>30 Oct. 2018	C. Tate	Procedure edits prior to EQM testing
v1_29<br>1 Dec. 2018	C. Tate	Procedure edits after EQM testing
v2_03<br>1 May 2019	C. Tate	Approved version prior to FM testing

Document Approval

Approved by James Bell — Date
Mastcam-Z PI
Arizona State University

Approved by Alexander Hayes — Date
Mastcam-Z Calibration Working Group Lead, Cornell University

[signature] 5/1/19
Approved by Justin Maki — Date
Mastcam-Z Deputy PI and Investigation Scientist, Jet Propulsion Laboratory

[signature] 5/1/19
Approved by: — Date
Ken Herkenhoff
Mastcam-Z Co-Investigator, USGS

Christian Tate 1-5-19
Approved by Christian Tate — Date
Procedure Author
Cornell University

Table of Contents

PHOTON TRANSFER PROCEDURE FOR MASTCAM-Z AMBIENT TESTING AT MSSS (PRO. 4.1.2) **1**

CHANGE LOG 2
DOCUMENT APPROVAL 2
TEST DESCRIPTION 4
SOFTWARE PREPARATION 4
Table 1. File naming convention for the camera script prefixes and frame filenames: "AAABBBBCDD" *4*
HARDWARE INSTALLATION 6
Figure 1. ASU Floor Plan for Geometric Testing in the TVAC Chamber. The MSSS Floor Plan allows for similar target and source placements relative to the chamber window. *6*
Table 2. Exposure times in milliseconds for each integrating sphere radiance value in Table 3 .... *8*
Table 3. The nominal integrating sphere output radiance values. *8*

MASTCAM-Z TESTS **9**

DARK CURRENT WITH THE RIGHT AND LEFT MASTCAM-ZS 9
CENTER THE INTEGRATING SPHERE ON THE LEFT MASTCAM-Z 10
RADIANCE VALUE 1 FOR THE LEFT MASTCAM-Z 11
RADIANCE VALUE 2 FOR THE LEFT MASTCAM-Z 12
RADIANCE VALUE 3 FOR THE LEFT MASTCAM-Z 13
RADIANCE VALUE 4 FOR THE LEFT MASTCAM-Z 14
RADIANCE VALUE 5 FOR THE LEFT MASTCAM-Z 15
RADIANCE VALUE 6 FOR THE LEFT MASTCAM-Z 16
RADIANCE VALUE 7 FOR THE LEFT MASTCAM-Z 17
RADIANCE VALUE 8 FOR THE LEFT MASTCAM-Z 18
DATA VALIDATION 19
DARK CURRENT WITH THE RIGHT AND LEFT MASTCAM-ZS 20

SHUTDOWN PROCEDURE **21**

Date 5/1/19 Time 16:05 Initial [illegible]

Test Description

Excerpt from the Calibration Plan 4.2,

> The objectives of these tests are to derive flat field images as well as the coefficients to allow a conversion from reduced (bias, dark, and flat field corrected) DN/s to absolute radiometric response (W/cm^2/sr per filter) for (a) the R, G, and B microfilters of the Bayer Pattern Filter detectors in each camera head (clear filter), (b) the 14 non-solar Mastcam-Z spectral filters "Science Filters", and, if time permits, (c) the two Mastcam-Z neutral density solar filters; and to provide an estimate of the uncertainty in these coefficients and, at Priority 2, their temperature dependence. This test builds off the Section 4.3 – Spectral Throughput Calibration to accurately account for the filter spectral response in the conversion. The requirement of knowing the relative response on the shape of the spectral throughput to ±5% combined with the absolute Radiance accuracy of the integration sphere at ±5% still allows the ±10% absolute radiometric calibration requirement to be met.

Software Preparation

The software and files required for this test are prepared well in advance of test day. This checklist ensures that the following are present, debugged, and executable: (1) all fast-look scripts, (2) automated header generation of all relevant camera parameters, target positioning, and metadata, (3) all camera scripts that command the camera unit, and (4) the directories/file-paths pointing to the data repositories of this specific test.

Table 1. File naming convention for the camera script prefixes and frame filenames: "AAABBBBCDD"

Code	Name	Example	Value
"AAA"	Calibration Plan Section	"411" = Cal. Plan 4.1.1 chapter 4, section 1, subsection 1	412
"BBBB"	Location of test or ASU Chamber temperature	"MSSS" = test at MSSS, "TN10" = ASU TVAC -10C, …	TAMB
"C"	Camera unit under test	"L" = Left Mastcam-Z, "R" = Right Mastcam-Z, "E" =EQM, "C" =COTS	R/L
"DD"	Part of test (radiance value)	"00" = test set up, "01" = first radiance value …	00-08

1. **[D]** [illegible] Look up the daily calibration schedule and record the scheduled start and end time of this test on the cover page of this document. Also fill out and double-check the other information on the cover page.
2. **[D]** ____ Ensure that all supplemental manuals are on hand. These are,
   - Labsphere_Manual,
   - Validator_Manual, Documentarian_Manual
   - MastcamZCalPlan
3. **[D]** [illegible] Ensure that the Image Log is present and ready to use. Find and open the Google Sheets file "Image_Log_42". There is a link on the Wiki.
4. **[V]** [illegible] Check that all Calgorithms fast-look and validation scripts are present, up-to-date, and ready to analyze test output. Find and open the "Radiometric_Calibration_42_Validation" Jupyter notebook. There is a link on the Wiki.
5. **[O]** [illegible] Check that all camera scripts required for this test are present, up-to-date and ready to command the ground support equipment (GSE). These are,
   - **412TAMBR00 - 412TAMBR08**
   - **412TAMBL00 - 412TAMBL08**
   - **441TEMPR03, 441TEMPL03**
6. **[O,V,D, L]** Notes:

LATE START DUE TO CLEAN ROOM SETUP, METROLOGY WALKTHROUGH, INITIALIZATION

Date 5/1/19 Time 18:50 Initial [illegible]

Hardware Installation

This procedure is for the ambient TVAC chamber testing at MSSS. Figure 1 shows the nominal layout of the TVAC chamber, workspace, Mastcam-Zs, ground support equipment (GSE), targets, sources, and other equipment necessary for this test if it happens at ASU. Although MSSS' cleanroom is different than ASU's, the placement of the targets and sources relative to the chamber window is similar.

Figure 1. ASU Floor Plan for Geometric Testing in the TVAC Chamber. The MSSS Floor Plan allows for similar target and source placements relative to the chamber window.

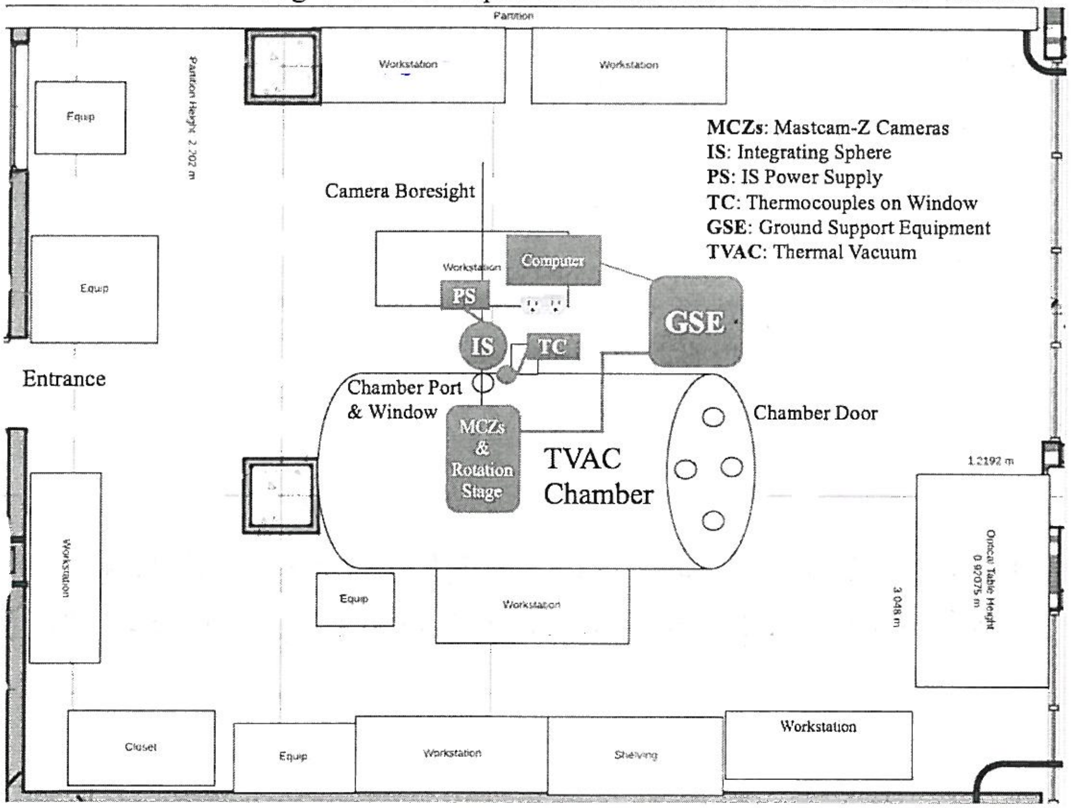

7. **[T,O, L]** ~~QL~~ Ensure that all personnel in the cleanroom are following the cleanroom practices for electrostatic discharge, proper clothing and other safety concerns.
8. ~~**[T]** ____ Double check that nitrogen is flowing over the Mas~~tcam-Zs. SKIP
9. **[T]** QL Verify that the thermocouples are turned on and properly reading out.
10. **[O,T]** QL Ensure that the camera unit and GSE wires are secure, kink-free, and do not present tripping hazards when the lights are turned off.

11. **[O,D]** [illegible] Check the camera temperature and ensure nominal operation. L 21.3, R 21.9°C
12. **[D]** [illegible] Record the following environmental information:
    - Cleanroom temperature 67.6°F pressure _______ humidity 55%
13. **[O,D, L]** Notes:

    LEFT CAMERA POWER OFF.

14. **[D,T]** [illegible] Take time-stamped pictures of the integrating sphere and the whole test/GSE set-up. RAN RT. CAMERA DARKCURRENT → STEPS 19,20,21,23
15. **[T]** [illegible] Power on the integrating sphere. Record the time the lamp is turned on 19:07. WARMUP 20 mins
16. **[D,T]** [illegible] Record the exact readout value of the integrating sphere's radiance: 1.0004 mW/cm2/sr.
17. ~~**[T,O, L]** ____ Confirm that the camera systems and GSEs are powered on and ready for use.~~ SKIP
18. **[D, L]** Notes:

    111 24.2° 132

Date 5/1 Time ______ Initial ______

Table 2. Exposure times in milliseconds for each integrating sphere radiance value in Table 3 at **100 mm focal length. The estimated duration is 13 minutes.** (Note that each sequence of exposure times begins and ends with a zero-time exposure in order to evaluate bias evolution.)

	**Time0**	**Time1**	**Time2**	**Time3**	**Time4**	**Time5**	**Time6**	**Time7**	**Time8**
**Radiance 1**	0.0	3.0	4.0	6.0	10.0	22.0	30.0	60.0	80.0
**Radiance 2**	0.0	3.0	4.0	6.0	10.0	14.0	22.0	40.0	80.0
**Radiance 3**	0.0	2.0	3.0	4.0	6.0	10.0	22.0	30.0	40.0
**Radiance 4**	0.0	2.0	3.0	4.0	6.0	10.0	14.0	22.0	30.0
**Radiance 5**	0.0	1.0	1.5	2.0	3.0	6.0	10.0	14.0	22.0
**Radiance 6**	0.0	0.5	1.0	2.0	3.0	4.0	6.0	10.0	14.0
**Radiance 7**	0.0	0.5	1.0	1.5	2.0	3.0	4.0	10.0	14.0
**Radiance 8**	0.0	0.5	1.0	1.5	2.0	2.5	3.0	6.0	10.0

Table 3. The nominal integrating sphere output radiance values.
If the pre-test reveals that a scaling is necessary, then the scaled radiance values are recorded here and used for the remainder of the photon transfer testing. (Note, these radiance values are in units of 1.0-2.4 micron band integrated spectral radiance mW/cm2/sr, true radiance is about a factor of two higher.)

**IS Output Radiances**	**Nominal Radiance [mW/cm2/sr]**	**Scaled Radiance [mW/cm2/sr]**
✓ Radiance 1	1.0	
Radiance 2	2.0	
Radiance 3	3.0	
Radiance 4	4.0	
✓ Radiance 5	5.0	
Radiance 6	6.0	
Radiance 7	8.0	
Radiance 8	10.0	

Mastcam-Z Tests

Dark Current with the ~~Right and Left~~ Mastcam-Zs

19. **[T]** Ea Turn off the lights and minimize the room's ambient light.
20. **[D]** Ea Record the following temperatures:
    - Camera CCD temp 21.9°C Optics temp ______
21. **[D,T]** Ea Take digital pictures of the integrating sphere's position and the whole test/GSE set-up. CAMERA POWERED ON
22. SKIP ~~**[O]** Load and execute camera script **441TEMPL03**, which captures 5 dark frames through filter 7 at the exposure times 0.0, 10.0, 20.0, and 100 seconds. The estimated duration is 12 minutes.~~
23. 2X **[O]** Load and execute camera script **441TEMPR03**, which captures 5 dark frames through filter 7 at the exposure times 0.0, 10.0, 20.0, and 100 seconds. The estimated duration is 12 minutes.
24. **[D]** Ea Record image names and parameters in Image Log.
25. **[T]** Ea Lights on.
26. **[D, L]** Notes: 24.5°C 1st
    25.1°C 2nd 154
    DIFFERENCE BETWEEN DARK FRAMES SHOWS NO SIGNIFICANT STRUCTURE AND AVERAGE ~~[illegible]~~ DARK CURRENT SIMILAR, CONSISTENT WITH SMALL CHANGE IN CCD TEMPERATURE.

Date 5/1/19 Time 19:29 Initial [initials]

Center the Integrating Sphere on the ~~Left~~ RIGHT Mastcam-Z

27. **[T]** [initials] Move the integrating sphere close to Mastcam-Z's boresight.
28. **[O]** [initials] Insert the note "ISOP=[radiance]" and execute camera script **423TAMB~~L~~R00**. OPEN EXIT PORT (REMOVE COVER)
    This script captures one auto-exposure at 40% full-well ~~and one bias frame~~ for filter 0 at 26 mm focal length. REMOVE EXIT PORT COVER, F.L.=79 mm,
29. **[V,O,T]** [initials] Open images, and if the images show that the integrating sphere is not centered, center the integrating sphere disc in the frame. Recapture **423TAMB~~L~~R00** frames if necessary. REPEATED @ 79mm, 100mm
30. **[D]** [initials] Record image names and parameters in the image Log.
31. **[T]** [initials] Take digital pictures of the integrating sphere's position and the whole test/GSE set-up.
32. **[T]** [initials] Visually estimate the distance from the integrating sphere and the Mastcam-Z's sunshade. This distance is approximately 36 cm.
33. **[T]** [initials] Lights off
34. **[D, L]** Notes:
    24.8°C, 25.0°C, 25.2°C, 25.3°C
    → 12
    SPHERE WAS LOWERED TO 19 7/8" ABOVE FLOOR TO CENTER EXIT PORT IN FOV.

Date 5/1/19 Time 19:56 Initial [illegible]

Radiance Value 1 for the ~~Left~~ RIGHT Mastcam-Z

35. **[D]** [illegible] Record temperature information:
    - Camera CCD temp 25.0°C Optics temp ______________
36. **[D,T]** [illegible] Set the integrating sphere output to this test's radiance value defined in Table 3.
37. **[D,T]** [illegible] Record exact integrating sphere readout value 1.0079 mW/cm2/sr.
38. SKIP ~~**[T]** ____ Take~~ time-stamped digital pictures of the setup and integrating sphere rea~~dout~~. LIGHTS OUT
39. **[O]** [illegible] Insert the note "ISOP=[radiance]" and execute camera script **412TAMB~~L~~R01**, which captures 10 frames for 9 exposure times with filter 0 at focus 3 m at 100mm focal length. The estimated duration is ~~22~~ 9 minutes.
40. **[D,T]** [illegible] Record exact integrating sphere readout value 1.0031 mW/cm2/sr.
41. **[D]** [illegible] Record image names and parameters in the Image Log.
42. **[D, L]** Notes: ~~[illegible]~~ 25.0°C
    → 89

Radiance Value 2 for the Left Mastcam-Z

43. **[D]** ____ Record temperature information:
    - Camera CCD temp _______________ Optics temp _______________
44. **[D,T]** ____ Set the integrating sphere output to this test's radiance value defined in Table 3.
45. **[D,T]** ____ Record exact integrating sphere readout value __________ mW/cm2/sr.
46. **[T]** ____ Take time-stamped digital pictures of the setup and integrating sphere readout.
47. **[O]** ____ Insert the note "ISOP=[radiance]" and execute camera script **412TAMBL02**, which captures 10 frames for 9 exposure times with filter 0 at focus 3 m at 100mm focal length. The estimated duration is 22 minutes.
48. **[D,T]** ____ Record exact integrating sphere readout value __________ mW/cm2/sr.
49. **[D]** ____ Record image names and parameters in the Image Log.
50. **[D, L]** Notes: ____

____

____

SKIP

Radiance Value 3 for the Left Mastcam-Z

51. **[D]** ____ Record temperature information:
    - Camera CCD temp ______________ Optics temp ______________
52. **[D,T]** ____ Set the integrating sphere output to this test's radiance value defined in Table 3.
53. **[D,T]** ____ Record exact integrating sphere readout value __________ mW/cm2/sr.
54. **[T]** ____ Take time-stamped digital pictures of the setup and integrating sphere readout.
55. **[O]** ____ Insert the note "ISOP=[radiance]" and execute camera script **412TAMBL03**, which captures 10 frames for 9 exposure times with filter 0 at focus 3 m at 100mm focal length. The estimated duration is 22 minutes.
56. **[D,T]** ____ Record exact integrating sphere readout value __________ mW/cm2/sr.
57. **[D]** ____ Record image names and parameters in the Image Log.
58. **[D, L]** Notes: ____

____

____

SKIP

Date ______ Time ______ Initial ______

Radiance Value 4 for the Left Mastcam-Z

59. **[D]** ____ Record temperature information:
    - Camera CCD temp ______________ Optics temp ______________
60. **[D,T]** ____ Set the integrating sphere output to this test's radiance value defined in Table 3.
61. **[D,T]** ____ Record exact integrating sphere readout value __________ mW/cm2/sr.
62. **[T]** ____ Take time-stamped digital pictures of the setup and integrating sphere readout.
63. **[O]** ____ Insert the note "ISOP=[radiance]" and execute camera script **412TAMBL04**, which captures 10 frames for 9 exposure times with filter 0 at focus 3 m at 100mm focal length. The estimated duration is 22 minutes.
64. **[D,T]** ____ Record exact integrating sphere readout value __________ mW/cm2/sr.
65. **[D]** ____ Record image names and parameters in the Image Log.
66. **[D, L]** Notes: ____

____

____

SKIP

Date 5/1/19 Time 20:00 Initial [illegible]

Radiance Value 5 for the ~~Left~~ RIGHT Mastcam-Z

67. **[D]** [initials] Record temperature information:
    - Camera CCD temp 25.0 Optics temp ______________
68. **[D,T]** [initials] Set the integrating sphere output to this test's radiance value defined in Table 3.
69. **[D,T]** [initials] Record exact integrating sphere readout value 5.0013 mW/cm2/sr.
70. ~~[T] ____ Take time-stamped~~ digital pictures of the ~~setup and~~ integrating sphere readout.
71. SKIP [~~O~~] [initials] ~~Insert the note~~ "ISOP=[radiance]" STET and execute camera script **412TAMB~~L~~R05**, which captures 10 frames for 9 exposure times with filter 0 at focus 3 m at 100mm focal length. The estimated duration is ~~22~~ 9 minutes.
72. **[D,T]** [initials] Record exact integrating sphere readout value 5.0088 mW/cm2/sr.
73. **[D]** [initials] Record image names and parameters in the Image Log.
74. **[D, L]** Notes: TURN OFF SPHERE
    25.1°C 9179

Date ______ Time ______ Initial ______

Radiance Value 6 for the Left Mastcam-Z

75. **[D]** ____ Record temperature information:
    - Camera CCD temp ______________ Optics temp ______________
76. **[D,T]** ____ Set the integrating sphere output to this test's radiance value defined in Table 3.
77. **[D,T]** ____ Record exact integrating sphere readout value __________ mW/cm2/sr.
78. **[T]** ____ Take time-stamped digital pictures of the setup and integrating sphere readout.
79. **[O]** ____ Insert the note "ISOP=[radiance]" and execute camera script **412TAMBL06**, which captures 10 frames for 9 exposure times with filter 0 at focus 3 m at 100mm focal length. The estimated duration is 22 minutes.
80. **[D,T]** ____ Record exact integrating sphere readout value __________ mW/cm2/sr.
81. **[D]** ____ Record image names and parameters in the Image Log.
82. **[D, L]** Notes: ____

____

____

SKIP

Date ______ Time ______ Initial ______

Radiance Value 7 for the Left Mastcam-Z

83. **[D]** ____ Record temperature information:
    - Camera CCD temp _______________Optics temp _______________
84. **[D,T]** ____ Set the integrating sphere output to this test's radiance value defined in Table 3.
85. **[D,T]** ____ Record exact integrating sphere readout value __________ mW/cm2/sr.
86. **[T]** ____ Take time-stamped digital pictures of the setup and integrating sphere readout.
87. **[O]** ____ Insert the note "ISOP=[radiance]" and execute camera script **412TAMBL07**, which captures 10 frames for 9 exposure times with filter 0 at focus 3 m at 100mm focal length. The estimated duration is 22 minutes.
88. **[D,T]** ____ Record exact integrating sphere readout value __________ mW/cm2/sr.
89. **[D]** ____ Record image names and parameters in the Image Log.
90. **[D, L]** Notes: ____

____

____

SKIP

Date ______ Time ______ Initial ______

Radiance Value 8 for the Left Mastcam-Z

SKIP

91. **[D]** ____ Record temperature information:
    - Camera CCD temp ______________ Optics temp ______________
92. **[D,T]** ____ Set the integrating sphere output to this test's radiance value defined in Table 3.
93. **[D,T]** ____ Record exact integrating sphere readout value __________ mW/cm2/sr.
94. **[T]** ____ Take time-stamped digital pictures of the setup and integrating sphere readout.
95. **[O]** ____ Insert the note "ISOP=[radiance]" and execute camera script **412TAMBL08**, which captures 10 frames for 9 exposure times with filter 0 at focus 3 m at 100mm focal length. The estimated duration is 22 minutes.
96. **[D,T]** ____ Record exact integrating sphere readout value __________ mW/cm2/sr.
97. **[D]** ____ Record image names and parameters in the Image Log.
98. **[D, L]** Notes: ____

____

____

Date 5/1/19 Time 20:19 Initial Zu

Data Validation

99. **[V]** ✓ Zu Upload data to server.

100. **[V]** Zu Run the Photon Transfer Jupyter notebook on the acquired data for the Right and Left Mastcam-Z. This analysis can take place while the test continues.

- Create preliminary photon transfer curves.
- Create preliminary flat-field images and radiometric coefficients for each filter.
- Save results in the calibration records.

101. **[V,D, L]** Notes: GAIN = 16.0 e⁻/DN, READ NOISE = 21.7 e⁻

Date 5/1/19 Time 20:15 Initial [illegible]

Dark Current with the Right ~~and Left~~ Mastcam-Zs

102. **[T]** [illegible] Turn off the lights and minimize the room's ambient light.
103. **[D]** [illegible] Record the following temperatures:
    - Camera CCD temp ~~25.8°C~~ 24.8 Optics temp ______________

SKIP 104. **[D,T]** ____ ~~Take digital~~ pictures of the integrating sphere's position, and the whole test/GSE set-up.

SKIP 105. **[O]** Load and execute camera script **441TEMPL03**, which captures 5 dark frames through filter 7 at the exposure times 0.0, 10.0, 20.0, and 100.0 seconds. The estimated duration is 12 minutes.

✓ 106. **[O]** Load and execute camera script **441TEMPR03**, which captures 5 dark frames through filter 7 at the exposure times 0.0, 10.0, 20.0, and 100.0 seconds. The estimated duration is 12 minutes.

107. **[D]** [illegible] Record image names and parameters in Image Log.
108. **[T]** [illegible] ~~Un~~cover the port window.
109. **[D, L]** Notes: TURN LIGHTS ON IN ROOM
25.1°C
→ 176

Date 5/1/19 Time 20:33 Initial [illegible]

Shutdown Procedure

110. **[D,T]** [illegible] Take digital pictures of this page and the test setup.
111. **[D,O]** [illegible] Review entries in Image Log, GSE command log, and image headers.
112. **[D, L]** [illegible] Review calibration procedure and ensure that each task is initialed.
113. **[D, L]** Notes: THE SPHERE WAS LOWERED TO 19 7/8" ABOVE FLOOR TO CENTER IN FOV

114. **[V, L]** [illegible] Before making the decision to break down the test setup, ensure that adequate data were acquired for the test requirements. See "MastcamZCalPlan" for these requirements.
115. **[V]** Notes: All good

Data Validator (signature) [signature]

Date 5-1-19 Time 8:40 (20:40)

116. **[V, L]** [illegible] Give the go/no-go decision. Have enough data been acquired to fulfill test requirements? See "MastcamZCalPlan" for these requirements.
117. **[D, L]** [illegible] Update the Log Document.
118. **[L]** Notes: SOME OF US HAVE BEEN HERE SINCE 7:30 THIS MORNING, SO WE QUIT BEFORE 9PM.

Calibration Lead (signature) [signature]

Date 5/1/19 Time 20:50

119. **[O, L]** JVB Ensure that the camera and GSE are in a safe state.
120. **[O,D]** JVB Review the Image Log with the documentarian. Exchange high-fives.
121. **[O]** Notes: Nothing of note.

Camera Operator (signature) [signature]

Date 5-1-19 Time 9:08 pm

122. **[T]** AW If the next test does not require the integrating sphere, position it away from the chamber or bench. Otherwise, be sure not to move it. The next test is METROLOGY
123. **[T]** AW Ensure that all other test equipment is safely put away.
124. **[T]** Notes: KAPTON TAPE MARKS FEET OF INT. SPHERE CART PLACEMENT NEAR CH3.

Technician (signature) [signature]

Date 5/1/19 Time 21:03

125. **[D, L]** PMC Double-check this procedure and ensure that the top of each page is initialed with the time and date.
126. **[D]** PMC Photo-scan this document, save it on the cloud, and file the hardcopy in the Log Binder. Upload the digital pictures taken during this test in the appropriate archive on the cloud. The required links are on the Wiki.
127. **[D]** PMC Double-check that every required cell the Image Log is accurately filled. When this is complete, print the Image Log and file it the Log Binder after this document.
128. **[D]** Notes:

Documentarian (signature) [signature]

Date 5/2/19 Time 8:37AM
