# Supplementary material for: Pre-Flight Calibration of the Mars 2020 Rover Mastcam Zoom (Mastcam-Z) Multispectral, Stereoscopic Imager
Source: Space Sci Rev. 2021 Feb 18;217(2):29. doi: 10.1007/s11214-021-00795-x (PMC7892537; doi:10.1007/s11214-021-00795-x)
Supplement: Supplementary file 1 — (ZIP 98.6 MB) [file 11214_2021_795_MOESM1_ESM.zip › CalPro_462_Geometric_v2_02.pdf]

Date 5/2 Time 8:30 Initials [illegible]

Geometric Calibration Procedure for Mastcam-Z Ambient Testing at MSSS (Pro. 4.6.2)

[Procedure version 2.~~01~~ 2.02, prepared by the Mastcam-Z calibration team at Cornell University]

These measurements are performed on the camera and at the temperature designated below as specified in the Mastcam-Z Calibration Plan,

Unit Under Test:

Left FM X **Right FM** X **EQM**____ **Other**____

These measurements are performed at temperature:

-35°C____ **-10°C**____ **+5°C**____ **Ambient** X **Other**____

These measurements are performed at,

MSSS X **ASU**____ **Other**____

Date 5/2/2019 **Start Time** 8:30am **End Time** 5/3/19 14:40

Estimated Duration 8.0 hours

Scheduled Start Time 8:30am **Sch. End Time** 4:30pm (16:30)

Calibration Lead [L] Justin Maki **Documentarian [D]** [illegible], Christian Tate

Camera Operator [O] Tex, Elsa **Technician [T]** Andy, Christian Tate

Data Validator [V] Paul, Ole. **Metrologist [M]** Mark Thompson

Other ____

Date 5/2 Time 8:30 Initials [illegible]

Change Log

Version	Name	Change
v1_01<br>25 Sep 2018	C. Tate	(first draft)
v1_07<br>1 Nov 2018	C. Tate	Procedure edits prior to EQM testing
v1_10<br>10 Dec. 2018	C. Tate	Procedure edits after EQM testing
v2_0~~1~~2<br>2 May, 2019	C. Tate	Approved version prior to FM testing

Document Approval

5/6/19
Approved by James Bell — Date
Mastcam-Z PI
Arizona State University

5/6/19
Approved by Alexander Hayes — Date
Mastcam-Z Calibration Working Group Lead, Cornell University

5/2/19
Approved by Justin Maki — Date
Mastcam-Z Deputy PI and Investigation Scientist, Jet Propulsion Laboratory

5/2/19
Approved by: — Date
Ken Herkenhoff
Mastcam-Z Co-Investigator, USGS

5/2/19
Approved by Christian Tate — Date
Procedure Author
Cornell University

Table of Contents

GEOMETRIC CALIBRATION PROCEDURE FOR MASTCAM-Z AMBIENT TESTING AT MSSS (PRO. 4.6.2) 1

CHANGE LOG 2
DOCUMENT APPROVAL 2
TEST DESCRIPTION 5
SOFTWARE PREPARATION 6
Table 1. File naming convention for the camera script prefixes and frame filenames: "AAABBBBCDD" 6
HARDWARE INSTALLATION 8
Figure 1. MSSS Floor Plan for Geometric Testing in the cleanroom. 8
Table 2. The nominal target positions for autofocusing 9

LEFT AND RIGHT MASTCAM-Z TESTS 10

26MM FOCAL LENGTH 1 FOR THE MASTCAM-ZS 10
Best Focus Model 10
Focal Length 1 and Focus 1 11
Focal Length 1 and Focus 2 12
Focal Length 1 and Focus 3 13
Focal Length 1 and Focus 4 14
Focal Length 1 and Focus 5 15
DATA VALIDATION 15
34MM FOCAL LENGTH 2 FOR THE MASTCAM-ZS 16
Best Focus Model 16
Focal Length 2 and Focus 1 17
Focal Length 2 and Focus 2 18
Focal Length 2 and Focus 3 19
Focal Length 2 and Focus 4 20
Focal Length 2 and Focus 5 21
DATA VALIDATION 21
48MM FOCAL LENGTH 3 FOR THE MASTCAM-ZS 22
Best Focus Model 22
Focal Length 3 and Focus 1 23
Focal Length 3 and Focus 2 24
Focal Length 3 and Focus 3 25
Focal Length 3 and Focus 4 26
Focal Length 3 and Focus 5 27
DATA VALIDATION 27
63MM FOCAL LENGTH 4 FOR THE MASTCAM-ZS 28
Best Focus Model 28
Focal Length 4 and Focus 1 29
Focal Length 4 and Focus 2 30
Focal Length 4 and Focus 3 31
Focal Length 4 and Focus 4 32
Focal Length 4 and Focus 5 33
DATA VALIDATION 33
79MM FOCAL LENGTH 5 FOR THE MASTCAM-ZS 34
Best Focus Model 34
Focal Length 5 and Focus 1 35

Focal Length 5 and Focus 2 .... *36*
Focal Length 5 and Focus 3 .... *37*
Focal Length 5 and Focus 4 .... *38*
Focal Length 5 and Focus 5 .... *39*
DATA VALIDATION 39
100MM FOCAL LENGTH 6 FOR THE MASTCAM-ZS 40
Best Focus Model *40*
Focal Length 6 and Focus 1 .... *41*
Focal Length 6 and Focus 2 .... *42*
Focal Length 6 and Focus 3 .... *43*
Focal Length 6 and Focus 4 .... *44*
Focal Length 6 and Focus 5 .... *45*
DATA VALIDATION 45
110MM FOCAL LENGTH 7 FOR THE MASTCAM-ZS 46
Best Focus Model *46*
Focal Length 7 and Focus 1 .... *47*
Focal Length 7 and Focus 2 .... *48*
Focal Length 7 and Focus 3 .... *49*
Focal Length 7 and Focus 4 .... *50*
Focal Length 7 and Focus 5 .... *51*
DATA VALIDATION 51
FIXED TARGET MECH CYCLING FOR THE MASTCAM-ZS 52
SHUTDOWN PROCEDURE **54**

Date 5/2 Time 8:30 Initials [illegible]

Test Description

Excerpt from the Calibration Plan 4.6

The objective of Geometric Calibration is to characterize the geometric distortion introduced by the Mastcam-Z optics into its images, and measure the effective focal length and field of view at each focus and zoom position. As the range of zoom positions available to Mastcam-Z represent a continuum, measurements will be acquired at a finite number of zoom settings and then interpolated to characterize distortion and other geometric parameters across the full zoom range. Targets should be imaged at ~50% full well using the Bayer RGB/805 nm (priority 1) and remaining non-solar filters (priority 3). The calibration data will be used to generate a geometric model for each camera. The camera models may exhibit wavelength dependence, so an attempt to measure the effect over all filters is desired (although not required).

Priority 2 and 3 tests will be performed at ASU utilizing both the MSL heritage technique as well as photogrammetric techniques and software developed for industrial/commercial applications. For most focus distances, images of an array of coded targets are to be obtained at 2 or more different translational camera locations to provide highly convergent image pairs. For near-field (2–3 meter) focus distances, a meter-scale test artifact with coded targets will be rotated about both horizontal and vertical axes to simulate the required convergent imaging geometry. In all cases, at each camera location, two images must be acquired: one with zero rotation and one rotated approximately 90° about the boresight. The rotated image is required to resolve known correlation between camera parameters. The rotation will be achieved by mounting the camera on a bracket attached to a heavy-duty rotation stage.

Date 5/2 Time 9:00 Initials [illegible]

Software Preparation

The software and files required for this test are prepared in advance of test day. This checklist ensures that the following are present, debugged, and executable: (1) all fast look scripts, (2) automated header generation of all relevant camera parameters, target positioning, and metadata, (3) all camera scripts that command the camera unit, and (4) the directories/file-paths pointing to the data repositories of this specific test.

Table 1. File naming convention for the camera script prefixes and frame filenames: "AAABBBBCDD"

Code	Name	Example	Value(s)
"AAA"	Calibration Plan Section	"462" = Cal. Plan 4.6.2 chapter 4, section 6, subsection 2	462
"BBBB"	Location of test or ASU Chamber Temperature	"AMB" = MSSS Ambient, "TN10" = MSSS TVAC -10C, …	TAMB
"C"	Camera unit under test	"L" = Left Mastcam-Z, "R" = Right Mastcam-Z, "E" =EQ "C" =COTS	L/R
"DD"	Part of test	"00" = test set up, "01" = first part,…	00-16

1. [D] ____ Look up the daily calibration schedule and record the scheduled start and end time of this test on the cover page of this document. Also, fill out and double-check the other information on the cover page.
2. [D] ____ Ensure that all supplemental manuals are on hand. These are,
   - Validator_Manual, Documentarian_Manual,
   - MastcamZCalPlan
3. [D] ____ Ensure that the Image Log is present and ready to use. Find and open the Google Sheets file "Image_Log_46". There is a link on the Wiki.
4. [V] ____ Check that all *Calgorithms* fast-look and validation scripts are present, up-to-date, and ready to analyze test output. Find and open the "Geometric_Calibration_46_Validation" Jupyter notebook. There is a link on the Wiki.

5. [O] CO Check that all camera scripts required for this test are present, up-to-date and ready to command the ground support equipment (GSE). These are,

- **462TAMBR00 - 462TAMBR16**
- **462TAMBL00 - 462TAMBL16**

6. [O, V, D, L] Notes: We added autofocus scripts for 48mm, 79mm, 110 mm focal lengths.

Date 5/2 Time 9:03 Initials [illegible]

Hardware Installation

This procedure is for the ambient TVAC chamber testing at MSSS. Figure 1 shows the nominal layout of the cleanroom, workspace, Mastcam-Zs, ground support equipment (GSE), targets, sources, and other equipment necessary.

Figure 1. MSSS Floor Plan for Geometric Testing in the cleanroom.

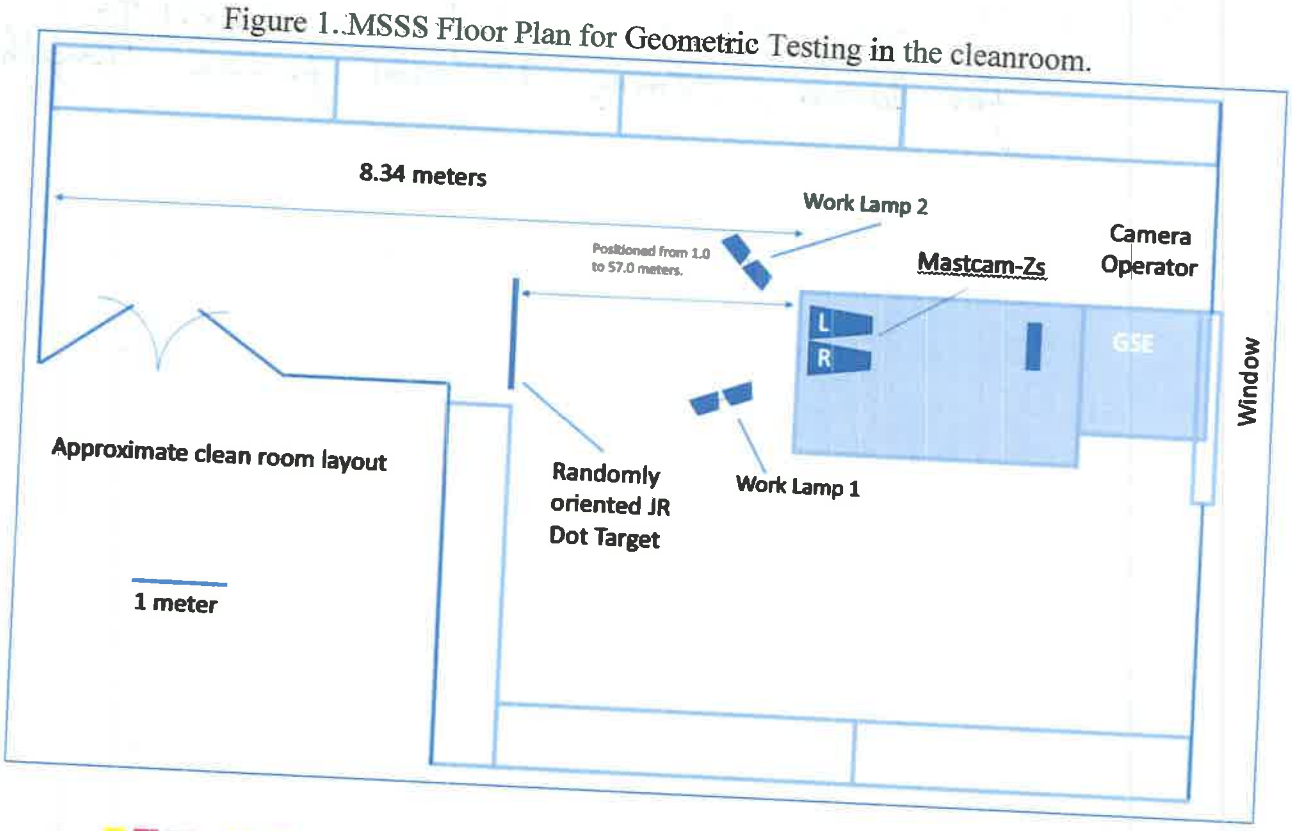

7. [T, O, L] ___ Ensure that all personnel in the cleanroom are following the cleanroom practices for electrostatic discharge, proper clothing, and other safety concerns.
8. [O, D] ___ Check the camera temperature and ensure nominal operation.
9. [T] N/A Double check that nitrogen is flowing over the Mastcam-Zs or the window port.
10. [T] ___ Install the lamps and position them to shine on the geometric target out of the camera's FOV. Power them on.
11. [O, T] ___ Ensure that the camera unit and GSE wires are secure, kink-free, and do not present tripping hazards when the lights are turned off.
12. [M] ___ Install the metrology equipment.
13. [D] ___ Record the following temperatures:

Date 5/2 Time 9:10 Initials [illegible]

- Cleanroom temperature 70°F pressure N/A humidity 65%

14. [O,D, L] Notes:

Table 2. The nominal target positions for autofocusing

	Target Positions	Notes
Position 1	The 40x40 dot target at 1.0 meter centered on the camera boresight	
Position 2	The 40x40 dot target at 2.0 meters	
Position 3	The 40x40 dot target at 3.0 meters	
Position 4	The 40x40 dot target at 4.0 meters	
Position 5	The 40x40 dot target at 5.0 meters	
Position 6	The 40x40 dot target at 7.0 meters	

Date 5/2 Time 9:20 Initials [illegible]

Left and Right Mastcam-Z Tests

26mm Focal Length 1 for the Mastcam-Zs

15. [M,T] ___ Position the 40x40 dot geometric target to Position 1 as described in Table 2.
16. [M] ___ Measure the locations of the camera on the optics bench. Record the location measurements in the Image Log and tables below.

17. [D] ___ Record the following temperatures:
    - Left Camera CCD temp 24.1
    - Right Camera CCD temp 24.6
18. [D,T] ___ Take pictures the geometric target position and the whole test/GSE set-up.

Best Focus Model

f [mm]	FL [mc2]	D [meters]	FD' FM2-L [mc0]	FD' FM1-R [mc0]
26	0	1.0	-3540	-3762
26	0	1.4	-3300 -2940	-3588 -3138
26	0	1.7	-2676	-2868
26	0	2.0	-2448 -2490	-2694 -2676
26	0	2.3	-2358	-2532
26	0	2.6	-2250	-2424
26	0	3.0	-2052 -2142	-2346 -2316
26	0	3.4	-2064	-2226
26	0	4.0	-1968	-2130
26	0	5.0	-1866	-2022
26	0	6.0	-1710 -1644 -1794	-1974 -1932 -1950
26	0	8.0	-1710	-1860
26	0	10.0	-1656	-1806
26	0	16.0	-1578	-1728
26	0	30.0	-1518	-1662
26	0	inf	-1452	-1596

The model checks out!

FM1

19. [O] CO Load and execute camera script **462TAMBL01** and **462TAMBR01**, which autofocuses and captures 1 frame with filter 0 at 26mm focal length. Insert the note "TARGET=DOT40,METROLOGY=[ID#]" in the GUI.
20. [M,O] CO Reposition the target to the 4 or 5 additional positions and capture identical frames saved as prefix **462TAMBL01** and **462TAMBR01**. For each run insert the note "TARGET=DOT40,METROLOGY=[ID#]" in the GUI.
21. [D] CO Record image names and parameters in Image Log.
22. [M,D, L] Notes: Autofocused at ~1.0 m
The first images (0-6) had saturated pixels.

Focal Length 1 and Focus 1

Time	Zoom [mc2]	Focus FM1 [mc0]	Focus FM2 [mc0]	Position #	Suffix and Notes
9:10	0	-3300	-3588	001	462TAMBR/L0000-03
9:19				002	462TAMBR/L01_00-01
9:21				003	03-06
9:28				004	07
9:40				005	08
10:09				006	09
10:13				007	10

23. [O] CO Load and execute camera script **462TAMBL01** and **462TAMBR01**, which autofocuses and captures 1 frame with filter 0 at 26mm focal length. Insert the note "TARGET=DOT40,METROLOGY=[ID#]" in the GUI.
24. [M,O] CO Reposition the target to the 4 or 5 additional positions and capture identical frames saved as prefix **462TAMBL01** and **462TAMBR01**. For each run insert the note "TARGET=DOT40,METROLOGY=[ID#]" in the GUI.
25. [D] CO Record image names and parameters in Image Log.
26. [M,D, L] Notes: AF @ ~70m

Focal Length 1 and Focus 2

Time	Zoom [mc2]	Focus FM1 [mc0]	Focus FM2 [mc0]	Position #	Suffix and Notes
10:30	0	-1644	-1932	008	11, 12
10:33				009	13
10:34				010	14
10:36				011	15
10:37				012	16
10:39				013	17
10:40				014	18

Date 5/2 Time 10:45 Initials CO

27. [O] CO Load and execute camera script **462TAMBL01** and **462TAMBR01**, which autofocuses and captures 1 frame with filter 0 at 26mm focal length. Insert the note "TARGET=DOT40,METROLOGY=[ID#]" in the GUI.
28. [M,O] CO Reposition the target to the 4 or 5 additional positions and capture identical frames saved as prefix **462TAMBL01** and **462TAMBR01**. For each run insert the note "TARGET=DOT40,METROLOGY=[ID#]" in the GUI.
29. [D] CO Record image names and parameters in Image Log.
30. [M,D, L] Notes: AF @ ~ 2.0m

Focal Length 1 and Focus 3

Time	Zoom [mc2]	Focus FM1 [mc0]	Focus FM2 [mc0]	Position #	Suffix and Notes
10:45	0	-2448	-2694	015	19, 20
	|	|	|	016	21
	|	|	|	017	22
	|	|	|	018	23
	|	|	|	019	24
10:58	|	|	|	020	25
11:00	|	|	|	021	26

Date 5/2 Time 11:02 Initials [illegible]

31. [O] [illegible] Load and execute camera script **462TAMBL01** and **462TAMBR01**, which autofocuses and captures 1 frame with filter 0 at 26mm focal length. Insert the note "TARGET=DOT40,METROLOGY=[ID#]" in the GUI.
32. [M,O] [illegible] Reposition the target to the 4 or 5 additional positions and capture identical frames saved as prefix **462TAMBL01** and **462TAMBR01**. For each run insert the note "TARGET=DOT40,METROLOGY=[ID#]" in the GUI.
33. [D] [illegible] Record image names and parameters in Image Log.
34. [M,D, L] Notes: AF @ ~6.0 m

Suffix 36 is a throw away, target was in movement

Focal Length 1 and Focus 4

Time	Zoom [mc2]	Focus FM1 [mc0]	Focus FM2 [mc0]	Position #	Suffix and Notes
11:02	0	-1710	-1979	022	27
	|	|	|	023	
	|	|	|	024	
	|	|	|	025	
11:20	|	|	|	026	34
	|	|	|	027	35
11:28	|	|	|	028	37

Date 5/2 Time 11:30 Initials CO

35. [O] CO Load and execute camera script **462TAMBL01** and **462TAMBR01**, which autofocuses and captures 1 frame with filter 0 at 26mm focal length. Insert the note "TARGET=DOT40,METROLOGY=[ID#]" in the GUI.
36. [M,O] CO Reposition the target to the 4 or 5 additional positions and capture identical frames saved as prefix **462TAMBL01** and **462TAMBR01**. For each run insert the note "TARGET=DOT40,METROLOGY=[ID#]" in the GUI.
37. [D] CO Record image names and parameters in Image Log.
38. [M,D, L] Notes: AF @ ~3.0 m

Focal Length 1 and Focus 5

Time	Zoom [mc2]	Focus FM1 [mc0]	Focus FM2 [mc0]	Position #	Suffix and Notes
11:30	0	-2052	-2346	029	38, 39, 40
11:40	|	|	|	030	41
	|	|	|	031	42
11:42	|	|	|	032	43
	|	|	|	033	44
	|	|	|	034	45
11:50	|			035	46

Data Validation

39. [V] CO Run the geometric validation Jupyter notebook on the acquired data from the Right and Left Mastcam-Zs.
40. [V,D, L] Notes: Data Look good

34mm Focal Length 2 for the Mastcam-Zs

41. [M,T] __JR__ Position the 40x40 dot geometric target to Position 1 as described in Table 2.
42. [M] __JR__ Measure the locations of the camera on the optics bench. Record the location measurements in the Image Log and tables below.

43. [D] __JR__ Record the following temperatures:
    - Left Camera CCD temp __24.1° C__
    - Right Camera CCD temp __24.7°C__
44. [D,T] __JR__ Take pictures the geometric target position and the whole test/GSE set-up.

Best Focus Model

f [mm]	FL [mc2]	D [meters]	FD' FM2-L [mc0]	FD' FM1-R [mc0]
34	2448	1.0	-1140 ~~-1212~~ -1326	-115~~2~~ -1278
34	2448	1.4	-732	-684
34	2448	1.7	-474	-420
34	2448	2.0	-288	-240
34	2448	2.3	-156	-102
34	2448	2.6	-54	0
34	2448	3.0	36	114
34	2448	3.4	138	192
34	2448	4.0	228	282
34	2448	5.0	330	384
34	2448	6.0	402	456
34	2448	8.0	486	540
34	2448	10.0	540	594
34	2448	16.0	618	672
34	2448	30.0	678	732
34	2448	inf	768	828

45. [O] ~~CJ~~ Load and execute camera script **462TAMBL01** and **462TAMBR01**, which autofocuses and captures 1 frame with filter 0 at 34mm focal length. Insert the note "TARGET=DOT40,METROLOGY=[ID#]" in the GUI.
46. [M,O] ~~CJ~~ Reposition the target to the 4 or 5 additional positions and capture identical frames saved as prefix **462TAMBL01** and **462TAMBR01**. For each run insert the note "TARGET=DOT40,METROLOGY=[ID#]" in the GUI.
47. [D] ~~CJ~~ Record image names and parameters in Image Log.
48. [M,D, L] Notes: AF @ ~1.0 m

Focal Length 2 and Focus 1

Time	Zoom [mc2]	Focus FM1 [mc0]	Focus FM2 [mc0]	Position #	Suffix and Notes
12:40	2448	~~-11582~~ -1152	~~-1212~~ -1140	036	47 (AF test)
				037	52
				038	53
				039	54
				040	55
				041	56
				042	

Date 5/2 Time 1:20 Initials JK

49. [O] JK Load and execute camera script **462TAMBL0~~2~~1** and **462TAMBR0~~2~~1**, which autofocuses and captures 1 frame with filter 0 at 34mm focal length. Insert the note "TARGET=DOT40,METROLOGY=[ID#]" in the GUI.
50. [M,O] JK Reposition the target to the 4 or 5 additional positions and capture identical frames saved as prefix **462TAMBL0~~2~~1** and **462TAMBR0~~2~~1**. For each run insert the note "TARGET=DOT40,METROLOGY=[ID#]" in the GUI.
51. [D] JK Record image names and parameters in Image Log.
52. [M,D, L] Notes: AF @ 6 meters

Focal Length 2 and Focus 2

Time	Zoom [mc2]	Focus FM1 [mc0]	Focus FM2 [mc0]	Position #	Suffix and Notes
	2448	-480	-498	042	58
				43	59
				044	60
				045	61
				046	62
				047	63
				048	64
				049	65

Date 5/2 Time [illegible] Initials [illegible]

53. [O] X Load and execute camera script **462TAMBL01** and **462TAMBR01**, which autofocuses and captures 1 frame with filter 0 at 34mm focal length. Insert the note "TARGET=DOT40,METROLOGY=[ID#]" in the GUI.
54. [M,O] X Reposition the target to the 4 or 5 additional positions and capture identical frames saved as prefix **462TAMBL01** and **462TAMBR01**. For each run insert the note "TARGET=DOT40,METROLOGY=[ID#]" in the GUI.
55. [D] X Record image names and parameters in Image Log.
56. [M,D, L] Notes: AF @ 2 meters

Focal Length 2 and Focus 3

Time	Zoom [mc2]	Focus FM1 [mc0]	Focus FM2 [mc0]	Position #	Suffix and Notes
	2448	-264	-282	050	66
	|	|	|	051	67
1:26	|	|	|	052	68
	|	|	|	053	69
1:30	|	|	|	054	70
	|	|	|	055	71
	|	|	|		
		|	|		

Date 5/12 Time 1:41 Initials JK

57. [O] JK Load and execute camera script **462TAMBL01** and **462TAMBR01**, which autofocuses and captures 1 frame with filter 0 at 34mm focal length. Insert the note "TARGET=DOT40,METROLOGY=[ID#]" in the GUI.
58. [M,O] JK Reposition the target to the 4 or 5 additional positions and capture identical frames saved as prefix **462TAMBL01** and **462TAMBR01**. For each run insert the note "TARGET=DOT40,METROLOGY=[ID#]" in the GUI.
59. [D] JK Record image names and parameters in Image Log.
60. [M,D, L] Notes: AF @ 6 meter
Re-take do to moving position

Focal Length 2 and Focus 4

Time	Zoom [mc2]	Focus FM1 [mc0]	Focus FM2 [mc0]	Position #	Suffix and Notes
	2448	4426	4426	057	73, 74, 75
		|	|	058	76
		|	|	059	77
		|	|	060	78
		|	|	061	79
		|	|	062	80
		|	|	063	81

61. [O] JK Load and execute camera script **462TAMBL0~~2~~1** and **462TAMBR0~~2~~1**, which autofocuses and captures 1 frame with filter 0 at 34mm focal length. Insert the note "TARGET=DOT40,METROLOGY=[ID#]" in the GUI.

62. [M,O] JK Reposition the target to the 4 or 5 additional positions and capture identical frames saved as prefix **462TAMBL0~~2~~1** and **462TAMBR0~~2~~1**. For each run insert the note "TARGET=DOT40,METROLOGY=[ID#]" in the GUI.

63. [D] JK Record image names and parameters in Image Log.

64. [M,D, L] Notes: AF @ 3 meters

Focal Length 2 and Focus 5

Time	Zoom [mc2]	Focus FM1 [mc0]	Focus FM2 [mc0]	Position #	Suffix and Notes
1:53	2448	+114	+48		
				064	84
				65	85
				66	86
				67	87
				68	88
				69	89
				070	90

Data Validation

65. [V] ____ Run the geometric validation Jupyter notebook on the acquired data from the Right and Left Mastcam-Zs.

66. [V,D, L] Notes: ______________________________

Date 5/2 Time 2:05 Initials R

48mm Focal Length 3 for the Mastcam-Zs

67. [M,T] R Position the 40x40 dot geometric target to Position 1 as described in Table 2.
68. [M] R Measure the locations of the camera on the optics bench. Record the location measurements in the Image Log and tables below.

69. [D] R Record the following temperatures:
    - Left Camera CCD temp 24.3
    - Right Camera CCD temp 24.8
70. [D,T] R Take pictures the geometric target position and the whole test/GSE set-up.

Best Focus Model

f [mm]	FL [mc2]	D [meters]	FD' FM1-L [mc0]	FD' FM1-R [mc0]
48	3834	1.0	-108	36
48	3834	1.4	480	630
48	3834	1.7	738	882
48	3834	2.0	918	1062
48	3834	2.3	1056	1194
48	3834	2.6	1158	1296
48	3834	3.0	1266	1398
48	3834	3.4	1344	1476
48	3834	4.0	1434	1566
48	3834	5.0	1536	1668
48	3834	6.0	1608	1734
48	3834	8.0	1692	1824
48	3834	10.0	1740	1872
48	3834	16.0	1818	1950
48	3834	30.0	1878	2004
48	3834	inf	1908	2034

71. [O] JK Load and execute camera script **462TAMBL0~~3~~1** and **462TAMBR0~~3~~1**, which autofocuses and captures 1 frame with filter 0 at 48mm focal length. Insert the note "TARGET=DOT40,METROLOGY=[ID#]" in the GUI.

72. [M,O] JK Reposition the target to the 4 or 5 additional positions and capture identical frames saved as prefix **462TAMBL0~~3~~1** and **462TAMBR0~~3~~1**. For each run insert the note "TARGET=DOT40,METROLOGY=[ID#]" in the GUI.

73. [D] JK Record image names and parameters in Image Log.

74. [M,D, L] Notes: AF @ 7 meters.
Note: ~~Flags~~ The Prefix in the software did not change to match procedure yet Log is changed.

Focal Length 3 and Focus 1

Time	Zoom [mc2]	Focus FM1 [mc0]	Focus FM2 [mc0]	Position #	Suffix and Notes
	3834	1710	1740		
				071	93
				072	94
				073	95
				074	96
				075	97
				076	98
				077	99

Date 5/2 Time 2:30 Initials SK

75. **[O]** SK Load and execute camera script **462TAMBL03** (1) and **462TAMBR03** (1), which autofocuses and captures 1 frame with filter 0 at 48mm focal length. Insert the note "TARGET=DOT40,METROLOGY=[ID#]" in the GUI.
76. **[M,O]** SK Reposition the target to the 4 or 5 additional positions and capture identical frames saved as prefix **462TAMBL03** (1) and **462TAMBR03** (1). For each run insert the note "TARGET=DOT40,METROLOGY=[ID#]" in the GUI.
77. **[D]** SK Record image names and parameters in Image Log.
78. **[M,D, L]** Notes: AF @ 1 meter

Focal Length 3 and Focus 2

* Autofocus failed *

Time	Zoom [mc2]	Focus FM1 [mc0]	Focus FM2 [mc0]	Position #	Suffix and Notes
2:23	3834	126	156	078	100* 101
				079	102
				080	103
				081	104
				082	105
				083	106
				084	107

Date 5/2 Time 2:40 Initials JS

79. [O] JS Load and execute camera script **462TAMBL01** and **462TAMBR01**, which autofocuses and captures 1 frame with filter 0 at 48mm focal length. Insert the note "TARGET=DOT40,METROLOGY=[ID#]" in the GUI.
80. [M,O] JS Reposition the target to the 4 or 5 additional positions and capture identical frames saved as prefix **462TAMBL01** and **462TAMBR01**. For each run insert the note "TARGET=DOT40,METROLOGY=[ID#]" in the GUI.
81. [D] JK Record image names and parameters in Image Log.
82. [M,D, L] Notes: AF @ 6 meters

* Jarlid Full Frame

Focal Length 3 and Focus 3

Time	Zoom [mc2]	Focus FM1 [mc0]	Focus FM2 [mc0]	Position #	Suffix and Notes
2:41	3834	1644	1680	~~[illegible]~~	
				85	108* 109 110 (110= White frame test)
				86	111
2:50				87	112
				88	113
				89	114
				90	115
				91	116

83. [O] JK Load and execute camera script **462TAMBL01** and **462TAMBR01**, which autofocuses and captures 1 frame with filter 0 at 48mm focal length. Insert the note "TARGET=DOT40,METROLOGY=[ID#]" in the GUI.
84. [M,O] JK Reposition the target to the 4 or 5 additional positions and capture identical frames saved as prefix **462TAMBL01** and **462TAMBR01**. For each run insert the note "TARGET=DOT40,METROLOGY=[ID#]" in the GUI.
85. [D] JK Record image names and parameters in Image Log.
86. [M,D, L] Notes: AE @ 2 meters

Focal Length 3 and Focus 4

Time	Zoom [mc2]	Focus FM1 [mc0]	Focus FM2 [mc0]	Position #	Suffix and Notes
	3834	966	1008	92	117
				93	118
				94	119
3:05				95	120
				96	121
				97	122
				98	123

Date 5/2 Time 3:09 Initials JS

87. [O] JS Load and execute camera script **462TAMBL03** and **462TAMBR03**, which autofocuses and captures 1 frame with filter 0 at 48mm focal length. Insert the note "TARGET=DOT40,METROLOGY=[ID#]" in the GUI.

88. [M,O] JS Reposition the target to the 4 or 5 additional positions and capture identical frames saved as prefix **462TAMBL03** and **462TAMBR03**. For each run insert the note "TARGET=DOT40,METROLOGY=[ID#]" in the GUI.

89. [D] JS Record image names and parameters in Image Log.

90. [M,D, L] Notes: AF @ 5 meters

Most acquisitions take 4 seconds.

Focal Length 3 and Focus 5

Time	Zoom [mc2]	Focus FM1 [mc0]	Focus FM2 [mc0]	Position #	Suffix and Notes
3:09	3834	1554	1602	099	~~Auto~~ 124, 125, 126
				100	127
				101	128
				102	129
				103	130
				104	131
				105	132
				106	133 134
				107	135
				108	136
				109	137
				110	138
				111	139
				112	

134 – Autofocus in full frame

At 3 meters

Data Validation

91. [V] ____ Run the geometric validation Jupyter notebook on the acquired data from the Right and Left Mastcam-Zs.

92. [V,D, L] Notes: ______

Date 5/2 Time 3:40 Initials JS

63mm Focal Length 4 for the Mastcam-Zs

93. [M,T] JS Position the 40x40 dot geometric target to Position 1 as described in Table 2.
94. [M] JS Measure the locations of the camera on the optics bench. Record the location measurements in the Image Log and tables below.

95. [D] JS Record the following temperatures:
   - Left Camera CCD temp 24.5
   - Right Camera CCD temp 25.0
96. [D,T] JS Take pictures the geometric target position and the whole test/GSE set-up.

Best Focus Model

f [mm]	FL [mc2]	D [meters]	FD' FM2-L [mc0]	FD' FM1-R [mc0]
63	5196	1.0	696	894
63	5196	1.4	1278	1464
63	5196	1.7	1536	1716
63	5196	2.0	1716	1890
63	5196	2.3	1848	2022
63	5196	2.6	1950	2124
63	5196	3.0	2058	2226
63	5196	3.4	2136	2304
63	5196	4.0	2226	2388
63	5196	5.0	2328	2490
63	5196	6.0	2394	2556
63	5196	8.0	2484	2640
63	5196	10.0	2532	2688
63	5196	16.0	2610	2766
63	5196	30.0	2670	2820
63	5196	inf	2784	2922

97. [O] JK Load and execute camera script **462TAMBL0~~4~~1** and **462TAMBR0~~4~~1**, which autofocuses and captures 1 frame with filter 0 at 63mm focal length. Insert the note "TARGET=DOT40,METROLOGY=[ID#]" in the GUI.

98. [M,O] JK Reposition the target to the 4 or 5 additional positions and capture identical frames saved as prefix **462TAMBL0~~4~~1** and **462TAMBR0~~4~~1**. For each run insert the note "TARGET=DOT40,METROLOGY=[ID#]" in the GUI.

99. [D] JK Record image names and parameters in Image Log.

100. [M,D, L] Notes: AF @ 7 meters

Focal Length 4 and Focus 1

* Junk
** sub-frame

Time	Zoom [mc2]	Focus FM1 [mc0]	Focus FM2 [mc0]	Position #	Suffix and Notes
	5196	2598	2640		
3:43				113	141* 142** 143■
3:53				114	144
				115	145
				116	146
				117	147
				118	148
				119	149

■ exposure increased to 200ms

Date 5/2 Time ______ Initials JK

101. [O] JK Load and execute camera script **462TAMBL0~~4~~1** and **462TAMBR0~~4~~1**, which autofocuses and captures 1 frame with filter 0 at 63mm focal length. Insert the note "TARGET=DOT40,METROLOGY=[ID#]" in the GUI.
102. [M,O] JK Reposition the target to the 4 or 5 additional positions and capture identical frames saved as prefix **462TAMBL0~~4~~1** and **462TAMBR0~~4~~1**. For each run insert the note "TARGET=DOT40,METROLOGY=[ID#]" in the GUI.
103. [D] JK Record image names and parameters in Image Log.
104. [M,D, L] Notes: AF @ 1meter

* Fail ** success

Focal Length 4 and Focus 2

Time	Zoom [mc2]	Focus FM1 [mc0]	Focus FM2 [mc0]	Position #	Suffix and Notes
	5196	1038	1068	120	150* 151**
	↓	↓	↓	121	152
				122	153
				123	154
				124	155
				125	156
				126	157

105. [O] JK Load and execute camera script **462TAMBL04** and **462TAMBR04**, which autofocuses and captures 1 frame with filter 0 at 63mm focal length. Insert the note "TARGET=DOT40,METROLOGY=[ID#]" in the GUI.

106. [M,O] JK Reposition the target to the 4 or 5 additional positions and capture identical frames saved as prefix **462TAMBL04** and **462TAMBR04**. For each run insert the note "TARGET=DOT40,METROLOGY=[ID#]" in the GUI.

107. [D] JK Record image names and parameters in Image Log.

108. [M,D, L] Notes: AF @ 6 meters

* No autofocus / full frame.

Focal Length 4 and Focus 3

Time	Zoom [mc2]	Focus FM1 [mc0]	Focus FM2 [mc0]	Position #	Suffix and Notes
	5196	2544	2556		
4:22				127	158* 159, 160 (full frame)
				128	161
				129	162
				130	163
				131	164
4:35				132	165
				133	166

Date 5/2 Time 4:38 Initials JK

109. [O] JK Load and execute camera script **462TAMBL04** and **462TAMBR04**, which autofocuses and captures 1 frame with filter 0 at 63mm focal length. Insert the note "TARGET=DOT40,METROLOGY=[ID#]" in the GUI.
110. [M,O] JK Reposition the target to the 4 or 5 additional positions and capture identical frames saved as prefix **462TAMBL04** and **462TAMBR04**. For each run insert the note "TARGET=DOT40,METROLOGY=[ID#]" in the GUI.
111. [D] JK Record image names and parameters in Image Log.
112. [M,D, L] Notes: AF @ 2 meters

Focal Length 4 and Focus 4

Time	Zoom [mc2]	Focus FM1 [mc0]	Focus FM2 [mc0]	Position #	Suffix and Notes
4:38	5196	1854	1908	134	167
				135	168
				136	169
				137	170
				138	171
				139	172
				140	173

Date 4:45 Time 5/2 Initials [illegible]

113. [O] [illegible] Load and execute camera script **462TAMBL01** and **462TAMBR01**, which autofocuses and captures 1 frame with filter 0 at 63mm focal length. Insert the note "TARGET=DOT40,METROLOGY=[ID#]" in the GUI.

114. [M,O] [illegible] Reposition the target to the 4 or 5 additional positions and capture identical frames saved as prefix **462TAMBL01** and **462TAMBR01**. For each run insert the note "TARGET=DOT40,METROLOGY=[ID#]" in the GUI.

115. [D] [illegible] Record image names and parameters in Image Log.

116. [M,D, L] Notes: AF @ 5 meters

Focal Length 4 and Focus 5

Time	Zoom [mc2]	Focus FM1 [mc0]	Focus FM2 [mc0]	Position #	Suffix and Notes
	5196	2460	2502		
4:46				141	174,
				142	175
				143	176
				144	177
				145	178
				146	179
				147	180

FM1 = 2184 New Autofocus at 3 meters →
FM2 = 2226

148	181	→ full frame [illegible]
149	182	
150	183	
151	184	
152	185	
153	186	
154	187	

Data Validation

117. [V] ____ Run the geometric validation Jupyter notebook on the acquired data from the Right and Left Mastcam-Zs.

118. [V,D, L] Notes: ____

Paused procedure at the end of the shift. [illegible]
Proceed at 8am tomorrow.
5:33 5/2/19

Date 5/3 Time 8:00 Initials JK

Proceeding with procedure Friday morning. [illegible]

79mm Focal Length 5 for the Mastcam-Zs

119. [M,T] JK Position the 40x40 dot geometric target to Position 1 as described in Table 2.

120. [M] JK Measure the locations of the camera on the optics bench. Record the location measurements in the Image Log and tables below.

121. [D] JK Record the following temperatures:
- Left Camera CCD temp 21.8 °C
- Right Camera CCD temp 22.6 °C

122. [D,T] JK Take pictures the geometric target position and the whole test/GSE set-up.

Best Focus Model

f [mm]	FL [mc2]	D [meters]	FD' FM2-L [mc0]	FD' FM2-R [mc0]
79	6720	1.0	1170	1368
79	6720	1.4	1746	1932
79	6720	1.7	1998	2178
79	6720	2.0	2178	2352
79	6720	2.3	2310	2484
79	6720	2.6	2412	2580
79	6720	3.0	2514	2682
79	6720	3.4	2592	2760
79	6720	4.0	2682	2850
79	6720	5.0	2784	2946
79	6720	6.0	2850	3012
79	6720	8.0	2934	3096
79	6720	10.0	2988	3144
79	6720	16.0	3060	3216
79	6720	30.0	3120	3276
79	6720	inf	3174	3330

Date 5/3 Time 8:26 Initials JK

123. [O] JK Load and execute camera script **462TAMBL0~~5~~1** and **462TAMBR0~~5~~1**, which autofocuses and captures 1 frame with filter 0 at 79mm focal length. Insert the note "TARGET=DOT40,METROLOGY=[ID#]" in the GUI.

124. [M,O] JK Reposition the target to the 4 or 5 additional positions and capture identical frames saved as prefix **462TAMBL0~~5~~1** and **462TAMBR0~~5~~1**. For each run insert the note "TARGET=DOT40,METROLOGY=[ID#]" in the GUI.

125. [D] JK Record image names and parameters in Image Log.

126. [M,D, L] Notes: AF @ 1 meter
Note = 200 ms exposure

* failure

Focal Length 5 and Focus 1

Time	Zoom [mc2]	Focus FM1 [mc0]	Focus FM2 [mc0]	Position #	Suffix and Notes
	6720	1434	1488		
				155	188*, 189
				156	190
				157	191
				158	192
				159	193
				160	194
				161 /~2m	195

162 /~1.5m | 196
163 | 197
164 /~1.25 | 198

Date 5/3 Time 8:54 Initials JK

127. [O] JK Load and execute camera script **462TAMBL05** and **462TAMBR05**, which autofocuses and captures 1 frame with filter 0 at 79mm focal length. Insert the note "TARGET=DOT40,METROLOGY=[ID#]" in the GUI.

128. [M,O] JK Reposition the target to the 4 or 5 additional positions and capture identical frames saved as prefix **462TAMBL05** and **462TAMBR05**. For each run insert the note "TARGET=DOT40,METROLOGY=[ID#]" in the GUI.

129. [D] JK Record image names and parameters in Image Log.

130. [M,D, L] Notes: AF @ 2 meters.

Focal Length 5 and Focus 2

Time	Zoom [mc2]	Focus FM1 [mc0]	Focus FM2 [mc0]	Position #	Suffix and Notes
	6720	2262	2328		
8:55				165	199 / 2 meters
				166	200 / ~1 meter
				167	201 / 1.25 m
				168	202 / 1.75 m
				169	203 / 2.2
				170	204
				171	205
				172	206 / 3 mete

Date 5/3 Time 9:06 Initials ______

131. [O] JK Load and execute camera script **462TAMBL05** and **462TAMBR05**, which autofocuses and captures 1 frame with filter 0 at 79mm focal length. Insert the note "TARGET=DOT40,METROLOGY=[ID#]" in the GUI.
132. [M,O] JK Reposition the target to the 4 or 5 additional positions and capture identical frames saved as prefix **462TAMBL05** and **462TAMBR05**. For each run insert the note "TARGET=DOT40,METROLOGY=[ID#]" in the GUI.
133. [D] JK Record image names and parameters in Image Log.
134. [M,D, L] Notes: AF @ 3 meters
Note 200 ms

Focal Length 5 and Focus 3

Time	Zoom [mc2]	Focus FM1 [mc0]	Focus FM2 [mc0]	Position #	Suffix and Notes
	6720	2580	2640	—	—
9:07	|	|	|	173	207
	|	|	|	174	208 / 2.0 m
	|	|	|	175	209 / 2.25 m
	|	|	|	176	210 / 2.5 m
	|	|	|	177	211 / 2.75 m
	|	|	|	178 / 3.25	212
	|	|	|	179 / 3.5	213
				180 / 3.75	214
				181 / 4.0m	215

Date 5/3 Time 9:16 Initials JK

135. [O] JK Load and execute camera script **462TAMBL05** (5 struck through, 1 written above) and **462TAMBR05** (5 struck through, 1 written above), which autofocuses and captures 1 frame with filter 0 at 79mm focal length. Insert the note "TARGET=DOT40,METROLOGY=[ID#]" in the GUI.

136. [M,O] JK Reposition the target to the 4 or 5 additional positions and capture identical frames saved as prefix **462TAMBL05** (5 struck through, 1 written above) and **462TAMBR05** (5 struck through, 1 written above). For each run insert the note "TARGET=DOT40,METROLOGY=[ID#]" in the GUI.

137. [D] JK Record image names and parameters in Image Log.

138. [M,D, L] Notes: AF @ 4.0 m

Focal Length 5 and Focus 4

Time	Zoom [mc2]	Focus FM1 [mc0]	Focus FM2 [mc0]	Position #	Suffix and Notes
	6720	2580	2808		
9:16	|	|	|	182	216
	|	|	|	183 /3m	217
	|	|	|	184/3.25	218
	|	|	|	185/3.5	219
	|	|	|	186/3.75	220
9:25	|	|	|	187/4.25	221
				188/4.5	222

189/4.75 | 223

190/5m | 224

 Date 5/5 Time 9:28 Initials JK

139. [O] JS Load and execute camera script **462TAMBL0~~5~~1** and **462TAMBR0~~5~~1**, which autofocuses and captures 1 frame with filter 0 at 79mm focal length. Insert the note "TARGET=DOT40,METROLOGY=[ID#]" in the GUI.

140. [M,O] JK Reposition the target to the 4 or 5 additional positions and capture identical frames saved as prefix **462TAMBL0~~5~~1** and **462TAMBR0~~5~~1**. For each run insert the note "TARGET=DOT40,METROLOGY=[ID#]" in the GUI.

141. [D] JR Record image names and parameters in Image Log.

142. [M,D, L] Notes: AF @ 5 meters
Each acquisition take 3-4 seconds

79 mm Focal length finished.

Focal Length 5 and Focus 5

Time	Zoom [mc2]	Focus FM1 [mc0]	Focus FM2 [mc0]	Position #	Suffix and Notes
	6720	2862	2916		
9:32	|	|	|	191	225
	|	|	|	192/4m	226
	|	|	|	193/4.25m	227
	|	|	|	194/4.5	228
	|	|	|	195/4.75	229
	|	|	|	196/5.25	230
		|		197/5.5	231
				198/5.75	232
				199/6.0	233

Data Validation

143. [V] ____ Run the geometric validation Jupyter notebook on the acquired data from the Right and Left Mastcam-Zs.

144. [V,D, L] Notes: AF @ 6 meters
FM1 = 2928
FM2 = 2988

Position	Suffix
200/6m	235
201	236
202/5m	237
203/5.25	
204/5.5	
206/6.25	241
207/6.5	242
208/6.75	243
209/7.0m	244

Date 5/3 Time 10:12 Initials JS

100mm Focal Length 6 for the Mastcam-Zs

145. [M,T] JS Position the 40x40 dot geometric target to Position 1 as described in Table
146. [M] JS Measure the locations of the camera on the optics bench. Record the location measurements in the Image Log and tables below.

147. [D] JS Record the following temperatures:
    - Left Camera CCD temp 23.3
    - Right Camera CCD temp 23.7
148. [D,T] JS Take pictures the geometric target position and the whole test/GSE set-up.

Best Focus Model

f [mm]	FL [mc2]	D [meters]	FD' FM2-L [mc0]	FD' FM1-R [mc0]
100	8652	1.0	1170	1338
100	8652	1.4	1740	1908
100	8652	1.7	1992	2160
100	8652	2.0	2166	2334
100	8652	2.3	2298	2460
100	8652	2.6	2394	2562
100	8652	3.0	2502	2664
100	8652	3.4	2580	2742
100	8652	4.0	2664	2826
100	8652	5.0	2766	2928
100	8652	6.0	2832	2994
100	8652	8.0	2916	3078
100	8652	10.0	2964	3126
100	8652	16.0	3042	3198
100	8652	30.0	3096	3258
100	8652	inf	3168	3336

149. [O] JK Load and execute camera script **462TAMBL0~~6~~1** and **462TAMBR0~~6~~1**, which autofocuses and captures 1 frame with filter 0 at 100mm focal length. Insert the note "TARGET=DOT40,METROLOGY=[ID#]" in the GUI.

150. [M,O] JK Reposition the target to the 4 or 5 additional positions and capture identical frames saved as prefix **462TAMBL0~~6~~1** and **462TAMBR0~~6~~1**. For each run insert the note "TARGET=DOT40,METROLOGY=[ID#]" in the GUI.

151. [D] JK Record image names and parameters in Image Log.

152. [M,D, L] Notes: AF @ 7.5 m
Note at 100m focal length

Focal Length 6 and Focus 1

Time	Zoom [mc2]	Focus FM1 [mc0]	Focus FM2 [mc0]	Position #	Suffix and Notes
JK	8652	3054	2988		
				217/7.5m	253
				218/7.25m	254
				219/7.0m	255
				220/6.5	256
				221/6.25	257
				222/6.0	258
				~~223~~	

Date 5/3 Time 10:4[illegible] Initials JS

153. [O] JS Load and execute camera script **462TAMBL06** and **462TAMBR06**, which autofocuses and captures 1 frame with filter 0 at 100mm focal length. Insert the note "TARGET=DOT40,METROLOGY=[ID#]" in the GUI.

154. [M,O] JS Reposition the target to the 4 or 5 additional positions and capture identical frames saved as prefix **462TAMBL06** and **462TAMBR06**. For each run insert the note "TARGET=DOT40,METROLOGY=[ID#]" in the GUI.

155. [D] JS Record image names and parameters in Image Log.

156. [M,D, L] Notes: AF @ 6 meters

Focal Length 6 and Focus 2

Time	Zoom [mc2]	Focus FM1 [mc0]	Focus FM2 [mc0]	Position #	Meters	Suffix and Notes
	8652	2922	2988			
				223		~~[illegible]~~ 259
				224	6.25	261, 260
				225	6.5	262
				226	6.75	263
				227	7	264
				228	?	265
				229	5.5	266
				230	5.25	267
				281	5	268

157. [O] JS Load and execute camera script **462TAMBL06** and **462TAMBR06**, which autofocuses and captures 1 frame with filter 0 at 100mm focal length. Insert the note "TARGET=DOT40,METROLOGY=[ID#]" in the GUI.
158. [M,O] JS Reposition the target to the 4 or 5 additional positions and capture identical frames saved as prefix **462TAMBL06** and **462TAMBR06**. For each run insert the note "TARGET=DOT40,METROLOGY=[ID#]" in the GUI.
159. [D] JS Record image names and parameters in Image Log.
160. [M,D, L] Notes: AF @ 5 meters

Focal Length 6 and Focus 3

Time	Zoom [mc2]	Focus FM1 [mc0]	Focus FM2 [mc0]	Position #	Meter	Suffix and Notes
	8652	2856	2922			
				232	5	269
				233		270
				23·4	5.75	271
				235	6.0	272
				236	4.75n	273
				238	4.5	~~275~~ 274
				238	4.25m	~~276~~ 275
				249	4.0	276
				240		277

161. [O] JR Load and execute camera script **462TAMBL06** and **462TAMBR06**, which autofocuses and captures 1 frame with filter 0 at 100mm focal length. Insert the note "TARGET=DOT40,METROLOGY=[ID#]" in the GUI.
162. [M,O] JR Reposition the target to the 4 or 5 additional positions and capture identical frames saved as prefix **462TAMBL06** and **462TAMBR06**. For each run insert the note "TARGET=DOT40,METROLOGY=[ID#]" in the GUI.
163. [D] ____ Record image names and parameters in Image Log.
164. [M,D, L] Notes: AF @ 4 meter

Focal Length 6 and Focus 4 * ** re-focus

Time	Zoom [mc2]	Focus FM1 [mc0]	Focus FM2 [mc0]	Position #	meters	Suffix and Notes
11:16	8652	2748	2814			
				241		278
				242		279*
				243	4.0	280**
				244	4.25	
				245	4.5	
				246	4.75	
				247	5.0	

248 285, 286, 287

165. [O] ___ Load and execute camera script **462TAMBL06** and **462TAMBR06**, which autofocuses and captures 1 frame with filter 0 at 100mm focal length. Insert the note "TARGET=DOT40,METROLOGY=[ID#]" in the GUI.

166. [M,O] ___ Reposition the target to the 4 or 5 additional positions and capture identical frames saved as prefix **462TAMBL06** and **462TAMBR06**. For each run insert the note "TARGET=DOT40,METROLOGY=[ID#]" in the GUI.

167. [D] ___ Record image names and parameters in Image Log.

168. [M,D, L] Notes: AF @ 2 meters

Focal Length 6 and Focus 5

Time	Zoom [mc2]	Focus FM1 [mc0]	Focus FM2 [mc0]	Position #	Meter	Suffix and Notes
	8652	2262	2334	[illegible]		
				261	2	301
				262	2.25	302
				263	2.5	303
				264	2.75	304 - 305
				265	3.0	306
				266	1.75	307
				267	1.5	308
				268	1.25m	309
				269	1.0m	310

Data Validation

169. [V] ___ Run the geometric validation Jupyter notebook on the acquired data from the Right and Left Mastcam-Zs.

170. [V,D, L] Notes:

165. [O] JK Load and execute camera script **462TAMBL06** and **462TAMBR06**, which autofocuses and captures 1 frame with filter 0 at 100mm focal length. Insert the note "TARGET=DOT40,METROLOGY=[ID#]" in the GUI.

166. [M,O] JK Reposition the target to the 4 or 5 additional positions and capture identical frames saved as prefix **462TAMBL06** and **462TAMBR06**. For each run insert the note "TARGET=DOT40,METROLOGY=[ID#]" in the GUI.

167. [D] JK Record image names and parameters in Image Log.

168. [M,D, L] Notes: AF @ 1 meter

Focal Length 6 and Focus 5

Time	Zoom [mc2]	Focus FM1 [mc0]	Focus FM2 [mc0]	Position #	Meter	Suffix and Notes
	8652	1422	1488	—	—	—
				270	1.0	311 / 312
				271	1.25	313
				272	1.5	314
				273	1.75	315
				274	2.0	316
				275	.75	317

Data Validation

169. [V] ____ Run the geometric validation Jupyter notebook on the acquired data from the Right and Left Mastcam-Zs.

170. [V,D, L] Notes: ____

165. [O] JS Load and execute camera script **462TAMBL06** and **462TAMBR06**, which autofocuses and captures 1 frame with filter 0 at 100mm focal length. Insert the note "TARGET=DOT40,METROLOGY=[ID#]" in the GUI.

166. [M,O] JS Reposition the target to the 4 or 5 additional positions and capture identical frames saved as prefix **462TAMBL06** and **462TAMBR06**. For each run insert the note "TARGET=DOT40,METROLOGY=[ID#]" in the GUI.

167. [D] JS Record image names and parameters in Image Log.

168. [M,D, L] Notes: AF @ 3.0 meters
Note their was a glitch with the procedure
refer to Log 46 for referal

Focal Length 6 and Focus 5

Time	Zoom [mc2]	Focus FM1 [mc0]	Focus FM2 [mc0]	Position #	Suffix and Notes
	8652	2580	2646		
				252	292
				253	293
				254	294
				255	295
				256	296
				257	297
				258	298

259 299
260 300

Data Validation

169. [V] ____ Run the geometric validation Jupyter notebook on the acquired data from the Right and Left Mastcam-Zs.

170. [V,D, L] Notes: ____

110mm Focal Length 7 for the Mastcam-Zs

171. [M,T] JK Position the 40x40 dot geometric target to Position 1 as described in Table
172. [M] JK Measure the locations of the camera on the optics bench. Record the location measurements in the Image Log and tables below.

173. [D] JK Record the following temperatures:
- Left Camera CCD temp Missed
- Right Camera CCD temp Missed

174. [D,T] JK Take pictures the geometric target position and the whole test/GSE set-up.

Best Focus Model

f [mm]	FL [mc2]	D [meters]	FD' FM2-L [mc0]	FD' FM2-R [mc0]
110	9600	1.0	942	1110
110	9600	1.4	1512	1680
110	9600	1.7	1764	1932
110	9600	2.0	1938	2112
110	9600	2.3	2064	2244
110	9600	2.6	2166	2340
110	9600	3.0	2268	2442
110	9600	3.4	2346	2520
110	9600	4.0	2436	2610
110	9600	5.0	2532	2712
110	9600	6.0	2598	2778
110	9600	8.0	2682	2862
110	9600	10.0	2730	2910
110	9600	16.0	2808	2988
110	9600	30.0	2862	3042
110	9600	inf	2934	3108

165. [O] JR Load and execute camera script **462TAMBL06** and **462TAMBR06**, which autofocuses and captures 1 frame with filter 0 at 100mm focal length. Insert the note “TARGET=DOT40,METROLOGY=[ID#]” in the GUI.
166. [M,O] JR Reposition the target to the 4 or 5 additional positions and capture identical frames saved as prefix **462TAMBL06** and **462TAMBR06**. For each run insert the note “TARGET=DOT40,METROLOGY=[ID#]” in the GUI.
167. [D] JR Record image names and parameters in Image Log.
168. [M,D, L] Notes: AF @ 1 meter

Focal Length 6 and Focus 5

Time	Zoom [mc2]	Focus FM1 [mc0]	Focus FM2 [mc0]	Position #	Meter	Suffix and Notes
	9600	1176	1242	277	1.0 m	319
				278	[illegible] .75m	[illegible] 320
				279	1.25	321
				280	1.5 m	322
				281	1.75 m	323
				282	2.0	324
				283	2.25	325

Data Validation

169. [V] JR Run the geometric validation Jupyter notebook on the acquired data from the Right and Left Mastcam-Zs.
170. [V,D, L] Notes:

165. [O] JS Load and execute camera script **462TAMBL06** and **462TAMBR06**, which autofocuses and captures 1 frame with filter 0 at 100mm focal length. Insert the note "TARGET=DOT40,METROLOGY=[ID#]" in the GUI.

166. [M,O] JS Reposition the target to the 4 or 5 additional positions and capture identical frames saved as prefix **462TAMBL06** and **462TAMBR06**. For each run insert the note "TARGET=DOT40,METROLOGY=[ID#]" in the GUI.

167. [D] JS Record image names and parameters in Image Log.

168. [M,D, L] Notes: AF @ 2 meters

Focal Length 6 and Focus 5

Time	Zoom [mc2]	Focus FM1 [mc0]	Focus FM2 [mc0]	Position #	Meter	Suffix and Notes
	9600	2028	2100			
				284	2 m	326
				285	1.0	327
				286	1.25	328
				287	1.5	329
				288	1.75	330
				289	2.25	331
				290	2.50	332
				291	2.75	333
				292	3.0	334

Data Validation

169. [V] ____ Run the geometric validation Jupyter notebook on the acquired data from the Right and Left Mastcam-Zs.

170. [V,D, L] Notes:

175. [O] JK Load and execute camera script **462TAMBL07** and **462TAMBR07**, which autofocuses and captures 1 frame with filter 0 at 110mm focal length. Insert the note "TARGET=DOT40,METROLOGY=[ID#]" in the GUI.
176. [M,O] JK Reposition the target to the 4 or 5 additional positions and capture identical frames saved as prefix **462TAMBL07** and **462TAMBR07**. For each run insert the note "TARGET=DOT40,METROLOGY=[ID#]" in the GUI.
177. [D] JK Record image names and parameters in Image Log.
178. [M,D, L] Notes: AF @ 3 meters

Note: AF @ 1 meter and @ 2 meter on notes paged

Focal Length 7 and Focus 1

Time	Zoom [mc2]	Focus FM1 [mc0]	Focus FM2 [mc0]	Position #	Suffix and Notes
	9600	2340	2418	293	335
				294 2.0m	336
				295 2.25m	337
				296 2.5m	338
				297 2.75m	339
				298 3.25	340
				299 3.5	341
				300 3.75	342
				301 4.0m	343

179. [O] JK Load and execute camera script 462TAMBL07 and 462TAMBR07, which autofocuses and captures 1 frame with filter 0 at 110mm focal length. Insert the note "TARGET=DOT40,METROLOGY=[ID#]" in the GUI.

180. [M,O] JS Reposition the target to the 4 or 5 additional positions and capture identical frames saved as prefix 462TAMBL07 and 462TAMBR07. For each run insert the note "TARGET=DOT40,METROLOGY=[ID#]" in the GUI.

181. [D] JK Record image names and parameters in Image Log.

182. [M,D, L] Notes: AF @ 4 meter

\# look at log.

Focal Length 7 and Focus 2

Time	Zoom [mc2]	Focus FM1 [mc0]	Focus FM2 [mc0]	Position #	meters	Suffix and Notes
	9600	2508	2580	[illegible]		
12:58				302 #		344
				303 #		345
				304 #	[illegible]	346
				305	3.0	347
				306	3.25	348
				307	3.5	349
				308	3.75	350
				309	4.25	351
				310	4.5	352
				311	4.75 m	353
				312		354

Date 5/3 Time 1:14 Initials JS

183. [O] JS Load and execute camera script **462TAMBL07** and **462TAMBR07**, which autofocuses and captures 1 frame with filter 0 at 110mm focal length. Insert the note "TARGET=DOT40,METROLOGY=[ID#]" in the GUI.
184. [M,O] JS Reposition the target to the 4 or 5 additional positions and capture identical frames saved as prefix **462TAMBL07** and **462TAMBR07**. For each run insert the note "TARGET=DOT40,METROLOGY=[ID#]" in the GUI.
185. [D] ____ Record image names and parameters in Image Log.
186. [M,D, L] Notes: AF @ 5 meters

Focal Length 7 and Focus 3

Time	Zoom [mc2]	Focus FM1 [mc0] 2616	Focus FM2 [mc0] 2688	Position #	Suffix and Notes
	9600	~~[illegible]~~	~~[illegible]~~	—	—
1:15	|	|	|	313	355
	|	|	|	314/4m	356
	|	|	|	315 /4.25m	357
	|	|	|	316 /4.5m	358
	|	|	|	317 /4.75m	359
1:25	|	|	|	318 / 5.25m	360
		|		319 / 5.5m	361
				320/ 5.75	362

13:25
Date 9/3 Time 1:25 Initials JS

187. [O] JS Load and execute camera script **462TAMBL07** and **462TAMBR07**, which autofocuses and captures 1 frame with filter 0 at 110mm focal length. Insert the note "TARGET=DOT40,METROLOGY=[ID#]" in the GUI.

188. [M,O] JS Reposition the target to the 4 or 5 additional positions and capture identical frames saved as prefix **462TAMBL07** and **462TAMBR07**. For each run insert the note "TARGET=DOT40,METROLOGY=[ID#]" in the GUI.

189. [D] JS Record image names and parameters in Image Log.

190. [M,D, L] Notes: AF @ 6 meters

Focal Length 7 and Focus 4

Time	Zoom [mc2]	Focus FM1 [mc0]	Focus FM2 [mc0]	Position #	Suffix and Notes
	9600	2682	2754	—	—
				322	364
				~~822~~ 323/5.0	365
				~~3~~324/5.25	366
1:30				325/5.5	367
				326/5.75	368
				327/6.25	36
				328/6.5	~~369~~ 370
				329/6.75	371
				330/ 7.0	372
				331/	373

Date 5/3 Time ~~1:36 p~~ 13:36 Initials JS

191. [O] JS Load and execute camera script **462TAMBL07** and **462TAMBR07**, which autofocuses and captures 1 frame with filter 0 at 110mm focal length. Insert the note "TARGET=DOT40,METROLOGY=[ID#]" in the GUI.
192. [M,O] JS Reposition the target to the 4 or 5 additional positions and capture identical frames saved as prefix **462TAMBL07** and **462TAMBR07**. For each run insert the note "TARGET=DOT40,METROLOGY=[ID#]" in the GUI.
193. [D] JX Record image names and parameters in Image Log.
194. [M,D, L] Notes: AF @ 7 meters.

Focal Length 7 and Focus 5

Time	Zoom [mc2]	Focus FM1 [mc0]	Focus FM2 [mc0]	Position #	Suffix and Notes
	9600	2730	2808	—	—
13:38	↓	↓	↓	331	373
				332/6.0	374
				333/6.25m	375
				334/6.5	376
				335/6.75m	377
				336/7.25m	378
				337/7.3m	379

Data Validation

195. [V] ____ Run the geometric validation Jupyter notebook on the acquired data from the Right and Left Mastcam-Zs.
196. [V,D, L] Notes: ____

skip

Fixed Target Mech Cycling for the Mastcam-Zs

197. [M,T] ____ Position the 40x40 dot geometric target as close to 3 meters as possible.
198. [T] ____ Install infrared bright illumination and turn them on.
199. [D, L] Notes:____

____

____

200. [O,T] ____ Load and execute the script **466TAMBL10**, which captures Z-stacks of 16 focus distances (from 1 meter to infinity) for each non-solar filter with the **63mm** focal length. Insert note "TARGET=DOT40". The estimated duration is 15 minutes.
201. [O,T] ____ Load and execute the script **466TAMBR10**, which captures Z-stacks of 16 focus distances (from 1 meter to infinity) for each non-solar filter with the **63mm** focal length. Insert note "TARGET=DOT40". The estimated duration is 15 minutes.
202. [D] ____ Record image names and parameters in Image Log.
203. [D, L] Notes:____

____

____

Skip

204. [O,T] ____ Load and execute the script **466TAMBL06**, which captures Z-stacks of 16 focus distances (from 1 meter to infinity) for filter 0 with seven focal lengths. Insert note "TARGET=DOT40". The estimated duration is 12 minutes.
205. [O,T] ____ Load and execute the script **466TAMBR06**, which captures Z-stacks of 16 focus distances (from 1 meter to infinity) for filter 0 with seven focal lengths. Insert note "TARGET=DOT40".". The estimated duration is 12 minutes.
206. [D] ____ Record image names and parameters in Image Log.
207. [D, L] Notes:____

____

____

208. [O,T] ____ If time permits, load and execute the script **466TAMBL07**, which seven focal lengths in reverse order (110, 100, …) for 3 meter focus with 0 filter. Insert note "TARGET=DOT40". The estimated duration is 4 minutes.
209. [O,T] ____ If time permits, load and execute the script **466TAMBR07**, which seven focal lengths in reverse order (110, 100, …) for 3 meter focus with 0 filter. Insert note "TARGET=DOT40". The estimated duration is 4 minutes.
210. [D] ____ Record image names and parameters in Image Log.
211. [D, L] Notes:____

____

____

Shutdown Procedure

212. [D,T] [illegible] Take pictures of the test setup.
213. [D,O] [illegible] Review entries in Image Log, GSE command log, and image headers.
214. [D, L] [illegible] Review calibration procedure and ensure that each task is initialed.
215. [D, L] Notes: ______

216. [V, L] [illegible] Before making the decision to break down the test setup, ensure that adequate data were acquired for the test requirements. See "MastcamZCalPlan" for these requirements.
217. [V] Notes: ______

Data Validator (signature) [illegible]

Date 5/7/19 Time 3:00 PM

218. [V, L] [illegible] Give the go/no-go decision. Have enough data been acquired to fulfill test requirements? See "MastcamZCalPlan" for these requirements.
219. [D, L] [illegible] Update the Log Document.
220. [L] Notes: defer zoom/focus rack to later, if time available

Calibration Lead (signature) [illegible] Justin

Date 5/7/19 Time 5/3/19 3:00 pm

Date 5/3 Time 2:40 Initials CO

221. [O, L] CO Ensure that the camera and GSE are in a safe state.
222. [O,D] CO Review the Image Log with the documentarian. Exchange high-fives.
223. [O] Notes: ______

Camera Operator (signature) [signature]

Date 5/7/19 Time 1 pm

224. [T] CO If the next test does not require the target, position it away from the chamber or bench. Otherwise, be sure not to move it. The next test is ______.
225. [T] CO Ensure that all other test equipment is safely put away.
226. [T] Notes: ______

Technician (signature) [signature] Justin and Mark

Date 5/3/19 Time 2:40

227. [D, L] JK Double-check this procedure and ensure that the top of each page has valid data, time and initials.
228. [D] JK Photo-scan this document, save it on the cloud, and file the hard-copy in the Log Binder. Upload the digital pictures taken during this test in the appropriate archive on the cloud. The required links are on the Wiki.
229. [D] JK Double-check that every required cell the Image Log is accurately filled. When this is complete, print the Image Log and file it the Log Binder after this document.
230. [D] Notes: [illegible] - completed through morning shift (5/3)

Documentarian (signature) [signature]

Date 5/3/19 Time 2:40
