# Supplementary material for: Pre-Flight Calibration of the Mars 2020 Rover Mastcam Zoom (Mastcam-Z) Multispectral, Stereoscopic Imager
Source: Space Sci Rev. 2021 Feb 18;217(2):29. doi: 10.1007/s11214-021-00795-x (PMC7892537; doi:10.1007/s11214-021-00795-x)
Supplement: Supplementary file 1 — (ZIP 98.6 MB) [file 11214_2021_795_MOESM1_ESM.zip › CalPro_465-7_JR_Geometric_v2_04_FL48_TEMP.pdf]

JR Geometric Calibration Procedure for the Right and Left Mastcam-Z TVAC Testing at MSSS (Pro. 4.6.5-8)

[Procedure version 2.04, prepared by the Mastcam-Z calibration team at Cornell University]

These measurements are performed on the camera and at the temperature designated below as specified in the Mastcam-Z Calibration Plan,

Unit Under Test:

Left FM X **Right FM** X **EQM**____ **Other**____

These measurements are performed at temperature:

-35°C____ **-10°C** X **+5°C**____ **Ambient**____ **Other**____

These measurements are performed at,

MSSS X **ASU**____ **Other**____

Date 4/28/2019 **Start Time** 8:30 **End Time** 16:10

Estimated Duration 8.0 hours

Scheduled Start Time 8:30 **Sch. End Time** 1730

Calibration Lead [L] Jim Bell **Documentarian [D]** Megan Barrington

Camera Operator [O] Tex, Elsa Jensen **Technician [T]** Andy, Christian Tate, Winhold

Data Validator [V] Paul Corlies **Metrologist [M]** Informal, Christian Tate

Other ____

Date 4/28 Time 830 Initials [illegible]

Change Log

Version	Name	Change
v1_01<br>26 Sep 2018	C. Tate	(first draft)
v1_07<br>1 Nov 2018	C. Tate	Procedure edits prior to EQM testing
V1_07-JR<br>8 Nov 2018	G. Paar	Distances more precisely reflected, change mode from v06 to v07 kept, fixed focus consistently at 2 tables & figure automatically referenced
v1_10<br>13 Dec. 2018	C. Tate	Procedure edits after EQM testing
v2_04<br>28 April 2019	C. Tate	Approved version prior to FM testing

Document Approval

X [signature] 5/6/19

Approved by James Bell — Date
Mastcam-Z PI
Arizona State University

X [signature] 5/6/19

Approved by Alexander Hayes — Date
Mastcam-Z Calibration Working Group
Lead, Cornell University

Approved by Justin Maki — Date
Mastcam-Z Deputy PI and Investigation
Scientist, Jet Propulsion Laboratory

Christian Tate 4-30-19

Approved by Christian Tate — Date
Procedure Author
Cornell University

Approved by: — Date

Table of Contents

JR GEOMETRIC CALIBRATION PROCEDURE FOR THE RIGHT AND LEFT MASTCAM-Z TVAC TESTING AT MSSS (PRO. 4.6.5-8) 1

Change Log 2
Document Approval 2
Test Description 4
Software Preparation 4
Table 1. File naming convention for the camera script prefixes and frame filenames: "AAABBBBCDD" 4
Hardware Installation 6
Figure 1. ASU Floor Plan for Geometric Testing in the TVAC Chamber. The MSSS Floor Plan allows for similar target and source placements relative to the chamber window. 6
Explanation of the Semi-Random Orientations 8
Figure 2. An example of the camera's FOV (black) and JR dot target's semi-random positions (red) 8
100+ Target Positions for the 48mm Right and Left Mastcam-Zs 9
Data Validation 11
Fixed Target Positions for the 34mm Right Mastcam-Z (Scene 1) 12
Fixed Target Positions for the 63mm Right Mastcam-Z (Scene 2) 14
Fixed Target Positions for the 100mm Right Mastcam-Z (Scene 3) 16
Fixed Target Positions for the 34mm Left Mastcam-Z (Scene 4) 18
Fixed Target Positions for the 63mm Left Mastcam-Z (Scene 5) 20
Fixed Target Positions for the 100mm Left Mastcam-Z (Scene 6) 22
Data Validation 24
Time Check 1 24
Fixed Target Positions for the 26mm Right Mastcam-Z (Scene 7) 25
Fixed Target Positions for the 26mm Left Mastcam-Z (Scene 8) 27
Time Check 2 29
100+ Target Positions for the 63mm Right and Left Mastcam-Zs 30
Data Validation 32

SHUTDOWN PROCEDURE 33

Test Description

Excerpt from the Calibration Plan 4.6

> The objective of Geometric Calibration is to characterize the geometric distortion introduced by the Mastcam-Z optics into its images, and measure the effective focal length and field of view at each focus and zoom position. As the range of zoom positions available to Mastcam-Z represent a continuum, measurements will be acquired at a finite number of zoom settings and then interpolated to characterize distortion and other geometric parameters across the full zoom range. Targets should be imaged at ~50% full well using the Bayer RGB/805 nm (priority 1) and remaining non-solar filters (priority 3). The calibration data will be used to generate a geometric model for each camera. The camera models may exhibit wavelength dependence, so an attempt to measure the effect overall filters is desired (although not required).

Software Preparation

The software and files required for this test are prepared in advance of test day. This checklist ensures that the following are present, debugged, and executable: (1) all fast look scripts, (2) automated header generation of all relevant camera parameters, target positioning, and metadata, (3) all camera scripts that command the camera unit, and (4) the directories/file-paths pointing to the data repositories of this specific test.

Table 1. File naming convention for the camera script prefixes and frame filenames: "AAABBBBCDD"

Code	Name	Example	Value(s)
"AAA"	Calibration Plan Section	"465" = Cal. Plan 4.6.5 chapter 4, section 6, subsection 5	465-8
"BBBB"	Location of test or ASU Chamber temperature	"ATLO" = test at JPL ATLO, "TN10" = MSSS TVAC -10C, …	TEMP
"C"	Camera unit under test	"L" = Left Mastcam-Z, "R" = Right Mastcam-Z, "E" =EQ "C" =COTS	L/R
"DD"	Part of test	"00" = test set up, "01" = first part,…	00-13

Date 4/28 Time 835 Initials JFB

1. **[D]** ✓ Look up the daily calibration schedule and record the scheduled start and end time of this test on the cover page of this document. Also, fill out and double-check the other information on the cover page.
2. **[D]** ✓ Ensure that all supplemental manuals are on hand. These are,
   - Validator_Manual, Documentarian_Manual, MastcamZ_Data_Manual,
   - MastcamZCalPlan OK
3. **[D]** ✓ Ensure that the Image Log is present and ready to use. Find and open the Google Sheets file "Image_Log_46". There is a link on the Wiki.
4. **[V]** ✓ Check that all *Calgorithms* fast-look and validation scripts are present, up-to-date, and ready to analyze test output. Find and open the "Geometric_Calibration_46_Validation" Jupyter notebook. There is a link on the Wiki.
5. **[O]** ✓ Check that all camera scripts required for this test are present, up-to-date and ready to command the ground support equipment (GSE). These are,
   - **465TEMPR01 - 465TEMPR09, 465TEMPL01 - 465TEMPL09**
   - **466TEMPR01 - 466TEMPR13, 466TEMPL01 - 466TEMPL13**
   - **467TEMPR01 - 467TEMPR04, 467TEMPL01 - 467TEMPL04**

6. **[O,V,D, L]** Notes:

Date ~~10~~ 4/28 Time 840 Initials [illegible]

Hardware Installation

This procedure is for the ambient TVAC chamber testing at MSSS. Figure 1 shows the nominal layout of the TVAC chamber, workspace, Mastcam-Zs, ground support equipment (GSE), targets, sources, and other equipment necessary for this test if it happens at ASU. Although MSSS' cleanroom is different than ASU's, the placement of the targets and sources relative to the chamber window is similar.

Figure 1. ASU Floor Plan for Geometric Testing in the TVAC Chamber. The MSSS Floor Plan allows for similar target and source placements relative to the chamber window.

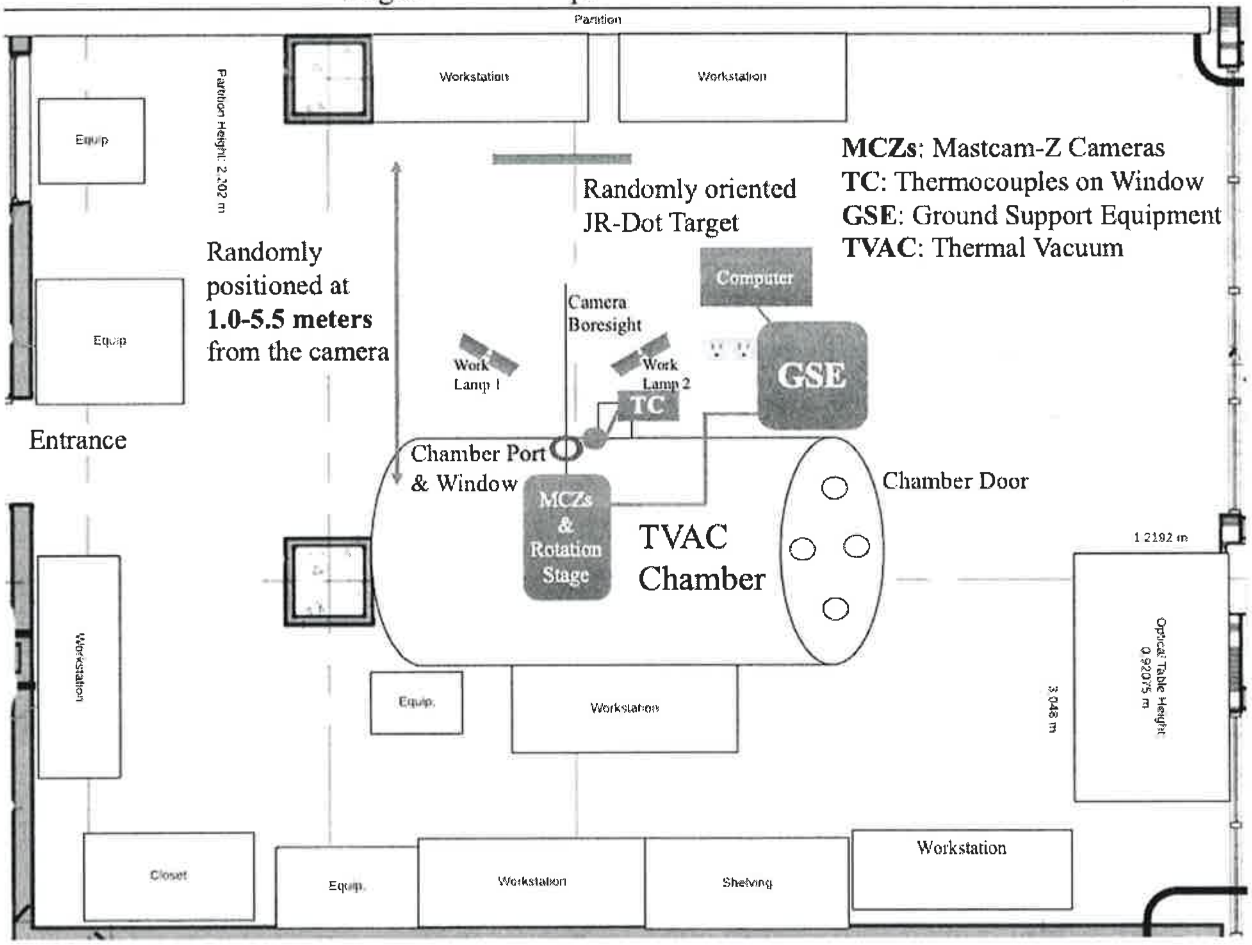

7. **[T,O, L]** ✓ Ensure that all personnel in the cleanroom are following the cleanroom practices for electrostatic discharge, proper clothing, and other safety concerns. See "ESD_Manual" and "Cleanroom_Manual".

Date 4/26 Time 845 Initials JFB

8. **[T]** ✓ Double check that nitrogen is flowing over the Mastcam-Zs or the window port.
9. **[O,T]** ✓ If not already done, mate the Right Mastcam-Z into the GSE. Follow the procedure in "MastcamZ_GSE_Manual".
10. **[T]** ✓ Verify that the thermocouples are turned on and properly reading out.
11. **[T]** ✓ Position the JR dot target approximately **2 meters** from the cameras
12. **[T]** ✓ Install the lamps and position them about 1 meter from the geometric target out of the camera's field of view (FOV). Power them on.
13. **[O,T]** ✓ Ensure that the camera unit and GSE wires are secure, kink-free, and do not present tripping hazards when the lights are turned off.
14. **[O,D]** ✓ Check the camera temperature and ensure nominal operation.
15. **[D]** ✓ Record the following environmental information:
    - Cleanroom temperature 26.6°C pressure Amb humidity 41%
16. **[O,D, L]** Notes:

____

____

____

Date 4/28 Time 845 Initials JHB

Explanation of the Semi-Random Orientations

Figure 2. An example of the camera's FOV (black) and JR dot target's semi-random positions (red)

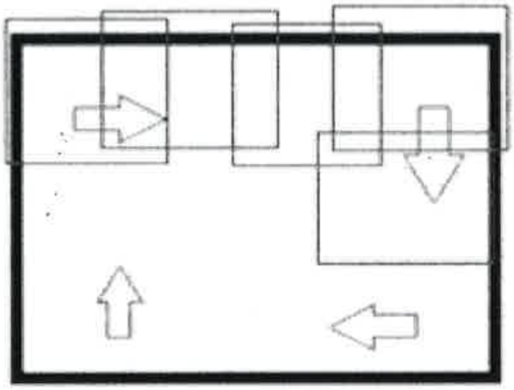

Figure 2 shows the desired orientations for the following tests that ask for a certain number of "semi-random orientations" of the JR dot target. The blue arrows show the motion of the target across the camera's FOV for optimal coverage. Note that some frames should be taken with the JR dot target rotated 90-degrees around the camera's line-of-sight.

17. **[O,T, L]** ✓ Capture a test frame with both cameras at 48mm to confirm that the chamber window does not clip the FOV (save images with prefixes **465TEMPR00** and **465TEMPL00**). If there is clipping, the next section will be taken at 63mm instead of 48mm.

18. **[D, L]** Notes:______________________________________

Date 4/21 Time 845 Initials JFB

100+ Target Positions for the 48mm Right and Left Mastcam-Zs

19. **[T]** ✓ Position the JR dot target approximately **2 meters** from the camera. Adjust lights accordingly.
20. **[D]** ✓ Record the following temperatures:
    - Chamber temp N/A Port temp N/A
    - Camera CCD temp −4.1°C Optics temp N/A
21. **[D,T]** ✓ Take digital pictures of the geometric target's position, and the whole test/GSE set-up.
22. **[O,T]** ✓ Capture test frames to find a standard exposure time (100 msec) for the 100 positions at ~~2~~ 3 OK meters focus. Save these test frames with the prefix name **465TEMPR00**, and update "var1" in the script **465TEMPR03** once this exposure time is found. UPDATE NOTE ✳
23. ✓ **[V,O,T]** Evaluate whether the target's dots are in-focus enough for discrimination. If the dots are too out-of-focus for JR's algorithm, move the target back.
24. **[O,T]** ✓ Capture test frames to find a standard exposure time (100 msec) for the 100 positions at ~~2~~ 3 ok meters focus. Save these test frames with the prefix name **465TEMPL00**, and update "var1" in the script **465TEMPL03** once this exposure time is found.
25. ✓ **[V,O,T]** Evaluate whether the target's dots are in-focus enough for discrimination. If the dots are too out-of-focus for JR's algorithm, move the target back.

CCD: −4.3°C

26. ✓ **[O,T]** ✓ Load and begin the script **465TEMPR03**, which captures 1 ~~frames~~ frame with filter 0 at **48mm** focal length one frame at a time, with a pause command between each frame.
27. ✓ **[O,T]** ✓ Load and begin the script **465TEMPL03**, which captures 1 ~~frames~~ frame with filter 0 at **48mm** focal length one frame at a time, with a pause command between each frame.

CCD: −5.7°C

Then →

28. **[O,T]** ✓ Capture **10 images** of the JR dot target in semi-random orientations (see Figure 2) normal to the camera approximately **1-meter** distance covering each edge of the camera's FOV. STARTS AT 9:07 am

✳ Be explicit − Move between images
FIRST TWO PAIRS AT SAME position.

Date 4/28 Time 9:08 Initials JFB

29. **[[redacted],T]** ✓ Capture **20 images** of the JR dot target in semi-random orientations (see Figure 2) normal to the camera approximately **2-meter** distance covering each edge of the camera's FOV. R CCD= -4.5°C, L= -5.7°C

30. **[O,T]** ✓ Capture **20 images** of the JR dot target in semi-random orientations (see Figure 2) normal to the camera approximately **3-meter** distance covering each edge of the camera's FOV. R CCD: -4.6°C L CCD: -5.9°C

31. **[O,T]** ✓ Capture **20 images** of the JR dot target in semi-random orientations (see Figure 2) normal to the camera approximately **4-meter** distance covering each edge of the camera's FOV. R CCD: -4.5°C L CCD: -5.9°C

32. **[O,T]** ✓ Capture **20 images** of the JR dot target in semi-random orientations (see Figure 2) normal to the camera approximately **5-meter** distance covering each edge of the camera's FOV. R CCD: -4.4°C L CCD: -5.8°C

SKIP. TIME... X 33. **[O,T]** ____ Capture **20 images** of the JR dot target in semi-random orientations (see Figure 2) normal to the camera as distant as possible covering each edge of the camera's FOV. *

NOT USEFUL. WE HAVE TO FOLLOW A SPECIFIC PROCEDURE

34. **[[redacted],T, L]** ✓ After more than 100 (150 would be perfect) usable frames have been captured, stop the prefix script.

35. **[D]** ✓ Record image names and parameters in Image Log.

36. **[D, L]** Notes: Test end at 11:09am; Final CCD Temps: R: -4.5°C L: -5.9°C

- Total ≈ 141 to 143 image pairs at all positions.

* DOCUMENTARIAN needs to work with TECHNICIAN to correct out-of-sync Left/Right pairs somewhere around/after pair #140...

Date 4/28 Time ~~11:10~~ 11:10 Initials JSP

Data Validation

37. **[V]** ✓ Run the "Geometric_46_Validation" Jupyter notebook on the acquired data for for the Right and Left Mastcam-Zs. This analysis can take place while the test continues.

38. **[V,D, L]** Notes: Good sampling of FOV in all distances!

ADDED #141 (Right) AND #143 (Left) "Group photo" shot to flush last JR Target image out of the GSE buffer
JIM, Tex, Christian, Andy...

Left camera homed and powered off at 11:25 am

OK

Fixed Target Positions for the 34mm Right Mastcam-Z (Scene 1)

39. **[M]** N/A If available, install the metrology equipment.
40. **[T]** ✓ Position the JR dot target approximately **1.7 meter** from the camera.
41. **[D]** ✓ Record the following temperatures:
    - Chamber temp N/A Port temp N/A
    - Camera CCD temp −4.6°C Optics temp N/A
42. **[D,T]** ✓ Take digital pictures of the geometric target's position, and the whole test/GSE set-up. *ADD: SET focal Length To 34mm
43. **[O,T]** ✓ Capture test frames to finely position the target to fill the **34mm** FOV and approximately centered in the 26mm FOV. Save these test frames with the prefix name **466TEMPR00**. ADD: Drive focus To 1.7 m
44. **[V,O,T]** ✓ Evaluate whether the target's dots are in-focus enough for discrimination.
45. **[M]** N/A Measure the locations of the geometric target and the camera.
46. **[M,D]** N/A Record the location measurements in the Image Log and tables below.

Target Location	Metrology ID#
Reference	
Top-Left Nest	
Top-Right Nest	
Bottom-Left Nest	
Bottom-Right Nest	

Camera/Chamber Location	Metrology ID#
Reference	
Nest 1	
Nest 2	
Nest 3	

47. **[M,D, L]** Notes: Distance To Target: ~~1.7M~~ 1.43M

NOTE DEFINE: DISTANCE FROM Bottom of FRONT of chamber port To Target center. NOT To cameras.

*NEEDS DIAGRAM

48. **[O,T]** ✓ Load and execute the script **466TEMPR06**, which captures Z-stacks of 16 focus distances (from 1 meter to infinity) for filter 0 with seven focal lengths. The estimated duration is 10 minutes.
49. **[D]** Record image names and parameters in Image Log.
50. **[O,T]** ✓ Load and execute the script **466TEMPR07**, which captures frames at 2-m focus distance for filter 0 with seven focal lengths in reverse. The estimated duration is 3 minutes.
51. **[O,T]** ✓ Load and execute the script **466TEMPR09**, which captures Z-stacks of 16 focus distances (from 1 meter to infinity) for each non-solar filter with the **34mm** focal length. The estimated duration is 10 minutes.
52. **[O,T]** ✓ If time permits, load and execute the script **467TEMPR01**, which auto-exposes and captures frames for ~140 ~~focal lengths~~ with filter 0 at a focus distance of 3 meters. The estimated duration is ~~12~~ 20 minutes.
53. **[D]** ✓ Record image names and parameters in Image Log.
54. **[D, L]** Notes:

STARTED #48 AT 11:32, CCD AT −4.5°C, finished 11:48, CCD −4.0°C
#50 AT 11:50, CCD AT −4.2°C, finished 11:54, CCD=−4.1°C
#51 AT 11:56, CCD AT −4.2°C, finished 12:12, CCD= −3.7°C
#52 AT 12:16, CCD @ −3.9°C, finished 12:25, CCD= ?

Date JFB Time 12:30 Initials 4/28

Fixed Target Positions for the 63mm Right Mastcam-Z (Scene 2)

55. [T] ✓ Position the JR dot target approximately **3 meters** from the camera.
56. [D] ✓ Record the following temperatures:
    - Chamber temp N/A Port temp N/A
    - Camera CCD temp -3.7°C Optics temp N/A
57. [D,T] ✓ Take digital pictures of the geometric target's position, and the whole test/GSE set-up.
58. [[redacted],T] ✓ Capture test frames to finely position the target to fill the **63mm** FOV and approximately centered in the 34mm FOV. Save these test frames with the prefix name **466TEMPR00**. *ADD: DRIVE FOCUS TO 3 M.
59. [M] N/A Measure the locations of the geometric target and the camera.
60. [M,D] ✓ Record the location measurements in the Image Log and tables below.

Target Location	Metrology ID#
Reference	
Top-Left Nest	
Top-Right Nest	
Bottom-Left Nest	
Bottom-Right Nest	

Camera/Chamber Location	Metrology ID#
Reference	
Nest 1	
Nest 2	
Nest 3	

61. [M,D, L] Notes: DISTANCE TO TARGET: 2.469 M

62. [O,T] ✓ Load and execute the script **466TEMPR06**, which captures Z-stacks of 16 focus distances (from 1 meter to infinity) for filter 0 with seven focal lengths. The estimated duration is ~~10~~ 14 minutes.
63. [D] ✓ Record image names and parameters in Image Log.

64. **[O,T]** ✓ Load and execute the script **466TEMPR10**, which captures Z-stacks of 16 focus distances (from 1 meter to infinity) for each non-solar filter with the **63mm** focal length. The estimated duration is 10 minutes.

65. **[O,T]** ✓ If time permits, load and execute the script **467TEMPR01**, which auto-exposes and captures frames for ~140 focal lengths with filter 0 at a focus distance of 3 meters. The estimated duration is ~~12~~ 20 minutes.

66. **[D]** ____ Record image names and parameters in Image Log.

67. **[D, L]** Notes: Started #62 at 12:52, CCD = -4.3°, Finished: 1:08, CCD: -3.9°C
#64 @ 1:11, CCD = -4.1°C, " 1:29, CCD: -3.7°C
#65 @ 1:33, CCD = -4.0°C, 1:51, CCD: -3.6°C

Date 4/24 Time 1145 Initials JFB

Fixed Target Positions for the 100mm Right Mastcam-Z (Scene 3)

68. **[T]** ✓ Position the JR dot target approximately **5 meters** from the camera.
69. **[D]** ✓ Record the following temperatures: Skip
    - Chamber temp ______________ Port temp ______________
    - Camera CCD temp ______________ Optics temp ______________
70. **[D,T]** ____ Take digital pictures of the geometric target's position, and the whole test/GSE set-up. * SET 100mm f/L, set focus To ~5m
71. **[[redacted],T]** ____ Capture test frames to finely position the target to fill the **100mm** FOV and approximately centered in the 63mm FOV. Save these test frames with the prefix name * **466TEMP~~L00~~**. R00
72. **[V,O,T]** Evaluate whether the target's dots are in-focus enough for discrimination.
73. **[M]** N/A Measure the locations of the geometric target and the camera.
74. **[M,D]** ✓ Record the location measurements in the Image Log and tables below.

Target Location	Metrology ID#
Reference	
Top-Left Nest	
Top-Right Nest	
Bottom-Left Nest	
Bottom-Right Nest	

Camera/Chamber Location	Metrology ID#
Reference	
Nest 1	
Nest 2	
Nest 3	

75. **[M,D, L]** Notes: ______________________________
    DISTANCE = 4.30 m
    ____

76. **[O,T]** ____ Load and execute the script **466TEMP~~L11~~** R11, which captures Z-stacks of 16 focus distances (from 1 meter to infinity) for each non-solar filter with the **100mm** focal length. The estimated duration is ~~10~~ 23 minutes.

Date 4/28 Time 2:45 Initials JFD

77. **[D]** ✓ Record image names and parameters in Image Log.
78. **[O,T]** ✓ Load and execute the script **467TEMPL04** (R04), which auto-exposes and captures frames for ~70 focal lengths with filter 0 at a focus distance of 5 meters. The estimated duration is 8 minutes.
79. **[O,T]** ____ If time permits, load and execute the script **466TEMPL06** (R06), which captures Z-stacks of 16 focus distances (from 1 meter to infinity) for filter 0 with seven focal lengths. The estimated duration is 10 minutes.
80. **[D]** ✓ Record image names and parameters in Image Log.
81. **[D, L]** Notes: Started #76 at 1:57pm, CCD: -3.7°C; Finished: 2:20, CCD: -3.7°C
#78 2:22 -3.8°C 2:33 -3.6°C
#79 2:34 -3.6°C

homed and powered down right camera at 2:52 pm

Fixed Target Positions for the 34mm Left Mastcam-Z (Scene 4)

82. **[M]** N/A If available, install the metrology equipment.
83. **[T]** ✓ Position the JR dot target approximately **1.7 meter** from the camera.
84. **[D]** ✓ Record the following temperatures:
    - Chamber temp N/A Port temp N/A
    - Camera CCD temp -6.1°C Optics temp N/A
85. **[D,T]** ✓ Take digital pictures of the geometric target's position, and the whole test/GSE set-up.
86. **[[redacted],T]** ✓ Capture test frames to finely position the target to fill the **34mm** FOV and approximately centered in the 26mm FOV. Save these test frames with the prefix name **466TEMPL00**.
87. **[V,O,T]** ✓ Evaluate whether the target's dots are in-focus enough for discrimination.
88. **[M]** ✓ Measure the locations of the geometric target and the camera.
89. **[M,D]** ✓ Record the location measurements in the Image Log and tables below.

**Target Location**	**Metrology ID#**
Reference	
Top-Left Nest	
Top-Right Nest	
Bottom-Left Nest	
Bottom-Right Nest	

**Camera/Chamber Location**	**Metrology ID#**
Reference	
Nest 1	
Nest 2	
Nest 3	

90. **[M,D, L]** Notes: DISTANCE = 1.61 M

91. **[O,T]** ✓ Load and execute the script **466TEMPL06**, which captures Z-stacks of 16 focus distances (from 1 meter to infinity) for filter 0 with seven focal lengths. The estimated duration is 10 minutes.
92. **[D]** Record image names and parameters in Image Log.
93. **[O,T]** ✓ Load and execute the script **466TEMPL07**, which captures frames at 2-m focus distance for filter 0 with seven focal lengths in reverse. The estimated duration is 3 minutes.
94. **[O,T]** ✓ Load and execute the script **466TEMPL09**, which captures Z-stacks of 16 focus distances (from 1 meter to infinity) for each non-solar filter with the **34mm** focal length. The estimated duration is 10 minutes.
95. **[O,T]** ✓ If time permits, load and execute the script **467TEMPL01**, which auto-exposes and captures frames for ~140 focal lengths with filter 0 at a focus distance of 3 meters. The estimated duration is ~~12~~ 20 minutes.
96. **[D]** ✓ Record image names and parameters in Image Log.
97. **[D, L]** Notes: STARTED #91 @ 2:57 CCD@ -5.8°; FINISHED @ 3:12 CCD@ -5.0°C

#93	3:14	-5.1°	3:18	-5.0°C
#94	3:19	-5.0°	3:34	-4.7°C
#95	3:35	-4.7°C	3:54	-4.8°C

Fixed Target Positions for the 63mm Left Mastcam-Z (Scene 5) OK

98. **[T]** ✓ Position the JR dot target approximately **3 meters** from the camera.
99. **[D]** ✓ Record the following temperatures:
    - Chamber temp N/A Port temp N/A
    - Camera CCD temp -5.2°C Optics temp N/A
100. **[D,T]** ✓ Take digital pictures of the geometric target's position, and the whole test/GSE set-up. * Set f/L To 63mm and focus To 3m
101. **[O,T]** ✓ Capture test frames to finely position the target to fill the **63mm** FOV and approximately centered in the 34mm FOV. Save these test frames with the prefix name **466TEMPL00**.
102. **[M]** N/A Measure the locations of the geometric target and the camera.
103. **[M,D]** ✓ Record the location measurements in the Image Log and tables below.

Target Location	Metrology ID#
Reference	
Top-Left Nest	
Top-Right Nest	
Bottom-Left Nest	
Bottom-Right Nest	

Camera/Chamber Location	Metrology ID#
Reference	
Nest 1	
Nest 2	
Nest 3	

104. **[M,D, L]** Notes: DISTANCE = 2.82M

105. **[O,T]** ✓ Load and execute the script **466TEMPL06**, which captures Z-stacks of 16 focus distances (from 1 meter to infinity) for filter 0 with seven focal lengths. The estimated duration is 10 minutes.
106. **[D]** ✓ Record image names and parameters in Image Log.

Date 4/28 Time 4:20p Initials JFB

107. **[O,T]** ✓ Load and execute the script **466TEMPL10**, which captures Z-stacks of 16 focus distances (from 1 meter to infinity) for each non-solar filter with the **63mm** focal length. The estimated duration is 10 minutes.

108. **[O,T]** ✓ If time permits, load and execute the script **467TEMPL01**, which auto-exposes and captures frames for ~140 focal lengths with filter 0 at a focus distance of 3 meters. The estimated duration is ~~12~~ 20 minutes.

109. **[D]** ✓ Record image names and parameters in Image Log.

110. **[D, L]** Notes: START #105 @ 4:05pm, CCD @ -5.2° ; Finished @ 4:21, CCD @ -4.7°
#107 4:23pm -4.8° 4:39pm -4.7°
#108 4:43pm -5.0° 5:02pm -4.7°

OK

Fixed Target Positions for the 100mm Left Mastcam-Z (Scene 6)

111. **[T]** ✓ Position the JR dot target approximately **5 meters** from the camera.
112. **[D]** ✓ Record the following temperatures:
    - Chamber temp N/A Port temp N/A
    - Camera/CCD temp -4.9°C Optics temp N/A
113. **[D,T]** ✓ Take digital pictures of the geometric target's position, and the whole test/GSE set-up. Set f/l to 100mm and focus to 5 meters
114. **[O,T]** ✓ Capture test frames to finely position the target to fill the **100mm** FOV and approximately centered in the 63mm FOV. Save these test frames with the prefix name **466TEMPL00**.
115. **[V,O,T]** Evaluate whether the target's dots are in-focus enough for discrimination.
116. **[M]** N/A Measure the locations of the geometric target and the camera.
117. **[M,D]** ✓ Record the location measurements in the Image Log and tables below.

Target Location	Metrology ID#
Reference	
Top-Left Nest	
Top-Right Nest	
Bottom-Left Nest	
Bottom-Right Nest	

Camera/Chamber Location	Metrology ID#
Reference	
Nest 1	
Nest 2	
Nest 3	

118. **[M,D, L]** Notes: DISTANCE = 4.38 m

119. **[O,T]** ✓ Load and execute the script **466TEMPL11**, which captures Z-stacks of 16 focus distances (from 1 meter to infinity) for each non-solar filter with the **100mm** focal length. The estimated duration is 10 minutes.

120. **[D]** ✓ Record image names and parameters in Image Log.
121. **[O,T]** ✓ Load and execute the script **467TEMPL04**, which auto-exposes and captures frames for ~70 focal lengths with filter 0 at a focus distance of 5 meters. The estimated duration is 8 minutes.
122. **[O,T]** ✓ If time permits, load and execute the script **466TEMPL06**, which captures Z-stacks of 16 focus distances (from 1 meter to infinity) for filter 0 with seven focal lengths. The estimated duration is ~~10~~ 20 minutes.
123. **[D]** ✓ Record image names and parameters in Image Log.
124. **[D, L]** Notes: start #119 @ 5:22, CCD = -5.1°; Finish @ 5:34, CCD = -4.6°
#121 5:41, -4.8°; 5:52, -4.7°
#122 5:55, -4.8°, 6:11, -4.5°

Data Validation

125. **[V]** _✓_ Run the "Geometric_46_Validation" Jupyter notebook on the acquired data for for the Right and Left Mastcam-Zs. This analysis can take place while the test continues.

126. **[V,D, L]** Notes: ____

Time Check 1

No time on 4/28

X

IF MORE THAN 2.0 HOURS AHEAD OF SCHEDULED END, CONTINUE. OTHERWISE, SKIP TO THE FIXED TARGET TESTS.

Scheduled End Time	Current Time	Time Ahead of Scheduled End
-	=	

127. **[D, L]** _✓_ Record the time in the table above and determine if there is time for more testing.

128. **[D, L]** Notes: ____

Skip

Fixed Target Positions for the 26mm Right Mastcam-Z (Scene 7)

129. **[T]** ____ Position the JR dot target approximately **1.2 meters** from the camera.
130. **[D]** ____ Record the following temperatures:
    - Chamber temp ______________ Port temp ______________
    - Camera CCD temp ______________ Optics temp ______________
131. **[D,T]** ____ Take digital pictures of the geometric target's position, and the whole test/GSE set-up.
132. **[O,T]** ____ Capture test frames to finely position the target to be filled and centered in the 26mm FOV. Save these test frames with the prefix name **466TEMPR00**.
133. **[V,O,T]** ____ Evaluate whether the target's dots are in-focus enough for discrimination.
134. **[D]** ____ Record the target distance measurement(s) in the Image Log.
135. **[D]** ____ Record image names and parameters in Image Log.
136. **[D, L]** Notes:____
    ____
    ____

137. **[M]** ____ Measure the locations of the geometric target and the camera.
138. **[M,D]** ____ Record the location measurements in the Image Log and tables below.

Date ______ Time ______ Initials ______

Skip

**Target Location**	**Metrology ID#**
Reference	
Top-Left Nest	
Top-Right Nest	
Bottom-Left Nest	
Bottom-Right Nest	

**Camera/Chamber Location**	**Metrology ID#**
Reference	
Nest 1	
Nest 2	
Nest 3	

139. **[M,D, L]** Notes: ____

____

____

140. **[[redacted],T]** ____ Load and execute the script **466TEMPR13**, which captures Z-stacks of 16 focus distances (from 1 meter to infinity) for each non-solar filter with the **26mm** focal length. The estimated duration is 10 minutes.
141. **[D]** Record image names and parameters in Image Log.
142. **[O,T]** ____ If time permits, load and execute the script **466TEMPR06**, which captures Z-stacks of 16 focus distances (from 1 meter to infinity) for filter 0 with seven focal lengths. The estimated duration is 10 minutes.
143. **[D]** Record image names and parameters in Image Log.
144. **[D, L]** Notes:____

____

____

Fixed Target Positions for the 26mm Left Mastcam-Z (Scene 8)

Skip

145. **[T]** ____ Position the JR dot target approximately **1.2 meters** from the camera.
146. **[D]** ____ Record the following temperatures:
    - Chamber temp _______________ Port temp _______________
    - Camera CCD temp _______________ Optics temp _______________
147. **[D,T]** ____ Take digital pictures of the geometric target's position, and the whole test/GSE set-up.
148. **[O,T]** ____ Capture test frames to finely position the target to be filled and centered in the 26mm FOV. Save these test frames with the prefix name **466TEMPR00**.
149. **[V,O,T]** ____ Evaluate whether the target's dots are in-focus enough for discrimination.
150. **[D]** ____ Record the target distance measurement(s) in the Image Log.
151. **[D]** ____ Record image names and parameters in Image Log.
152. **[D, L]** Notes:____

    ____

    ____

153. **[M]** ____ Measure the locations of the geometric target and the camera.
154. **[M,D]** ____ Record the location measurements in the Image Log and tables below.

Date ______ Time ______ Initials ______

Skip

Target Location	Metrology ID#
Reference	
Top-Left Nest	
Top-Right Nest	
Bottom-Left Nest	
Bottom-Right Nest	

Camera/Chamber Location	Metrology ID#
Reference	
Nest 1	
Nest 2	
Nest 3	

155. **[M,D, L]** Notes: ____

____

____

156. **[O,T]** ____ Load and execute the script **466TEMPR13**, which captures Z-stacks of 16 focus distances (from 1 meter to infinity) for each non-solar filter with the **26mm** focal length. The estimated duration is 10 minutes.
157. **[D]** Record image names and parameters in Image Log.
158. **[O,T]** ____ If time permits, load and execute the script **466TEMPR06**, which captures Z-stacks of 16 focus distances (from 1 meter to infinity) for filter 0 with seven focal lengths. The estimated duration is 10 minutes.
159. **[D]** Record image names and parameters in Image Log.
160. **[D, L]** Notes:____

____

____

Date ______ Time ______ Initials ______

Time Check 2 NO TIME ON 4/28

X

IF MORE THAN 1.0 HOUR AHEAD OF SCHEDULED END, CONTINUE. OTHERWISE, SKIP TO THE SHUTDOWN PROCEDURE.

Scheduled End Time	Current Time	Time Ahead of Scheduled End
-	=	

161. **[D, L]** ____ Record the time in the table above and determine if there is time for more testing

162. **[D, L]** Notes: ____

____

____

100+ Target Positions for the 63mm Right and Left Mastcam-Zs

Skip

163. **[T]** ____ Position the JR dot target approximately **3 meters** from the camera. Adjust lights accordingly.

164. **[D]** ____ Record the following temperatures:
   - Chamber temp ______________ Port temp ______________
   - Camera CCD temp ______________ Optics temp ______________

165. **[D,T]** ____ Take digital pictures of the geometric target's position, and the whole test/GSE set-up.

166. **[[redacted],T]** ____ Capture test frames to find a standard exposure time for the 100 positions at 3 meters focus. Save these test frames with the prefix name **465TEMPR00**, and update "var1" in the script **465TEMPR04** once this exposure time is found.

167. **[V,[redacted],T]** Evaluate whether the target's dots are in-focus enough for discrimination. If the dots are too out-of-focus for JR's algorithm, move the target back.

168. **[O,T]** ____ Capture test frames to find a standard exposure time for the 100 positions at 3 meters focus. Save these test frames with the prefix name **465TEMPL00**, and update "var1" in the script **465TEMPL04** once this exposure time is found.

169. **[V,O,T]** Evaluate whether the target's dots are in-focus enough for discrimination. If the dots are too out-of-focus for JR's algorithm, move the target back.

170. **[[redacted],T]** ____ Load and begin the script **465TEMPR04**, which captures frames with filter 0 at **63mm** focal length one frame at a time, with a pause command between each frame.

171. **[O,T]** ____ Load and begin the script **465TEMPL04**, which captures frames with filter 0 at **63mm** focal length one frame at a time, with a pause command between each frame.

172. **[O,T]** ____ Capture **10 images** of the JR dot target in semi-random orientations (see Figure 2) normal to the camera approximately **1-meter** distance covering each edge of the camera's FOV.

Skip

173. **[O,T]** ____ Capture **20 images** of the JR dot target in semi-random orientations (see Figure 2) normal to the camera approximately **2-meter** distance covering each edge of the camera's FOV.
174. **[O,T]** ____ Capture **20 images** of the JR dot target in semi-random orientations (see Figure 2) normal to the camera approximately **3-meter** distance covering each edge of the camera's FOV.
175. **[O,T]** ____ Capture **20 images** of the JR dot target in semi-random orientations (see Figure 2) normal to the camera approximately **4-meter** distance covering each edge of the camera's FOV.
176. **[O,T]** ____ Capture **20 images** of the JR dot target in semi-random orientations (see Figure 2) normal to the camera approximately **5-meter** distance covering each edge of the camera's FOV.
177. **[O,T]** ____ Capture **20 images** of the JR dot target in semi-random orientations (see Figure 2) normal to the camera as distant as possible covering each edge of the camera's FOV.
178. **[[redacted],T, L]** ____ After more than 100 usable frames have been captured, stop the prefix script.
179. **[D]** ____ Record image names and parameters in Image Log.
180. **[D, L]** Notes:____

____

____

Date 4/28 Time 6:10 Initials [illegible]

Data Validation

181. **[V]** ____ Run the "Geometric_46_Validation" Jupyter notebook on the acquired data for the Right and Left Mastcam-Zs. This analysis can take place while the test continues.

182. **[V,D, L]** Notes: All good!

Date 4/28 Time 6:10 Initials CT

Shutdown Procedure

183. **[D,T]** CT Take digital pictures of this page and the test setup.
184. **[D,O]** CO Review entries in Image Log, GSE command log, and image headers.
185. **[D, L]** CO Review calibration procedure and ensure that each task is initialed.
186. **[D, L]** Notes: ___

187. **[V, L]** CO Before making the decision to break down the test setup, ensure that adequate data were acquired for the test requirements. See "MastcamZCalPlan" for these requirements.
188. **[V]** Notes: ___

Data Validator (signature) [signature]

Date 4/28/19 Time 8:10 PM

189. **[V, L]** CO Give the go/no-go decision. Have enough data been acquired to fulfill test requirements? See "MastcamZCalPlan" for these requirements.
190. **[D, L]** CO Update the Log Document.
191. **[L]** Notes: ___

Calibration Lead (signature) [signature]

Date 4/28/19 Time 6:00 pm

192. [[redacted], L] [illegible] Ensure that the camera and GSE are in a safe state.
193. [O,D] [illegible] Review the Image Log with the documentarian. Exchange high-fives.
194. [[redacted]] Notes: ____________________

Camera Operator (signature) x [illegible] Elsa or Tex

Date 5/7/19 Time 1pm

195. [T] [illegible] If the next test does not require the target, position it away from the chamber or bench. Otherwise, be sure not to move it. The next test is Solar Flats.
196. [T] [illegible] Ensure that all other test equipment is safely put away.
197. [T] Notes: ____________________

Technician (signature) [illegible]

Date 5/3/19 Time 10:20

198. [D, L] [illegible] Double-check this procedure and ensure that the top of each page has valid data, time and initials.
199. [D] [illegible] Photo-scan this document, save it on the cloud, and file the hard-copy in the Log Binder. Upload the digital pictures taken during this test in the appropriate archive on the cloud. The required links are on the Wiki.
200. [D] [illegible] Double-check that every required cell the Image Log is accurately filled. When this is complete, print the Image Log and file it the Log Binder after this document.
201. [D] Notes: ____________________

Documentarian (signature) x [illegible] Megan and Christian

Date 5/7/19 Time 13:00
