# Supplementary material for: Pre-Flight Calibration of the Mars 2020 Rover Mastcam Zoom (Mastcam-Z) Multispectral, Stereoscopic Imager
Source: Space Sci Rev. 2021 Feb 18;217(2):29. doi: 10.1007/s11214-021-00795-x (PMC7892537; doi:10.1007/s11214-021-00795-x)
Supplement: Supplementary file 1 — (ZIP 98.6 MB) [file 11214_2021_795_MOESM1_ESM.zip › CalPro_465-7_JR_Geometric_v2_06_TVAC_Ambient.pdf]

Date 4/30 Time 13:10 Initials [signature]

JR Geometric Calibration Procedure for the Right and Left Mastcam-Z Ambient TVAC Testing at MSSS (Pro. 4.6.5-7)

[Procedure version 2.06, prepared by the Mastcam-Z calibration team at Cornell University]

These measurements are performed on the camera and at the Temperature designated below as specified in the Mastcam-Z Calibration Plan,

Unit Under Test:

Left FM __X__ **Right FM** __X__ **EQM**_____ **Other**_____________

These measurements are performed at Temperature:

-35°C_______ **-10°C**_______ **+5°C**_______ **Ambient**__X__ **Other**___________

These measurements are performed at,

MSSS__X__ **ASU**_______ **Other**________

Date 4/30 **Start Time** 15:40 **End Time** 18:00

Estimated Duration 4.0 hours

Scheduled Start Time 15:00 **Sch. End Time** 19:00

Calibration Lead [L] HERKENHOFF **Documentarian [D]** WINHOLD, VAN BEEK

Camera Operator [[redacted]] VAN BEEK, TK, KW **Technician [T]** ~~[illegible]~~ TATE, Winhold

Data Validator [V] CORLIES **Metrologist [M]** N/A

Other ______________________

Change Log

Version	Name	Change
v1_01<br>26 Sep 2018	C. Tate	(first draft)
v1_07<br>1 Nov 2018	C. Tate	Procedure edits prior to EQM testing
V1_07-JR<br>8 Nov 2018	G. Paar	Distances more precisely reflected, change mode from v06 to v07 kept, fixed focus consistently at 2 tables & figure automatically referenced
v1_10<br>13 Dec. 2018	C. Tate	Procedure edits after EQM testing
v2_05<br>30 April 2019	C. Tate	Approved version prior to FM testing

Document Approval

Approved by James Bell — Date
Mastcam-Z PI
Arizona State University

5/6/19
Approved by Alexander Hayes — Date
Mastcam-Z Calibration Working Group
Lead, Cornell University

Approved by Justin Maki — Date
Mastcam-Z Deputy PI and Investigation
Scientist, Jet Propulsion Laboratory

Christian Tate 4/30/19
Approved by Christian Tate — Date
Procedure Author
Cornell University

4/30/19
Approved by: — Date
KEN HERKENHOFF

Table of Contents

JR GEOMETRIC CALIBRATION PROCEDURE FOR THE RIGHT AND LEFT MASTCAM-Z AMBIENT TVAC TESTING AT MSSS (PRO. 4.6.5-7) **1**

CHANGE LOG 2
DOCUMENT APPROVAL 2
TEST DESCRIPTION 4
SOFTWARE PREPARATION 4
Table 1. File naming convention for the camera script prefixes and frame filenames: "AAABBBBCDD" *4*
HARDWARE INSTALLATION 6
Figure 1. ASU Floor Plan for Geometric Testing in the TVAC Chamber. The MSSS Floor Plan allows for similar target and source placements relative to the chamber window. *6*
FIXED TARGET POSITIONS FOR THE 63MM RIGHT AND LEFT MASTCAM-Z (SCENE 7) 8
DATA VALIDATION 14
TIME CHECK 1 .... **ERROR! BOOKMARK NOT DEFINED.**
EXPLANATION OF THE SEMI-RANDOM ORIENTATIONS 8
Figure 2. An example of the camera's FOV (black) and JR dot target's semi-random positions (red) *8*
100+ TARGET POSITIONS FOR THE 48MM RIGHT AND LEFT MASTCAM-ZS 8
DATA VALIDATION 10

SHUTDOWN PROCEDURE **15**

Test Description

Excerpt from the Calibration Plan 4.6

> The objective of Geometric Calibration is to characterize the geometric distortion introduced by the Mastcam-Z optics into its images and measure the effective focal length and field of view at each focus and zoom position. As the range of zoom positions available to Mastcam-Z represent a continuum, measurements will be acquired at a finite number of zoom settings and then interpolated to characterize distortion and other geometric parameters across the full zoom range. Targets should be imaged at ~50% full well using the Bayer RGB/805 nm (priority 1) and remaining non-solar filters (priority 3). The calibration data will be used to generate a geometric model for each camera.

Software Preparation

The software and files required for this test are prepared in advance of test day. This checklist ensures that the following are present, debugged, and executable: (1) all fast look scripts, (2) automated header generation of all relevant camera parameters, target positioning, and metadata, (3) all camera scripts that command the camera unit, and (4) the directories/file-paths pointing to the data repositories of this specific test.

Table 1. File naming convention for the camera script prefixes and frame filenames: "AAABBBBCDD"

Code	Name	Example	Value(s)
"AAA"	Calibration Plan Section	"465" = Cal. Plan 4.6.5 chapter 4, section 6, subsection 5	465-7
"BBBB"	Location of test or ASU Chamber Temperature	"ATLO" = test at JPL ATLO, "TN10" = MSSS TVAC -10C, …	TAMB
"C"	Camera unit under test	"L" = Left Mastcam-Z, "R" = Right Mastcam-Z, "E" =EQ "C" =COTS	L/R
"DD"	Part of test	"00" = test set up, "01" = first part,…	00-13

1. **[D]** [illegible] Look up the daily calibration schedule and record the scheduled start and end time of this test on the cover page of this document. Also, fill out and double-check the other information on the cover page.

2. **[D]** ___ Ensure that all supplemental manuals are on hand. These are,
    - Validator_Manual, Documentarian_Manual, MastcamZ_Data_Manual,
    - MastcamZCalPlan
3. **[D]** ___ Ensure that the Image Log is present and ready to use. Find and open the Google Sheets file "Image_Log_46". There is a link on the Wiki.
4. **[V]** ___ Check that all *Calgorithms* fast-look and validation scripts are present, up-to-date, and ready to analyze test output. Find and open the "Geometric_Calibration_46_Validation" Jupyter notebook. There is a link on the Wiki.
5. **[O]** ___ Check that all camera scripts required for this test are present, up-to-date and ready to command the ground support equipment (GSE). These are,
    - **465TAMBR01 - 465TAMBR09, 465TAMBL01 - 465TAMBL09**
    - **466TAMBR01 - 466TAMBR13, 466TAMBL01 - 466TAMBL13**
    - **467TAMBR01 - 467TAMBR04, 467TAMBL01 - 467TAMBL04**
6. **[O,V,D, L]** Notes:

Date 4/30/19 Time 15:51 Initials [illegible]

Hardware Installation

This procedure is for the ambient TVAC chamber testing at MSSS. Figure 1 shows the nominal layout of the TVAC chamber, workspace, Mastcam-Zs, ground support equipment (GSE), targets, sources, and other equipment necessary for this test if it happens at ASU. Although MSSS' cleanroom is different than ASU's, the placement of the targets and sources relative to the chamber window is similar.

Figure 1. ASU Floor Plan for Geometric Testing in the TVAC Chamber. The MSSS Floor Plan allows for similar target and source placements relative to the chamber window.

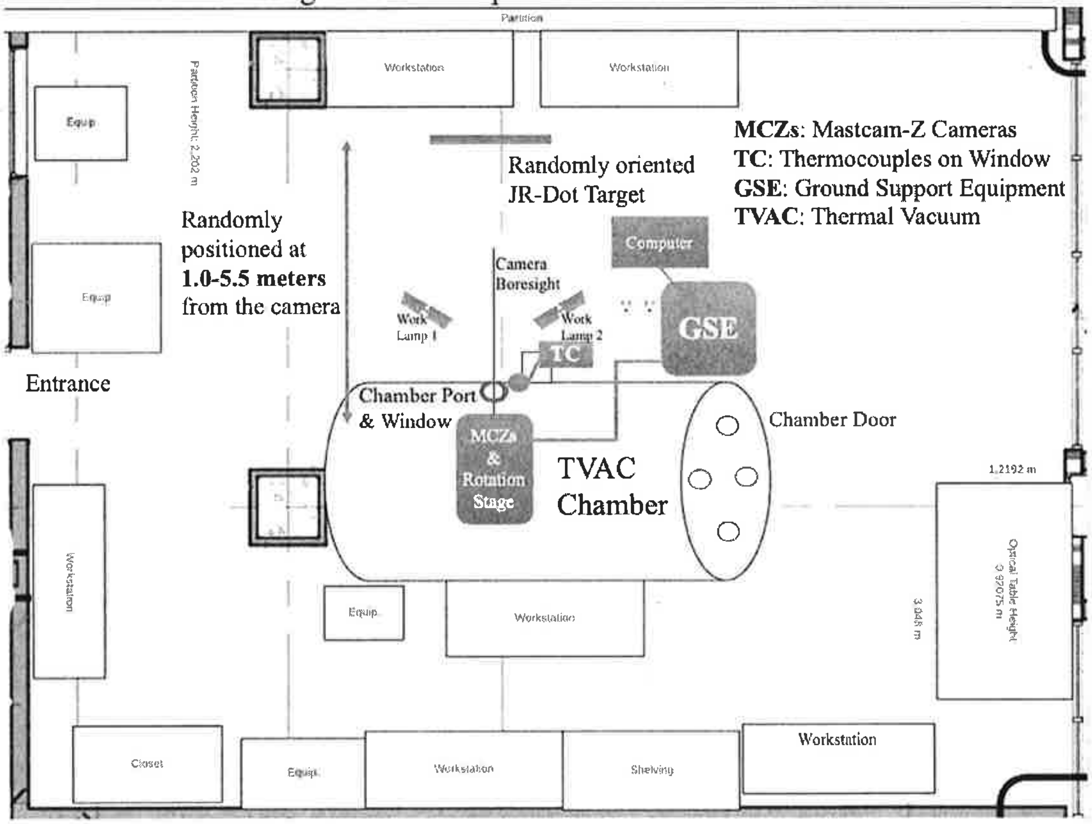

7. **[T,O, L]** [illegible] Ensure that all personnel in the cleanroom are following the cleanroom practices for electrostatic discharge, proper clothing, and other safety concerns. See "ESD_Manual" and "Cleanroom_Manual".
8. **[T]** [illegible] Double check that nitrogen is flowing over the Mastcam-Zs or the window port.

Date 4/30 Time ______ Initials [initials]

9. **[O,T]** ____ If not already done, mate the Right Mastcam-Z into the GSE. Follow the procedure in "MastcamZ_GSE_Manual". SKIP
10. **[T]** [initials] Verify that the thermocouples are turned on and properly reading out.
11. **[T]** [initials] Position the JR dot target approximately **3 meters** from the cameras
12. **[T]** [initials] Install the lamps and position them about 1 meter from the geometric target out of the camera's field of view (FOV). Power them on.
13. **[O,T]** [initials] Ensure that the camera unit and GSE wires are secure, kink-free, and do not present tripping hazards when the lights are turned off.
14. **[O,D]** [initials] Check the camera Temperature and ensure nominal operation.
15. **[D]** [initials] Record the following environmental information:
    - Cleanroom Temperature 25.0 pressure _______ humidity 45%
16. **[O,D, L]** Notes:

L: 26.8 R: 27.4°C

____

____

Explanation of the Semi-Random Orientations

Figure 2. An example of the camera's FOV (black) and JR dot target's semi-random positions (red)

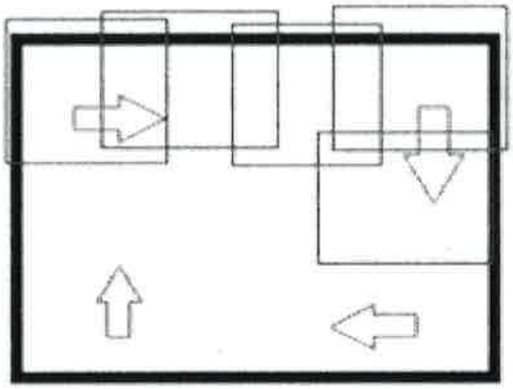

Figure 2 shows the desired orientations for the following tests that ask for a certain number of "semi-random orientations" of the JR dot target. The blue arrows show the motion of the target across the camera's FOV for optimal coverage. Note that some frames should be taken with the JR dot target rotated 90-degrees around the camera's line-of-sight.

100+ Target Positions for the 48mm Right and Left Mastcam-Zs

17. **[T]** Position the JR dot target approximately **3 meters** from the camera. Adjust lights accordingly.
18. **[D]** Record the following Temperatures:
    - Chamber Temp ambient Port Temp N/A
    - Camera CCD Temp 26.8, 27.4°C Optics Temp N/A
19. **[D,T]** Take digital pictures of the geometric target's position, and the whole test/GSE set-up.
20. **[[redacted],T]** Capture test frames to find a standard exposure time for the 100 positions at 3 meters focus. Save these test frames with the prefix name **465TAMBR00**, and update "var1" in the script **465TAMBR03** once this exposure time is found. 143 ms
21. **[V,O,T]** Evaluate whether the target's dots are in-focus enough for discrimination. If the dots are too out-of-focus for JR's algorithm, move the target back.

Date 4/30 Time 16:05 Initials [illegible]

22. [O,T] Capture test frames to find a standard exposure time for the 100 positions at 3 meters focus. Save these test frames with the prefix name **465TAMBL00**, and update "var1" in the script **465TAMBL03** once this exposure time is found. 151 ms

23. **[V,O,T]** Evaluate whether the target's dots are in-focus enough for discrimination. If the dots are too out-of-focus for JR's algorithm, move the target back.

24. [O,T] Load and begin the script **465TAMBR03**, which captures frames with filter 0 at **48mm** focal length one frame at a time, with a pause command between each frame.

25. [[redacted],T] Load and begin the script **465TAMBL03**, which captures frames with filter 0 at **48mm** focal length one frame at a time, with a pause command between each frame.

26. [[redacted],T] Capture **10 images** of the JR dot target in semi-random orientations (see Figure 2) normal to the camera approximately **1-meter** distance covering each edge of the camera's FOV.

27. [O,T] Capture **20 images** of the JR dot target in semi-random orientations (see Figure 2) normal to the camera approximately **2-meter** distance covering each edge of the camera's FOV. Adjust lighting if necessary, to keep the frames at approximately 50% full-well.

28. [O,T] Capture **20 images** of the JR dot target in semi-random orientations (see Figure 2) normal to the camera approximately **3-meter** distance covering each edge of the camera's FOV. Adjust lighting if necessary, to keep the frames at approximately 50% full-well. 9 IMAGES TAKEN AT 3.5m

29. [[redacted],T] Capture **20 images** of the JR dot target in semi-random orientations (see Figure 2) normal to the camera approximately **4-meter** distance covering each edge of the camera's FOV. Adjust lighting if necessary, to keep the frames at approximately 50% full-well. NEED L SIDE OF RT EYE + R SIDE OF LEFT EYE.

30. [[redacted],T] Capture **30 images** of the JR dot target in semi-random orientations (see Figure 2) normal to the camera approximately **5-meter** distance covering each edge of the camera's FOV. Adjust lighting if necessary, to keep the frames at approximately 50% full-well.

Date 4/30 Time 17:20 Initials [illegible]

✓31. **[V,O,T]** Evaluate whether the target's dots are evenly distributed over each fields of view.

32. **[O,T, L]** [illegible] After more than 100 usable frames have been captured, stop the prefix script.

33. **[D]** [illegible] Record image names and parameters in Image Log.

34. **[D, L]** Notes: 144 MAX SUFFIX

Data Validation

35. **[V]** [illegible] Run the "Geometric_46_Validation" Jupyter notebook on the acquired data for for the Right and Left Mastcam-Zs. This analysis can take place while the test continues.

36. **[V,D, L]** Notes: COVERED ALL CORNERS, BOTH EYES

Date 4/30 Time 17:23 Initials [illegible]

Fixed Target Positions for the 63mm Right and Left Mastcam-Z (Scene 7)

37. **[T]** [illegible] Position the JR dot target approximately **3 meters** from the camera.
38. **[D]** [illegible] Record the following Temperatures:
    - Chamber Temp 29.8°C Port Temp N/A
    - Camera CCD Temp 29.9, 30.7°C Optics Temp N/A
39. **[D,T]** [illegible] Take digital pictures of the geometric target's position, and the whole test/GSE set-up.
40. **[O,T]** [illegible] Capture test frames to finely position the target centered in the 34mm FOV of both cameras. Save these test frames with the prefix name **466TAMBR00** and **466TAMBL00**.

SKIP {
41. **[M]** ____ Measure the locations of the geometric target and the camera.
42. **[M,D]** ____ Record the location measurements in the Image Log and tables below.

Target Location	Metrology ID#
Reference	
Top-Left Nest	
Top-Right Nest	
Bottom-Left Nest	
Bottom-Right Nest	↑

Camera/Chamber Location	Metrology ID#
Reference	0.38 m Camera-Port dist.
Nest 1	
Nest 2	
Nest 3	

43. **[M,D, L]** Notes: 2.08 m PORT-TARGET DISTANCE

BREAK DURING DEBUG LEFT CAMERA GUI

SKIP

44. ~~[O,T] ____ For **466TAMBL06/466TAMBR06** and **466TAMBL10/466TAMBR10**, delete the first four long distance filter positions of each 16 Z-stack.~~

Skipping until tomorrow

45. **[O,T]** ____ Load and execute the script **466TAMBL06**, which captures Z-stacks of 12 focus distances (from 1 meter to infinity) for filter 0 with seven focal lengths. The estimated duration is 10 minutes.
46. **[O,T]** ____ Load and execute the script **466TAMBR06**, which captures Z-stacks of 12 focus distances (from 1 meter to infinity) for filter 0 with seven focal lengths. The estimated duration is 10 minutes.
47. **[D]** ____ Record image names and parameters in Image Log.
48. **[D, L]** Notes:______________________________

____

____

49. **[O,T]** ____ Load and execute the script **466TAMBL10**, which captures Z-stacks of 12 focus distances (from 1 meter to infinity) for each non-solar filter with the **63mm** focal length. The estimated duration is 10 minutes.
50. **[O,T]** ____ Load and execute the script **466TAMBR10**, which captures Z-stacks of 12 focus distances (from 1 meter to infinity) for each non-solar filter with the **63mm** focal length. The estimated duration is 10 minutes.
51. **[D]** ____ Record image names and parameters in Image Log.
52. **[D, L]** Notes:______________________________

____

____

Skip

53. **[O,T]** ____ If time permits, load and execute the script **467TAMBL01**, which auto-exposes and captures frames for ~140 focal lengths with filter 0 at a focus distance of 3 meters. The estimated duration is 16 minutes.
54. **[O,T]** ____ If time permits, load and execute the script **467TAMBR01**, which auto-exposes and captures frames for ~140 focal lengths with filter 0 at a focus distance of 3 meters. The estimated duration is 16 minutes.
55. **[D]** ____ Record image names and parameters in Image Log.
56. **[D, L]** Notes:____

____

____

57. [T] ____ **Be sure that the target does not move. Ensure that proper warning is posted for the people who may come into the room in our absence. Post a DO NOT MOVE sign.**

Date 4/30 Time 6:10 Initials [illegible]

Data Validation

Skip

58. **[V]** ____ Run the "Geometric_46_Validation" Jupyter notebook on the acquired data for for the Right and Left Mastcam-Zs. This analysis can take place while the test continues.
59. **[V,D, L]** Notes: ____

Date 4/30 Time 6:10 Initials [signature]

Shutdown Procedure

60. **[D,T]** [initials] Take digital pictures of the test setup.
61. **[D,O]** [initials] Review entries in Image Log, GSE command log, and image headers.
62. **[D, L]** [initials] Review calibration procedure and ensure that each task is initialed.
63. **[D, L]** Notes: __________________________________

SKIP

64. **[V, L]** ____ ~~Before making the decision to break down the test setup, ensure that adequate data were acquired for the test requirements. See "MastcamZCalPlan" for these requirements.~~
65. **[V]** Notes: __________________________________

Data Validator (signature) [signature]

Date 4/30/19 Time 6:15 PM

66. **[V, L]** [initials] Give the go/no-go decision. Have enough data been acquired to fulfill test requirements? See "MastcamZCalPlan" for these requirements.
67. **[D, L]** [initials] Update the Log Document.
68. **[L]** Notes: PROCEDURE HALTED DUE TO PROBLEM WITH LEFT CAMERA GUI

Calibration Lead (signature) [signature]

Date 4/30/19 Time 18:15

Date 4/30 Time 6:10 Initials CT

69. **[[redacted], L]** TK Ensure that the camera and GSE are in a safe state.
70. **[[redacted],D]** TK Review the Image Log with the documentarian. Exchange high-fives.
71. **[O]** Notes: ____

Camera Operator (signature) [signature]

Date 5/1/2019 Time 11:38

72. **[T]** CT If the next test does not require the target, position it away from the chamber or bench. Otherwise, be sure not to move it. The next test is JR geo. cal. (same)
73. **[T]** CT Ensure that all other test equipment is safely put away.
74. **[T]** Notes: ____

Technician (signature) Christian Dato

Date 4-30-19 Time 6:12

75. **[D, L]** [initials] Double-check this procedure and ensure that the top of each page has valid data, time and initials.
76. **[D]** ____ Photo-scan this document, save it on the cloud, and file the hard-copy in the Log Binder. Upload the digital pictures taken during this test in the appropriate archive on the cloud. The required links are on the Wiki. Skip
77. **[D]** ____ Double-check that every required cell the Image Log is accurately filled. When this is complete, print the Image Log and file it the Log Binder after this document. Skip
78. **[D]** Notes: ____

Documentarian (signature) [signature]

Date 4/30/19 Time 6:15
