# Supplementary material for: Pre-Flight Calibration of the Mars 2020 Rover Mastcam Zoom (Mastcam-Z) Multispectral, Stereoscopic Imager
Source: Space Sci Rev. 2021 Feb 18;217(2):29. doi: 10.1007/s11214-021-00795-x (PMC7892537; doi:10.1007/s11214-021-00795-x)
Supplement: Supplementary file 1 — (ZIP 98.6 MB) [file 11214_2021_795_MOESM1_ESM.zip › CalPro_465-7_JR_Geometric_v2_11.pdf]

Date 5/4 Time 15:50 Initials [illegible]

JR Geometric Calibration Procedure for the Right and Left Mastcam-Z Ambient Cleanroom Testing at MSSS (Pro. 4.6.5-7)

[Procedure version 2.11, prepared by the Mastcam-Z calibration team at Cornell University]

These measurements are performed on the camera and at the Temperature designated below as specified in the Mastcam-Z Calibration Plan,

Unit Under Test:

Left FM __X__ **Right FM** __X__ **EQM**_____ **Other**____________

These measurements are performed at Temperature:

-35°C______ **-10°C**______ **+5°C**______ **Ambient** __X__ **Other**__________

These measurements are performed at,

MSSS __X__ **ASU**_______ **Other**________

Date 5/4/19 **Start Time** 15:50 **End Time** 23:59

Estimated Duration 14.0 hours

Scheduled Start Time 7:30 **Sch. End Time** 19:30

Calibration Lead [L] KEN Herkenhoff **Documentarian [D]** ALEXIS, NATALIE

Camera Operator [O] JASON, DARIAN **Technician [T]** ANDY, MEGAN Barrington

Data Validator [V] MASON, TINA **Metrologist [M]** ———

Other ____________

Date 5/4 Time 16:00 Initials [illegible]

Change Log

Version	Name	Change
v1_01<br>26 Sep 2018	C. Tate	(first draft)
v1_07<br>1 Nov 2018	C. Tate	Procedure edits prior to EQM testing
V1_07-JR<br>8 Nov 2018	G. Paar	Distances more precisely reflected, change mode from v06 to v07 kept, fixed focus consistently at 2 tables & figure automatically referenced
v1_10<br>13 Dec. 2018	C. Tate	Procedure edits after EQM testing
v2_11<br>4 May 2019	G. Paar<br>C. Tate	Approved version prior to FM testing

Document Approval

Approved by James Bell — Date
Mastcam-Z PI
Arizona State University

Approved by Alexander Hayes — Date 5/6/19
Mastcam-Z Calibration Working Group Lead, Cornell University

Approved by Justin Maki — Date
Mastcam-Z Deputy PI and Investigation Scientist, Jet Propulsion Laboratory

Approved by Ken Herkenhoff — Date 5/4/19
Mastcam-Z Co-Investigator
USGS

Approved by — Date

Approved by Christian Tate — Date 5/4/19
Procedure Author
Cornell University

Table of Contents

JR GEOMETRIC CALIBRATION PROCEDURE FOR THE RIGHT AND LEFT MASTCAM-Z AMBIENT CLEANROOM TESTING AT MSSS (PRO. 4.6.5-7) **1**

Change Log 2
Document Approval 2
Test Description 4
Software Preparation 4
Table 1. File naming convention for the camera script prefixes and frame filenames: "AAABBBBCDD" 4
Hardware Installation 6
Figure 1. MSSS Floor Plan for Geometric Testing in the TVAC Chamber. 6
Table 2. The 16 canonical focus positions for each of the 7 canonical focal lengths 8
Positions for the 78, 100, 110mm to constrain the Mastcam-Zs infinity focus (Scene 12) 12
Data Validation 13
Fixed Target Positions for the 34mm Right and Left Mastcam-Z (Scene 14) 14
Data Validation 16
Fixed Target Positions for the 63mm Right and Left Mastcam-Z (Scene 15) 17
Data Validation 20
Fixed Target Positions for the 100mm Right and Left Mastcam-Z (Scene 16) 21
Data Validation 22
Fixed Target Positions for the 26mm Right and Left Mastcam-Z (Scene 17) 23
Data Validation 24
Explanation of the Semi-Random Orientations 25
Figure 2. An example of the camera's FOV (black) and JR dot target's semi-random positions (red) 25
100+ Target Positions for the 34mm Mastcam-Zs (Scene 18) 26
Data Validation 28
100+ Target Positions for the 48mm Right and Left Mastcam-Zs (Scene 19) 29
Data Validation 31
100+ Target Positions for the 100mm Right and Left Mastcam-Zs (Scene 20) 32
Data Validation 34
100+ Target Positions for the 26mm Right and Left Mastcam-Zs (Scene 21) 35
Data Validation 37
100+ Target Positions for the 63mm Right and Left Mastcam-Zs (Scene 22) 38
Data Validation 40
100+ Target Positions for the 110mm Right and Left Mastcam-Zs (Scene 23) 41
Data Validation 43
100+ Target Positions for the 78mm Right and Left Mastcam-Zs (Scene 24) 44
Data Validation 46

SHUTDOWN PROCEDURE **47**

Test Description

Excerpt from the Calibration Plan 4.6

> The objective of Geometric Calibration is to characterize the geometric distortion introduced by the Mastcam-Z optics into its images and measure the effective focal length and field of view at each focus and zoom position. As the range of zoom positions available to Mastcam-Z represent a continuum, measurements will be acquired at a finite number of zoom settings and then interpolated to characterize distortion and other geometric parameters across the full zoom range. Targets should be imaged at ~50% full well using the Bayer RGB/805 nm (priority 1) and remaining non-solar filters (priority 3). The calibration data will be used to generate a geometric model for each camera.

Software Preparation

The software and files required for this test are prepared in advance of test day. This checklist ensures that the following are present, debugged, and executable: (1) all fast look scripts, (2) automated header generation of all relevant camera parameters, target positioning, and metadata, (3) all camera scripts that command the camera unit, and (4) the directories/file-paths pointing to the data repositories of this specific test.

Table 1. File naming convention for the camera script prefixes and frame filenames: "AAABBBBCDD"

Code	Name	Example	Value(s)
"AAA"	Calibration Plan Section	"465" = Cal. Plan 4.6.5 chapter 4, section 6, subsection 5	465-7
"BBBB"	Location of test or ASU Chamber Temperature	"ATLO" = test at JPL ATLO, "TN10" = MSSS TVAC -10C, …	TAMB
"C"	Camera unit under test	"L" = Left Mastcam-Z, "R" = Right Mastcam-Z, "E" =EQ "C" =COTS	L/R
"DD"	Part of test	"00" = test set up, "01" = first part,…	00-13

1. **[D]** ACH Look up the daily calibration schedule and record the scheduled start and end time of this test on the cover page of this document. Also, fill out and double-check the other information on the cover page.

Date 5/4 Time 16:00 Initials CO

2. **[D]** AG LJ Ensure that all supplemental manuals are on hand. These are,
   - Validator_Manual, Documentarian_Manual, MastcamZ_Data_Manual,
   - MastcamZCalPlan
3. **[D]** AG LJ Ensure that the Image Log is present and ready to use. Find and open the Google Sheets file "Image_Log_46". There is a link on the Wiki.
4. **[V]** AGH Check that all *Calgorithms* fast-look and validation scripts are present, up-to-date, and ready to analyze test output. Find and open the "Geometric_Calibration_46_Validation" Jupyter notebook. There is a link on the Wiki.
5. **[O]** AGH Check that all camera scripts required for this test are present, up-to-date and ready to command the ground support equipment (GSE). These are,
   - **465TAMBR01 - 465TAMBR09, 465TAMBL01 - 465TAMBL09**
   - **466TAMBR01 - 466TAMBR13, 466TAMBL01 - 466TAMBL13**
   - **467TAMBR01 - 467TAMBR04, 467TAMBL01 - 467TAMBL04**

6. **[O,V,D, L]** Notes:

____

____

____

Hardware Installation

This procedure is for the ambient TVAC chamber testing at MSSS. Figure 1 shows the nominal layout of the TVAC chamber, workspace, Mastcam-Zs, ground support equipment (GSE), targets, sources, and other equipment necessary.

Figure 1. MSSS Floor Plan for Geometric Testing in the TVAC Chamber.

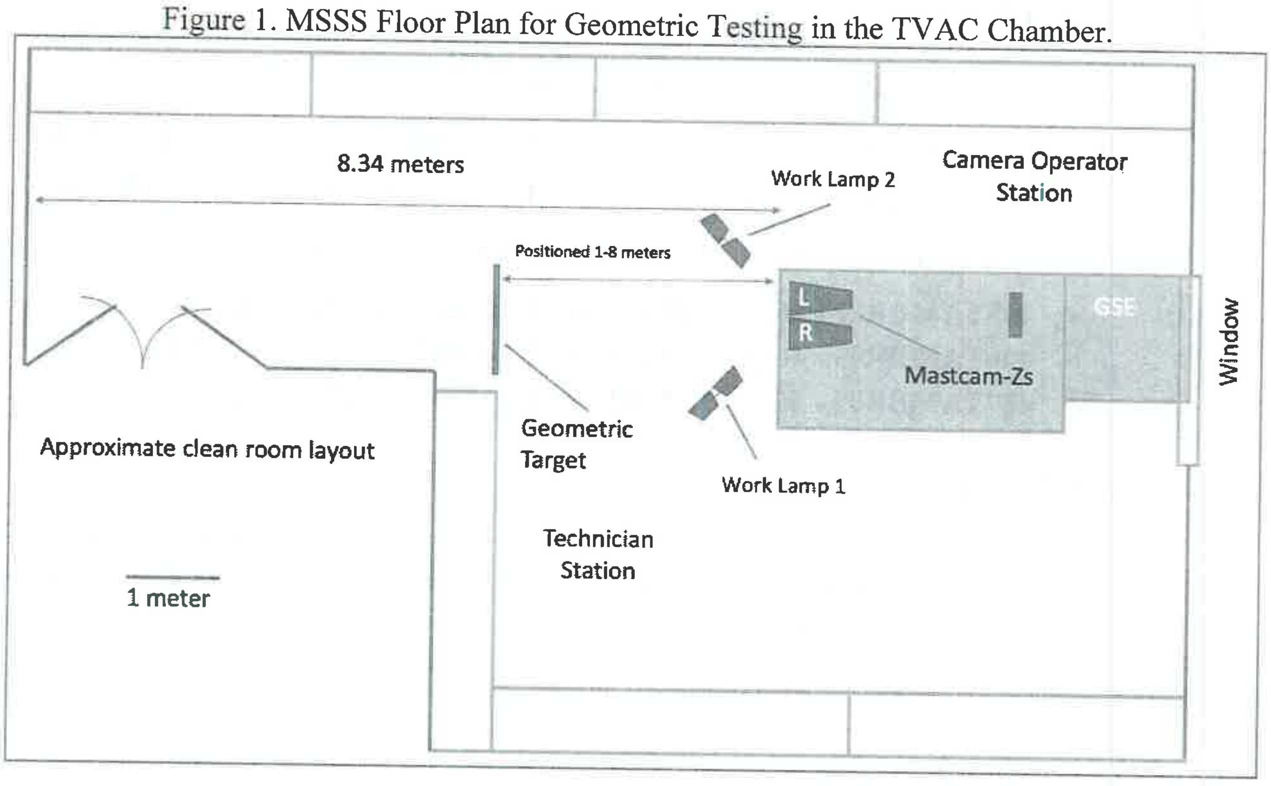

7. **[T,O, L]** ____ Ensure that all personnel in the cleanroom are following the cleanroom practices for electrostatic discharge, proper clothing, and other safety concerns. See “ESD_Manual” and “Cleanroom_Manual”.
8. **[T]** ____ Double check that the ionizers are blowing over the Mastcam-Zs.
9. **[O,T]** ____ Ensure that the camera unit and GSE wires are secure, kink-free, and do not present tripping hazards.
10. **[T]** ____ Install the JR dot target on the tripod. → Use natural targets as well for ∞ focus tests
11. **[T]** ____ Install the blue and infrared bright lamps. Position them about in front of the geometric target out of the camera’s field of view (FOV) with a high enough phase angle to prevent specular reflections. Power them on.

Date 5/4 Time 16:00 Initials [illegible]

12. **[O,D]** [illegible] Check the camera Temperature and ensure nominal operation.
13. **[D]** [illegible] Record the following environmental information:
    - Cleanroom Temperature ~~[illegible]~~ 68° F pressure _______ humidity 66 %
14. **[O,D, L]** Notes:

____

____

____

Date 5/4 Time 15:00 Initials [illegible]

Table 2. The 16 canonical focus positions for each of the 7 canonical focal lengths

f [mm]	FL [mc2]	D [meters]	FD' FM2-Left [mc0]	FD' FM1-Right [mc0]
26	0	1.0	-3540	-3762
26	0	1.4	-2940	-3138
26	0	1.7	-2676	-2868
26	0	2.0	-2490	-2676
26	0	2.3	-2358	-2532
26	0	2.6	-2250	-2424
26	0	3.0	-2142	-2316
26	0	3.4	-2064	-2226
26	0	4.0	-1968	-2130
26	0	5.0	-1866	-2022
26	0	6.0	-1794	-1950
26	0	8.0	-1710	-1860
26	0	10.0	-1656	-1806
26	0	16.0	-1578	-1728
26	0	30.0	-1518	-1662
26	0	inf	-1452	-1596

f [mm]	FL [mc2]	D [meters]	FD' FM2-Left [mc0]	FD' FM1-Right [mc0]
34	2448	1.0	-1326	-1278
34	2448	1.4	-732	-684
34	2448	1.7	-474	-420
34	2448	2.0	-288	-240
34	2448	2.3	-156	-102
34	2448	2.6	-54	0
34	2448	3.0	36	114
34	2448	3.4	138	192
34	2448	4.0	228	282
34	2448	5.0	330	384
34	2448	6.0	402	456
34	2448	8.0	486	540
34	2448	10.0	540	594
34	2448	16.0	618	672
34	2448	30.0	678	732
34	2448	inf	768	828

f [mm]	FL [mc2]	D [meters]	FD' FM2-Left [mc0]	FD' FM1-Right [mc0]
48	3834	1.0	-108	36
48	3834	1.4	480	630
48	3834	1.7	738	882
48	3834	2.0	918	1062
48	3834	2.3	1056	1194
48	3834	2.6	1158	1296
48	3834	3.0	1266	1398
48	3834	3.4	1344	1476
48	3834	4.0	1434	1566
48	3834	5.0	1536	1668
48	3834	6.0	1608	1734
48	3834	8.0	1692	1824
48	3834	10.0	1740	1872
48	3834	16.0	1818	1950
48	3834	30.0	1878	2004
48	3834	inf	1908	2034
f [mm]	FL [mc2]	D [meters]	FD' FM2-Left [mc0]	FD' FM1-Right [mc0]
63	5196	1.0	696	894
63	5196	1.4	1278	1464
63	5196	1.7	1536	1716
63	5196	2.0	1716	1890
63	5196	2.3	1848	2022
63	5196	2.6	1950	2124
63	5196	3.0	2058	2226
63	5196	3.4	2136	2304
63	5196	4.0	2226	2388
63	5196	5.0	2328	2490
63	5196	6.0	2394	2556
63	5196	8.0	2484	2640
63	5196	10.0	2532	2688
63	5196	16.0	2610	2766
63	5196	30.0	2670	2820
63	5196	inf	2784	2922

f [mm]	FL [mc2]	D [meters]	FD' FM2-Left [mc0]	FD' FM1-Right [mc0]
79	6720	1.4	1746	1932
79	6720	1.7	1998	2178
79	6720	2.0	2178	2352
79	6720	2.3	2310	2484
79	6720	2.6	2412	2580
79	6720	3.0	2514	2682
79	6720	3.4	2592	2760
79	6720	4.0	2682	2850
79	6720	5.0	2784	2946
79	6720	6.0	2850	3012
79	6720	8.0	2934	3096
79	6720	10.0	2988	3144
79	6720	16.0	3060	3216
79	6720	30.0	3120	3276
79	6720	inf	3174	3330
f [mm]	FL [mc2]	D [meters]	FD' FM2-Left [mc0]	FD' FM1-Right [mc0]
100	8652	1.0	1170	1338
100	8652	1.4	1740	1908
100	8652	1.7	1992	2160
100	8652	2.0	2166	2334
100	8652	2.3	2298	2460
100	8652	2.6	2394	2562
100	8652	3.0	2502	2664
100	8652	3.4	2580	2742
100	8652	4.0	2664	2826
100	8652	5.0	2766	2928
100	8652	6.0	2832	2994
100	8652	8.0	2916	3078
100	8652	10.0	2964	3126
100	8652	16.0	3042	3198
100	8652	30.0	3096	3258
100	8652	inf	3168	3336

Handwritten annotations beside the 100 mm rows:
- MTF: 3054 2970
- 3114 [illegible]
- 3006 Auto focus

Date 5/4 Time 16:00 Initials CP

f [mm]	FL [mc2]	D [meters]	FD' FM2-Left [mc0]	FD' FM1-Right [mc0]
110	9600	1.0	942	1110
110	9600	1.4	1512	1680
110	9600	1.7	1764	1932
110	9600	2.0	1938	2112
110	9600	2.3	2064	2244
110	9600	2.6	2166	2340
110	9600	3.0	2268	2442
110	9600	3.4	2346	2520
110	9600	4.0	2436	2610
110	9600	5.0	2532	2712
110	9600	6.0	2598	2778
110	9600	8.0	2682	2862
110	9600	10.0	2730	2910
110	9600	16.0	2808	2988
110	9600	30.0	2862	3042
110	9600	inf	2934	3108

MTF	~~[illegible]~~	~~[illegible]~~
Autofocus	2874	2766

Date 5/4 Time 16:00 Initials CB

Positions for the 78, 100, 110mm to constrain the Mastcam-Zs infinity focus (Scene 12)

Use actual target on wall.

15. **[T]** CB Position the JR ~~dot target approximately **8 meters**~~ from the camera.
16. **[D]** CB Record the following temperatures:
    - Left Mastcam-Z CCD temp 21.2
    - Right Mastcam-Z CCD temp 21.9
17. **[D,T]** CB Take digital pictures of the geometric target's position, and the whole test/GSE set-up.
18. ~~**[O,T]** CB Capture test frames to finely position the target centered in the 78mm (9) FOV of both cameras at the pre-determined infinity focus positions (see Table 2). Save these test frames with the prefix name **466TAMBL00** and **466TAMBR00**.~~
19. ~~**[M]** ____ Measure the locations of the geometric target and the camera.~~
20. **[M,D]** ____ Record the location measurements in the Image Log and tables below.
21. **[M,D, L]** Notes: ______________________________

22. **[O,T]** A64 Capture test frames to finely position the target centered in the ~~78~~ 79mm FOV of both cameras at the pre-determined infinity focus positions (see Table 2). Save these test frames with the prefix name **466TAMBL00** and **466TAMBR00**.
23. **[O,T]** A64 Capture test frames to finely position the target centered in the 100mm FOV of both cameras at the pre-determined infinity focus positions (see Table 2). Save these test frames with the prefix name **466TAMBL00** and **466TAMBR00**.
24. **[O,T]** A64 Capture test frames to finely position the target centered in the 110mm FOV of both cameras at the pre-determined infinity focus positions (see Table 2). Save these test frames with the prefix name **466TAMBL00** and **466TAMBR00**.

Date 5/4 Time 16:00 Initials CO

Data Validation

25. [V] A6H Run the "Geometric_46_Validation" Jupyter notebook on the acquired data for the Right and Left Mastcam-Zs. This analysis can take place while the test continues.
26. [V, L] A6H Determine if the dots are resolvable at infinity. Yes, they will be resolvable
27. [V,D, L] Notes: ______

______

______

28. [T] CO If time permits and the dots were resolvable in the above test, position the JR dot target approximately **6 meters** from the camera and recapture the above images at infinity focus. Call this **Scene 13** if performed. SKIP

Date 5/4 Time — Initials [illegible]

Fixed Target Positions for the 34mm Right and Left Mastcam-Z (Scene 14)

29. **[T]** AH Position the JR dot target approximately **2 meters** from the camera and finely adjust it to maximize the dots visible in both Mastcam-Z.
30. **[D]** AH Record the following temperatures:
    - Left Mastcam-Z CCD temp 23.0°C
    - Right Mastcam-Z CCD temp 23.5°C
31. **[D,T]** AH Take digital pictures of the geometric target's position, and the whole test/GSE set-up.
32. **[O,T]** AH Capture test frames to finely position the target centered in the 34mm ~~FOV~~ EFL of both cameras. Save these test frames with the prefix name **466TAMBL00** and **466TAMBR00**. SN 003 JR DOT
33. **[M]** AH Measure the locations of the geometric target and the camera.
34. **[M,D]** AH Record the location measurements in the Image Log and tables below.
35. **[M,D, L]** Notes:

    @ Target Range: 2.262

36. [O,T] A6H Load and execute the script **466TAMBL06**, which captures Z-stacks of 16 focus distances (from 1 meter to infinity) for filter 0 with seven focal lengths. The estimated duration is 10 minutes. 14:58
37. [O,T] A6H Load and execute the script **466TAMBR06**, which captures Z-stacks of 16 focus distances (from 1 meter to infinity) for filter 0 with seven focal lengths. The estimated duration is 10 minutes. 14:55
38. [D] A6H Record image names and parameters in Image Log.
39. [D, L] Notes: SCENE 14

40. [O,T] A6H Load and execute the script **466TAMBL09**, which captures Z-stacks of 16 focus distances (from 1 meter to infinity) for each non-solar filter with the **34mm** focal length. The estimated duration is 10 minutes.
41. [O,T] A6H Load and execute the script **466TAMBR09**, which captures Z-stacks of 16 focus distances (from 1 meter to infinity) for each non-solar filter with the **34mm** focal length. The estimated duration is 10 minutes.
42. [D] A6H Record image names and parameters in Image Log.
43. [D, L] Notes: SCENE 14

Data Validation

44. **[V]** NH Run the "Geometric_46_Validation" Jupyter notebook on the acquired data for the Right and Left Mastcam-Zs. This analysis can take place while the test continues.
45. **[V,D, L]** Notes: ______

Date 5/4 Time — Initials [illegible]

Fixed Target Positions for the 63mm Right and Left Mastcam-Z (Scene 15)

46. **[T]** A6H Position the JR dot target approximately ~~3~~ 4 **meters** from the camera and finely adjust it to maximize the dots visible in both Mastcam-Z.
47. **[D]** A6H Record the following temperatures:
    - Left Mastcam-Z CCD temp 24.5
    - Right Mastcam-Z CCD temp 25.0
48. **[D,T]** A6H Take digital pictures of the geometric target's position, and the whole test/GSE set-up.
49. [O,T] A6H Capture test frames to finely position the target centered in the 63mm FOV of both cameras. Save these test frames with the prefix name **466TAMBL00** and **466TAMBR00**.
50. [M] A6H Measure the locations of the geometric target and the camera.
51. [M,D] A6H Record the location measurements in the Image Log and tables below.
52. [M,D, L] Notes: Range to Target: 4.096

53. [O,T] AGH Load and execute the script **466TAMBL06**, which captures Z-stacks of 16 focus distances (from 1 meter to infinity) for filter 0 with seven focal lengths. The estimated duration is ~~10~~ 15 minutes.
54. [O,T] AGH Load and execute the script **466TAMBR06**, which captures Z-stacks of 16 focus distances (from 1 meter to infinity) for filter 0 with seven focal lengths. The estimated duration is ~~10~~ 15 minutes.
55. [D] [illegible] Record image names and parameters in Image Log.
56. [D, L] Notes: SCENE 15, 4m

57. [O,T] [illegible] Load and execute the script **466TAMBL10**, which captures Z-stacks of 16 focus distances (from 1 meter to infinity) for each non-solar filter with the **63mm** focal length. The estimated duration is 15 minutes.
58. [O,T] [illegible] Load and execute the script **466TAMBR10**, which captures Z-stacks of 16 focus distances (from 1 meter to infinity) for each non-solar filter with the **63mm** focal length. The estimated duration is 15 minutes.
59. [D] [illegible] Record image names and parameters in Image Log.
60. [D, L] Notes: 4m

redundant

61. **[O,T]** ____ Load and execute the script **466TAMBL10**, which captures Z-stacks of 16 focus distances (from 1 meter to infinity) for each non-solar filter with the **63mm** focal length. The estimated duration is 15 minutes.
62. **[O,T]** ____ Load and execute the script **466TAMBR10**, which captures Z-stacks of 16 focus distances (from 1 meter to infinity) for each non-solar filter with the **63mm** focal length. The estimated duration is 15 minutes.

63. **[O,T]** [illegible] If time permits, load and execute the script **467TAMBL01**, which auto-exposes and captures frames for ~140 focal lengths with filter 0 at a focus distance of ~~3~~ 4 meters. The estimated duration is 20 minutes.
64. **[O,T]** [illegible] If time permits, load and execute the script **467TAMBR01**, which auto-exposes and captures frames for ~140 focal lengths with filter 0 at a focus distance of ~~3~~ 4 meters. The estimated duration is 20 minutes.
65. **[D]** [illegible] Record image names and parameters in Image Log.
66. **[D, L]** Notes: SCENE 15

0 – 130

67. [O,T] [illegible] Load and execute the script **466TAMBL07**, which captures one frame at 3 meters focus at seven focal lengths in reverse order. The estimated duration is 4 minutes.
68. [O,T] [illegible] Load and execute the script **466TAMBR07**, which captures one frame at 3 meters focus at seven focal lengths in reverse order. The estimated duration is 4 minutes.
69. [D] [illegible] Record image names and parameters in Image Log.
70. [D, L] Notes: 7 → 13
    L = 24.7°C R = 25.1°C

Data Validation

71. [V] [illegible] Run the "Geometric_46_Validation" Jupyter notebook on the acquired data for the Right and Left Mastcam-Zs. This analysis can take place while the test continues.
72. [V,D, L] Notes: GOOD.

Date 5/4 Time 16:56 Initials Zn

Fixed Target Positions for the 100mm Right and Left Mastcam-Z (Scene 16)

73. [T] Zn Position the JR dot target approximately ~~5~~ 6 **meters** from the camera and finely adjust it to maximize the dots visible in both Mastcam-Z.
74. **[D]** Zn Record the following temperatures:
    - Left Mastcam-Z CCD temp 24.5°C
    - Right Mastcam-Z CCD temp 24.8°C
75. **[D,T]** Zn Take digital pictures of the geometric target's position, and the whole test/GSE set-up. IMAGE IDS: 1-3
76. **[O,T]** Zn Capture test frames to finely position the target centered in the ~~11~~ 100mm FOV of both cameras. Save these test frames with the prefix name **466TAMBL00** and **466TAMBR00**.
77. **[M]** Zn Measure the locations of the geometric target and the camera. ~~6.207m~~ 6.211 m
78. **[M,D]** Zn Record the location measurements in the Image Log and tables below.
79. **[M,D, L]** Notes: PROBLEM WITH RIGHT CAMERA FOCUS & COMMANDING. POWERED OFF RIGHT CAMERA, EXITED GUI AND RESTARTED IT. POWERED CAMERA ON, PROBLEM SOLVED, RESUMED STEP 76. RESET OUTPUT FILE, MOVED TO PROPER DIRECTORY

80. [O,T] [illegible] Load and execute the script **466TAMBL06**, which captures Z-stacks of 16 focus distances (from 1 meter to infinity) for filter 0 with seven focal lengths. The estimated duration is 10 minutes. 26, 34, 48, 63, 79, 100, 110
81. [O,T] [illegible] Load and execute the script **466TAMBR06**, which captures Z-stacks of 16 focus distances (from 1 meter to infinity) for filter 0 with seven focal lengths. The estimated duration is 10 minutes.
82. **[D]** [illegible] Record image names and parameters in Image Log.
83. **[D, L]** Notes: 476 – 594 L = 24.7°C
    6m SCENE 16 R = 25.1°C

84. [O,T] [illegible] Load and execute the script **466TAMBL11**, which captures Z-stacks of 16 focus distances (from 1 meter to infinity) for each non-solar filter with the **100mm** focal length. The estimated duration is 15 minutes.
85. [O,T] [illegible] Load and execute the script **466TAMBR11**, which captures Z-stacks of 16 focus distances (from 1 meter to infinity) for each non-solar filter with the **100mm** focal length. The estimated duration is 15 minutes.
86. **[D]** [illegible] Record image names and parameters in Image Log.
87. **[D, L]** Notes: 0-118

Data Validation

88. **[V]** [illegible] Run the "Geometric_46_Validation" Jupyter notebook on the acquired data for the Right and Left Mastcam-Zs. This analysis can take place while the test continues.
89. **[V,D, L]** Notes: GOOD.

Fixed Target Positions for the 26mm Right and Left Mastcam-Z (Scene 17)

90. **[T]** [illegible] Position the JR dot target approximately **1.5 meters** from the camera and finely adjust it to maximize the dots visible in both Mastcam-Z. 1.79m
91. **[D]** [illegible] Record the following temperatures:
    - Left Mastcam-Z CCD temp 24.2°C LØ
    - Right Mastcam-Z CCD temp 24.6°C RØ
92. **[D,T]** [illegible] Take digital pictures of the geometric target's position, and the whole test/GSE set-up. 4-6 IMAGES
93. **[O,T]** [illegible] Capture test frames to finely position the target centered in the ~~34~~ 26mm FOV of both cameras. Save these test frames with the prefix name **466TAMBL00** and **466TAMBR00**.
94. **[M]** [illegible] Measure the locations of the geometric target and the camera. ~~1.575m~~ 1.508m
95. **[M,D]** [illegible] Record the location measurements in the Image Log and tables below.
96. **[M,D, L]** Notes: TURNED OFF QUARTZ HALOGEN AND LED LIGHTS.

97. [O,T] Zm Load and execute the script **466TAMBL06**, which captures Z-stacks of 16 focus distances (from 1 meter to infinity) for filter 0 with seven focal lengths. The estimated duration is 10 minutes.
98. [O,T] Zm Load and execute the script **466TAMBR06**, which captures Z-stacks of 16 focus distances (from 1 meter to infinity) for filter 0 with seven focal lengths. The estimated duration is 10 minutes.
99. **[D]** ____ Record image names and parameters in Image Log.
100. **[D, L]** Notes: → 713

SKIP

101. [O,T] ____ Load and execute the script **466TAMBL08**, which captures Z-stacks of 16 focus distances (from 1 meter to infinity) for each non-solar filter with the **26mm** focal length. The estimated duration is 15 minutes.
102. [O,T] ____ Load and execute the script **466TAMBR08**, which captures Z-stacks of 16 focus distances (from 1 meter to infinity) for each non-solar filter with the **26mm** focal length. The estimated duration is 15 minutes.
103. **[D]** ____ Record image names and parameters in Image Log.
104. **[D, L]** Notes: SKIPPED BECAUSE SCRIPT IS NOT CONSISTENT WITH PROCEDURE.

Data Validation

105. **[V]** Zm Run the "Geometric_46_Validation" Jupyter notebook on the acquired data for the Right and Left Mastcam-Zs. This analysis can take place while the test continues.
106. **[V,D, L]** Notes: GOOD.

Explanation of the Semi-Random Orientations

Figure 2. An example of the camera's FOV (black) and JR dot target's semi-random positions (red)

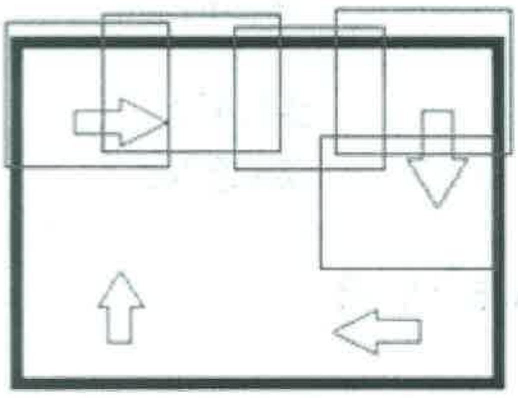

Figure 2 shows the desired orientations for the following tests that ask for a certain number of "semi-random orientations" of the JR dot target. The blue arrows show the motion of the target across the camera's FOV for optimal coverage. Note that some frames should be taken with the JR dot target rotated 90-degrees around the camera's line-of-sight.

100+ Target Positions for the 34mm Mastcam-Zs (Scene 18)

107. **[T]** [illegible] Position the JR dot target approximately **3 meters** from the camera. Adjust lights accordingly. 3.014 m 34mm
108. **[D]** [illegible] Record the following temperatures:
    - Left Mastcam-Z CCD temp 24.4°C
    - Right Mastcam-Z CCD temp 24.8°C
109. **[D,T]** [illegible] Take digital pictures of the geometric target's position, and the whole test/GSE set-up. 105 = 7-9

110. **[O,T]** [illegible] Capture test frames to find a standard exposure time for the 100 positions at 3 meters focus. Save these test frames with the prefix name **465TAMBL00**, and update "var1" in the script **465TAMBL02** once this exposure time is found. 161 ms
111. **[V,O,T]** [illegible] Evaluate whether the target's dots are in-focus enough for discrimination. If the dots are too out-of-focus for JR's algorithm, move the target back. Also watch out for high levels of saturated pixels.

112. **[O,T]** [illegible] Capture test frames to find a standard exposure time for the 100 positions at 3 meters focus. Save these test frames with the prefix name **465TAMBR00**, and update "var1" in the script **465TAMBR02** once this exposure time is found. 165ms
113. **[V,O,T]** [illegible] Evaluate whether the target's dots are in-focus enough for discrimination. If the dots are too out-of-focus for JR's algorithm, move the target back. Also watch out for high levels of saturated pixels.

114. **[O,T]** [illegible] Load and begin the script **465TAMBL02**, which captures frames with filter 0 at **34mm** focal length one frame at a time, with a pause command between each frame.
115. **[O,T]** [illegible] Load and begin the script **465TAMBR02**, which captures frames with filter 0 at **34mm** focal length one frame at a time, with a pause command between each frame.

FIRST RUN ABORTED
2nd " "
LEFT SCRIPT USED TO SYNC SUFFICES YZ

 Date 5/4/19 Time 20:14 Initials [illegible]

CHANGED TARGET SUPPORT EQUIPMENT TO BETTER ALLOW ROTATION.

116. [O,T] [illegible] Capture **10 images** of the JR dot target in semi-random orientations (see Figure 2) normal to the camera approximately **1-meter** distance covering each edge of the camera's FOV. ~~1.07m~~ 7 1.093m

117. [O,T] [illegible] Capture **20 images** of the JR dot target in semi-random orientations (see Figure 2) normal to the camera approximately **2-meter** distance covering each edge of the camera's FOV. Adjust lighting if necessary, to keep the frames at approximately 50% full-well. 1.993m L: UL, LL, R: UR, LR

118. [O,T] [illegible] Capture **30 images** of the JR dot target in semi-random orientations (see Figure 2) normal to the camera approximately **3-meter** distance covering each edge of the camera's FOV. Adjust lighting if necessary, to keep the frames at approximately 50% full-well. 3.20 m

SLIGHTLY MORE TO AVOID FLOOR PANELS

119. [O,T] [illegible] Capture **20 images** of the JR dot target in semi-random orientations (see Figure 2) normal to the camera approximately **4-meter** distance covering each edge of the camera's FOV. Adjust lighting if necessary, to keep the frames at approximately 50% full-well. 4.20 m

120. [O,T] [illegible] Capture **20 images** of the JR dot target in semi-random orientations (see Figure 2) normal to the camera approximately **5-meter** distance covering each edge of the camera's FOV. Adjust lighting if necessary, to keep the frames at approximately 50% full-well. 5.20

COULD NOT MOVE TARGET TO LEFT SIDE OF IMAGES

121. [O,T] [illegible] Capture **10 images** of the JR dot target in semi-random orientations (see Figure 2) normal to the camera approximately **7-meter** distance covering each edge of the camera's FOV. Adjust lighting if necessary, to keep the frames at approximately 50% full-well. 7.05m

HUMIDITY = 51%

AGAIN, INCOMPLETE COVERAGE

Date 5/4 Time 23:15 Initials [illegible]

✓122. [V,O,T] Evaluate whether the target's dots are evenly distributed over each fields of view. Also watch out for high levels of saturated pixels.

123. [O,T, L] [illegible] After more than 100 usable frames have been captured, stop the prefix script.

124. [D] [illegible] Record image names and parameters in Image Log.

125. [D, L] Notes: HUMIDITY = 67%

DONE w/ SCENE 18

Data Validation

126. [V] [illegible] Run the "Geometric_46_Validation" Jupyter notebook on the acquired data for for the Right and Left Mastcam-Zs. This analysis can take place while the test continues.

127. [V,D, L] Notes: GOOD.

 Date 5/4 Time 23:16 Initials [illegible]

100+ Target Positions for the 48mm Right and Left Mastcam-Zs (Scene 19)

128. **[T]** [illegible] Position the JR dot target approximately **3 meters** from the camera. Adjust lights accordingly.
129. **[D]** [illegible] Record the following temperatures:
    - Left Mastcam-Z CCD temp 24.3°C
    - Right Mastcam-Z CCD temp 24.0°C
130. **[D,T]** ____ Take digital pictures of the geometric target's position, and the whole test/GSE set-up. IMAGE IDS =

Skip

131. **[O,T]** ____ Capture test frames to find a standard exposure time for the 100 positions at 3 meters focus. Save these test frames with the prefix name **465TAMBL00**, and update "var1" in the script **465TAMBL03** once this exposure time is found.
132. **[V,O,T]** ____ Evaluate whether the target's dots are in-focus enough for discrimination. If the dots are too out-of-focus for JR's algorithm, move the target back. Also watch out for high levels of saturated pixels.

133. **[O,T]** ____ Capture test frames to find a standard exposure time for the 100 positions at 3 meters focus. Save these test frames with the prefix name **465TAMBR00**, and update "var1" in the script **465TAMBR03** once this exposure time is found.
134. **[V,O,T]** ____ Evaluate whether the target's dots are in-focus enough for discrimination. If the dots are too out-of-focus for JR's algorithm, move the target back. Also watch out for high levels of saturated pixels.

135. **[O,T]** ____ Load and begin the script **465TAMBL03**, which captures frames with filter 0 at **48mm** focal length one frame at a time, with a pause command between each frame.
136. **[O,T]** ____ Load and begin the script **465TAMBR03**, which captures frames with filter 0 at **48mm** focal length one frame at a time, with a pause command between each frame.

Skip

137. **[O,T]** ____ Capture **10 images** of the JR dot target in semi-random orientations (see Figure 2) normal to the camera approximately **1-meter** distance covering each edge of the camera's FOV.
138. **[O,T]** ____ Capture **20 images** of the JR dot target in semi-random orientations (see Figure 2) normal to the camera approximately **2-meter** distance covering each edge of the camera's FOV. Adjust lighting if necessary, to keep the frames at approximately 50% full-well.
139. **[O,T]** ____ Capture **20 images** of the JR dot target in semi-random orientations (see Figure 2) normal to the camera approximately **3-meter** distance covering each edge of the camera's FOV. Adjust lighting if necessary, to keep the frames at approximately 50% full-well.
140. **[O,T]** ____ Capture **20 images** of the JR dot target in semi-random orientations (see Figure 2) normal to the camera approximately **4-meter** distance covering each edge of the camera's FOV. Adjust lighting if necessary, to keep the frames at approximately 50% full-well.
141. **[O,T]** ____ Capture **20 images** of the JR dot target in semi-random orientations (see Figure 2) normal to the camera approximately **5-meter** distance covering each edge of the camera's FOV. Adjust lighting if necessary, to keep the frames at approximately 50% full-well.
142. **[O,T]** ____ Capture **10 images** of the JR dot target in semi-random orientations (see Figure 2) normal to the camera approximately **7-meter** distance covering each edge of the camera's FOV. Adjust lighting if necessary, to keep the frames at approximately 50% full-well.

Skip

143. [V,O,T] ____ Evaluate whether the target's dots are evenly distributed over each fields of view. Also watch out for high levels of saturated pixels.
144. [O,T, L] ____ After more than 100 usable frames have been captured, stop the prefix script.
145. [D] ____ Record image names and parameters in Image Log.
146. [D, L] Notes:____
____
____

Data Validation

147. [V] ____ Run the "Geometric_46_Validation" Jupyter notebook on the acquired data for for the Right and Left Mastcam-Zs. This analysis can take place while the test continues.
148. [V,D, L] Notes: ______________________________________
____
____

100+ Target Positions for the 100mm Right and Left Mastcam-Zs (Scene 20)

skip

149. **[T]** ____ Position the JR dot target approximately **3 meters** from the camera. Adjust lights accordingly.
150. **[D]** ____ Record the following temperatures:
    - Left Mastcam-Z CCD temp ______________
    - Right Mastcam-Z CCD temp ______________
151. **[D,T]** ____ Take digital pictures of the geometric target's position, and the whole test/GSE set-up.

152. **[O,T]** ____ Capture test frames to find a standard exposure time for the 100 positions at 3 meters focus. Save these test frames with the prefix name **465TAMBL00**, and update "var1" in the script **465TAMBL06** once this exposure time is found.
153. **[V,O,T]** ____ Evaluate whether the target's dots are in-focus enough for discrimination. If the dots are too out-of-focus for JR's algorithm, move the target back. Also watch out for high levels of saturated pixels.

154. **[O,T]** ____ Capture test frames to find a standard exposure time for the 100 positions at 3 meters focus. Save these test frames with the prefix name **465TAMBR00**, and update "var1" in the script **465TAMBR06** once this exposure time is found.
155. **[V,O,T]** ____ Evaluate whether the target's dots are in-focus enough for discrimination. If the dots are too out-of-focus for JR's algorithm, move the target back. Also watch out for high levels of saturated pixels.

156. **[O,T]** ____ Load and begin the script **465TAMBL06**, which captures frames with filter 0 at **100mm** focal length one frame at a time, with a pause command between each frame.
157. **[O,T]** ____ Load and begin the script **465TAMBR06**, which captures frames with filter 0 at **100mm** focal length one frame at a time, with a pause command between each frame.

Skip

158. **[O,T]** ____ Capture **10 images** of the JR dot target in semi-random orientations (see Figure 2) normal to the camera approximately **2-meter** distance covering each edge of the camera's FOV.
159. **[O,T]** ____ Capture **10 images** of the JR dot target in semi-random orientations (see Figure 2) normal to the camera approximately **3-meter** distance covering each edge of the camera's FOV. Adjust lighting if necessary, to keep the frames at approximately 50% full-well.
160. **[O,T]** ____ Capture **20 images** of the JR dot target in semi-random orientations (see Figure 2) normal to the camera approximately **4-meter** distance covering each edge of the camera's FOV. Adjust lighting if necessary, to keep the frames at approximately 50% full-well.
161. **[O,T]** ____ Capture **20 images** of the JR dot target in semi-random orientations (see Figure 2) normal to the camera approximately **5-meter** distance covering each edge of the camera's FOV. Adjust lighting if necessary, to keep the frames at approximately 50% full-well.
162. **[O,T]** ____ Capture **20 images** of the JR dot target in semi-random orientations (see Figure 2) normal to the camera approximately **6-meter** distance covering each edge of the camera's FOV. Adjust lighting if necessary, to keep the frames at approximately 50% full-well.
163. **[O,T]** ____ Capture **20 images** of the JR dot target in semi-random orientations (see Figure 2) normal to the camera approximately **8-meter** distance covering each edge of the camera's FOV. Adjust lighting if necessary, to keep the frames at approximately 50% full-well.

skip

Date ______ Time ______ Initials ______

164. [V,O,T] ____ Evaluate whether the target's dots are evenly distributed over each fields of view. Also watch out for high levels of saturated pixels.
165. [O,T, L] ____ After more than 100 usable frames have been captured, stop the prefix script.
166. [D] ____ Record image names and parameters in Image Log.
167. [D, L] Notes:____
____
____

Data Validation

168. [V] ____ Run the "Geometric_46_Validation" Jupyter notebook on the acquired data for for the Right and Left Mastcam-Zs. This analysis can take place while the test continues.
169. [V,D, L] Notes: ____
____
____

100+ Target Positions for the 26mm Right and Left Mastcam-Zs (Scene 21) Skip

170. **[T]** ____ Position the JR dot target approximately **2 meters** from the camera. Adjust lights accordingly.
171. **[D]** ____ Record the following temperatures:
    - Left Mastcam-Z CCD temp ______________
    - Right Mastcam-Z CCD temp ______________
172. **[D,T]** ____ Take digital pictures of the geometric target's position, and the whole test/GSE set-up.

173. **[O,T]** ____ Capture test frames to find a standard exposure time for the 100 positions at 3 meters focus. Save these test frames with the prefix name **465TAMBL00**, and update "var1" in the script **465TAMBL01** once this exposure time is found.
174. **[V,O,T]** ____ Evaluate whether the target's dots are in-focus enough for discrimination. If the dots are too out-of-focus for JR's algorithm, move the target back. Also watch out for high levels of saturated pixels.

175. **[O,T]** ____ Capture test frames to find a standard exposure time for the 100 positions at 3 meters focus. Save these test frames with the prefix name **465TAMBR00**, and update "var1" in the script **465TAMBR01** once this exposure time is found.
176. **[V,O,T]** ____ Evaluate whether the target's dots are in-focus enough for discrimination. If the dots are too out-of-focus for JR's algorithm, move the target back. Also watch out for high levels of saturated pixels.

177. **[O,T]** ____ Load and begin the script **465TAMBL01**, which captures frames with filter 0 at **26mm** focal length one frame at a time, with a pause command between each frame.
178. **[O,T]** ____ Load and begin the script **465TAMBR01**, which captures frames with filter 0 at **26mm** focal length one frame at a time, with a pause command between each frame.

Skip

179. **[O,T]** ____ Capture **20 images** of the JR dot target in semi-random orientations (see Figure 2) normal to the camera approximately **1-meter** distance covering each edge of the camera's FOV.
180. **[O,T]** ____ Capture **20 images** of the JR dot target in semi-random orientations (see Figure 2) normal to the camera approximately **2-meter** distance covering each edge of the camera's FOV. Adjust lighting if necessary, to keep the frames at approximately 50% full-well.
181. **[O,T]** ____ Capture **20 images** of the JR dot target in semi-random orientations (see Figure 2) normal to the camera approximately **3-meter** distance covering each edge of the camera's FOV. Adjust lighting if necessary, to keep the frames at approximately 50% full-well.
182. **[O,T]** ____ Capture **20 images** of the JR dot target in semi-random orientations (see Figure 2) normal to the camera approximately **4-meter** distance covering each edge of the camera's FOV. Adjust lighting if necessary, to keep the frames at approximately 50% full-well.
183. **[O,T]** ____ Capture **10 images** of the JR dot target in semi-random orientations (see Figure 2) normal to the camera approximately **5-meter** distance covering each edge of the camera's FOV. Adjust lighting if necessary, to keep the frames at approximately 50% full-well.
184. **[O,T]** ____ Capture **10 images** of the JR dot target in semi-random orientations (see Figure 2) normal to the camera approximately **7-meter** distance covering each edge of the camera's FOV. Adjust lighting if necessary, to keep the frames at approximately 50% full-well.

skip

185. **[V,O,T]** ____ Evaluate whether the target's dots are evenly distributed over each fields of view. Also watch out for high levels of saturated pixels.
186. **[O,T, L]** ____ After more than 100 usable frames have been captured, stop the prefix script.
187. **[D]** ____ Record image names and parameters in Image Log.
188. **[D, L]** Notes:____

____

____

Data Validation

189. **[V]** ____ Run the "Geometric_46_Validation" Jupyter notebook on the acquired data for for the Right and Left Mastcam-Zs. This analysis can take place while the test continues.
190. **[V,D, L]** Notes:____

____

____

100+ Target Positions for the 63mm Right and Left Mastcam-Zs (Scene 22)

191. **[T]** [illegible] Position the JR dot target approximately **3 meters** from the camera. Adjust lights accordingly. 3.35m
192. **[D]** [illegible] Record the following temperatures:
    - Left Mastcam-Z CCD temp ~~25.0°C~~ 24.4°C
    - Right Mastcam-Z CCD temp 25.0°C
193. **[D,T]** [illegible] Take digital pictures of the geometric target's position, and the whole test/GSE set-up. IMAGE IDS =

194. **[O,T]** [illegible] Capture test frames to find a standard exposure time for the 100 positions at 3 meters focus. Save these test frames with the prefix name **465TAMBL00**, and update "var1" in the script **465TAMBL04** once this exposure time is found. 205ms
195. **[V,O,T]** [illegible] Evaluate whether the target's dots are in-focus enough for discrimination. If the dots are too out-of-focus for JR's algorithm, move the target back. Also watch out for high levels of saturated pixels.

196. **[O,T]** [illegible] Capture test frames to find a standard exposure time for the 100 positions at 3 meters focus. Save these test frames with the prefix name **465TAMBR00**, and update "var1" in the script **465TAMBR04** once this exposure time is found.
197. **[V,O,T]** [illegible] Evaluate whether the target's dots are in-focus enough for discrimination. If the dots are too out-of-focus for JR's algorithm, move the target back. Also watch out for high levels of saturated pixels.

198. **[O,T]** [illegible] Load and begin the script **465TAMBL04**, which captures frames with filter 0 at **63mm** focal length one frame at a time, with a pause command between each frame.
199. **[O,T]** [illegible] Load and begin the script **465TAMBR04**, which captures frames with filter 0 at63 focal length one frame at a time, with a pause command between each frame.

200. [O,T] ____ Capture **10 images** of the JR dot target in semi-random orientations (see Figure 2) normal to the camera approximately **1-meter** distance covering each edge of the camera's FOV. ~~1.15m~~ 1.10m RESTARTED GUI, ABORTED.

201. [O,T] ____ Capture **20 images** of the JR dot target in semi-random orientations (see Figure 2) normal to the camera approximately **2-meter** distance covering each edge of the camera's FOV. Adjust lighting if necessary, to keep the frames at approximately 50% full-well.

202. [O,T] ____ Capture **20 images** of the JR dot target in semi-random orientations (see Figure 2) normal to the camera approximately **3-meter** distance covering each edge of the camera's FOV. Adjust lighting if necessary, to keep the frames at approximately 50% full-well.

203. [O,T] ____ Capture **20 images** of the JR dot target in semi-random orientations (see Figure 2) normal to the camera approximately **4-meter** distance covering each edge of the camera's FOV. Adjust lighting if necessary, to keep the frames at approximately 50% full-well.

204. [O,T] ____ Capture **20 images** of the JR dot target in semi-random orientations (see Figure 2) normal to the camera approximately **5-meter** distance covering each edge of the camera's FOV. Adjust lighting if necessary, to keep the frames at approximately 50% full-well.

205. [O,T] ____ Capture **10 images** of the JR dot target in semi-random orientations (see Figure 2) normal to the camera approximately **7-meter** distance covering each edge of the camera's FOV. Adjust lighting if necessary, to keep the frames at approximately 50% full-well.

Date ______ Time ______ Initials ______

Skip

206. **[V,O,T]** ____ Evaluate whether the target's dots are evenly distributed over each fields of view. Also watch out for high levels of saturated pixels.
207. **[O,T, L]** ____ After more than 100 usable frames have been captured, stop the prefix script.
208. **[D]** ____ Record image names and parameters in Image Log.
209. **[D, L]** Notes:____

____

____

Data Validation

210. **[V]** ____ Run the "Geometric_46_Validation" Jupyter notebook on the acquired data for for the Right and Left Mastcam-Zs. This analysis can take place while the test continues.
211. **[V,D, L]** Notes: ____

____

____

100+ Target Positions for the 110mm Right and Left Mastcam-Zs (Scene 23)

Skip

212. **[T]** ____ Position the JR dot target approximately **3 meters** from the camera. Adjust lights accordingly.
213. **[D]** ____ Record the following temperatures:
    - Left Mastcam-Z CCD temp ______________
    - Right Mastcam-Z CCD temp ______________
214. **[D,T]** ____ Take digital pictures of the geometric target's position, and the whole test/GSE set-up.

215. **[O,T]** ____ Capture test frames to find a standard exposure time for the 100 positions at 3 meters focus. Save these test frames with the prefix name **465TAMBL00**, and update "var1" in the script **465TAMBL07** once this exposure time is found.
216. **[V,O,T]** ____ Evaluate whether the target's dots are in-focus enough for discrimination. If the dots are too out-of-focus for JR's algorithm, move the target back. Also watch out for high levels of saturated pixels.

217. **[O,T]** ____ Capture test frames to find a standard exposure time for the 100 positions at 3 meters focus. Save these test frames with the prefix name **465TAMBR00**, and update "var1" in the script **465TAMBR07** once this exposure time is found.
218. **[V,O,T]** ____ Evaluate whether the target's dots are in-focus enough for discrimination. If the dots are too out-of-focus for JR's algorithm, move the target back. Also watch out for high levels of saturated pixels.

219. **[O,T]** ____ Load and begin the script **465TAMBL07**, which captures frames with filter 0 at **110mm** focal length one frame at a time, with a pause command between each frame.
220. **[O,T]** ____ Load and begin the script **465TAMBR07**, which captures frames with filter 0 at **110mm** focal length one frame at a time, with a pause command between each frame.

Skip

221. **[O,T]** ____ Capture **10 images** of the JR dot target in semi-random orientations (see Figure 2) normal to the camera approximately **2-meter** distance covering each edge of the camera's FOV.
222. **[O,T]** ____ Capture **10 images** of the JR dot target in semi-random orientations (see Figure 2) normal to the camera approximately **3-meter** distance covering each edge of the camera's FOV. Adjust lighting if necessary, to keep the frames at approximately 50% full-well.
223. **[O,T]** ____ Capture **20 images** of the JR dot target in semi-random orientations (see Figure 2) normal to the camera approximately **4-meter** distance covering each edge of the camera's FOV. Adjust lighting if necessary, to keep the frames at approximately 50% full-well.
224. **[O,T]** ____ Capture **20 images** of the JR dot target in semi-random orientations (see Figure 2) normal to the camera approximately **5-meter** distance covering each edge of the camera's FOV. Adjust lighting if necessary, to keep the frames at approximately 50% full-well.
225. **[V,O,T]** ____ Evaluate whether the target's dots are in-focus enough for discrimination. If the dots are too out-of-focus for JR's algorithm, move the target back. Also watch out for high levels of saturated pixels.
226. **[V, L]** ____ If the dots are indistinguishable, consider terminating the script and skip to the end of the section.

227. **[O,T]** ____ Capture **20 images** of the JR dot target in semi-random orientations (see Figure 2) normal to the camera approximately **7-meter** distance covering each edge of the camera's FOV. Adjust lighting if necessary, to keep the frames at approximately 50% full-well.
228. **[O,T]** ____ Capture **20 images** of the JR dot target in semi-random orientations (see Figure 2) normal to the camera approximately **8-meter** distance covering each edge of

Skip

the camera's FOV. Adjust lighting if necessary, to keep the frames at approximately 50% full-well.

229. **[V,O,T]** ____ Evaluate whether the target's dots are evenly distributed over each fields of view. Also watch out for high levels of saturated pixels.
230. **[O,T, L]** ____ After more than 100 usable frames have been captured, stop the prefix script.
231. **[D]** ____ Record image names and parameters in Image Log.
232. **[D, L]** Notes:____

____

____

Data Validation

233. **[V]** ____ Run the "Geometric_46_Validation" Jupyter notebook on the acquired data for for the Right and Left Mastcam-Zs. This analysis can take place while the test continues.
234. **[V,D, L]** Notes: ____

____

____

100+ Target Positions for the 78mm Right and Left Mastcam-Zs (Scene 24) Skip

235. **[T]** ____ Position the JR dot target approximately **3 meters** from the camera. Adjust lights accordingly.

236. **[D]** ____ Record the following temperatures:
    - Left Mastcam-Z CCD temp ______________
    - Right Mastcam-Z CCD temp ______________

237. **[D,T]** ____ Take digital pictures of the geometric target's position, and the whole test/GSE set-up.

238. **[O,T]** ____ Capture test frames to find a standard exposure time for the 100 positions at 3 meters focus. Save these test frames with the prefix name **465TAMBL00**, and update "var1" in the script **465TAMBL05** once this exposure time is found.

239. **[V,O,T]** ____ Evaluate whether the target's dots are in-focus enough for discrimination. If the dots are too out-of-focus for JR's algorithm, move the target back. Also watch out for high levels of saturated pixels.

240. **[O,T]** ____ Capture test frames to find a standard exposure time for the 100 positions at 3 meters focus. Save these test frames with the prefix name **465TAMBR00**, and update "var1" in the script **465TAMBR05** once this exposure time is found.

241. **[V,O,T]** ____ Evaluate whether the target's dots are in-focus enough for discrimination. If the dots are too out-of-focus for JR's algorithm, move the target back. Also watch out for high levels of saturated pixels.

242. **[O,T]** ____ Load and begin the script **465TAMBL05**, which captures frames with filter 0 at **78mm** focal length one frame at a time, with a pause command between each frame.

243. **[O,T]** ____ Load and begin the script **465TAMBR05**, which captures frames with filter 0 at **78mm** focal length one frame at a time, with a pause command between each frame.

Skip

244. **[O,T]** ____ Capture **10 images** of the JR dot target in semi-random orientations (see Figure 2) normal to the camera approximately **2-meter** distance covering each edge of the camera's FOV.
245. **[O,T]** ____ Capture **10 images** of the JR dot target in semi-random orientations (see Figure 2) normal to the camera approximately **3-meter** distance covering each edge of the camera's FOV. Adjust lighting if necessary, to keep the frames at approximately 50% full-well.
246. **[O,T]** ____ Capture **20 images** of the JR dot target in semi-random orientations (see Figure 2) normal to the camera approximately **4-meter** distance covering each edge of the camera's FOV. Adjust lighting if necessary, to keep the frames at approximately 50% full-well.
247. **[O,T]** ____ Capture **20 images** of the JR dot target in semi-random orientations (see Figure 2) normal to the camera approximately **5-meter** distance covering each edge of the camera's FOV. Adjust lighting if necessary, to keep the frames at approximately 50% full-well.
248. **[V,O,T]** ____ Evaluate whether the target's dots are in-focus enough for discrimination. If the dots are too out-of-focus for JR's algorithm, move the target back. Also watch out for high levels of saturated pixels.
249. **[V, L]** ____ If the dots are indistinguishable, consider terminating the script and skip to the end of the section.

250. **[O,T]** ____ Capture **20 images** of the JR dot target in semi-random orientations (see Figure 2) normal to the camera approximately **7-meter** distance covering each edge of the camera's FOV. Adjust lighting if necessary, to keep the frames at approximately 50% full-well.
251. **[O,T]** ____ Capture **20 images** of the JR dot target in semi-random orientations (see Figure 2) normal to the camera approximately **8-meter** distance covering each edge of

Skip

the camera's FOV. Adjust lighting if necessary, to keep the frames at approximately 50% full-well.

252. **[V,O,T]** ____ Evaluate whether the target's dots are evenly distributed over each fields of view. Also watch out for high levels of saturated pixels.
253. **[O,T, L]** ____ After more than 100 usable frames have been captured, stop the prefix script.
254. **[D]** ____ Record image names and parameters in Image Log.
255. **[D, L]** Notes:____

Data Validation

256. **[V]** ____ Run the "Geometric_46_Validation" Jupyter notebook on the acquired data for for the Right and Left Mastcam-Zs. This analysis can take place while the test continues.
257. **[V,D, L]** Notes: ____

Shutdown Procedure

Skip

258. [D,T] ____ Take digital pictures of the test setup.
259. [D,O] ____ Review entries in Image Log, GSE command log, and image headers. Procedure not completed
260. [D, L] ____ Review calibration procedure and ensure that each task is initialed.
261. [D, L] Notes: ____

262. [V, L] ____ Before making the decision to break down the test setup, ensure that adequate data were acquired for the test requirements. See "MastcamZCalPlan" for these requirements.
263. [V] Notes: ____

Data Validator (signature) ______________________

Date ____________ Time ____________

264. [V, L] ____ Give the go/no-go decision. Have enough data been acquired to fulfill test requirements? See "MastcamZCalPlan" for these requirements.
265. [D, L] ____ Update the Log Document.
266. [L] Notes: ____

Calibration Lead (signature) x______________________ Ken

Date ____________ Time ____________

267. **[O, L]** ____ Ensure that the camera and GSE are in a safe state.
268. **[O,D]** ____ Review the Image Log with the documentarian. Exchange high-fives. Skip
269. **[O]** Notes: ____
____ Procedure not completed
____

Camera Operator (signature) ________________________

Date ______________ Time ______________

270. **[T]** ____ If the next test does not require the target, position it away from the chamber or bench. Otherwise, be sure not to move it. The next test is _________________.
271. **[T]** ____ Ensure that all other test equipment is safely put away.
272. **[T]** Notes: ____
____
____

Technician (signature) ________________________

Date ______________ Time ______________

273. **[D, L]** ____ Double-check this procedure and ensure that the top of each page has valid data, time and initials.
274. **[D]** ____ Photo-scan this document, save it on the cloud, and file the hard-copy in the Log Binder. Upload the digital pictures taken during this test in the appropriate archive on the cloud. The required links are on the Wiki.
275. **[D]** ____ Double-check that every required cell the Image Log is accurately filled. When this is complete, print the Image Log and file it the Log Binder after this document.
276. **[D]** Notes: ____
____
____

Documentarian (signature) ________________________

Date ______________ Time ______________
