# Supplementary material for: Pre-Flight Calibration of the Mars 2020 Rover Mastcam Zoom (Mastcam-Z) Multispectral, Stereoscopic Imager
Source: Space Sci Rev. 2021 Feb 18;217(2):29. doi: 10.1007/s11214-021-00795-x (PMC7892537; doi:10.1007/s11214-021-00795-x)
Supplement: Supplementary file 1 — (ZIP 98.6 MB) [file 11214_2021_795_MOESM1_ESM.zip › CalPro_465-7_JR_Geometric_v2_13.pdf]

Date 5/7 Time ~~22:20~~ 23:20 Initials CC

JR Geometric Calibration Procedure for the Right and Left Mastcam-Z Ambient Cleanroom Testing at MSSS (Pro. 4.6.5-7)

[Procedure version 2.13, prepared by the Mastcam-Z calibration team at Cornell University]

These measurements are performed on the camera and at the Temperature designated below as specified in the Mastcam-Z Calibration Plan,

Unit Under Test:

Left FM __X__ **Right FM** __X__ **EQM**____ **Other**________

These measurements are performed at Temperature:

-35°C____ **-10°C**____ **+5°C**____ **Ambient** __X__ **Other**________

These measurements are performed at,

MSSS __X__ **ASU**____ **Other**________

Date 5/7/19 **Start Time** 11:20p **End Time** 12:30 Am

Estimated Duration 4.0 hours

Scheduled Start Time 9:00pm **Sch. End Time**________

Calibration Lead [L] Jeff Johnson **Documentarian [D]** Nathalie Turenne

Camera Operator [O] Jason Van Beek **Technician [T]** Christian Tate

Data Validator [V] Ole Jensen Noel **Metrologist [M]** N/A

Other ________

Date 5/7 Time 24 Initials [illegible]

Change Log

Version	Name	Change
v1_01<br>26 Sep 2018	C. Tate	(first draft)
v1_07<br>1 Nov 2018	C. Tate	Procedure edits prior to EQM testing
v1_07-JR<br>8 Nov 2018	G. Paar	Distances more precisely reflected, change mode from v06 to v07 kept, fixed focus consistently at 2 tables & figure automatically referenced
v1_10<br>13 Dec. 2018	C. Tate	Procedure edits after EQM testing
v2_13<br>7 May 2019	G. Paar<br>C. Tate	Approved version prior to FM testing

Document Approval

Approved by James Bell Date
Mastcam-Z PI
Arizona State University

5/7/19
Approved by Alexander Hayes Date
Mastcam-Z Calibration Working Group Lead, Cornell University

Approved by Justin Maki Date
Mastcam-Z Deputy PI and Investigation Scientist, Jet Propulsion Laboratory

Approved by Ken Herkenhoff Date
Mastcam-Z Co-Investigator
USGS

5/8/19
Approved by Date
JEFFREY R. JOHNSON

5/7/19
Approved by Christian Tate Date
Procedure Author
Cornell University

Table of Contents

JR GEOMETRIC CALIBRATION PROCEDURE FOR THE RIGHT AND LEFT MASTCAM-Z AMBIENT CLEANROOM TESTING AT MSSS (PRO. 4.6.5-7) **1**

- Change Log 2
- Document Approval 2
- Test Description 4
- Software Preparation 4
  - *Table 1. File naming convention for the camera script prefixes and frame filenames: "AAABBBBCDD"* *4*
- Hardware Installation 6
  - *Figure 1. MSSS Floor Plan for Geometric Testing in the Cleanroom Chamber.* *6*
- Fixed Target Positions for the 100mm Right and Left Mastcam-Z (Scene 16) 8
- Data Validation 9
- Explanation of the Semi-Random Orientations 10
  - *Figure 2. An example of the camera's FOV (black) and JR dot target's semi-random positions (red)* *10*
- 40+ Target Positions for the 100mm Right and Left Mastcam-Zs (Scene 21) 11
- Data Validation 13
- 100+ Target Positions for the 63mm Right and Left Mastcam-Zs (Scene 22) 14
- Data Validation 16
- 100+ Target Positions for the 26mm Right and Left Mastcam-Zs (Scene 21) 17
- Data Validation 19

SHUTDOWN PROCEDURE **20**

Test Description

Excerpt from the Calibration Plan 4.6

> The objective of Geometric Calibration is to characterize the geometric distortion introduced by the Mastcam-Z optics into its images and measure the effective focal length and field of view at each focus and zoom position. As the range of zoom positions available to Mastcam-Z represent a continuum, measurements will be acquired at a finite number of zoom settings and then interpolated to characterize distortion and other geometric parameters across the full zoom range. Targets should be imaged at ~50% full well using the Bayer RGB/805 nm (priority 1) and remaining non-solar filters (priority 3). The calibration data will be used to generate a geometric model for each camera.

Software Preparation

The software and files required for this test are prepared in advance of test day. This checklist ensures that the following are present, debugged, and executable: (1) all fast look scripts, (2) automated header generation of all relevant camera parameters, target positioning, and metadata, (3) all camera scripts that command the camera unit, and (4) the directories/file-paths pointing to the data repositories of this specific test.

Table 1. File naming convention for the camera script prefixes and frame filenames: "AAABBBBCDD"

Code	Name	Example	Value(s)
"AAA"	Calibration Plan Section	"465" = Cal. Plan 4.6.5 chapter 4, section 6, subsection 5	465-7
"BBBB"	Location of test or MSSS TVAC temperature	"TAMB" = ambient test at MSSS, "TN10" = MSSS TVAC at -10C, …	TAMB
"C"	Camera unit under test	"L" = Left Mastcam-Z, "R" = Right Mastcam-Z, "E" =EQ "C" =COTS	L/R
"DD"	Part of test	"00" = test set up, "01" = first part,…	00-13

1. **[D]** [illegible] Look up the daily calibration schedule and record the scheduled start and end time of this test on the cover page of this document. Also, fill out and double-check the other information on the cover page.

Date 5/7 Time 24 Initials [illegible]

2. **[D]** ___ Ensure that all supplemental manuals are on hand. These are,
    - Validator_Manual, Documentarian_Manual, MastcamZ_Data_Manual,
    - MastcamZCalPlan
3. **[D]** ___ Ensure that the Image Log is present and ready to use. Find and open the Google Sheets file "Image_Log_46". There is a link on the Wiki.
4. **[V]** ___ Check that all *Calgorithms* fast-look and validation scripts are present, up-to-date, and ready to analyze test output. Find and open the "Geometric_Calibration_46_Validation" Jupyter notebook. There is a link on the Wiki.
5. **[O]** ___ Check that all camera scripts required for this test are present, up-to-date and ready to command the ground support equipment (GSE). These are,
    - **465TAMBR01 - 465TAMBR09, 465TAMBL01 - 465TAMBL09**
    - **466TAMBR01 - 466TAMBR13, 466TAMBL01 - 466TAMBL13**
    - **467TAMBR01 - 467TAMBR04, 467TAMBL01 - 467TAMBL04**
6. **[O,V,D, L]** Notes:

____

____

____

Hardware Installation

This procedure is for the ambient cleanroom testing at MSSS. Figure 1 shows the nominal layout of the cleanroom, workspace, Mastcam-Zs, ground support equipment (GSE), targets, sources, and other equipment necessary.

Figure 1. MSSS Floor Plan for Geometric Testing in the Cleanroom Chamber.

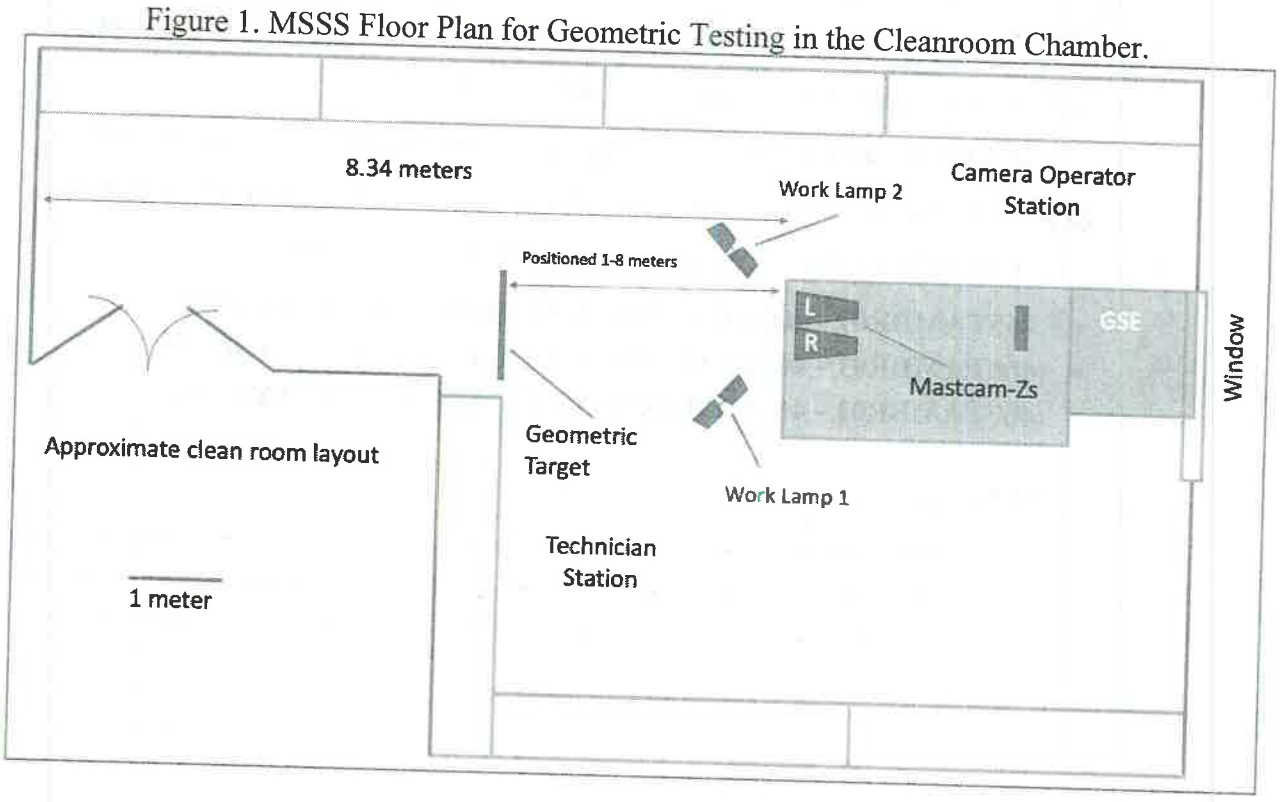

7. [T,O, L] ____ Ensure that all personnel in the cleanroom are following the cleanroom practices for electrostatic discharge, proper clothing, and other safety concerns. See "ESD_Manual" and "Cleanroom_Manual".
8. [T] ____ Double check that the ionizers are blowing over the Mastcam-Zs.
9. [O,T] ____ Ensure that the camera unit and GSE wires are secure, kink-free, and do not present tripping hazards.
10. [T] ____ Install the JR dot target on the tripod.
11. [T] ____ Install the blue and infrared bright lamps. Position them about in front of the geometric target out of the camera's field of view (FOV) with a high enough phase angle to prevent specular reflections. Power them on.

Date 5/7 Time 24 Initials [illegible]

12. **[O,D]** ___ Check the camera Temperature and ensure nominal operation.
13. **[D]** ___ Record the following environmental information:
    - Cleanroom Temperature N/A pressure ______ humidity ______
14. **[O,D, L]** Notes:

____

____

____

Fixed Target Positions for the 100mm Right and Left Mastcam-Z (Scene 16)

15. **[T]** CO Position the JR dot target approximately ~~7~~ 8 **meters** from the camera and finely adjust it to maximize the dots visible in both Mastcam-Z.
16. **[D]** CO Record the following temperatures:
    - Left Mastcam-Z CCD temp 24.1
    - Right Mastcam-Z CCD temp 24.7
17. **[D,T]** CO Take digital pictures of the geometric target's position, and the whole test/GSE set-up. PICTURES 390-398
18. **[O,T]** CO Capture test frames to finely position the target centered in the 1mm FOV of both cameras. Save these test frames with the prefix name **466TAMBL00** and **466TAMBR00**. FILTER 0 100 mm FOCUS: 8m

23:30

19. **[M]** CO Measure the locations of the geometric target and the camera.
20. **[M,D]** CO Record the location measurements in the Image Log and tables below.
21. **[M,D, L]** Notes: ALARM COMPANY ISSUE (>11PM) CAUSED DELAYS TEST FRAMES DONE ONCE.

Date 5/7 Time 23:30 Initials [illegible]

22. [O,T] ____ Load and execute the script **466TAMBL06**, which captures Z-stacks of 16 focus distances (from 1 meter to infinity) for filter 0 with seven focal lengths. The estimated duration is 10 minutes. ↳ STARTS AT 24mm
23. [O,T] ____ Load and execute the script **466TAMBR06**, which captures Z-stacks of 16 focus distances (from 1 meter to infinity) for filter 0 with seven focal lengths. The estimated duration is 10 minutes.
24. **[D]** ____ Record image names and parameters in Image Log.
25. [D, L] Notes: RUN IN PARALLEL. AE 155 → AE 100 (CHANGED)

LAMPS ON:

26. [O,T] ____ Load and execute the script **466TAMBL11**, which captures Z-stacks of 16 focus distances (from 1 meter to infinity) for each non-solar filter with the **100mm** focal length. The estimated duration is 15 minutes.
27. [O,T] ____ Load and execute the script **466TAMBR11**, which captures Z-stacks of 16 focus distances (from 1 meter to infinity) for each non-solar filter with the **100mm** focal length. The estimated duration is 15 minutes.
28. **[D]** ____ Record image names and parameters in Image Log.
29. [D, L] Notes: START TIME: 2355

Data Validation

30. **[V]** ____ Run the "Geometric_46_Validation" Jupyter notebook on the acquired data for the Right and Left Mastcam-Zs. This analysis can take place while the test continues.
31. **[V,D, L]** Notes: ____

Explanation of the Semi-Random Orientations

Figure 2. An example of the camera's FOV (black) and JR dot target's semi-random positions (red)

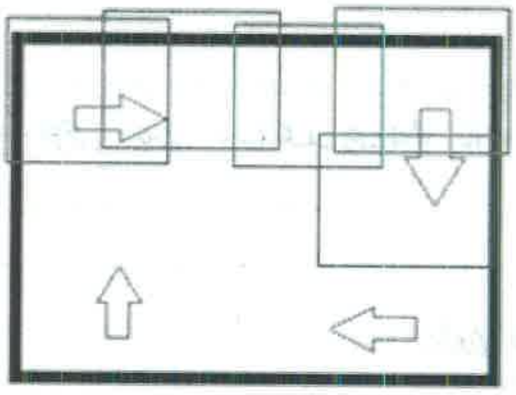

Figure 2 shows the desired orientations for the following tests that ask for a certain number of "semi-random orientations" of the JR dot target. The blue arrows show the motion of the target across the camera's FOV for optimal coverage. Note that some frames should be taken with the JR dot target rotated 90-degrees around the camera's line-of-sight.

Date 5/7 Time 23:30 Initials CS

40+ Target Positions for the 100mm Right and Left Mastcam-Zs (Scene 21)

skip

32. **[T]** ____ Position the JR dot target approximately **8 meters** from the camera. Adjust lights accordingly.
33. **[D]** ____ Record the following temperatures:
    - Left Mastcam-Z CCD temp ______________
    - Right Mastcam-Z CCD temp ______________
34. **[D,T]** ____ Take digital pictures of the geometric target's position, and the whole test/GSE set-up. 390 - 398

35. **[O,T]** ____ Capture test frames to find a standard exposure time for the 100 positions at 3 meters focus. Save these test frames with the prefix name **465TAMBL00**, and update "var1" in the script **465TAMBL06** once this exposure time is found. SKIP
36. **[V,O,T]** ____ Evaluate whether the target's dots are in-focus enough for discrimination. If the dots are too out-of-focus for JR's algorithm, move the target back. Also watch out for high levels of saturated pixels. SKIP

37. **[O,T]** ____ Capture test frames to find a standard exposure time for the 100 positions at 3 meters focus. Save these test frames with the prefix name **465TAMBR00**, and update "var1" in the script **465TAMBR06** once this exposure time is found. SKIP
38. **[V,O,T]** ____ Evaluate whether the target's dots are in-focus enough for discrimination. If the dots are too out-of-focus for JR's algorithm, move the target back. Also watch out for high levels of saturated pixels. SKIP

use ~~[illegible]~~ 30 msec exposure   START 1215

39. **[O,T]** ____ Load and begin the script **465TAMBL06**, which captures frames with filter 0 at ~~26mm~~ 100mm focal length one frame at a time, with a pause command between each frame.
40. **[O,T]** ____ Load and begin the script **465TAMBR06**, which captures frames with filter 0 at ~~26mm~~ 100mm focal length one frame at a time, with a pause command between each frame.

41. **[O,T] ____** Capture **10 images** of the JR dot target in semi-random orientations (see Figure 2) normal to the camera approximately **8-meter** distance covering each edge of the camera's FOV. Adjust lighting if necessary, to keep the frames at approximately 50% full-well.

42. **[O,T] ____** Capture **10 images** of the JR dot target in semi-random orientations (see Figure 2) normal to the camera approximately **6-meter** distance covering each edge of the camera's FOV. Adjust lighting if necessary, to keep the frames at approximately 50% full-well.

43. **[O,T] ____** Capture **10 images** of the JR dot target in semi-random orientations (see Figure 2) normal to the camera approximately **4-meter** distance covering each edge of the camera's FOV. Adjust lighting if necessary, to keep the frames at approximately 50% full-well.

44. **[O,T] ____** Capture **5 images** of the JR dot target in semi-random orientations (see Figure 2) normal to the camera approximately **2-meter** distance covering each edge of the camera's FOV. Adjust lighting if necessary, to keep the frames at approximately 50% full-well.

45. **[V,O,T]** ____ Evaluate whether the target's dots are evenly distributed over each fields of view. Also watch out for high levels of saturated pixels.
46. **[O,T, L]** ____ After more than 100 usable frames have been captured, stop the prefix script.
47. **[D]** ____ Record image names and parameters in Image Log.
48. **[D, L]** Notes:____

Data Validation

49. **[V]** ____ Run the "Geometric_46_Validation" Jupyter notebook on the acquired data for for the Right and Left Mastcam-Zs. This analysis can take place while the test continues.
50. **[V,D, L]** Notes: ____

100+ Target Positions for the 63mm Right and Left Mastcam-Zs (Scene 22)

51. **[T]** ____ Position the JR dot target approximately **2 meters** from the camera. Adjust lights accordingly.
52. **[D]** ____ Record the following temperatures:
    - Left Mastcam-Z CCD temp ______________
    - Right Mastcam-Z CCD temp ______________
53. **[D,T]** ____ Take digital pictures of the geometric target's position, and the whole test/GSE set-up.

54. **[O,T]** ____ Capture test frames to find a standard exposure time for the 100 positions at 3 meters focus. Save these test frames with the prefix name **465TAMBL00**, and update "var1" in the script **465TAMBL04** once this exposure time is found.
55. **[V,O,T]** ____ Evaluate whether the target's dots are in-focus enough for discrimination. If the dots are too out-of-focus for JR's algorithm, move the target back. Also watch out for high levels of saturated pixels.

56. **[O,T]** ____ Capture test frames to find a standard exposure time for the 100 positions at 3 meters focus. Save these test frames with the prefix name **465TAMBR00**, and update "var1" in the script **465TAMBR04** once this exposure time is found.
57. **[V,O,T]** ____ Evaluate whether the target's dots are in-focus enough for discrimination. If the dots are too out-of-focus for JR's algorithm, move the target back. Also watch out for high levels of saturated pixels.

58. **[O,T]** ____ Load and begin the script **465TAMBL04**, which captures frames with filter 0 at **63mm** focal length one frame at a time, with a pause command between each frame.
59. **[O,T]** ____ Load and begin the script **465TAMBR04**, which captures frames with filter 0 at63 focal length one frame at a time, with a pause command between each frame.

60. [O,T] ____ Capture approximately **10 images** of the JR dot target in semi-random orientations (see Figure 2) normal to the camera approximately **2-meter** distance covering each edge of the camera's FOV. Adjust lighting if necessary, to keep the frames at approximately 50% full-well.
61. [O,T] ____ Capture approximately **20 images** of the JR dot target in semi-random orientations (see Figure 2) normal to the camera approximately **3-meter** distance covering each edge of the camera's FOV. Adjust lighting if necessary, to keep the frames at approximately 50% full-well.
62. [O,T] ____ Capture approximately **20 images** of the JR dot target in semi-random orientations (see Figure 2) normal to the camera approximately **4-meter** distance covering each edge of the camera's FOV. Adjust lighting if necessary, to keep the frames at approximately 50% full-well.
63. [O,T] ____ Capture approximately **20 images** of the JR dot target in semi-random orientations (see Figure 2) normal to the camera approximately **5-meter** distance covering each edge of the camera's FOV. Adjust lighting if necessary, to keep the frames at approximately 50% full-well.
64. [O,T] ____ Capture approximately **20 images** of the JR dot target in semi-random orientations (see Figure 2) normal to the camera approximately **6-meter** distance covering each edge of the camera's FOV. Adjust lighting if necessary, to keep the frames at approximately 50% full-well.
65. [O,T] ____ Capture approximately **10 images** of the JR dot target in semi-random orientations (see Figure 2) normal to the camera approximately **8-meter** distance covering each edge of the camera's FOV. Adjust lighting if necessary, to keep the frames at approximately 50% full-well.

66. **[V,O,T]** ____ Evaluate whether the target's dots are evenly distributed over each fields of view. Also watch out for high levels of saturated pixels.
67. **[O,T, L]** ____ After more than 100 usable frames have been captured, stop the prefix script.
68. **[D]** ____ Record image names and parameters in Image Log.
69. **[D, L]** Notes:____

____

____

Data Validation

70. **[V]** ____ Run the "Geometric_46_Validation" Jupyter notebook on the acquired data for for the Right and Left Mastcam-Zs. This analysis can take place while the test continues.
71. **[V,D, L]** Notes: ____

____

____

100+ Target Positions for the 26mm Right and Left Mastcam-Zs (Scene 21)

72. **[T]** ____ Position the JR dot target approximately **1.5 meters** from the camera. Adjust lights accordingly.
73. **[D]** ____ Record the following temperatures:
    - Left Mastcam-Z CCD temp ______________
    - Right Mastcam-Z CCD temp ______________
74. **[D,T]** ____ Take digital pictures of the geometric target's position, and the whole test/GSE set-up. 390 - 398

BEGIN 12:21 EXP TIME: 30 msec

SKIP

75. **[O,T]** ____ Capture test frames to find a standard exposure time for the 100 positions at 3 meters focus. Save these test frames with the prefix name **465TAMBL00**, and update "var1" in the script **465TAMBL01** once this exposure time is found.
76. **[V,O,T]** ____ Evaluate whether the target's dots are in-focus enough for discrimination. If the dots are too out-of-focus for JR's algorithm, move the target back. Also watch out for high levels of saturated pixels.

SKIP

77. **[O,T]** ____ Capture test frames to find a standard exposure time for the 100 positions at 3 meters focus. Save these test frames with the prefix name **465TAMBR00**, and update "var1" in the script **465TAMBR01** once this exposure time is found.
78. **[V,O,T]** ____ Evaluate whether the target's dots are in-focus enough for discrimination. If the dots are too out-of-focus for JR's algorithm, move the target back. Also watch out for high levels of saturated pixels.

BEGIN 12:21 EXP = 30 msec

79. **[O,T]** ____ Load and begin the script **465TAMBL01**, which captures frames with filter 0 at **26mm** focal length one frame at a time, with a pause command between each frame.
80. **[O,T]** ____ Load and begin the script **465TAMBR01**, which captures frames with filter 0 at **26mm** focal length one frame at a time, with a pause command between each frame.

81. **[O,T]** ____ Capture approximately **10 images** of the JR dot target in semi-random orientations (see Figure 2) normal to the camera approximately **1.5-meter** distance covering each edge of the camera's FOV.
82. **[O,T]** ____ Capture approximately **10 images** of the JR dot target in semi-random orientations (see Figure 2) normal to the camera approximately **2-meter** distance covering each edge of the camera's FOV. Adjust lighting if necessary, to keep the frames at approximately 50% full-well.
83. **[O,T]** ____ Capture approximately **25 images** of the JR dot target in semi-random orientations (see Figure 2) normal to the camera approximately **3-meter** distance covering each edge of the camera's FOV. Adjust lighting if necessary, to keep the frames at approximately 50% full-well.
84. **[O,T]** ____ Capture approximately **30 images** of the JR dot target in semi-random orientations (see Figure 2) normal to the camera approximately **4-meter** distance covering each edge of the camera's FOV. Adjust lighting if necessary, to keep the frames at approximately 50% full-well.
85. **[O,T]** ____ Capture approximately **10 images** of the JR dot target in semi-random orientations (see Figure 2) normal to the camera approximately **5-meter** distance covering each edge of the camera's FOV. Adjust lighting if necessary, to keep the frames at approximately 50% full-well.
86. **[O,T]** ____ Capture approximately **5 images** of the JR dot target in semi-random orientations (see Figure 2) normal to the camera approximately **6-meter**.
87. **[O,T]** ____ Capture approximately **5 images** of the JR dot target in semi-random orientations (see Figure 2) normal to the camera approximately **7-meter**.
88. **[O,T]** ____ Capture approximately **5 images** of the JR dot target in semi-random orientations (see Figure 2) normal to the camera approximately **8-meter**.

89. **[V,O,T]** ____ Evaluate whether the target's dots are evenly distributed over each fields of view. Also watch out for high levels of saturated pixels.
90. **[O,T, L]** ____ After more than 100 usable frames have been captured, stop the prefix script.
91. **[D]** ____ Record image names and parameters in Image Log.
92. **[D, L]** Notes:____

____

____

Data Validation

93. [V] ____ Run the "Geometric_46_Validation" Jupyter notebook on the acquired data for for the Right and Left Mastcam-Zs. This analysis can take place while the test continues.
94. [V,D, L] Notes:____

____

____

Date 5/8 Time 00:30 Initials CS

Shutdown Procedure

95. [D,T] CS Take digital pictures of the test setup.
96. [D,O] CS Review entries in Image Log, GSE command log, and image headers.
97. [D, L] CS Review calibration procedure and ensure that each task is initialed.
98. [D, L] Notes: ______________________________

99. [V, L] CS Before making the decision to break down the test setup, ensure that adequate data were acquired for the test requirements. See "MastcamZCalPlan" for these requirements.
100. [V] Notes: ______________________________

Data Validator (signature) Ole B. Jensen

Date 9/5-2019 Time 12:35 AM

101. [V, L] CS Give the go/no-go decision. Have enough data been acquired to fulfill test requirements? See "MastcamZCalPlan" for these requirements.
102. [D, L] CS Update the Log Document. — ??
103. [L] Notes: ______________________________

Calibration Lead (signature) [illegible signature]

Date 5/8/19 Time 00:30

104. **[O, L]** JVB Ensure that the camera and GSE are in a safe state.
105. **[O,D]** JVB Review the Image Log with the documentarian. Exchange high-fives.
106. **[O]** Notes: Nothing of Note

Camera Operator (signature) Angela Magee

Date 5/8/19 Time 12:35AM

107. **[T]** ____ If the next test does not require the target, position it away from the chamber or bench. Otherwise, be sure not to move it. The next test is ________________.
108. **[T]** ____ Ensure that all other test equipment is safely put away.
109. **[T]** Notes: ____________________

Technician (signature) Christian Tate

Date 5/8/19 Time 00:30

110. **[D, L]** CT Double-check this procedure and ensure that the top of each page has valid data, time and initials.
111. **[D]** CT Photo-scan this document, save it on the cloud, and file the hard-copy in the Log Binder. Upload the digital pictures taken during this test in the appropriate archive on the cloud. The required links are on the Wiki.
112. **[D]** CT Double-check that every required cell the Image Log is accurately filled. When this is complete, print the Image Log and file it the Log Binder after this document.
113. **[D]** Notes: ____________________

Documentarian (signature) [illegible]

Date May 8 Time 12:35
