# Supplementary material for: Pre-Flight Calibration of the Mars 2020 Rover Mastcam Zoom (Mastcam-Z) Multispectral, Stereoscopic Imager
Source: Space Sci Rev. 2021 Feb 18;217(2):29. doi: 10.1007/s11214-021-00795-x (PMC7892537; doi:10.1007/s11214-021-00795-x)
Supplement: Supplementary file 1 — (ZIP 98.6 MB) [file 11214_2021_795_MOESM1_ESM.zip › CalPro_466_JR_Geometric_v2_07_TVAC_Ambient.pdf]

 Date 5/1 Time 8:30 Initials [illegible]

JR Geometric Calibration Procedure for the Right and Left Mastcam-Z Ambient TVAC Testing at MSSS (Pro. 4.6.6)

[Procedure version 2.07, prepared by the Mastcam-Z calibration team at Cornell University]

These measurements are performed on the camera and at the Temperature designated below as specified in the Mastcam-Z Calibration Plan,

Unit Under Test:

Left FM X **Right FM** X **EQM**____ **Other**________

These measurements are performed at Temperature:

-35°C____ **-10°C**____ **+5°C**____ **Ambient** X **Other**________

These measurements are performed at,

MSSS X **ASU**____ **Other**____

Date 5/1/19 **Start Time** 8:30 **End Time** 11:30

Estimated Duration 3.0 hours

Scheduled Start Time 8:00 **Sch. End Time** 11:00

Calibration Lead [L] Maki **Documentarian [D]** Christian, Megan (shadow)

Camera Operator [O] Elsa/Tex, Angela shadow **Technician [T]** Andy

Data Validator [V] Paul, Ole (shadow) **Metrologist [M]** N/A

Other ________

Date 5/1 Time 8:47 Initials [illegible]

Change Log

Version	Name	Change
v1_01<br>26 Sep 2018	C. Tate	(first draft)
v1_07<br>1 Nov 2018	C. Tate	Procedure edits prior to EQM testing
V1_07-JR<br>8 Nov 2018	G. Paar	Distances more precisely reflected, change mode from v06 to v07 kept, fixed focus consistently at 2 tables & figure automatically referenced
v1_10<br>13 Dec. 2018	C. Tate	Procedure edits after EQM testing
v2_07<br>1 May 2019	C. Tate	Approved version prior to FM testing

Document Approval

[signature] 5/1/19
Approved by James Bell Date
Mastcam-Z PI
Arizona State University

[signature] 5/6/19
Approved by Alexander Hayes Date
Mastcam-Z Calibration Working Group
Lead, Cornell University

[signature] 5/1/19
Approved by Justin Maki Date
Mastcam-Z Deputy PI and Investigation
Scientist, Jet Propulsion Laboratory

[signature] 5/1/19
Approved by: Date
Ken Herkenhoff
Mastcam-Z Co-Investigator, USGS

[signature] 5/1/19
Approved by Christian Tate Date
Procedure Author
Cornell University

Date 5/1 Time 8:47 Initials [illegible]

Table of Contents

JR GEOMETRIC CALIBRATION PROCEDURE FOR THE RIGHT AND LEFT MASTCAM-Z AMBIENT TVAC TESTING AT MSSS (PRO. 4.6.6).. 1

Change Log.. 2
Document Approval.. 2
Test Description.. 4
Software Preparation.. 4
Table 1. File naming convention for the camera script prefixes and frame filenames: "AAABBBBCDD"............ 4
Hardware Installation.. 6
Figure 1. ASU Floor Plan for Geometric Testing in the TVAC Chamber. The MSSS Floor Plan allows for similar target and source placements relative to the chamber window.. 6
Fixed Target Positions for the 63mm Right and Left Mastcam-Z (Scene 7, window on).. 8
Data Validation.. 10
Open Chamber Door.. 10
Fixed Target Positions for the 63mm Right and Left Mastcam-Z (Scene 8, window off).. 11
Data Validation.. 13
Remove Test Equipment.. 13

SHUTDOWN PROCEDURE.. 14

Date 5/1 Time 8:48 Initials [illegible]

Test Description

Excerpt from the Calibration Plan 4.6

> The objective of Geometric Calibration is to characterize the geometric distortion introduced by the Mastcam-Z optics into its images and measure the effective focal length and field of view at each focus and zoom position. As the range of zoom positions available to Mastcam-Z represent a continuum, measurements will be acquired at a finite number of zoom settings and then interpolated to characterize distortion and other geometric parameters across the full zoom range. Targets should be imaged at ~50% full well using the Bayer RGB/805 nm (priority 1) and remaining non-solar filters (priority 3). The calibration data will be used to generate a geometric model for each camera.

Software Preparation

The software and files required for this test are prepared in advance of test day. This checklist ensures that the following are present, debugged, and executable: (1) all fast look scripts, (2) automated header generation of all relevant camera parameters, target positioning, and metadata, (3) all camera scripts that command the camera unit, and (4) the directories/file-paths pointing to the data repositories of this specific test.

Table 1. File naming convention for the camera script prefixes and frame filenames: "AAABBBBCDD"

**Code**	**Name**	**Example**	**Value(s)**
"AAA"	Calibration Plan Section	"465" = Cal. Plan 4.6.5 chapter 4, section 6, subsection 5	466
"BBBB"	Location of test or ASU Chamber Temperature	"ATLO" = test at JPL ATLO, "TN10" = MSSS TVAC -10C, …	TAMB
"C"	Camera unit under test	"L" = Left Mastcam-Z, "R" = Right Mastcam-Z, "E" =EQ "C" =COTS	L/R
"DD"	Part of test	"00" = test set up, "01" = first part,…	00-13

1. **[D]** ✓ Look up the daily calibration schedule and record the scheduled start and end time of this test on the cover page of this document. Also, fill out and double-check the other information on the cover page.

Date 5/1 Time 8:48 Initials [illegible]

2. **[D]** ✓ Ensure that all supplemental manuals are on hand. These are,
   - ✓Validator_Manual, ✓Documentarian_Manual, ~~MastcamZ_Data_Manual~~,
   - MastcamZCalPlan✓
3. **[D]** ✓ Ensure that the Image Log is present and ready to use. Find and open the Google Sheets file "Image_Log_46". There is a link on the Wiki.
4. **[V]** ✓ Check that all *Calgorithms* fast-look and validation scripts are present, up-to-date, and ready to analyze test output. Find and open the "Geometric_Calibration_46_Validation" Jupyter notebook. There is a link on the Wiki.
5. **[O]** ✓ Check that all camera scripts required for this test are present, up-to-date and ready to command the ground support equipment (GSE). These are,
   - **466TAMBR01 - 466TAMBR13, 466TAMBL01 - 466TAMBL13**
6. **[O,V,D, L]** Notes:

Date 5/1 Time 8:54 Initials [illegible]

Hardware Installation

This procedure is for the ambient TVAC chamber testing at MSSS. Figure 1 shows the nominal layout of the TVAC chamber, workspace, Mastcam-Zs, ground support equipment (GSE), targets, sources, and other equipment necessary for this test if it happens at ASU. Although MSSS' cleanroom is different than ASU's, the placement of the targets and sources relative to the chamber window is similar.

map is out of date

Figure 1. ASU Floor Plan for Geometric Testing in the TVAC Chamber. The MSSS Floor Plan allows for similar target and source placements relative to the chamber window.

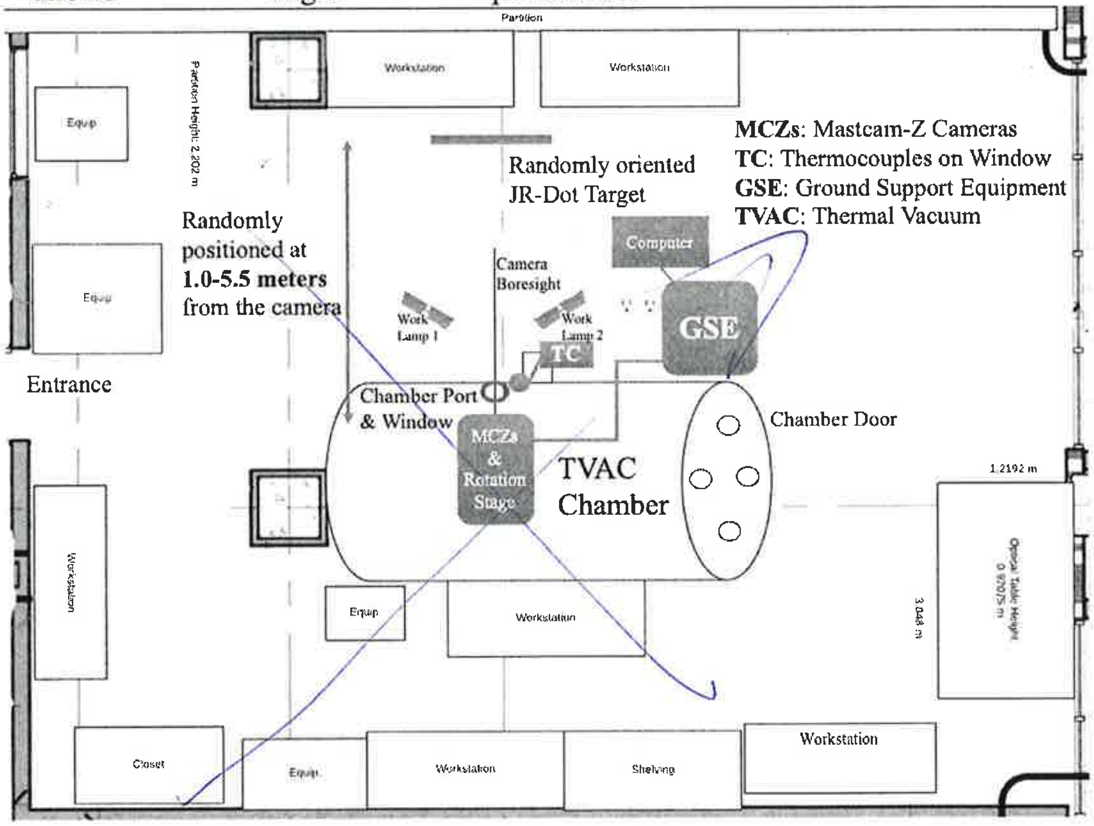

7. [T,O, L] ✓ Ensure that all personnel in the cleanroom are following the cleanroom practices for electrostatic discharge, proper clothing, and other safety concerns. See "ESD_Manual" and "Cleanroom_Manual".
8. [T] ✓ Double check that nitrogen is flowing over the Mastcam-Zs or the window port.

air ionizer

Date 5/1 Time 8:59 Initials [illegible]

9. **[O,T]** ____ skip ~~If not already~~ done done, mate the Right Mastcam-Z into the GSE. Follow the procedure in "MastcamZ_GSE_Manual".

10. **[T]** ✓ Verify that the thermocouples are turned on and properly reading out. Aux platen: 29.27 °C

11. **[T]** ~~____ If necessary, position the JR dot target approximately **3 meters** from the cameras.~~ done

12. **[T]** ✓ Place the Macbeth target in front of the JR dot target.

13. **[T]** ✓ Install the lamps and position them about 1 meter from the geometric target out of the camera's field of view (FOV). Power them on. Right Camera

14. **[O,T]** ✓ Ensure that the camera unit and GSE wires are secure, kink-free, and do not present tripping hazards when the lights are turned off.

15. **[O,D]** ✓ Check the camera Temperature and ensure nominal operation. L 29.4 °C R 30.2 °C

16. **[D]** ✓ Record the following environmental information:

- Cleanroom Temperature 25.5 °C ~~pressure~~ n/a humidity 38%

17. **[O,D, L]** Notes:

____

____

____

Date 5/1 Time 7:30 Initials [illegible]

Fixed Target Positions for the 63mm Right and Left Mastcam-Z (Scene 7, window on)

18. **[T]** ____ Position the JR dot target approximately **3 meters** from the camera.
19. **[D]** ____ Record the following Temperatures:
    - Chamber Temp ______________ Port Temp ______________
    - Camera CCD Temp ______________ Optics Temp ______________
20. **[D,T]** ✓ Take digital pictures of the geometric target's position, and the whole test/GSE set-up.
21. **[O,T]** ✓ Capture test frames to finely position the target centered in the 34mm FOV of both cameras. Save these test frames with the prefix name **466TAMBR00** and **466TAMBL00**. Make sure that the Macbeth target is fully visible and properly illuminated through the window (the whole target may have to move up). 48 mm suffix: 2
22. **[M]** ✓ Measure the locations of the geometric target and the camera.
23. **[M,D]** ✓ Record the location measurements in the Image Log and tables below.

Target Location	Metrology ID#
Reference	
Top-Left Nest	
Top-Right Nest	
Bottom-Left Nest	
Bottom-Right Nest	

Camera/Chamber Location	Metrology ID#
Reference	
Nest 1	
Nest 2	
Nest 3	

24. **[M,D, L]** Notes: distance = 1.63 cm from bar to target (Macbeth)

Su.

Date 5/1 Time 9:30 Initials [illegible]

25. **[O,T]** ✓ Load and execute the script **466TAMBL10** 9, which captures Z-stacks of 16 focus distances (from 1 meter to infinity) for each non-solar filter with the ~~63mm~~ 43 34mm focal length. The estimated duration is ~~10~~ 9 minutes.
26. **[O,T]** ✓ Load and execute the script **466TAMBR10** 9, which captures Z-stacks of 16 focus distances (from 1 meter to infinity) for each non-solar filter with the ~~63mm~~ 34mm focal length. The estimated duration is 10 minutes.
27. **[D]** ✓ Record image names and parameters in Image Log.
28. **[D, L]** Notes:______________________________

29. **[T]** ✓ Remove the Macbeth target. Take note of the placement. ✓

L = 29.9 °C
R = 30.7 °C
suffix: 118

30. **[O,T]** ✓ Load and execute the script **466TAMBL06**, which captures Z-stacks of 16 focus distances (from 1 meter to infinity) for filter 0 with seven focal lengths. The estimated duration is 10 minutes.
31. **[O,T]** ✓ Load and execute the script **466TAMBR06**, which captures Z-stacks of 16 focus distances (from 1 meter to infinity) for filter 0 with seven focal lengths. The estimated duration is 10 minutes.
32. **[D]** ✓ Record image names and parameters in Image Log.
33. **[D, L]** Notes:______________________________

suffix: 118
L = 30.1 °C
R = 30.8 °C

Date 5/1 Time 10:27 Initials [illegible]

Data Validation

34. **[V]** ✓ Run the "Geometric_46_Validation" Jupyter notebook on the acquired data for for the Right and Left Mastcam-Zs. This analysis can take place while the test continues.
35. **[V,D, L]** Notes: ______________________________

35.a. ✓ Power off camera heads

36. **[T]** ✓ **Be sure that the target and lamps do not move. Ensure that proper warning is posted for the people who may come into the room in our absence. Post a DO NOT MOVE sign.**

Open Chamber Door

37. **[O,T]** ✓ Call in the hardware team to open the door WITHOUT moving the target, lights, or Mastcam-Zs.
38. **[O,T, D, L]** Notes: Acquire image & setup 10:28 AM

Date 5/1 Time 10:32 Initials [illegible]

Fixed Target Positions for the 63mm Right and Left Mastcam-Z (Scene 8, window off)

39. **[[redacted],T]** ✓ Load and execute the script **466TAMBL06**, which captures Z-stacks of 16 focus distances (from 1 meter to infinity) for filter 0 with seven focal lengths. Insert note "Window=0". The estimated duration is ~~10~~ 15 minutes.
40. **[[redacted],T]** ✓ Load and execute the script **466TAMBR06**, which captures Z-stacks of 16 focus distances (from 1 meter to infinity) for filter 0 with seven focal lengths. Insert note "Window=0". The estimated duration is ~~10~~ 15 minutes.
41. **[D]** ✓ Record image names and parameters in Image Log.
42. **[D, L]** Notes: Validated per validator
    Note: "window=0" not recorded in image header

43. **[[redacted],T]** ✓ If time permits, load and execute the script **466TAMBL07**, which seven focal lengths in reverse order (110, 100, …) for 3 meter focus with 0 filter. Insert note "Window=0". The estimated duration is 3 minutes.
44. **[O,T]** ✓ If time permits, load and execute the script **466TAMBR07**, which seven focal lengths in reverse order (110, 100, …) for 3 meter focus with 0 filter. Insert note "Window=0". The estimated duration is 3 minutes.
45. **[D]** ✓ Record image names and parameters in Image Log.
46. **[D, L]** Notes: final suffix = 6

L = 30.0°C
R = 30.8°C

✓ 46a. validated per validator

Date 5/1 Time 11:00 Initials

47. **[T]** ✓ Insert the Macbeth target in approximately the same place.

48. **[O,T]** ✓ Load and execute the script **466TAMBL10**, which captures Z-stacks of 16 focus distances (from 1 meter to infinity) for each non-solar filter with the **63mm** focal length. Insert note "Window=0". The estimated duration is 10 minutes.

49. **[O,T]** ✓ Load and execute the script **466TAMBR10**, which captures Z-stacks of 16 focus distances (from 1 meter to infinity) for each non-solar filter with the **63mm** focal length. Insert note "Window=0". The estimated duration is 10 minutes.

50. **[D]** ✓ Record image names and parameters in Image Log.

51. **[D, L]** Notes: Validated per validator

Data Validation

52. **[V]** ✓ Run the "Geometric_46_Validation" Jupyter notebook on the acquired data for for the Right and Left Mastcam-Zs. This analysis can take place while the test continues.
53. **[V,D, L]** Notes: validated
    shadow on JR target

Remove Test Equipment

54. **[T]** ✓ Carefully remove the target, lights and other test equipment from the chamber room.
55. **[T, L]** Notes: ______

Date 5/1 Time 11:49 Initials gM

Shutdown Procedure

56. **[D,T]** ✓ Take digital pictures of the test setup. "tons" of pictures
57. ~~[D,O]~~ ✓ Review entries in Image Log, GSE command log, and image headers.
58. **[D, L]** ✓ Review calibration procedure and ensure that each task is initialed.
59. **[D, L]** Notes: ______

60. **[V, L]** ✓ Before making the decision to break down the test setup, ensure that adequate data were acquired for the test requirements. See "MastcamZCalPlan" for these requirements.
61. **[V]** Notes: ______

Data Validator (signature) [signature]

Date 5/1/19 Time 11:35 AM

62. **[V, L]** ✓ Give the go/no-go decision. Have enough data been acquired to fulfill test requirements? See "MastcamZCalPlan" for these requirements.
63. **[D, L]** ____ Update the Log Document.
64. **[L]** Notes: ______

Calibration Lead (signature) [signature]

Date 5/1/19 Time 11:35 AM

Date 5/1 Time 11:49 Initials JR

perform prior to step 56.

65. [O, L] ✓ Ensure that the camera and GSE are in a safe state.
66. [O,D] ✓ Review the Image Log with the documentarian. Exchange high-fives.
67. [O] Notes: ______

Camera Operator (signature) ______

Date 5/1/2019 Time 11:38

68. [T] ✓ If the next test does not require the target, position it away from the chamber or bench. Otherwise, be sure not to move it. The next test is going to be in the cleanroom.
69. [T] ✓ Ensure that all other test equipment is safely put away.
70. [T] Notes: None

Technician (signature) ______

Date 5/1/19 Time 11:50

71. [D, L] ✓ Double-check this procedure and ensure that the top of each page has valid data, time and initials.
72. [D] ______ Photo-scan this document, save it on the cloud, and file the hard-copy in the Log Binder. Upload the digital pictures taken during this test in the appropriate archive on the cloud. The required links are on the Wiki.
73. [D] ______ Double-check that every required cell the Image Log is accurately filled. When this is complete, print the Image Log and file it the Log Binder after this document.
74. [D] Notes: ______

Documentarian (signature) Megan Barrington

Date 1 May 2019 Time 12:33 pm
