# Supplementary material for: Pre-Flight Calibration of the Mars 2020 Rover Mastcam Zoom (Mastcam-Z) Multispectral, Stereoscopic Imager
Source: Space Sci Rev. 2021 Feb 18;217(2):29. doi: 10.1007/s11214-021-00795-x (PMC7892537; doi:10.1007/s11214-021-00795-x)
Supplement: Supplementary file 1 — (ZIP 98.6 MB) [file 11214_2021_795_MOESM1_ESM.zip › CalPro_468_JR_Geometric_v2_04.pdf]

JR Geometric Calibration Procedure for the Right and Left Mastcam-Z TVAC Ramping at MSSS (Pro. 4.6.8)

[Procedure version 2.04, prepared by the Mastcam-Z calibration team at Cornell University]

These measurements are performed on the camera and at the temperature designated below as specified in the Mastcam-Z Calibration Plan,

Unit Under Test:

Left FM __X__ Right FM __X__ EQM_____ Other__________

These measurements are performed at temperature:

-35°C_____ -10°C_____ +5°C_____ Ambient_____ Other -10 to +35 ramp

These measurements are performed at,

MSSS __X__ ASU_____ Other_____

Date 4-29-2019 **Start Time** 10:05 **End Time** ~~16:10~~ 13:15

Estimated Duration 3.0 hours

Scheduled Start Time 9:00 **Sch. End Time** 12:00

Calibration Lead [L] Justin Maki **Documentarian [D]** Christian Tate

Camera Operator [[redacted]] Tex & Elsa Jensen **Technician [T]** Andy Windhold

Data Validator [V] Paul Corlies **Metrologist [M]** __________

Other __________

Change Log

Version	Name	Change
v1_01<br>26 Sep 2018	C. Tate	(first draft)
v1_07<br>1 Nov 2018	C. Tate	Procedure edits prior to EQM testing
V1_07-JR<br>8 Nov 2018	G. Paar	Distances more precisely reflected, change mode from v06 to v07 kept, fixed focus consistently at 2 tables & figure automatically referenced
v1_10<br>13 Dec. 2018	C. Tate	Procedure edits after EQM testing
v2_04<br>29 April 2019	C. Tate	Approved version prior to FM testing

Document Approval

x [signature] 5/6/19
Approved by James Bell Date
Mastcam-Z PI
Arizona State University

x [signature] 5/6/19
Approved by Alexander Hayes Date
Mastcam-Z Calibration Working Group
Lead, Cornell University

x [signature] 4/30/19
Approved by Justin Maki Date
Mastcam-Z Deputy PI and Investigation
Scientist, Jet Propulsion Laboratory

[signature] 4/29/19
Approved by Christian Tate Date
Procedure Author
Cornell University

Approved by: Date

Table of Contents

JR GEOMETRIC CALIBRATION PROCEDURE FOR THE RIGHT AND LEFT MASTCAM-Z TVAC RAMPING AT MSSS (PRO. 4.6.8) 1

CHANGE LOG 2
DOCUMENT APPROVAL 2
TEST DESCRIPTION 4
SOFTWARE PREPARATION 4
Table 1. File naming convention for the camera script prefixes and frame filenames: "AAABBBBCDD" 4
HARDWARE INSTALLATION 6
Figure 1. ASU Floor Plan for Geometric Testing in the TVAC Chamber. The MSSS Floor Plan allows for similar target and source placements relative to the chamber window. 6
TARGET METROLOGY 8
TEST SCRIPTS THE RIGHT AND LEFT MASTCAM-ZS 9
TEMP AT RIGHT AND LEFT MASTCAM-ZS 10
TEMP AT RIGHT AND LEFT MASTCAM-ZS 11
TEMP AT RIGHT AND LEFT MASTCAM-ZS 12
TEMP AT RIGHT AND LEFT MASTCAM-ZS 13
TEMP AT RIGHT AND LEFT MASTCAM-ZS 14
TEMP AT RIGHT AND LEFT MASTCAM-ZS 15
TEMP AT RIGHT AND LEFT MASTCAM-ZS 16
TEMP AT RIGHT AND LEFT MASTCAM-ZS 17
TEMP AT RIGHT AND LEFT MASTCAM-ZS 18
TEMP AT RIGHT AND LEFT MASTCAM-ZS 19
TEMP AT RIGHT AND LEFT MASTCAM-ZS 20
TEMP AT RIGHT AND LEFT MASTCAM-ZS 21
TEMP AT RIGHT AND LEFT MASTCAM-ZS 22
TEMP AT RIGHT AND LEFT MASTCAM-ZS 23
TEMP AT RIGHT AND LEFT MASTCAM-ZS 24
TEMP AT RIGHT AND LEFT MASTCAM-ZS 25
TEMP AT RIGHT AND LEFT MASTCAM-ZS 26
TEMP AT RIGHT AND LEFT MASTCAM-ZS 27
TEMP AT RIGHT AND LEFT MASTCAM-ZS 28
TEMP AT RIGHT AND LEFT MASTCAM-ZS 29
TEMP AT RIGHT AND LEFT MASTCAM-ZS 30
TEMP AT RIGHT AND LEFT MASTCAM-ZS 31
TEMP AT RIGHT AND LEFT MASTCAM-ZS 32
DATA VALIDATION 33
DARK CURRENT WITH THE RIGHT AND LEFT MASTCAM-ZS 34
DATA VALIDATION 35

SHUTDOWN PROCEDURE 36

Test Description

Excerpt from the Calibration Plan 4.6

> The objective of Geometric Calibration is to characterize the geometric distortion introduced by the Mastcam-Z optics into its images, and measure the effective focal length and field of view at each focus and zoom position. As the range of zoom positions available to Mastcam-Z represent a continuum, measurements will be acquired at a finite number of zoom settings and then interpolated to characterize distortion and other geometric parameters across the full zoom range. Targets should be imaged at ~50% full well using the Bayer RGB/805 nm (priority 1) and remaining non-solar filters (priority 3). The calibration data will be used to generate a geometric model for each camera. The camera models may exhibit wavelength dependence, so an attempt to measure the effect overall filters is desired (although not required).

Software Preparation

The software and files required for this test are prepared in advance of test day. This checklist ensures that the following are present, debugged, and executable: (1) all fast look scripts, (2) automated header generation of all relevant camera parameters, target positioning, and metadata, (3) all camera scripts that command the camera unit, and (4) the directories/file-paths pointing to the data repositories of this specific test.

Table 1. File naming convention for the camera script prefixes and frame filenames: "AAABBBBCDD"

Code	Name	Example	Value(s)
"AAA"	Calibration Plan Section	"465" = Cal. Plan 4.6.5 chapter 4, section 6, subsection 5	468, 473, 441
"BBBB"	Location of test or ASU Chamber temperature	"ATLO" = test at JPL ATLO, "TN10" = MSSS TVAC -10C, …	TEMP
"C"	Camera unit under test	"L" = Left Mastcam-Z, "R" = Right Mastcam-Z, "E" =EQ "C" =COTS	L/R
"DD"	Part of test	"00" = test set up, "01" = first part,…	00-13

Date 4-29 Time 2.47 Initials CO

1. **[D]** CS Look up the daily calibration schedule and record the scheduled start and end time of this test on the cover page of this document. Also, fill out and double-check the other information on the cover page.
2. **[D]** CS Ensure that all supplemental manuals are on hand. These are,
   - Validator_Manual, Documentarian_Manual, MastcamZ_Data_Manual,
   - MastcamZCalPlan
3. **[D]** CS Ensure that the Image Log is present and ready to use. Find and open the Google Sheets file "Image_Log_46". There is a link on the Wiki.
4. **[V]** Cd Check that all *Calgorithms* fast-look and validation scripts are present, up-to-date, and ready to analyze test output. Find and open the "Geometric_Calibration_46_Validation" Jupyter notebook. There is a link on the Wiki.
5. **[O]** CO Check that all camera scripts required for this test are present, up-to-date and ready to command the ground support equipment (GSE). These are,
   - **473TEMPR01 - 473TEMPR13**
   - **473TEMPL01 - 473TEMPL13**
   - **441TEMPR03 and 441TEMPL03**
6. **[O,V,D, L]** Notes: All good!

Hardware Installation

This procedure is for the ambient TVAC chamber testing at MSSS. Figure 1 shows the nominal layout of the TVAC chamber, workspace, Mastcam-Zs, ground support equipment (GSE), targets, sources, and other equipment necessary for this test if it happens at ASU. Although MSSS' cleanroom is different than ASU's, the placement of the targets and sources relative to the chamber window is similar.

Figure 1. ASU Floor Plan for Geometric Testing in the TVAC Chamber. The MSSS Floor Plan allows for similar target and source placements relative to the chamber window.

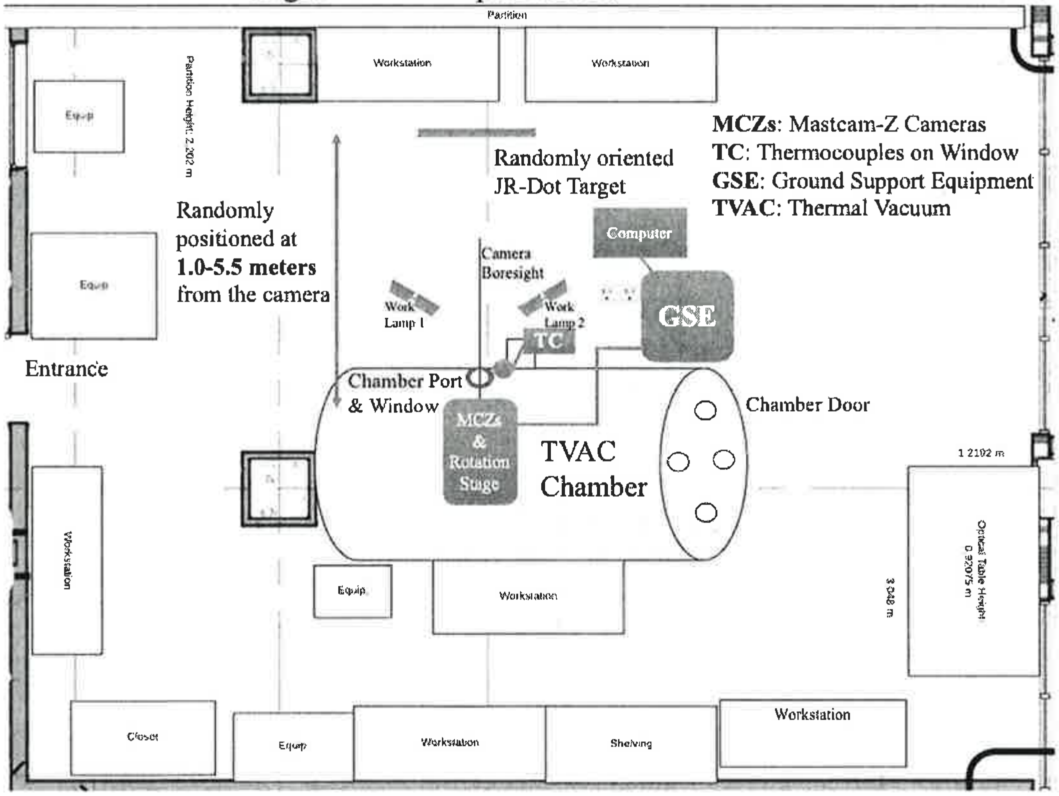

7. **[T,O, L]** ~~____~~ [initials] Ensure that all personnel in the cleanroom are following the cleanroom practices for electrostatic discharge, proper clothing, and other safety concerns. See "ESD_Manual" and "Cleanroom_Manual".
8. **[T]** [initials] Double check ~~that nitrogen~~ *air ionizer on* is flowing over the Mastcam-Zs or the window port.
9. **[O,T]** [initials] If ~~not already~~ *done* done, mate the Right Mastcam-Z into the GSE. Follow the procedure in "MastcamZ_GSE_Manual".
10. **[T]** [initials] Verify that the thermocouples are turned on and properly reading out.

MCZ CH PRT Temp: Right -5.3°C Left -6.4°C

TVAC Chamber: Aux. Plat -10.81°C Main +5.49°C Shroud = -11.92 Cold = -143.68°C

 Date 4/29 Time 10:45 AM Initials [illegible]

11. **[T]** [initials] Position the JR dot target approximately **3 meters** from the cameras 2:07 bar

12. **[T]** [initials] ~~Install the lamps and position them about 1 meter from the geometric target out of the camera's field of view (FOV). Power them on.~~ no lamps

13. **[O,T]** [initials] Ensure that the camera unit and GSE wires are secure, kink-free, and do not present tripping hazards when the lights are turned off. ✓ ok

14. **[[redacted],D]** [initials] Check the camera temperature and ensure nominal operation.

15. **[D]** [initials] Record the following environmental information:
    - Cleanroom temperature 22.8°C pressure earth ambient humidity ~~38%~~ 37% RH

16. **[O,D, L]** Notes:

17. **[[redacted],T, L]** [initials] Capture a test frame with both cameras at 34mm focused at 3 meters with filter 0 to center the target in both cameras. Save images with prefixes **468TEMPR00** and **468TEMPL00**. 4 test images acquired

18. **[D, L]** Notes: positioned target for optimal dot coverages.

10:40 AM "Fog" seen in (autofocus images)
10:45 AM Caplinger wiped the window
10:57 AM ramp started

11:02 AM CH temp: L: -4.6 °C
R: -3.7 °C

Aux. platen: -3.1 °C
main 5.49°
shroud -10.81 °C
cold plate -143.83 °C

Suffixes: 468TEMPR/L 0-4
Suffices: 473TEMPR/L 0-15

Target ~~Metrology~~ setup

19. **[T]** cg Once the target is centered at approximately ~~**3**~~ 2.07 meters **meters** from both cameras, use lidar to measure the target position. ✓
20. **[D]** cg Record the following temperatures:
    - Chamber temp N/A Port temp N/A
    - Camera CCD temp N/A Optics temp N/A
21. **[D,T]** ✓ Take digital pictures of the geometric target's position, and the whole test/GSE set-up. Andy acquired: 2.07 meters from
22. **[M]** cg Measure the locations of the geometric target and the camera.
23. **[M,D]** cg Record the location measurements in the Image Log and tables below.

Target Location	~~Metrology ID#~~
Reference	
Top-Left Nest	
Top-Right Nest	
Bottom-Left Nest	
Bottom-Right Nest	

Camera/Chamber Location	~~Metrology ID#~~
Reference	
Nest 1	
Nest 2	
Nest 3	

24. **[M,D, L]** Notes: no metrology for ramp test

 Date 4/29 Time 10:56 Initials JM

Test Scripts the Right and Left Mastcam-Zs ✓ #1 10:56 AM

25. **[D]** CO Record the following temperatures:
    - Chamber temp ~ -3°C Port temp NA
    - Camera CCD temp R: -3.8 Optics temp L: -2.5
26. **[D,T]** ✓ ~~Take digital pictures of the geometric target's position, and the whole test/GSE set-up.~~ Andy, step 21
27. **[O,T]** ✓ Load and execute script **473TEMPR09**, which captures autofocused frames for three focal lengths with filter 0. Insert the note "TARGET=JR_DOT". The estimated duration is 4 minutes.
28. **[O,T]** ✓ Load and execute script **473TEMPL09**, which captures autofocused frames for three focal lengths with filter 0. Insert the note "TARGET=JR_DOT". The estimated duration is 4 minutes.
29. **[V,O,T]** Evaluate whether the target's dots are in-focus enough for discrimination.
30. **[D]** CO Record image names and parameters in Image Log.
31. **[D, L]** Notes: image suffix: 16 - 27

Temp at Right and Left Mastcam-Zs

#2 11:06 AM

32. **[D]** [illegible] Record the following temperatures:
    - Chamber temp ~1°C Port temp N/A
    - Camera CCD temp R: +1 Optics temp L: +2
33. ~~**[D,T]** ____ Take digital pictures of the geometric target's position, and the whole test/GSE set-up.~~
34. **[O,T]** ✓ Load and execute script **473TEMPR09**, which captures autofocused frames for three focal lengths with filter 0. Insert the note "TARGET=JR_DOT". The estimated duration is 4 minutes.
35. **[O,T]** ✓ Load and execute script **473TEMPL09**, which captures autofocused frames for three focal lengths with filter 0. Insert the note "TARGET=JR_DOT". The estimated duration is 4 minutes.
36. **[V,O,T]** Evaluate whether the target's dots are in-focus enough for discrimination.
37. **[D]** [illegible] Record image names and parameters in Image Log.
38. **[D, L]** Notes: image suffix: 28 - 39

Temp at Right and Left Mastcam-Zs

#3

~11:15AM

39. **[D]** [illegible] Record the following temperatures:
    - Chamber temp ~5°C Port temp N/A
    - Camera CCD temp R: 5.3 Optics temp L: 5.8
40. **[D,T]** ~~Take digital pictures of the geometric target's position, and the whole test/GSE set-up.~~
41. **[O,T]** ✓ Load and execute script **473TEMPR09**, which captures autofocused frames for three focal lengths with filter 0. Insert the note "TARGET=JR_DOT". The estimated duration is 4 minutes.
42. **[O,T]** ✓ Load and execute script **473TEMPL09**, which captures autofocused frames for three focal lengths with filter 0. Insert the note "TARGET=JR_DOT". The estimated duration is 4 minutes.
43. **[V,O,T]** Evaluate whether the target's dots are in-focus enough for discrimination.
44. **[D]** [illegible] Record image names and parameters in Image Log.
45. **[D, L]** Notes: image suffix: 40-51

Temp at Right and Left Mastcam-Zs

#4 ~11:20 AM

46. **[D]** [illegible] Record the following temperatures:
    - Chamber temp ~11°C Port temp N/A
    - Camera CCD temp R: 11.7 Optics temp L: 11.2
47. ~~**[D,T]** ____ Take digital pictures~~ of the geometric target's position, and the whole test/GSE set-up.
48. **[O,T]** ✓ Load and execute script **473TEMPR09**, which captures autofocused frames for three focal lengths with filter 0. Insert the note "TARGET=JR_DOT". The estimated duration is 4 minutes.
49. **[O,T]** ✓ Load and execute script **473TEMPL09**, which captures autofocused frames for three focal lengths with filter 0. Insert the note "TARGET=JR_DOT". The estimated duration is 4 minutes.
50. **[V,O,T]** Evaluate whether the target's dots are in-focus enough for discrimination.
51. **[D]** [illegible] Record image names and parameters in Image Log.
52. **[D, L]** Notes: image suffix: 52 - 63

Temp at Right and Left Mastcam-Zs #5 ~11:30AM

53. **[D]** ✓ Record the following temperatures:
    - Chamber temp ~18°C Port temp N/A
    - Camera CCD temp R: 18.1°C Optics temp L: 17.7 °C
54. **[D,T]** ~~Take digital pictures of the geometric target's position, and the whole test/GSE set-up.~~
55. **[O,T]** ✓ Load and execute script **473TEMPR09**, which captures autofocused frames for three focal lengths with filter 0. Insert the note "TARGET=JR_DOT". The estimated duration is 4 minutes.
56. **[O,T]** ✓ Load and execute script **473TEMPL09**, which captures autofocused frames for three focal lengths with filter 0. Insert the note "TARGET=JR_DOT". The estimated duration is 4 minutes.
57. **[V,O,T]** Evaluate whether the target's dots are in-focus enough for discrimination.
58. **[D]** ✓ Record image names and parameters in Image Log.
59. **[D, L]** Notes: image suffix: 64-75

Date 4/29 Time 11:37 Am Initials [illegible]

#6 ~11:37 AM

Temp at Right and Left Mastcam-Zs

60. **[D]** [illegible] Record the following temperatures:
    - Chamber temp ~26C Port temp [illegible]
    - ~~Camera~~ Right CCD temp 26.1 C ~~Optics~~ Left temp 25.8 °C
61. **[D,T]** ____ ~~Take digital pictures of the geometric target's position, and the whole test/GSE set-up.~~
62. **[O,T]** ✓ Load and execute script **473TEMPR09**, which captures autofocused frames for three focal lengths with filter 0. Insert the note "TARGET=JR_DOT". The estimated duration is 4 minutes.
63. **[O,T]** ✓ Load and execute script **473TEMPL09**, which captures autofocused frames for three focal lengths with filter 0. Insert the note "TARGET=JR_DOT". The estimated duration is 4 minutes.
64. **[V,O,T]** Evaluate whether the target's dots are in-focus enough for discrimination.
65. **[D]** [illegible] Record image names and parameters in Image Log.
66. **[D, L]** Notes: 76-87
    image suffix: 87 both

Date 4/29 Time 11:40AM Initials JM

#7 ~ 11:40 AM

Temp at Right and Left Mastcam-Zs

67. **[D]** ✓ Record the following temperatures:
    - Chamber temp ~30 Port temp ✓
    - Camera CCD temp Left 32.2 ~~Optics~~ Right temp 31.4
68. **[D,T]** ~~____ Take digital pictures of the geometric target's position, and the whole test/GSE set-up.~~
69. **[O,T]** ✓ Load and execute script **473TEMPR09**, which captures autofocused frames for three focal lengths with filter 0. Insert the note "TARGET=JR_DOT". The estimated duration is 4 minutes.
70. **[O,T]** ✓ Load and execute script **473TEMPL09**, which captures autofocused frames for three focal lengths with filter 0. Insert the note "TARGET=JR_DOT". The estimated duration is 4 minutes.
71. **[V,O,T]** Evaluate whether the target's dots are in-focus enough for discrimination.
72. **[D]** ✓ Record image names and parameters in Image Log.
73. **[D, L]** Notes: 88-99
    image suffix: 99 both

Date 4/29 Time 11:50 AM Initials [illegible]

Temp at Right and Left Mastcam-Zs #8

11:50 AM

74. **[D]** ___ Record the following temperatures:
    - Chamber temp N/A Port temp N/A
    - Camera CCD temp N/A Optics temp N/A
75. **[D,T]** ~~___ Take digital pictures of the geometric target's position, and the whole test/GSE set-up.~~
76. **[O,T]** √ Load and execute script **473TEMPR09**, which captures autofocused frames for three focal lengths with filter 0. Insert the note "TARGET=JR_DOT". The estimated duration is 4 minutes.
77. **[O,T]** √ Load and execute script **473TEMPL09**, which captures autofocused frames for three focal lengths with filter 0. Insert the note "TARGET=JR_DOT". The estimated duration is 4 minutes.
78. **[V,O,T]** Evaluate whether the target's dots are in-focus enough for discrimination.
79. **[D]** ___ Record image names and parameters in Image Log.
80. **[D, L]** Notes: image suffix: 111 both

Date 4/21 Time 11:55AM Initials gr

#9 ✓11:55AM

Temp at Right and Left Mastcam-Zs

81. **[D]** CS Record the following temperatures:
    - Chamber temp ~38 °C Port temp N/A
    - Camera CCD temp R: 38.2°C Optics temp L: 36.7°C
82. **[D,T]** ~~Take digital pictures of the geometric target's position, and the whole test/GSE set-up.~~
83. **[O,T]** ✓ Load and execute script **473TEMPR09**, which captures autofocused frames for three focal lengths with filter 0. Insert the note "TARGET=JR_DOT". The estimated duration is 4 minutes.
84. **[O,T]** ✓ Load and execute script **473TEMPL09**, which captures autofocused frames for three focal lengths with filter 0. Insert the note "TARGET=JR_DOT". The estimated duration is 4 minutes.
85. **[V,O,T]** Evaluate whether the target's dots are in-focus enough for discrimination.
86. **[D]** CS Record image names and parameters in Image Log.
87. **[D, L]** Notes:

image suffix: 123 both

Date 4/29 Time 11:59 AM Initials [illegible]

Temp at Right and Left Mastcam-Zs #10 11:59 AM

88. **[D]** [illegible] Record the following temperatures:
    - Chamber temp ~40°C Port temp N/A
    - Camera CCD temp R: 40.0°C Optics temp L: 38.4°C
89. **[D,T]** [illegible] Take digital pictures of the geometric target's position, and the whole test/GSE set-up.
90. **[O,T]** [illegible] Load and execute script **473TEMPR09**, which captures autofocused frames for three focal lengths with filter 0. Insert the note "TARGET=JR_DOT". The estimated duration is 4 minutes.
91. **[O,T]** [illegible] Load and execute script **473TEMPL09**, which captures autofocused frames for three focal lengths with filter 0. Insert the note "TARGET=JR_DOT". The estimated duration is 4 minutes.
92. **[V,O,T]** Evaluate whether the target's dots are in-focus enough for discrimination.
93. **[D]** [illegible] Record image names and parameters in Image Log.
94. **[D, L]** Notes:

image suffix: 135 both

Temp at Right and Left Mastcam-Zs #11

95. **[D]** CO Record the following temperatures:
    - Chamber temp (Aux. platen) 39.35°C Port temp N/A
    - Camera CCD temp " Optics temp N/A
96. **[D,T]** CO Take digital pictures of the geometric target's position, and the whole test/GSE set-up.
97. **[O,T]** CO Load and execute script **473TEMPR09**, which captures autofocused frames for three focal lengths with filter 0. Insert the note "TARGET=JR_DOT". The estimated duration is 4 minutes.
98. **[O,T]** [illegible] Load and execute script **473TEMPL09**, which captures autofocused frames for three focal lengths with filter 0. Insert the note "TARGET=JR_DOT". The estimated duration is 4 minutes.
99. **[V,O,T]** Evaluate whether the target's dots are in-focus enough for discrimination.
100. **[D]** [illegible] Record image names and parameters in Image Log.
101. **[D, L]** Notes: image suffix: 157 both

 Date 4/29 Time 12:17 pm Initials [illegible]

Temp at Right and Left Mastcam-Zs #12

102. **[D]** ___ Record the following temperatures:
    - Chamber temp ~40 °C Port temp N/A
    - Camera CCD temp R: 41.8° C Optics temp L: 40.3 °C
103. **[D,T]** ___ Take digital pictures of the geometric target's position, and the whole test/GSE set-up.
104. **[O,T]** ___ Load and execute script **473TEMPR09**, which captures autofocused frames for three focal lengths with filter 0. Insert the note "TARGET=JR_DOT". The estimated duration is 4 minutes.
105. **[O,T]** ___ Load and execute script **473TEMPL09**, which captures autofocused frames for three focal lengths with filter 0. Insert the note "TARGET=JR_DOT". The estimated duration is 4 minutes.
106. **[V,O,T]** Evaluate whether the target's dots are in-focus enough for discrimination.
107. **[D]** ___ Record image names and parameters in Image Log.
108. **[D, L]** Notes:____

____

____

Temp at Right and Left Mastcam-Zs #13

109. **[D]** CS Record the following temperatures:
   - Chamber temp aux platen: 38.58°C Port temp N/A
   - Camera CCD temp 42.4°C Optics temp 40.8 °C
110. **[D,T]** CX Take digital pictures of the geometric target's position, and the whole test/GSE set-up.
111. **[O,T]** ✓ Load and execute script **473TEMPR09**, which captures autofocused frames for three focal lengths with filter 0. Insert the note "TARGET=JR_DOT". The estimated duration is 4 minutes.
112. **[O,T]** ✓ Load and execute script **473TEMPL09**, which captures autofocused frames for three focal lengths with filter 0. Insert the note "TARGET=JR_DOT". The estimated duration is 4 minutes.
113. **[V,O,T]** Evaluate whether the target's dots are in-focus enough for discrimination.
114. **[D]** CS Record image names and parameters in Image Log.
115. **[D, L]** Notes: image suffix: 171

Date 4/29 Time 12:33 pm Initials gv

Temp at Right and Left Mastcam-Zs #14

116. **[D]** CS Record the following temperatures:
  - Chamber temp aux. plate 38.7 °C Port temp N/A
  - Camera CCD temp " Optics temp N/A

117. ~~**[D,T]** ____ Take digital pictures of the geometric target's position, and the whole test/GSE set-up.~~

118. **[O,T]** CS Load and execute script **473TEMPR09**, which captures autofocused frames for three focal lengths with filter 0. Insert the note "TARGET=JR_DOT". The estimated duration is 4 minutes.

119. **[O,T]** CS Load and execute script **473TEMPL09**, which captures autofocused frames for three focal lengths with filter 0. Insert the note "TARGET=JR_DOT". The estimated duration is 4 minutes. CS

120. **[V,[redacted],T]** Evaluate whether the target's dots are in-focus enough for discrimination.

121. **[D]** CS Record image names and parameters in Image Log.

122. **[D, L]** Notes:

R 172 ← aborted/terminated early ~12:38 pm

L 175

↳ Tex reports that the camera has returned a mechanism health flag. He ~~believes~~ says that this appears to be a known issue ~~so~~ and will confer with Caplinger.

→ Focus mech position at 0, backlash adds uncertainty, throws a mech fault homed mechanism, status flag clear continue, all nominal

Date 4/29 Time 12:54pm Initials gm

Temp at Right and Left Mastcam-Zs #15

123. **[D]** CK Record the following temperatures:
   - Chamber temp ~43 Port temp N/A
   - Camera CCD temp 43.2 Optics temp 41.3
124. **[D,T]** CK ~~Take digital pictures of the geometric target's position, and the whole test/GSE set-up.~~
125. **[O,T]** ✓ Load and execute script **473TEMPR09**, which captures autofocused frames for three focal lengths with filter 0. Insert the note "TARGET=JR_DOT". The estimated duration is 4 minutes.
126. **[O,T]** ✓ Load and execute script **473TEMPL09**, which captures autofocused frames for three focal lengths with filter 0. Insert the note "TARGET=JR_DOT". The estimated duration is 4 minutes.
127. **[V,O,T]** CB Evaluate whether the target's dots are in-focus enough for discrimination.
128. **[D]** CB Record image names and parameters in Image Log.
129. **[D, L]** Notes:
   R: 184
   L 187

declare Ramp complete

Temp at Right and Left Mastcam-Zs

130. **[D]** ____ Record the following temperatures:
    - Chamber temp _______________ Port temp _______________
    - Camera CCD temp _______________ Optics temp _______________
131. **[D,T]** ____ Take digital pictures of the geometric target's position, and the whole test/GSE set-up.
132. **[O,T]** ____ Load and execute script **473TEMPR09**, which captures autofocused frames for three focal lengths with filter 0. Insert the note "TARGET=JR_DOT". The estimated duration is 4 minutes.
133. **[O,T]** ____ Load and execute script **473TEMPL09**, which captures autofocused frames for three focal lengths with filter 0. Insert the note "TARGET=JR_DOT". The estimated duration is 4 minutes.
134. **[V,O,T]** Evaluate whether the target's dots are in-focus enough for discrimination.
135. **[D]** ____ Record image names and parameters in Image Log.
136. **[D, L]** Notes:____

____

____

skip

Temp at Right and Left Mastcam-Zs

137. **[D]** ____ Record the following temperatures:
    - Chamber temp _______________ Port temp _______________
    - Camera CCD temp _______________ Optics temp _______________
138. **[D,T]** ____ Take digital pictures of the geometric target's position, and the whole test/GSE set-up.
139. **[O,T]** ____ Load and execute script **473TEMPR09**, which captures autofocused frames for three focal lengths with filter 0. Insert the note "TARGET=JR_DOT". The estimated duration is 4 minutes.
140. **[O,T]** ____ Load and execute script **473TEMPL09**, which captures autofocused frames for three focal lengths with filter 0. Insert the note "TARGET=JR_DOT". The estimated duration is 4 minutes.
141. **[V,O,T]** Evaluate whether the target's dots are in-focus enough for discrimination.
142. **[D]** ____ Record image names and parameters in Image Log.
143. **[D, L]** Notes:____
    ____
    ____

skip

Temp at Right and Left Mastcam-Zs

144. **[D]** ____ Record the following temperatures:
    - Chamber temp _______________ Port temp _______________
    - Camera CCD temp _______________ Optics temp _______________
145. **[D,T]** ____ Take digital pictures of the geometric target's position, and the whole test/GSE set-up.
146. **[O,T]** ____ Load and execute script **473TEMPR09**, which captures autofocused frames for three focal lengths with filter 0. Insert the note "TARGET=JR_DOT". The estimated duration is 4 minutes.
147. **[O,T]** ____ Load and execute script **473TEMPL09**, which captures autofocused frames for three focal lengths with filter 0. Insert the note "TARGET=JR_DOT". The estimated duration is 4 minutes.
148. **[V,O,T]** Evaluate whether the target's dots are in-focus enough for discrimination.
149. **[D]** ____ Record image names and parameters in Image Log.
150. **[D, L]** Notes:____

____

____

Skip

Temp at Right and Left Mastcam-Zs

151. **[D]** ____ Record the following temperatures:
   - Chamber temp _______________ Port temp _______________
   - Camera CCD temp _______________ Optics temp _______________
152. **[D,T]** ____ Take digital pictures of the geometric target's position, and the whole test/GSE set-up.
153. **[O,T]** ____ Load and execute script **473TEMPR09**, which captures autofocused frames for three focal lengths with filter 0. Insert the note "TARGET=JR_DOT". The estimated duration is 4 minutes.
154. **[O,T]** ____ Load and execute script **473TEMPL09**, which captures autofocused frames for three focal lengths with filter 0. Insert the note "TARGET=JR_DOT". The estimated duration is 4 minutes.
155. **[V,O,T]** Evaluate whether the target's dots are in-focus enough for discrimination.
156. **[D]** ____ Record image names and parameters in Image Log.
157. **[D, L]** Notes:____

____

____

skip

Temp at Right and Left Mastcam-Zs

158. **[D]** ____ Record the following temperatures:
    - Chamber temp _______________ Port temp _______________
    - Camera CCD temp _______________ Optics temp _______________
159. **[D,T]** ____ Take digital pictures of the geometric target's position, and the whole test/GSE set-up.
160. **[O,T]** ____ Load and execute script **473TEMPR09**, which captures autofocused frames for three focal lengths with filter 0. Insert the note "TARGET=JR_DOT". The estimated duration is 4 minutes.
161. **[O,T]** ____ Load and execute script **473TEMPL09**, which captures autofocused frames for three focal lengths with filter 0. Insert the note "TARGET=JR_DOT". The estimated duration is 4 minutes.
162. **[V,O,T]** Evaluate whether the target's dots are in-focus enough for discrimination.
163. **[D]** ____ Record image names and parameters in Image Log.
164. **[D, L]** Notes:____
____
____

skip

Temp at Right and Left Mastcam-Zs

165. **[D]** ____ Record the following temperatures:
   - Chamber temp ______________ Port temp ______________
   - Camera CCD temp ______________ Optics temp ______________
166. **[D,T]** ____ Take digital pictures of the geometric target's position, and the whole test/GSE set-up.
167. **[O,T]** ____ Load and execute script **473TEMPR09**, which captures autofocused frames for three focal lengths with filter 0. Insert the note "TARGET=JR_DOT". The estimated duration is 4 minutes.
168. **[O,T]** ____ Load and execute script **473TEMPL09**, which captures autofocused frames for three focal lengths with filter 0. Insert the note "TARGET=JR_DOT". The estimated duration is 4 minutes.
169. **[V,O,T]** Evaluate whether the target's dots are in-focus enough for discrimination.
170. **[D]** ____ Record image names and parameters in Image Log.
171. **[D, L]** Notes:____
____
____

skip

Temp at Right and Left Mastcam-Zs

172. **[D]** ____ Record the following temperatures:
    - Chamber temp _______________ Port temp _______________
    - Camera CCD temp _______________ Optics temp _______________
173. **[D,T]** ____ Take digital pictures of the geometric target's position, and the whole test/GSE set-up.
174. **[O,T]** ____ Load and execute script **473TEMPR09**, which captures autofocused frames for three focal lengths with filter 0. Insert the note "TARGET=JR_DOT". The estimated duration is 4 minutes.
175. **[O,T]** ____ Load and execute script **473TEMPL09**, which captures autofocused frames for three focal lengths with filter 0. Insert the note "TARGET=JR_DOT". The estimated duration is 4 minutes.
176. **[V,O,T]** Evaluate whether the target's dots are in-focus enough for discrimination.
177. **[D]** ____ Record image names and parameters in Image Log.
178. **[D, L]** Notes:____

____

____

skip

Temp at Right and Left Mastcam-Zs

179. **[D]** ____ Record the following temperatures:
    - Chamber temp ______________ Port temp ______________
    - Camera CCD temp ______________ Optics temp ______________
180. **[D,T]** ____ Take digital pictures of the geometric target's position, and the whole test/GSE set-up.
181. **[O,T]** ____ Load and execute script **473TEMPR09**, which captures autofocused frames for three focal lengths with filter 0. Insert the note "TARGET=JR_DOT". The estimated duration is 4 minutes.
182. **[O,T]** ____ Load and execute script **473TEMPL09**, which captures autofocused frames for three focal lengths with filter 0. Insert the note "TARGET=JR_DOT". The estimated duration is 4 minutes.
183. **[V,O,T]** Evaluate whether the target's dots are in-focus enough for discrimination.
184. **[D]** ____ Record image names and parameters in Image Log.
185. **[D, L]** Notes:____
____
____

skip

Temp at Right and Left Mastcam-Zs

186. **[D]** ____ Record the following temperatures:
    - Chamber temp ______________ Port temp ______________
    - Camera CCD temp ______________ Optics temp ______________
187. **[D,T]** ____ Take digital pictures of the geometric target's position, and the whole test/GSE set-up.
188. **[O,T]** ____ Load and execute script **473TEMPR09**, which captures autofocused frames for three focal lengths with filter 0. Insert the note "TARGET=JR_DOT". The estimated duration is 4 minutes.
189. **[O,T]** ____ Load and execute script **473TEMPL09**, which captures autofocused frames for three focal lengths with filter 0. Insert the note "TARGET=JR_DOT". The estimated duration is 4 minutes.
190. **[V,O,T]** Evaluate whether the target's dots are in-focus enough for discrimination.
191. **[D]** ____ Record image names and parameters in Image Log.
192. **[D, L]** Notes:____

____

____

skip

Date 4/29 Time 13:08 Initials [illegible]

Target distance 2.06 m ✓ unchanged

Data Validation

193. **[V]** ✓ Run the "Geometric_46_Validation" Jupyter notebook on the acquired data for the Right and Left Mastcam-Zs. This analysis can take place while the test continues.

194. **[V,D, L]** Notes: Paul is running a variant of this analysis software
Paul reports data are nominal

end of ramp test

Test image: 44

Date 4/29 Time 13:14 Initials [illegible]

Dark Current with the Right and Left Mastcam-Zs

195. **[T]** ✓ Cover the port window and turn off the lights.
196. **[D]** ____ Record the following temperatures:
    - Chamber temp ____________ Port temp ____________
    - Camera CCD temp R= 43.0 °C ~~Optics~~ temp L= 41.3 °C
197. **[D,T]** ✓ Take digital pictures of the ~~geometric target's position~~ setup, and the whole test/GSE set-up.
198. ✓ **[O]** Load and execute camera script **441TEMPR03**, which captures 5 dark frames through filter 7 at the exposure times 0.0, 1.0, 2.0, and 100 seconds. The estimated duration is 9 minute. 0 10. 20.0 100.0
199. ✓ **[O]** Load and execute camera script **441TEMPL03**, which captures 5 dark frames through filter 7 at the exposure times 0.0, 1.0, 2.0, and 100 seconds. The estimated duration is 9 minute. 0, 10.0 20.0, 100.0
200. **[D]** ____ Record image names and parameters in Image Log.
201. **[D, L]** Notes: img suffix: 66

Data Validation

202. **[V]** ✓ Run the "Dark_41_Validation" Jupyter notebook on the acquired data for the Right and Left Mastcam-Zs. This analysis can take place while the test continues.

203. **[V,D, L]** Notes: ok per paul

Date 4/29 Time 13:30 Initials [illegible]

Shutdown Procedure

204. **[D,T]** ✓ Take digital pictures of this page and the test setup.
205. **[D,O]** [illegible] Review entries in Image Log, GSE command log, and image headers.
206. **[D, L]** [illegible] Review calibration procedure and ensure that each task is initialed.
207. **[D, L]** Notes: ______

208. **[V, L]** ✓ Before making the decision to break down the test setup, ensure that adequate data were acquired for the test requirements. See "MastcamZCalPlan" for these requirements.
209. **[V]** Notes: ok per Paul analysis

Data Validator (signature) [illegible]

Date 4-29-19 Time 1:54 PM

210. **[V, L]** ✓ Give the go/no-go decision. Have enough data been acquired to fulfill test requirements? See "MastcamZCalPlan" for these requirements.
211. **[D, L]** [illegible] ~~Update the Log Document~~. procedure is the log
212. **[L]** Notes: ______

Calibration Lead (signature) [illegible]

Date 4/29/19 Time 13:55

213. **[O, L]** ✓ Ensure that the camera and GSE are in a safe state. off & safe per Tex
214. **[[redacted],D]** ✓ Review the Image Log with the documentarian. Exchange high-fives.
215. **[[redacted]]** Notes: high fives were exchanged

Camera Operator (signature) [signature]

Date 4/29/19 Time 13:56

216. **[T]** ✓ If the next test does not require the target, position it away from the chamber or bench. Otherwise, be sure not to move it. The next test is radiometric cal
Andy moved target
217. **[T]** ✓ Ensure that all other test equipment is safely put away.
218. **[T]** Notes:

Technician (signature) [signature]

Date 4/29/19 Time 13:58

219. **[D, L]** [initials] Double-check this procedure and ensure that the top of each page has valid data, time and initials.
220. **[D]** [initials] Photo-scan this document, save it on the cloud, and file the hard-copy in the Log Binder. Upload the digital pictures taken during this test in the appropriate archive on the cloud. The required links are on the Wiki.
221. **[D]** [initials] Double-check that every required cell the Image Log is accurately filled. When this is complete, print the Image Log and file it the Log Binder after this document.
222. **[D]** Notes:

Documentarian (signature) Christian [signature]

Date 4-29-19 Time 15:08
