# Supplementary material for: Pre-Flight Calibration of the Mars 2020 Rover Mastcam Zoom (Mastcam-Z) Multispectral, Stereoscopic Imager
Source: Space Sci Rev. 2021 Feb 18;217(2):29. doi: 10.1007/s11214-021-00795-x (PMC7892537; doi:10.1007/s11214-021-00795-x)
Supplement: Supplementary file 1 — (ZIP 98.6 MB) [file 11214_2021_795_MOESM1_ESM.zip › CalPro_471_MTF_v2_06.pdf]

Date 4/30 Time 8:30 Initials [illegible]

MTF Calibration Procedure for Mastcam-Z Ambient TVAC Testing (Pro. 4.7.1)

[Procedure version 2.06, prepared by the Mastcam-Z calibration team at Cornell University.]

These measurements are performed on the camera and at the temperature designated below as specified in the Calibration Plan (Document #),

Unit Under Test:

R FM X **L FM** X **EQM**____ **Other**____

Test Performed at Temperature:

-35°C____ **- 10°C**____ **+5°C**____ **Ambient** X **Other** ____

These measurements are performed at,

MSSS X **ASU**____ **Other**____

Date 4/30/19 **Start Time** 8:30 AM **End Time** 15:00

Estimated Duration 4.0 hours

Scheduled Start Time 8:30 AM **Sch. End Time** 12:30

Calibration Lead [L] Maki, Justin **Documentarian [D]** Christian Tate

Camera Operator [O] Tex, Elsa **Technician [T]** Andy Winhold

Data Validator [V] Paul Corlies **Metrologist [M]** N/A

Other ____

4/30
Date ~~5/30~~ Time 9:04 Initials gm

Change Log

Version	Name	Change
v1_01<br>1 Oct 2018	C. Tate	(first draft)
v2_03<br>30 April 2019	C. Tate	Approved version prior to FM testing

Document Approval

Approved by James Bell — Date
Mastcam-Z PI
Arizona State University

5/6/19
Approved by Alexander Hayes — Date
Mastcam-Z Calibration Working Group Lead, Cornell University

4/30/19
Approved by Justin Maki — Date
Mastcam-Z Deputy PI and Investigation Scientist, Jet Propulsion Laboratory

Christian Tate 4/30/19
Approved by Christian Tate — Date
Procedure Author
Cornell University

Approved by: — Date

Date 5/30 Time 9:04 Initials [illegible]

Table of Contents

MTF CALIBRATION PROCEDURE FOR MASTCAM-Z AMBIENT TVAC TESTING (PRO. 4.7.1) 1

CHANGE LOG 2
DOCUMENT APPROVAL 2
TEST DESCRIPTION 3
SOFTWARE PREPARATION 4
Table 1. File naming convention for the camera script prefixes and frame filenames: "AAABBBBCDD" 4
HARDWARE INSTALLATION 5
Table 2. The nominal target placement scenes for the geometric testing 7
SCENE 3 FOR THE LEFT MASTCAM-Z 8
SCENE 3 FOR THE RIGHT MASTCAM-Z 9
SCENE 1 FOR THE LEFT MASTCAM-Z 10
SCENE 1 FOR THE RIGHT MASTCAM-Z 11
SCENE 2 FOR THE LEFT MASTCAM-Z 12
SCENE 2 FOR THE RIGHT MASTCAM-Z 13
TIME CHECK 1 14
SCENE 3 Z-STACK FOR THE LEFT MASTCAM-Z 15
SCENE 3 Z-STACK FOR THE RIGHT MASTCAM-Z 16

SHUTDOWN PROCEDURE 17

Test Description

Excerpt from the Calibration Plan 4.7

The objective of this test is to image well-characterized bar targets at multiple focus and zoom positions in order to characterize the Modulation Transfer Function (MTF) and depth of field of each camera. Targets should be imaged at ~50% full well using the Bayer RGB (priority 1), 805 nm, (priority 2), and remaining non-solar filters (priority 3). Obtain a minimum of 3 images of each target per filter, focus, and zoom position. Multiple images are needed to reduce errors in determining target locations in the image plane.

MTF is an effective means of specifying the resolution of an optical system. Resolution is defined as the minimum feature size of an object that can be distinguished by an imaging system. The Point Spread Function (PSF) is the inverse Fourier Transform of the MTF—the PSF describes optical performance in the spatial domain while the MTF expresses optical performance in the frequency domain. Images of the bar targets and knife edge or point source targets at various zoom and focus positions will be used to determine PSF, depth of field, and MTF. The bar target shall consist of a chart containing horizontal, vertical, and diagonal lines and bars of varying thicknesses as well as circular dots of various sub- and super-pixel sizes.

In addition to determining the optical performance of the optomechanical assemblies, the images collected during MTF/PSF calibration will also be used to determine the numerical value and repeatability of the stepper motor counts for the Hall Effect sensors used to measure the position of the focus group and two moving zoom groups in the optical zoom assemblies. This will determine the relationship between stepper motor count for each focus/zoom group, working distance, and pixel scale. Owing to thickness variations between spectral filters, focus shifts may occur and images would ideally be obtained using all non-solar filters.

Note that the Mastcam-Z instrument has an onboard focus merge algorithm that selects and merges the best-focus portions of a scene using a focus stack (or z-stack) of multiple images (up to 16) at varying focal positions. While usually only the best-focus or merged product is saved to file, the raw frames for the focus stack can also be saved to file without appreciably increasing the observation or product generation time. For tests where images at multiple focus positions are desired, such as during MTF/PSF Calibrations, the entire focus stack of images will be written to file. Since images at multiple focus positions are acquired irrespective of whether or not they are written to memory, saving multiple focus positions per zoom setting does not require additional test time (outside of the time required to write the files to disk in the GSE). Multiple focus position images shall be saved during both Stand-Alone and ATLO MTF/PSF testing.

Software Preparation

The software and files required for this test are prepared well in advance of test day. This checklist ensures that the following are present, debugged, and executable: (1) all fast look scripts, (2) automated header generation of all relevant camera parameters, target positioning, and metadata, (3) all camera scripts that command the camera unit, and (4) the directories/file-paths pointing to the data repositories of this specific test.

Table 1. File naming convention for the camera script prefixes and frame filenames: "AAABBBBCDD"

Code	Name	Example	Value(s)
"AAA"	Calibration Plan Section	"411" = Cal. Plan 4.1.1 chapter 4, section 1, subsection 1	471, 491
"BBBB"	Location of test or ASU TVAC temperature	"MSSS" = test at MSSS, "ATLO" = test at JPL ATLO, "TN10" = ASU TVAC -10C, …	TAMB
"C"	Camera unit under test	"L" = Left Mastcam-Z, "R" = Right Mastcam-Z, "E" =EQM, "C" =COTS	R/L
"DD"	Part of test	"00" = test set up, "01" = first radiance level …	00-12

Date 4/30 Time 9:05 Initials JR

1. **[D]** N/A Look up the daily calibration schedule and record the scheduled start and end time of this test on the cover page of this document. Also, fill out and double-check the other information on the cover page.
2. **[D]** ✓ Ensure that all supplemental manuals are on hand. These are,
   - Validator_Manual, Documentarian_Manual,
   - MastcamZCalPlan
3. **[D]** ✓ Ensure that the Image Log is present and ready to use. Find and open the Google Sheets file "Image_Log_46". The duration is 2 minutes. There is a link on the Wiki.
4. **[V]** ✓ Check that all *Calgorithms* fast-look and validation scripts are present, up-to-date, and ready to analyze test output. Find and open the "Geometric_Calibration_47_Validation" Jupyter notebook. There is a link on the Wiki.
5. **[O]** ✓ Check that all camera scripts required for this test are present, up-to-date and ready to command the ground support equipment (GSE). These are,
   - **471TAMBR00 - 471TAMBR13, 491TAMBR00 - 491TAMBR12**
   - **471TAMBL00 - 471TAMBL13, 491TAMBL00 - 491TAMBL12**
6. **[O,V,D, L]** Notes: scripts confirmed

Hardware Installation

This procedure is for the ambient TVAC chamber testing at MSSS. Figure 1 shows the nominal layout of the TVAC chamber, workspace, Mastcam-Zs, ground support equipment (GSE), targets, sources, and other equipment necessary for this test if it happens at ASU. Although MSSS' cleanroom is different than ASU's, the placement of the targets and sources relative to the chamber window is similar.

Date ______ Time ______ Initials ______

MSSS see photos

Figure 1. A~~S~~U Floor Plan for Geometric Testing in the TVAC Chamber. The MSSS Floor Plan allows for similar target and source placements relative to the chamber window.

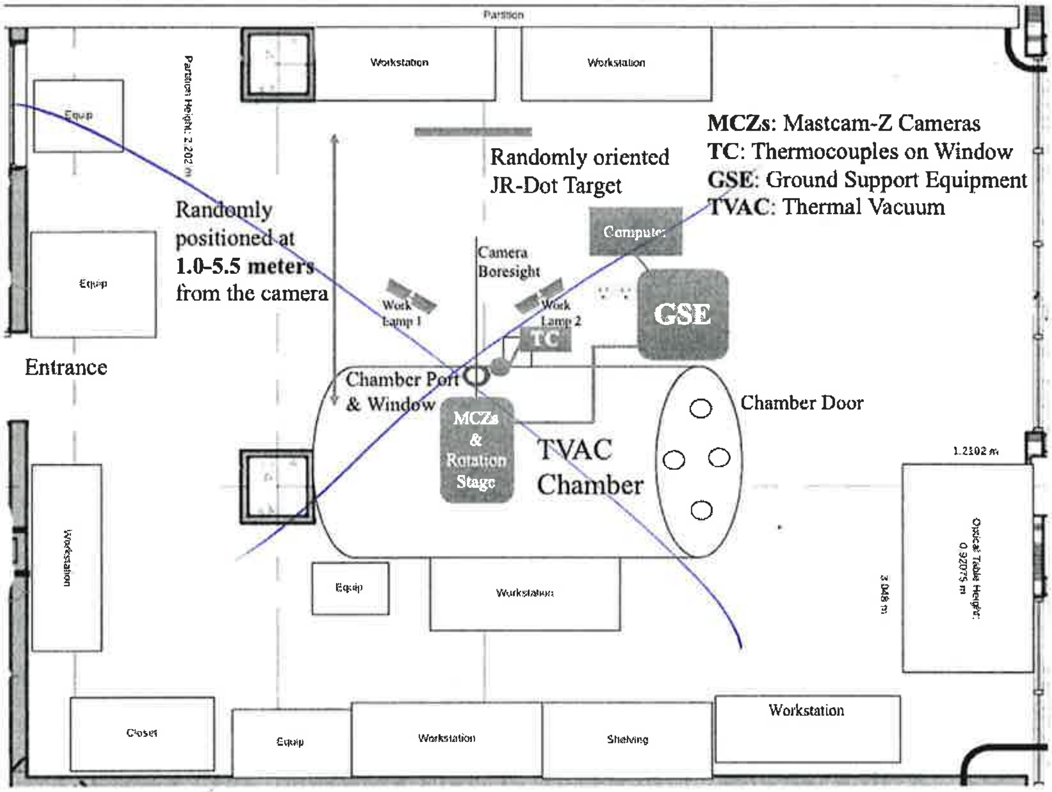

7. **[T,O, L]** _✓_ Ensure that all personnel in the cleanroom are following the cleanroom practices for electrostatic discharge, proper clothing, and other safety concerns. See "ESD_Manual" and "Cleanroom_Manual". The duration is 2 minutes.

8. **[T]** ____ ~~Double check that nitrogen is flowing over the Mastcam-Zs or the window port.~~ skip, not required used air ionizer

9. **[[redacted],T]** ____ ~~If not already done, mate the Right Mastcam-Z into the GSE.~~ done already

10. **[T]** _✓_ Verify that the thermocouples are turned on and properly reading out.

11. **[T]** _✓_ Install the lamps and position them about 1 meter from the geometric target out of the camera's field of view (FOV). Power them on.

12. **[O,T]** _✓_ Ensure that the camera unit and GSE wires are secure, kink-free, and do not present tripping hazards when the lights are turned off.

13. **[O,D]** _✓_ Check the camera temperature and ensure nominal operation. L: 25.6°C R: 26.5 C

14. **[D]** _✓_ Record the following environmental information:

- Cleanroom temperature ~~26.~~ 25.6° C pressure atm. humidity 48% RH

Date 4/30 Time 10:06 Initials [illegible]

15. **[O,D, L]** Notes: NA

Table 2. The nominal target placement scenes for the geometric testing.

	**Target Placements**	**Notes**
**Scene 1**	Small MTF_SN005 (Side A) target at about 2 meters centered on the 63mm boresight	
**Scene 2**	Small MTF_SN005 (Side A) target at about 3 meters centered on the 100mm boresight	
**Scene 3**	Large MTF_SN007 (Side A) target at about 3 meters centered on the 34mm boresight	
**Scene 4**	Large MTF_SN007 (Side A) target at about 2 meters centered on the 26mm boresight	

Date 4/30 Time 10:08 Initials JM

Scene 3 for the Left Mastcam-Z

SN 007

16. **[M,T]** Position the MTF target to Scene 3 as described in Table 2.
17. **[M]** Measure and record the location of the MTF target.

distance = 2.5 meters

18. **[D]** Record climate information:
    - TVAC temp 2 Port temp N/A
    - Camera CCD temp L 25.6° C ~~Optics~~ temp R 26.5°C
19. **[D,T]** Take digital pictures of the setup and MTF target. ✓
20. **[O]** Capture a tests frame at 34mm with filter 0, and rsync data to the validator. Use prefix **471TAMBL00.** ✓
21. **[V,T]** Load the image in MTF Mapper to find the correct target position. Recapture an autofocused frame if necessary. ✓
22. [[redacted]] Load and execute camera script **491TAMBL05**, which autofocuses and captures 3 frames at 34mm focal length with filters 0 through 6. Insert note "TARGET=MTF_SN007". The estimated duration is 8 minutes. 10:18 AM
23. **[D]** Record image names and parameters in Image Log. ✓
24. **[V]** Run fast-look script to verify that the required data were obtained. ✓
25. **[D, L]** Notes:

2 autos, + 2 manuals,
filter 3,4,5 shows faint contrast in center slanted edges

Test image: 9:41 AM, suffix 0 → reacquired with different autoexposure settings

O_2 sensor: 19.8 %

Temperature: 29.2°
final suffix: 27

Was: DN thres: 180
pix frac. 10%

Is: DN thres: 150
pix frac: 1%

final: 55 → still seeing MTF dropout in center of image, but appear minor.

Date 4/30 Time 10:08 Initials [illegible]

Scene 3 for the Right Mastcam-Z SN 007

26. **[M,T]** Position the MTF target to Scene 3 as described in Table 2.
27. **[M]** Measure and record the location of the MTF target.
test image, suffix 0
distance = 2.7 meter

28. **[D]** Record climate information:
    - ~~TVAC~~ temp Room: 23.9°C ~~Port temp~~ RH % 51%
    - Camera ~~CCD temp~~ L: 29.3°C ~~Optic~~s temp R: 29.3°C
29. **[D,T]** Take digital pictures of the setup and MTF target.
30. ✓ **[O]** Capture a tests frame at 34mm with filter 0, and rsync data to the validator. Use prefix **471TAMBR00.** 10:59 [illegible]
31. ✓ **[V,T]** Load the image in MTF Mapper to find the correct target position. Recapture an autofocused frame if necessary.
32. ✓ **[O]** Load and execute camera script **491TAMBR05**, which autofocuses and captures 3 frames at 34mm focal length with filters 0 through 6. Insert note "TARGET=MTF_SN007". The estimated duration is 8 minutes. → with autoexpose mode
33. ✓ **[D]** Record image names and parameters in Image Log.
34. ✓ **[V]** Run fast-look script to verify that the required data were obtained.
35. **[D, L]** Notes:

filter 6 autofocus failed (out of range)
reacquiring filter 6 only (suffix = 31)

Date 4/30 Time 11:54 Am Initials [illegible]

Scene 1 for the ~~Left~~ Right Mastcam-Z SN 005

36. **[M,T]** Position the MTF target to Scene 1 as described in Table 2.
37. **[M]** Measure and record the location of the MTF target.
    distance = 1.4 meters
    1.25 m.
    2.1 meters from camera
38. **[D]** Record climate information:
    - ~~TVAC~~ Room temp 24.6 °C ~~Port temp~~ Room RH 49 %
    - Camera CCD temp R = 30.3 °C Optics ~~temp~~ N/A
39. ✓ **[D,T]** Take digital pictures of the setup and MTF target.
40. ✓ **[O]** Capture a tests frame at 63mm with filter 0, and rsync data to the validator. Use prefix **471TAMBL00.**
41. ✓ **[V,T]** Load the image in MTF Mapper to find the correct target position. Recapture an autofocused frame if necessary.
42. ✓ [[redacted]] Load and execute camera script **491TAMB~~L~~R06**, which autofocuses and captures 3 frames at 63mm focal length with filters 0 through 6. Insert note "TARGET=MTF_SN005". The estimated duration is 8 minutes.
43. ✓ **[V]** Run fast-look script ~~to~~ verify that the required data were obtained.
44. **[D, L]** Notes: validated

Time check: 12:08 pm

3 scenes out of 8

2 hours for 3 scenes

Date 4/30 Time 12:20pm Initials [illegible]

Scene 1 for the ~~Right~~ Left Mastcam-Z SN 005

45. **[M,T]** Position the MTF target to Scene 1 as described in Table 2.
46. **[M]** Measure and record the location of the MTF target.
    target dst= 2.01 m
47. **[D]** Record climate information:
    - ~~TVAC~~ Room temp 25.0 °C ~~Port temp~~ Room RH 46%
    - Camera C~~CD temp~~ L=29.7 °C ~~Optics temp~~ N/A
48. ✓ **[D,T]** Take digital pictures of the setup and MTF target.
49. ✓ **[O]** Capture a tests frame at 63mm with filter 0, and rsync data to the validator. Use prefix **471TAMB~~R~~L00.**
50. ✓ **[V,T]** Load the image in MTF Mapper to find the correct target position. Recapture an autofocused frame if necessary.
51. ✓ **[O]** Load and execute camera script **491TAMB~~R~~L06**, which autofocuses and captures 3 frames at 63mm focal length with filters 0 through 6. Insert note "TARGET=MTF_SN005". The estimated duration is 8 minutes.
52. ✓ **[V]** Run fast-look script to verify that the required data were obtained.
53. ✓ **[D, L]** Notes: Validated

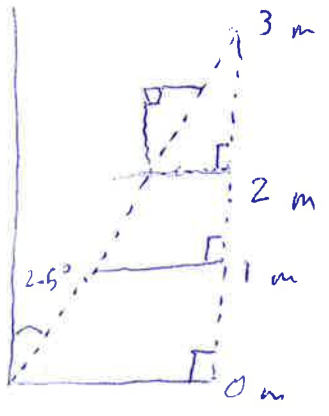

Scene 2 for the Left Mastcam-Z

54. [M,T] Position the MTF target to Scene 2 as described in Table 2.
55. [M] Measure and record the location of the MTF target.
    distance = 3.3 meters
56. ✓ [D] Record climate information: RH = 46 %
    - ~~TVAC~~ Room temp 25.0 °C ~~Port temp~~ ______
    - Camera CCD temp 29.8 °C Optics temp N/A
57. ✓ [D,T] Take digital pictures of the setup and MTF target.
58. ✓ [O] Capture a tests frame at 100mm with filter 0, and rsync data to the validator. Use prefix ~~471~~ 491 **TAMBL00.**
59. ✓ [V,T] Load the image in MTF Mapper to find the correct target position. Recapture an autofocused frame if necessary.
60. ✓ [O] Load and execute camera script ~~4791~~ 491 **TAMBL07**, which autofocuses and captures 3 frames at 100mm focal length with filters 0 through 6. Insert note "TARGET=MTF_SN005". The estimated duration is 8 minutes.
61. [D] Record image names and parameters in Image Log.
62. [V] Run fast-look script to verify that the required data were obtained.
63. [D, L] Notes:
    filter 6 autofocus fail (432 nm)
    filter 6 " "

success suffix: 39

Date 4/30 Time 13:16 Initials [illegible]

Scene 2 for the Right Mastcam-Z

64. ✓ **[M,T]** Position the MTF target to Scene 2 as described in Table 2.
65. **[M]** Measure and record the location of the MTF target.
    test image (13) 3.3 meters
66. ✓ **[D]** Record climate information:
    - ~~TVAC~~ Room temp 24.8°C ~~Port temp~~ RH 46%
    - ~~Camera CCD~~ R temp 30.2°C Optics temp N/A
67. ✓ **[D,T]** Take digital pictures of the setup and MTF target.
68. ✓ **[O]** Capture a tests frame at 100mm with filter 0, and rsync data to the validator. Use prefix **471TAMBR00.**
69. ✓ **[V,T]** Load the image in MTF Mapper to find the correct target position. Recapture an autofocused frame if necessary.
70. ✓ **[O]** Load and execute camera script **491TAMBR07**, which autofocuses and captures 3 frames at 100mm focal length with filters 0 through 6. Insert note "TARGET=MTF_SN005". The estimated duration is 8 minutes.
71. ✓ **[D]** Record image names and parameters in Image Log.
72. ✓ **[V]** Run fast-look script to verify that the required data were obtained.
73. **[D, L]** Notes: validated

last suffix: 27

filters: 5 lower MTF → 975 nm
6 lower MTF → 1013 nm
long exposure times, higher dark current
→ re-run filters 5 & 6 with lights
repositioned lights closer to target
now 1.5 m away from target
last suffix: 35

Date 4/30 Time 14:00 Initials [illegible]

Time Check 1

IF MORE THAN 1.0 HOUR AHEAD OF SCHEDULED END, CONTINUE.
OTHERWISE, SKIP TO THE SHUTDOWN PROCEDURE.

Scheduled End Time	Current Time	Time Ahead of Scheduled End
12:30 pm -	2:05 m =	N/A

74. **[D, L]** ✓ Record the time in the table above and determine if there is time for more testing

75. **[D, L]** Notes: defer Z-style testing to ambient cleanroom

Date ______ Time ______ Initials ______

Scene 3 Z-Stack for the Left Mastcam-Z

skip

76. **[M,T]** Position the MTF target to Scene 3 as described in Table 2.
77. **[M]** Measure and record the location of the MTF target.

____

____

____

78. **[D]** Record climate information:
    - TVAC temp ______________ Port temp ______________
    - Camera CCD temp ______________ Optics temp ______________
79. **[D,T]** Take digital pictures of the setup and MTF target.
80. **[O]** Capture a tests frame at 34mm with filter 0, and rsync data to the validator. Use prefix **471TAMBL00.**
81. **[V,T]** Load the image in MTF Mapper to find the correct target position. Recapture an autofocused frame if necessary.
82. **[O]** Autofocus once the target is properly placed.
83. **[O,V]** From the autofocus results, update the following script to capture a z-stack of 16 frames in 24 motor-count increments centered on the best focus position.
84. **[O]** Load and execute camera script **471TAMBL06**, which captures a z-stack of 16 frames at 34mm focal length with filter 0. Insert note "TARGET=MTF_SN007". The duration is 2 minutes.
85. **[D]** Record image names and parameters in Image Log.
86. **[V]** Run fast-look script to verify that the required data were obtained.
87. **[D, L]** Notes: ____

____

____

Date ______ Time ______ Initials ______

skip

Scene 3 Z-Stack for the Right Mastcam-Z

88. **[M,T]** Position the MTF target to Scene 3 as described in Table 2.
89. **[M]** Measure and record the location of the MTF target.

____

____

____

90. **[D]** Record climate information:
    - TVAC temp ______________ Port temp ______________
    - Camera CCD temp ______________ Optics temp ______________
91. **[D,T]** Take digital pictures of the setup and MTF target.
92. **[O]** Capture a tests frame at 34mm with filter 0, and rsync data to the validator. Use prefix **471TAMBR00.**
93. **[V,T]** Load the image in MTF Mapper to find the correct target position. Recapture an autofocused frame if necessary.
94. **[O]** Autofocus once the target is properly placed.
95. **[O,V]** From the autofocus results, update the following script to capture a z-stack of 16 frames in 24 motor-count increments centered on the best focus position.
96. **[O]** Load and execute camera script **471TAMBR06**, which captures a z-stack of 16 frames at 34mm focal length with filter 0. Insert note "TARGET=MTF_SN007". The duration is 2 minutes.
97. **[D]** Record image names and parameters in Image Log.
98. **[V]** Run fast-look script to verify that the required data were obtained.
99. **[D, L]** Notes: ____

____

____

Date 4/30 Time 14:07 Initials [illegible]

Shutdown Procedure

skip

100. **[D,T]** ____ Take digital pictures of this page and the test setup.
101. **[D,O]** ✓ Review entries in Image Log, GSE command log, and image headers.
102. **[D, L]** ~~✓~~ Review calibration procedure and ensure that each task is initialed.
103. **[D, L]** Notes: check marks in lieu of initials

104. **[V, L]** ✓ Before making the decision to break down the test setup, ensure that adequate data were acquired for the test requirements. See "MastcamZCalPlan" for these requirements.
105. **[V]** Notes: ____

Data Validator (signature) [illegible]

Date 4/30/19 Time 2:08 PM

106. **[V, L]** ✓ Give the go/no-go decision. Have enough data been acquired to fulfill test requirements? See "MastcamZCalPlan" for these requirements.
107. **[D, L]** ~~✓~~ Update the Log ~~Document~~. "as-run"
108. **[L]** Notes: ____

Calibration Lead (signature) [illegible]

Date 4/30/19 Time 14:08

Date 4/30 Time 14:16 Initials [illegible]

109. **[O, L]** ✓ Ensure that the camera and GSE are in a safe state. mech. homed camera heads powered
110. **[O,D]** ✓ Review the Image Log with the documentarian. Exchange high-fives.
111. **[O]** Notes: ______

Camera Operator (signature) [signature]

Date 4/30/19 Time 14:15

112. **[T]** ✓ If the next test does not require the target, position it away from the chamber or bench. Otherwise, be sure not to move it. The next test is JR
113. **[T]** ✓ Ensure that all other test equipment is safely put away.
114. **[T]** Notes: ______

Technician (signature) [signature]

Date 4-30-19 Time 2:15

115. **[D, L]** ✓ Double-check this procedure and ensure that the top of each page has valid data, time and initials.
116. **[D]** [illegible] Photo-scan this document, save it on the cloud, and file the hard-copy in the Log Binder. Upload the digital pictures taken during this test in the appropriate archive on the cloud. The required links are on the Wiki.
117. **[D]** ✓ Double-check that every required cell the Image Log is accurately filled. When this is complete, print the Image Log and file it the Log Binder after this document.
118. **[D]** Notes: ______

Documentarian (signature) [signature]

Date 4/30/19 Time 14:27
