# Supplementary material for: Pre-Flight Calibration of the Mars 2020 Rover Mastcam Zoom (Mastcam-Z) Multispectral, Stereoscopic Imager
Source: Space Sci Rev. 2021 Feb 18;217(2):29. doi: 10.1007/s11214-021-00795-x (PMC7892537; doi:10.1007/s11214-021-00795-x)
Supplement: Supplementary file 1 — (ZIP 98.6 MB) [file 11214_2021_795_MOESM1_ESM.zip › CalPro_471_MTF_v2_08.pdf]

Date 5/3/19 Time 18:00 Initials [illegible]

MTF Calibration Procedure for Mastcam-Z Ambient TVAC Testing (Pro. 4.7.1)

[Procedure version 2.08, prepared by the Mastcam-Z calibration team at Cornell University.]

These measurements are performed on the camera and at the temperature designated below as specified in the Calibration Plan (Document #),

Unit Under Test:

R FM __X__ **L FM** __X__ **EQM**____ **Other**________________

Test Performed at Temperature:

-35°C______ **- 10°C**____ **+5°C**______ **Ambient** __X__ **Other** __________

These measurements are performed at,

MSSS __X__ **ASU**_______ **Other**________

Date 5/3/19 **Start Time** 17:20 **End Time** 00:40

Estimated Duration __6.0 hours__

Scheduled Start Time 16:00 **Sch. End Time** N/A

Calibration Lead [L] KEN **Documentarian [D]** EMILY, TINA

Camera Operator [[redacted]] JASON, KW, DD **Technician [T]** ANDY, MEGAN

Data Validator [V] MASON **Metrologist [M]** —

Other ________________

Change Log

Version	Name	Change
v1_01<br>1 Oct 2018	C. Tate	(first draft)
v2_08<br>3 May 2019	C. Tate	Approved version prior to FM testing

Document Approval

Approved by James Bell — Date
Mastcam-Z PI
Arizona State University

5/3/19
Approved by Alexander Hayes — Date
Mastcam-Z Calibration Working Group
Lead, Cornell University

5/3/19
Approved by Justin Maki — Date
Mastcam-Z Deputy PI and Investigation
Scientist, Jet Propulsion Laboratory

5/3/19
Approved by: — Date
Ken Herkenhoff
Mastcam-Z Co-Investigator, USGS

Approved by — Date

5/3/19
Approved by Christian Tate — Date
Procedure Author
Cornell University

Table of Contents

MTF CALIBRATION PROCEDURE FOR MASTCAM-Z AMBIENT TVAC TESTING (PRO. 4.7.1) 1

CHANGE LOG 2
DOCUMENT APPROVAL 2
TEST DESCRIPTION 3
SOFTWARE PREPARATION 4
Table 1. File naming convention for the camera script prefixes and frame filenames: "AAABBBBCDD" 4
HARDWARE INSTALLATION 5
Figure 1. MSSS Floor Plan for Geometric Testing in the cleanroom 6
Table 2. The nominal target placement scenes for the geometric testing 7
SCENE 1 FOR THE MASTCAM-ZS 8
SCENE 2 FOR THE MASTCAM-ZS 9
SCENE 3 FOR THE MASTCAM-ZS 10
SCENE 4 FOR THE MASTCAM-ZS 12
SCENE 5 FOR THE MASTCAM-ZS 13
SCENE 6 FOR THE MASTCAM-ZS 14
SCENE 7 FOR THE MASTCAM-ZS 15

SHUTDOWN PROCEDURE 17

Test Description

Excerpt from the Calibration Plan 4.7

The objective of this test is to image well-characterized bar targets at multiple focus and zoom positions in order to characterize the Modulation Transfer Function (MTF) and depth of field of each camera. Targets should be imaged at ~50% full well using the Bayer RGB (priority 1), 805 nm, (priority 2), and remaining non-solar filters (priority 3). Obtain a minimum of 3 images of each target per filter, focus, and zoom position. Multiple images are needed to reduce errors in determining target locations in the image plane.

MTF is an effective means of specifying the resolution of an optical system. Resolution is defined as the minimum feature size of an object that can be distinguished by an imaging system. The Point Spread Function (PSF) is the inverse Fourier Transform of the MTF—the PSF describes optical performance in the spatial domain while the MTF expresses optical performance in the frequency domain. Images of the bar targets and knife edge or point source targets at various zoom and focus positions will be used to determine PSF, depth of field, and MTF. The bar target shall consist of a chart containing horizontal, vertical, and diagonal lines and bars of varying thicknesses as well as circular dots of various sub- and super-pixel sizes.

In addition to determining the optical performance of the optomechanical assemblies, the images collected during MTF/PSF calibration will also be used to determine the numerical value and repeatability of the stepper motor counts for the Hall Effect sensors used to measure the position

of the focus group and two moving zoom groups in the optical zoom assemblies. This will determine the relationship between stepper motor count for each focus/zoom group, working distance, and pixel scale. Owing to thickness variations between spectral filters, focus shifts may occur and images would ideally be obtained using all non-solar filters.

Note that the Mastcam-Z instrument has an onboard focus merge algorithm that selects and merges the best-focus portions of a scene using a focus stack (or z-stack) of multiple images (up to 16) at varying focal positions. While usually only the best-focus or merged product is saved to file, the raw frames for the focus stack can also be saved to file without appreciably increasing the observation or product generation time. For tests where images at multiple focus positions are desired, such as during MTF/PSF Calibrations, the entire focus stack of images will be written to file. Since images at multiple focus positions are acquired irrespective of whether or not they are written to memory, saving multiple focus positions per zoom setting does not require additional test time (outside of the time required to write the files to disk in the GSE). Multiple focus position images shall be saved during both Stand-Alone and ATLO MTF/PSF testing.

Software Preparation

The software and files required for this test are prepared well in advance of test day. This checklist ensures that the following are present, debugged, and executable: (1) all fast look scripts, (2) automated header generation of all relevant camera parameters, target positioning, and metadata, (3) all camera scripts that command the camera unit, and (4) the directories/file-paths pointing to the data repositories of this specific test.

Table 1. File naming convention for the camera script prefixes and frame filenames: "AAABBBBCDD"

Code	Name	Example	Value(s)
"AAA"	Calibration Plan Section	"411" = Cal. Plan 4.1.1 chapter 4, section 1, subsection 1	471, 491
"BBBB"	Location of test or ASU TVAC temperature	"MSSS" = test at MSSS, "ATLO" = test at JPL ATLO, "TN10" = ASU TVAC -10C, …	TAMB
"C"	Camera unit under test	"L" = Left Mastcam-Z, "R" = Right Mastcam-Z, "E" =EQM, "C" =COTS	R/L
"DD"	Part of test	"00" = test set up, "01" = first radiance level …	00-12

Date 5/3/19 Time 17:20 Initials

1. **[D]** ____ Look up the daily calibration schedule and record the scheduled start and end time of this test on the cover page of this document. Also, fill out and double-check the other information on the cover page.
2. **[D]** ____ Ensure that all supplemental manuals are on hand. These are,
   - Validator_Manual, Documentarian_Manual,
   - MastcamZCalPlan
3. **[D]** ____ Ensure that the Image Log is present and ready to use. Find and open the Google Sheets file "Image_Log_46". The duration is 2 minutes. There is a link on the Wiki.
4. **[V]** ____ Check that all *Calgorithms* fast-look and validation scripts are present, up-to-date, and ready to analyze test output. Find and open the "Geometric_Calibration_47_Validation" Jupyter notebook. There is a link on the Wiki.
5. **[O]** ____ Check that all camera scripts required for this test are present, up-to-date and ready to command the ground support equipment (GSE). These are,
   - **471TAMBR00 - 471TAMBR13, 491TAMBR00 - 491TAMBR12**
   - **471TAMBL00 - 471TAMBL13, 491TAMBL00 - 491TAMBL12**
6. **[O,V,D, L]** Notes:

____

____

____

Hardware Installation

This procedure is for the ambient TVAC chamber testing at MSSS. Figure 1 shows the nominal layout of the cleanroom, workspace, Mastcam-Zs, ground support equipment (GSE), targets, sources, and other equipment necessary.

Date 5/3 Time 17:47 Initials ZL

Figure 1. MSSS Floor Plan for Geometric Testing in the cleanroom.

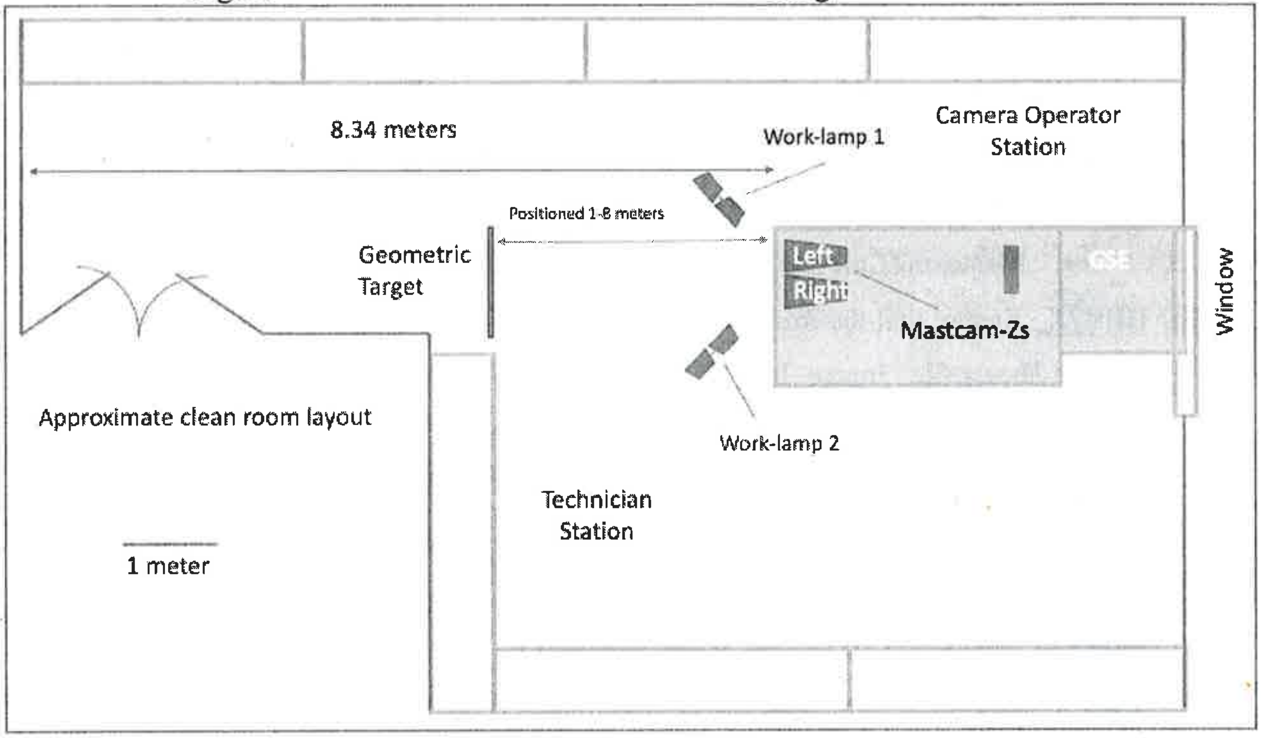

7. **[T,O, L]** ZL Ensure that all personnel in the cleanroom are following the cleanroom practices for electrostatic discharge, proper clothing, and other safety concerns. See "ESD_Manual" and "Cleanroom_Manual". The duration is 2 minutes.
8. **[T]** ZL Double check that ionized air is flowing over the Mastcam-Zs.
9. **[T]** ____ Install the blue and infrared bright lamps and position them in front of the geometric target and out of the camera's field of view (FOV). Power them on and optimally position them.
10. **[O,T]** ZL Ensure that the camera unit and GSE wires are secure, kink-free, and do not present tripping hazards when the lights are turned off.
11. **[O,D]** ZL Check the camera temperature and ensure nominal operation.
12. **[D]** ZL Record the following environmental information:
    - Cleanroom temperature 67.2°F pressure ______ humidity 66%
13. **[O,D, L]** Notes:

____

____

Date 5/3 Time 17:48 Initials [illegible]

TEST RIGHT SCRIPT FIRST 471 TAMB R00

Table 2. The nominal target placement scenes for the geometric testing.

Scenes	Target Placements	Notes
**Scene 1**	Large DEPTH_SN007 (Side B) target at about 2 meters centered on the 48mm boresight	ROTATED TARGET BETWEEN IMAGE SETS
**Scene 2**	Large MTF_SN007 (Side A) target at about 2 meters centered on the 34mm boresight	SETUP DIFFICULT, ERRORS ENCOUNTERED
**Scene 3**	Large MTF_SN007 (Side A) target at about 3 meters centered on the 63mm boresight	
**Scene 4**	Small MTF_SN005 (Side A) target at about 2 meters centered on the 100mm boresight	
**Scene 5**	Small MTF_SN005 (Side A) target at about 3 meters centered on the 63mm boresight	
**Scene 6**	Large Star_ (Side A) target at about 2 meters centered on the 34mm boresight	
**Scene 7**	Large Star_ (Side A) target at about 3 meters centered on the 63mm boresight	

Date 9/3/19 Time 17:48 Initials [illegible]

Scene 1 for the Mastcam-Zs

14. **[M,T]** [illegible] Position the Depth of Field target to Scene 1 as described in Table 2.
15. **[M]** [illegible] Measure and record the location of the MTF target.

    METROLOGY

16. **[D]** [illegible] Record the following temperatures:
    - Left Camera CCD temp 24.3°C
    - Right Camera CCD temp 24.9°C
17. **[D,T]** [illegible] Take pictures the geometric target position and the whole test/GSE set-up.
18. **[O]** [illegible] Capture a tests frames at 34mm with filter 0 of both cameras, and rsync data to the validator. Use prefixes **471TAMBL00** and **471TAMBR00.** STET
19. **[V,T]** [illegible] Load the image in MTF Mapper to find the correct target position. Recapture an autofocused frame if necessary.

20. **[O]** ____ Load and execute camera script **491TAMBL05**, which autofocuses and captures 3 frames at 34mm focal length with filters 0 through 6. Insert note "TARGET=Depth_SN007". The estimated duration is 12 minutes.
21. **[[redacted]]** ____ Load and execute camera script **491TAMBL05**, which autofocuses and captures 3 frames at 34mm focal length with filters 0 through 6. Insert note "TARGET= Depth _SN007". The estimated duration is 12 minutes.
22. **[D, L]** Notes: ____________________

23. **[[redacted],V, L]** Load camera script **491TAMBL09** and edit all manual z-stack focus values around filter 0's best focus found by the previous autofocus. Insert note "TARGET= Depth _SN007". The estimated duration is 16 minutes.
24. **[O,V, L]** Load camera script **491TAMBR09** and edit all manual z-stack values around filter 0's best focus found by the previous autofocus. Insert note "TARGET= Depth _SN007". The estimated duration is 16 minutes.

SKIP

25. **[D]** Record image names and parameters in Image Log.
26. **[V]** Run fast-look script to verify that the required data were obtained.
27. **[D, L]** Notes: ROTATE TARGET AND REPEAT @ 48mm

Date 5/3 Time 8:35 Initials [illegible]

R=25.1°C L=24.6°C
TEST IMAGES IN L2+R2; GO TO 34mm F.L.
INCANDESCENT FIRST, AUTOEXPOSURE = 191.3, 453.3 ms
LED LAMP SECOND, AUTOEXPOSURE = 3200., 10,000. ms !

Scene 2 for the Mastcam-Zs

28. [M,T] [illegible] Position the MTF target to Scene 2 as described in Table 2.
29. [M] [illegible] Measure and record the location of the MTF target.
~~2.008~~ ~~[illegible]~~m FROM CAMERAS TO TARGET = 2.55 m

30. [D] [illegible] Record the following temperatures:
    - Left Camera CCD temp 24.5°C
    - Right Camera CCD temp 24.9°C
31. [D,T] [illegible] Take pictures the geometric target position and the whole test/GSE set-up. IMAGE IDS ~~[illegible]~~20–~~[illegible]~~24
32. [O] [illegible] Capture a tests frames at 34mm with filter 0 of both cameras, and rsync data to the validator. Use prefixes **471TAMBL00** and **471TAMBR00.** →50
    TURN ON INCANDESCENT BULB
33. [V,T] [illegible] Load the image in MTF Mapper to find the correct target position. Recapture an autofocused frame if necessary. MOVED BACK TO 2.43m, AUTOFOCUSED ROTATED CLOCKWISE, YAWED RT., THEN CHANGED TO DIFFERENT TRIPOD.
34. [O] [illegible] Load and execute camera script **4~~9~~71TAMBL05**, which autofocuses and captures 3 frames at 34mm focal length with (1ST SET) filters 0 through 6. Insert note "TARGET=MTF_SN007". The estimated duration is 12 minutes.
35. [O] [illegible] Load and execute camera script **4~~9~~71TAMB~~L~~R05** (2ND SET), which autofocuses and captures 3 frames at 34mm focal length with filters 0 through 6. Insert note "TARGET=MTF_SN007". The estimated duration is 12 minutes.
36. [D, L] Notes: 24.7°C (R), 24.2°C (L)
    AUTOFOCUS FAILURES ON R2-6, L1 — ~~WRONG SCRIPTS~~! RERUN,
    ~~SHOULD BE 471TAMBL05 AND 471TAMBR05~~ WITH NEW FOCUS SEEDS.
    BEST FOCUS L0 = -84, R0 = -36. ← NEGATIVE VALUES NOT ALLOWED IN SCRIPTS
37. [[redacted],V, L] Load camera script **4~~9~~71TAMBL09** and edit all manual z-stack focus values around filter 0's best focus found by the previous autofocus. Insert note "TARGET=MTF_SN007". The estimated duration is 16 minutes.

ERROR: MOTOR COUNTS BETWEEN 50 AND 102 COMMANDED → NO MECH ERROR. EDITED SCRIPT TO REPLACE MATH WITH MANUALLY-CALCULATED VALUES.

Date 5/3 Time 22:05 Initials [signature]

38. [[redacted],V, L] Load camera script 4~~9~~7 1TAMBR09 and edit all manual z-stack values around filter 0's best focus found by the previous autofocus. Insert note "TARGET=MTF_SN007". The estimated duration is 16 minutes.
    MANUAL EDITS RAN FINE.
39. [D] Record image names and parameters in Image Log.
40. [V] Run fast-look script to verify that the required data were obtained.
41. [D, L] Notes: EXTRA LAMPS OFF
    24.5°C

Scene 3 for the Mastcam-Zs

42. [M,T] [initials] Position the MTF target to Scene 3 as described in Table 2.
43. [M] [initials] Measure and record the location of the MTF target.
    ~~4.98 m~~ ~~3.993m~~ 4.68 m
44. [D] [initials] Record the following temperatures:
    - Left Camera CCD temp 23.7°C
    - Right Camera CCD temp 24.1°C
45. [D,T] [initials] Take pictures the geometric target position and the whole test/GSE set-up. IMAGE IDS 33-37
46. [O] [initials] Capture a tests frames at 63mm with filter 0 of both cameras, and rsync data to the validator. Use prefixes **471TAMBL00** and **471TAMBR00.**
47. [V,T] [initials] Load the image in MTF Mapper to find the correct target position. Recapture an autofocused frame if necessary. REPEATED
    EXTRA LAMPS ON
48. [O] [initials] Load and execute camera script 4~~9~~7 1TAMBL06 #7TET, which autofocuses and captures 3 frames at 63mm focal length with filters 0 through 6. Insert note "TARGET=MTF_SN007". The estimated duration is 12 minutes.

Date 5/3 Time 23:44 Initials [illegible]

STET
R

49. **[O]** [illegible] Load and execute camera script **491TAMBL06**, which autofocuses and captures 3 frames at 63mm focal length with filters 0 through 6. Insert note "TARGET=MTF_SN007". The estimated duration is 12 minutes.

50. **[D, L]** Notes: EXTRA LAMPS OFF
BEST FOCUS LØ=2472, RØ = ~~[illegible]~~ 2418

✓51. **[O,V, L]** Load camera script **4~~9~~1TAMBL10** (7) and edit all manual z-stack focus values around filter 0's best focus found by the previous autofocus. Insert note "TARGET=MTF_SN007". The estimated duration is ~~16~~ minutes.

✓52. **[O,V, L]** Load camera script **4~~9~~1TAMBR10** (7) and edit all manual z-stack values around filter 0's best focus found by the previous autofocus. Insert note "TARGET=MTF_SN007". The estimated duration is ~~16~~ minutes. MUCH SHORTER

✓53. **[D]** Record image names and parameters in Image Log.

✓54. **[V]** Run fast-look script to verify that the required data were obtained.

55. **[D, L]** Notes: STOPPED FOR THE NIGHT AT THIS POINT.
POWERED DOWN CAMERAS

Skip

Scene 4 for the Mastcam-Zs

56. **[M,T]** ____ Position the MTF target to Scene 4 as described in Table 2.
57. **[M]** ____ Measure and record the location of the MTF target.

____

____

____

58. **[D]** ____ Record the following temperatures:
    - Left Camera CCD temp ______________
    - Right Camera CCD temp ______________
59. **[D,T]** ____ Take pictures the geometric target position and the whole test/GSE set-up. *IMAGE IDS*
60. **[O]** ____ Capture a tests frames at 63mm with filter 0 of both cameras, and rsync data to the validator. Use prefixes **471TAMBL00** and **471TAMBR00.**
61. **[V,T]** ____ Load the image in MTF Mapper to find the correct target position. Recapture an autofocused frame if necessary.

EXTRA LAMPS ON *STET*

62. **[O]** ____ Load and execute camera script **491TAMBL06**, which autofocuses and captures 3 frames at 63mm focal length with filters 0 through 6. Insert note "TARGET=MTF_SN005". The estimated duration is 12 minutes.
63. **[[redacted]]** ____ Load and execute camera script **491TAMBL06**, which autofocuses and captures 3 frames at 63mm focal length with filters 0 through 6. Insert note "TARGET=MTF_SN005". The estimated duration is 12 minutes. *STET*
64. **[D, L]** Notes: *EXTRA LAMPS OFF*

____

____

65. **[O,V, L]** Load camera script **491TAMBL10** and edit all manual z-stack focus values around filter 0's best focus found by the previous autofocus. Insert note "TARGET=MTF_SN005". The estimated duration is 16 minutes.
66. **[[redacted],V, L]** Load camera script **491TAMBR10** and edit all manual z-stack values around filter 0's best focus found by the previous autofocus. Insert note "TARGET=MTF_SN005". The estimated duration is 16 minutes.

67. **[D]** Record image names and parameters in Image Log.
68. **[V]** Run fast-look script to verify that the required data were obtained.
69. **[D, L]** Notes: ____

____

____

Skip

Scene 5 for the Mastcam-Zs

70. **[M,T]** ____ Position the MTF target to Scene 5 as described in Table 2.
71. **[M]** ____ Measure and record the location of the MTF target.

____

____

____

72. **[D]** ____ Record the following temperatures:
    - Left Camera CCD temp ______________
    - Right Camera CCD temp ______________
73. **[D,T]** ____ Take pictures the geometric target position and the whole test/GSE set-up.
74. **[O]** ____ Capture a tests frames at 63mm with filter 0 of both cameras, and rsync data to the validator. Use prefixes **471TAMBL00** and **471TAMBR00.**
75. **[V,T]** ____ Load the image in MTF Mapper to find the correct target position. Recapture an autofocused frame if necessary.

EXTRA LAMPS ON

76. **[O]** ____ Load and execute camera script **4~~9~~71TAMBL07**, which autofocuses and captures 3 frames at 63mm focal length with filters 0 through 6. Insert note "TARGET=MTF_SN005". The estimated duration is 12 minutes.
77. **[O]** ____ Load and execute camera script **4~~9~~71TAMBL07**, which autofocuses and captures 3 frames at 63mm focal length with filters 0 through 6. Insert note "TARGET=MTF_SN005". The estimated duration is 12 minutes.
78. **[D, L]** Notes: EXTRA LAMPS OFF ____

____

____

79. **[O,V, L]** Load camera script **4~~9~~71TAMBL11** and edit all manual z-stack focus values around filter 0's best focus found by the previous autofocus. Insert note "TARGET=MTF_SN005". The estimated duration is 16 minutes.

80. **[**[redacted]**,V, L]** Load camera script **491TAMBR11** and edit all manual z-stack values around filter 0's best focus found by the previous autofocus. Insert note "TARGET=MTF_SN005". The estimated duration is 16 minutes.

81. **[D]** Record image names and parameters in Image Log.
82. **[V]** Run fast-look script to verify that the required data were obtained.
83. **[D, L]** Notes: ____

Skip

Scene 6 for the Mastcam-Zs

84. **[M,T]** ____ Position the MTF target to Scene 2 as described in Table 2.
85. **[M]** ____ Measure and record the location of the MTF target.

86. **[D]** ____ Record the following temperatures:
    - Left Camera CCD temp ____________
    - Right Camera CCD temp ____________
87. **[D,T]** ____ Take pictures the geometric target position and the whole test/GSE set-up. IMAGE IDS
88. **[O]** ____ Capture a tests frames at 34mm with filter 0 of both cameras, and rsync data to the validator. Use prefixes **471TAMBL00** and **471TAMBR00.**
89. **[V,T]** ____ Load the image in MTF Mapper to find the correct target position. Recapture an autofocused frame if necessary.

EXTRA LAMPS ON

90. **[**[redacted]**]** ____ Load and execute camera script **491TAMBL05**, which autofocuses and captures 3 frames at 34mm focal length with filters 0 through 6. Insert note "TARGET=Star_". The estimated duration is 12 minutes.
91. **[O]** ____ Load and execute camera script **491TAMBL05**, which autofocuses and captures 3 frames at 34mm focal length with filters 0 through 6. Insert note "TARGET= Star_". The estimated duration is 12 minutes.
92. **[D, L]** Notes: EXTRA LAMPS OFF

____

____

Skip

93. **[O,V, L]** Load camera script **471TAMBL09** and edit all manual z-stack focus values around filter 0's best focus found by the previous autofocus. Insert note "TARGET= Star_". The estimated duration is 16 minutes.
94. **[O,V, L]** Load camera script **471TAMBR09** and edit all manual z-stack values around filter 0's best focus found by the previous autofocus. Insert note "TARGET= Star_". The estimated duration is 16 minutes.

95. **[D]** Record image names and parameters in Image Log.
96. **[V]** Run fast-look script to verify that the required data were obtained.
97. **[D, L]** Notes: ____

____

____

Scene 7 for the Mastcam-Zs

98. **[M,T]** ____ Position the MTF target to Scene 3 as described in Table 2.
99. **[M]** ____ Measure and record the location of the MTF target.

____

____

____

100. **[D]** ____ Record the following temperatures:
    - Left Camera CCD temp ______________
    - Right Camera CCD temp ______________
101. **[D,T]** ____ Take pictures the geometric target position and the whole test/GSE set-up.
102. **[O]** ____ Capture a tests frames at 63mm with filter 0 of both cameras, and rsync data to the validator. Use prefixes **471TAMBL00** and **471TAMBR00.**
103. **[V,T]** ____ Load the image in MTF Mapper to find the correct target position. Recapture an autofocused frame if necessary.

Skip

104. **[O]** ____ Load and execute camera script **491TAMBL06**, which autofocuses and captures 3 frames at 63mm focal length with filters 0 ~~through 6.~~ Insert note "TARGET= Star_". The estimated duration is 12 minutes.

105. **[O]** ____ Load and execute camera script **491TAMBL06**, which autofocuses and captures 3 frames at 63mm focal length with filters 0 ~~through 6.~~ Insert note "TARGET= Star_". The estimated duration is 12 minutes.

106. **[D, L]** Notes: ____

____

____

107. **[O,V, L]** Load camera script **491TAMBL10** and edit all manual z-stack focus values around filter 0's best focus found by the previous autofocus. Insert note "TARGET= Star_". The estimated duration is 16 minutes.

108. **[O,V, L]** Load camera script **491TAMBR10** and edit all manual z-stack values around filter 0's best focus found by the previous autofocus. Insert note "TARGET= Star_". The estimated duration is 16 minutes.

109. **[D]** Record image names and parameters in Image Log.

110. **[V]** Run fast-look script to verify that the required data were obtained.

111. **[D, L]** Notes: ____

____

____

Date 5/4 Time 00:23 Initials [illegible]

Shutdown Procedure

112. **[D,T]** [illegible] Take pictures of the test setup. 54-56 IMAGE IDS
113. **[D,O]** [illegible] Review entries in Image Log, GSE command log, and image headers.
114. **[D, L]** [illegible] Review calibration procedure and ensure that each task is initialed.
115. **[D, L]** Notes: REMOVE LED LAMP FROM CLEAN ROOM

116. **[V, L]** [illegible] Before making the decision to break down the test setup, ensure that adequate data were acquired for the test requirements. See "MastcamZCalPlan" for these requirements.
117. **[V]** Notes: Paul was able to load some images into MTF Mapper that Marco wasn't able to.

Data Validator (signature) [illegible]

Date 5/4/19 Time 00:45

118. **[V, L]** [illegible] Give the go/no-go decision. Have enough data been acquired to fulfill test requirements? See "MastcamZCalPlan" for these requirements.
119. **[D, L]** [illegible] Update the Log Document.
120. **[L]** Notes: SEE HANDOVER NOTES

Calibration Lead (signature) [illegible]

Date 5/4/19 Time 00:45

121. [[redacted], L] ____ Ensure that the camera and GSE are in a safe state.
122. [[redacted],D] ____ Review the Image Log with the documentarian. Exchange high-fives.
123. [[redacted]] Notes: ____

Camera Operator (signature) ____

Date 5/4/19 Time 00:56

124. **[T]** ____ If the next test does not require the target, position it away from the chamber or bench. Otherwise, be sure not to move it. The next test is JR CAL.
125. **[T]** ____ Ensure that all other test equipment is safely put away.
126. **[T]** Notes: ALL LIGHTS FOR TARGET OFF, UNPLUGGED, AND COOLED. JR TARGET IS IN THE CONFERENCE ROOM.

Technician (signature) ____

Date 5/4/19 Time 00:56

127. **[D, L]** ____ Double-check this procedure and ensure that the top of each page has valid data, time and initials.
128. **[D]** ____ Photo-scan this document, save it on the cloud, and file the hard-copy in the Log Binder. Upload the digital pictures taken during this test in the appropriate archive on the cloud. The required links are on the Wiki.
129. **[D]** ____ Double-check that every required cell the Image Log is accurately filled. When this is complete, print the Image Log and file it the Log Binder after this document.
130. **[D]** Notes: Spreadsheet was set to Eastern time zone. We switched it to Pacific + times updated. Times recorded from earlier days may be off by 3 hours - need to check.

Documentarian (signature) ____

Date 5/4/19 Time 12:50 am

Day 8, Shift 2

Lead: Ken
Operations: Jason, Katherine, Darian
Technician: Andy, Megan shadowing
Validator: Mason
Documentarian: Emily, Tina shadowing

Hardware Status:

Mech State: Homed.
DEA State: Power on
Camera Head State: Power off
Go/No-Go, Facilities, Hardware Prep

Procedures Completed During Last Shift

Depth of field images were taken at 48 mm focal length with the target in the surveyed configuration, then again after rotating the target about a vertical axis. MTF data were acquired using the large target (Scenes 2 and 3) only.

Data Review Status

Most data were nominal, see exceptions below.

Any data to be reacquired?

Yes, we need data for L6 only, Scene 3 (491TAMBL06) due to the autofocus failure described below.

Lessons Learned/Anomalies from last shift

Adjusting the orientation of the MTF target relative to cameras was difficult, requiring changing to a tripod with a ball joint. We realized later that the 1-degree target orientation tolerance (in all 3 axes) that we were working toward was too restrictive, so we wasted some time on Scenes 2 and 3. !

Typos in the MTF procedure confused us repeatedly—at steps 37 and 38, we realized that the script names should start with 471 instead of 491. We had to change the autofocus seed for the 491TAMB*05 scripts to enable focus to be found. The L0 and R0 autofocus positions found by autofocus in the first step were used to replace "var1" in the 471 script, which then failed because the resulting motor counts were between 50 and 102, causing BAD MECH errors. We could not avoid the errors by deleting commands in this range, so had to call Tex back in. Math in scripts does not work on negative values, so the values were calculated manually and edited into the scripts. This solved the problem.

The autofocus seeds were proactively changed in the 491TAMB*06 scripts based on the experience described above, which worked well. But we had autoexposure failures on a few

filters, which required more script edits and another run on both cameras. Autofocus failed again on filter L6, and we could not figure out why.

Script 471TAMB*10 was predicted to take 16 minutes, but ran in under 2 minutes.

General Reminders

Jason is authorized to confirm measurements relative to flight hardware in clean room.

Hardware changes must be approved by operations lead and MSSS staff as appropriate, and anyone entering the clean room must be accompanied by an MSSS staff member (ops team).

Techs should be very careful near flight hardware in the clean room! Maintain ESD safe distance (> 1 meter). Wear ESD wrist straps.

Foreign Nationals will be wearing blue badges

New arrivals to be scheduled as shadows initially

Overview of next shift plan

JR geometric calibration

Prep required for next shift:

The top priority of the MTF testing is the L/R0 filters, so we can skip the z-stacks on the other filters. We may be able to use the L/R0 scripts for the other filters. Seiman star images are needed only in L/R0.

We should compare the MTF from the large and small targets at 63 mm to confirm that we get the same result.

Longer term overview

JR geometric calibration Saturday, followed by more MTF if schedule allows
Spectral throughput Sunday
Radiometry/flat field Monday 1st shift
Stray light Tuesday 1st shift
Repeat whole schedule estimate Monday afternoon

New Arrivals/Orientations

Alex, Gerhard, Ernest, Tina, Ravine

Any open items?

Open questions from team members

From: **Herkenhoff, Kenneth** 000002a3bcd0bd75-dmarc-request@LISTS.ASU.EDU
Subject: Mastcam-Z Flight camera calibration status - Day 8, Shift 2
Date: May 4, 2019 at 1:44 AM
To: MASTCAMZ@asu.edu MASTCAMZ@ASU.EDU

KH

Calibration did not go as smoothly this evening. We started by taking depth of field target images in two configurations, then started imaging the large MTF target. We spent a lot of time precisely orienting the target before we realized that such high precision was not required. Some of the imaging scripts had to be edited to get good autofocusing, and we discovered that script arithmetic does not work with negative numbers. We worked around this problem by manually editing focus settings in the script. Then we had some autoexposure failures that required more script edits and another run on both cameras. Autofocus failed again during filter L6 imaging despite our edits, and we are not yet sure why. Hopefully we will have better luck tomorrow!

Lead: Ken
Operations: Jason, Katherine, Darian
Technician: Andy, Megan shadowing
Validator: Mason
Documentarian: Emily, Tina shadowing

--
****

Ken Herkenhoff
U. S. Geological Survey
Astrogeology Science Center
2255 N. Gemini Drive
Flagstaff, AZ 86001-1698
(928) 556-7205 Fax: (928) 556-7014
https://astrogeology.usgs.gov/people/ken-herkenhoff

****

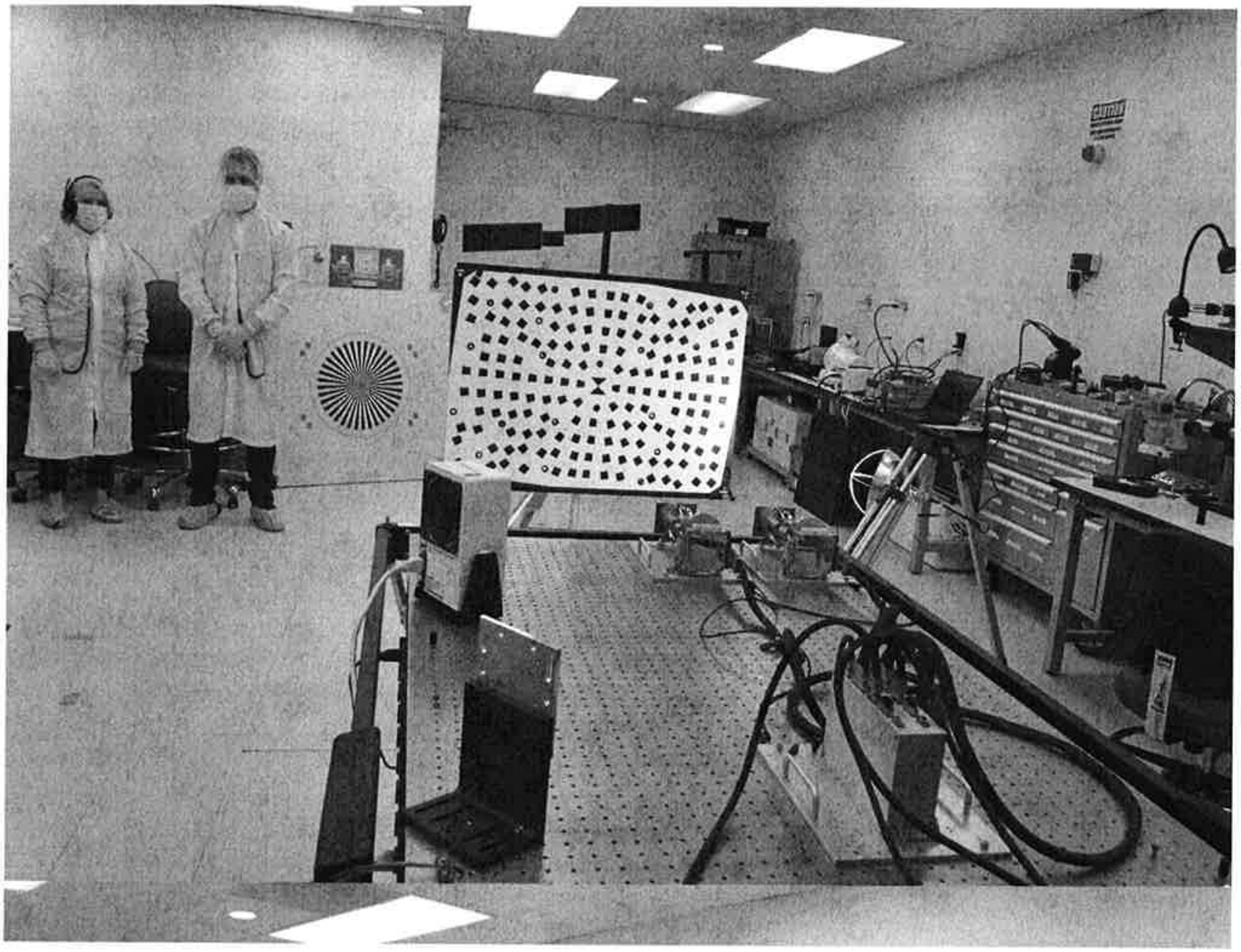

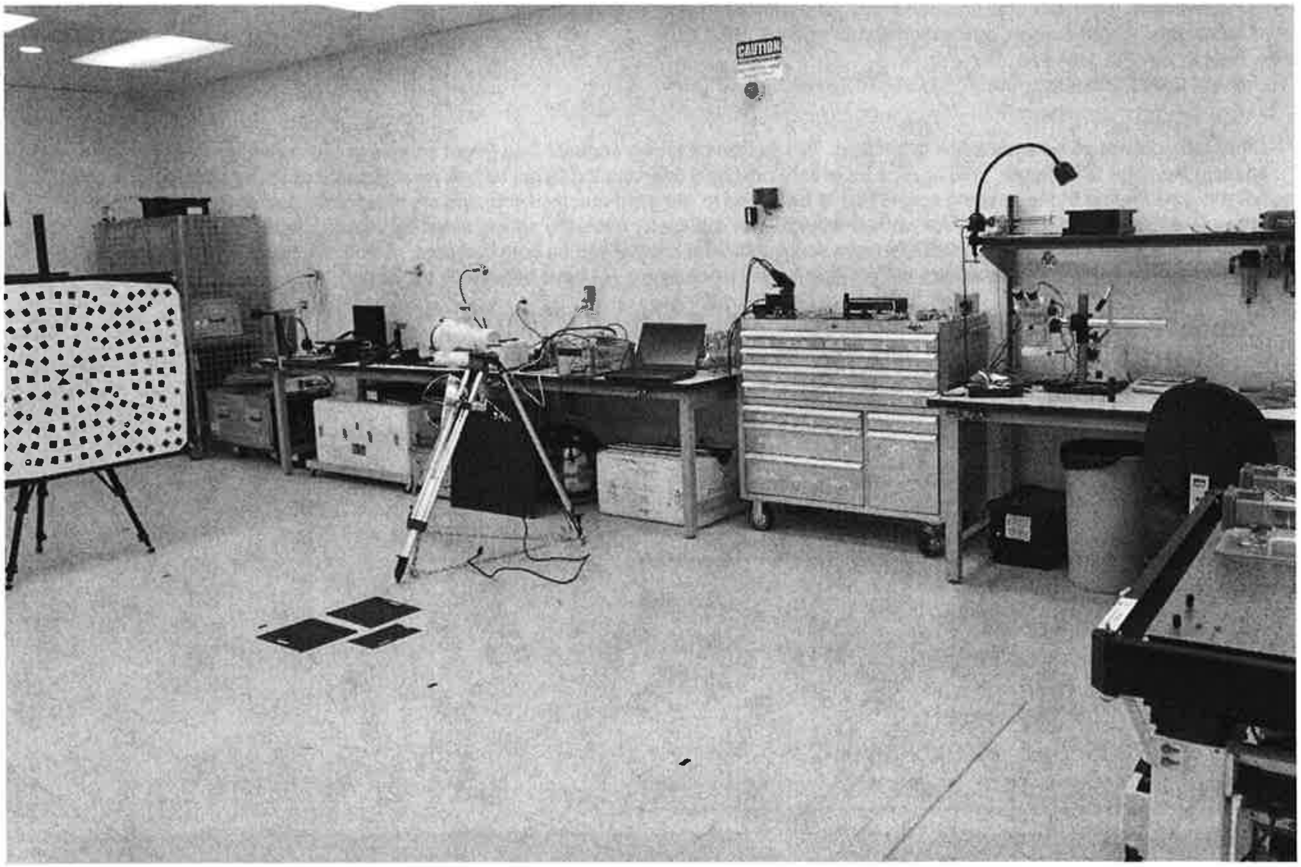
CAUTION

From: **Mike Caplinger** mc@MSSS.COM
Subject: Re: Mastcam-Z Flight camera calibration status - Day 8, Shift 2
Date: May 4, 2019 at 5:52 AM
To: MASTCAMZ@asu.edu MASTCAMZ@ASU.EDU

MC

On 5/4/19 1:42 AM, Herkenhoff, Kenneth wrote:

> Autofocus failed again during filter L6 imaging despite our edits, and we are not yet sure why. Hopefully we will have better luck tomorrow!

Looking at the data I believe this is happening on the visible narrowband filters because the color interpolation kernels aren't set appropriately in the DEA to match the camera hardware (my bad). You should avoid use of autofocus on these filters until I can get this fixed on Monday. Affected filters would be L4, L5, L6 and maybe L3 (and L7 if you can get any light into it). You can recognize this by looking at the autofocus curve results and observing that the metric values are unusually high relative to other filters (the system is trying to compress the Bayer pattern print-though which isn't being removed by the correct interpolation and getting confused.)

If it works on a particular filter, great, but if not, that's likely why. L6 is typically the most challenging one because of low blue signal.
