# Supplementary material for: Pre-Flight Calibration of the Mars 2020 Rover Mastcam Zoom (Mastcam-Z) Multispectral, Stereoscopic Imager
Source: Space Sci Rev. 2021 Feb 18;217(2):29. doi: 10.1007/s11214-021-00795-x (PMC7892537; doi:10.1007/s11214-021-00795-x)
Supplement: Supplementary file 1 — (ZIP 98.6 MB) [file 11214_2021_795_MOESM1_ESM.zip › CalPro_471_MTF_v2_12.pdf]

Date 5/7/19 Time 1800 Initials JRJ

MTF Calibration Procedure for Mastcam-Z Ambient Cleanroom Testing (Pro. 4.7.1)

[Procedure version 2.12, prepared by the Mastcam-Z calibration team at Cornell University]

These measurements are performed on the camera and at the temperature designated below as specified in the Calibration Plan (Document #),

Unit Under Test:

R FM X **L FM** X **EQM**____ **Other**________

Test Performed at Temperature:

-35°C____ **- 10°C**____ **+5°C**____ **Ambient** X **Other** ______

These measurements are performed at,

MSSS X **ASU**____ **Other**____

Date 5/7/19 **Start Time** 1800 **End Time** ~2300

Estimated Duration 3.0 hours

Scheduled Start Time N/A **Sch. End Time** N/A

**Calibration Lead [L]**	Jeff Johnson	**Documentarian [D]**	Nathalie Turenne
**Camera Operator [O]**	Jex, Elsa, Chris	**Technician [T]**	Andy Winhold, Christian Tate
**Data Validator [V]**	Paul, Ole, Noel	**Metrologist [M]**	
**Other**	Alex Hayes		

Change Log

Version	Name	Change
v1_01<br>1 Oct 2018	C. Tate	(first draft)
v2_12<br>7 May 2019	C. Tate	Approved version prior to FM testing

Document Approval

Approved by James Bell Date
Mastcam-Z PI
Arizona State University

5/7/19
Approved by Alexander Hayes Date
Mastcam-Z Calibration Working Group Lead, Cornell University

Approved by Justin Maki Date
Mastcam-Z Deputy PI and Investigation Scientist, Jet Propulsion Laboratory

5/8/19
Approved by:
Date
Jeff Johnson
Mastcam-Z Co-Investigator, APL

Approved by Date

Approved by Christian Tate Date
Procedure Author
Cornell University

Table of Contents

MTF CALIBRATION PROCEDURE FOR MASTCAM-Z AMBIENT CLEANROOM TESTING (PRO. 4.7.1) 1
Change Log 2
Document Approval 2
Test Description 3
Software Preparation 4
Table 1. File naming convention for the camera script prefixes and frame filenames: "AAABBBBCDD" 4
Hardware Installation 5
Figure 1. MSSS Floor Plan for Geometric Testing in the cleanroom. 5
Table 2. The nominal target placement scenes for the geometric testing 7
Scene 6 for the Left and Right Mastcam-Z 8
Scene 7 for the Left and Right Mastcam-Z 10
Scene 8 for the Left and Right Mastcam-Z 11
Scene 9 for the Left and Right Mastcam-Z 12
Scene 10 for the Left Mastcam-Z 14
Scene 10 for the Right Mastcam-Z 15
SHUTDOWN PROCEDURE 16

Test Description

Excerpt from the Calibration Plan 4.7

The objective of this test is to image well-characterized bar targets at multiple focus and zoom positions in order to characterize the Modulation Transfer Function (MTF) and depth of field of each camera. Targets should be imaged at ~50% full well using the Bayer RGB (priority 1), 805 nm, (priority 2), and remaining non-solar filters (priority 3). Obtain a minimum of 3 images of each target per filter, focus, and zoom position. Multiple images are needed to reduce errors in determining target locations in the image plane.

MTF is an effective means of specifying the resolution of an optical system. Resolution is defined as the minimum feature size of an object that can be distinguished by an imaging system. The Point Spread Function (PSF) is the inverse Fourier Transform of the MTF—the PSF describes optical performance in the spatial domain while the MTF expresses optical performance in the frequency domain. Images of the bar targets and knife edge or point source targets at various zoom and focus positions will be used to determine PSF, depth of field, and MTF. The bar target shall consist of a chart containing horizontal, vertical, and diagonal lines and bars of varying thicknesses as well as circular dots of various sub- and super-pixel sizes.

In addition to determining the optical performance of the optomechanical assemblies, the images collected during MTF/PSF calibration will also be used to determine the numerical value and repeatability of the stepper motor counts for the Hall Effect sensors used to measure the position of the focus group and two moving zoom groups in the optical zoom assemblies. This will

determine the relationship between stepper motor count for each focus/zoom group, working distance, and pixel scale. Owing to thickness variations between spectral filters, focus shifts may occur and images would ideally be obtained using all non-solar filters.

Software Preparation

The software and files required for this test are prepared well in advance of test day. This checklist ensures that the following are present, debugged, and executable: (1) all fast look scripts, (2) automated header generation of all relevant camera parameters, target positioning, and metadata, (3) all camera scripts that command the camera unit, and (4) the directories/file-paths pointing to the data repositories of this specific test.

Table 1. File naming convention for the camera script prefixes and frame filenames: "AAABBBBCDD"

Code	Name	Example	Value(s)
"AAA"	Calibration Plan Section	"411" = Cal. Plan 4.1.1 chapter 4, section 1, subsection 1	471
"BBBB"	Location of test or MSSS TVAC temperature	"TAMB" = ambient test at MSSS, "TN10" = MSSS TVAC at -10C, …	TAMB
"C"	Camera unit under test	"L" = Left Mastcam-Z, "R" = Right Mastcam-Z, "E" =EQM, "C" =COTS	R/L
"DD"	Part of test	"00" = test set up, "01" = first radiance level …	00-30

1. **[D]** ____ Look up the daily calibration schedule and record the scheduled start and end time of this test on the cover page of this document. Also, fill out and double-check the other information on the cover page.
2. **[D]** ____ Ensure that all supplemental manuals are on hand.
3. **[D]** ____ Ensure that the Image Log is present and ready to use. Find and open the Google Sheets file "Image_Log_46". The duration is 2 minutes. There is a link on the Wiki.

4. [V] [illegible] Check that all *Calgorithms* fast-look and validation scripts are present, up-to-date, and ready to analyze test output. Find and open the "Geometric_Calibration_47_Validation" Jupyter notebook. There is a link on the Wiki.
5. [O] [illegible] Check that all camera scripts required for this test are present, up-to-date and ready to command the ground support equipment (GSE). These are,
   - **466TAMBR00 - 466TAMBR10, 471TAMBR00 - 471TAMBR27**
   - **466TAMBL00 - 466TAMBL10, 471TAMBL00 - 471TAMBL27**
6. [O,V,D, L] Notes:

____

____

____

Hardware Installation

This procedure is for the ambient cleanroom testing at MSSS. Figure 1 shows the nominal layout of the cleanroom, workspace, Mastcam-Zs, ground support equipment (GSE), targets, sources, and other equipment necessary.

Figure 1. MSSS Floor Plan for Geometric Testing in the cleanroom.

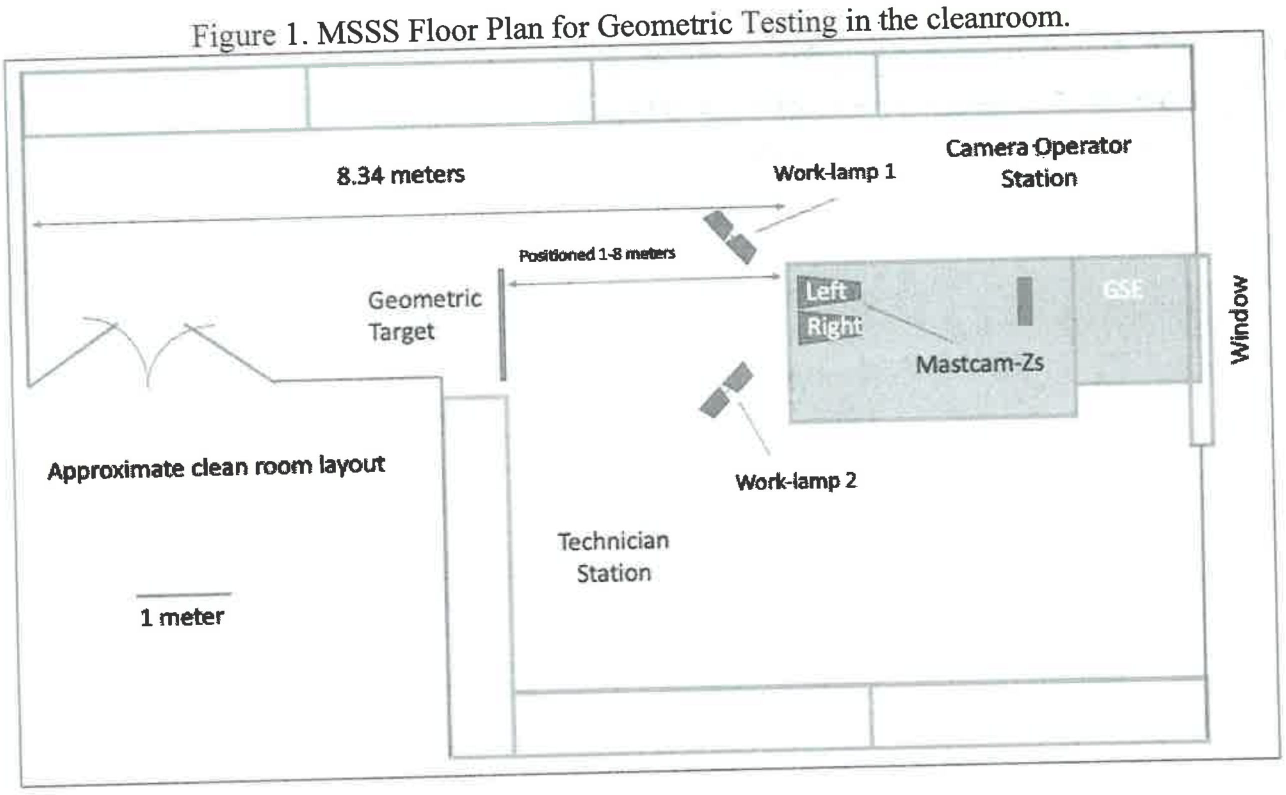

7. [T,O, L] CO Ensure that all personnel in the cleanroom are following the cleanroom practices for electrostatic discharge, proper clothing, and other safety concerns.
8. [T] CO Double check that ionized air is flowing over the Mastcam-Zs.
9. [T] CO Install the blue and infrared bright lamps and position them in front of the geometric target and out of the camera's field of view (FOV). Power them on and optimally position them.
10. [O,T] CO Ensure that the camera unit and GSE wires are secure, kink-free, and do not present tripping hazards when the lights are turned off.
11. [O,D] CO Check the camera temperature and ensure nominal operation.
12. [D] CO Record the following environmental information:
    - Cleanroom temperature N/A pressure _______ humidity _______
13. [O,D, L] Notes:

POWER ON R CAMERA @ 1815 → p.8

~~TEST IMAGES @ 24 mm AT [illegible] DISTANCE~~

26 3.17 m

Table 2. The nominal target placement scenes for the geometric testing.

Scenes	Target Placements	Notes
**Scene 6**	JPL 40x40 dot target about 3 meters centered on the 26mm boresight	
**Scene 7**	Large Star_ (Side A) target about 2 meters centered on the 34mm boresight	
**Scene 8**	Large Star_ (Side A) target about 3 meters centered on the 63mm boresight	
**Scene 9**	Large Star_ (Side A) target about 6 meters centered on the 100mm boresight	
**Scene 10**	Large MTF_SN007 (Side A) target about 3 meters centered on the ~~63~~34mm boresight	

RH: 58% @ 18:45
T: 67.9°

Date 5/7 Time 18:40 Initials CB

Scene 6 for the Left and Right Mastcam-Z

14. [M,T] ____ Position the 40x40 dot geometric target about 3 meters away centered on the Mastcam-Zs' shared boresights.
15. [T] ____ Install the diffuse blue and infrared bright illumination and turn them on.
16. [D, L] Notes: ~~TAKE TEST IMAGE 26mm @~~ ~~[illegible]~~ 3.171 m DISTANCE ~~FOCUS~~ R0 FILTER 466 TAMBR00, AUTOEXP ON
 - POWER ON L CAM - 26 mm @ 3m FOCUS 466 TAMBL00
 • ADJUST IMAGE CENTER & RETRY w/ 300 msec EXPOSURE
 • AGAIN... GOOD POSITION
 • LIGHTS ON
17. [O,T] ____ Load and execute the script 466TAMBL~~10~~ 9, ~~10 for 63mm~~ which captures Z-stacks of 16 focus distances (from 1 meter to infinity) for each non-solar filter with the ~~63~~ 34mm focal length. Insert note "TARGET=DOT40". The estimated duration is 15 minutes. (18:59) DONE FOR BOTH L + R (BL09, BR09)
18. ~~[O,T] ____ Load and execute the script 466TAMBR10 BL, which captures Z-stacks of 16 focus distances (from 1 meter to infinity) for each non-solar filter with the 63mm focal length. Insert note "TARGET=DOT40". The estimated duration is 15 minutes.~~ SKIP
19. [D] ____ Record image names and parameters in Image Log.
20. [D, L] Notes: DECIDED AFTER STARTING 17 THAT 21/22 HIGHER PRIORITY, SO SKIPPED 18 AND 20b

 → ~~ALSO DONE WITH 100mm FOCAL 466TAMB(L/R)11~~ SKIP

20b: → ~~TEST IMAGE TO CHECK FOR SPECULAR REFLECTION ON L & R 466TAMB L/R 00~~ SKIP

Date 5/7 Time 18:40 Initials [illegible]

ALEX: 607-793-7531

IN PARALLEL

21. [O,T] ____ Load and execute the script **466TAMBL06**, which captures Z-stacks of 16 focus distances (from 1 meter to infinity) for filter 0 with seven focal lengths. Insert note "TARGET=DOT40". The estimated duration is 15 minutes. → STARTS AT 26mm
22. [O,T] ____ Load and execute the script **466TAMBR06**, which captures Z-stacks of 16 focus distances (from 1 meter to infinity) for filter 0 with seven focal lengths. Insert note "TARGET=DOT40".". The estimated duration is 15 minutes.
23. **[D]** ____ Record image names and parameters in Image Log.
24. [D, L] Notes: DISTANCE TO TARGET REMAINS SAME

T_L=24.7° T_R=25.2° → AE=100. PROBLEMS W/ SATURATION OWING TO AE DONE ON OUT OF FOCUS IMAGES.

DECISION: ADJUST VIA MANUAL EXPOSURE OF 20msec AND REDONE. THIS WAS DONE AND

T_L=24.? T_R=25.4 → RESULTS LOOKED GOOD.

SKIP

25. [O,T] ____ ~~If time permits, load and execute the script **466TAMBL07**, which seven focal lengths in reverse order (110, 100, …) for 3 meter focus with 0 filter. Insert note "TARGET=DOT40". The estimated duration is 5 minutes.~~

SKIP

26. [O,T] ____ ~~If time permits, load and execute the script **466TAMBR07**, which seven focal lengths in reverse order (110, 100, …) for 3 meter focus with 0 filter. Insert note "TARGET=DOT40". The estimated duration is 5 minutes.~~
27. **[D]** ____ Record image names and parameters in Image Log.
28. [D, L] Notes: SKIPPED TO SAVE TIME

Skip

Scene 7 for the Left and Right Mastcam-Z

29. **[M,T]** ____ Position the Star target to Scene 7 as described in Table 2.
30. **[M]** ____ Measure and record the location of the Star target.
____
____
____

31. **[D]** ____ Record the following temperatures:
    - Left Camera CCD temp ____________
    - Right Camera CCD temp ____________
32. **[D,T]** ____ Take pictures the geometric target position and the whole test/GSE set-up.
33. **[O]** ____ Capture a tests frames at 34mm with filter 0 of both cameras, and rsync data to the validator. Use prefixes **471TAMBL00.**
34. **[O]** ____ Capture a tests frames at 34mm with filter 0 of both cameras, and rsync data to the validator. Use prefixes **471TAMBR00.**

35. **[O]** ____ Edit the script **471TAMBL25** with 16 focus positions near filter 0's best focus. Remove filters 1-6. Insert note "TARGET= STAR".
36. **[D, L]** Notes: ____
____
____

37. **[O]** ____ Edit the script **471TAMBR25** with 16 focus positions near filter 0's best focus. Remove filters 1-6. Insert note "TARGET= STAR".
38. **[D, L]** Notes: ____
____
____

39. **[O,V, L]** Execute the edited script **471TAMBL25** for the manual z-stack focus values around filter 0's best focus found by the previous autofocus. Insert note "TARGET= STAR". The estimated duration is 16 minutes.
40. **[O,V, L]** Execute the edited script **471TAMBR25** for the manual z-stack focus values around filter 0's best focus found by the previous autofocus. Insert note "TARGET= STAR". The estimated duration is 16 minutes.

41. **[D]** Record image names and parameters in Image Log.

This one is correct

Table 2. The nominal target placement scenes for the geometric testing.

Scenes	Target Placements	Notes
Scene 6	JPL 40x40 dot target about 3 meters centered on the 26mm boresight	
Scene 7	Large Star (Side A) target about 2 meters centered on the **Left** 34mm boresight	
Scene 8	Large Star (Side A) target about 2 meters centered on the **Right** 34mm boresight	
Scene 9	Large MTF_SN007 (Side A) target about 3 meters centered on the **Left** 34mm boresight	
Scene 10	Large MTF_SN007 (Side A) target about 3 meters centered on the **Right** 34mm boresight	Skipped

Date ______ Time ______ Initials ______

Scene 6 for the Left and Right Mastcam-Zs

Skip

14. **[M,T]** ____ Position the 40x40 dot geometric target about 3 meters away centered on the Mastcam-Zs' shared boresights.
15. **[T]** ____ Install the diffuse blue and infrared bright illumination and turn them on.
16. **[O,T]** ____ Capture a test frames at 34mm with filter 0. Center the target on the boresight and take test images as needed. Use prefix **471TAMBL00**.
17. **[O,T]** ____ Capture a test frames at 34mm with filter 0. Center the target on the boresight and take test images as needed. Use prefix **471TAMBR00**.
18. **[D, L]** Notes:____

19. **[O,T]** ____ Load and execute the script **466TAMBL06**, which captures Z-stacks of 16 focus distances (from 1 meter to infinity) for filter 0 with seven focal lengths. Insert note "TARGET=DOT40". The estimated duration is 15 minutes.
20. **[O,T]** ____ Load and execute the script **466TAMBR06**, which captures Z-stacks of 16 focus distances (from 1 meter to infinity) for filter 0 with seven focal lengths. Insert note "TARGET=DOT40".". The estimated duration is 15 minutes.
21. **[D]** ____ Record image names and parameters in Image Log.
22. **[D, L]** Notes:____

Skip

23. [O,T] ____ Load and execute the script **466TAMBL~~10~~** 09, which captures Z-stacks of 16 focus distances (from 1 meter to infinity) for each non-solar filter with the ~~63~~ 34**mm** focal length. Insert note "TARGET=DOT40". The estimated duration is 15 minutes.
24. [O,T] ____ Load and execute the script **466TAMBR~~10~~** 09, which captures Z-stacks of 16 focus distances (from 1 meter to infinity) for each non-solar filter with the ~~63~~ 34**mm** focal length. Insert note "TARGET=DOT40". The estimated duration is 15 minutes.
25. **[D]** ____ Record image names and parameters in Image Log.
26. [D, L] Notes:______________________________

27. [O,T] ____ If time permits, load and execute the script **466TAMBL07**, which seven focal lengths in reverse order (110, 100, …) for 3 meter focus with 0 filter. Insert note "TARGET=DOT40". The estimated duration is 5 minutes.
28. [O,T] ____ If time permits, load and execute the script **466TAMBR07**, which seven focal lengths in reverse order (110, 100, …) for 3 meter focus with 0 filter. Insert note "TARGET=DOT40". The estimated duration is 5 minutes.
29. **[D]** ____ Record image names and parameters in Image Log.
30. [D, L] Notes:______________________________

Date 5/7 Time 21 Initials CS

Scene 7 for the Left Mastcam-Z

31. [M,T] ____ Position the Star target to Scene 7 as described in Table 2.
32. [D, L] Notes: TARGET AT ~~[illegible]~~ 2.40 m; ⊥ TO CAMERAS VERIFIED

33. [D] ____ Record the following temperatures:
    - Left Camera CCD temp 24.7°
    - Right Camera CCD temp 25.0'
34. [D,T] ____ Take pictures the geometric target position and the whole test/GSE set-up. ✓ PHOTOS 209 210
35. [O,T] ____ Capture a test frame at 34mm with filter 0. Center the star on the boresight and take test images as needed. Use prefix **471TAMBL00.** 35b — 63 mm
36. [O,T] ____ Capture a test frame at 100mm with filter 0 to make sure that the star's center is aligned with the boresight. Use prefix **471TAMBL00.**

36 → 37. [O,V, L] Edit script **471TAMBL30** for **100mm** w/ NEW FOCUS and execute to capture a manual z-stack focus values around filter 0's best focus for all filters. Insert note "TARGET= STAR". The estimated duration is 12 minutes. 21:00

35b → 38. [O,V, L] Edit script **471TAMBL30** for **63mm** w/ NEW FOCUS and execute to capture a manual z-stack focus values around filter 0's best focus for all filters. Insert note "TARGET= STAR". The estimated duration is 12 minutes.

35 → 39. [O,V, L] Edit script **471TAMBL30** for **34mm** w/ NEW FOCUS and execute to capture a manual z-stack focus values around filter 0's best focus for all filters. Insert note "TARGET= STAR". The estimated duration is 12 minutes.

40. [D] Record image names and parameters in Image Log.
41. [V] Run fast-look script to verify that the required data were obtained.
42. [D, L] Notes: DID 100 mm FIRST; TOOK 3 TRIES TO GET CENTERED. NEW FOCUS POS^n = 2508 AE 155 REPLACED W/ AE 100. HAD TO TURN LIGHTS ON AND RE-RUN AFTER ABORTING AFTER 4 MIN; 2ND ATTEMPT WORKED BETTER
    - 63 mm FOCUS POS^n: 2088
    - 34 mm FOCUS POS^n: -36 HOWEVER, HAD TO DELETE "MOTABS" OF 90, 72, 54 AND REPLACED 54 WITH 48; AND REMOVING 90 AND 72 FOR EACH FILTER. REASON: GSE S/W ISSUE THAT DOESN'T ALLOW MOTOR POSITIONS BETWEEN 50-102

Date 5/7 Time 21 Initials [illegible]

Scene 8 for the Right Mastcam-Z

43. [M,T] ____ Position the Star target to Scene 8 as described in Table 2.
44. [D, L] Notes: BEGIN @ 22:00
CENTER DISTANCE = 2.5 m

45. [D] ____ Record the following temperatures:
    - Left Camera CCD temp 24.3°
    - Right Camera CCD temp 24.7°
46. [D,T] ____ Take pictures the geometric target position and the whole test/GSE set-up. PHOTOS 211 212 213
47. [O,T] ____ Capture a test frame at 34mm with filter 0. Center the star on the boresight and take test images as needed. Use prefix **471TAMBR00.**
    ~~47.b - @ 63mm~~
48. [O,T] ____ Capture a test frame at 100mm with filter 0 to make sure that the star's center is aligned with the boresight. Use prefix **471TAMBR00.**

48 → 49. [O,V, L] Edit script **471TAMBR31** for **100mm** and execute to capture a manual z-stack focus values around filter 0's best focus for all filters. Insert note "TARGET= STAR". The estimated duration is 12 minutes.

47.b → 50. ~~[O,V, L] Edit script **471TAMBR31** for **63mm** and execute to capture a manual z-stack focus values around filter 0's best focus for all filters. Insert note "TARGET= STAR". The estimated duration is 12 minutes.~~ SKIP

47 → 51. [O,V, L] Edit script **471TAMBR31** for **34mm** and execute to capture a manual z-stack focus values around filter 0's best focus for all filters. Insert note "TARGET= STAR". The estimated duration is 12 minutes.

52. [D] Record image names and parameters in Image Log.
53. [V] Run fast-look script to verify that the required data were obtained.
54. [D, L] Notes: CENTERING OF 100mm - 3 TRIES. AE → 100.
COMPLETE 23:15

100 mm FOCUS POS^n = 2454
63 mm FOCUS POS^n = SKIPPED → WANT TO GET TO SR TARGETS AT EXPENSE OF "SCENE 9"
34 mm FOCUS POS^n = -12   SAME ISSUE WITH MOTOR COUNTS 50-102, SO REMOVED 96 → 108
60 → 48
78 → i = 108
Z VALUES WITH 108

NOTE: WHITE LATEX ON FLOORS, NOT FROM OPERATORS

Scene 9 for the Left Mastcam-Z RUN IN PARALLEL (L+R)

55. **[M,T]** ____ Position the ~~Star~~ MTF target to Scene 9 as described in Table 2.
56. **[D, L]** Notes: NEEDS SCUTS + HALOGEN LIGHTS
____
____

57. **[D]** ____ Record the following temperatures:
    - Left Camera CCD temp ______________
    - Right Camera CCD temp ______________
58. **[D,T]** ____ Take pictures the geometric target position and the whole test/GSE set-up.
59. **[O,T]** ____ Capture a test frame at 34mm with filter 0. Center the target on the boresight and take test images as needed. Use prefix **471TAMBL00.** AND BR00
60. **[V,T]** ____ Load the image in MTF Mapper to find the correct target position. Recapture an autofocused frame if necessary. TOLERANCE IS ~1° IN YAW/PITCH/TILT

61. **[O,V, L]** Edit script **471TAMBL30** for **34mm** AND BR31 and execute to capture a manual z-stack focus values around filter 0's best focus for all filters. Insert note "TARGET= MTF_SN007". The estimated duration is 12 minutes.

62. **[D]** Record image names and parameters in Image Log.
63. **[V]** Run fast-look script to verify that the required data were obtained.
64. **[D, L]** Notes: ____
____
____

Date 5/7 Time 23 Initials [illegible]

Scene 10 for the Left Mastcam-Z

65. [M,T] ____ Position the ~~Star~~ MTF target to Scene 10 as described in Table 2.
66. [D, L] Notes: ____

67. [D] ____ Record the following temperatures:
    - Left Camera CCD temp ____________
    - Right Camera CCD temp ____________
68. [D,T] ____ Take pictures the geometric target position and the whole test/GSE set-up.
69. [O,T] ____ Capture a test frame at 34mm with filter 0. Center the target on the boresight and take test images as needed. Use prefix **471TAMBR00.**
70. [V,T] ____ Load the image in MTF Mapper to find the correct target position. Recapture an autofocused frame if necessary.

71. [O,V, L] Edit script **471TAMBR31** for **34mm** and execute to capture a manual z-stack focus values around filter 0's best focus for all filters. Insert note "TARGET= MTF_SN007". The estimated duration is 12 minutes.

72. [D] Record image names and parameters in Image Log.
73. [V] Run fast-look script to verify that the required data were obtained.
74. [D, L] Notes: ____

Shutdown Procedure

75. [D,T] [illegible] Take pictures of the test setup.
76. [D,O] [illegible] Review entries in Image Log, GSE command log, and image headers.
77. [D, L] [illegible] Review calibration procedure and ensure that each task is initialed.
78. [D, L] Notes: ______

79. [V, L] [illegible] Before making the decision to break down the test setup, ensure that adequate data were acquired for the test requirements. See "MastcamZCalPlan" for these requirements.
80. [V] Notes: ______

Data Validator (signature) Ole B. Jensen

Date 9/5-2019 Time 12:35 AM

81. [V, L] [illegible] Give the go/no-go decision. Have enough data been acquired to fulfill test requirements? See "MastcamZCalPlan" for these requirements.
82. [D, L] [illegible] Update the Log Document. – [illegible]
83. [L] Notes: ______

Calibration Lead (signature) [illegible]

Date 5/8/19 Time 12:35

84. [O, L] JVB Ensure that the camera and GSE are in a safe state.
85. [O,D] JVB Review the Image Log with the documentarian. Exchange high-fives.
86. [O] Notes: Nothing of Notes

Camera Operator (signature) Angela Magee

Date 5/8/19 Time 12:35AM

87. [T] CD If the next test does not require the target, position it away from the chamber or bench. Otherwise, be sure not to move it. The next test is JR.
88. [T] CD Ensure that all other test equipment is safely put away.
89. [T] Notes: ______

Technician (signature) Christian Dent

Date 5/7/19 Time 23:00

90. [D, L] CD Double-check this procedure and ensure that the top of each page has valid data, time and initials.
91. [D] CD Photo-scan this document, save it on the cloud, and file the hard-copy in the Log Binder. Upload the digital pictures taken during this test in the appropriate archive on the cloud. The required links are on the Wiki.
92. [D] CD Double-check that every required cell the Image Log is accurately filled. When this is complete, print the Image Log and file it the Log Binder after this document.
93. [D] Notes: ______

Documentarian (signature) [illegible]

Date May 8 Time 12:35
