# Supplementary material for: Pre-Flight Calibration of the Mars 2020 Rover Mastcam Zoom (Mastcam-Z) Multispectral, Stereoscopic Imager
Source: Space Sci Rev. 2021 Feb 18;217(2):29. doi: 10.1007/s11214-021-00795-x (PMC7892537; doi:10.1007/s11214-021-00795-x)
Supplement: Supplementary file 1 — (ZIP 98.6 MB) [file 11214_2021_795_MOESM1_ESM.zip › CalPro_473_MTF_v2_02.pdf]

Date 4/27 Time 8:30 Initials JFB

MTF Calibration Procedure for Mastcam-Z TVAC Testing (Pro. 4.7.~~2~~ 3)

[Procedure version ~~2.21~~ v2.02, prepared by the Mastcam-Z calibration team at Cornell University.]

These measurements are performed on the camera and at the temperature designated below as specified in the Calibration Plan (Document #),

Unit Under Test:

R FM __X__ L FM __X__ EQM_____ Other__________

Test Performed at Temperature:

-35°C______ - 10°C___X___ +5°C______ Ambient ______ Other ______

These measurements are performed at,

MSSS___X___ ASU_______ Other_______

Date 4/27/2019 **Start Time** 8:45 AM **End Time** ______

Estimated Duration __2.5 hours__

Scheduled Start Time 8:30 AM **Sch. End Time**______

**Calibration Lead [L]**	Jim Bell	**Documentarian [D]**	Jim Bell
**Camera Operator [O]**	Tex Kubacki	**Technician [T]**	Christian Tate
**Data Validator [V]**	Paul Corlies	**Metrologist [M]**	Christian ~~[illegible]~~ Tate **
**Other**	ANDY Winhold		

Date 4/27 Time 830 Initials JFB

Table of Contents

MTF CALIBRATION PROCEDURE FOR MASTCAM-Z AMBIENT TVAC TESTING (PRO. 4.7.2) **1**

TEST DESCRIPTION 3

SOFTWARE PREPARATION 4

Table 1. File naming convention for the camera script prefixes and frame filenames: "AAABBBBCDD"............ 4

HARDWARE INSTALLATION .... **ERROR! BOOKMARK NOT DEFINED.**

Figure 1. ASU Floor Plan for Geometric Testing in the TVAC Chamber. The MSSS Floor Plan allows for similar target and source placements relative to the chamber window. .... ***Error! Bookmark not defined.***

Table 2. The nominal target placement scenes for the geometric testing.. 7

SCENE 1 FOR THE RIGHT MASTCAM-Z 8

Document Approval

4/27/19

Approved by James Bell Date
Mastcam-Z PI
Arizona State University

x 5/6/19

Approved by Alexander Hayes Date
Mastcam-Z Calibration Working Group
Lead, Cornell University

Approved by Justin Maki Date
Mastcam-Z Deputy PI and Investigation
Scientist, Jet Propulsion Laboratory

Approved by Christian Tate Date
Procedure Author
Cornell University

Approved by: Date

Date 4/17 Time 845 Initials JCB

Test Description

Excerpt from the Calibration Plan 4.7

The objective of this test is to image well-characterized bar targets at multiple focus and zoom positions in order to characterize the Modulation Transfer Function (MTF) and depth of field of each camera. Targets should be imaged at ~50% full well using the Bayer RGB (priority 1), 805 nm, (priority 2), and remaining non-solar filters (priority 3). Obtain a minimum of 3 images of each target per filter, focus, and zoom position. Multiple images are needed to reduce errors in determining target locations in the image plane.

MTF is an effective means of specifying the resolution of an optical system. Resolution is defined as the minimum feature size of an object that can be distinguished by an imaging system. The Point Spread Function (PSF) is the inverse Fourier Transform of the MTF—the PSF describes optical performance in the spatial domain while the MTF expresses optical performance in the frequency domain. Images of the bar targets and knife edge or point source targets at various zoom and focus positions will be used to determine PSF, depth of field, and MTF. The bar target shall consist of a chart containing horizontal, vertical, and diagonal lines and bars of varying thicknesses as well as circular dots of various sub- and super-pixel sizes.

In addition to determining the optical performance of the optomechanical assemblies, the images collected during MTF/PSF calibration will also be used to determine the numerical value and repeatability of the stepper motor counts for the Hall Effect sensors used to measure the position of the focus group and two moving zoom groups in the optical zoom assemblies. This will determine the relationship between stepper motor count for each focus/zoom group, working distance, and pixel scale. Owing to thickness variations between spectral filters, focus shifts may occur and images would ideally be obtained using all non-solar filters.

Note that the Mastcam-Z instrument has an onboard focus merge algorithm that selects and merges the best-focus portions of a scene using a focus stack (or z-stack) of multiple images (up to 16) at varying focal positions. While usually only the best-focus or merged product is saved to file, the raw frames for the focus stack can also be saved to file without appreciably increasing the observation or product generation time. For tests where images at multiple focus positions are desired, such as during MTF/PSF Calibrations, the entire focus stack of images will be written to file. Since images at multiple focus positions are acquired irrespective of whether or not they are written to memory, saving multiple focus positions per zoom setting does not require additional test time (outside of the time required to write the files to disk in the GSE). Multiple focus position images shall be saved during both Stand-Alone and ATLO MTF/PSF testing.

Date 4/7/ Time 8:45 Initials JM

Software Preparation

The software and files required for this test are prepared well in advance of test day. This checklist ensures that the following are present, debugged, and executable: (1) all fast look scripts, (2) automated header generation of all relevant camera parameters, target positioning, and metadata, (3) all camera scripts that command the camera unit, and (4) the directories/file-paths pointing to the data repositories of this specific test.

Table 1. File naming convention for the camera script prefixes and frame filenames: "AAABBBBCDD"

Code	Name	Example	Value(s)
"AAA"	Calibration Plan Section	"411" = Cal. Plan 4.1.1 chapter 4, section 1, subsection 1	473, 492
"BBBB"	Location of test or ASU TVAC temperature	"MSSS" = test at MSSS, "ATLO" = test at JPL ATLO, "TN10" = ASU TVAC -10C, …	TN10
"C"	Camera unit under test	"L" = Left Mastcam-Z, "R" = Right Mastcam-Z, "E" =EQM, "C" =COTS	R/L
"DD"	Part of test	"00" = test set up, "01" = first radiance level …	00-12

1. **[D]** ✓ Look up the daily calibration schedule and record the scheduled start and end time of this test on the cover page of this document. Also, fill out and double-check the other information on the cover page.
2. **[D]** ✓ Ensure that all supplemental manuals are on hand. These are,
   - Validator_Manual, Documentarian_Manual,
   - MastcamZCalPlan
3. **[D]** ✓ Ensure that the Image Log is present and ready to use. Find and open the Google Sheets file "Image_Log_46". There is a link on the Wiki. (47)
4. **[V]** ✓ Check that all *Calgorithms* fast-look and validation scripts are present, up-to-date, and ready to analyze test output. Find and open the "Geometric_Calibration_46_Validation" Jupyter notebook. There is a link on the Wiki.
5. **[O]** ✓ Check that all camera scripts required for this test are present, up-to-date and ready to command the ground support equipment (GSE). These are,

- **473TN10R00 - 473TN10R06, 492TN10R01 - 492TN10R12**
- **473TN10L00 - 473TN10L06, 492TN10L01 - 492TN10L12**

6. **[O,V,D, L]** Notes:
   All good!

Hardware Installation

This procedure is for the ambient TVAC chamber testing at MSSS. Figure 1 shows the nominal layout of the TVAC chamber, workspace, Mastcam-Zs, ground support equipment (GSE), targets, sources, and other equipment necessary for this test if it happens at ASU. Although MSSS' cleanroom is different than ASU's, the placement of the targets and sources relative to the chamber window is similar.

Figure 1. ASU Floor Plan for Geometric Testing in the TVAC Chamber. The MSSS Floor Plan allows for similar target and source placements relative to the chamber window.

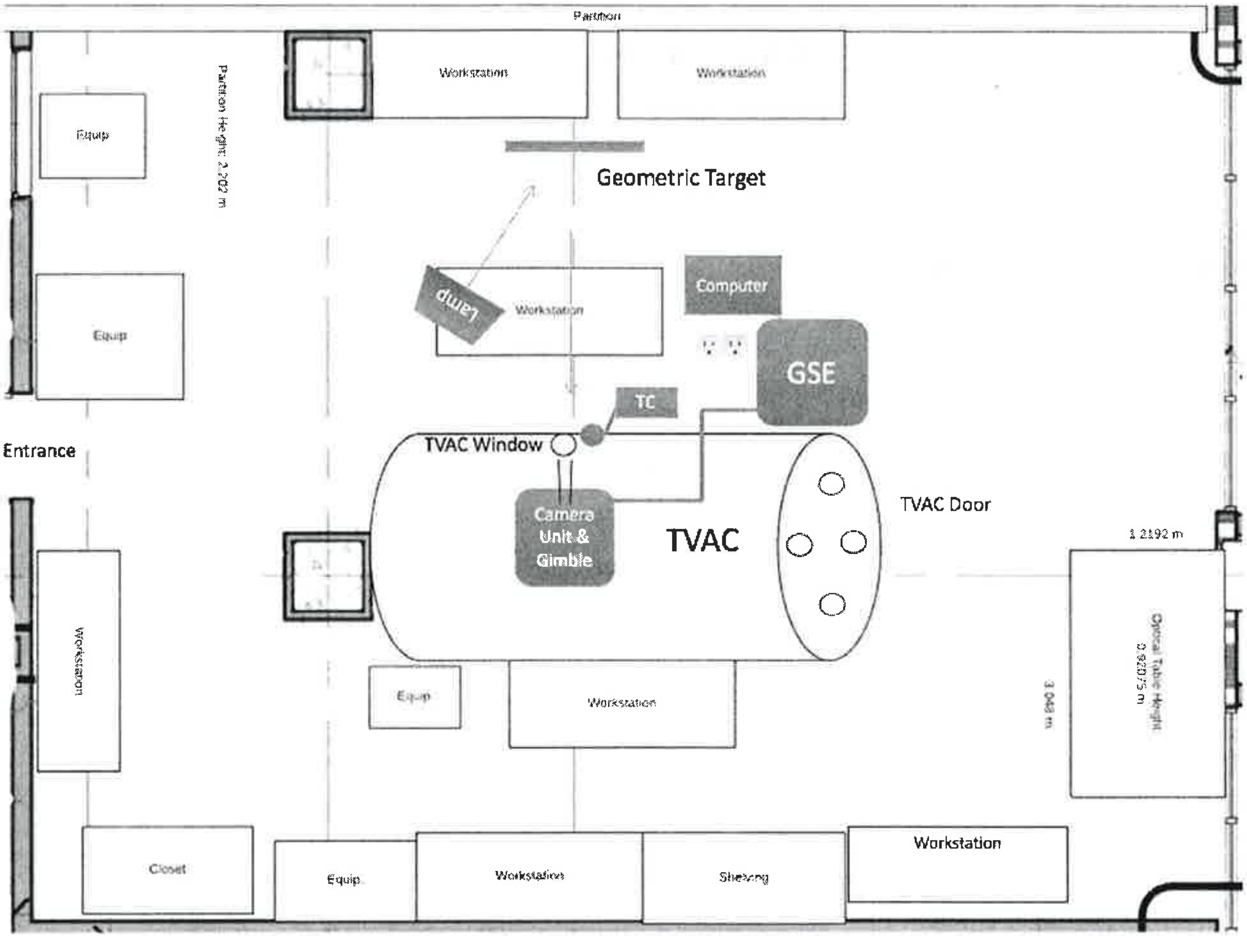

7. **[T,O, L]** ✓ Ensure that all personnel in the cleanroom are following the cleanroom practices for electrostatic discharge, proper clothing, and other safety concerns. See "ESD_Manual" and "Cleanroom_Manual".
8. **[T]** ✓ Double check that nitrogen is flowing over the Mastcam-Zs or the window port.
9. **[O,T]** ✓ If not already done, mate the Right Mastcam-Z into the GSE. Follow the procedure in "MastcamZ_GSE_Manual".
10. **[T]** ✓ Verify that the thermocouples are turned on and properly reading out.
11. **[T]** ✓ Install the lamps and position them 1 meter from the geometric target out of the camera's field of view (FOV). Power them on.

Date 4/27 Time 850 Initials JFB

12. **[O,T]** ✓ Ensure that the camera unit and GSE wires are secure, kink-free, and do not present tripping hazards when the lights are turned off.
13. **[O,D]** ✓ Check the camera temperature and ensure nominal operation.
14. **[D]** [illegible] Record the following environmental information:
    - Cleanroom temperature 26.8 pressure AMB humidity 38% [illegible]
15. **[[redacted],D, L]** Notes:

photo of setup by Christina
~~[illegible]~~ camera Temp -7.7, -5.7 L,R

Table 2. The nominal target placement scenes for the geometric testing.

	Target Placements	Notes
**Scene 1**	MTF_SN005 (Side A) at 2 meters centered on the 63mm boresight	1.65m from window to target
**Scene 2**	MTF_SN005 (Side A) at 3 meters centered on the 100mm boresight	2.92m distance
**Scene 3**	MTF_SN007 (Side A) at 3 meters centered on the 34mm boresight	
**Scene 4**	MTF_SN007 (Side A) at 2 meters centered on the 26mm boresight	NIX – out of time

1.65

Date 4/27 Time 920 Initials JFB

Scene 1 for the Right Mastcam-Z

✓ 16. **[M,T]** Position the MTF target to Scene 1 as described in Table 2.
✓ 17. **[M]** Measure and record the location of the MTF target.

Tweaking, Taking Test images

Distance 1.65 m

✓ 18. **[D]** Record climate information:

- TVAC temp -10°C Port temp N/A
- Camera CCD temp -3.4°C Optics temp N/A

✓ 19. **[D,T]** Take digital pictures of the setup and MTF target.
✓ 20. **[O]** Capture one autofocused frame at 63mm with filter 0, and rsync data to the validator.
✓ 21. **[V,T]** Load the image in MTF Mapper to find the correct target position. Recapture an autofocused frame if necessary.

Skipping due to time X 22. **[O]** Load and execute camera script **473TN10R03**, which autofocus and takes 3 frames at 63mm focal length with filter 0. Insert note "TARGET=MTF_SN005".
✓ 23. **[O]** Load and execute camera script **492TN10R06**, which autofocus and takes three frames at 63mm focal length with filters 0-6. Insert note * "TARGET=MTF_SN005". The duration is 7 minutes.
✓ 24. **[D]** Record image names and parameters in Image Log.
✓ 25. **[V]** Run fast-look script to verify that required data were obtained.
✓ 26. **[D, L]** Notes:

Script Aborted first time – Target Bumped

Camera Temp -2.94 for 2nd run of script.

Procedure change

* Change Insert Note To Before Load and execute...

– Auto focus failed on filter 6 (1000 nm)

→ Re-running with new af parameters for filter 6 only

– range was too small for longest-wave filter

– GSE operators homed mechanisms, powered off Right camera at 9:38 AM

Date 4/27 Time 9:40 Initials JFB

Scene 1 for the Left Mastcam-Z

✓27. **[M,T]** Position the MTF target to Scene 1 as described in Table 2.

28. **[M]** Measure and record the location of the MTF target.

Taking Test Images
Starting camera Temp = -10°C
Distance = 1.65 m

✓29. **[D]** Record climate information:

- TVAC temp -10°C Port temp N/A
- Camera CCD temp -10°C Optics temp N/A

✓30. **[D,T]** Take digital pictures of the setup and MTF target.

✓31. **[O]** Capture one autofocused frame at 63mm with filter 0, and rsync data to the validator.

✓32. **[V,T]** Load the image in MTF Mapper to find the correct target position. Recapture an autofocused frame if necessary.

SKIP X 33. **[O]** Load and execute camera script **473TN10L03**, which autofocus and takes 3 frames at 63mm focal length with filter 0. Insert note "TARGET=MTF_SN005".

✓34. **[O]** Load and execute camera script **492TN10L06**, which autofocus and takes three frames at 63mm focal length with filters 0-6. Insert note "TARGET=MTF_SN005". The duration is 7 minutes.

✓35. **[D]** Record image names and parameters in Image Log.

36. **[V]** Run fast-look script to verify that required data were obtained.

37. **[D, L]** Notes:

Script started at 9:54 am, Camera Temp at -6.0°C
finished at 10:01 am -5.2°C

All files transferred
Quick-look Looks good!

Date 4/27 Time 10:08 Initials SCD

Scene 2 for the Left Mastcam-Z

✓ 38. [M,T] Position the MTF target to Scene 2 as described in Table 2.

✓ 39. [M] Measure and record the location of the MTF target.

Target is 2.92m distance

CT

✓ 40. [D] Record climate information:

- TVAC temp -10°C Port temp N/A
- Camera CCD temp -5.41 Optics temp N/A

✓ 41. [D,T] Take digital pictures of the setup and MTF target.

✓ 42. [0] Capture one autofocused frame at 100mm with filter 0, and rsync data to the validator.

✓ 43. [V,T] Load the image in MTF Mapper to find the correct target position. Recapture an autofocused frame if necessary.

SKIP ✗ 44. [0] Load and execute camera script **473TN10L04**, which autofocus and takes 3 frames at 100mm focal length with filter 0. Insert note "TARGET=MTF_SN005".

✓ 45. [0] Load and execute camera script **492TN10L07**, which autofocus and takes three frames at 100mm focal length with filters 0-6. Insert note "TARGET=MTF_SN005". The duration is 7 minutes.

✓ 46. [D] Record image names and parameters in Image Log.

✓ 47. [V] Run fast-look script to verify that required data were obtained.

✓ 48. [D, L] Notes:

Script started at 10:17. Aborted, Target bumped

Script re-started at 10:25 camera at -5.0

script ~~completed at~~ aborted at 10:32, autofocus failed on filter 6.

⇒ af parameters expanded, filter 6 part of script re-run 10:33

failed again. re-setting parameters, re-running 10:36

failed again, manually diagnosing focus

af not working well for 440nm filter

focus set to 2730 manually for 5-stack on filter 6

filter 6, re-run script ~~at~~ without af, start at 11:03

Left camera homed and powered off at 11:07 Am

Date 4/27 Time 11:08 Initials JFB

Scene 2 for the Right Mastcam-Z

✓49. **[M,T]** Position the MTF target to Scene 2 as described in Table 2.

✓50. **[M]** Measure and record the location of the MTF target.

Distance to Target = 3.01 m

✓51. **[D]** ~~Record~~ CK climate information:

- TVAC temp -10°C Port temp N/A
- Camera CCD temp -4.6°C Optics temp N/A

✓52. **[D,T]** Take digital pictures of the setup and MTF target.

✓53. **[O]** Capture one autofocused frame at 100mm with filter 0, and rsync data to the validator.

✓54. **[V,T]** Load the image in MTF Mapper to find the correct target position. Recapture an autofocused frame if necessary.

SKIP ✗ ~~55. **[O]** Load and execute camera script **473TN10R04**, which autofocus and takes 3 frames at 100mm focal length with filter 0. Insert note "TARGET=MTF_SN005".~~

56. [[redacted]] Load and execute camera script **492TN10R07**, which autofocus and takes three frames at 100mm focal length with filters 0-6. Insert note "TARGET=MTF_SN005". The duration is 7 minutes.

✓57. **[D]** Record image names and parameters in Image Log.

58. **[V]** Run fast-look script to verify that required data were obtained.

59. **[D, L]** Notes: Started script at 12:00 Camera at -2.5°C

autofocus not behaving well. Tex calling Caplinger... (11:20 am)

Caplinger assessing (11:30 am)

hypothesis: Too much scattered light onto Target from people moving around the room. NEW RULE: Everyone either seated, or out of the room, when acquiring data.

⇒ working well with folks in room stationary!

Date 4/27 Time 12:30 Initials JFB

Scene 3 for the Right Mastcam-Z

✓ 60. [M,T] Position the MTF target to Scene 3 as described in Table 2.

✓ 61. [M] Measure and record the location of the MTF target.

2.95m To Target

62. [D] Record climate information:
    - TVAC temp -10°C Port temp N/A
    - Camera CCD temp -3.1°C Optics temp N/A

✓ 63. [D,T] Take digital pictures of the setup and MTF target.

✓ 64. [O] Capture one autofocused frame at 34mm with filter 0, and rsync data to the validator.

✓ 65. [V,T] Load the image in MTF Mapper to find the correct target position. Recapture an autofocused frame if necessary.

SKIP X 66. [[redacted]] Load and execute camera script **473TN10R02**, which autofocus and takes 3 frames at 34mm focal length with filter 0. Insert note "TARGET=MTF_SN007".

67. [[redacted]] Load and execute camera script **492TN10R05**, which autofocus and takes three frames at 34mm focal length with filters 0-6. Insert note "TARGET=MTF_SN007". The duration is 7 minutes.

✓ 68. [D] Record image names and parameters in Image Log.

69. [V] Run fast-look script to verify that required data were obtained.

70. [D, L] Notes:

Started script at 12:36 pm

script failed af on filter 4 → need to widen af range.
re-started at 12:36 pm, camera at -3.0°C

Date 4/21 Time 1:00p Initials JAB

Scene 3 for the Left Mastcam-Z

✓ 71. [M,T] Position the MTF target to Scene 3 as described in Table 2.

72. [M] Measure and record the location of the MTF target.

Distance ≈ 2.89m

73. [D] Record climate information:

- TVAC temp -10°C ~~N/A~~ Port temp N/A
- Camera CCD temp - 5.6 °C Optics temp N/A

✓ 74. [D,T] Take digital pictures of the setup and MTF target.

✓ 75. [O] Capture one autofocused frame at 34mm with filter 0, and rsync data to the validator.

✓ 76. [V,T] Load the image in MTF Mapper to find the correct target position. Recapture an autofocused frame if necessary.

SKIP X 77. [O] Load and execute camera script **473TN10L02**, which autofocus and takes 3 frames at 34mm focal length with filter 0. Insert note "TARGET=MTF_SN007".

✓ 78. [O] Load and execute camera script **492TN10L05**, which autofocus and takes three frames at 34mm focal length with filters 0-6. Insert note "TARGET=MTF_SN007". The duration is 7 minutes.

79. [D] Record image names and parameters in Image Log.

80. [V] Run fast-look script to verify that required data were obtained.

81. [D, L] Notes:

script started at 1:05pm, camera -5.6°C

script completed at 1:15pm -4.1°C

Validated!

Date 4/27 Time 1p Initials JFB

Scene 4 for the Left Mastcam-Z

Skip

82. **[M,T]** Position the MTF target to Scene 4 as described in Table 2.
83. **[M]** Measure and record the location of the MTF target.

____

____

____

84. **[D]** Record climate information:
    - TVAC temp __________ Port temp __________
    - Camera CCD temp __________ Optics temp __________
85. **[D,T]** Take digital pictures of the setup and MTF target.
86. **[O]** Capture one autofocused frame at 26mm with filter 0, and rsync data to the validator.
87. **[V,T]** Load the image in MTF Mapper to find the correct target position. Recapture an autofocused frame if necessary.
88. **[O]** Load and execute camera script **473TN10L02**, which autofocus and takes 3 frames at 26mm focal length with filter 0. Insert note "TARGET=MTF_SN07".
89. **[O]** Load and execute camera script **492TN10L05**, which autofocus and takes three frames at 26mm focal length with filters 0-6. Insert note "TARGET=MTF_SN007". The duration is 7 minutes.
90. **[D]** Record image names and parameters in Image Log.
91. **[V]** Run fast-look script to verify that required data were obtained.
92. **[D, L]** Notes: ____________________________________

____

____

Date 4/21 Time ______ Initials JKL

Scene 4 for the Right Mastcam-Z

Skip

93. [M,T] Position the MTF target to Scene 4 as described in Table 2.
94. [M] Measure and record the location of the MTF target.

____
____
____

95. [D] Record climate information:
    - TVAC temp ______________ Port temp ______________
    - Camera CCD temp ______________ Optics temp ______________
96. [D,T] Take digital pictures of the setup and MTF target.
97. [O] Capture one autofocused frame at 26mm with filter 0, and rsync data to the validator.
98. [V,T] Load the image in MTF Mapper to find the correct target position. Recapture an autofocused frame if necessary.
99. [O] Load and execute camera script **473TN10R02**, which autofocus and takes 3 frames at 26mm focal length with filter 0. Insert note "TARGET=MTF_SN007".
100. [O] Load and execute camera script **492TN10R05**, which autofocus and takes three frames at 26mm focal length with filters 0-6. Insert note "TARGET=MTF_SN007". The duration is 7 minutes.
101. [D] Record image names and parameters in Image Log.
102. [V] Run fast-look script to verify that required data were obtained.
103. [D, L] Notes: ____

____
____

Date 4/27 Time JFB Initials 1:15pm

Shutdown Procedure

✓1. **[D,T]** CB Take digital pictures of this page and the test setup.
✓ 2. **[D,O]** CB Review entries in Image Log, GSE command log, and image headers.
✓ 3. **[D, L]** CB Review calibration procedure and ensure that each task is initialed.
4. **[D, L]** Notes: ______

✓5. **[V, L]** JFB Before making the decision to break down the test setup, ensure that adequate data were acquired for the test requirements. See "MastcamZCalPlan" for these requirements.
6. **[V]** Notes: ______

Data Validator (signature) [signature]

Date 4/27/19 Time 1:15 pm

✓ 7. **[V, L]** JFB Give the go/no-go decision. Have enough data been acquired to fulfill test requirements? See "MastcamZCalPlan" for these requirements.
8. **[D, L]** CB Update the Log Document.
9. **[L]** Notes: ______

Calibration Lead (signature) [signature]

Date 4/27/19 Time 1:20pm

Date 4/27 Time 1:20 Initials JFB

✓10. [O, L] ✓ Ensure that the camera and GSE are in a safe state.

11. [O,D] CG Review the Image Log with the documentarian. Exchange high-fives. ✓

12. [O] Notes: ____

Camera Operator (signature) [signature]

Date 4/27 Time 1:20

13. [T] ✓ If the next test does not require the target, position it away from the chamber or bench. Otherwise, be sure not to move it. The next test is INT. Sphere.

14. [T] ✓ Ensure that all other test equipment is safely put away.

15. [T] Notes: ____

Technician (signature) [signature]

Date 4-27 Time 1:45

✓16. [D, L] ✓ Double-check this procedure and ensure that the top of each page has valid data, time and initials.

17. [D] CG Photo-scan this document, save it on the cloud, and file the hard-copy in the Log Binder. Upload the digital pictures taken during this test in the appropriate archive on the cloud. The required links are on the Wiki.

18. [D] ✓ Double-check that every required cell the Image Log is accurately filled. When this is complete, print the Image Log and file it the Log Binder after this document.

19. [D] Notes: ____

Documentarian (signature) [signature]

Date 4-27 Time 1:22
