# Supplementary material for: Pre-Flight Calibration of the Mars 2020 Rover Mastcam Zoom (Mastcam-Z) Multispectral, Stereoscopic Imager
Source: Space Sci Rev. 2021 Feb 18;217(2):29. doi: 10.1007/s11214-021-00795-x (PMC7892537; doi:10.1007/s11214-021-00795-x)
Supplement: Supplementary file 1 — (ZIP 98.6 MB) [file 11214_2021_795_MOESM1_ESM.zip › CalPro_491_Sample_Observations_v2_03.pdf]

Date 5/8 Time 3:39p Initials BH

Geological and EPO Imaging for the Mastcam-Zs at Ambient Cleanroom Testing (Pro. 4.9.1)

[Procedure version 2.03, prepared by the Mastcam-Z calibration team at Cornell University]

These measurements are performed on the camera and at the temperature designated below as specified in the Calibration Plan (Document #),

Unit Under Test:

R FM X **L FM** X **EQM**____ **Other**____

Test Performed at Temperature:

-35°C____ **- 10°C**____ **+5°C**____ **Ambient** X **Other** ____

These measurements are performed at,

MSSS X **ASU**____ **Other**____

Date 5/8/2019 **Start Time** 8:15 am (post tag-up) **End Time** 3:00 pm

Estimated Duration 8.0 hours

Scheduled Start Time 7:30 **Sch. End Time** 4:00pm

Calibration Lead [L] Briony Horgan **Documentarian [D]** Rob Sullivan

Camera Operator [O] Tex Kubacki / Elsa Jensen **Technician [T]** Chris Donaldson/Andy Winhold

Data Validator [V] Paul Corlies **Metrologist [M]** Justin Maki

Other Jim Bell, Noel Scudder, Christian Tate, Jeff Johnson, Alexis Parkinson

Date 3/8/19 Time 3:39p Initials BH

44,729

Change Log

Version	Name	Change
v1_01<br>8 Nov. 2018	C. Tate	(first draft)
v1_04<br>7 Dec. 2018	C. Tate	Procedure edits after EQM testing
v2_03<br>8 May 2018	C. Tate	Approved version prior to FM testing

Document Approval

5/8/19
Approved by James Bell — Date
Mastcam-Z PI
Arizona State University

Approved by Alexander Hayes — Date
Mastcam-Z Calibration Working Group
Lead, Cornell University

5/8/19
Approved by Justin Maki — Date
Mastcam-Z Deputy PI and Investigation
Scientist, Jet Propulsion Laboratory

5/8/19
Approved by: — Date
Jeff Johnson
Mastcam-Z Co-Investigator, APL

5/8/19
Approved by Briony Horgan — Date
MCZ Co-I, Purdue

5/8/19
Approved by Christian Tate — Date
Procedure Author
Cornell University

Date 3/8/19 Time 3:39p Initials BH

Software Preparation

The software and files required for this test are prepared well in advance of test day. This checklist ensures that the following are present, debugged, and executable: (1) all fast look scripts, (2) automated header generation of all relevant camera parameters, target positioning, and metadata, (3) all camera scripts that command the camera unit, and (4) the directories/file-paths pointing to the data repositories of this specific test.

Table 1. File naming convention for the camera script prefixes and frame filenames: "AAABBBBCDD"

Code	Name	Example	Value(s)
"AAA"	Calibration Plan Section	"411" = Cal. Plan 4.1.1 chapter 4, section 1, subsection 1	465, 471, 491
"BBBB"	Location of test or MSSS temperature	"TAMB" = ambient test at MSSS, "TN10" = MSSS TVAC at -10C, …	TAMB
"C"	Camera unit under test	"L" = Left Mastcam-Z, "R" = Right Mastcam-Z, "E" =EQM, "C" =COTS	R/L
"DD"	Part of test	"00" = test set up, "01" = first radiance level …	00-12

1. **[D]** BH Look up the daily calibration schedule and record the scheduled start and end time of this test on the cover page of this document. Also, fill out and double-check the other information on the cover page. 8:15
2. **[D]** BH Ensure that all supplemental manuals are on hand. These are,
   - Validator_Manual, Documentarian_Manual,
   - MastcamZCalPlan
3. **[D]** BH Ensure that the Image Log is present and ready to use. Find and open the Google Sheets file "Image_Log_46". The duration is 2 minutes. There is a link on the Wiki.
4. **[V]** BH Check that all *Calgorithms* fast-look and validation scripts are present, up-to-date, and ready to analyze test output.

Date 5/8/19 Time 3:40p Initials BH

5. **[O]** BH Check that all camera scripts required for this test are present, up-to-date and ready to command the ground support equipment (GSE). These are,
   - **491TAMBL00 - 491TAMBL12, 471TAMBL00 - 471TAMBL27, 471TAMBL30**
   - **491TAMBR00 - 491TAMBR12, 471TAMBR00 - 471TAMBR27, 471TAMBR31**
6. **[O,V,D, L]** Notes:

___

___

___

Date 5/8/16 Time 3:40p Initials BH

Hardware Installation

This procedure is for the ambient cleanroom testing at MSSS. Figures 1, 2 and 3 show the nominal layout of the cleanroom, workspace, Mastcam-Zs, ground support equipment (GSE), targets, sources, and other equipment necessary.

7. **[T,O, L]** BH Ensure that all personnel in the cleanroom are following the cleanroom practices for electrostatic discharge, proper clothing, and other safety concerns.
8. **[T]** BH Double check that ionized air is flowing over the Mastcam-Zs.
9. **[T]** BH Install the blue and infrared bright lamps and position them to the front right of the target and out of the camera's field of view (FOV) and as far as possible. Power them on and optimally position them. The lighting placement is shown in Figures 1 and 2.
10. **[O,T]** BH Ensure that the camera unit and GSE wires are secure, kink-free, and do not present tripping hazards when the lights are turned off.
11. **[O,D]** BH Check the camera temperature and ensure nominal operation.
12. **[D]** BH Record the following environmental information:
    - Cleanroom temperature 69.4°F pressure ______ humidity 59% AM (~9 am)
13. **[O,D, L]** Notes: 69.2°F 57% PM (2:43pm)

____

____

____

Date [illegible] Time [illegible] Initials [illegible]

Table 2. The nominal target placement scenes for the Geological Board and EPO testing.

Scenes	Target Placements	Notes
**Scene 1**	Centered on the rotated **Cal. Target** at 1.4 meters centered on the **Left** Mastcam-Z's boresight	1.26m
**Scene 2**	Centered on the rotated **Cal. Target** at 1.4 meters centered on the **Right** Mastcam-Z's boresight	1.26m
**Scene 3**	Centered on the **Geoboard** placed about 2 meters away in the 34mm shared boresights and filling the field of view	34/63/100 @ 3.28m
**Scene 4**	Centered on the **Geoboard** ~~placed about~~ 3 meters ~~away in the~~ 63mm shared ~~boresights~~ and filling the field of view	
**Scene 5**	Centered on the **Geoboard** ~~placed~~ about 4 meters ~~away in~~ the 100mm shared ~~boresights~~ and filling the field of view	
**Scene 6**	Centered on the **3D Validation target** about 2 meters away in the 63mm shared boresights and filling the field of view	
**Scene 7**	~~Centered on the middle-left of the Geoboard about 2 meters away in the 63mm shared fields of view~~	
**Scene 8**	Group picture with **Jim Bell** in the cleanroom holding the 2x2 foot mirror reflecting the rest of the team in the window	

Date 5/8/19 Time [illegible] Initials BH

Figure 1. MSSS Test Floor Plan for Scene 1 Imaging

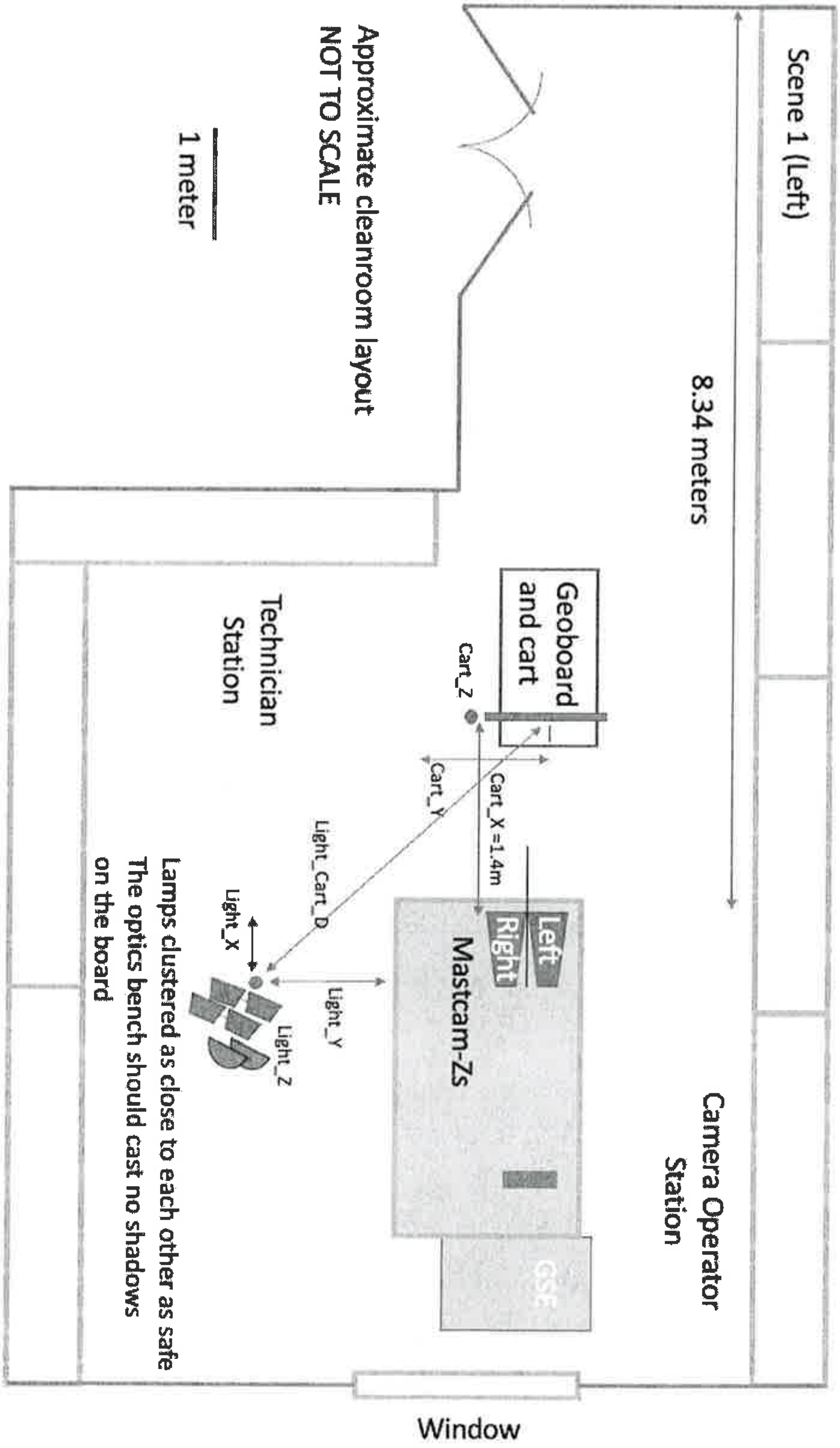

Date 5/8/19 Time 3:40p Initials BH

Scene 1 for the Left Mastcam-Z

14. [M,T] BH Position the Geoboard target to Scene 1 as described in Table 2. Make sure that the Caltarget is rotated 90 degrees towards the lights.

15. [O,T] BH Capture a test frames at 100mm with filter 0. Center the target on the boresight while taking test images as needed. Use prefix **491TAMBL00.** exp time → cut in half

16. [D,T] BH Take pictures the target position, lighting, and the whole camera/GSE set-up.

17. [O,T] BH Load and execute the script **467TAMBL03**, which captures frames with filter 0 at 70 focal lengths keeping in focus at about 1.4 meters. Insert note "TARGET= CALTARGET". The estimated duration is 12 minutes. assumed that approx. zoom is ok

(margin note: Why images? focus stack)

18. [O,V, L] Edit script **471TAMBL30** for **100mm** and execute to capture a manual z-stack focus values around filter 0's best focus for all filters. Insert note "TARGET= CALTARGET". The estimated duration is 10 minutes.

19. [D] BH Record image names and parameters in Image Log.

20. [M,T] BH Before moving the target, measure all labeled measurements in Figure 1 and annotate the figure. Take one last picture the target and light positions.

21. [D, L] Notes:

Some saturation

visibly looked fine, so might be ok?

cal target on side

light elevated

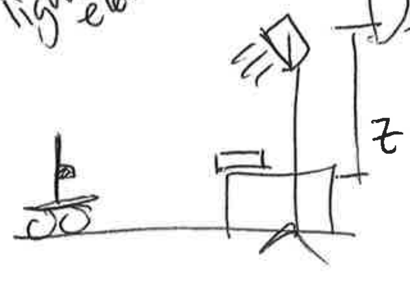

Determined actual distance

at ~1.2m

(Bell et al., 2017)

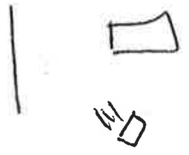

Moved cal target between L/R

Date 5/8/19 Time 3:44? Initials BH

Figure 2. MSSS Test Floor Plan for Scene 2 Imaging

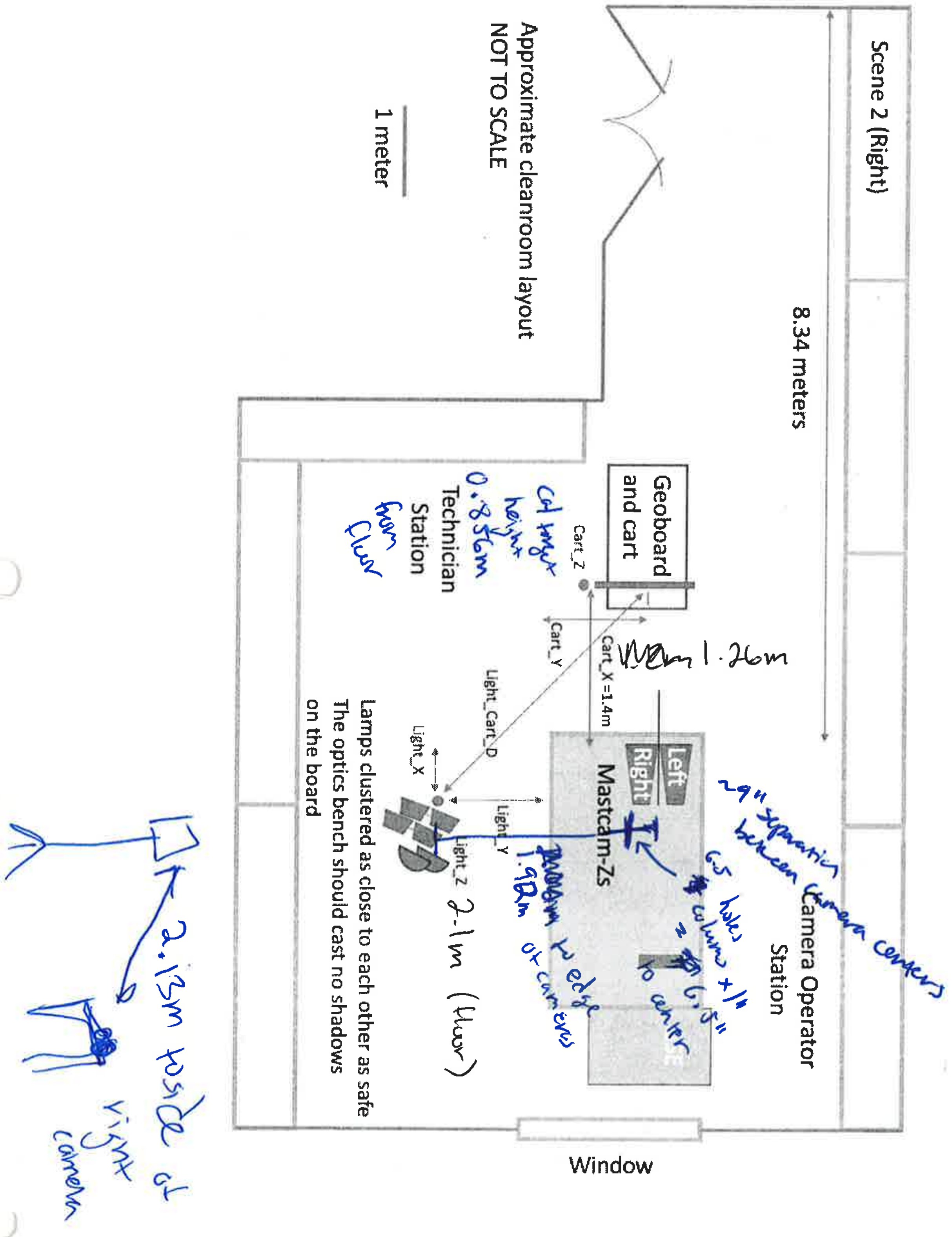

Date 5/8/19 Time 3:14p Initials BH

Scene 2 for the Right Mastcam-Z

22. **[M,T]** BH Position the Geoboard target to Scene 2 as described in Table 2. Make sure that the Caltarget is rotated 90 degrees towards the lights.

23. **[O,T]** BH Capture a test frames at 100mm with filter 0. Center the target on the boresight while taking test images as needed. Use prefix **491TAMBR00.**

24. **[D,T]** BH Take pictures the target position, lighting, and the whole camera/GSE set-up.

25. **[O,T]** BH Load and execute the script **467TAMBR03**, which captures frames with filter 0 at 70 focal lengths keeping in focus at about 1.4 meters. Insert note "TARGET= CALTARGET". The estimated duration is 12 minutes.

26. **[O,V, L]** Edit script **471TAMBR31** for **100mm** and execute to capture a manual z-stack focus values around filter 0's best focus for all filters. Insert note "TARGET= CALTARGET". The estimated duration is 15 minutes.

27. **[D]** BH Record image names and parameters in Image Log.

28. **[M,T]** BH Before moving the target, measure all labeled measurements in Figure 2 and annotate the figure. Take one last picture the target and light positions.

29. **[D, L]** Notes:____

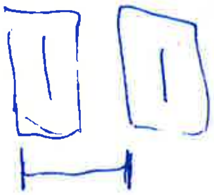

target names not all correct e.g. geoboard should be cal target

Date 5/8/19 Time 3:40p Initials BH

Figure 3. MSSS Test Floor Plan for Scenes 3-8 Imaging

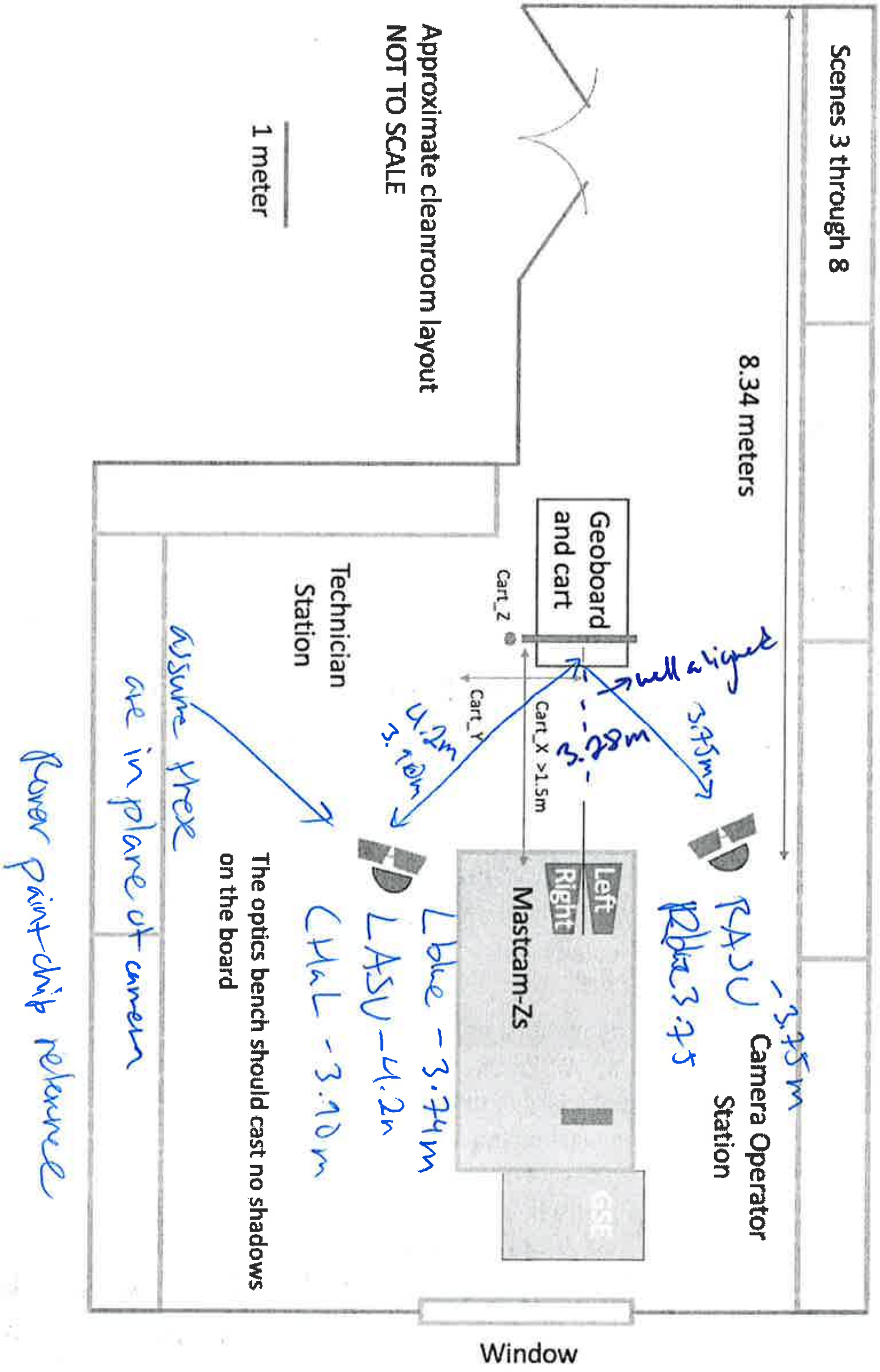

Date 3/8/19 Time 340p Initials BH

~~30. [T] ____ Rotate the Caltarget right side up.~~
~~31. [T] ____ Distribute the lights across the room to maximize diffuse illumination.~~
~~32. [T, L] Notes: ______________________________________~~

____

____

(1)

Scene 3 for the Left and Right Mastcam-Zs

33. **[M,T]** BH Position the Geoboard target to Scene 3 as described in Table 2. Make sure that the Caltarget is right side up.

34. **[O,T]** BH Capture a test frames with both cameras at 34mm with filter 0. Center the target on the boresight while taking test images as needed. Use prefix **491TAMBL00** and **491TAMBR00.**

35. **[O,V, L]** Load and execute ~~**491TAMBL05**~~ 491TAMBR13 for the **34mm** (→ 34/63/100) focal length to capture auto-exposed frames in all non-solar filters. Insert note “TARGET= GEOBOARD”. The estimated duration is 10 minutes.

36. **[O,V, L]** Load and execute ~~**491TAMBR05**~~ 491TABR13 for the **34mm** focal length to capture auto-exposed frames in all non-solar filters. Insert note “TARGET= GEOBOARD”. The estimated duration is 10 minutes.

37. **[D]** Record image names and parameters in Image Log.

38. **[M,T]** ____ Before moving the target, measure all labeled measurements in Figure 3 and annotate the figure. Take one last picture the target and light positions.

39. **[V]** Run fast-look script to verify that the required data were obtained.

40. **[D, L]** Notes: two sets acquired: lighting halogen only + halogen/blue

no bias images on R blue/halogen

need to take

Date 3/8/19 Time 3:40p Initials BH

Scene 4 for the Left and Right Mastcam-Zs

41. **[M,T]** ____ Position the Geoboard target to Scene 4 as described in Table 2.

42. **[O,T]** ____ Capture a test frames with both cameras at 63mm with filter 0. Center the target on the boresight while taking test images as needed. Use prefix **491TAMBL00** and **491TAMBR00**.

43. **[O,V, L]** Load and execute **491TAMBL06** for the **63mm** focal length to capture auto-exposed frames in all non-solar filters. Insert note "TARGET= GEOBOARD". The estimated duration is 10 minutes.

44. **[O,V, L]** Load and execute **491TAMBR06** for the **63mm** focal length to capture auto-exposed frames in all non-solar filters. Insert note "TARGET= GEOBOARD". The estimated duration is 10 minutes.

45. **[D]** Record image names and parameters in Image Log.

46. **[M,T]** ____ Before moving the target, take one last picture the target and light positions.

47. **[V]** Run fast-look script to verify that the required data were obtained.
48. **[D, L]** Notes: ____

____

____

Scene 5 for the Left and Right Mastcam-Zs

49. **[M,T]** ____ Position the Geoboard target to Scene 5 as described in Table 2.

50. **[O,T]** ____ Capture a test frames with both cameras at 100mm with filter 0. Center the target on the boresight while taking test images as needed. Use prefix **491TAMBL00** and **491TAMBR00.**

51. **[O,V, L]** Load and execute **491TAMBL07** for the **100mm** focal length to capture auto-exposed frames in all non-solar filters. Insert note "TARGET= GEOBOARD". The estimated duration is 10 minutes.

52. **[O,V, L]** Load and execute **491TAMBR07** for the **100mm** focal length to capture auto-exposed frames in all non-solar filters. Insert note "TARGET= GEOBOARD". The estimated duration is 10 minutes.

53. **[D]** Record image names and parameters in Image Log.

54. **[M,T]** ____ Before moving the target, take one last picture the target and light positions.

55. **[V]** Run fast-look script to verify that the required data were obtained.
56. **[D, L]** Notes: ____

____

____

Date 5/8/16 Time 2:45p Initials BH

Scene 6 for the Left and Right Mastcam-Zs

57. **[M,T]** BH Position the Geoboard target to Scene 6 as described in Table 2.

58. **[O,T]** BH Capture a test frames with both cameras at ~~100mm~~ 63 with filter 0. Center the target on the boresight while taking test images as needed. Use prefix **491TAMBL00** and **491TAMBR00.**

491TAMBL06

59. **[O,V, L]** Load and execute script **~~491TAMBL08~~** to capture auto-focused images with filters 0 and ~~1~~ for ~~three~~ focal lengths. Insert note "TARGET= VALTARGET". The estimated duration is 5 minutes.

491TAMBR06

60. **[O,V, L]** Load and execute script **~~491TAMBR08~~** to capture auto-focused images with filters 0 and ~~1~~ for ~~three~~ focal lengths. Insert note "TARGET= VALTARGET". The estimated duration is 5 minutes.

61. **[D]** Record image names and parameters in Image Log.

62. **[M,T]** ____ Before moving the target, take one last picture the target and light positions.

63. **[V]** Run fast-look script to verify that the required data were obtained.
64. **[D, L]** Notes: ____________________

got confused by scene numbers

used prefix from 7

Date 3/8/21 Time 3:45p Initials BH

65. **[L]** Skip this scene if we are running behind time, and everyone is ready for the group photos.

Scene 7 for the Left and Right Mastcam-Zs

66. **[M,T]** BH Position the Geoboard target to Scene 7 as described in Table 2.

67. **[O,T]** BH Capture a test frames with both cameras at 63 63mm with filter 0. Center the target on the boresight while taking test images as needed. Use prefix **491TAMBL00** and **491TAMBR00.**

68. **[O,V, L]** Load and execute script **491TAMBL06** to capture auto-focused images with filters 0 through 6 for **63mm** focal lengths. Insert note "TARGET= GEOBOARD". The estimated duration is 7 minutes.

69. **[[[redacted],V, L]** Load and execute script **491TAMBR06** to capture auto-focused images with filters 0 through 6 for **63mm** focal lengths. Insert note "TARGET= GEOBOARD". The estimated duration is 7 minutes.

70. **[D]** Record image names and parameters in Image Log.

71. **[M,T]** BH Before moving the target, take one last picture the target and light positions.

72. **[V]** Run fast-look script to verify that the required data were obtained.

73. **[D, L]** Notes: ______________________________

accomplished in Scene 3 imaging
(L0/R0 w/ different lighting
conditions)

Date 3/8/19 Time 3:45p Initials BH

Scene 8 for the Left and Right Mastcam-Zs

74. **[M,T]** BH Position the Geoboard target to Scene 8 as described in Table 2.

75. **[O,T]** BH Capture a test frames with both cameras at ~~63mm~~ 34 with filter 0. Center the target on the boresight while taking test images as needed. Use prefix **491TAMBL00** and **491TAMBR00.**

76. **[O,V, L]** Load and execute script **465TAMBL02** to single images of the group at 34mm focal lengths filter 0 and 3 meters focus. Insert note "TARGET= GROUP". Capture these images manually from the GUI if necessary.

77. **[O,V, L]** Load and execute script **465TAMR02** to single images of the group at 34mm focal lengths filter 0 and 3 meters focus. Insert note "TARGET= GROUP". Capture these images manually from the GUI if necessary.

78. **[D]** Record image names and parameters in Image Log.

79. **[V]** Run fast-look script to verify that the required data were obtained.

80. **[D, L]** Notes: ______________________________

____

____

Shutdown Procedure

81. **[D,T]** BH Take pictures of the test setup.
82. **[D,O]** BH Review entries in Image Log, GSE command log, and image headers.
83. **[D, L]** BH Review calibration procedure and ensure that each task is initialed.
84. **[D, L]** Notes: ______

85. **[V, L]** BH Before making the decision to break down the test setup, ensure that adequate data were acquired for the test requirements. See "MastcamZCalPlan" for these requirements.
86. **[V]** Notes: Some saturation on the central ring of the cal target during filters / zoom sweeps on left eye [illegible]

Data Validator (signature) [signature]

Date 5/8/19 Time 3:20 PM

87. **[V, L]** BH Give the go/no-go decision. Have enough data been acquired to fulfill test requirements? See "MastcamZCalPlan" for these requirements.
88. **[D, L]** BH Update the Log Document.
89. **[L]** Notes: ______

Calibration Lead (signature) [signature]

Date 5/8/19 Time 3:20 pm

Date 5/8/19 Time 3:45p Initials BH

90. [[redacted], L] BH Ensure that the camera and GSE are in a safe state.
91. [O,D] BH Review the Image Log with the documentarian. Exchange high-fives.
92. [[redacted]] Notes: ____

Camera Operator (signature) [signature]

Date 5/8/19 Time 3:30 pm

93. [T] CT If the next test does not require the target, position it away from the chamber or bench. Otherwise, be sure not to move it. The next test is Done with Cal.
94. [T] CT Ensure that all other test equipment is safely put away.
95. [T] Notes: ____

Technician (signature) [signature]

Date 5/8/19 Time 15:30

96. [D, L] ____ Double-check this procedure and ensure that the top of each page has valid data, time and initials.
97. [D] ____ Photo-scan this document, save it on the cloud, and file the hard-copy in the Log Binder. Upload the digital pictures taken during this test in the appropriate archive on the cloud. The required links are on the Wiki.
98. [D] ____ Double-check that every required cell the Image Log is accurately filled. When this is complete, print the Image Log and file it the Log Binder after this document.
99. [D] Notes: 454 - 459 Non rotated
46~~8~~5 - 480 Rotated

Documentarian (signature) [signature]

Date 5/8/19 Time 15:57

Date 5/8/19 Time 3:35pm Initials [illegible]

Table 2. The nominal target placement scenes for the Geological Board and EPO testing.

Scenes	Target Placements	Notes
12:45 Scene 1	Centered on the rotated **Cal. Target** at 1.4 meters centered on the **Left** Mastcam-Z's boresight	100mm center??
12:15 Scene 2	Centered on the rotated **Cal. Target** at 1.4 meters centered on the **Right** Mastcam-Z's boresight	
Scene ~~3~~ 1 abc	Centered on the **Geoboard** placed about ~~2~~ meters away in the 34mm shared boresights and filling the field of view	34/63/100
~~Scene 4~~	~~Centered on the **Geoboard** placed about 3 meters away in the 63mm shared boresights~~ and filling the field of view	optional
Scene ~~5~~ 5	Centered on the **Geoboard** ~~placed about 4 meters away~~ in the 100mm shared ~~boresights~~ and filling the field of view	
Scene ~~6~~ 3	Centered on the **3D Validation target** about 2 meters away in the ~~63mm~~ 100 63 shared boresights and filling the field of view	~~add, image of star~~ column w L0/R0
Scene ~~7~~ 3	Centered on the ~~**Color Rock target**~~ or **Xrite Checker** ~~target about~~ 2 meters ~~away~~ in the 63mm shared fields of view	→ acq. in ① 100? filters?
Scene ~~8~~ 4	Group picture with **Jim Bell** in the cleanroom holding the 2x2 foot mirror reflecting the rest of the team in the window	

3D positions of cameras, lights, target

64cm closest approach
